# Global Protein Conjugation by Ubiquitin-Like-Modifiers during Ischemic Stress Is Regulated by MicroRNAs and Confers Robust Tolerance to Ischemia

**DOI:** 10.1371/journal.pone.0047787

**Published:** 2012-10-18

**Authors:** Yang-ja Lee, Kory R. Johnson, John M. Hallenbeck

**Affiliations:** 1 Stroke Branch, National Institute of Neurological Disorders and Stroke (NINDS), National Institutes of Health (NIH), Bethesda, Maryland, United States of America; 2 Bioinformatics Section, Information Technology & Bioinformatics Program, Division of Intramural Research (DIR), National Institute of Neurological Disorders and Stroke (NINDS), National Institutes of Health (NIH), Bethesda, Maryland, United States of America; University of Muenster, Germany

## Abstract

Hibernation torpor provides an excellent model of natural tolerance to ischemia. We have previously shown that massive global SUMOylation occurs during hibernation torpor in ground squirrels. We have also shown that overexpression of Ubc9, SUMO-1, or SUMO-2/3 provides protection against ischemic damage in cell lines and cortical neurons exposed to oxygen/glucose deprivation, and in mice exposed to middle cerebral artery occlusion. We have now extended our study to other Ubiquitin-Like- Modifiers (ULMs), which have multiple cellular functions during stress, in order to assess the possibility that they also have roles in tolerance to ischemia. We found that not only SUMO conjugation, but also global protein conjugation by other ULMs including NEDD8, ISG15, UFM1 and FUB1 were significantly increased in the brains of hibernating ground squirrels during torpor. By means of miRNA microarrays of ground squirrel brain samples (from active and torpor phase) we found that the miR-200 family (miR-200a,b,c/miR-141/miR-429) and the miR-182 family (miR-182/miR-183/miR-96) were among the most consistently depressed miRNAs in the brain during the torpor phase as compared to active animals. In addition, we showed that these miRNAs are involved in the expression of various ULM proteins and their global conjugation to proteins. We observed that inhibition of the miR-200 family and/or miR-182 family miRNA activities in SHSY5Y cells increases global protein conjugation by the above ULMs and makes these cells more tolerant to OGD-induced cell death. This is the first report to describe that the natural tolerance to brain ischemia in hibernators is linked to regulation by microRNAs of a broad range of ubiquitin-like modifiers.

## Introduction

Hibernation is an excellent model of natural tolerance to ischemia. During torpor, hibernating animals lower their energy consumption, blood flow and body temperature to otherwise lethal levels, but because of special adaptive changes, suffer no CNS damage or cellular loss despite levels of brain blood flow that are characteristic of the ischemic core of a stroke [1]. We reported earlier that massive global SUMOylation, a form of post-translational protein modification with the Small Ubiquitin-like MOdifer (by both SUMO-1 and SUMO-2/3), occurs during hibernation in the brains of 13-lined ground squirrels (*Ictidomys tridecemilineatus*) [2]. We also showed that global SUMOylation is involved in ischemic tolerance in human neuroblastoma cells, primary cortical neuronal cultures from rats and mice [3], and mouse brains [4]. We generated mice with a Ubc9 transgene (Ubc9 is the sole E2 conjugase for SUMOs), and these transgenic mice indeed showed an increased level of global SUMO-conjugation and increased tolerance to middle cerebral artery occlusion (MCAO), a focal preclinical stroke model [4]. However, the observed increases in global SUMOylation were modest and the ischemic tolerance levels in these transgenic mice, although clearly enhanced, did not reach the degree of tolerance to brain hypoperfusion found in hibernating ground squirrels. It is possible that SUMOylation levels were not high enough in these transgenic mice to reach the full protection seen in hibernators, but it is also possible that additional factors in combination with SUMOylation might be participating.

Here, we first examined the role in ischemic tolerance of other ubiquitin-like protein modifiers (ULMs), of which at least 10 are known in mammals [5]. Even though the sequence homology is not very high, the family of ULMs have a common 3D structure, the ubiquitin fold, and a C-terminal glycine residue, with a carboxy group that is the site of attachment to the lysine residue of substrate proteins via isopeptide bond formation [5]. Conjugation of ULMs changes the surface topography of a substrate protein, which may facilitate or inhibit the binding of the protein to another molecule, affect enzyme activity, subcellular localization, or other functions (reviewed in [5]).

Next, we examined microRNA profiles. MicroRNAs are small noncoding RNAs that regulate a broad spectrum of genes and are believed to be one of the most powerful regulators of gene expression in complex cellular processes (reviewed in [6]). We conducted microRNA (miRNA) microarray assays of brain samples from active and torpor phase ground squirrels in order to see whether any miRNA(s) are expressed differentially between the two states. Since ground squirrel miRNAs were not among the available probe sets that represented 131 different organisms, the detection (hybridization) levels were very low, which made the determination of fold-changes or significance of the changes between the two groups very difficult. However, we found that the detection level of some miRNAs from a wide range of species were consistently lower (and others were consistently higher) in the brain samples from torpor phase ground squirrels compared to active animals, suggesting that these results can be reliable.

Here we show that not only global SUMO conjugation, but also global conjugation of other ULMs including NEDD8, ISG15, UFM1 and FUB1 were significantly increased in the brains of hibernating ground squirrels during torpor. We also report that two miRNA families, miR-200 family (miR-200a/miR-200b/miR-200c/miR-141/miR-429) and miR-182 family (miR-182/miR-183/miR-96), which were consistently lower in the brain samples from torpor phase ground squirrels compared to active animals, are involved in expression of various ULM proteins and their global protein conjugation. We also show in SHSY5Y cells that inhibition of miR-200 family and/or miR-182 family miRNA activity makes cells more tolerant to oxygen/glucose deprivation (OGD), perhaps via an increase in post-translational modification of cellular proteins by various ULM species.

## Materials and Methods

### Animal Preparation

Thirteen-lined ground squirrels, *I. tridecemlineatus*, were captured by USDA-licensed trappers (TLS Research, Bartlett, IL, USA). Experiments were approved by the NINDS Animal Care and Use Committee. Both male and female ground squirrels were used equally for this study, and all animals were between one and three years of age, but because the animals were caught from the wild there is no way to know the exact age of the animals. Ground squirrels were housed, fed and induced to hibernate as described in the previous paper [2]. Animals in six different phases of the hibernation bout (cycle) were differentiated by body temperature (Tb), time, and respiratory rate as described previously [2]. ACR: active for 4 to 5 days in the cold chamber with an ambient temperature of 4°C to 5°C (Tb = 34°C to 37°C); EN: entrance phase of hibernation (Tb = 31°C to 12°C); ET: early torpor phase (1 day) (Tb = 5°C to 8°C); LT: later torpor phase (>5 days) (Tb = 5°C to 8°C); AR: arousing from torpor spontaneously with a respiratory rate >60/min and a persistent low body temperature (Tb = 9°C to 12°C); IB (interbout): aroused from a torpor state and returned to a normal metabolic state for 7 h (Tb = 34°C to 37°C). At each time point, brain was removed quickly, frozen instantly in 2-methylbutane (−50°C), and stored at −70°C until use. Six animals were used for each time point.

### Western Blot Analysis

Frozen whole brain was crushed on dry ice to make powder, added to the cold buffer containing 2% SDS, 60 mM Tris-HCl (pH 6.8), protease inhibitor cocktail (Roche), 50 mM EDTA, 2.5 mM sodium pyrophosphate, 1 mM PMSF and 25 mM N-ethylmaleimide, and homogenized on ice. The homogenates were sonicated for 10 to 15 sec, boiled for 5 min at 95°C and centrifuged at 15,000 *g* for 10 min at 4°C. After protein concentrations were measured, supernatants were boiled again with 5% -mercaptoethanol and 2% glycerol, then subjected (20 µg/lane) to SDS-PAGE (4–20%). Western blot analyses were performed using the following antibodies: rabbit polyclonal anti-SUMO-1 antibodies raised against the processed form of human SUMO-1 (in-house), rabbit polyclonal anti-SUMO-2/3 antibodies (in-house), rabbit monoclonal anti-Ubc9 (abcam), rabbit monoclonal anti-ISG15 (Epitomics), rabbit polyclonal anti-NEDD8 (Cell Signaling), mouse monoclonal anti-FUB1 (Abnova), rabbit monoclonal anti-UFM1 (Epitomics), mouse monoclonal anti-ubiquitin (Zymed), and mouse monoclonal anti-β-actin (Sigma). These antibodies were used at 1∶1000 dilutions, except anti-β-actin which was used at 1∶10,000.

**Figure 1:**
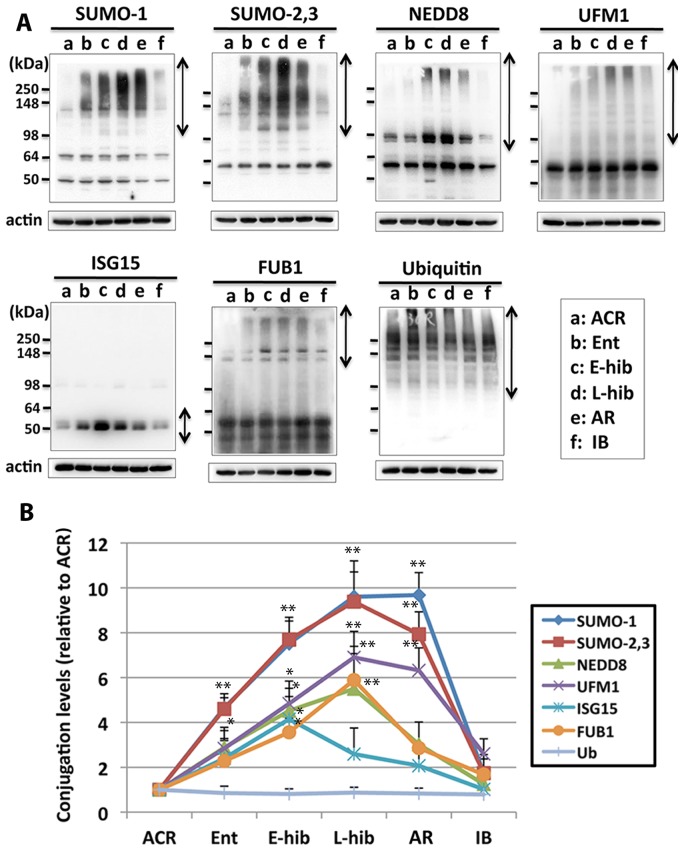
Protein Conjugation by various ULMs increases during hibernation torpor in the brains of 13-lined ground squirrels. (A) Representative Immunoblots of various ULMs in the brain samples from different stages of hibernation cycle. Arrow in each panel shows the molecular weight range of conjugates of the ULM indicated on top of the panel. a: ACR (active in the cold room); b: Ent (entrance); c: E-hib (early hibernation); d: L-hib (late hibernation); e:AR (arousal); f: IB (interabout). β-actin served as a loading control. Each immunoblot is representative of at least three different brain samples at each time point. (B) Quantitative analyses of the conjugates of various ULMs from three independent experiments. Cropped areas are shown by arrows; densities were measured, normalized by corresponding β-actin level, and shown as the ratio to ACR samples. Data represent the mean±SD of three independent experiments. **p<0.01, *p<0.05 compared to ACR.

Intensities of bands were analyzed by the Macintosh densitometry program ImageJ (NIH). For conjugate band analysis, the higher molecular weight area (indicated with arrows in Figure 1A) in each lane was cropped and analyzed.

### Sample Preparation for microRNA Microarray processing

Total RNA including small RNAs in each squirrel brain were purified according to the protocol supplied with the miRNeasy Mini Kit (Qiagen) and DNase treatment was included as part of the isolation to remove possible contaminating DNA. The Bioanalyzer nanochip (Agilent Technologies, Santa Clara, CA, USA) and NanoDrop (Thermo Scientific, Wilmington, DE, USA) were used to assess the quality and quantity of the RNA prior to labeling. 2 µg of total RNA was used per sample in conjunction with the 3DNA array detection FlashTag Biotin microRNA labeling kit (HSR10FTA) from Genisphere, Inc. Biotin labeled microRNAs were hybridized on the GeneChip® miRNA 2.0 Arrays, washed, stained and scanned on an Affymetrix platform (Affymetrix, Inc). Six animal samples were used for each condition .

### Microarray Data Analysis

The miRNA QC Tool Version 1.1.1.0 (Affymetrix, Inc) was used for miRNA expression summarization and normalization. Afterward, the statistical programming language R (http://cran.r-project.org/) was used. First, data quality was confirmed by Tukey box plot, covariance-based Principal Component Analysis (PCA) scatter plot and correlation-based Heat Map. Then, system noise was defined as the lowest observed mean expression value at which the LOWESS fit of the data (CV∼mean) changes from non-linear to linear. miRNAs not having at least one expression value across all samples greater than system noise were deemed non-informative and excluded from further analysis. Expression for the remaining miRNA were floored to system noise if less than system noise and the fold-difference between means for LH and ACR were calculated. miRNA having an absolute mean difference ≥1.25 were grouped in subsets regardless of species by parent miRNA family. For each miRNA family represented, the total number of miRNAs grouped therein was determined along with the number of miRNAs that were up-regulated vs. down-regulated. Results were summarized by bar plot.

### microRNA quantification

Total RNA including small RNAs, from ground squirrel brains were purified according to the protocol supplied with the miRNeasy Mini Kit (Qiagen), and miRNAs from cultured cells were isolated by mirVana miRNA isolation kit (Ambion). Polyadenylation of miRNAs from total RNA (or miRNA) and synthesis of first-strand cDNA from the tailed miRNAs for use in real-time PCR were carried out using NCode miRNA First-Strand cDNA Synthesis and qRT-PCR Kits (Invitrogen). Then, miRNA levels were analyzed by qPCR using the SYBR-Green qPCR (Qiagen)/MyiQ (BioRad) detection system. Mature sequence (miRbase, http://www.mirbase.org/ftp.shtml) of individual miRNA was used for forward primer and universal primer (from the kit) was used for reverse primer in each qPCR reaction. miRNA expression levels in each sample were normalized to miR-103, which was among the most stably expressed (expression levels were not changed during hibernation bout) miRNAs in ground squirrel brains. Relative expression was calculated using the comparative Ct method (2^−ΔΔCt^) [7].

### Transfection of miRNA mimics and/or inhibitors

Human neuroblastoma SHSY5Y cells were transfected with miRNAs (miR200c, miR183, miR182, miR141, miR-96) mimics or inhibitors (Thermo Scientific Dhamacon miRIDIAN microRNA Mimics & Hairpin Inhibitors) using Lipofectamine RNAimax (Invitrogen). Forty ∼120 nM (final concentration) of mimics or inhibitors were used in each transfection. Typically, two days after each transfection, miRNA levels (by qPCR) and/or protein levels (by Western blot) were examined.

### Reporter plasmid construction and reporter assay

Oligonucleotide pairs were designed to contain putative miRNA target sequences, with proper overhangs and an internal NotI site for clone confirmation. Annealing and ligating these pairs into the pmirGLO vector (Promega), maintained the miRNA target region in the correct 5′ to 3′ orientation. The oligonucleotide sequences used for each reporter plasmid construction are listed in Table 1. Oligonucleotide pairs (sense and antisense) were annealed (90°C for 3 min, cooled to 37°C over 30 min and kept at 4°C for 60 min), ligated with the PmeI-XbaI digested and gel-purified pmirGLO vector, and transformed into *E.coli* DH5α. Positive clones were identified by digesting miniprep –purified DNA with NotI (positive clones give a 125 bp insert). SHSY5Y cells were transfected with these constructs (100∼150 ng per well of 96 well plate) with or without miRNA mimics (∼50 nM) as mentioned above. All transfections were carried out in at least triplicate. Cells were lysed and luciferase activities were measured using the Dual-Glo Luciferase Assay System (Promega).

**Table 1:** Oligonucleotides used for reporter plasmid construction (Predicted miR target sequences in each gene are shown in bold letters).

Target Gene	miRNA	Sequences for oligo pairs
	miR-200b,c	5′-aaactagcggccgctagt**attttaatattgatgtcagtatttca**t-3′ (S)
		5′-ctaga**tgaaatactgacatcaatattaaaat**actagcggccgctagttt-3′ (AS)
UBE2I	miR-182	5′-aaactagcggccgctagt**tgccgctcctctctagaacct**t-3′ (S)
		5′-ctaga**aggttctagagaggagcggca**actagcggccgctagttt-3′ (AS)
	miR-183	5′-aaactagcggccgctagt**ggcatcgagaccctggcaactgcaccggtgccagct**t-3′ (S)
		5′-ctaga**agctggcaccggtgcagttgccagggtctcgatgcc**actagcggccgctagttt-3′ (AS)
	miR-200b,c	5′-aaactagcggccgctagt**tgtgaggatcccaggattcagtattc**t-3′ (S)
NEDD8		5′-ctaga**gaatactgaatcctgggatcctcaca**actagcggccgctagttt-3′ (AS)
	miR-182	5′-aaactagcggccgctagt**ttatgactgtgtccctggttgtcaat**t-3′ (S)
		5′-ctaga**attgacaaccagggacacagtcataa**actagcggccgctagttt-3′ (AS)
	miR-141	5′-aaactagcggccgctagt**attagccaactgttaactggaagctt**t-3′ (S)
UFM1		5′-ctaga**aagcttccagttaacagttggctaat**actagcggccgctagttt-3′ (AS)
	miR-183	5′-aaactagcggccgctagt**aaaagaattatggaccctggatggcaatttgc**t-3′ (S)
		5′-ctaga**gcaaattgccatccagggtccataattctttt**actagcggccgctagttt-3′ (AS)
	miR-141	5′-aaactagcggccgctagt**cactcctggactgtgactttcagtgggagatg**t-3′ (S)
SUMO-1		5′-ctaga**catctcccactgaaagtcacagtccaggagtg**actagcggccgctagttt-3′ (AS)
	miR-96	5′-aaactagcggccgctagt**ttgcataaatactggaaattgcacatggta**t-3′ (S)
		5′-ctaga**taccatgtgcaatttccagtatttatgcaag**actagcggccgctagttt-3′ (AS)
	miR-200c	5′-aaactagcggccgctagt**catcctcgcattgctgttgaatggtgagcacg**t-3′ (S)
SUMO-3		5′-ctag**acgtgctcaccattcaacagcaatgcgaggatg**actagcggccgctagttt-3′ (AS)
	miR-200c′	5′-aaactagcggccgctagt**ttcctgtttgctgtatgggctcgggtg**t-3′ (S)
		5′-ctag**acacccgagcccatacagcaaacaggaa**actagcggccgctagttt-3′ (AS)

### Cell viability assay

A premixed WST-1 cell proliferation reagent was used for cell viability assays as per the manufacturer's instructions (Clontech). The premixed WST-1 cell proliferation reagent provides a method to measure cell proliferation based on the enzymatic cleavage by mitochondrial succinate-tetrazolium reductase, which is present in viable cells, of the tetrazolium salt WST-1 to a water-soluble formazan dye that can be detected by absorbance at 420–480 nm. After a 4–6 hr incubation with premixed WST-1, the absorbances at 450 nm and a reference wavelength at 690 nm were measured. The corrected absorbance (A450–A690) was compared to a control cell group. The cell viability assay was performed in a 96-well plate format at least in triplicate in each experiment.

### Statistical analysis

Variables were analyzed by Student's *t*-test (two-tailed) and a P<0.05 was considered statistically significant. Values are expressed as mean±SD (standard deviation) of at least three independent experiments.

## Results

### Global conjugation levels of various ubiquitin-like protein modifiers (ULMs) increase in the brain of 13-lined ground squirrels during hibernation torpor

We had found that during hibernation, massive conjugation of SUMO-1 and SUMO-2/3, small ubiquitin-like protein modifiers, occurred in the brain (and many other organs) of 13-lined ground squirrels [2], and hypothesized that these increased conjugation levels were partly (if not completely) responsible for the natural tolerance to ischemia in these hibernating animals. In recent years many other ULMs have been discovered and their wide range of functions have been reported (reviewed in [5]). We wondered whether other ULMs were also involved in controlling the induction of ischemic tolerance. Namely, did conjugation levels of these ULMs change like global SUMOylation levels during hibernation torpor? We checked conjugation levels of various ULMs in the ground squirrel brain from different stages of the hibernation cycle (3–4 animals were used for each time point). As shown in the Figure 1, we found that not only SUMO conjugations, but also global conjugation of other ULMs including NEDD8, ISG15, UFM1 and FUB1 were increased in the brains of hibernating ground squirrels during torpor. Note, however, that ubiquitin conjugation was unchanged. The level of conjugation of most ULMs peaked around late hibernation except for ISG15 conjugation, which peaked in the early hibernation stage. What are the mechanisms and the possible regulators of these increases?

### miRNA Profiling

**Figure 2:**
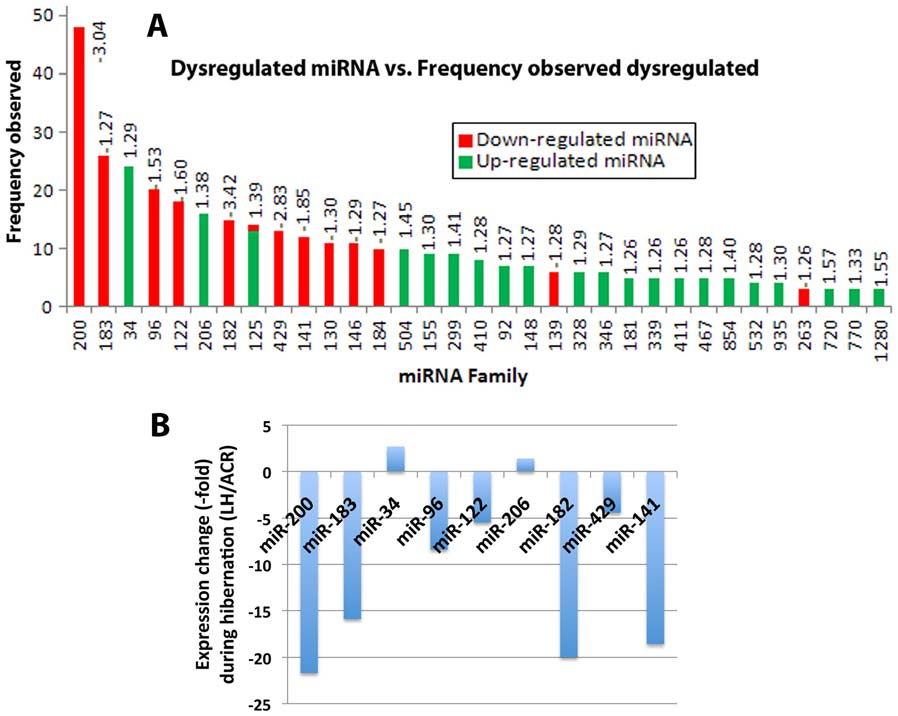
Differentially regulated miRNA families in the brains of 13-lined ground squirrels during hibernation. (A) 405 miRNAs which had an absolute mean difference between LH and ACR≥1.25 (using 6 different animal samples for each condition) were grouped into 33 families, and plotted against the frequencies observed. Down-regulated miRNAs were shown in red, and up-regulated miRNAs were shown in green. The number on top of each column is the fold change (ACR vs LH). (negative number = decrease, positive number = increase). (B) A subset of differentially regulated miRNAs detected by miRNA microarray was validated by qPCR using the same RNA samples that had been used for the miRNA microarray. The levels of the miRNAs were normalized by the level of miR-103, which was among the most stably expressed miRNAs with expression levels that did not change during the hibernation bout, and expressed as ratios of LH/ACR.

MicroRNAs are small noncoding RNAs that regulate a broad spectrum of genes post-transcriptionally particularly under stress conditions [8]. We ran microRNA arrays on brain samples from active and torpor phase ground squirrels (6 samples from each condition). 20,180 miRNA expression measurements representing 131 different organisms were generated per sample. No samples were removed as outliers. Measurements for 1,154 miRNAs were excluded as non-informative (miRNA not having at least one expression value across all samples greater than system noise were deemed non-informative), leaving 19,026 miRNA comparisons, of which 405 had an absolute mean difference in mean expression between LH and ACR≥1.25 (Table S1). For these 405 miRNAs, 33 families are represented (Fig. 2A). Of these families, miR-200 had the greatest number of differentially regulated members (n = 48) and the largest magnitude difference between means of the LH and ACR states (Δ = −3.04). Fold-differences for members within each family were found concordant 100% of the time with the exception of one family (miR-125)(Fig. 2A). In particular, two miRNA families, the miR-200 family (miR-200a, b, c, miR-496, and miR-141) and the miR-182 family (miR-182, miR-183 and miR-96) were consistently down regulated during torpor phase. In general, however, more miRNA families were upregulated during the torpor phase (Fig. 2A). We verified selected microRNA array data by reverse transcriptase (RT)-qPCR. We could confirm that miR-200, miR-182, miR-183, miR-141, miR-96, miR-122 and miR-429 were indeed down regulated, and miR-34 and miR-206 were upregulated during hibernation torpor (Fig. 2B). MiR-182, miR-96, miR-200, miR-183 and miR-141 were among the most highly regulated miRNAs with a 13∼20-fold decrease during the torpor phase of the hibernation cycle.

### Effect of overexpression or inhibition of miRNAs (miRs 200c, 182, 183, and 141) on ULM protein expressions or ULM conjugation levels in SHSY5Y cells

**Figure 3:**
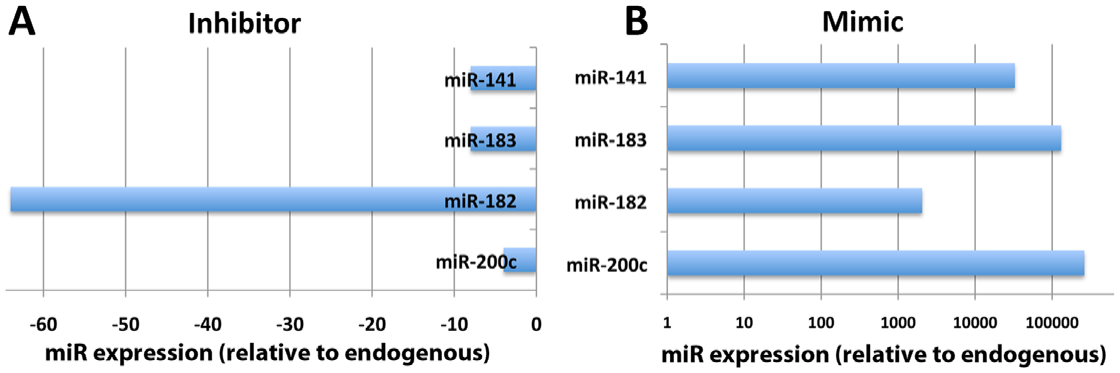
Transfection of inhibitors decrease their corresponding endogenous miRNAs and transfection of mimics increase corresponding miRNAs in SHSY5Y cells. (A) SHSY5Y cells were transfected with hairpin inhibitors for human miR-200c, miR-182, miR-183 and miR-141 individually, the levels of these miRNAs were measured by qPCR, and expressed relative (fold change) to endogenous levels. (B) SHSY5Y cells were transfected with the double stranded miRNA mimics listed on the figure individually, the expression levels were measured by qPCR and the fold changes from endogenous levels were plotted.

**Figure 4:**
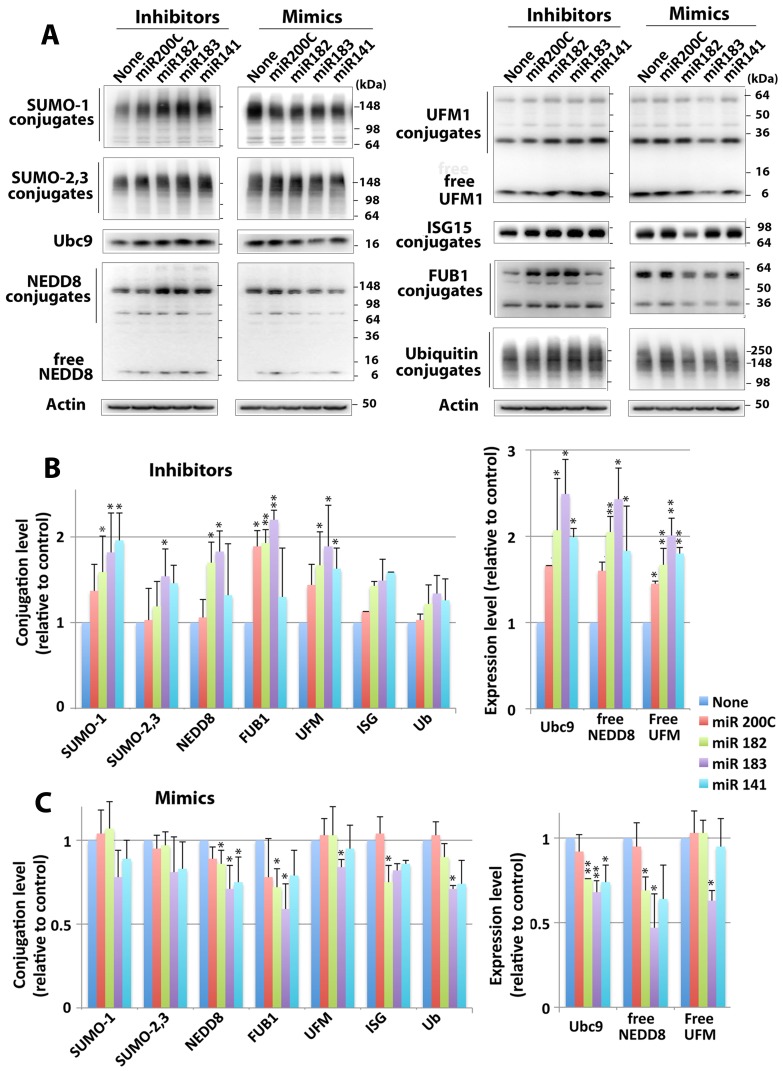
Effect of miRNA inhibitors and mimics for miR-200 family and miR-182 family members on ULM conjugation levels in SHSY5Y cells. (A) Representative Immunoblots of various ULMs in SHSY5Y cells that had been transfected with either miR inhibitors (left column) or mimics (right column) in each panel. (B) Quantitative analyses of the conjugates (left panel) or free form (right panel) of various ULMs in inhibitor-transfected SHSY5Y. (C) Quantitive analyses of the conjugates (left panel) or free form (right panel) of various ULMs in mimic-transfected SHSY5Y cells. Densities of conjugates or free forms were measured, normalized by corresponding β-actin level, and shown as the ratio to endogenous (negative control-transfected) level. Data represent the mean±SD of three independent experiments. **p<0.01, *p<0.05 compared with endogenous level.

We then used either inhibitors or mimics that were specific to the miRNAs of interest in order to examine the effects (or roles) of these miRNAs in regulating global ULM conjugation levels. We used miRIDIAN miRNA Mimics (Dharmacon), which are double-stranded RNA oligonucleotides chemically enhanced to mimic endogenous mature miRNA function, and miRIDIAN miRNA hairpin inhibitors (Dharmacon), which are single-stranded oligonucleotides designed to inhibit endogenous mature miRNA functions. As shown in Figure 3, inhibitors lowered the levels of these miRNAs (4∼64-fold compared to negative control transfected) (A), and mimics increased corresponding miRNAs levels (2000∼262,000-fold above negative control transfectants) (B). Although an ‘inhibitor’ would generally be thought not to change the level of the miRNA, but to prevent its mechanisms of action instead, the Dharmacon ‘inhibitor’ acts as a sink and reduces the availability of the miRNA, so the detected level becomes low. In many of the miRNA inhibitor assays, the active miRNA concentrations were lowered to background levels limiting the accuracy of the fold-inhibition calculation. The order of endogenous levels of the microRNAs tested in SHSY5Y cells is miR-182>>miR-141≥miR-183>miR-200c. The endogenous level of miR-182 is much higher than those of other miRNAs tested, thus showing greater apparent inhibition by its specific inhibitor, but a reduced fold increase by mimics. Conversely, the endogenous level of miR-200c is very low, so the apparent fold inhibition is low, but the fold increase by its specific mimic is huge. We transfected SHSY5Y cells with these inhibitors or mimics, and two days later ULM proteins and ULM global conjugation levels in the cells were analyzed by Western blot. As shown in Figure 4, when these miRNA expressions were inhibited by their specific inhibitors, most of the ULM conjugations examined were indeed increased. Conversely, when these miRNAs were overexpressed by their specific mimics, the conjugation levels of these ULMs were depressed. In addition, the expression of some of the free (unconjugated) ULMs (NEDD8 or UFM1) and the SUMO E2 conjugase, Ubc9, were also controlled by these miRNAs.

### Identification of potential targets of miR-200 family and miR-183/96/182 family miRNAs in ULMs and/or ULM-related genes

**Figure 5:**
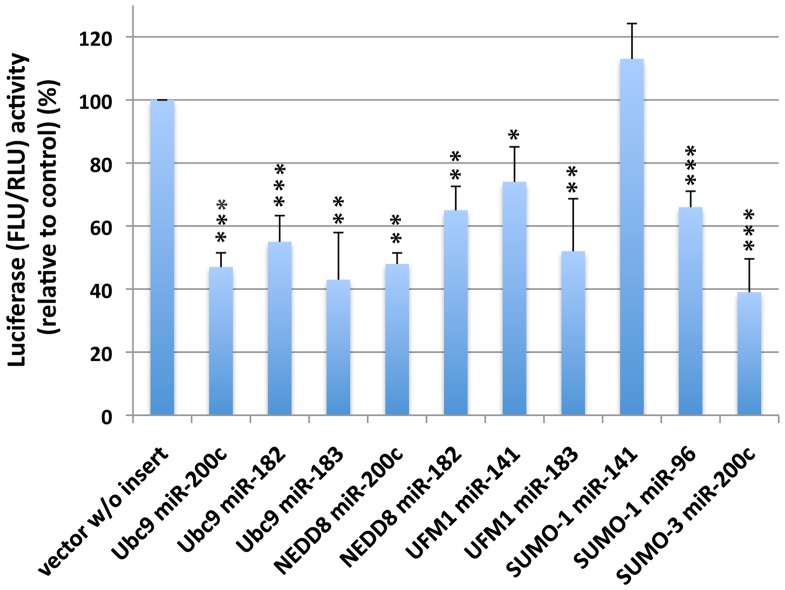
miR-200 family and miR-182 family members of miRNAs indeed target various ULM and/or ULM related genes. SHSY5Y cells were co-transfected with the pmirGLO Dual-Luciferase miRNA Target Expression (Promega) constructs into which putative target sites for the various ULMs had been inserted, and with mimics of miRNAs as indicated. Two days later, cells were lysed, luciferase acivities (both firefly and Renilla) were measured, firefly luciferase activities were normalized with Renilla firefly activity, and expressed as relative to control (transfected with empty vector and negative control mimic). Data are shown as the mean±SD of three independent experiments. ***p<0.001, **p<0.01, *p<0.05 compared to control.

In order to confirm that miR-200 family miRNAs and miR-182/96/183 family miRNAs are involved in ULM and/or ULM-related gene expression, we compiled a list of potential targets of these miRNAs in various ULM mRNAs using three computational target prediction programs: TargetScan (www.targetscan.org); MicroInspector (http://bioinfo.uni-plovdiv.bg/microinspector/); RegRNA tool (http://regrna.mbc.nctu.edu.tw/html/prediction.html). We found quite a few potential target sites for miR-200 and miR-141, and some for miR-182/183/96 in SUMO-related genes and other ULMs as shown in Table 2. miRNA::mRNA target prediction is based on assumptions of certain values for temperature, salt and other conditions and the results are all too often dependent on the algorithm employed by each tool. For example, some of the potential target sites were predicted at 32°C but not at 37°C using the MicroInspector which is the only program you can run at different temperature settings. The DNA sequences of target sites for miR-200 family and/or miR-182 family miRNAs in each gene are shown in the supplemental materials (Table S2). In order to examine whether some (if not all) of these predicted target sites are really recognized by either miR-200 family or miR-182/183/96 family miRNAs, we made firefly luciferase reporter constructs containing individual predicted target sequences for the miRNAs of interest. The pmirGLO Dual-Luciferase miRNA Target Expression Vector (Promega) was designed to quantitatively evaluate miRNA activity by the insertion of putative target sites 3′ of the firefly luciferase gene. The oligonucleotides used for these reporter plasmids are shown in Table 1 with the predicted miRNA target sequences in each gene shown in bold letters. Firefly luciferase is the primary reporter gene, and reduced firefly luciferase expression indicates the endogenous or introduced miRNA level of binding to the cloned miRNA target sequence. SHSY5Y human neuroblastoma cells were transfected with these designed constructs along with a corresponding miRNA mimic, and luciferase activities were measured. The firefly luciferase activities were normalized by Renilla luciferase activities, and shown as the percentage of control (i.e. transfected with empty vector and miRNA mimic negative control) activity (Fig. 5). As shown in the figure, each of these transfectants, except one that was transfected with the SUMO-1 miR-141 target site incorporated construct and miR-141 mimic, showed significantly reduced activities. The results strongly suggest that theses predicted target sites are real and SUMO and other ULM mRNAs are indeed targeted and controlled by the miR-200 family and the miR-182 family miRNAs.

**Table 2:** Number of potential target sites of miRNAs of interest in SUMO-related genes and other ULMs.

miRNA	miR-200	miR-182	miR-183	miR-141	miR-96	miR-122	miR-34
Gene							
**SUMO-1**				**++**	**+**		
**SUMO-2**				**++**			
**SUMO-3**	**+++**			**+**			
**SAE1**		**++**		**+**		**+**	**+**
**SAE2(UBA2)**							**+++**
**UBE2I(Ubc9)**	++*	+*	+*	++*			**+**
**SENP1**	**+**		**+**				
**SENP2**	**++**			**+**			
**SENP6**	**++++**			**+**			**++**
**UFM1**	+*	+*	++*	+*			
**ISG15**	**++**			**+**		**+**	
**FUB1**							
**NEDD8**	+*+*	+*					
**Ubiquitin**	**+++**		**+**	**+++**			**+++**

**+**: one potential target site of each miRNA to the gene indicated in the first column (predicted by MicroInspector at 32°C http://bioinfo.uni-plovdiv.bg/microinspector/)

+*: one potential target site of each miRNA to the gene indicated in the first column (predicted by RegRNA tool at 37°C (http://regrna.mbc.nctu.edu.tw/html/prediction.html)

### Effect of inhibition or overexpression of the miR-200 family and the miR-182 family miRNAs on OGD-induced cell death in SHSY5Y cells

**Figure 6:**
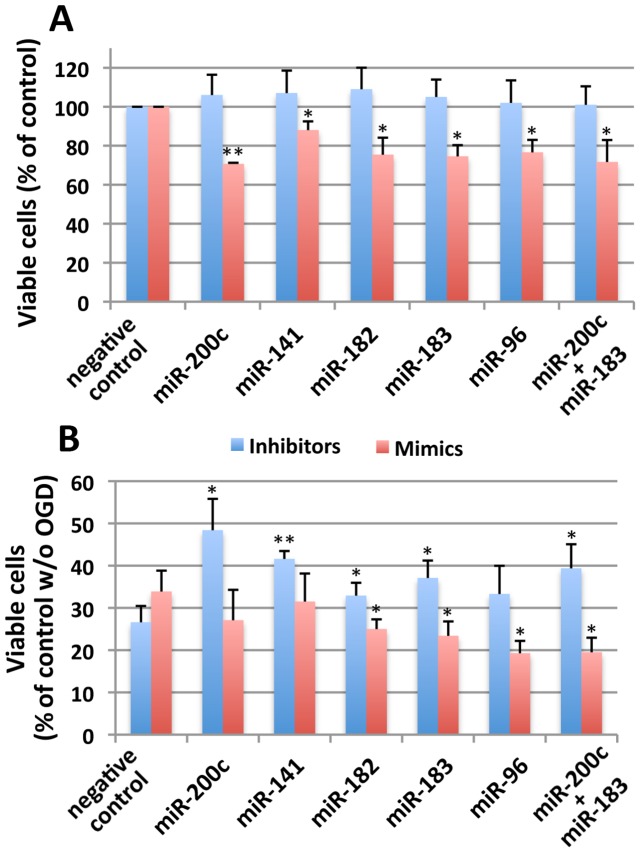
Effects of inhibitors and mimics for miR-200 family and miR-182 family miRNAs on OGD-induced cell death in SHSY5Y. (A) SHSY5Y cells were transfected with either inhibitors or mimics as indicated and 2days later the cell viability was measured by WST-1. Viability was expressed as percentage of negative control for either inhibitor or mimic transfected cells. (B) SHSY5Y cells transfected as mentioned above, and 2days later these cells were subjected to OGD (16 h), and viable cells were measured by WST-1. Viability was expressed as percentage of negative control cells (negative control inhibitor or negative control mimic transfected and not subjected to OGD). In both A and B, inhibitor transfected cells were shown in blue, and mimic transfected cells were shown in red. Data are shown as the mean±SD of three independent experiments. **p<0.01, *p<0.05 compared to control.

We have established that the miR-200 family and the miR-182 family miRNAs target SUMO and other ULMs, and that inactivation of these miRNAs increases both global SUMOylation and global conjugation of other ULMs resembling the changes in posttranslational modifications that have been noted in ground squirrels during hibernation torpor. Conversely, overexpression of these miRNAs by introducing their mimics caused a decrease in ULM conjugation levels. We wondered whether the tolerance to in vitro “ischemia” by OGD in cultured cells (e.g. SHSY5Y) would change if miR-200 family and/or miR-182 family miRNA activity levels were reduced or increased. We transfected SHSY5Y cells with inhibitors or mimics for several miR-200 family and the miR-182 family miRNAs (miR-200c, miR-141, miR-182, miR-183 and miR-96) in duplicate plates, incubated them for 2 days, and subjected one plate of cells to OGD, and the other plate of cells to normal culture conditiions as a control. As shown in Fig. 6A, without OGD the viability of cells transfected with miRNA inhibitors equaled or exceeded that of negative control transfected cells, but cells transfected with miRNA mimics showed 10∼30% lower viability than the cells transfected with a negative control inhibitor or mimic construct. OGD overnight (16 h) caused 60∼70% cell death (30∼40% in viability) in negative control transfected cells (Fig. 6B). Cells transfected with miRNA inhibitors, especially miR-200 family (miR-200c and miR-141) showed much far less cell death (Fig. 6B), but cells transfected with miRNA mimics, especially from the miR-182 family, caused more cell death (Fig. 6B). These results suggest that inhibiting miR-200 family and miR-182 family miRNAs does protect SHSY5Y cells from OGD-induced cell death. In the case of mimics, however, overexpressing these miRNAs alone caused some cell death without OGD, so the cell death after OGD might not be completely reflective of OGD-caused cell death.

## Discussion

We have studied 13-lined ground squirrels, *Ictidomys tridecemlineatus*), which rank among the most brain hypoperfusion-resistant mammals known, as a model of natural tolerance to brain ischemia. The purpose of our study is to understand the molecular mechanisms exploited by these hibernators to greatly increase their resistance to levels of blood flow that, absent such adjustments, would rapidly lead to brain cell death. These findings may guide translational efforts to develop innovative therapies for stroke. We have reported that during ground squirrel hibernation torpor, massive global SUMOylation occurs in many organs including the brain [2]. Here we also report that not only SUMOylation, but also global protein conjugation by a number of other ULMs that have received increasing attention in recent years [9], [10] are elevated during hibernation torpor. We have inferred that energy saving post-translational modifications might play a big role in regulating stress tolerance in metabolically suppressed hibernating animals. The finding that global protein conjugation by many ULMs markedly increase during hibernation torpor provides some support this hypothesis. Most ULMs were discovered during the last 10 years, and they have a broad range of functions in the biological network (reviewed in [5]). For example, ISG15 (Interferon-Stimulated Gene-15), a 15 kDa protein, was first discovered as a protein whose expression could be induced by type I interferons (hence the name); it was later recognized to function like the ubiquitin system [11]. ISG15 is a ULM that had been linked primarily to immune-defense mechanisms, but its functional significance has more recently been extended to cell growth and differentiation (reviewed in [9]) and neuroprotection [12]. NEDD8 (neural precursor cell-expressed developmentally downregulated-8) gene is one of the genes which are down-regulated in neural precursor cells during the development of the murine brain [13]. The NEDD8 gene encodes a 9-kDa protein that displays 80% sequence homology with ubiquitin, and is known to regulate cell cycle progression [14]. It mainly functions as a regulator of ubiquitin ligases leading to proteasomal degradation for a subset of proteins [15]. Less studied, but not necessarily less important, Fau ubiquitin-like protein (FUB1)/monoclonal nonspecific suppressor factor (MNSF) β has been identified as a ULM that exerts anti-inflammatory effects [16], [17], and ubiquitin-fold modifier (UFM)-1 is a ULM that has a potential role in the endoplasmic reticulum stress response [10], [18]. We haven't studied all the ULMs identified, but we have examined several ULMs including ones mentioned above and find that the global protein conjugation by most of the ULMs we have examined, with the notable exception of ubiquitin, increase during hibernation torpor in 13-lined ground squirrels in patterns that resembles the SUMO posttranslational modifications noted earlier (Fig. 1) [2]. We reasoned that these elevated ULM conjugation levels during the torpor phase of a hibernation bout can orchestrate the cellular events in hibernating ground squirrels that induce the state of natural tolerance in these animals through their multipronged effects.

We then questioned which mechanisms could possibly regulate this wide range of ULM conjugations. Comparisons of transcriptomic and proteomic changes during torpor and arousal phases of the hibernation cycle for a large number of genes [19], [20] reveal that there is substantial regulation of translation during hibernation. MicroRNA represents an important posttranscriptional regulatory mechanism for translation of individual mRNA transcripts. From the results of our microRNA array study of ground squirrel brain (comparing active and torpor phase), we found that most miRNAs were unchanged or increased during torpor phase, but certain miRNAs such as miR-200 family (miR-200a, -200b, -200c, -141, -429) and miR-182 family (miR-182, -183, -96) miRNAs were consistently decreased (Fig. 2A and Table S1). We have shown that inhibition of these miRNAs by their specific hairpin inhibitors in SHSY5Y cells increases global protein conjugation by many ULMs including SUMO-1 and SUMO-2/3 paralogs, ISG15, NEDD8, UFM1, and FUB1 (Fig. 4); overexpression of these miRNAs by transfecting SHSY5Y cells with their mimics decreased the conjugation levels (Fig. 4). These results strongly suggest the involvement of these miRNAs in the process of ULM conjugations. MicroRNA target prediction programs indicated several sites for these miRNAs in each ULM mRNA (Table 2), and the reporter assay using constructs in which the target sequences of each mRNA of these ULMs were incorporated (Table 1) confirmed the associations of these miRNAs with these ULM mRNAs (Fig. 5). Interestingly, SHSY5Y cells became more tolerant to OGD-induced cell death when activities of miR-200 family and/or miR-182 family miRNAs were inhibited (Fig. 6B), and more sensitive when mimics of these miRNAs were transfected (Fig. 6B). At the moment, we cannot ascertain whether the protection (or sensitization) was either by direct effects of these miR inhibitors (or mimics), or through miR-inhibitor-caused increase (or miR-mimics-caused decrease) of ULM conjugation.

There have been only a few reports on miRNAs in hibernators. Morin et al. [21] first examined selected miRNA expression in four organs (heart, kidney, liver, skeletal muscle) of hibernating 13-lined ground squirrels by RT-PCR. In their report, several miRNAs (e.g. miR-24, miR-122a, miR-1 and miR-21) were described to be differentially regulated between euthermic (non-hibernating) and hibernating animals, but the pattern differed from one organ to another. Their results did not overlap with ours except for miR-122a, which was found only in the skeletal muscle and was underexpressed during hibernation [21]. Liu et al. was the first to systematically examine the miRNA profiles in the liver of an extreme mammalian hibernator, the arctic ground squirrel by means of Illumina sequencing technology [22]. They constructed small RNA libraries of liver samples from three different physiological stages, early arousal (EA), late torpor (LT) and post reproduction (PR) as a non-hibernating stage, sequenced them and identified differentially expressed miRNAs [22]. The data were compared with the results from two other miRNA profiling methods: Agilent miRNA microarray and real-time PCR. They found that miR-320, miR-378, miR-211, miR-200a,b and miR-184 were significantly down-regulated during both stages of hibernation compared with non-hibernating animals, whereas miR-486, miR-451, miR-144 and miR-142 were significantly overexpressed in late torpor phase [22]. These results had only a limited overlap with our findings that was restricted to miR-200a,b, which were among the miRNAs we found to be down-regulated during hibernation. The lack of overlap between the work mentioned above and ours is not surprising, since the samples (organs, species) analyzed were not the same. In addition, the probes and methods used were different. Indeed, the authors pointed out that different methods gave very limited overlap even though the same samples were used for analyses, but the consistency was very high for those miRNAs that did overlap [22].

The roles of miR-200 family and miR-182 family miRNAs, which we found to be down-regulated during hibernation torpor, have been examined in various areas of research, especially cancer, but the studies have generally been correlational. Close relationships of miR-200 family miRNAs with cancer cell invasion or cancer metastasis have been reported repeatedly [23]-[26]. As a non- cancer related function, miR-200c was reported to be upregulated by oxidative stress and to induce endothelial cell apoptosis and senescence [27]. Members of the miR-182 family miRNAs, were also mainly described in cancer-related studies. These miRNAs were often reported to act like oncogenes: overexpressed in many cancer cells and promote cancer progression, invasion and/or metastasis [28]–[32], but some investigators reported these miRNAs to act as tumor suppressors [33], [34]. It is not clear whether these apparently opposite functions are tumor type-dependent or are examples of the need for more clarity and precision in our understanding of how these miRNAs function within the biological network. Some functions of miR-182 family miRNAs that are not directly related to cancer have also been reported. Examples include a role in DNA repair [35], differential regulation by different light levels in the mouse retina [36], and involvement in ischemic preconditioning [37]. Roh's group [37] reported that miR-200 family and miR-182 family miRNAs increased at 3 hrs after an ischemic preconditioning (15 min transient focal cerebral ischemia) in mice and that overexpressing these miRNAs made mouse neuroblast cells (Neuro-2a) more resistant to OGD (16 h). . The clear increase in expression of miR-200 and miR-182 family miRNAs at the 3 hour timepoint may have reflected the sublethal ischemic stress that induces ischemic tolerance, but at the times that OGD tolerance was tested, these miRNA levels may well have been depressed. In any case, it is quite interesting that two independent labs using different screening techniques found the identical sets of miRNAs to be involved in tolerance to ischemia. The Vemuganti group, however, reported a completely different set of miRNAs to be involved in ischemic pre-conditioning (an endogenous neuroprotective mechanism) in rats [38] possibly adding even more complicated scenarios. A single microRNA generally regulates many genes and a gene is generally regulated by many different microRNAs, so the exact role of a miRNA in any given cellular process can be difficult to sort out. Further work will be needed to better understand the mechanisms involved, but we describe in this report for the first time that ground squirrel natural tolerance to profoundly reduced brain blood flows is linked to both ubiquitin-like modifiers and microRNAs. Examination of the genomic and proteomic changes induced by these linked regulators may enrich our understanding of ischemic brain tolerance.

## Supporting Information

Table S1 **The list of miRNAs which had an absolute mean difference in mean expression between LH and ACR≥1.25.**
(XLS)

Table S2 **miR target prediction**
(PDF)
