# Supplementary material for: Global Protein Conjugation by Ubiquitin-Like-Modifiers during Ischemic Stress Is Regulated by MicroRNAs and Confers Robust Tolerance to Ischemia
Source: PLoS One. 2012 Oct 18;7(10):e47787. doi: 10.1371/journal.pone.0047787 (PMC3475703; doi:10.1371/journal.pone.0047787)
Supplement: Table S2 — miR target prediction (PDF) [file pone.0047787.s002.pdf]

Supplement Table 2. miRNA Target Predictions

Results Page Start	Results Page Stop	Target Gene	Prediction Tool	Hybridization Temperature
2	3	NEDD8	MicroInspector	NA
4	17	NEDD8	RegRNA	37C
18	98	UBC9	RegRNA	37C
99	149	UFM1	RegRNA	37C
150	150	SUMO1	MicroInspector	NA
151	151	SUMO2	MicroInspector	NA
152	152	SUMO3	MicroInspector	NA
153	153	UBA2	MicroInspector	NA
154	154	UBC9	MicroInspector	NA
155	229	UBC9	RegRNA	32C
230	230	UFM1	MicroInspector	NA
231	277	UFM1	RegRNA	32C

RESULTS

POSITION	SEQUENCE OF TARGET	NAME OF MIRNA	SEQUENCE OF MIRNA	FREE ENERGY	LINK (SEC.STRUCTURE .ps)
102	CAGCCATGGGCTGGGACCTGACGGTGAAGATG	hsa-miR-200a-5p MI	caucuuaccggacagugcugga	-23.35	image
424	CCTGAAGCAGCAAGTGAGCGGGCTGGAGGGTG	hsa-miR-141-5p MIM	caucuuccaguacaguguugga	-23.26	image
142	CAACGAATTCCAGGTGTCCCTGAGCAGCTCCA	hsa-miR-122-5p MIM	uggagugugacaaugguguuug	-20.98	image
102	CAGCCATGGGCTGGGACCTGACGGTGAAGATG	hsa-miR-200b-5p MI	caucuuacugggcagcauugga	-20.87	image

Results in .CSV format (Right click and 'Save as')

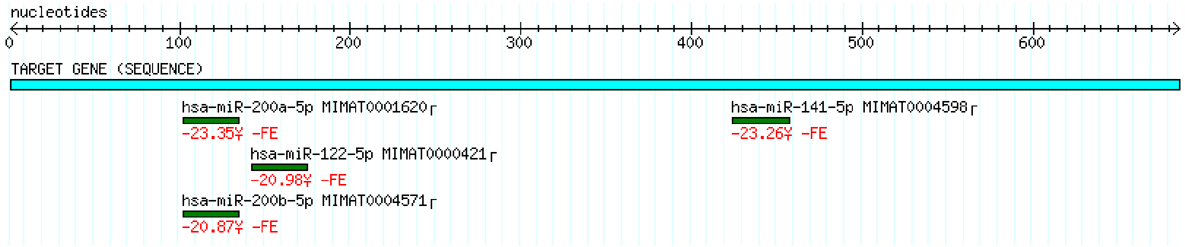

RESULTS

POSITION	SEQUENCE OF TARGET	NAME OF MIRNA	SEQUENCE OF MIRNA	FREE ENERGY	LINK (SEC.STRUCTURE .ps)

Results in .CSV format (Right click and 'Save as')

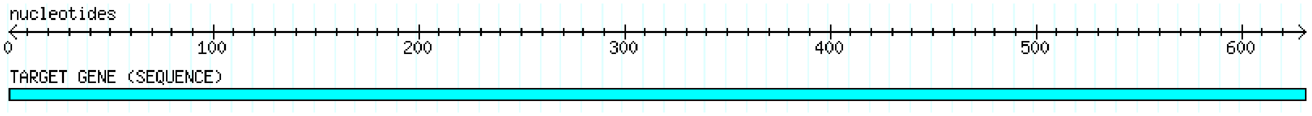

miRNA Target Sites Table View

Target Sequence	><br>AGTAGAAGTGGCCCTTGCAGGCAAGAGTGCTGGAGGGCGGCAGCGGCGACCGGAGCGGTAGGAGCAGCAATTTATCCGTG<br>TGCAGCCCCAAACTGGAAAGAAGATGCTAATTAAAGTGAAGACGCTGACCGGAAAGGAGATTGAGATTGACATTGAACCT<br>ACAGACAAGGTGGAGCGAATCAAGGAGCGTGTGGAGGAGAAAGAGGGAATCCCCCCACAACAGCAGAGGCTCATCTACAG<br>TGGCAAGCAGATGAATGATGAGAAGACAGCAGCTGATTACAAGATTTTAGGTGGTTCAGTCCTTCACCTGGTGTTGGCTC<br>TGAGAGGAGGAGGTGGTCTTAGGCAGTGATGGACCCTCCATTTTACCTCTTTACCCTGTCGCTCATAATGAGGCATCATA<br>TATCCTCTCACTCTCTGGGACACCATAGCCACTGCCCCCTCCCCTGGATGCCCAGTAATGTATGTCTACTGGTGGGAGAC<br>TGTGAGGATCCCAGGATTCAGTATTCCTGGCCCAGAGGGCCCTTGCTGGCTACTGGGTGTTAGTTTGCAGTCCTGTGTGC<br>TTCCCTCTCTTATGACTGTGTCCCTGGTTGTCAATAAAATATTTCCTGGCCTCCTGGAATCTTTC

<table>
<tr><th>No.</th><th>miRNA ID</th><th>Location</th><th>Len</th><th>Hybridization</th><th>Minimum Free Energy</th><th>Score</th><th>Profile</th></tr>
<tr><td>1</td><td>hsa-let-7e</td><td>345~370</td><td>26</td><td>miRNA: 3' uugaUAUGUUGGAG----GAUGGAGu 5'
              ||: | |||| :||||||
Target:5' agtgATGGACCCTCCATTTTACCTCt 3'</td><td>-15.70</td><td>140.00</td><td></td></tr>
<tr><td>2</td><td>hsa-miR-103-2*</td><td>78~105</td><td>28</td><td>miRNA: 3' guucCGUCG-----UGACAUUUCUUCga 5'
              ||||| |||| |||||||
Target:5' gtgtGCAGCCCCAAACTGGAAAGAAGat 3'</td><td>-16.60</td><td>151.00</td><td></td></tr>
<tr><td>3</td><td>hsa-miR-10b*</td><td>600~624</td><td>25</td><td>miRNA: 3' uaAGGGGAUCUUAG---CUUAGAca 5'
            |::|||:| || ||||||
Target:5' taTTTCCTGGCCTCCTGGAATCTtt 3'</td><td>-13.07</td><td>140.00</td><td></td></tr>
<tr><td rowspan="2">4</td><td rowspan="2">hsa-miR-1183</td><td>212~242</td><td>31</td><td>miRNA: 3' acGGGUGAGAGUGGU-----AGUGGAUGUCAc 5'
            ||||| || || |||:|||||||
Target:5' ccCCCAC-AACAGCAGAGGCTCATCTACAGTg 3'</td><td>-29.60</td><td>168.00</td><td rowspan="2"></td></tr>
<tr><td>138~166</td><td>29</td><td>miRNA: 3' acgggugagAGUGGUAG--UGGAUGUCac 5'
                   |:: |||: ||||||||
Target:5' agattgagaTTGACATTGAACCTACAGac 3'</td><td>-13.50</td><td>140.00</td></tr>
<tr><td rowspan="2">5</td><td rowspan="2">hsa-miR-1185</td><td>382~408</td><td>27</td><td>miRNA: 3' uuGUAUGUUUCC------CAUAGGAGa 5'
            |||| :||| |||||||
Target:5' ctCATAATGAGGCATCATATATCCTCt 3'</td><td>-12.70</td><td>149.00</td><td rowspan="2"></td></tr>
<tr><td>382~408</td><td>27</td><td>miRNA: 3' uuGUAUGUUUCC------CAUAGGAGa 5'
            |||| :||| |||||||
Target:5' ctCATAATGAGGCATCATATATCCTCt 3'</td><td>-12.70</td><td>149.00</td></tr>
<tr><td>6</td><td>hsa-miR-1205</td><td>2~21</td><td>20</td><td>miRNA: 3' gagUUUCGUUUGGACGUCu 5'
             :|||::: ||:|||||
Target:5' gtaGAAGTGGCCCTTGCAGg 3'</td><td>-16.00</td><td>145.00</td><td></td></tr>
<tr><td></td><td>hsa-</td><td></td><td></td><td></td><td></td><td></td><td></td></tr>
</table>

<table>
<tr><td>7</td><td>[illegible]miR-1224-3p</td><td>317~338</td><td>22</td><td>miRNA: 3' gacucCUCU-CUCCUCCACCcc 5'
     |||| ||||||||||
Target:5' gctctGAGAGGAGGAGGTGGtc 3'</td><td>-27.90</td><td>152.00</td><td>?</td></tr>
<tr><td>8</td><td>hsa-miR-1236</td><td>170~200</td><td>31</td><td>miRNA: 3' gaCCUCUCU--GUUCC-------CCUUCUCc 5'
  |||| || ||||| |||:|||
Target:5' gtGGAGCGAATCAAGGAGCGTGTGGAGGAGa 3'</td><td>-20.91</td><td>146.00</td><td>?</td></tr>
<tr><td>9</td><td>hsa-miR-1249</td><td>17~40</td><td>24</td><td>miRNA: 3' acUUC-UUC-CCCCCCUUCCCGCa 5'
  |:| ||| | | |:|||||
Target:5' gcAGGCAAGAGTGCTGGAGGGCGg 3'</td><td>-15.90</td><td>140.00</td><td>?</td></tr>
<tr><td>10</td><td>hsa-miR-1254</td><td>488~512</td><td>25</td><td>miRNA: 3' ugacGUCCGAGGUCG-AAGGUCCGa 5'
    |||| |:|||: |||| |||
Target:5' atccCAGGATTCAGTATTCCTGGCc 3'</td><td>-21.90</td><td>144.00</td><td>?</td></tr>
<tr><td>11</td><td>hsa-miR-125a-3p</td><td>292~311</td><td>20</td><td>miRNA: 3' ccgAGGGUUCUUGGAGUGGACa 5'
   |:| || |:|||||||
Target:5' tggTTC--AGTCCTTCACCTGg 3'</td><td>-19.00</td><td>151.00</td><td>?</td></tr>
<tr><td>12</td><td>hsa-miR-1260</td><td>460~478</td><td>19</td><td>miRNA: 3' accACCG-UCUCCACCCUa 5'
   || | | |||||||
Target:5' gtaTGTCTACTGGTGGGAg 3'</td><td>-11.50</td><td>143.00</td><td>?</td></tr>
<tr><td>13</td><td>hsa-miR-1260b</td><td>459~478</td><td>20</td><td>miRNA: 3' uaccACCG-UCACCACCCUa 5'
    || | | ||||||||
Target:5' tgtaTGTCTACTGGTGGGAg 3'</td><td>-12.70</td><td>151.00</td><td>?</td></tr>
<tr><td>14</td><td>hsa-miR-1264</td><td>265~288</td><td>24</td><td>miRNA: 3' uuGUCCACGAGU-UUAUUCUGAAc 5'
  ||| ||| | | ||||:||
Target:5' gaCAGCAGCTGATTACAAGATTTt 3'</td><td>-9.20</td><td>141.00</td><td>?</td></tr>
<tr><td>15</td><td>hsa-miR-1266</td><td>304~325</td><td>22</td><td>miRNA: 3' ucgGGACAAGAUGUCGGGACUCc 5'
   |||| | | ::||:|||||
Target:5' tcaCCTGGTGT-TGGCTCTGAGa 3'</td><td>-18.10</td><td>147.00</td><td>?</td></tr>
<tr><td>16</td><td>hsa-miR-1269</td><td>284~304</td><td>21</td><td>miRNA: 3' ggucAUCGUGCCGAGUCAGGuc 5'
    ||| ::||:|||||||
Target:5' atttTAG-GTGGTTCAGTCCtt 3'</td><td>-19.00</td><td>145.00</td><td>?</td></tr>
<tr><td>17</td><td>hsa-miR-1281</td><td>320~336</td><td>17</td><td>miRNA: 3' ccCUCUCCUCCUCCGCu 5'
  ||||||||||||:|
Target:5' ctGAGAGGAGGAGGTGg 3'</td><td>-29.20</td><td>159.00</td><td>?</td></tr>
<tr><td></td><td></td><td>[illegible]</td><td>[illegible]</td><td>miRNA: 3' uccaGAGUGAAACAACGGGUCu 5'</td><td>[illegible]</td><td>[illegible]</td><td></td></tr>
</table>

18	hsa-miR-1285	435~456	22	||| | | | ||||||| Target:5' ccccCTCCCCTGGATGCCCAGt 3'	-14.80	158.00	
		435~456	22	miRNA: 3' uccaGAGUGAAACAACGGGUCu 5'<br>||| | | | ||||||| Target:5' ccccCTCCCCTGGATGCCCAGt 3'	-14.80	158.00	
19	hsa-miR-1286	534~557	24	miRNA: 3' ucCCG-AGUAGAAC--CAGGACGu 5'<br>||: |:| :||| ||||||:<br>Target:5' tgGGTGTTAGTTTGCAGTCCTGTg 3'	-15.70	141.00	
20	hsa-miR-1288	530~554	25	miRNA: 3' agaGGUC--UAGUC--CCGUCAGGu 5'<br>|::| :|:|| ||||||| Target:5' ctaCTGGGTGTTAGTTTGCAGTCCt 3'	-16.00	146.00	
21	hsa-miR-1298	230~259	30	miRNA: 3' auGUAG-----ACC---UGUCGGCUUACUu 5'<br>|||| ||| :||| :|||||| Target:5' ctCATCTACAGTGGCAAGCAGATGAATGAt 3'	-14.90	144.00	
22	hsa-miR-1302	566~586	21	miRNA: 3' aaaucGUAUUCAUACAGGGuu 5'<br>:||:| |:|||||| Target:5' tctctTATGACTGTGTCCCtg 3'	-13.20	140.00	
		566~586	21	miRNA: 3' aaaucGUAUUCAUACAGGGuu 5'<br>:||:| |:|||||| Target:5' tctctTATGACTGTGTCCCtg 3'	-13.20	140.00	
		566~586	21	miRNA: 3' aaaucGUAUUCAUACAGGGuu 5'<br>:||:| |:|||||| Target:5' tctctTATGACTGTGTCCCtg 3'	-13.20	140.00	
		566~586	21	miRNA: 3' aaaucGUAUUCAUACAGGGuu 5'<br>:||:| |:|||||| Target:5' tctctTATGACTGTGTCCCtg 3'	-13.20	140.00	
		566~586	21	miRNA: 3' aaaucGUAUUCAUACAGGGuu 5'<br>:||:| |:|||||| Target:5' tctctTATGACTGTGTCCCtg 3'	-13.20	140.00	
		566~586	21	miRNA: 3' aaaucGUAUUCAUACAGGGuu 5'<br>:||:| |:|||||| Target:5' tctctTATGACTGTGTCCCtg 3'	-13.20	140.00	
		566~586	21	miRNA: 3' aaaucGUAUUCAUACAGGGuu 5'<br>[illegible]	-13.20	140.00	

<table>
<tr><td></td><td></td><td></td><td></td><td>:||:| |:||||||
Target:5' tctctTATGACTGTGTCCCtg 3'</td><td></td><td></td><td></td></tr>
<tr><td></td><td></td><td>566~586</td><td>21</td><td>miRNA: 3' aaaucGUAUUCAUACAGGGuu 5'
:||:| |:||||||
Target:5' tctctTATGACTGTGTCCCtg 3'</td><td>-13.20</td><td>140.00</td><td></td></tr>
<tr><td></td><td></td><td>566~586</td><td>21</td><td>miRNA: 3' aaaucGUAUUCAUACAGGGuu 5'
:||:| |:||||||
Target:5' tctctTATGACTGTGTCCCtg 3'</td><td>-13.20</td><td>140.00</td><td></td></tr>
<tr><td></td><td></td><td>566~586</td><td>21</td><td>miRNA: 3' aaaucGUAUUCAUACAGGGuu 5'
:||:| |:||||||
Target:5' tctctTATGACTGTGTCCCtg 3'</td><td>-13.20</td><td>140.00</td><td></td></tr>
<tr><td></td><td></td><td>566~586</td><td>21</td><td>miRNA: 3' aaaucGUAUUCAUACAGGGuu 5'
:||:| |:||||||
Target:5' tctctTATGACTGTGTCCCtg 3'</td><td>-13.20</td><td>140.00</td><td></td></tr>
<tr><td>23</td><td>hsa-miR-130b*</td><td>186~206</td><td>21</td><td>miRNA: 3' cauCACGUUGUCCCUUUCUCa 5'
|||::: :| |||||||
Target:5' agcGTGTGGAGGAGAAAGAGg 3'</td><td>-13.70</td><td>158.00</td><td></td></tr>
<tr><td>24</td><td>hsa-miR-132</td><td>463~485</td><td>23</td><td>miRNA: 3' gcuGGU-ACCGACAUCUGACAau 5'
|:| |||: | |||||||
Target:5' tgtCTACTGGTGGGAGACTGTga 3'</td><td>-14.70</td><td>143.00</td><td></td></tr>
<tr><td>25</td><td>hsa-miR-1322</td><td>381~400</td><td>20</td><td>miRNA: 3' gucGUAGU-CGUCGUAGUAg 5'
||| | | :|||||||
Target:5' gctCATAATGAGGCATCATa 3'</td><td>-11.30</td><td>152.00</td><td></td></tr>
<tr><td>26</td><td>hsa-miR-145</td><td>70~98</td><td>29</td><td>miRNA: 3' ucccUAAGGAC-CCU-----UUUGACCUg 5'
|| | || | | ||||||||
Target:5' atttATCCGTGTGCAGCCCCAAACTGGAa 3'</td><td>-10.20</td><td>147.00</td><td></td></tr>
<tr><td>27</td><td>hsa-miR-149*</td><td>423~444</td><td>22</td><td>miRNA: 3' cgUGUCGGGGGC-AGGGAGGGa 5'
|:|||| |:| |||||||
Target:5' ccATAGCCACTGCCCCCTCCCc 3'</td><td>-28.50</td><td>163.00</td><td></td></tr>
<tr><td></td><td></td><td>545~570</td><td>26</td><td>miRNA: 3' cgUGUCGGGG-----GCAGGGAGGGa 5'
:|||:||: | ||||||:|
Target:5' ttGCAGTCCTGTGTGCTTCCCTCTCt 3'</td><td>-25.40</td><td>143.00</td><td></td></tr>
<tr><td></td><td>hsa-</td><td>235~259</td><td>25</td><td>miRNA: 3' ugaGUGGCUG-UCG-CAACUUACaa 5'
|| :|:| ||| | ||||||
Target:5' ctaCAGTGGCAAGCAGATGAATGat 3'</td><td>-15.80</td><td>140.00</td><td></td></tr>
</table>

<table>
<tr><td>28</td><td>miR-181a</td><td></td><td></td><td></td><td></td><td></td></tr>
<tr><td></td><td></td><td>235~259</td><td>25</td><td>miRNA: 3' ugaGUGGCUG-UCG-CAACUUACaa 5'
|| :|:| ||| | ||||||
Target:5' ctaCAGTGGCAAGCAGATGAATGat 3'</td><td>-15.80</td><td>140.00</td></tr>
<tr><td>29</td><td>hsa-miR-181b</td><td>235~259</td><td>25</td><td>miRNA: 3' uggGUGGCUG-UCGU-UACUUACaa 5'
|| :|:| |||| |||||||
Target:5' ctaCAGTGGCAAGCAGATGAATGat 3'</td><td>-17.80</td><td>148.00</td></tr>
<tr><td></td><td></td><td>235~259</td><td>25</td><td>miRNA: 3' uggGUGGCUG-UCGU-UACUUACaa 5'
|| :|:| |||| |||||||
Target:5' ctaCAGTGGCAAGCAGATGAATGat 3'</td><td>-17.80</td><td>148.00</td></tr>
<tr><td>30</td><td>hsa-miR-181d</td><td>235~259</td><td>25</td><td>miRNA: 3' uggGUGGCUGUU-GU-UACUUACaa 5'
|| :|:||| || |||||||
Target:5' ctaCAGTGGCAAGCAGATGAATGat 3'</td><td>-16.00</td><td>148.00</td></tr>
<tr><td>31</td><td>hsa-miR-182</td><td>569~595</td><td>27</td><td>miRNA: 3' ucACACUCA-AGAUGG--UAACGGUUu 5'
| ||| | | | || :|||:|||
Target:5' ctTATGACTGTGTCCCTGGTTGTCAAt 3'</td><td>-9.50</td><td>140.00</td></tr>
<tr><td>32</td><td>hsa-miR-185*</td><td>69~90</td><td>22</td><td>miRNA: 3' cuggucuccuuucgGUCGGGGa 5'
|||||||
Target:5' aatttatccgtgtgCAGCCCCa 3'</td><td>-13.30</td><td>140.00</td></tr>
<tr><td>33</td><td>hsa-miR-18a*</td><td>18~41</td><td>24</td><td>miRNA: 3' ggUCUUCCUCGUGA-AUCCCGUCa 5'
:| | |||::|| |||||:|
Target:5' caGGCAAGAGTGCTGGAGGGCGGc 3'</td><td>-20.70</td><td>145.00</td></tr>
<tr><td>34</td><td>hsa-miR-197</td><td>319~340</td><td>22</td><td>miRNA: 3' cgACCCACCUCUUCCACCAcuu 5'
|| | ||||:|||||||
Target:5' tcTGAGAGGAGGAGGTGGTctt 3'</td><td>-24.72</td><td>140.00</td></tr>
<tr><td>35</td><td>hsa-miR-1976</td><td>43~65</td><td>23</td><td>miRNA: 3' ugUCGUU--CCUC-CCGUCCUCc 5'
:||:| |||| ||:||||
Target:5' gcGGCGACCGGAGCGGTAGGAGc 3'</td><td>-26.00</td><td>148.00</td></tr>
<tr><td>36</td><td>hsa-miR-199a-3p</td><td>515~536</td><td>22</td><td>miRNA: 3' auugguuacACGUCUGAUGACa 5'
||| |:||||||
Target:5' gagggccctTGCTGGCTACTGg 3'</td><td>-16.10</td><td>141.00</td></tr>
<tr><td></td><td></td><td>515~536</td><td>22</td><td>miRNA: 3' auugguuacACGUCUGAUGACa 5'
||| |:||||||
Target:5' gagggccctTGCTGGCTACTGg 3'</td><td>-16.10</td><td>141.00</td></tr>
</table>

<table>
<tr><td>37</td><td>hsa-miR-199b-3p</td><td>515~536</td><td>22</td><td>miRNA: 3' auugguuacACGUCUGAUGACa 5'
                   ||| |:||||||
Target:5' gagggccctTGCTGGCTACTGg 3'</td><td>-16.10</td><td>141.00</td><td>?</td></tr>
<tr><td>38</td><td>hsa-miR-200b</td><td>482~506</td><td>25</td><td>miRNA: 3' aguaguaAUGGUCC---GUCAUAAu 5'
                 | ||||| |||||||
Target:5' gtgaggaTCCCAGGATTCAGTATTc 3'</td><td>-12.70</td><td>155.00</td><td>?</td></tr>
<tr><td>39</td><td>hsa-miR-200c</td><td>481~506</td><td>26</td><td>miRNA: 3' agguaguaAUGGGCC---GUCAUAAu 5'
                  | || || |||||||
Target:5' tgtgaggaTCCCAGGATTCAGTATTc 3'</td><td>-11.40</td><td>147.00</td><td>?</td></tr>
<tr><td>40</td><td>hsa-miR-204</td><td>189~209</td><td>21</td><td>miRNA: 3' ucCGUAUCCUACUGUUUCCCUu 5'
            |:: |||| || |:|||||
Target:5' gtGTGGAGGA-GAAAGAGGGAa 3'</td><td>-16.90</td><td>147.00</td><td>?</td></tr>
<tr><td>41</td><td>hsa-miR-2053</td><td>130~153</td><td>24</td><td>miRNA: 3' caUUUAUCUCCAAAU-UAAUUGUg 5'
            :|| :||| || | |||:|||
Target:5' cgGAAAGGAGATTGAGATTGACAt 3'</td><td>-7.80</td><td>149.00</td><td>?</td></tr>
<tr><td>42</td><td>hsa-miR-21*</td><td>296~316</td><td>21</td><td>miRNA: 3' ugUCGGGUAGCUGACCACAAc 5'
            ||:|| || ||||||||
Target:5' tcAGTCCTTCACCTGGTGTTg 3'</td><td>-25.50</td><td>167.00</td><td>?</td></tr>
<tr><td>43</td><td>hsa-miR-211</td><td>189~209</td><td>21</td><td>miRNA: 3' ucCGCUUCCUACUGUUUCCCUu 5'
            |:|:|||| || |:|||||
Target:5' gtGTGGAGGA-GAAAGAGGGAa 3'</td><td>-23.00</td><td>155.00</td><td>?</td></tr>
<tr><td>44</td><td>hsa-miR-2114*</td><td>213~233</td><td>21</td><td>miRNA: 3' uucagggaacgaaCUCCGAGc 5'
                       |||||||
Target:5' ccccacaacagcaGAGGCTCa 3'</td><td>-12.70</td><td>140.00</td><td>?</td></tr>
<tr><td>45</td><td>hsa-miR-212</td><td>465~485</td><td>21</td><td>miRNA: 3' ccggcACUGACCUCUGACAau 5'
               ||:: |||||||||
Target:5' tctacTGGTGGGAGACTGTga 3'</td><td>-17.40</td><td>144.00</td><td>?</td></tr>
<tr><td>46</td><td>hsa-miR-216a</td><td>127~149</td><td>23</td><td>miRNA: 3' agugucaacggUC-GACUCUAAu 5'
                     || :|||||||
Target:5' gaccggaaaggAGATTGAGATTg 3'</td><td>-12.72</td><td>143.00</td><td>?</td></tr>
<tr><td>47</td><td>hsa-miR-222*</td><td>516~537</td><td>22</td><td>miRNA: 3' ucCUAGAUGUGACCGAUGACUc 5'
            |: | ::||||||||||:
Target:5' agGGCCCTTGCTGGCTACTGGg 3'</td><td>-25.60</td><td>148.00</td><td>?</td></tr>
</table>

<table>
<tr><th></th><th></th><th></th><th></th><th></th><th></th><th></th></tr>
<tr><td>48</td><td>hsa-miR-2276</td><td>1~21</td><td>21</td><td>miRNA: 3' ggagcggagACUGUGAACGUCu 5'
||:| |||||||
Target:5' -agtagaagTGGCCCTTGCAGg 3'</td><td>-14.80</td><td>153.00</td></tr>
<tr><td>49</td><td>hsa-miR-27a</td><td>466~486</td><td>21</td><td>miRNA: 3' cgccuugAAUCGGUGACACUu 5'
| :| |||||||
Target:5' ctactggTGGGAGACTGTGAg 3'</td><td>-13.90</td><td>142.00</td></tr>
<tr><td>50</td><td>hsa-miR-27b</td><td>466~486</td><td>21</td><td>miRNA: 3' cgucuugAAUCGGUGACACUu 5'
| :| |||||||
Target:5' ctactggTGGGAGACTGTGAg 3'</td><td>-12.30</td><td>142.00</td></tr>
<tr><td>51</td><td>hsa-miR-302f</td><td>55~73</td><td>19</td><td>miRNA: 3' uuuguaCCU--UCGUUAAu 5'
||| |||||||
Target:5' gcggtaGGAGCAGCAATTt 3'</td><td>-8.30</td><td>145.00</td></tr>
<tr><td>52</td><td>hsa-miR-3065-3p</td><td>294~317</td><td>24</td><td>miRNA: 3' gaGGUU-GUUAUAGGACCACGACu 5'
:||: | | |||||||:||
Target:5' gtTCAGTCCTTCACCTGGTGTTGg 3'</td><td>-20.40</td><td>141.00</td></tr>
<tr><td>53</td><td>hsa-miR-30b*</td><td>389~411</td><td>23</td><td>miRNA: 3' cuUCAUUUGUAGGU-GGAGGGUc 5'
|| | ||| :| ||||:||
Target:5' tgAGGCATCATATATCCTCTCAc 3'</td><td>-14.20</td><td>140.00</td></tr>
<tr><td>54</td><td>hsa-miR-3117</td><td>534~556</td><td>23</td><td>miRNA: 3' gaCCGUGAU-AUAC-UCAGGAUa 5'
||:::|| | || ||||||:
Target:5' tgGGTGTTAGTTTGCAGTCCTGt 3'</td><td>-17.70</td><td>143.00</td></tr>
<tr><td rowspan="2">55</td><td rowspan="2">hsa-miR-3126-5p</td><td>567~587</td><td>21</td><td>miRNA: 3' acGAAGACCGUAGACAGGGAgu 5'
||| || | | |||||||
Target:5' ctCTTATGAC-TGTGTCCCTgg 3'</td><td>-14.10</td><td>143.00</td></tr>
<tr><td>283~306</td><td>24</td><td>miRNA: 3' acGAAGACCGU--AGACAGGGAGu 5'
:||: ||:: || ||||:||
Target:5' gaTTTTAGGTGGTTCAGTCCTTCa 3'</td><td>-15.70</td><td>142.00</td></tr>
<tr><td rowspan="2">56</td><td rowspan="2">hsa-miR-3130-5p</td><td>520~539</td><td>20</td><td>miRNA: 3' ccGACGUGGCCUCUGACCCAu 5'
|| ::|:|| |||||||
Target:5' ccCT-TGCTGGCTACTGGGTg 3'</td><td>-18.40</td><td>154.00</td></tr>
<tr><td>520~539</td><td>20</td><td>miRNA: 3' ccGACGUGGCCUCUGACCCAu 5'
|| ::|:|| |||||||
Target:5' ccCT-TGCTGGCTACTGGGTg 3'</td><td>-18.40</td><td>154.00</td></tr>
<tr><td></td><td>hsa-</td><td></td><td></td><td>miRNA: 3' [illegible] 5'</td><td></td><td></td></tr>
</table>

<table>
<tr><td>57</td><td>miR-3132</td><td>354~377</td><td>24</td><td>miRNA: 3' agGAGACUCGAGGAAGAGAUGGGu 5'
||| :| | |||:|||||
Target:5' ccCTCCATTTTACCTCTTTACCCt 3'</td><td>-18.72</td><td>142.00</td><td></td></tr>
<tr><td>58</td><td>hsa-miR-3136</td><td>280~303</td><td>24</td><td>miRNA: 3' uuaCUGGGAUGGA-UAAGUCAGuc 5'
||:::|| | :|||||||
Target:5' caaGATTTTAGGTGGTTCAGTCct 3'</td><td>-12.30</td><td>140.00</td><td></td></tr>
<tr><td>59</td><td>hsa-miR-3162</td><td>351~374</td><td>24</td><td>miRNA: 3' gagGGGUGGGAAGAU-GAGGGAUu 5'
||| || ||:|| |||::||
Target:5' ggaCCCTCCATTTTACCTCTTTAc 3'</td><td>-16.62</td><td>140.00</td><td></td></tr>
<tr><td>60</td><td>hsa-miR-3163</td><td>343~367</td><td>25</td><td>miRNA: 3' cagaAUGAC--GGGA-GUAAAAUau 5'
|: || |||| |||||||
Target:5' gcagTGATGGACCCTCCATTTTAcc 3'</td><td>-10.30</td><td>140.00</td><td></td></tr>
<tr><td rowspan="2">61</td><td rowspan="2">hsa-miR-3169</td><td>533~555</td><td>23</td><td>miRNA: 3' gauaCACGGUU-CGUGUCAGGAu 5'
|||::|: ::|||||||
Target:5' ctggGTGTTAGTTTGCAGTCCTg 3'</td><td>-19.80</td><td>154.00</td><td rowspan="2"></td></tr>
<tr><td>287~304</td><td>18</td><td>miRNA: 3' gaUACACGGUUCGUGUCAGGAu 5'
| ||| |: |||||||
Target:5' ttAGGTG----GTTCAGTCCTt 3'</td><td>-13.00</td><td>146.00</td></tr>
<tr><td>62</td><td>hsa-miR-3171</td><td>382~405</td><td>24</td><td>miRNA: 3' cuaUAUAUGUCUAAGGUAUGUAGa 5'
||| ||: :||||:|||
Target:5' ctcATAATGAGGCATCATATATCc 3'</td><td>-9.80</td><td>141.00</td><td></td></tr>
<tr><td>63</td><td>hsa-miR-3173</td><td>352~373</td><td>22</td><td>miRNA: 3' accGGACGGAUAAAGGAGGAAa 5'
||| | | || ||||:||
Target:5' gacCCTCCATTTTACCTCTTTa 3'</td><td>-12.00</td><td>147.00</td><td></td></tr>
<tr><td>64</td><td>hsa-miR-3186-3p</td><td>63~83</td><td>21</td><td>miRNA: 3' guuUCGGUAGAGAGGCGCACu 5'
||| ||:| ||||:|||
Target:5' agcAGCAATTTATCCGTGTGc 3'</td><td>-17.40</td><td>154.00</td><td></td></tr>
<tr><td>65</td><td>hsa-miR-3198</td><td>404~426</td><td>23</td><td>miRNA: 3' agAG-GUAAGGGGUCCUGAGGUg 5'
|| || ||:|::|||| |||
Target:5' ccTCTCACTCTCTGGGACACCAt 3'</td><td>-23.30</td><td>140.00</td><td></td></tr>
<tr><td>66</td><td>hsa-miR-323-3p</td><td>443~462</td><td>20</td><td>miRNA: 3' ucuCCAGCUGGCACAUUACAc 5'
|| :| || |||||||
Target:5' cctGGATG-CCCAGTAATGTa 3'</td><td>-9.60</td><td>149.00</td><td></td></tr>
<tr><td></td><td></td><td>451~473</td><td>23</td><td>miRNA: 3' ugugaAUGACCUGUGGAUGAUCc 5'
|| || |:::|||||:|</td><td>-11.30</td><td>146.00</td><td></td></tr>
</table>

<table>
<tr><td rowspan="2">67</td><td rowspan="2">hsa-miR-325</td><td></td><td></td><td>Target:5' cccagTAATGTATGTCTACTGGt 3'</td><td></td><td></td><td rowspan="2"></td></tr>
<tr><td>519~537</td><td>19</td><td>miRNA: 3' uguGAAUGACCUGUGGAUGAUCc 5'
|||:|||| |||||:|
Target:5' gccCTTGCTGG----CTACTGGg 3'</td><td>-21.50</td><td>146.00</td></tr>
<tr><td>68</td><td>hsa-miR-326</td><td>499~518</td><td>20</td><td>miRNA: 3' gaccuccuucCCGGGUCUCc 5'
|||||||||
Target:5' cagtattcctGGCCCAGAGg 3'</td><td>-21.30</td><td>150.00</td><td></td></tr>
<tr><td>69</td><td>hsa-miR-330-5p</td><td>489~518</td><td>30</td><td>miRNA: 3' cgGAUUCUGUGU--------CCGGGUCUCu 5'
| |:||: || |||||||||
Target:5' tcCCAGGATTCAGTATTCCTGGCCCAGAGg 3'</td><td>-24.10</td><td>154.00</td><td></td></tr>
<tr><td>70</td><td>hsa-miR-330-3p</td><td>542~565</td><td>24</td><td>miRNA: 3' agAGACGUCCGG-CACACGAAacg 5'
|:||||| || ||||||||
Target:5' agTTTGCAGTCCTGTGTGCTTccc 3'</td><td>-22.80</td><td>145.00</td><td></td></tr>
<tr><td>71</td><td>hsa-miR-335*</td><td>245~265</td><td>21</td><td>miRNA: 3' ccaGUCCUCGUUAUUACUUUUu 5'
|| || |||:||||:||
Target:5' aagCA-GATGAATGATGAGAAg 3'</td><td>-13.30</td><td>146.00</td><td></td></tr>
<tr><td>72</td><td>hsa-miR-34a</td><td>419~437</td><td>19</td><td>miRNA: 3' uguUGGUCGAUUCUGUGACGGu 5'
|||| | || |||||||
Target:5' gacACCA--T-AGCCACTGCCc 3'</td><td>-15.60</td><td>154.00</td><td></td></tr>
<tr><td>73</td><td>hsa-miR-34c-5p</td><td>416~437</td><td>22</td><td>miRNA: 3' cguuaGUCG-AUUGAUGUGACGGa 5'
|| | ||:| |||||||
Target:5' tgggaCACCATAGC--CACTGCCc 3'</td><td>-12.30</td><td>150.00</td><td></td></tr>
<tr><td>74</td><td>hsa-miR-3654</td><td>283~301</td><td>19</td><td>miRNA: 3' aaGGAGUCGAACAGGUCAg 5'
::|:|| | ||:||||
Target:5' gaTTTTAGGTGGTTCAGTc 3'</td><td>-15.70</td><td>141.00</td><td></td></tr>
<tr><td>75</td><td>hsa-miR-3657</td><td>406~424</td><td>19</td><td>miRNA: 3' uuAGUGGUUAUUACCCUGUGu 5'
||||: | ||||||||
Target:5' tcTCACT-CT-CTGGGACACc 3'</td><td>-14.90</td><td>149.00</td><td></td></tr>
<tr><td>76</td><td>hsa-miR-3669</td><td>485~509</td><td>25</td><td>miRNA: 3' auaUAAGG-CAUAUGU-AUAAGGca 5'
|| || | || || ||||||
Target:5' aggATCCCAGGATTCAGTATTCCtg 3'</td><td>-11.00</td><td>140.00</td><td></td></tr>
<tr><td>77</td><td>hsa-miR-3675-5p</td><td>67~92</td><td>26</td><td>miRNA: 3' cuUUAGAGAUGU---CUUCGGGGUau 5'
|||:| | |: | |||||||
Target:5' gcAATTTATCCGTGTGCAGCCCCAaa 3'</td><td>-12.40</td><td>141.00</td><td></td></tr>
</table>

<table>
<tr><td>78</td><td>hsa-miR-3679-5p</td><td>386~408</td><td>23</td><td>miRNA: 3' aggggaagGGACGGUAUAGGAGu 5'
          | | :||||||||
Target:5' taatgaggCATCATATATCCTCt 3'</td><td>-11.00</td><td>147.00</td><td>?</td></tr>
<tr><td>79</td><td>hsa-miR-3689a-3p</td><td>392~411</td><td>20</td><td>miRNA: 3' ugGUGCUAUAGUGUGGAGGGUc 5'
  ||: |||| |: ||||:||
Target:5' ggCATCATAT-AT-CCTCTCAc 3'</td><td>-12.70</td><td>142.00</td><td>?</td></tr>
<tr><td>80</td><td>hsa-miR-3689b*</td><td>382~411</td><td>30</td><td>miRNA: 3' ugGUGUUA-----UAGUGU---GGAGGGUc 5'
  ||:||| ||||:| ||||:||
Target:5' ctCATAATGAGGCATCATATATCCTCTCAc 3'</td><td>-15.40</td><td>148.00</td><td>?</td></tr>
<tr><td rowspan="2">81</td><td rowspan="2">hsa-miR-3909</td><td>501~521</td><td>21</td><td>miRNA: 3' ucUGACGUCCGGGAUCUCCUGu 5'
  |:| | ||||| |||||:|
Target:5' gtATTCCTGGCCC-AGAGGGCc 3'</td><td>-21.10</td><td>151.00</td><td rowspan="2">?</td></tr>
<tr><td>308~330</td><td>23</td><td>miRNA: 3' ucugACGUCCGGGA-UCUCCUgu 5'
    ||: |||:|| ||||||
Target:5' ctggTGTTGGCTCTGAGAGGAgg 3'</td><td>-19.20</td><td>146.00</td></tr>
<tr><td>82</td><td>hsa-miR-3916</td><td>346~371</td><td>26</td><td>miRNA: 3' gaCU-CUUGGUCGGUAAAGAAGGAGAa 5'
  || |:||| |||||| | |||||
Target:5' gtGATGGACCCTCCATTT-TACCTCTt 3'</td><td>-22.80</td><td>147.00</td><td>?</td></tr>
<tr><td>83</td><td>hsa-miR-3918</td><td>299~323</td><td>25</td><td>miRNA: 3' ucaGAGGUAGAC----GCCGGGACa 5'
   ||:|| ||| :|||:|||
Target:5' gtcCTTCACCTGGTGTTGGCTCTGa 3'</td><td>-19.20</td><td>144.00</td><td>?</td></tr>
<tr><td>84</td><td>hsa-miR-3938</td><td>190~211</td><td>22</td><td>miRNA: 3' ggcccaauagaUGUUCCCUUAa 5'
           | :|||||||
Target:5' tgtggaggagaAAGAGGGAATc 3'</td><td>-11.54</td><td>143.00</td><td>?</td></tr>
<tr><td>85</td><td>hsa-miR-411</td><td>452~471</td><td>20</td><td>miRNA: 3' gcaugcgAUAUGCCAGAUGAu 5'
       |:||: |||||||
Target:5' ccagtaaTGTAT-GTCTACTg 3'</td><td>-13.80</td><td>149.00</td><td>?</td></tr>
<tr><td>86</td><td>hsa-miR-412</td><td>155~176</td><td>22</td><td>miRNA: 3' ugccGAUCACCUGGUCCACUUCa 5'
    ||| ||| |||||:||
Target:5' gaacCTA-CAGACAAGGTGGAGc 3'</td><td>-14.90</td><td>142.00</td><td>?</td></tr>
<tr><td>87</td><td>hsa-miR-4252</td><td>225~245</td><td>21</td><td>miRNA: 3' accaCGACU-GA-GUCACCGg 5'
    ||| | || |||||||
Target:5' agagGCTCATCTACAGTGGCa 3'</td><td>-16.10</td><td>151.00</td><td>?</td></tr>
</table>

<table>
<tr><td>88</td><td>hsa-miR-4260</td><td>69~92</td><td>24</td><td>miRNA: 3' acccugAGGUA-----CGGGGUUc 5'
|||:| |||||||
Target:5' aatttaTCCGTGTGCAGCCCCAAa 3'</td><td>-14.50</td><td>145.00</td><td></td></tr>
<tr><td>89</td><td>hsa-miR-4268</td><td>314~334</td><td>21</td><td>miRNA: 3' guguaGGACUCUCCUCCUCgg 5'
:|||||||||||||
Target:5' ttggcTCTGAGAGGAGGAGgt 3'</td><td>-26.60</td><td>156.00</td><td></td></tr>
<tr><td>90</td><td>hsa-miR-4270</td><td>399~418</td><td>20</td><td>miRNA: 3' cgggAGGGGACUGAGGGACu 5'
|||:|| ||||:|||
Target:5' tataTCCTCTCACTCTCTGg 3'</td><td>-21.60</td><td>152.00</td><td></td></tr>
<tr><td>91</td><td>hsa-miR-4279</td><td>184~201</td><td>18</td><td>miRNA: 3' cuUCG-GC-CCUCCUCUc 5'
||| :| ||||||||
Target:5' ggAGCGTGTGGAGGAGAa 3'</td><td>-18.70</td><td>150.00</td><td></td></tr>
<tr><td>92</td><td>hsa-miR-429</td><td>482~506</td><td>25</td><td>miRNA: 3' ugccaaaAUGGUCU---GUCAUAAu 5'
| ||||: |||||||
Target:5' gtgaggaTCCCAGGATTCAGTATTc 3'</td><td>-11.50</td><td>151.00</td><td></td></tr>
<tr><td>93</td><td>hsa-miR-4290</td><td>18~39</td><td>22</td><td>miRNA: 3' cuCCCUUCUU---UCCUCCCGu 5'
|| ||||: |||||||
Target:5' caGGCAAGAGTGCTGGAGGGCg 3'</td><td>-22.50</td><td>153.00</td><td></td></tr>
<tr><td>94</td><td>hsa-miR-4310</td><td>18~33</td><td>16</td><td>miRNA: 3' ccCUGUACUUACGACg 5'
|:|| ||:|||||
Target:5' caGGCAAGAGTGCTGg 3'</td><td>-14.80</td><td>142.00</td><td></td></tr>
<tr><td>95</td><td>hsa-miR-4324</td><td>328~344</td><td>17</td><td>miRNA: 3' aaUUCCAAUCCCAGAGUCCc 5'
:|||| |||||:|||
Target:5' agGAGGT---GGTCTTAGGc 3'</td><td>-17.40</td><td>143.00</td><td></td></tr>
<tr><td>96</td><td>hsa-miR-4327</td><td>5~23</td><td>19</td><td>miRNA: 3' ggUCAGGGGGUACGUUCGg 5'
||| ||| ||||:||
Target:5' gaAGTGGCCCTTGCAGGCa 3'</td><td>-17.40</td><td>145.00</td><td></td></tr>
<tr><td>97</td><td>hsa-miR-449a</td><td>414~437</td><td>24</td><td>miRNA: 3' uggucgauUGUUAU--GUGACGGu 5'
|| ||| |||||||
Target:5' tctgggacACCATAGCCACTGCCc 3'</td><td>-13.20</td><td>152.00</td><td></td></tr>
<tr><td>98</td><td>hsa-miR-449b</td><td>414~437</td><td>24</td><td>miRNA: 3' cggucgauUGUUAU--GUGACGGa 5'
|| ||| |||||||
Target:5' tctgggacACCATAGCCACTGCCc 3'</td><td>-13.60</td><td>152.00</td><td></td></tr>
</table>

<table>
<tr><td>99</td><td>hsa-miR-449c</td><td>408~438</td><td>31</td><td>miRNA: 3' ugUCGGCGAUC--GU--UAU--GUGACGGau 5'
            | :| ||:| || ||| |||||||
Target:5' tcACTCTCTGGGACACCATAGCCACTGCCcc 3'</td><td>-15.60</td><td>141.00</td><td></td></tr>
<tr><td>100</td><td>hsa-miR-450b-3p</td><td>472~494</td><td>23</td><td>miRNA: 3' auaCCU---ACGUUUUACUAGGGUu 5'
             ||| ||::|: |||||||
Target:5' gtgGGAGACTGTGAG--GATCCCAg 3'</td><td>-15.64</td><td>151.00</td><td></td></tr>
<tr><td>101</td><td>hsa-miR-452*</td><td>234~255</td><td>22</td><td>miRNA: 3' gugaaugaagaaaCGUCUACUc 5'
                       ||||||||
Target:5' tctacagtggcaaGCAGATGAa 3'</td><td>-10.10</td><td>145.00</td><td></td></tr>
<tr><td>102</td><td>hsa-miR-486-3p</td><td>419~439</td><td>21</td><td>miRNA: 3' uaggacaugacucGACGGGGc 5'
                       |||||||
Target:5' gacaccatagccaCTGCCCCc 3'</td><td>-13.20</td><td>140.00</td><td></td></tr>
<tr><td rowspan="2">103</td><td rowspan="2">hsa-miR-505*</td><td>593~615</td><td>23</td><td>miRNA: 3' uguagUUAUGAAGGACC-GAGGg 5'
               ||||:||||||| ||||
Target:5' aataaAATATTTCCTGGCCTCCt 3'</td><td>-23.20</td><td>149.00</td><td rowspan="2"></td></tr>
<tr><td>293~322</td><td>30</td><td>miRNA: 3' uguAGUUAUGAAG--------GACCGAGGg 5'
             |||:| |||| :||||||:
Target:5' ggtTCAGTCCTTCACCTGGTGTTGGCTCTg 3'</td><td>-14.60</td><td>141.00</td></tr>
<tr><td>104</td><td>hsa-miR-505</td><td>298~319</td><td>22</td><td>miRNA: 3' uccuuuGGUCGUUCACAACUGc 5'
                :|| | :||||||:|
Target:5' agtcctTCACCTGGTGTTGGCt 3'</td><td>-11.60</td><td>140.00</td><td></td></tr>
<tr><td rowspan="2">105</td><td rowspan="2">hsa-miR-509-5p</td><td>534~552</td><td>19</td><td>miRNA: 3' acuaACGGUGACAGACGUCAu 5'
              ||::| ||:||||||

Target:5' tgggTGTTA--GTTTGCAGTc 3'</td><td>-13.50</td><td>141.00</td><td rowspan="2"></td></tr>
<tr><td>534~552</td><td>19</td><td>miRNA: 3' acuaACGGUGACAGACGUCAu 5'
              ||::| ||:||||||
Target:5' tgggTGTTA--GTTTGCAGTc 3'</td><td>-13.50</td><td>141.00</td></tr>
<tr><td>106</td><td>hsa-miR-509-3-5p</td><td>532~552</td><td>21</td><td>miRNA: 3' guACUAACGGUGCAGACGUCAu 5'
            ||: ||::| ||:||||||
Target:5' acTGGGTGTTA-GTTTGCAGTc 3'</td><td>-16.20</td><td>151.00</td><td></td></tr>
<tr><td>107</td><td>hsa-miR-532-3p</td><td>459~479</td><td>21</td><td>miRNA: 3' acGUUCGGAACCCACACCCUCc 5'
            :| |:|| | |||||||
Target:5' tgTATGTCTACTG-GTGGGAGa 3'</td><td>-16.80</td><td>147.00</td><td></td></tr>
<tr><td></td><td>hsa-</td><td></td><td></td><td></td><td></td><td></td><td></td></tr>
</table>

<table>
<tr><td>108</td><td>miR-549</td><td>572~593</td><td>22</td><td>miRNA: 3' ucucGA-GUAGGUAUCAACAGu 5'
|| ::||| |:||||||
Target:5' atgaCTGTGTCCCTGGTTGTCa 3'</td><td>-14.90</td><td>145.00</td><td>?</td></tr>
<tr><td>109</td><td>hsa-miR-572</td><td>43~60</td><td>18</td><td>miRNA: 3' acCCGGUGGCGGCUCGCCug 5'
||| |||| ||||||
Target:5' gcGGCGACCG--GAGCGGta 3'</td><td>-22.40</td><td>142.00</td><td>?</td></tr>
<tr><td>110</td><td>hsa-miR-583</td><td>343~373</td><td>31</td><td>miRNA: 3' caUUAC--CCUGG-------AA-GGAGAAAc 5'
|:|| ||||| || |||||||
Target:5' gcAGTGATGGACCCTCCATTTTACCTCTTTa 3'</td><td>-16.10</td><td>153.00</td><td>?</td></tr>
<tr><td>111</td><td>hsa-miR-584</td><td>407~428</td><td>22</td><td>miRNA: 3' gaGUCAGGGUCCGUUUGGUAUu 5'
|| ||:| || | ||||||
Target:5' ctCACTCTCTGGGACACCATAg 3'</td><td>-19.70</td><td>140.00</td><td>?</td></tr>
<tr><td>112</td><td>hsa-miR-593</td><td>213~231</td><td>19</td><td>miRNA: 3' ucuuUGGGGUCGUCUCUGu 5'
|| ||||||||:|
Target:5' ccccACAACAGCAGAGGCt 3'</td><td>-18.60</td><td>143.00</td><td>?</td></tr>
<tr><td rowspan="2">113</td><td rowspan="2">hsa-miR-612</td><td>492~516</td><td>25</td><td>miRNA: 3' uuCCUCGAGUCUUCGGGA-CGGGUCg 5'
||| :|||| | :||| ||||||
Target:5' caGGA-TTCAGTATTCCTGGCCCAGa 3'</td><td>-26.80</td><td>146.00</td><td rowspan="2">?</td></tr>
<tr><td>428~456</td><td>29</td><td>miRNA: 3' uuccucgagucuucGGG----ACGGGUCg 5'
||| |||||||
Target:5' gccactgccccctcCCCTGGATGCCCAGt 3'</td><td>-17.50</td><td>141.00</td></tr>
<tr><td>114</td><td>hsa-miR-621</td><td>511~531</td><td>21</td><td>miRNA: 3' uccaUUCGCGACAACGAUCGg 5'
:|| || |||||:||
Target:5' cccaGAGGGCCCTTGCTGGCt 3'</td><td>-15.00</td><td>141.00</td><td>?</td></tr>
<tr><td>115</td><td>hsa-miR-626</td><td>253~271</td><td>19</td><td>miRNA: 3' uucuguaaaagUCUGUCGa 5'
|||||||
Target:5' gaatgatgagaAGACAGCa 3'</td><td>-8.80</td><td>140.00</td><td>?</td></tr>
<tr><td>116</td><td>hsa-miR-639</td><td>24~50</td><td>27</td><td>miRNA: 3' ugUCGCGA----GCGUUGGCGUCGCUa 5'
||:||| ||::| ||:||||
Target:5' agAGTGCTGGAGGGCGGCAGCGGCGAc 3'</td><td>-23.50</td><td>147.00</td><td>?</td></tr>
<tr><td>117</td><td>hsa-miR-642a</td><td>181~209</td><td>29</td><td>miRNA: 3' guUCUGUGUAAACC-------UCUCCCUg 5'
||: :|:| ||| |||||||
Target:5' caAGGAGCGTGTGGAGGAGAAAGAGGGAa 3'</td><td>-20.11</td><td>152.00</td><td>?</td></tr>
<tr><td>118</td><td>hsa-miR-[illegible]</td><td>559~583</td><td>25</td><td>miRNA: 3' cccAGGGAGAG---GUUUACACAGa 5'
|||||||| ::| ||||||</td><td>-20.30</td><td>143.00</td><td>?</td></tr>
</table>

<table>
<tr><td></td><td>[illegible]</td><td></td><td></td><td>Target:5' gctTCCCTCTCTTATGACTGTGTCc 3'</td><td></td><td></td><td></td></tr>
<tr><td rowspan="2">119</td><td rowspan="2">hsa-miR-665</td><td>600~618</td><td>19</td><td>miRNA: 3' uccccGGAGUCGGAGGACCa 5'
               ||| :|||||||||
Target:5' tatttCCT-GGCCTCCTGGa 3'</td><td>-22.80</td><td>158.00</td><td rowspan="2">?</td></tr>
<tr><td>491~511</td><td>21</td><td>miRNA: 3' uccCCGGAGUCG-GAGGACCa 5'
             || :||||: :||||||
Target:5' ccaGGATTCAGTATTCCTGGc 3'</td><td>-19.40</td><td>145.00</td></tr>
<tr><td>120</td><td>hsa-miR-766</td><td>14~36</td><td>23</td><td>miRNA: 3' cgAC-UCCGACACCCCGACCUCa 5'
            || |||| | |||||||
Target:5' ctTGCAGGCAAGAGTGCTGGAGg 3'</td><td>-16.90</td><td>152.00</td><td>?</td></tr>
<tr><td>121</td><td>hsa-miR-769-3p</td><td>472~494</td><td>23</td><td>miRNA: 3' uuggUUCUGGGGCCUCUAGGGUc 5'
              :||||: :| :|||||||
Target:5' gtggGAGACTGTGAGGATCCCAg 3'</td><td>-26.00</td><td>163.00</td><td>?</td></tr>
<tr><td rowspan="2">122</td><td rowspan="2">hsa-miR-770-5p</td><td>9~34</td><td>26</td><td>miRNA: 3' acCGGGACUGU--GCAC-CAUGACCu 5'
            ||||| :|| | | ||:||||
Target:5' tgGCCCTTGCAGGCAAGAGTGCTGGa 3'</td><td>-25.80</td><td>143.00</td><td rowspan="2">?</td></tr>
<tr><td>297~318</td><td>22</td><td>miRNA: 3' acCGGGACUGUGCACCAUGACCu 5'
            |:||| ||| ||||::|||
Target:5' caGTCCT-TCACCTGGTGTTGGc 3'</td><td>-22.30</td><td>140.00</td></tr>
<tr><td>123</td><td>hsa-miR-93</td><td>52~74</td><td>23</td><td>miRNA: 3' gaUGGACGUGCUUGUCGUGAAAc 5'
            | | |:| ||:||||| |||
Target:5' ggAGCGGTAGGAGCAGCAATTTa 3'</td><td>-13.93</td><td>141.00</td><td>?</td></tr>
<tr><td>124</td><td>hsa-miR-936</td><td>451~472</td><td>22</td><td>miRNA: 3' gacgcuaaggagggAGAUGACa 5'
                        |||||||
Target:5' cccagtaatgtatgTCTACTGg 3'</td><td>-8.60</td><td>140.00</td><td>?</td></tr>
</table>

miRNA Target Sites Table View

Target Sequence	><br>GCCCGCGCCAGGGTCCTCGGAGCTGCTCTGGCTGCGCGCGGAGCGGGCTCCGGAGGGAAGTCCCGAGACAAAGGGAAGCG<br>CCGCCGCCGCCGCCCCGCTCGGTCCTCCACCTGTCCGCTACGCTCGCCGGGGCTGCGGCCGCCCGAGGGACTTTGAACAT<br>GTCGGGGATCGCCCTCAGCAGACTCGCCCAGGAGAGGAAAGCATGGAGGAAAGACCACCCATTTGGTTTCGTGGCTGTCC<br>CAACAAAAAATCCCGATGGCACGATGAACCTCATGAACTGGGAGTGCGCCATTCCAGGAAAGAAAGGGACTCCGTGGGAA<br>GGAGGCTTGTTTAAACTACGGATGCTTTTCAAAGATGATTATCCATCTTCGCCACCAAAATGTAAATTCGAACCACCATT<br>ATTTCACCCGAATGTGTACCCTTCGGGGACAGTGTGCCTGTCCATCTTAGAGGAGGACAAGGACTGGAGGCCAGCCATCA<br>CAATCAAACAGATCCTATTAGGAATACAGGAACTTCTAAATGAACCAAATATCCAAGACCCAGCTCAAGCAGAGGCCTAC<br>ACGATTTACTGCCAAAACAGAGTGGAGTACGAGAAAAGGGTCCGAGCACAAGCCAAGAAGTTTGCGCCCTCATAAGCAGC<br>GACCTTGTGGCATCGTCAAAAGGAAGGGATTGGTTTGGCAAGAACTTGTTTACAACATTTTTGCAAATCTAAAGTTGCTC<br>CATACAATGACTAGTCACCTGGGGGGGTTGGGCGGGCGCCATCTTCCATTGCCGCCGCGGGTGTGCGGTCTCGATTCGCT<br>GAATTGCCCGTTTCCATACAGGGTCTCTTCCTTCGGTCTTTTGTATTTTTGATTGTTATGTAAAACTCGCTTTTATTTTA<br>ATATTGATGTCAGTATTTCAACTGCTGTAAAATTATAAACTTTTATACTTGGGTAAGTCCCCCAGGGGCGAGTTCCTCGC<br>TCTGGGATGCAGGCATGCTTCTCACCGTGCAGAGCTGCACTTGGCCTCAGCTGGCTGTATGGAAATGCACCCTCCCTCCT<br>GCCGCTCCTCTCTAGAACCTTCTAGAACCTGGGCTGTGCTGCTTTTGAGCCTCAGACCCCAGGTCAGCATCTCGGTTCTG<br>CGCCACTTCCTTTGTGTTTATATGGCGTTTTGTCTGTGTTGCTGTTTAGAGTAAATAAACTGTTTATATAAAGGTTTTGG<br>TTGCATTATTATCATTGAAAGTGAGAGGAGGCGGCCTCCCAGTGCCCGGCCCTCCCCACCCACCTGCAGCCCCACCGCGG<br>GCCAGGACCAGGCTCTCCATCTGCTTCGGATGCACGCAGGCTGTGAGGCTCTGTCTTGCCCTGGATCTTTGTAAACAGGG<br>CTGTGTACAAAGTGCTGCTGAGGTTTCTGTGCTCCCCGCATCTGCGGGCTGTAGAGCGCTGGGCAGCTAAGATCTGCATA<br>GGTCGGGATTGGCATCGAGACCCTGGCAACTGCACCGGTGCCAGCTGTCTTGGGGGCCACAAGGCCAGGTCCAGACCAGG<br>GCTGGGGGCTGCCTGAGGACTCCTATCCGGGCAGCCTGCTGGCGGGGGTTCCCCTCTTCAGTGGCCAGGTCACAGGGATG<br>GAGCTGCGCTGTGCATAGGGTGCCACCTCAGGTGTCTGTCCCTTGTGTCCTCAGGAGGCAGCCTTGCTACCACCCGTGGC<br>AAACGCCAGGTGCTTTTTCTGGGAGAGCCCACAGCCGTGGCCCTCCAGGGCTTCCCCGACCCTTAGCGCCAGGTAGAGGG<br>CCCTGGGCAGCCTGTGTCTGGAATTCTTCGTCCTGAGGCCACCTGAGTGTGGTCTGTCCTGGGGAGGCTGTGCGCCTCAG<br>CAGCCGTCCTGACGCTGAGCCCTCTGCAAAGGTTGGGCCGGCCAGGCCTCTTGGGGCTGCCTGAGCCACTGCAGGAAGTG<br>GCCTGGCTGGGAAGTTGGGTGCCGGTCACCTCCCAGCAGGAAGGCACAGTGGACAGAGATGGGAAGCCCTGGGGGACACA<br>GCCCGGTGCTCCCAGCCCTCCAACCTCTGGCTCCCAACCCAGTCTCCCCATCCTAGCGAGCTTGGCCCTCCTCAGTTTCG<br>TTTCAAGCCTTGGGGCTGGAGCTGGCCCTGCTGCCCTGGCACCCCCCGGTGGCTGGAGCTGGGTCCCCGTGGCCCAAGTG<br>CAGGGTCCCAAGAGGGCAGGGCGGGGCTCCCCAAAGGAGCAAAGAATGCAGGGAGGGCGGTCCAGGGCCCTGGGAAGGGG<br>AGCTCGGCACCCTCCAGGTCCGTGTGGGACTCCAGCCGCTGTTGGCTGGGAATCGAAGTTAGAGGTGACTTCCAAAGGCC<br>CCCCGAGCCGGCAGTGCCCCCCACCACCCCTCCAGCGACTCTGCGGTGCCAGTGCCTTGTTGGCTTTTCCGGCTACGCAC<br>CCTGCAGTCACTGAGCTCTCGGTCTGACGTCTGATGTTTGTGGTTTGTTTATAACACGGGGCCTTACCTGGGGAATTCAG<br>CTGGTTTGAATATTTGTAGCCCGCTCCCAGAATGTCTTATTTTGTAATGACTGAACTACATTTAGTAATAGTTACACATG<br>TATATGGTTAATACATATGGAAATTCAATATATTTTGTAGTTAACGTATTCTGAAGTAACGGATGTTTCTCGCCAATCGT<br>AGTGACTTCAGCTAACGAAATGTTCTTTTGTAGTACCACGGTCCTCGGCCTAACGAAGGACGTGAACCTTGTAAGAGGAG<br>AGCTCTGAAACGCGGTCACCTTTGTTTAGTGGAAGGGAAAGTGTGTTCCCGGCATGAGGTGCCTCGGAATTAGTAAAGAA<br>TTGTGGGCAATGGATTAACCACTGTATCTAAGAATCCACCATTAAAGCATTTGCACAGACAAAAAAAAAAA

<table>
<tr><th>No.</th><th>miRNA ID</th><th>Location</th><th>Len</th><th>Hybridization</th><th>Minimum Free Energy</th><th>Score</th><th>Profile</th></tr>
<tr><td>1</td><td>hsa-let-7a-2*</td><td>1592~1616</td><td>25</td><td>miRNA: 3' ccUUUCGAUCCUC----
CGACAUGUc 5'
|::| | ||||
|||||:||
Target:5' acAGGGAT-
GGAGCTGCGCTGTGCAt 3'</td><td>-15.80</td><td>141.00</td><td>Show result profile</td></tr>
<tr><td>2</td><td>hsa-let-7f-2*</td><td>1349~1368</td><td>20</td><td>miRNA: 3' ccuuUCUGUCAUCUGACAUAUc
5'
| |||| |:||||:||
Target:5' ttgtAAACAG--GGCTGTGTAc
3'</td><td>-11.70</td><td>142.00</td><td>Show result profile</td></tr>
<tr><td>3</td><td>hsa-let-7i*</td><td>607~629</td><td>23</td><td>miRNA: 3' ucGUUCCG-UCAUCGAACGCGuc
5'
|||| | || ||:||||||
Target:5' caCAAGCCAAGAAGTTTGCGCcc
3'</td><td>-17.70</td><td>152.00</td><td>Show result profile</td></tr>
<tr><td></td><td></td><td></td><td></td><td></td><td></td><td></td><td></td></tr>
</table>

4	hsa-miR-103	1355~1379	25	miRNA: 3' agUAUCGGGACAUG--<br>UUACGACGa 5'<br>| :||: ||||| |:|||||| Target:5'<br>acAGGGCTGTGTACAAAGTGCTGCt 3'	-23.50	155.00	Show result profile
		1060~1083	24	miRNA: 3' aguAUC-GGGACAUGUUACGACGa<br>5'<br>||| |||| :| :|||||| Target:5' ttcTAGAACCTGGGCTGTGCTGCt<br>3'	-18.50	148.00	
5	hsa-miR-103-as	1342~1366	25	miRNA: 3' ucgUCGUAACA--<br>UGUCCCGAUACu 5'<br>| | |||| ||||||||:|| Target:5'<br>tggATCTTTGTAAACAGGGCTGTGt 3'	-20.10	158.00	Show result profile
6	hsa-miR-103-2*	2177~2201	25	miRNA: 3' guUCCGUCGUGA--<br>CAUUUCUUCGa 5'<br>:|||:| :|| ||||:||| Target:5'<br>caGGGCGGGGCTCCCCAAAGGAGCa 3'	-21.50	143.00	Show result profile
7	hsa-miR-103	1355~1379	25	miRNA: 3' agUAUCGGGACAUG--<br>UUACGACGa 5'<br>| :||: ||||| |:|||||| Target:5'<br>acAGGGCTGTGTACAAAGTGCTGCt 3'	-23.50	155.00	Show result profile
		1060~1083	24	miRNA: 3' aguAUC-GGGACAUGUUACGACGa<br>5'<br>||| |||| :| :|||||| Target:5' ttcTAGAACCTGGGCTGTGCTGCt<br>3'	-18.50	148.00	
8	hsa-miR-103-as	1342~1366	25	miRNA: 3' ucgUCGUAACA--<br>UGUCCCGAUACu 5'<br>| | |||| ||||||||:|| Target:5'<br>tggATCTTTGTAAACAGGGCTGTGt 3'	-20.10	158.00	Show result profile
9	hsa-miR-105	2833~2854	22	miRNA: 3' ugguGUCCUCAGACUCGUAAACu<br>5'<br>|| | |: |||||||| Target:5' aatcCACCA-TTAAAGCATTTGc<br>3'	-10.96	146.00	Show result profile
		2833~2854	22	miRNA: 3' ugguGUCCUCAGACUCGUAAACu<br>5'<br>|| | |: |||||||| Target:5' aatcCACCA-TTAAAGCATTTGc<br>3'	-10.96	146.00	
10	hsa-miR-106b*	409~437	29	miRNA: 3' cgUCGUUCAUGGG-------<br>UGUCACGCc 5'<br>| :: |||||| ||||||:| Target:5'<br>cgAATGTGTACCCTTCGGGGACAGTGTGc 3'	-16.91	140.00	Show result profile
				miRNA: 3' acUAUCGGGACAUG--			

11	hsa-miR-107	1355~1379	25	UUACGACGa 5'<br>| :||: ||||| |:|||||| Target:5' acAGGGCTGTGTACAAAGTGCTGCt 3'	-21.60	155.00	Show result profile
		1060~1083	24	miRNA: 3' acuAUC-GGGACAUGUUACGACGa 5'<br>||| |||| :| :|||||| Target:5' ttcTAGAACCTGGGCTGTGCTGCt 3'	-18.10	148.00	
12	hsa-miR-10a	798~825	28	miRNA: 3' guGUUUAA---GCCUAG--AUGUCCCAu 5'<br>::|||| || || ||||||| Target:5' gcTGAATTGCCCGTTTCCATACAGGGTc 3'	-12.40	159.00	Show result profile
13	hsa-miR-10b	798~825	28	miRNA: 3' guGUUUAA---GCCAAG--AUGUCCCAu 5'<br>::|||| || ||| ||||||| Target:5' gcTGAATTGCCCGTTTCCATACAGGGTc 3'	-13.40	167.00	Show result profile
14	hsa-miR-1178	2790~2810	21	miRNA: 3' gaUCCCUUCUUGUCACUCGUu 5'<br>|| |||||: |||:||| Target:5' ttAGTAAAGAATTGTGGGCAa 3'	-16.80	151.00	Show result profile
15	hsa-miR-1179	2187~2211	25	miRNA: 3' gguuGGUUAC----UUUCUUACGaa 5'<br>|||| | ||||||||| Target:5' ctccCCAAAGGAGCAAAGAATGCag 3'	-12.00	143.00	Show result profile
16	hsa-miR-1182	1443~1465	23	miRNA: 3' caguguagggagGGUUCUGGGAg 5'<br>:|:||||||| Target:5' tcgggattggcaTCGAGACCCTg 3'	-15.82	147.00	Show result profile
17	hsa-miR-1183	1244~1271	28	miRNA: 3' acGGGUGAGAGUGGU-AGUGGAUGUCac 5'<br>|| :| ||| ||| |||||:||| Target:5' gcCCGGCCCTCCCCACCCACCTGCAGcc 3'	-28.60	145.00	Show result profile
		2381~2409	29	miRNA: 3' acgGGUGAGAGUGGU-AGU-GGAUGUCAc 5'<br>|: :|| :|:| || |||:|||| Target:5' tggCTTTTCCGGCTACGCACCCTGCAGTc 3'	-18.60	140.00	
18	hsa-miR-1197	1632~1652	21	miRNA: 3' ucuucaucuGGUACACAGGAu 5'<br>|| |||||||| Target:5' gtgtctgtcCCTTGTGTCCTc 3'	-13.30	152.00	Show result profile
19	hsa-miR-	1636~1657	22	miRNA: 3' cuccgagucuuaccGAGUCCUc 5'<br>|||||||	-13.90	140.00	Show result profile

<table>
<tr><td></td><td>1200</td><td></td><td></td><td>Target:5' ctgtcccttgtgtcCTCAGGAg 3'</td><td></td><td></td><td></td></tr>
<tr><td rowspan="3">20</td><td rowspan="3">hsa-miR-1203</td><td>29~54</td><td>26</td><td>miRNA: 3' cuCGACGU-AG----GACCGAGGCCc 5'
|||||: | | ||||||||
Target:5' tgGCTGCGCGCGGAGCGGGCTCCGGa 3'</td><td>-24.60</td><td>153.00</td><td rowspan="3">Show result profile</td></tr>
<tr><td>1292~1310</td><td>19</td><td>miRNA: 3' cucGACGUAGGACCGAGGCCc 5'
|| ||||: |||:|||
Target:5' gctCTCCATCT--GCTTCGGa 3'</td><td>-15.40</td><td>142.00</td></tr>
<tr><td>946~966</td><td>21</td><td>miRNA: 3' cucgacguAGGACCGAGGCCc 5'
|||| ||||:||
Target:5' gggcgagtTCCTCGCTCTGGg 3'</td><td>-24.70</td><td>141.00</td></tr>
<tr><td rowspan="2">21</td><td rowspan="2">hsa-miR-1205</td><td>2391~2408</td><td>18</td><td>miRNA: 3' gaGUUUCGUUUGGGACGUCu 5'
| | || |||||||||
Target:5' ggCTACGC--ACCCTGCAGt 3'</td><td>-18.70</td><td>154.00</td><td rowspan="2">Show result profile</td></tr>
<tr><td>1250~1270</td><td>21</td><td>miRNA: 3' gaguuucGUUUG-GGACGUCu 5'
|| | |||||||
Target:5' ccctcccCACCCACCTGCAGc 3'</td><td>-12.73</td><td>141.00</td></tr>
<tr><td rowspan="2">22</td><td rowspan="2">hsa-miR-1206</td><td>262~279</td><td>18</td><td>miRNA: 3' cgaAUUUGUAGAUGUACUUGu 5'
|:||| || |||||||
Target:5' cgaTGAAC--CT-CATGAACt 3'</td><td>-9.30</td><td>153.00</td><td rowspan="2">Show result profile</td></tr>
<tr><td>506~526</td><td>21</td><td>miRNA: 3' cgaaUUUGUAGAUGUACUUGu 5'
:||| |||| ||||||
Target:5' acagGAACTTCTAAATGAACc 3'</td><td>-10.70</td><td>141.00</td></tr>
<tr><td>23</td><td>hsa-miR-1207-3p</td><td>1471~1488</td><td>18</td><td>miRNA: 3' cuuuacUCCCGGUCGACu 5'
:| |||||||
Target:5' tgcaccGGTGCCAGCTGt 3'</td><td>-16.20</td><td>148.00</td><td>Show result profile</td></tr>
<tr><td>24</td><td>hsa-miR-122*</td><td>1128~1149</td><td>22</td><td>miRNA: 3' auaAAUCACACUAU-UACCGCAa 5'
|| |||| || |||||||
Target:5' tccTTTGTGT-TTATATGGCGTt 3'</td><td>-14.00</td><td>158.00</td><td>Show result profile</td></tr>
<tr><td>25</td><td>hsa-miR-1224-5p</td><td>1636~1654</td><td>19</td><td>miRNA: 3' ggUGGAGGGCUCAGGAGUg 5'
::| |::| |||||||
Target:5' ctGTCCCTTGTGTCCTCAg 3'</td><td>-17.60</td><td>153.00</td><td>Show result profile</td></tr>
<tr><td rowspan="2">26</td><td rowspan="2">hsa-miR-1225-3p</td><td>2170~2190</td><td>21</td><td>miRNA: 3' gaCCCCCGCCGUGUCCCCGAGu 5'
| |||| | :| |||||||
Target:5' aaGAGGGCAGGGC-GGGGCTCc 3'</td><td>-24.00</td><td>159.00</td><td rowspan="2">Show result profile</td></tr>
<tr><td></td><td></td><td></td><td></td><td></td></tr>
</table>

<table>
<tr><td></td><td></td><td>1547~1572</td><td>26</td><td>miRNA: 3' gaCCC--CCGCCG--
UGUCCCCGAGu 5'
||| | | ||
:|:||||:||
Target:5'
ccGGGCAGCCTGCTGGCGGGGGTTCc 3'</td><td>-22.60</td><td>140.00</td><td></td></tr>
<tr><td rowspan="9">27</td><td rowspan="9">hsa-miR-1226*</td><td>607~633</td><td>27</td><td>miRNA: 3' ggGGUAGG-UC--
CGGACGUACGGGAGUg 5'
| | || || |::|||
|||||||
Target:5' caCAAGCCAAGAAGTTTGC--
GCCCTCAt 3'</td><td>-19.70</td><td>158.00</td><td rowspan="9">Show result profile</td></tr>
<tr><td>2046~2072</td><td>27</td><td>miRNA: 3' ggGGUAGGUCCGGACGUA-
CGGGAGug 5'
|||||| || || |
||||||
Target:5'
ccCCATCCTAGCGAGCTTGGCCCTCct 3'</td><td>-30.40</td><td>152.00</td></tr>
<tr><td>155~178</td><td>24</td><td>miRNA: 3' gggGUAGGUCCGGACGUA-
CGGGAGUg 5'
||| : || | ||
|||||||
Target:5' gaaCATGTCGG---
GGATCGCCCTCAg 3'</td><td>-17.70</td><td>152.00</td></tr>
<tr><td>983~1010</td><td>28</td><td>miRNA: 3' ggGGUAGGUCCGGACGU--
ACGGGAGUg 5'
||:| ||| ||||| ||
|||||
Target:5'
caCCGTGCAGAGCTGCACTTGGCCTCAg 3'</td><td>-26.50</td><td>150.00</td></tr>
<tr><td>1621~1654</td><td>34</td><td>miRNA: 3' ggGGUAG-GUC--CGGAC-----
GUACGGGAGUg 5'
||| | ||| |:|||
::||:|||||
Target:5'
tgCCACCTCAGGTGTCTGTCCCTTGTGTCCTCAg
3'</td><td>-25.30</td><td>150.00</td></tr>
<tr><td>1842~1866</td><td>25</td><td>miRNA: 3' ggGGUAGGUCCG-
GACGUACGGGAGug 5'
||:||| | | ||| |
||||||
Target:5' agCCGTCCTGACGCTG-A-
GCCCTCtg 3'</td><td>-25.10</td><td>146.00</td></tr>
<tr><td>1996~2022</td><td>27</td><td>miRNA: 3' gggGUAGGUCCG-
GACGUACGGGAGug 5'
|| || ||: || |
||||||
Target:5'
acaCAGCCCGGTGCTCCCAGCCCTCca 3'</td><td>-20.70</td><td>143.00</td></tr>
<tr><td>1233~1256</td><td>24</td><td>miRNA: 3' ggGGUAGGUC-
CGGACGUACGGGAGug 5'
|| |||| ||| |
||||||
Target:5' ggCCTCCCAGTGCCCG---
GCCCTCcc 3'</td><td>-26.60</td><td>141.00</td></tr>
<tr><td></td><td></td><td></td><td></td><td></td></tr>
<tr><td>28</td><td>hsa-miR-</td><td>2468~2487</td><td>20</td><td>miRNA: 3' gaUCCCUUGUGUCCCGACCAcu
5'</td><td>-20.20</td><td>144.00</td><td>Show result profile</td></tr>
</table>

<table>
<tr><td></td><td>1226</td><td></td><td></td><td>:|||||: || ||||||
Target:5' ctGGGGAATTCA--GCTGGTtt
3'</td><td></td><td></td><td></td></tr>
<tr><td>29</td><td>hsa-miR-1228*</td><td>78~97</td><td>20</td><td>miRNA: 3' guguGUGGACGGGGGCGGGug 5'
|:|| ||| ||||||
Target:5' gcgcCGCC-GCCGCCGCCCcg 3'</td><td>-30.30</td><td>140.00</td><td>Show result profile</td></tr>
<tr><td>30</td><td>hsa-miR-1228</td><td>767~786</td><td>20</td><td>miRNA: 3' ccccCCGCUCCGUCCACACu 5'
| || ||:||||||
Target:5' cattGCCGCCGCGGGTGTGc 3'</td><td>-16.60</td><td>140.00</td><td>Show result profile</td></tr>
<tr><td rowspan="2">31</td><td rowspan="2">hsa-miR-1233</td><td>2170~2190</td><td>21</td><td>miRNA: 3' gaCGCCC-UCCUGUCCCGAGu 5'
| ||| |||:|:||||||
Target:5' aaGAGGGCAGGGCGGGGCTCc 3'</td><td>-27.90</td><td>154.00</td><td rowspan="2">Show result profile</td></tr>
<tr><td>2170~2190</td><td>21</td><td>miRNA: 3' gaCGCCC-UCCUGUCCCGAGu 5'
| ||| |||:|:||||||
Target:5' aaGAGGGCAGGGCGGGGCTCc 3'</td><td>-27.90</td><td>154.00</td></tr>
<tr><td>32</td><td>hsa-miR-1234</td><td>1868~1890</td><td>23</td><td>miRNA: 3' cacCCCACCC-ACCAGUCCGGcu
5'
|| |||| || ||||||
Target:5' aaaGGTTGGGCCGGCCAGGCCtc
3'</td><td>-21.10</td><td>143.00</td><td>Show result profile</td></tr>
<tr><td>33</td><td>hsa-miR-1236</td><td>60~81</td><td>22</td><td>miRNA: 3' gaccuCUCUGUUCCCCUUCUCc
5'
||||||| |||||| |
Target:5' gtcccGAGACAAAGGGAAGCGc
3'</td><td>-17.82</td><td>145.00</td><td>Show result profile</td></tr>
<tr><td rowspan="3">34</td><td rowspan="3">hsa-miR-124</td><td>2358~2379</td><td>22</td><td>miRNA: 3' ccguAAGUGGCG--CACGGAAu
5'
| |: :|| |||||||
Target:5' actcTGCGGTGCCAGTGCCTTg
3'</td><td>-16.00</td><td>146.00</td><td rowspan="3">Show result profile</td></tr>
<tr><td>2358~2379</td><td>22</td><td>miRNA: 3' ccguAAGUGGCG--CACGGAAu
5'
| |: :|| |||||||
Target:5' actcTGCGGTGCCAGTGCCTTg
3'</td><td>-16.00</td><td>146.00</td></tr>
<tr><td>2358~2379</td><td>22</td><td>miRNA: 3' ccguAAGUGGCG--CACGGAAu
5'
| |: :|| |||||||
Target:5' actcTGCGGTGCCAGTGCCTTg
3'</td><td>-16.00</td><td>146.00</td></tr>
<tr><td rowspan="2"></td><td rowspan="2"></td><td>2515~2541</td><td>27</td><td>miRNA: 3' uuGGUAGAGUAUGUUUGG-
UUGAUGaa 5'
::||:|::|| :||:
||||||
Target:5'
tcTTATTTTGTAATGACTGAACTACat 3'</td><td>-8.80</td><td>148.00</td><td rowspan="2"></td></tr>
<tr><td></td><td></td><td>miRNA: 3'</td><td></td><td></td></tr>
</table>

35	hsa-miR-1244	883~907	25	uuggUAGAGUAUGUUUGGUUGAUGAa 5' || ||| ::| :|||||:|| Target:5' attgATGTCA-GTATTTCAACTGCTg 3'	-12.80	145.00	Show result profile
		2515~2541	27	miRNA: 3' uuGGUAGAGUAUGUUUGG-UUGAUGaa 5' ::||:|::|| :||: |||||| Target:5' tcTTATTTTGTAATGACTGAACTACat 3'	-8.80	148.00	
		883~907	25	miRNA: 3' uuggUAGAGUAUGUUUGGUUGAUGAa 5' || ||| ::| :|||||:|| Target:5' attgATGTCA-GTATTTCAACTGCTg 3'	-12.80	145.00	
		2515~2541	27	miRNA: 3' uuGGUAGAGUAUGUUUGG-UUGAUGaa 5' ::||:|::|| :||: |||||| Target:5' tcTTATTTTGTAATGACTGAACTACat 3'	-8.80	148.00	
		883~907	25	miRNA: 3' uuggUAGAGUAUGUUUGGUUGAUGAa 5' || ||| ::| :|||||:|| Target:5' attgATGTCA-GTATTTCAACTGCTg 3'	-12.80	145.00	
36	hsa-miR-1246	2821~2840	20	miRNA: 3' ggACG-AGGUUUUUAGGUaa 5' ||: ||:||:|||||| Target:5' acTGTATCTAAGAATCCAcc 3'	-16.50	145.00	Show result profile
37	hsa-miR-1247	738~757	20	miRNA: 3' agGCCCCUGCUUGCCCUGCCCa 5' :|||| |::: ||:|||| Target:5' ccTGGGG--GGGTTGGGCGGGc 3'	-28.80	140.00	Show result profile
38	hsa-miR-1248	2148~2177	30	miRNA: 3' aaAUCGUG-UCACG-AAUA-UGUUCUUCCa 5' |:|| | ||||| :| |||||:|| Target:5' cgTGGCCCAAGTGCAGGGTCCCAAGAGGGc 3'	-18.30	145.00	Show result profile
39	hsa-miR-1249	2200~2220	21	miRNA: 3' acUUCUUCCCCCCCUUCCCGCa 5' ||||| | ||:|||||| Target:5' caAAGAA-TGCAGGGAGGGCGg 3'	-19.10	147.00	Show result profile
40	hsa-miR-1251	2084~2102	19	miRNA: 3' ucgCGGAAACCGUCGAUCUCa 5' ||||| || |||:||| Target:5' caaGCCTTGGG--GCTGGAGc 3'	-19.10	146.00	Show result profile
				miRNA: 3' ucGCGGAAACCG----------			

		2275~2305	31	UCGAUCUCa 5' |||: ||||| ||:||||| Target:5' gcCGCTGTTGGCTGGGAATCGAAGTTAGAGg 3'	-18.30	141.00	
41	hsa-miR-1252	816~835	20	miRNA: 3' auuuaCUUA-AGUUAAAGGAAGa 5' |::| || ||||||| Target:5' atacaGGGTCTC---TTCCTTCg 3'	-7.80	142.00	Show result profile
42	hsa-miR-1254	2235~2260	26	miRNA: 3' ugacgucCGAG--GUCGAAGGUCCGa 5' |||| || | ||||||: Target:5' aaggggaGCTCGGCACCCTCCAGGTc 3'	-17.90	143.00	Show result profile
		1532~1553	22	miRNA: 3' ugACGUCCGAGGUCGAAGGUCCGa 5' || :| |||| : |||:||| Target:5' ccTGAGGACTCC--TATCCGGGCa 3'	-22.60	142.00	
		278~300	23	miRNA: 3' ugaCGUC-CGAGGUCGAAGGUCCga 5' | || || ||| ||||||| Target:5' ctgGGAGTGCGCCA--TTCCAGGaa 3'	-18.70	141.00	
43	hsa-miR-125a-3p	716~742	27	miRNA: 3' ccGAGG---GUUCUUGG--AGUGGACa 5' |||| ||| :||: ||||||| Target:5' tgCTCCATACAATGACTAGTCACCTGg 3'	-19.00	162.00	Show result profile
		2449~2471	23	miRNA: 3' ccgaggGU-UCUUGGAGUGGACa 5' || :|::|||:||||| Target:5' ttataaCACGGGGCCTTACCTGg 3'	-19.60	144.00	
44	hsa-miR-126*	2530~2553	24	miRNA: 3' gcGCAUGGUUU---UCAUUAUUAc 5' :| ||:| | |||||||:| Target:5' acTGAACTACATTTAGTAATAGTt 3'	-7.60	143.00	Show result profile
45	hsa-miR-1260	1967~1985	19	miRNA: 3' acCACC-GUCUCCACCCUa 5' |||| ||||| ||||| Target:5' caGTGGACAGAGATGGGAa 3'	-20.60	140.00	Show result profile
46	hsa-miR-1261	346~366	21	miRNA: 3' uucGGUUUCGG--AAUAGGUa 5' :||||| : ||||||| Target:5' tttTCAAAGATGATTATCCAt 3'	-13.40	154.00	Show result profile
47	hsa-miR-1262	170~191	22	miRNA: 3' uaGGAAGAUGUUUAAGUGGGUa 5' || || :||:| ||:|||| Target:5' cgCCCTCAGCAGACTCGCCCAg	-17.90	152.00	Show result profile

<table>
<tr><td></td><td></td><td></td><td></td><td>3'</td><td></td><td></td><td></td></tr>
<tr><td>48</td><td>hsa-miR-1263</td><td>1604~1626</td><td>23</td><td>miRNA: 3' ugaGUCAUACG-GUCCCAUGGUa 5'
|: |:||| :|||||:|||
Target:5' ctgCGCTGTGCATAGGGTGCCAc 3'</td><td>-22.10</td><td>151.00</td><td>Show result profile</td></tr>
<tr><td rowspan="3">49</td><td rowspan="3">hsa-miR-1266</td><td>1774~1798</td><td>25</td><td>miRNA: 3' ucgGGAC---AAGAUGUCGGGACUCc 5'
:||| |||| | |:||||||
Target:5' gtgTCTGGAATTCTTC-GTCCTGAGg 3'</td><td>-22.40</td><td>147.00</td><td rowspan="3">Show result profile</td></tr>
<tr><td>2213~2235</td><td>23</td><td>miRNA: 3' ucgggaCAAGAUGUCGGGACUCc 5'
| || | :||||||:|
Target:5' gagggcGGTCCAGGGCCCTGGGa 3'</td><td>-18.30</td><td>141.00</td></tr>
<tr><td>1746~1768</td><td>23</td><td>miRNA: 3' ucgGGACAAGAUGUCGGGACUCc 5'
|| | | | :||||||:|
Target:5' gcgCCAGGTAGAGGGCCCTGGGc 3'</td><td>-23.00</td><td>140.00</td></tr>
<tr><td rowspan="2">50</td><td rowspan="2">hsa-miR-1267</td><td>163~182</td><td>20</td><td>miRNA: 3' acCCCUAAUGUGAAGUUGUCc 5'
||||| :| | |||:|||
Target:5' cgGGGATCGC-CCTCAGCAGa 3'</td><td>-18.50</td><td>146.00</td><td rowspan="2">Show result profile</td></tr>
<tr><td>1820~1844</td><td>25</td><td>miRNA: 3' acCCCU---A-AUGUGAAGUUGUCc 5'
|||| | |:|:| |||:|||
Target:5' tgGGGAGGCTGTGCGCCTCAGCAGc 3'</td><td>-22.20</td><td>143.00</td></tr>
<tr><td rowspan="2">51</td><td rowspan="2">hsa-miR-1271</td><td>1603~1627</td><td>25</td><td>miRNA: 3' acuCACGA-ACG-AU-CCACGGUuc 5'
| ||| ||| || |||||||
Target:5' gctGCGCTGTGCATAGGGTGCCAcc 3'</td><td>-14.90</td><td>143.00</td><td rowspan="2">Show result profile</td></tr>
<tr><td>2352~2373</td><td>22</td><td>miRNA: 3' acUCAC-GA-ACGAUCCACGGUUc 5'
|| | || ||| |||||||:
Target:5' ccAGCGACTCTGC--GGTGCCAGt 3'</td><td>-14.30</td><td>140.00</td></tr>
<tr><td>52</td><td>hsa-miR-1272</td><td>1188~1216</td><td>29</td><td>miRNA: 3' aaagUCUUAAA-CGACG--GUAGUAGUAg 5'
||: ||| |:||| :||:|||||
Target:5' ataaAGGTTTTGGTTGCATTATTATCATt 3'</td><td>-15.10</td><td>156.00</td><td>Show result profile</td></tr>
<tr><td></td><td>hsa-</td><td>526~552</td><td>27</td><td>miRNA: 3' aggUGAAGGACC-----CAAGUUCGUu 5'
|: ||| | | |||||||
Target:5' caaATATCCAAGACCCAGCTCAAGCAg 3'</td><td>-14.00</td><td>143.00</td><td></td></tr>
</table>

<table>
<tr><td rowspan="2">53</td><td rowspan="2">miR-1273e</td><td></td><td></td><td></td><td></td><td></td><td rowspan="2">Show result profile</td></tr>
<tr><td>2071~2090</td><td>20</td><td>miRNA: 3' agGUGAAGGACCCAAGUUCGuu 5'
                 || ||:| | |||||||
Target:5' ctCAGTTTC--GTTTCAAGCct 3'</td><td>-10.90</td><td>140.00</td></tr>
<tr><td>54</td><td>hsa-miR-1274a</td><td>1580~1599</td><td>20</td><td>miRNA: 3' accgCGGACU--UGUCCCUg 5'
                   ||| |: |||||||
Target:5' agtgGCCAGGTCACAGGGAt 3'</td><td>-18.90</td><td>148.00</td><td>Show result profile</td></tr>
<tr><td>55</td><td>hsa-miR-1274b</td><td>1345~1361</td><td>17</td><td>miRNA: 3' accgcgggcUUGUCCCu 5'
                        |||||||
Target:5' atctttgtaAACAGGGc 3'</td><td>-10.00</td><td>140.00</td><td>Show result profile</td></tr>
<tr><td>56</td><td>hsa-miR-1275</td><td>929~945</td><td>17</td><td>miRNA: 3' cugucggagAGGGGGUg 5'
                        |||||||
Target:5' ttgggtaagTCCCCCAg 3'</td><td>-12.90</td><td>140.00</td><td>Show result profile</td></tr>
<tr><td>57</td><td>hsa-miR-1281</td><td>1215~1234</td><td>20</td><td>miRNA: 3' ccCUCUC---CUCCUCCGCu 5'
                 || || |||||||||
Target:5' ttGAAAGTGAGAGGAGGCGg 3'</td><td>-20.50</td><td>155.00</td><td>Show result profile</td></tr>
<tr><td>58</td><td>hsa-miR-1282</td><td>1667~1686</td><td>20</td><td>miRNA: 3' uucgucuuuuuCCGUUUGCu 5'
                          ||||||||
Target:5' ctaccacccgtGGCAAACGc 3'</td><td>-10.90</td><td>145.00</td><td>Show result profile</td></tr>
<tr><td rowspan="5">59</td><td rowspan="5">hsa-miR-1283</td><td>2655~2674</td><td>20</td><td>miRNA: 3' ucUUUCGCGAAAGGAAACAUCu 5'
                 :|| :| |||:|||||||
Target:5' acGAAATG--TTCTTTTGTAGt 3'</td><td>-14.20</td><td>160.00</td><td rowspan="5">Show result profile</td></tr>
<tr><td>2475~2500</td><td>26</td><td>miRNA: 3' ucuuUCG-CGAAA---GGAAACAUCu 5'
                   ||| | ||| :|||||||
Target:5' attcAGCTGGTTTGAATATTTGTAGc 3'</td><td>-8.00</td><td>150.00</td></tr>
<tr><td>2580~2601</td><td>22</td><td>miRNA: 3' ucUUUCGCGAAAGGAAACAUCu 5'
                 || | | | :|||||||
Target:5' gaAATTCAATATATTTTGTAGt 3'</td><td>-7.10</td><td>148.00</td></tr>
<tr><td>2655~2674</td><td>20</td><td>miRNA: 3' ucUUUCGCGAAAGGAAACAUCu 5'
                 :|| :| |||:|||||||
Target:5' acGAAATG--TTCTTTTGTAGt 3'</td><td>-14.20</td><td>160.00</td></tr>
<tr><td>2475~2500</td><td>26</td><td>miRNA: 3' ucuuUCG-CGAAA---GGAAACAUCu 5'
                   ||| | ||| :|||||||
Target:5' attcAGCTGGTTTGAATATTTGTAGc 3'</td><td>-8.00</td><td>150.00</td></tr>
</table>

<table>
<tr><td></td><td></td><td>2580~2601</td><td>22</td><td>miRNA: 3' ucUUUCGCGAAAGGAAACAUCu 5'
|| | | | :|||||||
Target:5' gaAATTCAATATATTTTGTAGt 3'</td><td>-7.10</td><td>148.00</td><td></td></tr>
<tr><td>60</td><td>hsa-miR-1286</td><td>1102~1122</td><td>21</td><td>miRNA: 3' ucccgaGUAGAACCAGGACGu 5'
||||| |||:||||
Target:5' ggtcagCATCTCGGTTCTGCg 3'</td><td>-21.60</td><td>151.00</td><td>Show result profile</td></tr>
<tr><td>61</td><td>hsa-miR-1288</td><td>2205~2224</td><td>20</td><td>miRNA: 3' agAGGUCUAGUCCCGUCAGGu 5'
| |||: |||||:||||
Target:5' aaTGCAGG-GAGGGCGGTCCa 3'</td><td>-26.00</td><td>146.00</td><td>Show result profile</td></tr>
<tr><td rowspan="6">62</td><td rowspan="6">hsa-miR-1289</td><td>2249~2274</td><td>26</td><td>miRNA: 3' uuuuACGUCUAAGGA---CCUGAGGu 5'
| |||:| | | |||||||
Target:5' acccTCCAGGTCCGTGTGGGACTCCa 3'</td><td>-20.90</td><td>155.00</td><td rowspan="6">Show result profile</td></tr>
<tr><td>1519~1544</td><td>26</td><td>miRNA: 3' uuuuACGUCU-AAGGA--CCUGAGGu 5'
|| :|: | ||| |||||||
Target:5' gggcTGGGGGCTGCCTGAGGACTCCt 3'</td><td>-17.50</td><td>153.00</td></tr>
<tr><td>281~314</td><td>34</td><td>miRNA: 3' uuUUACGU-CUAAGG----------ACCUGAGGu 5'
|:|||: ||||| |||||||
Target:5' ggAGTGCGCCATTCCAGGAAAGAAAGGGACTCCg 3'</td><td>-21.70</td><td>147.00</td></tr>
<tr><td>2249~2274</td><td>26</td><td>miRNA: 3' uuuuACGUCUAAGGA---CCUGAGGu 5'
| |||:| | | |||||||
Target:5' acccTCCAGGTCCGTGTGGGACTCCa 3'</td><td>-20.90</td><td>155.00</td></tr>
<tr><td>1519~1544</td><td>26</td><td>miRNA: 3' uuuuACGUCU-AAGGA--CCUGAGGu 5'
|| :|: | ||| |||||||
Target:5' gggcTGGGGGCTGCCTGAGGACTCCt 3'</td><td>-17.50</td><td>153.00</td></tr>
<tr><td>281~314</td><td>34</td><td>miRNA: 3' uuUUACGU-CUAAGG----------ACCUGAGGu 5'
|:|||: ||||| |||||||
Target:5' ggAGTGCGCCATTCCAGGAAAGAAAGGGACTCCg 3'</td><td>-21.70</td><td>147.00</td></tr>
<tr><td></td><td>hsa-</td><td>2819~2838</td><td>20</td><td>miRNA: 3' aggGAC-UAGGUUUUUAGGu 5'
||| |||:||:|||||
Target:5' ccaCTGTATCTAAGAATCCa 3'</td><td>-19.60</td><td>152.00</td><td></td></tr>
</table>

63	miR-1290	232~254	23	miRNA: 3' aggGACUAGG-----UUUUUAGGu 5'<br>||| ||| |||||||| Target:5' tggCTG-TCCCAACAAAAAATCCc 3'	-10.90	151.00	Show result profile
64	hsa-miR-1291	803~826	24	miRNA: 3' ugACGACCAGAAGUCAGUCCCGGu 5'<br>||| ||:| || |||||:| Target:5' atTGCCCGTTTCCATACAGGGTCt 3'	-18.10	150.00	Show result profile
		2205~2230	26	miRNA: 3' ugACG-ACCAGAAGUCA-GUCCCGGu 5'<br>||| || |:|| ||||||| Target:5' aaTGCAGGGAGGGCGGTCCAGGGCCc 3'	-22.90	150.00	
		1337~1363	27	miRNA: 3' ugacGACC-AGAAGUCA---GUCCCGGu 5'<br>|||| ||||: || ||||||:<br>Target:5' tgccCTGGATCTTT-GTAAACAGGGCTg 3'	-19.50	147.00	
		2440~2464	25	miRNA: 3' ugaCGACCAGA-AGUCAGUCCCGGu 5'<br>|:| ||:| | | |:||||| Target:5' gtgGTTTGTTTATAACACGGGGCCt 3'	-16.46	141.00	
		1502~1524	23	miRNA: 3' ugacGACCAGAAGUC-AGUCCCGGu 5'<br>| |||| ||| ||||||:<br>Target:5' aggcCAGGTC--CAGACCAGGGCTg 3'	-18.30	140.00	
65	hsa-miR-1292	789~816	28	miRNA: 3' gucGC-AGACGGCCU--UGGGCAAGGGu 5'<br>|| |: ||:| | :||||||:|| Target:5' tctCGATTCGCTGAATTGCCCGTTTCCa 3'	-24.30	148.00	Show result profile
66	hsa-miR-1293	2329~2350	22	miRNA: 3' cgUGUUUAGAGGUCUGGUGGGu 5'<br>:||: | || ||||||| Target:5' cgGCAGTGCCCCCCACCACCCc 3'	-16.70	152.00	Show result profile
		200~221	22	miRNA: 3' cguguuuagaggUCUGGUGGGu 5'<br>||||||||| Target:5' agcatggaggaaAGACCACCCa 3'	-23.50	150.00	
		1655~1676	22	miRNA: 3' cguguUUAGAGGUCUGGUGGGu 5'<br>|: ||: ||||||| Target:5' gaggcAGCCTTGCTACCACCCg 3'	-16.10	145.00	

<table>
<tr><td rowspan="1">67</td><td rowspan="1">hsa-miR-1294</td><td>256~275</td><td>20</td><td>miRNA: 3' ucuguUGUUACGGUUGGAGUGu 5'
||:||| |||||||:
Target:5' atggcACGATG--AACCTCATg 3'</td><td>-16.10</td><td>145.00</td><td>Show result profile</td></tr>
<tr><td>68</td><td>hsa-miR-1295</td><td>2673~2693</td><td>21</td><td>miRNA: 3' agUGGGUCUAGACGCCGGAUu 5'
||| |:|| |||||||
Target:5' gtACCACGGTCCTCGGCCTAa 3'</td><td>-22.80</td><td>159.00</td><td>Show result profile</td></tr>
<tr><td rowspan="3">69</td><td rowspan="3">hsa-miR-1296</td><td>2210~2233</td><td>24</td><td>miRNA: 3' ccUCUACCUC--GGUCCCGGGAUu 5'
:|| || | ||||||||||:
Target:5' agGGAGGGCGGTCCAGGGCCCTGg 3'</td><td>-32.60</td><td>154.00</td><td rowspan="3">Show result profile</td></tr>
<tr><td>2092~2111</td><td>20</td><td>miRNA: 3' ccUCUACCUCGGUCCCGGGAUu 5'
:| ||||||: ||||||:
Target:5' ggGGCTGGAGCT--GGCCCTGc 3'</td><td>-26.20</td><td>148.00</td></tr>
<tr><td>1749~1766</td><td>18</td><td>miRNA: 3' ccUCUACCUCGGUCCCGGGAUu 5'
||:| || ||||||||:
Target:5' ccAGGT--AG--AGGGCCCTGg 3'</td><td>-19.00</td><td>140.00</td></tr>
<tr><td>70</td><td>hsa-miR-1299</td><td>1045~1067</td><td>23</td><td>miRNA: 3' agGGAGUGUGUCUU--AAGGUCUu 5'
|||| | :|||| |||:|||
Target:5' ctCCTCTC-TAGAACCTTCTAGAa 3'</td><td>-16.90</td><td>149.00</td><td>Show result profile</td></tr>
<tr><td rowspan="2">71</td><td rowspan="2">hsa-miR-1301</td><td>969~1000</td><td>32</td><td>miRNA: 3' cuUCAGU-----GAG-GGUCCG--UCGACGUu 5'
|| || ||| ||: || |||||||
Target:5' gcAGGCATGCTTCTCACCGTGCAGAGCTGCAc 3'</td><td>-17.50</td><td>156.00</td><td rowspan="2">Show result profile</td></tr>
<tr><td>1586~1609</td><td>24</td><td>miRNA: 3' cuUCAGUGAGGGUCCGUCGACGUu 5'
:||||| | | ||||||:
Target:5' caGGTCACAGGGATGGAGCTGCGc 3'</td><td>-18.60</td><td>142.00</td></tr>
<tr><td rowspan="3"></td><td rowspan="3"></td><td>222~243</td><td>22</td><td>miRNA: 3' aaAUCGUAUUCAU--ACAGGGUu 5'
|:|: | ||: |||||||
Target:5' ttTGGT-TTCGTGGCTGTCCCAa 3'</td><td>-13.70</td><td>144.00</td><td rowspan="3"></td></tr>
<tr><td>222~243</td><td>22</td><td>miRNA: 3' aaAUCGUAUUCAU--ACAGGGUu 5'
|:|: | ||: |||||||
Target:5' ttTGGT-TTCGTGGCTGTCCCAa 3'</td><td>-13.70</td><td>144.00</td></tr>
<tr><td></td><td></td><td></td><td></td><td></td></tr>
</table>

<table>
<tr><td rowspan="9">72</td><td rowspan="9">hsa-miR-1302</td><td>222~243</td><td>22</td><td>miRNA: 3' aaAUCGUAUUCAU--ACAGGGUu 5'
|:|: | ||: |||||||
Target:5' ttTGGT-TTCGTGGCTGTCCCAa 3'</td><td>-13.70</td><td>144.00</td><td rowspan="9">Show result profile</td></tr>
<tr><td>222~243</td><td>22</td><td>miRNA: 3' aaAUCGUAUUCAU--ACAGGGUu 5'
|:|: | ||: |||||||
Target:5' ttTGGT-TTCGTGGCTGTCCCAa 3'</td><td>-13.70</td><td>144.00</td></tr>
<tr><td>222~243</td><td>22</td><td>miRNA: 3' aaAUCGUAUUCAU--ACAGGGUu 5'
|:|: | ||: |||||||
Target:5' ttTGGT-TTCGTGGCTGTCCCAa 3'</td><td>-13.70</td><td>144.00</td></tr>
<tr><td>222~243</td><td>22</td><td>miRNA: 3' aaAUCGUAUUCAU--ACAGGGUu 5'
|:|: | ||: |||||||
Target:5' ttTGGT-TTCGTGGCTGTCCCAa 3'</td><td>-13.70</td><td>144.00</td></tr>
<tr><td>222~243</td><td>22</td><td>miRNA: 3' aaAUCGUAUUCAU--ACAGGGUu 5'
|:|: | ||: |||||||
Target:5' ttTGGT-TTCGTGGCTGTCCCAa 3'</td><td>-13.70</td><td>144.00</td></tr>
<tr><td>222~243</td><td>22</td><td>miRNA: 3' aaAUCGUAUUCAU--ACAGGGUu 5'
|:|: | ||: |||||||
Target:5' ttTGGT-TTCGTGGCTGTCCCAa 3'</td><td>-13.70</td><td>144.00</td></tr>
<tr><td>222~243</td><td>22</td><td>miRNA: 3' aaAUCGUAUUCAU--ACAGGGUu 5'
|:|: | ||: |||||||
Target:5' ttTGGT-TTCGTGGCTGTCCCAa 3'</td><td>-13.70</td><td>144.00</td></tr>
<tr><td>222~243</td><td>22</td><td>miRNA: 3' aaAUCGUAUUCAU--ACAGGGUu 5'
|:|: | ||: |||||||
Target:5' ttTGGT-TTCGTGGCTGTCCCAa 3'</td><td>-13.70</td><td>144.00</td></tr>
<tr><td>222~243</td><td>22</td><td>miRNA: 3' aaAUCGUAUUCAU--ACAGGGUu 5'
|:|: | ||: |||||||
Target:5' ttTGGT-TTCGTGGCTGTCCCAa 3'</td><td>-13.70</td><td>144.00</td></tr>
<tr><td>73</td><td>hsa-miR-1304</td><td>990~1011</td><td>22</td><td>miRNA: 3' gugUAGA-GUGACAUCGGAGUUu 5'
| || |||| |:||||||:
Target:5' cagAGCTGCACT-TGGCCTCAGc 3'</td><td>-16.70</td><td>146.00</td><td>Show result profile</td></tr>
<tr><td></td><td></td><td>110~132</td><td>23</td><td>miRNA: 3' guGCUGGC--UGCGGUGCGGCUCa 5'</td><td>-24.10</td><td>145.00</td><td></td></tr>
</table>

74	hsa-miR-1307			:| ||| ||||: |||||:| Target:5' ccTGTCCGCTACGCT-CGCCGGGg 3'			Show result profile
		1839~1860	22	miRNA: 3' guGCUGGCUGCGGUGCGGCUCa 5'<br>| :||| | : ||||:||| Target:5' agCAGCCGTCCTGACGCTGAGc 3'	-19.30	144.00	
75	hsa-miR-130b*	281~305	25	miRNA: 3' caUCACGUUGU----CCCUUUCUca 5'<br>|||||: || ||||||| Target:5' ggAGTGCGCCATTCCAGGAAAGAaa 3'	-13.90	141.00	Show result profile
76	hsa-miR-1321	1020~1038	19	miRNA: 3' uagUGUAAGU-GGAGGGAc 5'<br>| || || ||||||| Target:5' tggAAATGCACCCTCCCTc 3'	-13.00	151.00	Show result profile
77	hsa-miR-1324	1134~1157	24	miRNA: 3' cuuucACGUAUCUUAAGACAGACc 5'<br>| :||:| :||:|||||| Target:5' gtgttTATATGGCGTTTTGTCTGt 3'	-14.20	151.00	Show result profile
78	hsa-miR-134	2384~2412	29	miRNA: 3' ggGGAGACCAGU---UG----GUCAGUGu 5'<br>::|| || :| || ||||||| Target:5' ctTTTCCGGCTACGCACCCTGCAGTCACt 3'	-15.00	146.00	Show result profile
		2720~2740	21	miRNA: 3' gggGAGACCAGUUG-GUCAGUGu 5'<br>||||| ||| |:||||| Target:5' gagCTCTG--AAACGCGGTCACc 3'	-18.10	143.00	
		2022~2047	26	miRNA: 3' ggGGAGACC-A--GUU-GGUCAGUGu 5'<br>||||||| | ||| |||||| | Target:5' aaCCTCTGGCTCCCAACCCAGTCTCc 3'	-23.00	142.00	
79	hsa-miR-135b*	2377~2407	31	miRNA: 3' ggguACCGAAAA--UC-------GGGAUGUa 5'<br>|||||||| :| ||||:|| Target:5' ttgtTGGCTTTTCCGGCTACGCACCCTGCAg 3'	-18.80	140.00	Show result profile
80	hsa-miR-136	565~588	24	miRNA: 3' agGUAGUAGUUUUGU-UUACCUCa 5'<br>:| :: |||||| |:||||| Target:5' ttTACTGCCAAAACAGAGTGGAGt 3'	-16.00	153.00	Show result profile
		2798~2824	27	miRNA: 3' gaUGGUAUCCCAUU------UUGGUGAc 5'<br>|::::| ||| ||	-14.70	149.00	

<table>
<tr><td>81</td><td>hsa-miR-140-5p</td><td></td><td></td><td>|||||||
Target:5' gaATTGT-GGGCAATGGATTAACCACTg 3'</td><td></td><td></td><td>Show result profile</td></tr>
<tr><td></td><td></td><td>372~398</td><td>27</td><td>miRNA: 3' gaUGGUAUCCCAUU-----UUGGUGac 5'
|||| | ||||
||||||
Target:5' ccACCAAAATGTAAATTCGAACCACca 3'</td><td>-10.40</td><td>140.00</td><td></td></tr>
<tr><td>82</td><td>hsa-miR-141</td><td>410~437</td><td>28</td><td>miRNA: 3' ggUAGAAAUGG------UCUGUCACAau 5'
|| | ||||
:||||||||
Target:5' gaATGTGTACCCTTCGGGGACAGTGTgc 3'</td><td>-12.50</td><td>142.00</td><td>Show result profile</td></tr>
<tr><td>83</td><td>hsa-miR-143*</td><td>1453~1476</td><td>24</td><td>miRNA: 3' ugguCUCUACG-UCG-UGACGUGg 5'
|||| | :|| |||||||
Target:5' catcGAGACCCTGGCAACTGCACc 3'</td><td>-20.30</td><td>154.00</td><td>Show result profile</td></tr>
<tr><td></td><td></td><td>980~1001</td><td>22</td><td>miRNA: 3' uggucucUACGUCGUGACGUGg 5'
:||||| :||||||
Target:5' tctcaccGTGCAGAGCTGCACt 3'</td><td>-20.00</td><td>147.00</td><td></td></tr>
<tr><td></td><td></td><td>1890~1915</td><td>26</td><td>miRNA: 3' ugGUCUCUACG---UC-GUGACGUGg 5'
::|:| ||| || |||||||
Target:5' ctTGGGGCTGCCTGAGCCACTGCAgg 3'</td><td>-13.90</td><td>140.00</td><td></td></tr>
<tr><td>84</td><td>hsa-miR-145*</td><td>485~507</td><td>23</td><td>miRNA: 3' ucUUGUCAUA--AAGGUCCUUAgg 5'
||||| || | ::||||||
Target:5' caAACAG-ATCCTATTAGGAATac 3'</td><td>-10.60</td><td>141.00</td><td>Show result profile</td></tr>
<tr><td></td><td></td><td>2272~2295</td><td>24</td><td>miRNA: 3' ucUUGUC-AUAA-AGGUCCUUAGg 5'
|:| | |:|| |::||||||
Target:5' ccAGCCGCTGTTGGCTGGGAATCg 3'</td><td>-13.40</td><td>140.00</td><td></td></tr>
<tr><td>85</td><td>hsa-miR-146b-3p</td><td>1341~1362</td><td>22</td><td>miRNA: 3' ggUCUUGACUCAGGUGUCCCGu 5'
:|| || || |||||||
Target:5' ctGGATCTTTGTAAACAGGGCt 3'</td><td>-19.30</td><td>156.00</td><td>Show result profile</td></tr>
<tr><td></td><td></td><td>2155~2183</td><td>29</td><td>miRNA: 3' ggUCUUGACUCAGG-------UGUCCCGu 5'
|| :| |:||||
:||||||
Target:5' caAGTGCAGGGTCCCAAGAGGGCAGGGCg 3'</td><td>-20.31</td><td>140.00</td><td></td></tr>
<tr><td>86</td><td>hsa-miR-</td><td>802~821</td><td>20</td><td>miRNA: 3' cgucuucGUAAAGGUGUGUg 5'</td><td>-15.00</td><td>145.00</td><td>Show result profile</td></tr>
</table>

<table>
<tr><td></td><td>147</td><td></td><td></td><td>|:||||||:|||
Target:5' aattgccCGTTTCCATACAg 3'</td><td></td><td></td><td></td></tr>
<tr><td>87</td><td>hsa-miR-1470</td><td>35~58</td><td>24</td><td>miRNA: 3' gccccaCGUGCCC---GCCUCCCg 5'
| :|||| |||||||
Target:5' cgcgcgGAGCGGGCTCCGGAGGGa 3'</td><td>-27.40</td><td>151.00</td><td>Show result profile</td></tr>
<tr><td>88</td><td>hsa-miR-148a*</td><td>663~688</td><td>26</td><td>miRNA: 3' ucagCCUCA-CAGA--G-UCUUGAAa 5'
||| | ||:| | |||||||
Target:5' gaagGGATTGGTTTGGCAAGAACTTg 3'</td><td>-11.40</td><td>152.00</td><td>Show result profile</td></tr>
<tr><td rowspan="4">89</td><td rowspan="4">hsa-miR-149*</td><td>1237~1257</td><td>21</td><td>miRNA: 3' cguGUCGGGGGCAGGGAGGGa 5'
|||: |||| |||||||
Target:5' tccCAGTGCCCGGCCCTCCCc 3'</td><td>-30.40</td><td>170.00</td><td rowspan="4">Show result profile</td></tr>
<tr><td>1702~1728</td><td>27</td><td>miRNA: 3' cgUGUCGGG----GGCA--GGGAGGga 5'
| ||||| |||| ||||||
Target:5' ggAGAGCCCACAGCCGTGGCCCTCCag 3'</td><td>-26.50</td><td>143.00</td></tr>
<tr><td>1022~1041</td><td>20</td><td>miRNA: 3' cguguCGGGGGCAGGGAGGGa 5'
|| ||| |||||||:
Target:5' gaaatGCACCC-TCCCTCCTg 3'</td><td>-19.80</td><td>143.00</td></tr>
<tr><td>2003~2023</td><td>21</td><td>miRNA: 3' cguguCGGGGGCAGGGAGGga 5'
||:||| ||||||
Target:5' ccggtGCTCCCAGCCCTCCaa 3'</td><td>-25.30</td><td>140.00</td></tr>
<tr><td>90</td><td>hsa-miR-151-5p</td><td>940~960</td><td>21</td><td>miRNA: 3' ugaucugaCACUCGAGGAGCu 5'
| |||:||||||
Target:5' ccccagggGCGAGTTCCTCGc 3'</td><td>-19.10</td><td>141.00</td><td>Show result profile</td></tr>
<tr><td>91</td><td>hsa-miR-152</td><td>1324~1344</td><td>21</td><td>miRNA: 3' ggUUCAAGACAGUACGUGACu 5'
|:| |||||| ||| |||
Target:5' tgAGGCTCTGTCTTGCCCTGg 3'</td><td>-17.20</td><td>143.00</td><td>Show result profile</td></tr>
<tr><td rowspan="2">92</td><td rowspan="2">hsa-miR-15a</td><td>1062~1084</td><td>23</td><td>miRNA: 3' gugUUUGGUAAU--ACACGACGAu 5'
:|||| | : |||||||||
Target:5' ctaGAACC-TGGGCTGTGCTGCTt 3'</td><td>-17.50</td><td>156.00</td><td rowspan="2">Show result profile</td></tr>
<tr><td>1354~1380</td><td>27</td><td>miRNA: 3' guGU-UUGGUA-A---UACACGACGAu 5'
|| ::|::| | | ||||||||
Target:5' aaCAGGGCTGTGTACAAAGTGCTGCTg 3'</td><td>-15.50</td><td>148.00</td></tr>
<tr><td>93</td><td>hsa-miR-</td><td>1902~1925</td><td>24</td><td>miRNA: 3' acUCCGU--CGUGUUAUACCGGAc 5'</td><td>-21.10</td><td>146.00</td><td>Show result profile</td></tr>
</table>

<table>
<tr><td></td><td>15a*</td><td></td><td></td><td>|| || ||| :| :||||||
Target:5' tgAGCCACTGCAGGAAGTGGCCTg
3'</td><td></td><td></td><td></td></tr>
<tr><td rowspan="2">94</td><td rowspan="2">hsa-miR-15b</td><td>1061~1084</td><td>24</td><td>miRNA: 3' acAUUUGGUACU--ACACGACGAu
5'
||:| | ||: |||||||||
Target:5' tcTAGAACCTGGGCTGTGCTGCTt
3'</td><td>-19.60</td><td>166.00</td><td rowspan="2">Show result profile</td></tr>
<tr><td>1350~1380</td><td>31</td><td>miRNA: 3' acAUUU-----GGUAC----
UACACGACGAu 5'
|||| |::|| |
||||||||
Target:5'
tgTAAACAGGGCTGTGTACAAAGTGCTGCTg 3'</td><td>-17.90</td><td>154.00</td></tr>
<tr><td rowspan="4">95</td><td rowspan="4">hsa-miR-16</td><td>1356~1380</td><td>25</td><td>miRNA: 3' gcgguuAUAAAUG---
CACGACGAu 5'
|:| ||| ||||||||
Target:5'
cagggcTGTGTACAAAGTGCTGCTg 3'</td><td>-14.60</td><td>156.00</td><td rowspan="4">Show result profile</td></tr>
<tr><td>1060~1084</td><td>25</td><td>miRNA: 3' gcGGUUAUAAAU---
GCACGACGAu 5'
|:|: | |: :||||||||
Target:5'
ttCTAGAACCTGGGCTGTGCTGCTt 3'</td><td>-14.60</td><td>148.00</td></tr>
<tr><td>1356~1380</td><td>25</td><td>miRNA: 3' gcgguuAUAAAUG---
CACGACGAu 5'
|:| ||| ||||||||
Target:5'
cagggcTGTGTACAAAGTGCTGCTg 3'</td><td>-14.60</td><td>156.00</td></tr>
<tr><td>1060~1084</td><td>25</td><td>miRNA: 3' gcGGUUAUAAAU---
GCACGACGAu 5'
|:|: | |: :||||||||
Target:5'
ttCTAGAACCTGGGCTGTGCTGCTt 3'</td><td>-14.60</td><td>148.00</td></tr>
<tr><td>96</td><td>hsa-miR-17*</td><td>1891~1915</td><td>25</td><td>miRNA: 3' gauguucACGGA---
AGUGACGUCa 5'
||||| ||||||||
Target:5'
ttggggcTGCCTGAGCCACTGCAGg 3'</td><td>-19.90</td><td>155.00</td><td>Show result profile</td></tr>
<tr><td rowspan="2">97</td><td rowspan="2">hsa-miR-181a-2*</td><td>2729~2753</td><td>25</td><td>miRNA: 3' ccaUGUCAGUUG---
CCAGUCACCa 5'
:|:|||| | | |:|||||
Target:5'
aacGCGGTCACCTTTGTTTAGTGGa 3'</td><td>-16.60</td><td>143.00</td><td rowspan="2">Show result profile</td></tr>
<tr><td>1564~1585</td><td>22</td><td>miRNA: 3' ccaugucaguugccAGUCACCa
5'
|||||||
Target:5' gggggttcccctctTCAGTGGc
3'</td><td>-14.60</td><td>140.00</td></tr>
<tr><td>98</td><td>hsa-miR-182*</td><td>1040~1060</td><td>21</td><td>miRNA: 3' aucaaccguucAGAUCUUGGu 5'
|||||||||
Target:5' tgccgctcctcTCTAGAACCt 3'</td><td>-11.40</td><td>150.00</td><td>Show result profile</td></tr>
</table>

<table>
<tr><td>99</td><td>hsa-miR-183</td><td>1464~1485</td><td>22</td><td>miRNA: 3' ucacUUAAGAUGGUCACGGUau 5'
               || | |||:||||||
Target:5' tggcAACTGCACCGGTGCCAgc 3'</td><td>-16.10</td><td>142.00</td><td>Show result profile</td></tr>
<tr><td rowspan="2">100</td><td rowspan="2">hsa-miR-184</td><td>1769~1794</td><td>26</td><td>miRNA: 3' ugGGA-AUAG---UCAAGAGGCAGGu 5'
            ||| |:|| ||||:|||||
Target:5' agCCTGTGTCTGGAATTCTTCGTCCt 3'</td><td>-20.50</td><td>152.00</td><td rowspan="2">Show result profile</td></tr>
<tr><td>1621~1642</td><td>22</td><td>miRNA: 3' ugGGAAUAGUCAAGAGGCAGGu 5'
            || |||| | ||:||||
Target:5' tgCCACCTCAGGTGTCTGTCCc 3'</td><td>-16.90</td><td>144.00</td></tr>
<tr><td rowspan="2">101</td><td rowspan="2">hsa-miR-185*</td><td>1987~2006</td><td>20</td><td>miRNA: 3' cuGGUCUCCUUUCGGUCGGGga 5'
            |::|:||| | ||||||
Target:5' ccCTGGGGGACA--CAGCCCgg 3'</td><td>-20.90</td><td>140.00</td><td rowspan="2">Show result profile</td></tr>
<tr><td>1253~1274</td><td>22</td><td>miRNA: 3' cuggucuccuuucgGUCGGGGa 5'
                        |||||||
Target:5' tccccacccacctgCAGCCCCa 3'</td><td>-13.64</td><td>140.00</td></tr>
<tr><td>102</td><td>hsa-miR-187*</td><td>2478~2501</td><td>24</td><td>miRNA: 3' cggGCCCAGG---ACACAACAUCGg 5'
             | |||:: | | |||||||
Target:5' cagCTGGTTTGAATAT-TTGTAGCc 3'</td><td>-15.20</td><td>146.00</td><td>Show result profile</td></tr>
<tr><td>103</td><td>hsa-miR-188-5p</td><td>649~671</td><td>23</td><td>miRNA: 3' ggGAGGUGG--UACGUUCCCUAc 5'
            | :|::| | | |||||||
Target:5' ggCATCGTCAAAAGGAAGGGATt 3'</td><td>-14.10</td><td>149.00</td><td>Show result profile</td></tr>
<tr><td>104</td><td>hsa-miR-188-3p</td><td>2244~2270</td><td>27</td><td>miRNA: 3' acguuUGGGACGU------ACACCCUc 5'
               ||||| || |||||||
Target:5' tcggcACCCTCCAGGTCCGTGTGGGAc 3'</td><td>-18.90</td><td>154.00</td><td>Show result profile</td></tr>
<tr><td>105</td><td>hsa-miR-18a*</td><td>2157~2180</td><td>24</td><td>miRNA: 3' gguCUUCCUCGUG-AAUCCCGUCa 5'
             | |||: | | |||||||
Target:5' agtGCAGGGTCCCAAGAGGGCAGg 3'</td><td>-17.20</td><td>148.00</td><td>Show result profile</td></tr>
<tr><td>106</td><td>hsa-miR-1908</td><td>81~100</td><td>20</td><td>miRNA: 3' cugguuaGCGGCAGGGGCGGc 5'
                 ||||| ||||||:
Target:5' ccgccgcCGCCG-CCCCGCTc 3'</td><td>-27.30</td><td>141.00</td><td>Show result profile</td></tr>
</table>

<table>
<tr><td>107</td><td>hsa-miR-1909</td><td>1021~1043</td><td>23</td><td>miRNA: 3' gccacuCGUGGG-CCGGGGACGc 5'
|||||| |:|||||
Target:5' ggaaatGCACCCTCCCTCCTGCc 3'</td><td>-20.80</td><td>140.00</td><td>Show result profile</td></tr>
<tr><td>108</td><td>hsa-miR-190b</td><td>140~164</td><td>25</td><td>miRNA: 3' uuGGGUUAU----AGUUUGUAUAGu 5'
|||:| : |::|||||:||
Target:5' cgCCCGAGGGACTTTGAACATGTCg 3'</td><td>-15.50</td><td>141.00</td><td>Show result profile</td></tr>
<tr><td>109</td><td>hsa-miR-191*</td><td>2143~2164</td><td>22</td><td>miRNA: 3' ccccuGCUUUAGGUUCGCGUCg 5'
|| :: |||||:||||
Target:5' gtccccCGTGGCCCAAGTGCAGg 3'</td><td>-18.90</td><td>145.00</td><td>Show result profile</td></tr>
<tr><td>110</td><td>hsa-miR-1910</td><td>448~467</td><td>20</td><td>miRNA: 3' ucCGCCGUCCGUGUCCUGACc 5'
| || :| || |||||||
Target:5' taGAGGAGGACA-AGGACTGg 3'</td><td>-21.80</td><td>154.00</td><td>Show result profile</td></tr>
<tr><td>111</td><td>hsa-miR-1911*</td><td>1492~1511</td><td>20</td><td>miRNA: 3' ccUCUGGUGUUACGGACCAc 5'
:|:|||||| ||| |||
Target:5' ggGGGCCACAAGGCCAGGTc 3'</td><td>-24.10</td><td>142.00</td><td>Show result profile</td></tr>
<tr><td rowspan="2">112</td><td rowspan="2">hsa-miR-1914</td><td>1599~1620</td><td>22</td><td>miRNA: 3' guCUUCACCCGGCCCGUGUCCc 5'
||: || ||:| |||:|||
Target:5' tgGAGCTGCGCTGTGCATAGGg 3'</td><td>-23.40</td><td>152.00</td><td rowspan="2">Show result profile</td></tr>
<tr><td>1576~1597</td><td>22</td><td>miRNA: 3' gucuUCACCCGGCCCGUGUCCc 5'
||||| | || ||||||
Target:5' cttcAGTGGCCAGGTCACAGGg 3'</td><td>-25.80</td><td>142.00</td></tr>
<tr><td>113</td><td>hsa-miR-1914*</td><td>2327~2353</td><td>27</td><td>miRNA: 3' ggaggGUCAC-----GCCCUGGGGAGg 5'
||||| | |||||||
Target:5' gccggCAGTGCCCCCCACCACCCCTCc 3'</td><td>-24.60</td><td>145.00</td><td>Show result profile</td></tr>
<tr><td rowspan="3"></td><td rowspan="3">hsa-</td><td>1745~1768</td><td>24</td><td>miRNA: 3' ggGCGGCGCA----GCGGGACCCc 5'
|||| || ||||||||
Target:5' agCGCCAGGTAGAGGGCCCTGGGc 3'</td><td>-23.10</td><td>152.00</td><td rowspan="3"></td></tr>
<tr><td>2213~2235</td><td>23</td><td>miRNA: 3' gggCGGCGCAG----CGGGACCCc 5'
| || ||| ||||||||
Target:5' gagGGCG-GTCCAGGGCCCTGGGa 3'</td><td>-21.20</td><td>150.00</td></tr>
<tr><td>1975~1994</td><td>20</td><td>miRNA: 3' gggCGGCGCAGCGGGACCCc 5'
| :| | ||||||||
Target:5' agaGATGGGAAGCCCTGGGg 3'</td><td>-23.60</td><td>149.00</td></tr>
</table>

<table>
<tr><td rowspan="4">114</td><td rowspan="4">miR-1915</td><td></td><td></td><td></td><td></td><td></td><td rowspan="4">Show result profile</td></tr>
<tr><td>1327~1346</td><td>20</td><td>miRNA: 3' ggGCGGCGCAGCGGGACCcc 5'
| |:|: |:|||||||
Target:5' ggCTCTGTCTTGCCCTGGat 3'</td><td>-19.90</td><td>142.00</td></tr>
<tr><td>2102~2121</td><td>20</td><td>miRNA: 3' gggCGGCGCAGCGGGACCcc 5'
||| :| :|||||||
Target:5' ctgGCCCTGCTGCCCTGGca 3'</td><td>-22.20</td><td>141.00</td></tr>
<tr><td>948~967</td><td>20</td><td>miRNA: 3' gggcggcGCAGCGGGACCCc 5'
| ||||:|||||
Target:5' gcgagttCCTCGCTCTGGGa 3'</td><td>-19.70</td><td>141.00</td></tr>
<tr><td>115</td><td>hsa-miR-192*</td><td>1427~1455</td><td>29</td><td>miRNA: 3' gacaCUGGA--UA-----
CCUUAACCGUc 5'
||:|| ||
||:|||||||
Target:5'
ctaaGATCTGCATAGGTCGGGATTGGCAt 3'</td><td>-16.40</td><td>156.00</td><td>Show result profile</td></tr>
<tr><td>116</td><td>hsa-miR-193a-5p</td><td>521~542</td><td>22</td><td>miRNA: 3' aguagagcgggcGUUUCUGGGu
5'
| |||||||
Target:5' tgaaccaaatatCCAAGACCCa
3'</td><td>-13.30</td><td>142.00</td><td>Show result profile</td></tr>
<tr><td>117</td><td>hsa-miR-193a-3p</td><td>457~476</td><td>20</td><td>miRNA: 3' ugacCCUGAAACAUCCGGUCaa
5'
|||| || |||||||
Target:5' acaaGGAC--TGGAGGCCAGcc
3'</td><td>-20.00</td><td>142.00</td><td>Show result profile</td></tr>
<tr><td>118</td><td>hsa-miR-193b*</td><td>1080~1101</td><td>22</td><td>miRNA: 3' aguAGAGC-GGGAGUUUUGGGGc
5'
|:|:| |||| |:|||||
Target:5' tgcTTTTGAGCCTC-AGACCCCa
3'</td><td>-19.50</td><td>142.00</td><td>Show result profile</td></tr>
<tr><td rowspan="3">119</td><td rowspan="3">hsa-miR-195</td><td>1064~1084</td><td>21</td><td>miRNA: 3' cgguuauaaaGACACGACGAu 5'
||||||||||
Target:5' agaacctgggCTGTGCTGCTt 3'</td><td>-18.40</td><td>155.00</td><td rowspan="3">Show result profile</td></tr>
<tr><td>1144~1164</td><td>21</td><td>miRNA: 3' cgGUUAUAAAGACACGACGAu 5'
|: | | ||||||:||||
Target:5' ggCGTTTTGTCTGTGTTGCTg 3'</td><td>-18.60</td><td>151.00</td></tr>
<tr><td>1360~1380</td><td>21</td><td>miRNA: 3' cgguuauaaagaCACGACGAu 5'
||||||||
Target:5' gctgtgtacaaaGTGCTGCTg 3'</td><td>-13.50</td><td>145.00</td></tr>
<tr><td>120</td><td>hsa-miR-196a*</td><td>1140~1164</td><td>25</td><td>miRNA: 3' gaguCCGUCAA--AGA-
ACAACGGc 5'
|||: || ||| ||||||:
Target:5'
atatGGCGTTTTGTCTGTGTTGCTg 3'</td><td>-16.80</td><td>144.00</td><td>Show result profile</td></tr>
</table>

<table>
<tr><td>121</td><td>hsa-miR-196b*</td><td>219~241</td><td>23</td><td>miRNA: 3' cuuccGUCACAGCA-CGACAGcu 5'
::|| |||| ||||||
Target:5' ccattTGGTTTCGTGGCTGTCcc 3'</td><td>-13.50</td><td>141.00</td><td>Show result profile</td></tr>
<tr><td rowspan="4">122</td><td rowspan="4">hsa-miR-1972</td><td>1902~1926</td><td>25</td><td>miRNA: 3' acUCGGUGAC--AC-GGACCGGACu 5'
|||||||| | |||||||
Target:5' tgAGCCACTGCAGGAAGTGGCCTGg 3'</td><td>-29.60</td><td>158.00</td><td rowspan="4">Show result profile</td></tr>
<tr><td>1795~1817</td><td>23</td><td>miRNA: 3' acUCGGU-GACACGGACCGGACu 5'
:|||| ||| |: |||:|||
Target:5' gaGGCCACCTGAGTGTGGTCTGt 3'</td><td>-23.30</td><td>152.00</td></tr>
<tr><td>1902~1926</td><td>25</td><td>miRNA: 3' acUCGGUGAC--AC-GGACCGGACu 5'
|||||||| | |||||||
Target:5' tgAGCCACTGCAGGAAGTGGCCTGg 3'</td><td>-29.60</td><td>158.00</td></tr>
<tr><td>1795~1817</td><td>23</td><td>miRNA: 3' acUCGGU-GACACGGACCGGACu 5'
:|||| ||| |: |||:|||
Target:5' gaGGCCACCTGAGTGTGGTCTGt 3'</td><td>-23.30</td><td>152.00</td></tr>
<tr><td rowspan="2">123</td><td rowspan="2">hsa-miR-19a*</td><td>846~867</td><td>22</td><td>miRNA: 3' acaucacguUGAUACGUUUUGa 5'
::||||:|||||
Target:5' tttttgattGTTATGTAAAACt 3'</td><td>-10.80</td><td>141.00</td><td rowspan="2">Show result profile</td></tr>
<tr><td>892~914</td><td>23</td><td>miRNA: 3' acAUCACGUUGAU-ACGUUUUGa 5'
|| | |||||: ||:||||:
Target:5' agTATTTCAACTGCTGTAAAATt 3'</td><td>-12.60</td><td>140.00</td></tr>
<tr><td>124</td><td>hsa-miR-19a</td><td>2834~2857</td><td>24</td><td>miRNA: 3' agucaaaacGUAUC-UAAACGUGu 5'
:| || ||||||||
Target:5' atccaccatTAAAGCATTTGCACa 3'</td><td>-10.30</td><td>150.00</td><td>Show result profile</td></tr>
<tr><td rowspan="2">125</td><td rowspan="2">hsa-miR-19b</td><td>2835~2857</td><td>23</td><td>miRNA: 3' agucaaaacgUACCUAAACGUGu 5'
| | ||||||||
Target:5' tccaccattaAAGCATTTGCACa 3'</td><td>-9.70</td><td>149.00</td><td rowspan="2">Show result profile</td></tr>
<tr><td>2835~2857</td><td>23</td><td>miRNA: 3' agucaaaacgUACCUAAACGUGu 5'
| | ||||||||
Target:5' tccaccattaAAGCATTTGCACa 3'</td><td>-9.70</td><td>149.00</td></tr>
</table>

<table>
<tr><td>126</td><td>hsa-miR-200b</td><td>875~898</td><td>24</td><td>miRNA: 3' aguAGUAAUGGU--CCGUCAUAAu 5'
|:| ||::: | |||||||
Target:5' attTTAATATTGATGTCAGTATTt 3'</td><td>-8.60</td><td>153.00</td><td>Show result profile</td></tr>
<tr><td>127</td><td>hsa-miR-200c</td><td>874~898</td><td>25</td><td>miRNA: 3' agguAGUAAUGG--GCCGUCAUAAu 5'
|:| ||:: :| |||||||
Target:5' tattTTAATATTGATGTCAGTATTt 3'</td><td>-10.40</td><td>153.00</td><td>Show result profile</td></tr>
<tr><td rowspan="2">128</td><td rowspan="2">hsa-miR-202*</td><td>1424~1444</td><td>21</td><td>miRNA: 3' guuucUUCAUAUACGUAUCCuu 5'
||| || ||||||||
Target:5' cagctAAG-ATCTGCATAGGtc 3'</td><td>-12.50</td><td>144.00</td><td rowspan="2">Show result profile</td></tr>
<tr><td>1596~1621</td><td>26</td><td>miRNA: 3' guUUCUUC-AU---AUACGUAUCCUu 5'
| |:|| |: |:||||||||:
Target:5' ggATGGAGCTGCGCTGTGCATAGGGt 3'</td><td>-15.10</td><td>144.00</td></tr>
<tr><td rowspan="2">129</td><td rowspan="2">hsa-miR-204</td><td>57~77</td><td>21</td><td>miRNA: 3' uccguaucCUACUGUUUCCCUu 5'
|| ||||||||||
Target:5' gaagtcccGA-GACAAAGGGAa 3'</td><td>-16.60</td><td>157.00</td><td rowspan="2">Show result profile</td></tr>
<tr><td>287~310</td><td>24</td><td>miRNA: 3' uccGUA---UCCUACUGUUUCCCUu 5'
||| |||| | |||||||
Target:5' cgcCATTCCAGGA-AAGAAAGGGAc 3'</td><td>-11.50</td><td>154.00</td></tr>
<tr><td>130</td><td>hsa-miR-20a*</td><td>2191~2212</td><td>22</td><td>miRNA: 3' gaaaUUCACGAGUAUUACGUCa 5'
|:| || | |||||||
Target:5' ccaaAGGAGCAAAGAATGCAGg 3'</td><td>-9.90</td><td>154.00</td><td>Show result profile</td></tr>
<tr><td rowspan="2">131</td><td rowspan="2">hsa-miR-20b*</td><td>319~342</td><td>24</td><td>miRNA: 3' gaCCUUC--ACGGGUAUGAUGUCa 5'
|||:| ||:::| |||||:|
Target:5' aaGGAGGCTTGTTTAAACTACGGa 3'</td><td>-17.60</td><td>150.00</td><td rowspan="2">Show result profile</td></tr>
<tr><td>1891~1915</td><td>25</td><td>miRNA: 3' gaCCUUCACGGGU---AUGAUGUCa 5'
||:: ||||:: |||:|||
Target:5' ttGGGGCTGCCTGAGCCACTGCAGg 3'</td><td>-18.60</td><td>140.00</td></tr>
<tr><td></td><td></td><td>53~77</td><td>25</td><td>miRNA: 3' ucCGCUUC----CUACUGUUUCCCUu 5'
| |||| || |||||||||
Target:5' gaGGGAAGTCCCGA-GACAAAGGGAa 3'</td><td>-23.00</td><td>165.00</td><td></td></tr>
</table>

<table>
<tr><td rowspan="3">132</td><td rowspan="3">hsa-miR-211</td><td>290~310</td><td>21</td><td>miRNA: 3' uccgcuUCCUACUGUUUCCCUu 5'
|||| | |||||||
Target:5' cattccAGGA-AAGAAAGGGAc 3'</td><td>-11.30</td><td>151.00</td><td rowspan="3">Show result profile</td></tr>
<tr><td>580~602</td><td>23</td><td>miRNA: 3' ucCGCUUCCUACU-GUUUCCCuu 5'
|:|:|| | || ||||||
Target:5' gaGTGGAGTACGAGAAAAGGGtc 3'</td><td>-14.40</td><td>140.00</td></tr>
<tr><td>2215~2242</td><td>28</td><td>miRNA: 3' ucCGCU---UCCU---ACUGUUUCCCUu 5'
|||: |||: ||: ||:||||
Target:5' ggGCGGTCCAGGGCCCTGGGAAGGGGAg 3'</td><td>-24.80</td><td>140.00</td></tr>
<tr><td rowspan="2">133</td><td rowspan="2">hsa-miR-2113</td><td>1352~1372</td><td>21</td><td>miRNA: 3' cacUGUCUCGGUUCGUGUUUa 5'
||||:||:: |:|||||
Target:5' taaACAGGGCTGTGTACAAAg 3'</td><td>-17.20</td><td>154.00</td><td rowspan="2">Show result profile</td></tr>
<tr><td>592~613</td><td>22</td><td>miRNA: 3' cacUGUCUC-GGUUCGUGUUUa 5'
| ||:| ||:|||||||:
Target:5' agaAAAGGGTCCGAGCACAAGc 3'</td><td>-18.90</td><td>150.00</td></tr>
<tr><td rowspan="3">134</td><td rowspan="3">hsa-miR-2114</td><td>284~312</td><td>29</td><td>miRNA: 3' cugGCG--AAG-----UUCCUUCCCUGAu 5'
||| ||| ||| ||||||||
Target:5' gtgCGCCATTCCAGGAAAGAAAGGGACTc 3'</td><td>-18.40</td><td>161.00</td><td rowspan="3">Show result profile</td></tr>
<tr><td>651~672</td><td>22</td><td>miRNA: 3' cuGGCGAAGUUCCUUCCCUGAu 5'
:||: ||||||||||:|
Target:5' caTCGTCAAAAGGAAGGGATTg 3'</td><td>-24.50</td><td>152.00</td></tr>
<tr><td>128~153</td><td>26</td><td>miRNA: 3' cuggCGAAGUU--C--CUUCCCUGAu 5'
||| |:: | :|||||||
Target:5' cgggGCTGCGGCCGCCCGAGGGACTt 3'</td><td>-20.00</td><td>142.00</td></tr>
<tr><td>135</td><td>hsa-miR-2114*</td><td>1311~1332</td><td>22</td><td>miRNA: 3' uucaGGGAACGA-ACUCCGAGc 5'
| | ||| ||||||||
Target:5' tgcaCGCAGGCTGTGAGGCTCt 3'</td><td>-17.00</td><td>153.00</td><td>Show result profile</td></tr>
<tr><td>136</td><td>hsa-miR-2116</td><td>2816~2836</td><td>21</td><td>miRNA: 3' ucUGGAGG-AUACGAUUCUUGg 5'
||| |: ||| |||||||:
Target:5' taACCACTGTAT-CTAAGAATc 3'</td><td>-14.60</td><td>146.00</td><td>Show result profile</td></tr>
<tr><td></td><td></td><td></td><td></td><td>miRNA: 3' ugacGGACAGAC---</td><td></td><td></td><td></td></tr>
</table>

<table>
<tr><td rowspan="2">137</td><td rowspan="2">hsa-miR-214</td><td>1538~1562</td><td>25</td><td>ACGGACGACa 5'
||| || | ||||||||
Target:5' gactCCTATCCGGGCAGCCTGCTGg 3'</td><td>-19.30</td><td>154.00</td><td rowspan="2">Show result profile</td></tr>
<tr><td>2091~2114</td><td>24</td><td>miRNA: 3' ugaCGGAC--AGACACGGACGACa 5'
| ||| ||| |||||||
Target:5' tggGGCTGGAGCTGGCCCTGCTGc 3'</td><td>-20.40</td><td>153.00</td></tr>
<tr><td>138</td><td>hsa-miR-216a</td><td>1367~1386</td><td>20</td><td>miRNA: 3' agUGUCAACGGUCGACUCUAAu 5'
| || |||: ||||||:||
Target:5' acAAAG-TGCT-GCTGAGGTTt 3'</td><td>-18.10</td><td>146.00</td><td>Show result profile</td></tr>
<tr><td>139</td><td>hsa-miR-219-1-3p</td><td>777~798</td><td>22</td><td>miRNA: 3' gcCCUGCAGGUCUGAGUUGAGa 5'
||::|| |:| |||:|:||
Target:5' gcGGGTGTGCGGTCTCGATTCg 3'</td><td>-25.40</td><td>140.00</td><td>Show result profile</td></tr>
<tr><td>140</td><td>hsa-miR-22</td><td>1407~1429</td><td>23</td><td>miRNA: 3' ugucaagaaguUGA-CCGUCGAa 5'
:|| |||||||
Target:5' ggctgtagagcGCTGGGCAGCTa 3'</td><td>-12.50</td><td>143.00</td><td>Show result profile</td></tr>
<tr><td>141</td><td>hsa-miR-222*</td><td>1360~1382</td><td>23</td><td>miRNA: 3' uccUAGAUGUGAC-CGAUGACUc 5'
:| |||| | |||:||||
Target:5' gctGTGTACAAAGTGCTGCTGAg 3'</td><td>-17.80</td><td>143.00</td><td>Show result profile</td></tr>
<tr><td>142</td><td>hsa-miR-223*</td><td>1004~1031</td><td>28</td><td>miRNA: 3' uuGAGUCGA---ACA---GUUUAUGUGc 5'
||||||| |||
||||:|||
Target:5' gcCTCAGCTGGCTGTATGGAAATGCACc 3'</td><td>-15.70</td><td>152.00</td><td>Show result profile</td></tr>
<tr><td rowspan="2">143</td><td rowspan="2">hsa-miR-224</td><td>2630~2649</td><td>20</td><td>miRNA: 3' uugccUUGGUGAUCACUGAAc 5'
||:|: |||||||||
Target:5' tcgccAATCG-TAGTGACTTc 3'</td><td>-14.10</td><td>159.00</td><td rowspan="2">Show result profile</td></tr>
<tr><td>2292~2312</td><td>21</td><td>miRNA: 3' uuGCCUUGGUGAUCACUGAAc 5'
|| |:::| :|||||||
Target:5' atCGAAGTTAGAGGTGACTTc 3'</td><td>-12.80</td><td>155.00</td></tr>
<tr><td>144</td><td>hsa-miR-2277-5p</td><td>1~9</td><td>9</td><td>miRNA: 3' cugaccgucgcgaguCGGGCGCGa 5'
||||||||
Target:5' ---------------GCCCGCGCc 3'</td><td>-15.80</td><td>145.00</td><td>Show result profile</td></tr>
<tr><td>145</td><td>hsa-miR-2278</td><td>8~30</td><td>23</td><td>miRNA: 3' ggUCCGUUGUGUGU-GACGAGAg 5'
||| | |: | |||||||
Target:5' ccAGGGTCCTCGGAGCTGCTCTg 3'</td><td>-18.80</td><td>148.00</td><td>Show result profile</td></tr>
</table>

<table>
<tr><td rowspan="2">146</td><td rowspan="2">hsa-miR-2355-5p</td><td>1805~1826</td><td>22</td><td>miRNA: 3' aaCAGGUAACAUA-GACCCCUa 5'
|| :: | |:| |||||||
Target:5' gaGTGTGGTCTGTCCTGGGGAg 3'</td><td>-14.30</td><td>151.00</td><td rowspan="2">Show result profile</td></tr>
<tr><td>2455~2475</td><td>21</td><td>miRNA: 3' aacagguaacAUAGACCCCUa 5'
|| |||||||
Target:5' cacggggcctTACCTGGGGAa 3'</td><td>-12.60</td><td>147.00</td></tr>
<tr><td>147</td><td>hsa-miR-2355-3p</td><td>438~461</td><td>24</td><td>miRNA: 3' uagAGGU---UUGUCGUUCCUGUUa 5'
|||| | || :|||||||
Target:5' ctgTCCATCTTAGAG-GAGGACAAg 3'</td><td>-15.90</td><td>150.00</td><td>Show result profile</td></tr>
<tr><td rowspan="6">148</td><td rowspan="6">hsa-miR-24</td><td>1840~1862</td><td>23</td><td>miRNA: 3' gacaaGGACGACU-UGACUCGGu 5'
| | |||| :|||||||
Target:5' gcagcCGTCCTGACGCTGAGCCc 3'</td><td>-19.00</td><td>157.00</td><td rowspan="6">Show result profile</td></tr>
<tr><td>1885~1908</td><td>24</td><td>miRNA: 3' gacaAGGA---CGACUUGACUCGGu 5'
||:| |||| |||||||
Target:5' ggccTCTTGGGGCTG-CCTGAGCCa 3'</td><td>-19.30</td><td>153.00</td></tr>
<tr><td>2396~2418</td><td>23</td><td>miRNA: 3' gacaaGGACGAC-UUGACUCGGu 5'
||||| | |||||||:
Target:5' cgcacCCTGCAGTCACTGAGCTc 3'</td><td>-18.10</td><td>145.00</td></tr>
<tr><td>1840~1862</td><td>23</td><td>miRNA: 3' gacaaGGACGACU-UGACUCGGu 5'
| | |||| :|||||||
Target:5' gcagcCGTCCTGACGCTGAGCCc 3'</td><td>-19.00</td><td>157.00</td></tr>
<tr><td>1885~1908</td><td>24</td><td>miRNA: 3' gacaAGGA---CGACUUGACUCGGu 5'
||:| |||| |||||||
Target:5' ggccTCTTGGGGCTG-CCTGAGCCa 3'</td><td>-19.30</td><td>153.00</td></tr>
<tr><td>2396~2418</td><td>23</td><td>miRNA: 3' gacaaGGACGAC-UUGACUCGGu 5'
||||| | |||||||:
Target:5' cgcacCCTGCAGTCACTGAGCTc 3'</td><td>-18.10</td><td>145.00</td></tr>
<tr><td>149</td><td>hsa-miR-25*</td><td>356~374</td><td>19</td><td>miRNA: 3' guUAACGGGUUCAGAGGCGGa 5'
||| :||| |||:||||
Target:5' tgATTATCCA--TCTTCGCCa 3'</td><td>-16.70</td><td>147.00</td><td>Show result profile</td></tr>
<tr><td></td><td></td><td>920~941</td><td>22</td><td>miRNA: 3' acGAGUGUUCGUCGAUUCGGGa 5'
:|:|:| ::| ||||:||</td><td>-11.00</td><td>140.00</td><td></td></tr>
</table>

<table>
<tr><td rowspan="2">150</td><td rowspan="2">hsa-miR-27a*</td><td></td><td></td><td>Target:5' ctTTTATACTTGGGTAAGTCCc
3'</td><td></td><td></td><td rowspan="2">Show result profile</td></tr>
<tr><td>1834~1863</td><td>30</td><td>miRNA: 3' acGAGUGUUCG-------U-
CGAUUCGGGa 5'
          |||| ||| |
|||:|||||
Target:5'
gcCTCAGCAGCCGTCCTGACGCTGAGCCCt 3'</td><td>-21.50</td><td>140.00</td></tr>
<tr><td rowspan="2">151</td><td rowspan="2">hsa-miR-27b*</td><td>2395~2419</td><td>25</td><td>miRNA: 3' caaGUG----
GUUAGUCGAUUCGAGa 5'
           ||| ||:|||
||:|||||
Target:5' acgCACCCTGCAGTCA-
CTGAGCTCt 3'</td><td>-20.70</td><td>148.00</td><td rowspan="2">Show result profile</td></tr>
<tr><td>105~126</td><td>22</td><td>miRNA: 3' caaGUGGUUAGUCGAUUCGAGa
5'
           |||| :|| |||| ||||
Target:5' ctcCACCTGTCCGCTACGCTCg
3'</td><td>-16.80</td><td>143.00</td></tr>
<tr><td rowspan="2">152</td><td rowspan="2">hsa-miR-2861</td><td>1494~1513</td><td>20</td><td>miRNA: 3' ggCGG-GUGGCGGUCCGGGg 5'
            ||| || ||||||:||
Target:5' ggGCCACAAGGCCAGGTCCa 3'</td><td>-25.30</td><td>145.00</td><td rowspan="2">Show result profile</td></tr>
<tr><td>2245~2262</td><td>18</td><td>miRNA: 3' ggCGGGUGGCGGUCCGGGg 5'
            || | || |||||:||
Target:5' cgGCAC-CCTCCAGGTCCg 3'</td><td>-20.70</td><td>140.00</td></tr>
<tr><td rowspan="2">153</td><td rowspan="2">hsa-miR-296-3p</td><td>1725~1744</td><td>20</td><td>miRNA: 3' ccUCUCGGAGGUGGGUUGGGAg
5'
            ||:||:|| |||:|||||
Target:5' ccAGGGCTTC--CCCGACCCTt
3'</td><td>-26.40</td><td>156.00</td><td rowspan="2">Show result profile</td></tr>
<tr><td>1996~2020</td><td>25</td><td>miRNA: 3' ccUCUCGG---
AGGUGGGUUGGGAg 5'
            | |||| | | ||||:||||
Target:5'
acACAGCCCGGTGCTCCCAGCCCTc 3'</td><td>-25.30</td><td>148.00</td></tr>
<tr><td>154</td><td>hsa-miR-299-3p</td><td>1378~1402</td><td>25</td><td>miRNA: 3' uuCGCCAAAUGGUA----
GGGUGUAu 5'
            | ||||| |::|
|||:|||
Target:5' ctGAGGTTT-
CTGTGCTCCCCGCATc 3'</td><td>-12.80</td><td>141.00</td><td>Show result profile</td></tr>
<tr><td>155</td><td>hsa-miR-300</td><td>1628~1649</td><td>22</td><td>miRNA: 3' ucUCUCUCAGAC-GGGAACAUAu
5'
            || | ||||| |||||||:|
Target:5' tcAG-GTGTCTGTCCCTTGTGTc
3'</td><td>-20.40</td><td>155.00</td><td>Show result profile</td></tr>
<tr><td>156</td><td>hsa-miR-302a</td><td>2825~2853</td><td>29</td><td>miRNA: 3' aguGGUUUU------
GUACCUUCGUGAAu 5'
             |:||:| |||
|||||:||
Target:5'
tatCTAAGAATCCACCATTAAAGCATTTg 3'</td><td>-12.20</td><td>142.00</td><td>Show result profile</td></tr>
</table>

<table>
<tr><td></td><td></td><td></td><td></td><td></td><td></td><td></td><td></td></tr>
<tr><td>157</td><td>hsa-miR-302b</td><td>2825~2853</td><td>29</td><td>miRNA: 3' gauGAUUUU------GUACCUUCGUGAAu 5'
||||:| ||| |||||:||
Target:5' tatCTAAGAATCCACCATTAAAGCATTTg 3'</td><td>-10.70</td><td>146.00</td><td>Show result profile</td></tr>
<tr><td>158</td><td>hsa-miR-302c</td><td>2831~2853</td><td>23</td><td>miRNA: 3' ggUGACUUUGUACCUUCGUGAAu 5'
| | | ||| |||||:||
Target:5' agAATCCACCATTAAAGCATTTg 3'</td><td>-12.90</td><td>141.00</td><td>Show result profile</td></tr>
<tr><td>159</td><td>hsa-miR-302d</td><td>2831~2853</td><td>23</td><td>miRNA: 3' ugUGAGUUUGUACCUUCGUGAAu 5'
| || | ||| |||||:||
Target:5' agAATCCACCATTAAAGCATTTg 3'</td><td>-9.20</td><td>149.00</td><td>Show result profile</td></tr>
<tr><td>160</td><td>hsa-miR-30a</td><td>672~694</td><td>23</td><td>miRNA: 3' gaAGGUCAGCUCCU--ACAAAUGu 5'
|:::| | || | |||||||
Target:5' ggTTTGG-CAAGAACTTGTTTACa 3'</td><td>-7.70</td><td>149.00</td><td>Show result profile</td></tr>
<tr><td>161</td><td>hsa-miR-30a*</td><td>1200~1221</td><td>22</td><td>miRNA: 3' cgACGU--UUGUAGGCUGACUUUc 5'
|||| | :||| |:|||||
Target:5' gtTGCATTATTATC--ATTGAAAg 3'</td><td>-14.50</td><td>142.00</td><td>Show result profile</td></tr>
<tr><td rowspan="2">162</td><td rowspan="2">hsa-miR-30b</td><td>667~694</td><td>28</td><td>miRNA: 3' ucgACUCACAUC---CU---ACAAAUGu 5'
| :|| |:| || |||||||
Target:5' ggaTTGGTTTGGCAAGAACTTGTTTACa 3'</td><td>-8.09</td><td>147.00</td><td rowspan="2">Show result profile</td></tr>
<tr><td>2429~2453</td><td>25</td><td>miRNA: 3' ucGACU-C--ACAUCCUACAAAUGu 5'
|||| | |||:| ||||||:
Target:5' gtCTGATGTTTGTGGTTTGTTTATa 3'</td><td>-13.90</td><td>146.00</td></tr>
<tr><td rowspan="2">163</td><td rowspan="2">hsa-miR-30b*</td><td>1936~1956</td><td>21</td><td>miRNA: 3' cuUCAUUUGUAGGUGGAGGGUc 5'
:|| : |: :|||||||||
Target:5' tgGGT-GCCGGTCACCTCCCAg 3'</td><td>-24.60</td><td>155.00</td><td rowspan="2">Show result profile</td></tr>
<tr><td>1224~1242</td><td>19</td><td>miRNA: 3' cuUCAUUUGUAGGUGGAGGGUc 5'
|| |::|: :|||||||
Target:5' agAGGAGGCG---GCCTCCCAg 3'</td><td>-21.90</td><td>149.00</td></tr>
<tr><td></td><td></td><td>663~694</td><td>32</td><td>miRNA: 3' cgaCUCU---CACAUC---CU---ACAAAUGu 5'
|:|| || |:| || |||||||
Target:5' gaaGGGATTGGTTTGGCAAGAACTTGTTTACa 3'</td><td>-10.80</td><td>148.00</td><td></td></tr>
</table>

<table>
<tr><td rowspan="2">164</td><td rowspan="2">hsa-miR-30c</td><td></td><td></td><td></td><td></td><td></td><td rowspan="2">Show result profile</td></tr>
<tr><td>2430~2453</td><td>24</td><td>miRNA: 3' cgACU-CUCACAUCCUACAAAUGu 5'
||| | |||:| ||||||:
Target:5' tcTGATGTTTGTGGTTTGTTTATa 3'</td><td>-16.00</td><td>145.00</td></tr>
<tr><td>165</td><td>hsa-miR-30c-1*</td><td>2027~2050</td><td>24</td><td>miRNA: 3' ccucauuuGUUGGG--AGAGGGuc 5'
|||||| ||||||
Target:5' ctggctccCAACCCAGTCTCCCca 3'</td><td>-16.90</td><td>140.00</td><td>Show result profile</td></tr>
<tr><td rowspan="2">166</td><td rowspan="2">hsa-miR-30c</td><td>663~694</td><td>32</td><td>miRNA: 3' cgaCUCU---CACAUC---CU---ACAAAUGu 5'
|:|| || |:| || |||||||
Target:5' gaaGGGATTGGTTTGGCAAGAACTTGTTTACa 3'</td><td>-10.80</td><td>148.00</td><td rowspan="2">Show result profile</td></tr>
<tr><td>2430~2453</td><td>24</td><td>miRNA: 3' cgACU-CUCACAUCCUACAAAUGu 5'
||| | |||:| ||||||:
Target:5' tcTGATGTTTGTGGTTTGTTTATa 3'</td><td>-16.00</td><td>145.00</td></tr>
<tr><td>167</td><td>hsa-miR-30d</td><td>667~694</td><td>28</td><td>miRNA: 3' gaaGGUCAGCCC---CU---ACAAAUGu 5'
:::||: || || |||||||
Target:5' ggaTTGGTTTGGCAAGAACTTGTTTACa 3'</td><td>-8.80</td><td>147.00</td><td>Show result profile</td></tr>
<tr><td>168</td><td>hsa-miR-30e</td><td>672~694</td><td>23</td><td>miRNA: 3' gaAGGUCAGUUCCU--ACAAAUGu 5'
|:::| |||| | |||||||
Target:5' ggTTTGG-CAAGAACTTGTTTACa 3'</td><td>-10.70</td><td>157.00</td><td>Show result profile</td></tr>
<tr><td>169</td><td>hsa-miR-31</td><td>1319~1341</td><td>23</td><td>miRNA: 3' ucGAUACGGUCG---UAGAACGGa 5'
||:|| :|| :|||||||
Target:5' ggCTGTG-AGGCTCTGTCTTGCCc 3'</td><td>-19.20</td><td>150.00</td><td>Show result profile</td></tr>
<tr><td rowspan="3"></td><td rowspan="3"></td><td>2390~2412</td><td>23</td><td>miRNA: 3' ucuuaaaaGUAUUACGUCAGUGu 5'
|| |||||||||
Target:5' cggctacgCACCCTGCAGTCACt 3'</td><td>-13.50</td><td>151.00</td><td rowspan="3"></td></tr>
<tr><td>711~739</td><td>29</td><td>miRNA: 3' ucUUAA--AAGUA--UUAC--GUCAGUGu 5'
|:|| | ||| |||| :||||||
Target:5' aaAGTTGCTCCATACAATGACTAGTCACc 3'</td><td>-8.90</td><td>147.00</td></tr>
<tr><td>2719~2740</td><td>22</td><td>miRNA: 3' ucUUAAAAGUAUUACGUCAGUGu 5'
|: | | | || ||:|||||</td><td>-10.41</td><td>140.00</td></tr>
</table>

				Target:5' agAGCTCTGA-AACGCGGTCACc<br>3'			
		2390~2412	23	miRNA: 3' ucuuaaaaGUAUUACGUCAGUGu<br>5'<br>|| ||||||||| Target:5' cggctacgCACCCTGCAGTCACt<br>3'	-13.50	151.00	
		711~739	29	miRNA: 3' ucUUAA--AAGUA--UUAC--<br>GUCAGUGu 5'<br>|:|| | ||| |||| :|||||| Target:5'<br>aaAGTTGCTCCATACAATGACTAGTCACc 3'	-8.90	147.00	
		2719~2740	22	miRNA: 3' ucUUAAAAGUAUUACGUCAGUGu<br>5'<br>|: | | | || ||:||||| Target:5' agAGCTCTGA-AACGCGGTCACc<br>3'	-10.41	140.00	
		2390~2412	23	miRNA: 3' ucuuaaaaGUAUUACGUCAGUGu<br>5'<br>|| ||||||||| Target:5' cggctacgCACCCTGCAGTCACt<br>3'	-13.50	151.00	
		711~739	29	miRNA: 3' ucUUAA--AAGUA--UUAC--<br>GUCAGUGu 5'<br>|:|| | ||| |||| :|||||| Target:5'<br>aaAGTTGCTCCATACAATGACTAGTCACc 3'	-8.90	147.00	
170	hsa-miR-3118	2719~2740	22	miRNA: 3' ucUUAAAAGUAUUACGUCAGUGu<br>5'<br>|: | | | || ||:||||| Target:5' agAGCTCTGA-AACGCGGTCACc<br>3'	-10.41	140.00	Show result profile
		2390~2412	23	miRNA: 3' ucuuaaaaGUAUUACGUCAGUGu<br>5'<br>|| ||||||||| Target:5' cggctacgCACCCTGCAGTCACt<br>3'	-13.50	151.00	
		711~739	29	miRNA: 3' ucUUAA--AAGUA--UUAC--<br>GUCAGUGu 5'<br>|:|| | ||| |||| :|||||| Target:5'<br>aaAGTTGCTCCATACAATGACTAGTCACc 3'	-8.90	147.00	
		2719~2740	22	miRNA: 3' ucUUAAAAGUAUUACGUCAGUGu<br>5'<br>|: | | | || ||:||||| Target:5' agAGCTCTGA-AACGCGGTCACc<br>3'	-10.41	140.00	
		2390~2412	23	miRNA: 3' ucuuaaaaGUAUUACGUCAGUGu<br>5'	-13.50	151.00	

<table>
<tr><td></td><td></td><td></td><td></td><td>|| |||||||||
Target:5' cggctacgCACCCTGCAGTCACt
3'</td><td></td><td></td><td></td></tr>
<tr><td></td><td></td><td>711~739</td><td>29</td><td>miRNA: 3' ucUUAA--AAGUA--UUAC--
GUCAGUGu 5'
|:|| | ||| ||||
:||||||
Target:5'
aaAGTTGCTCCATACAATGACTAGTCACc 3'</td><td>-8.90</td><td>147.00</td><td></td></tr>
<tr><td></td><td></td><td>2719~2740</td><td>22</td><td>miRNA: 3' ucUUAAAAGUAUUACGUCAGUGu
5'
|: | | | || ||:|||||
Target:5' agAGCTCTGA-AACGCGGTCACc
3'</td><td>-10.41</td><td>140.00</td><td></td></tr>
<tr><td></td><td></td><td>2390~2412</td><td>23</td><td>miRNA: 3' ucuuaaaaGUAUUACGUCAGUGu
5'
|| |||||||||
Target:5' cggctacgCACCCTGCAGTCACt
3'</td><td>-13.50</td><td>151.00</td><td></td></tr>
<tr><td></td><td></td><td>711~739</td><td>29</td><td>miRNA: 3' ucUUAA--AAGUA--UUAC--
GUCAGUGu 5'
|:|| | ||| ||||
:||||||
Target:5'
aaAGTTGCTCCATACAATGACTAGTCACc 3'</td><td>-8.90</td><td>147.00</td><td></td></tr>
<tr><td></td><td></td><td>2719~2740</td><td>22</td><td>miRNA: 3' ucUUAAAAGUAUUACGUCAGUGu
5'
|: | | | || ||:|||||
Target:5' agAGCTCTGA-AACGCGGTCACc
3'</td><td>-10.41</td><td>140.00</td><td></td></tr>
<tr><td>171</td><td>hsa-miR-3120</td><td>1147~1166</td><td>20</td><td>miRNA: 3' acGGACAGAUGUGAACGACAc 5'
::||||||::: |||||||
Target:5' gtTTTGTCTGTG-TTGCTGTt 3'</td><td>-15.80</td><td>162.00</td><td>Show result profile</td></tr>
<tr><td>172</td><td>hsa-miR-3122</td><td>219~244</td><td>26</td><td>miRNA: 3' uucuGGCAGGAG-A---
ACAGGGUUg 5'
::| ::|| |
||||||||
Target:5'
ccatTTGGTTTCGTGGCTGTCCCAAc 3'</td><td>-17.80</td><td>146.00</td><td>Show result profile</td></tr>
<tr><td></td><td></td><td>2151~2172</td><td>22</td><td>miRNA: 3' uucuggcaggagaaCAGGGUUg
5'
|||||||
Target:5' ggcccaagtgcaggGTCCCAAg
3'</td><td>-13.40</td><td>140.00</td><td></td></tr>
<tr><td>173</td><td>hsa-miR-3125</td><td>1115~1134</td><td>20</td><td>miRNA: 3' agAGAGGUGUCGAAGGAGAu 5'
||| |:| ||||||:|
Target:5' gtTCTGCGCCACTTCCTTTg 3'</td><td>-19.50</td><td>146.00</td><td>Show result profile</td></tr>
<tr><td>174</td><td>hsa-miR-3126-5p</td><td>1624~1645</td><td>22</td><td>miRNA: 3' acGAAGACCGUAGACAGGGAGu
5'
| || ||::|||||||||:
Target:5' caCCTCAGGTGTCTGTCCCTTg
3'</td><td>-21.10</td><td>160.00</td><td>Show result profile</td></tr>
</table>

<table>
<tr><td>175</td><td>hsa-miR-3126-3p</td><td>1671~1692</td><td>22</td><td>miRNA: 3' agacaCACUGCCUACGGUCUAc 5'
|||:| | |||||:|
Target:5' cacccGTGGCAAACGCCAGGTg 3'</td><td>-17.70</td><td>141.00</td><td>Show result profile</td></tr>
<tr><td>176</td><td>hsa-miR-3127</td><td>2213~2234</td><td>22</td><td>miRNA: 3' gaaggGUAAGGUGUUCGGGACUa 5'
|: |||| ::||||||:
Target:5' gagggCGGTCCA-GGGCCCTGGg 3'</td><td>-19.50</td><td>141.00</td><td>Show result profile</td></tr>
<tr><td>177</td><td>hsa-miR-3129</td><td>887~908</td><td>22</td><td>miRNA: 3' uuUGGUUAGAGAUGUGAUGACg 5'
::||:| |:| |||:|||
Target:5' atGTCAGTATTTCAACTGCTGt 3'</td><td>-12.80</td><td>144.00</td><td>Show result profile</td></tr>
<tr><td rowspan="2">178</td><td rowspan="2">hsa-miR-3130-5p</td><td>2127~2145</td><td>19</td><td>miRNA: 3' ccgACGUGGCCUCUGACCCAu 5'
|| :|:||| :||||||
Target:5' cggTG-GCTGGA-GCTGGGTc 3'</td><td>-21.50</td><td>140.00</td><td rowspan="2">Show result profile</td></tr>
<tr><td>2127~2145</td><td>19</td><td>miRNA: 3' ccgACGUGGCCUCUGACCCAu 5'
|| :|:||| :||||||
Target:5' cggTG-GCTGGA-GCTGGGTc 3'</td><td>-21.50</td><td>140.00</td></tr>
<tr><td rowspan="3">179</td><td rowspan="3">hsa-miR-3131</td><td>1~20</td><td>20</td><td>miRNA: 3' uucCGGGAAGGUGGU--CAGGAGCu 5'
|||| |:||| |||||||
Target:5' ---GCCC--GCGCCAGGGTCCTCGg 3'</td><td>-26.00</td><td>158.00</td><td rowspan="3">Show result profile</td></tr>
<tr><td>2659~2688</td><td>30</td><td>miRNA: 3' uucCGGGAAG-----GUGGU--CAGGAGCu 5'
|::|||: :|||| |||||||
Target:5' aatGTTCTTTTGTAGTACCACGGTCCTCGg 3'</td><td>-22.70</td><td>158.00</td></tr>
<tr><td>86~109</td><td>24</td><td>miRNA: 3' uucCGGGAAGGUG-GUCAGGAGcu 5'
||| ||:| |:||||||
Target:5' gccGCCGCCCCGCTCGGTCCTCca 3'</td><td>-21.20</td><td>140.00</td></tr>
<tr><td>180</td><td>hsa-miR-3134</td><td>346~368</td><td>23</td><td>miRNA: 3' uuauacaUCAGAAAAUAGGUAGu 5'
|| : |||||||||
Target:5' ttttcaaAGATGATTATCCATCt 3'</td><td>-9.90</td><td>152.00</td><td>Show result profile</td></tr>
<tr><td>181</td><td>hsa-miR-3138</td><td>91~119</td><td>29</td><td>miRNA: 3' ugaGGGAGA---UGGA-GU-GACAGGUGu 5'
||| || ||| || ||||||:|
Target:5' cgcCCCGCTCGGTCCTCCACCTGTCCGCt 3'</td><td>-24.40</td><td>145.00</td><td>Show result profile</td></tr>
</table>

<table>
<tr><td>182</td><td>hsa-miR-3140</td><td>2300~2320</td><td>21</td><td>miRNA: 3' ugauggACUUAAGGGUUUUCGa 5'
||| || |||||:||
Target:5' tagaggTGACTT-CCAAAGGCc 3'</td><td>-11.20</td><td>143.00</td><td>Show result profile</td></tr>
<tr><td>183</td><td>hsa-miR-3141</td><td>1831~1851</td><td>21</td><td>miRNA: 3' agGAGGAG--GUGGGCGGGAg 5'
| |||| || |||:|||
Target:5' tgCGCCTCAGCAGCCGTCCTg 3'</td><td>-21.40</td><td>143.00</td><td>Show result profile</td></tr>
<tr><td>184</td><td>hsa-miR-3144-5p</td><td>2128~2149</td><td>22</td><td>miRNA: 3' gauauauagagaAACCAGGGGa 5'
| |||||||
Target:5' ggtggctggagcTGGGTCCCCg 3'</td><td>-15.50</td><td>142.00</td><td>Show result profile</td></tr>
<tr><td>185</td><td>hsa-miR-3145</td><td>511~534</td><td>24</td><td>miRNA: 3' guuAAGGUUUGUGAGUUUUAUAGa 5'
|||:|||:: | |||||||
Target:5' aacTTCTAAATGAACCAAATATCc 3'</td><td>-7.80</td><td>169.00</td><td>Show result profile</td></tr>
<tr><td rowspan="3">186</td><td rowspan="3">hsa-miR-3147</td><td>2674~2695</td><td>22</td><td>miRNA: 3' aguGUGGGAGGAGUGACGGGUUGg 5'
||| |||||: |||:|||
Target:5' tacCACGGTCCTCG--GCCTAACg 3'</td><td>-20.30</td><td>149.00</td><td rowspan="3">Show result profile</td></tr>
<tr><td>1229~1251</td><td>23</td><td>miRNA: 3' agUGUGGGAGGAGUGACGGGUUGg 5'
:|: ||||| || |||||::|
Target:5' agGCGGCCTCC-CAGTGCCCGGCc 3'</td><td>-33.00</td><td>141.00</td></tr>
<tr><td>1254~1276</td><td>23</td><td>miRNA: 3' aguGUGGGAGGAGUGACGGGUUGg 5'
||||| ||| :| |||| ||
Target:5' cccCACCCACCT-GCAGCCCCACc 3'</td><td>-24.00</td><td>140.00</td></tr>
<tr><td>187</td><td>hsa-miR-3148</td><td>1677~1701</td><td>25</td><td>miRNA: 3' uuCGUGUGUGGU-CA--AAAAAGGu 5'
||| ||:||| || ||||||:
Target:5' tgGCAAACGCCAGGTGCTTTTTCTg 3'</td><td>-16.90</td><td>154.00</td><td>Show result profile</td></tr>
<tr><td rowspan="2">188</td><td rowspan="2">hsa-miR-3149</td><td>705~728</td><td>24</td><td>miRNA: 3' uaUGUGUGUGUAU-AGGUAUGUUu 5'
|: :| | |: |||||||||
Target:5' aaATCTAAAGTTGCTCCATACAAt 3'</td><td>-9.40</td><td>153.00</td><td rowspan="2">Show result profile</td></tr>
<tr><td>800~822</td><td>23</td><td>miRNA: 3' uauguGUGUGUAUAGGUAUGUUu 5'
::| |:| ||||||||:
Target:5' tgaatTGCCCGTTTCCATACAGg 3'</td><td>-13.70</td><td>146.00</td></tr>
<tr><td></td><td></td><td>2034~2051</td><td>18</td><td>miRNA: 3' ggUUGGAGCUCCUAGAGGGGUc 5'
|||| | || ||||||||
Target:5' ccAACC-C-AG--TCTCCCCAt</td><td>-21.10</td><td>154.00</td><td></td></tr>
</table>

<table>
<tr><td rowspan="3">189</td><td rowspan="3">hsa-miR-3150</td><td></td><td></td><td>3'</td><td></td><td></td><td rowspan="3">Show result profile</td></tr>
<tr><td>2175~2194</td><td>20</td><td>miRNA: 3' gguuggaGCUCCUAGAGGGGUc 5'
                 ||:|| |||||||
Target:5' ggcagggCGGGG--CTCCCCAa 3'</td><td>-21.20</td><td>151.00</td></tr>
<tr><td>1238~1259</td><td>22</td><td>miRNA: 3' gguuggagcuCCUAGAGGGGUc 5'
                    || |||||||
Target:5' cccagtgcccGGCCCTCCCCAc 3'</td><td>-19.20</td><td>144.00</td></tr>
<tr><td>190</td><td>hsa-miR-3151</td><td>1256~1276</td><td>21</td><td>miRNA: 3' uggacuaGGGUAACGGGGUGg 5'
                 ||:: |||||||
Target:5' ccacccaCCTGCAGCCCCACc 3'</td><td>-21.10</td><td>146.00</td><td>Show result profile</td></tr>
<tr><td>191</td><td>hsa-miR-3153</td><td>2370~2391</td><td>22</td><td>miRNA: 3' uuUACAGGGAUGAGCGAAAGGGg 5'
            :|| ||:|::| |||||:||
Target:5' caGTG-CCTTGTTGGCTTTTCCg 3'</td><td>-20.50</td><td>152.00</td><td>Show result profile</td></tr>
<tr><td>192</td><td>hsa-miR-3155</td><td>2043~2065</td><td>23</td><td>miRNA: 3' ucAAGGGU--GA-CGUCUCGGACc 5'
            | |||| || || ||||:||
Target:5' tcTCCCCATCCTAGC-GAGCTTGg 3'</td><td>-20.20</td><td>140.00</td><td>Show result profile</td></tr>
<tr><td rowspan="3">193</td><td rowspan="3">hsa-miR-3156</td><td>1326~1350</td><td>25</td><td>miRNA: 3' acaGAGGGUGAA---GGUCUAGAAa 5'
             |||: ||| |::||||||
Target:5' aggCTCTGTCTTGCCCTGGATCTTt 3'</td><td>-15.10</td><td>143.00</td><td rowspan="3">Show result profile</td></tr>
<tr><td>1326~1350</td><td>25</td><td>miRNA: 3' acaGAGGGUGAA---GGUCUAGAAa 5'
             |||: ||| |::||||||
Target:5' aggCTCTGTCTTGCCCTGGATCTTt 3'</td><td>-15.10</td><td>143.00</td></tr>
<tr><td>1326~1350</td><td>25</td><td>miRNA: 3' acaGAGGGUGAA---GGUCUAGAAa 5'
             |||: ||| |::||||||
Target:5' aggCTCTGTCTTGCCCTGGATCTTt 3'</td><td>-15.10</td><td>143.00</td></tr>
<tr><td rowspan="2">194</td><td rowspan="2">hsa-miR-3157</td><td>1906~1931</td><td>26</td><td>miRNA: 3' ucUGACG----UGAUCGGACCGACUu 5'
            ||||| ||:|||||||||:
Target:5' ccACTGCAGGAAGTGGCCTGGCTGGg 3'</td><td>-26.50</td><td>158.00</td><td rowspan="2">Show result profile</td></tr>
<tr><td>992~1019</td><td>28</td><td>miRNA: 3' ucUGACGUG-AUCG-----GACCGACuu 5'
            :|||||| |:|| |||||||
Target:5' gaGCTGCACTTGGCCTCAGCTGGCTGta 3'</td><td>-23.90</td><td>148.00</td></tr>
</table>

		2108~2137	30	miRNA: 3' ucUGACGUGAUC------GG--ACCGACUu 5'<br>:|||| ||:| || ||||||:<br>Target:5' ctGCTGCCCTGGCACCCCCCGGTGGCTGGa 3'	-22.20	140.00	
195	hsa-miR-3158	1968~1991	24	miRNA: 3' caggacGUCUCU--CCUUCGGGAa 5'<br>|||||| ||||||||| Target:5' agtggaCAGAGATGGGAAGCCCTg 3'	-23.90	170.00	Show result profile
		1968~1991	24	miRNA: 3' caggacGUCUCU--CCUUCGGGAa 5'<br>|||||| ||||||||| Target:5' agtggaCAGAGATGGGAAGCCCTg 3'	-23.90	170.00	
196	hsa-miR-3162	1027~1055	29	miRNA: 3' gagGGGUGGGAAGAU------GAGGGAUu 5'<br>||| |||| ||: |||:||| Target:5' gcaCCCTCCCTCCTGCCGCTCCTCTCTAg 3'	-23.60	146.00	Show result profile
197	hsa-miR-3163	860~882	23	miRNA: 3' cagaaUGA-CGGGAGUAAAAUau 5'<br>||| ||::|:|||||| Target:5' gtaaaACTCGCTTTTATTTTAat 3'	-10.40	145.00	Show result profile
		2506~2526	21	miRNA: 3' cagaaUGACGGGAGUAAAAUAu 5'<br>| || :||:|||||:| Target:5' cccagAATG-TCTTATTTTGTa 3'	-9.40	140.00	
198	hsa-miR-3165	2820~2841	22	miRNA: 3' acuccaguguaaCGUAGGUGGa 5'<br>| ||||||| Target:5' cactgtatctaaGAATCCACCa 3'	-9.79	142.00	Show result profile
199	hsa-miR-3166	1757~1782	26	miRNA: 3' auCCGGUCAUCCGU---A-ACAGACgc 5'<br>|||| |:|||| | |||||| Target:5' agGGCC-CTGGGCAGCCTGTGTCTGga 3'	-23.10	140.00	Show result profile
200	hsa-miR-3169	86~107	22	miRNA: 3' gauacaCGGUUCGUGUCAGGAu 5'<br>||| || |:||||| Target:5' gccgccGCCCCGCTCGGTCCTc 3'	-17.10	140.00	Show result profile
201	hsa-miR-3171	2557~2578	22	miRNA: 3' cuAUAUAUGUCUAAGGUAUGUAga 5'<br>|:||||:\:| |: |||||| Target:5' caTGTATATGG--TTAATACATat 3'	-11.60	146.00	Show result profile

<table>
<tr><td>202</td><td>hsa-miR-3175</td><td>2029~2050</td><td>22</td><td>miRNA: 3' ugcagugacgcaagAGAGGGGc 5'
|||||||
Target:5' ggctcccaacccagTCTCCCCa 3'</td><td>-13.70</td><td>140.00</td><td>Show result profile</td></tr>
<tr><td rowspan="2">203</td><td rowspan="2">hsa-miR-3176</td><td>1490~1509</td><td>20</td><td>miRNA: 3' ggCCAUCAG-GGUCCGGUCa 5'
|| :| | | |||||||
Target:5' ttGGGGGCCACAAGGCCAGg 3'</td><td>-18.40</td><td>149.00</td><td rowspan="2">Show result profile</td></tr>
<tr><td>457~475</td><td>19</td><td>miRNA: 3' ggccaucagggUCCGGUCa 5'
|||||||
Target:5' acaaggactggAGGCCAGc 3'</td><td>-15.01</td><td>140.00</td></tr>
<tr><td>204</td><td>hsa-miR-3177</td><td>1812~1834</td><td>23</td><td>miRNA: 3' ugcACAGGGGUC--ACGGCACGu 5'
|||||: :| ||:||||
Target:5' gtcTGTCCTGGGGAGGCTGTGCg 3'</td><td>-22.50</td><td>140.00</td><td>Show result profile</td></tr>
<tr><td rowspan="2">205</td><td rowspan="2">hsa-miR-3180-5p</td><td>1758~1784</td><td>27</td><td>miRNA: 3' gcugcacCCCGCC--UCGCAGACCUUc 5'
|||| | |:||||||||
Target:5' gggccctGGGCAGCCTGTGTCTGGAAt 3'</td><td>-22.60</td><td>160.00</td><td rowspan="2">Show result profile</td></tr>
<tr><td>939~968</td><td>30</td><td>miRNA: 3' gcugcaCCCCGCCUC-----GC-AGACCUUc 5'
||||| ||| || |||||:|
Target:5' cccccaGGGGC-GAGTTCCTCGCTCTGGGAt 3'</td><td>-22.00</td><td>142.00</td></tr>
<tr><td>206</td><td>hsa-miR-3180-3p</td><td>76~97</td><td>22</td><td>miRNA: 3' ccgGAGGCCUUCGAGGCGGGGu 5'
| ||| : | |||||||
Target:5' aagCGCCGCCGCCGCCGCCCCg 3'</td><td>-26.30</td><td>151.00</td><td>Show result profile</td></tr>
<tr><td rowspan="2">207</td><td rowspan="2">hsa-miR-3180-5p</td><td>1758~1784</td><td>27</td><td>miRNA: 3' gcugcacCCCGCC--UCGCAGACCUUc 5'
|||| | |:||||||||
Target:5' gggccctGGGCAGCCTGTGTCTGGAAt 3'</td><td>-22.60</td><td>160.00</td><td rowspan="2">Show result profile</td></tr>
<tr><td>939~968</td><td>30</td><td>miRNA: 3' gcugcaCCCCGCCUC-----GC-AGACCUUc 5'
||||| ||| || |||||:|
Target:5' cccccaGGGGC-GAGTTCCTCGCTCTGGGAt 3'</td><td>-22.00</td><td>142.00</td></tr>
<tr><td>208</td><td>hsa-miR-3180-3p</td><td>76~97</td><td>22</td><td>miRNA: 3' ccgGAGGCCUUCGAGGCGGGGu 5'
| ||| : | |||||||
Target:5' aagCGCCGCCGCCGCCGCCCCg 3'</td><td>-26.30</td><td>151.00</td><td>Show result profile</td></tr>
</table>

<table>
<tr><td rowspan="2">209</td><td rowspan="2">hsa-miR-3180-5p</td><td>1758~1784</td><td>27</td><td>miRNA: 3' gcugcacCCCGCC--UCGCAGACCUUc 5'
|||| | |:||||||||
Target:5' gggccctGGGCAGCCTGTGTCTGGAAt 3'</td><td>-22.60</td><td>160.00</td><td rowspan="2">Show result profile</td></tr>
<tr><td>939~968</td><td>30</td><td>miRNA: 3' gcugcaCCCCGCCUC-----GC-AGACCUUc 5'
||||| ||| || |||||:|
Target:5' cccccaGGGGC-GAGTTCCTCGCTCTGGGAt 3'</td><td>-22.00</td><td>142.00</td></tr>
<tr><td>210</td><td>hsa-miR-3180-3p</td><td>76~97</td><td>22</td><td>miRNA: 3' ccgGAGGCCUUCGAGGCGGGGu 5'
| ||| : | |||||||
Target:5' aagCGCCGCCGCCGCCGCCCCg 3'</td><td>-26.30</td><td>151.00</td><td>Show result profile</td></tr>
<tr><td rowspan="2">211</td><td rowspan="2">hsa-miR-3180</td><td>79~97</td><td>19</td><td>miRNA: 3' gaGGCCUUCGAGGCGGGGu 5'
||| : | |||||||
Target:5' cgCCGCCGCCGCCGCCCCg 3'</td><td>-24.00</td><td>149.00</td><td rowspan="2">Show result profile</td></tr>
<tr><td>79~97</td><td>19</td><td>miRNA: 3' gaGGCCUUCGAGGCGGGGu 5'
||| : | |||||||
Target:5' cgCCGCCGCCGCCGCCCCg 3'</td><td>-24.00</td><td>149.00</td></tr>
<tr><td>212</td><td>hsa-miR-3183</td><td>173~198</td><td>26</td><td>miRNA: 3' aggcUCG-CUGA---GGCUCUCUCCg 5'
||| |||| || :||||||
Target:5' cctcAGCAGACTCGCCCAGGAGAGGa 3'</td><td>-21.10</td><td>146.00</td><td>Show result profile</td></tr>
<tr><td>213</td><td>hsa-miR-3188</td><td>2071~2092</td><td>22</td><td>miRNA: 3' ggGGCAUAGGCGUGUUUCGGAGa 5'
| || |:||: : ||||||:
Target:5' ctCAGT-TTCGTTTCAAGCCTTg 3'</td><td>-13.10</td><td>140.00</td><td>Show result profile</td></tr>
<tr><td>214</td><td>hsa-miR-3189</td><td>2142~2160</td><td>19</td><td>miRNA: 3' gauGGGGUAGUCUGGGUUCcc 5'
||||:| |:||||||
Target:5' ggtCCCCGT--GGCCCAAGtg 3'</td><td>-23.70</td><td>142.00</td><td>Show result profile</td></tr>
<tr><td>215</td><td>hsa-miR-3190</td><td>749~770</td><td>22</td><td>miRNA: 3' agagaCCGGCAGAUGGAAGGUGu 5'
|| || | |:||||||:
Target:5' tgggcGGGCG-CCATCTTCCATt 3'</td><td>-17.70</td><td>141.00</td><td>Show result profile</td></tr>
<tr><td>216</td><td>hsa-miR-3191</td><td>2129~2149</td><td>21</td><td>miRNA: 3' gaCAGACCGGUCGAUGCAGGGGu 5'
| |||| ||||: ||||||
Target:5' gtGGCTGG--AGCTGGGTCCCCg 3'</td><td>-27.60</td><td>141.00</td><td>Show result profile</td></tr>
<tr><td></td><td></td><td>1221~1243</td><td>23</td><td>miRNA: 3' aaggugacgaUGUUGGAGGGUCu 5'
:|::||||||||
Target:5' gtgagaggagGCGGCCTCCCAGt</td><td>-22.40</td><td>153.00</td><td></td></tr>
</table>

<table>
<tr><td rowspan="5">217</td><td rowspan="5">hsa-miR-3192</td><td></td><td></td><td>3'</td><td></td><td></td><td rowspan="5">Show result profile</td></tr>
<tr><td>1934~1957</td><td>24</td><td>miRNA: 3' aaggugAC-GAUGUUGGAGGGUCu
5'
                || | :: |||||||||
Target:5' gttgggTGCCGGTCACCTCCCAGc
3'</td><td>-19.30</td><td>153.00</td></tr>
<tr><td>1994~2016</td><td>23</td><td>miRNA: 3' aagGUGACGAUGUUG-GAGGGUCu
5'
             ||| || |::: |||||||
Target:5' ggaCACAGC-CCGGTGCTCCCAGc
3'</td><td>-17.10</td><td>151.00</td></tr>
<tr><td>2488~2511</td><td>24</td><td>miRNA: 3' aagGUGACGAUGUUG---
GAGGGUCu 5'
             :|:| |::|:|
|||||||
Target:5' gaaTATT--
TGTAGCCCGCTCCCAGa 3'</td><td>-16.20</td><td>148.00</td></tr>
<tr><td>1045~1066</td><td>22</td><td>miRNA: 3' aaGGUGACGAUGUUGGAGGGUCu
5'
            || || ||| |||||:|:||
Target:5' ctCCTCT-CTAGAACCTTCTAGa
3'</td><td>-23.80</td><td>144.00</td></tr>
<tr><td rowspan="2">218</td><td rowspan="2">hsa-miR-3193</td><td>1301~1321</td><td>21</td><td>miRNA: 3' ugaGGAGUCUAGGAUGCGUCCu
5'
             |:||:||| | |||||||
Target:5' ctgCTTCGGATGC-ACGCAGGc
3'</td><td>-24.40</td><td>166.00</td><td rowspan="2">Show result profile</td></tr>
<tr><td>952~974</td><td>23</td><td>miRNA: 3' ugAGGAGUCUAGG--AUGCGUCCu
5'
            ||||| | ||: |:|||||
Target:5' gtTCCTC-GCTCTGGGATGCAGGc
3'</td><td>-20.90</td><td>141.00</td></tr>
<tr><td>219</td><td>hsa-miR-3194</td><td>2117~2138</td><td>22</td><td>miRNA: 3' guCG-GGAGGACCACCGACCgg
5'
            || || || |||||||||
Target:5' tgGCACCCCCCGGTGGCTGGag
3'</td><td>-28.60</td><td>151.00</td><td>Show result profile</td></tr>
<tr><td rowspan="3">220</td><td rowspan="3">hsa-miR-3196</td><td>80~97</td><td>18</td><td>miRNA: 3' cucCGGGGACGGCGGGGc 5'
             ||| | ||||||||
Target:5' gccGCCGCCGCCGCCCCg 3'</td><td>-29.30</td><td>159.00</td><td rowspan="3">Show result profile</td></tr>
<tr><td>1032~1049</td><td>18</td><td>miRNA: 3' cuccGGGGACGGCGGGGc 5'
              |:||||||||:||
Target:5' ctccCTCCTGCCGCTCCt 3'</td><td>-25.10</td><td>150.00</td></tr>
<tr><td>129~146</td><td>18</td><td>miRNA: 3' cuCCGGGGACGGCGGGgc 5'
            |||: | |||||||
Target:5' ggGGCTGCGGCCGCCCga 3'</td><td>-25.70</td><td>140.00</td></tr>
<tr><td></td><td></td><td></td><td></td><td>miRNA: 3' gcGGAAAGG----CUCGG--</td><td></td><td></td><td></td></tr>
</table>

<table>
<tr><td rowspan="2">221</td><td rowspan="2">hsa-miR-3197</td><td>1811~1839</td><td>29</td><td>ACGCGGAGg 5'
:|| ||| ||| |
||||||||
Target:5'
ggTCTGTCCTGGGGAGGCTGTGCGCCTCa 3'</td><td>-24.80</td><td>161.00</td><td rowspan="2">Show result profile</td></tr>
<tr><td>2761~2786</td><td>26</td><td>miRNA: 3' gcggaAAGGCUCGGA---
CGCGGAGg 5'
|||| :|| |
|:|||||
Target:5'
gtgtgTTCCCGGCATGAGGTGCCTCg 3'</td><td>-20.50</td><td>142.00</td></tr>
<tr><td rowspan="2">222</td><td rowspan="2">hsa-miR-3198</td><td>2251~2275</td><td>25</td><td>miRNA: 3' agAGGUAAGGG---
GUCCUGAGGUg 5'
|||| :|| ::||||||||
Target:5'
ccTCCAGGTCCGTGTGGGACTCCAg 3'</td><td>-25.00</td><td>160.00</td><td rowspan="2">Show result profile</td></tr>
<tr><td>1524~1545</td><td>22</td><td>miRNA: 3' agagguAAGGGGUCCUGAGGug
5'
| ||: ||||||||
Target:5' gggggcTGCCTGAGGACTCCta
3'</td><td>-19.70</td><td>140.00</td></tr>
<tr><td rowspan="2">223</td><td rowspan="2">hsa-miR-3199</td><td>2025~2047</td><td>23</td><td>miRNA: 3' uuGAAAGAGGAUUCCGUCAGGGa
5'
|| |||| || |||||:|
Target:5' ctCTGGCTCCCAACCCAGTCTCc
3'</td><td>-16.70</td><td>149.00</td><td rowspan="2">Show result profile</td></tr>
<tr><td>2025~2047</td><td>23</td><td>miRNA: 3' uuGAAAGAGGAUUCCGUCAGGGa
5'
|| |||| || |||||:|
Target:5' ctCTGGCTCCCAACCCAGTCTCc
3'</td><td>-16.70</td><td>149.00</td></tr>
<tr><td rowspan="2">224</td><td rowspan="2">hsa-miR-3200-5p</td><td>1613~1634</td><td>22</td><td>miRNA: 3' uggAACACGCGGAAGAGUCUAa
5'
| | |:||| |||||:|
Target:5' gcaTAGGGTGCCACCTCAGGTg
3'</td><td>-16.20</td><td>143.00</td><td rowspan="2">Show result profile</td></tr>
<tr><td>1071~1098</td><td>28</td><td>miRNA: 3' uggaACACG-CGGAA-----
GAGUCUaa 5'
||||| ||:||
||||||
Target:5'
gggcTGTGCTGCTTTTGAGCCTCAGAcc 3'</td><td>-15.30</td><td>142.00</td></tr>
<tr><td rowspan="2">225</td><td rowspan="2">hsa-miR-323-5p</td><td>195~220</td><td>26</td><td>miRNA: 3' cgCUUGCGCGGUGC----
CUGGUGGa 5'
||| || : : |
|||||||
Target:5'
agGAAAGCATGGAGGAAAGACCACCc 3'</td><td>-15.90</td><td>146.00</td><td rowspan="2">Show result profile</td></tr>
<tr><td>153~174</td><td>22</td><td>miRNA: 3' cgCUUGCGCGGUGCCUGGUGGa
5'
|||| :|:|: |||:|:||
Target:5' ttGAACATGTCGGGGATCGCCc
3'</td><td>-27.20</td><td>140.00</td></tr>
</table>

<table>
<tr><td>226</td><td>hsa-miR-324-5p</td><td>946~971</td><td>26</td><td>miRNA: 3' ugUGGUUACGG---
GAUCCCCUACGc 5'
:| |:| || ||
|||||||
Target:5'
ggGCGAGTTCCTCGCTCTGGGATGCa 3'</td><td>-18.40</td><td>153.00</td><td>Show result profile</td></tr>
<tr><td rowspan="2">227</td><td rowspan="2">hsa-miR-328</td><td>2209~2231</td><td>23</td><td>miRNA: 3' ugCCUUCCCGUC-UCUCCCGGuc
5'
||:|||||:| ||||||
Target:5' caGGGAGGGCGGTCCAGGGCCct
3'</td><td>-29.50</td><td>148.00</td><td rowspan="2">Show result profile</td></tr>
<tr><td>1741~1764</td><td>24</td><td>miRNA: 3' ugccUUCCCGUC--UCUCCCGGuc
5'
|: | ||| ||||||||
Target:5' ccttAGCGCCAGGTAGAGGGCCct
3'</td><td>-18.50</td><td>140.00</td></tr>
<tr><td rowspan="2">228</td><td rowspan="2">hsa-miR-330-3p</td><td>1066~1087</td><td>22</td><td>miRNA: 3' agaGACGUCCGGCAC-ACGAAAcg
5'
||| |||:||| ||||||
Target:5' aacCTG--GGCTGTGCTGCTTTtg
3'</td><td>-18.70</td><td>148.00</td><td rowspan="2">Show result profile</td></tr>
<tr><td>2358~2380</td><td>23</td><td>miRNA: 3' agAGACGUC-CGGCACACGAAACg
5'
|||||:| ||| |||| |||
Target:5' acTCTGCGGTGCC-AGTGCCTTGt
3'</td><td>-25.00</td><td>140.00</td></tr>
<tr><td>229</td><td>hsa-miR-331-3p</td><td>926~949</td><td>24</td><td>miRNA: 3' aaGAUCCUAUCC---GGGUCCCCg
5'
|| ||:|| | ||||||||
Target:5' taCTTGGGTAAGTCCCCCAGGGGc
3'</td><td>-25.70</td><td>163.00</td><td>Show result profile</td></tr>
<tr><td>230</td><td>hsa-miR-339-5p</td><td>2160~2182</td><td>23</td><td>miRNA: 3' gcaCUCGAGGACCUCCUGUCCCu
5'
|:|: || ||||:|||||
Target:5' gcaGGGTCCCAAGAGGGCAGGGc
3'</td><td>-25.10</td><td>152.00</td><td>Show result profile</td></tr>
<tr><td>231</td><td>hsa-miR-33b*</td><td>130~153</td><td>24</td><td>miRNA: 3' ccCGACG-UGAC-GGCUCCGUGAc
5'
||||| :| | |||||| |||
Target:5' ggGCTGCGGCCGCCCGAGGGACTt
3'</td><td>-28.30</td><td>140.00</td><td>Show result profile</td></tr>
<tr><td rowspan="2">232</td><td rowspan="2">hsa-miR-340*</td><td>1213~1235</td><td>23</td><td>miRNA: 3' cgauaUUUCA-UUGACUCUGCCu
5'
||||| |: |||:|||
Target:5' cattgAAAGTGAGAGGAGGCGGc
3'</td><td>-16.70</td><td>141.00</td><td rowspan="2">Show result profile</td></tr>
<tr><td>1961~1983</td><td>23</td><td>miRNA: 3' cgauaUUUCA-UUGACUCUGCCu
5'
| ||| :|| ||||:||
Target:5' aaggcACAGTGGACAGAGATGGg
3'</td><td>-14.60</td><td>141.00</td></tr>
<tr><td></td><td>hsa-</td><td></td><td></td><td>miRNA: 3' aguuagugucuAUCGUGGGGa 5'</td><td></td><td></td><td></td></tr>
</table>

233	miR-342-5p	2106~2126	21	|:||||||| Target:5' ccctgctgcccTGGCACCCCc 3'	-14.40	146.00	Show result profile
234	hsa-miR-34a	1893~1914	22	miRNA: 3' ugUUGGUCGAUUCUGUGACGgu<br>5'<br>::|:: ||:|| |||||| Target:5' ggGGCTGCCTGAGCCACTGCag<br>3'	-16.60	144.00	Show result profile
235	hsa-miR-34b*	1879~1903	25	miRNA: 3' guuaGUC--<br>GAUUACUGUGACGGAu 5' ||| || ||: :|||||| Target:5'<br>cggcCAGGCCTCTTGGGGCTGCCTg 3'	-20.60	141.00	Show result profile
236	hsa-miR-34c-5p	545~574	30	miRNA: 3' cgUUAGUC----GAU-UG--<br>AUGUGACGGa 5' || ||| ||| || | :|||||| Target:5'<br>tcAAGCAGAGGCCTACACGATTTACTGCCa 3'	-14.91	141.00	Show result profile
237	hsa-miR-3605-5p	348~371	24	miRNA: 3' ccgaaggaAC-GAUAGGUAGGAGu<br>5' || :||||||||:|| Target:5' ttcaaagaTGATTATCCATCTTCg<br>3'	-17.00	147.00	Show result profile
238	hsa-miR-3605-3p	186~209	24	miRNA: 3' gaucUCCUGUCCAUU-GUGCCUCc<br>5' |||| ||| || ||:|||| Target:5' gcccAGGAGAGGAAAGCATGGAGg<br>3'	-26.00	155.00	Show result profile
239	hsa-miR-3607-3p	677~696	20	miRNA: 3' guagUCUUUCGCAAAUGUca 5' |||| :||||||| Target:5' ggcaAGAACTTGTTTACAac 3'	-8.50	140.00	Show result profile
240	hsa-miR-361-3p	720~746	27	miRNA: 3' uuUAGUCUUAGU-----<br>GUGGACCCCCu 5' 						
								| Target:5' ccAT-<br>ACAATGACTAGTCACCTGGGGGg 3'	-23.10	160.00	Show result profile
		1977~1996	20	miRNA: 3' uuUAGUCUUAGUGUGGACCCCCu<br>5' || :|||	||||||| Target:5' agATGGGAAGC---CCTGGGGGa<br>3'	-22.80	158.00	
		1508~1529	22	miRNA: 3' uuuaGUCUUAGUGUGGACCCCCu<br>5' |||	|| : ||||||| Target:5' ggtcCAG-ACCAGGGCTGGGGGc<br>3'	-20.80	154.00	
		2453~2475	23	miRNA: 3' uuuaGUCUUAGUGUGGACCCCcu<br>5' |:|::	:||||||| Target:5' aacaCGGGGCCTTACCTGGGGaa	-23.50	143.00	

<table>
<tr><td></td><td></td><td></td><td></td><td>3'</td><td></td><td></td><td></td></tr>
<tr><td>241</td><td>hsa-miR-3614-5p</td><td>2138~2161</td><td>24</td><td>miRNA: 3' cccgUCGGAAGU-CUAGGUUCACc 5'
:|:| |: |: |||||||
Target:5' gctgGGTCCCCGTGGCCCAAGTGc 3'</td><td>-17.90</td><td>147.00</td><td>Show result profile</td></tr>
<tr><td>242</td><td>hsa-miR-3615</td><td>130~150</td><td>21</td><td>miRNA: 3' cuCGGCGCUCCUCGGCUCUCu 5'
||:|||: : |||||:|
Target:5' ggGCTGCGGCCGCCCGAGGGa 3'</td><td>-26.60</td><td>143.00</td><td>Show result profile</td></tr>
<tr><td>243</td><td>hsa-miR-3617</td><td>2499~2519</td><td>21</td><td>miRNA: 3' ggguaGAACGUUGAUACAGAAa 5'
|| ||: |||||||
Target:5' gcccgCTCCCAG-AATGTCTTa 3'</td><td>-14.60</td><td>144.00</td><td>Show result profile</td></tr>
<tr><td rowspan="3">244</td><td rowspan="3">hsa-miR-3619</td><td>1541~1562</td><td>22</td><td>miRNA: 3' cgacguGGUCGGACGGACGACu 5'
||:| | ||||||||
Target:5' tcctatCCGGGCAGCCTGCTGg 3'</td><td>-22.90</td><td>160.00</td><td rowspan="3">Show result profile</td></tr>
<tr><td>2092~2114</td><td>23</td><td>miRNA: 3' cgaCGUGGUCGGAC-GGACGACu 5'
|| |||: | |||||||
Target:5' gggGCTGGAGCTGGCCCTGCTGc 3'</td><td>-21.30</td><td>151.00</td></tr>
<tr><td>1469~1488</td><td>20</td><td>miRNA: 3' cgACGUGGUCGGACGGACGACu 5'
||||||:| |||| ||||
Target:5' acTGCACCGG--TGCCAGCTGt 3'</td><td>-27.00</td><td>144.00</td></tr>
<tr><td>245</td><td>hsa-miR-362-5p</td><td>1576~1600</td><td>25</td><td>miRNA: 3' ugAGU-GUGGAUCCAAGGUUCCUAa 5'
||| :: | |||| ||:||||
Target:5' ctTCAGTGGCCAGGTCACAGGGATg 3'</td><td>-16.90</td><td>146.00</td><td>Show result profile</td></tr>
<tr><td>246</td><td>hsa-miR-3620</td><td>1600~1623</td><td>24</td><td>miRNA: 3' gacCCACGCCCUAC--GUCCCACu 5'
| |||| :|| :||||||
Target:5' ggaGCTGCGCTGTGCATAGGGTGc 3'</td><td>-18.30</td><td>141.00</td><td>Show result profile</td></tr>
<tr><td rowspan="2">247</td><td rowspan="2">hsa-miR-3622a-3p</td><td>1609~1635</td><td>27</td><td>miRNA: 3' ugucCGUACCCUCC-----AGUCCACu 5'
|||| ||: | |||||||
Target:5' ctgtGCATAGGGTGCCACCTCAGGTGt 3'</td><td>-17.30</td><td>154.00</td><td rowspan="2">Show result profile</td></tr>
<tr><td>1922~1942</td><td>21</td><td>miRNA: 3' uguCCGUACCCUCCAGUCCACu 5'
||| ||||| ||::||||
Target:5' cctGGC-TGGGAAGTTGGGTGc 3'</td><td>-22.40</td><td>142.00</td></tr>
</table>

<table>
<tr><td>248</td><td>hsa-miR-3622b-3p</td><td>1610~1635</td><td>26</td><td>miRNA: 3' gucCGUG-CCC----UCGAGUCCACu 5'
|||: ||| | ||||||||
Target:5' tgtGCATAGGGTGCCACCTCAGGTGt 3'</td><td>-20.80</td><td>156.00</td><td>Show result profile</td></tr>
<tr><td>249</td><td>hsa-miR-3646</td><td>2057~2084</td><td>28</td><td>miRNA: 3' accCGACCC-----GAGU-AAAGUAAAa 5'
||| || |||| ||||:|||
Target:5' cgaGCTTGGCCCTCCTCAGTTTCGTTTc 3'</td><td>-11.90</td><td>147.00</td><td>Show result profile</td></tr>
<tr><td>250</td><td>hsa-miR-3648</td><td>2131~2154</td><td>24</td><td>miRNA: 3' ggGAGC-CG--CUAGGGGCGCCGa 5'
|| | || |:||||||:|||
Target:5' ggCTGGAGCTGGGTCCCCGTGGCc 3'</td><td>-27.40</td><td>149.00</td><td>Show result profile</td></tr>
<tr><td rowspan="2">251</td><td rowspan="2">hsa-miR-365*</td><td>40~65</td><td>26</td><td>miRNA: 3' ugUCG-ACGGGGACU---UUCAGGGa 5'
||| ||:|| || |||||||
Target:5' ggAGCGGGCTCCGGAGGGAAGTCCCg 3'</td><td>-24.80</td><td>160.00</td><td rowspan="2">Show result profile</td></tr>
<tr><td>919~942</td><td>24</td><td>miRNA: 3' ugucGACGGGGACU--UUCAGGGa 5'
:| : |:||: |||||||
Target:5' acttTTATACTTGGGTAAGTCCCc 3'</td><td>-14.20</td><td>148.00</td></tr>
<tr><td>252</td><td>hsa-miR-3651</td><td>1387~1413</td><td>27</td><td>miRNA: 3' agUAC-AUGGUCGCUG--GCCCGAUAc 5'
:|| | || || | ||||||:|
Target:5' ctGTGCTCCCCGCATCTGCGGGCTGTa 3'</td><td>-16.80</td><td>140.00</td><td>Show result profile</td></tr>
<tr><td rowspan="2">253</td><td rowspan="2">hsa-miR-3652</td><td>2261~2278</td><td>18</td><td>miRNA: 3' aggagugugGAGGUCGGc 5'
||||||||
Target:5' cgtgtgggaCTCCAGCCg 3'</td><td>-18.90</td><td>145.00</td><td rowspan="2">Show result profile</td></tr>
<tr><td>2341~2358</td><td>18</td><td>miRNA: 3' aggaGUGUGGAGGUCGgc 5'
||| ||||||||
Target:5' ccacCACCCCTCCAGCga 3'</td><td>-19.80</td><td>142.00</td></tr>
<tr><td>254</td><td>hsa-miR-3657</td><td>1980~2000</td><td>21</td><td>miRNA: 3' uuagugguuauuaCCCUGUGu 5'
|||||||
Target:5' tgggaagccctggGGGACACa 3'</td><td>-15.50</td><td>140.00</td><td>Show result profile</td></tr>
<tr><td rowspan="2"></td><td rowspan="2"></td><td>1076~1106</td><td>31</td><td>miRNA: 3' guCGAC--AGGCUC--A-----GGGUCCAGu 5'
|||| |::||| | ||||||||
Target:5' gtGCTGCTTTTGAGCCTCAGACCCCAGGTCa 3'</td><td>-22.80</td><td>156.00</td><td rowspan="2"></td></tr>
<tr><td></td><td></td><td>miRNA: 3' guCGACAGG---CUCAG------</td><td></td><td></td></tr>
</table>

<table>
<tr><td rowspan="3">255</td><td rowspan="3">hsa-miR-3661</td><td>1482~1512</td><td>31</td><td>GGUCCAGu 5'
||||||: |:| |
|||||||
Target:5'
caGCTGTCTTGGGGGCCACAAGGCCAGGTCc 3'</td><td>-27.20</td><td>154.00</td><td rowspan="3">Show result profile</td></tr>
<tr><td>1571~1592</td><td>22</td><td>miRNA: 3' gucGACAGGCUCA-GGGUCCAGu
5'
|| |:| ||| |||||||
Target:5' cccCTCTTC-AGTGGCCAGGTCa
3'</td><td>-16.80</td><td>154.00</td></tr>
<tr><td>2240~2261</td><td>22</td><td>miRNA: 3' gucgacaggcucaGGGUCCAGu
5'
:|||||||
Target:5' gagctcggcacccTCCAGGTCc
3'</td><td>-16.60</td><td>141.00</td></tr>
<tr><td rowspan="2">256</td><td rowspan="2">hsa-miR-3663-5p</td><td>1499~1520</td><td>22</td><td>miRNA: 3' ggcUCGUGGUGC--GUCUGGUCg
5'
|| :||| | ||||||||
Target:5' acaAG-GCCAGGTCCAGACCAGg
3'</td><td>-18.30</td><td>155.00</td><td rowspan="2">Show result profile</td></tr>
<tr><td>1265~1286</td><td>22</td><td>miRNA: 3' ggcUCGUGGU-GCGUCUGGUCg
5'
||| ||| |||:|:||||
Target:5' tgcAGCCCCACCGCGGGCCAGg
3'</td><td>-25.20</td><td>142.00</td></tr>
<tr><td>257</td><td>hsa-miR-3663-3p</td><td>1984~2012</td><td>29</td><td>miRNA: 3' cgCGGG----CC-G-
GACACACCACGAGu 5'
|||| || | | |
|||||||
Target:5'
aaGCCCTGGGGGACACAGCCCGGTGCTCc 3'</td><td>-23.70</td><td>143.00</td><td>Show result profile</td></tr>
<tr><td>258</td><td>hsa-miR-3664</td><td>561~584</td><td>24</td><td>miRNA: 3' ugaguACUCAC--UUCUGUCUCAa
5'
| | || || |||||||
Target:5' acgatTTACTGCCAAAACAGAGTg
3'</td><td>-7.70</td><td>151.00</td><td>Show result profile</td></tr>
<tr><td rowspan="3">259</td><td rowspan="3">hsa-miR-3665</td><td>1249~1268</td><td>20</td><td>miRNA: 3' gcGGCGGG--GCGUGGACGa 5'
|| ||| | |||||||
Target:5' gcCCTCCCCACCCACCTGCa 3'</td><td>-21.00</td><td>154.00</td><td rowspan="3">Show result profile</td></tr>
<tr><td>1389~1406</td><td>18</td><td>miRNA: 3' gcgGCGGGGCGUGGACGa 5'
| |||||||:||||
Target:5' gtgCTCCCCGCATCTGCg 3'</td><td>-27.80</td><td>151.00</td></tr>
<tr><td>98~115</td><td>18</td><td>miRNA: 3' gcGGCGGGGCGUGGACGa 5'
| |:||: ||||||:
Target:5' ctCGGTCCTCCACCTGTc 3'</td><td>-18.50</td><td>140.00</td></tr>
<tr><td>260</td><td>hsa-miR-3667-5p</td><td>807~828</td><td>22</td><td>miRNA: 3' ugGAAGAGGAGUUACCCAGAaa
5'
| |:||| :| ||||||
Target:5' ccCGTTTCCATACAGGGTCTct
3'</td><td>-15.00</td><td>140.00</td><td>Show result profile</td></tr>
</table>

<table>
<tr><td rowspan="3">261</td><td rowspan="3">hsa-miR-3667-3p</td><td>646~668</td><td>23</td><td>miRNA: 3' uuuCUG-GGUACCUCUCCUUCCa 5'
                |:| :|:| | |||||||
Target:5' tgtGGCATCGTCAAAAGGAAGGg 3'</td><td>-13.20</td><td>151.00</td><td rowspan="3">Show result profile</td></tr>
<tr><td>1947~1965</td><td>19</td><td>miRNA: 3' uuucuGGGUACCUCUCCUUCCa 5'
                |||| : |||||||
Target:5' cacctCCCA---GCAGGAAGGc 3'</td><td>-15.50</td><td>146.00</td></tr>
<tr><td>182~202</td><td>21</td><td>miRNA: 3' uuucUGGGUACCUCUCCUUCCa 5'
               :|||| ||||||||| |
Target:5' actcGCCCA-GGAGAGGAAAGc 3'</td><td>-21.70</td><td>141.00</td></tr>
<tr><td>262</td><td>hsa-miR-3669</td><td>2589~2614</td><td>26</td><td>miRNA: 3' auAUAAGGCAU--A-UGUAUAAGGCa 5'
            ||||::||| | ||:||||||:|
Target:5' taTATTTTGTAGTTAACGTATTCTGa 3'</td><td>-18.50</td><td>159.00</td><td>Show result profile</td></tr>
<tr><td>263</td><td>hsa-miR-3670</td><td>2395~2419</td><td>25</td><td>miRNA: 3' aucucuuccUGUC-GACACUCGAGa 5'
                   :||| | |||||||
Target:5' acgcaccctGCAGTCACTGAGCTCt 3'</td><td>-14.30</td><td>147.00</td><td>Show result profile</td></tr>
<tr><td>264</td><td>hsa-miR-3673</td><td>274~297</td><td>24</td><td>miRNA: 3' auaaGGCAUAUAU---GUAAGGUa 5'
              |:| : :|: |||||||
Target:5' tgaaCTGGGAGTGCGCCATTCCAg 3'</td><td>-9.20</td><td>141.00</td><td>Show result profile</td></tr>
<tr><td>265</td><td>hsa-miR-3675-5p</td><td>702~724</td><td>23</td><td>miRNA: 3' cuuuagAGAUGUCUUCGGGGUAu 5'
                |||| || ||:||||
Target:5' tgcaaaTCTAAAGTTGCTCCATa 3'</td><td>-16.00</td><td>145.00</td><td>Show result profile</td></tr>
<tr><td>266</td><td>hsa-miR-3678-3p</td><td>1843~1869</td><td>27</td><td>miRNA: 3' ggcCAGGCAUG---UUU--GAGACGUc 5'
             |||| || :|: |||||||
Target:5' gccGTCCTGACGCTGAGCCCTCTGCAa 3'</td><td>-16.90</td><td>149.00</td><td>Show result profile</td></tr>
<tr><td>267</td><td>hsa-miR-3679-5p</td><td>1633~1653</td><td>21</td><td>miRNA: 3' agGGGAAGGGACGGUAUAGGAGu 5'
            :|: ||||| ::|:|||||
Target:5' tgTCTGTCCCT--TGTGTCCTCa 3'</td><td>-19.90</td><td>145.00</td><td>Show result profile</td></tr>
<tr><td>268</td><td>hsa-miR-3680*</td><td>842~865</td><td>24</td><td>miRNA: 3' ggAUGAGGG-UCCCAGUACGUUUu 5'
            ||:|::: | ||:|||:|||
Target:5' tgTATTTTTGATTGTTATGTAAAa 3'</td><td>-10.60</td><td>145.00</td><td>Show result profile</td></tr>
</table>

<table>
<tr><td rowspan="2">269</td><td rowspan="2">hsa-miR-3681*</td><td>2806~2827</td><td>22</td><td>miRNA: 3' ucaUCACCUACUUCGUGACAca
5'
| ||||| || ||||||
Target:5' ggcAATGGATTAACCACTGTat
3'</td><td>-12.00</td><td>151.00</td><td rowspan="2">Show result profile</td></tr>
<tr><td>1591~1614</td><td>24</td><td>miRNA: 3' ucaUCACCUACUU---
CGUGACACa 5'
|| |||||:| ||:|||||
Target:5' cacAG-
GGATGGAGCTGCGCTGTGc 3'</td><td>-20.90</td><td>150.00</td></tr>
<tr><td>270</td><td>hsa-miR-3688</td><td>792~818</td><td>27</td><td>miRNA: 3' ucUCACCG-UUUCA---G-
AAAGGUAu 5'
| | || :|| | |
|||||||
Target:5'
cgATTCGCTGAATTGCCCGTTTCCATa 3'</td><td>-9.20</td><td>144.00</td><td>Show result profile</td></tr>
<tr><td rowspan="2">271</td><td rowspan="2">hsa-miR-3689a-3p</td><td>1933~1956</td><td>24</td><td>miRNA: 3' uggugCUAUAGU--GUGGAGGGUc
5'
|:|: |: |||||||||
Target:5' agttgGGTGCCGGTCACCTCCCAg
3'</td><td>-23.30</td><td>155.00</td><td rowspan="2">Show result profile</td></tr>
<tr><td>1221~1242</td><td>22</td><td>miRNA: 3' uggugcuauagugUGGAGGGUc
5'
:|||||||
Target:5' gtgagaggaggcgGCCTCCCAg
3'</td><td>-18.10</td><td>141.00</td></tr>
<tr><td rowspan="2">272</td><td rowspan="2">hsa-miR-3689b*</td><td>1933~1956</td><td>24</td><td>miRNA: 3' uggugUUAUAGU--GUGGAGGGUc
5'
::|: |: |||||||||
Target:5' agttgGGTGCCGGTCACCTCCCAg
3'</td><td>-21.00</td><td>151.00</td><td rowspan="2">Show result profile</td></tr>
<tr><td>1221~1242</td><td>22</td><td>miRNA: 3' ugguguuauagugUGGAGGGUc
5'
:|||||||
Target:5' gtgagaggaggcgGCCTCCCAg
3'</td><td>-17.20</td><td>141.00</td></tr>
<tr><td>273</td><td>hsa-miR-3690</td><td>2205~2227</td><td>23</td><td>miRNA: 3' gaaacagaugcgacCCAGGUCCa
5'
||||||||
Target:5' aatgcagggagggcGGTCCAGGg
3'</td><td>-15.51</td><td>145.00</td><td>Show result profile</td></tr>
<tr><td>274</td><td>hsa-miR-3692*</td><td>1937~1960</td><td>24</td><td>miRNA: 3' guCAUAGGU--
GAGGACUGGUCGUCc 5'
||: ||: | |||
|||||||
Target:5' ggGTG-CCGGTCACCT-
CCCAGCAGg 3'</td><td>-23.50</td><td>150.00</td><td>Show result profile</td></tr>
<tr><td>275</td><td>hsa-miR-3692</td><td>2248~2270</td><td>23</td><td>miRNA: 3' ugaaGACGUCACAGUCACACCUUg
5'
|| ||| ||| ||||||:|
Target:5' caccCTCCAG-GTCCGTGTGGGAc
3'</td><td>-22.40</td><td>155.00</td><td>Show result profile</td></tr>
</table>

<table>
<tr><td rowspan="3">276</td><td rowspan="3">hsa-miR-370</td><td>1940~1961</td><td>22</td><td>miRNA: 3' ugGUCCAAGGUGGGGUCGUCCg 5'
| ||| | :|||||||||
Target:5' tgCCGGTCACCTCCCAGCAGGa 3'</td><td>-28.00</td><td>164.00</td><td rowspan="3">Show result profile</td></tr>
<tr><td>161~183</td><td>23</td><td>miRNA: 3' ugGUCCAAGGUGGG-GUCGUCcg 5'
|:|| :|:||| ||||||
Target:5' gtCGGGGATCGCCCTCAGCAGac 3'</td><td>-27.60</td><td>144.00</td></tr>
<tr><td>1822~1845</td><td>24</td><td>miRNA: 3' uggUCC-A-AGGUGGGGUCGUCcg 5'
||| | | |:||:||||||
Target:5' gggAGGCTGTGCGCCTCAGCAGcc 3'</td><td>-23.20</td><td>143.00</td></tr>
<tr><td rowspan="2">277</td><td rowspan="2">hsa-miR-3714</td><td>1065~1086</td><td>22</td><td>miRNA: 3' uguccсCUCGUGACGACGGAAg 5'
|:|| ||||||:||
Target:5' gaacctGGGCTGTGCTGCTTTt 3'</td><td>-16.30</td><td>144.00</td><td rowspan="2">Show result profile</td></tr>
<tr><td>1472~1492</td><td>21</td><td>miRNA: 3' uguccCCUCGUGACGACGGAAg 5'
|| || | ||||:|||
Target:5' gcaccGGTGC-CAGCTGTCTTg 3'</td><td>-14.30</td><td>140.00</td></tr>
<tr><td>278</td><td>hsa-miR-373*</td><td>1071~1090</td><td>20</td><td>miRNA: 3' ccuuuCGCGGGGGUAAAACUCa 5'
|:||: | |||||||
Target:5' gggctGTGCTGC--TTTTGAGc 3'</td><td>-14.40</td><td>149.00</td><td>Show result profile</td></tr>
<tr><td>279</td><td>hsa-miR-373</td><td>2831~2853</td><td>23</td><td>miRNA: 3' ugUGGGGUUUUAGCUUCGUGAAg 5'
| :||| ||: |||||:||
Target:5' agAATCCACCATTAAAGCATTTg 3'</td><td>-10.80</td><td>149.00</td><td>Show result profile</td></tr>
<tr><td>280</td><td>hsa-miR-376c</td><td>1140~1160</td><td>21</td><td>miRNA: 3' ugcACCUUAAAGGAGAUACAa 5'
||| :|||: |||:|||
Target:5' ataTGGCGTTTTGTCTGTGTt 3'</td><td>-15.10</td><td>150.00</td><td>Show result profile</td></tr>
<tr><td>281</td><td>hsa-miR-377*</td><td>2004~2028</td><td>25</td><td>miRNA: 3' cuUAAGUGGUUC---CCGUUGGAGa 5'
:| | || || |||||||
Target:5' cgGTGCTCCCAGCCCTCCAACCTCt 3'</td><td>-14.00</td><td>144.00</td><td>Show result profile</td></tr>
<tr><td>282</td><td>hsa-miR-378*</td><td>1425~1449</td><td>25</td><td>miRNA: 3' uguGUCCUGGACCU---CAGUCCUc 5'
:| ||:||| | |||:|||
Target:5' agcTAAGATCTGCATAGGTCGGGAt 3'</td><td>-15.90</td><td>143.00</td><td>Show result profile</td></tr>
<tr><td></td><td>hsa-</td><td></td><td></td><td>miRNA: 3' ugUCUCUCGAACGGGAACAUAu 5'</td><td></td><td></td><td></td></tr>
</table>

<table>
<tr><th></th><th></th><th></th><th></th><th></th><th></th><th></th><th></th></tr>
<tr><td>283</td><td>miR-381</td><td>1628~1649</td><td>22</td><td>||: : || |||||||:|
Target:5' tcAGGTGTCTGTCCCTTGTGTc 3'</td><td>-16.80</td><td>144.00</td><td>Show result profile</td></tr>
<tr><td rowspan="2">284</td><td rowspan="2">hsa-miR-3909</td><td>2155~2178</td><td>24</td><td>miRNA: 3' ucUGACGUCC--GGGAUCUCCUGu 5'
| |||||| ||| |||||:|
Target:5' caAGTGCAGGGTCCCAAGAGGGCa 3'</td><td>-26.70</td><td>158.00</td><td rowspan="2">Show result profile</td></tr>
<tr><td>1977~1998</td><td>22</td><td>miRNA: 3' ucUGACGUCCGGGAUCUCCUGu 5'
|: | | |||||:|:||||
Target:5' agATGGGAAGCCCTGGGGGACa 3'</td><td>-26.20</td><td>140.00</td></tr>
<tr><td rowspan="4">285</td><td rowspan="4">hsa-miR-3913</td><td>2149~2172</td><td>24</td><td>miRNA: 3' ucuguaGUUCUAGU--CAGGGUUu 5'
|||| || |||||||
Target:5' gtggccCAAGTGCAGGGTCCCAAg 3'</td><td>-15.00</td><td>154.00</td><td rowspan="4">Show result profile</td></tr>
<tr><td>224~244</td><td>21</td><td>miRNA: 3' ucuguAGUUCUAGUCAGGGUUu 5'
||: |: | |||||||
Target:5' tggttTCGTGG-CTGTCCCAAc 3'</td><td>-12.30</td><td>148.00</td></tr>
<tr><td>2149~2172</td><td>24</td><td>miRNA: 3' ucuguaGUUCUAGU--CAGGGUUu 5'
|||| || |||||||
Target:5' gtggccCAAGTGCAGGGTCCCAAg 3'</td><td>-15.00</td><td>154.00</td></tr>
<tr><td>224~244</td><td>21</td><td>miRNA: 3' ucuguAGUUCUAGUCAGGGUUu 5'
||: |: | |||||||
Target:5' tggttTCGTGG-CTGTCCCAAc 3'</td><td>-12.30</td><td>148.00</td></tr>
<tr><td>286</td><td>hsa-miR-3917</td><td>588~607</td><td>20</td><td>miRNA: 3' ggguggacgagUCAGGCUCg 5'
:|||||||
Target:5' tacgagaaaagGGTCCGAGc 3'</td><td>-15.84</td><td>141.00</td><td>Show result profile</td></tr>
<tr><td rowspan="3">287</td><td rowspan="3">hsa-miR-3918</td><td>2093~2111</td><td>19</td><td>miRNA: 3' ucaGAGGUAGACGCCGGGACa 5'
|| | || |||||||
Target:5' gggCTGGAGCT--GGCCCTGc 3'</td><td>-16.90</td><td>146.00</td><td rowspan="3">Show result profile</td></tr>
<tr><td>2217~2233</td><td>17</td><td>miRNA: 3' ucagAGGUAGACGCCGGGACa 5'
|||| | |||||||
Target:5' gcggTCCA---G-GGCCCTGg 3'</td><td>-16.50</td><td>145.00</td></tr>
<tr><td>1744~1766</td><td>23</td><td>miRNA: 3' ucaGAGGUAGA-C-GCCGGGACa 5'
| ||| | | |||||||
Target:5' tagCGCCAGGTAGAGGGCCCTGg 3'</td><td>-16.30</td><td>142.00</td></tr>
</table>

<table>
<tr><td rowspan="2">288</td><td rowspan="2">hsa-miR-3922</td><td>1491~1509</td><td>19</td><td>miRNA: 3' uuUCUCAGUUCAGUUCCGGUCu 5'
:|:| || |||||||||
Target:5' tgGGGGCCA---CAAGGCCAGg 3'</td><td>-19.60</td><td>157.00</td><td rowspan="2">Show result profile</td></tr>
<tr><td>450~475</td><td>26</td><td>miRNA: 3' uuUCU-CAGUUC---AGUUCCGGUCu 5'
:|| | |||| |
:|||||||
Target:5' gaGGAGGACAAGGACTGGAGGCCAGc 3'</td><td>-20.00</td><td>156.00</td></tr>
<tr><td>289</td><td>hsa-miR-3924</td><td>2544~2578</td><td>35</td><td>miRNA: 3' ucAUCGUCA--GUGUA-----------UAUGUAUa 5'
|| :||| |||||
|||||||
Target:5' agTAATAGTTACACATGTATATGGTTAATACATAt 3'</td><td>-16.15</td><td>150.00</td><td>Show result profile</td></tr>
<tr><td rowspan="2">290</td><td rowspan="2">hsa-miR-3928</td><td>931~959</td><td>29</td><td>miRNA: 3' cggcUUC--GAGGUUC-----CAAGGAGg 5'
||| | |||:|
|||||||
Target:5' gggtAAGTCCCCCAGGGGCGAGTTCCTCg 3'</td><td>-17.40</td><td>152.00</td><td rowspan="2">Show result profile</td></tr>
<tr><td>2610~2632</td><td>23</td><td>miRNA: 3' cgGCUUCG-AGGUUCCAAGGAGg 5'
:||||: | :| |||:|||
Target:5' tcTGAAGTAACGGATGTTTCTCg 3'</td><td>-16.20</td><td>140.00</td></tr>
<tr><td rowspan="2">291</td><td rowspan="2">hsa-miR-3937</td><td>1513~1536</td><td>24</td><td>miRNA: 3' gggGGUAACGA-UGUCGGCGGACa 5'
||| ||| : :||:|||||
Target:5' agaCCAGGGCTGGGGGCTGCCTGa 3'</td><td>-24.20</td><td>144.00</td><td rowspan="2">Show result profile</td></tr>
<tr><td>124~146</td><td>23</td><td>miRNA: 3' gggGGUAACGAUGUCGGCGGACa 5'
||: |||:|:|||||| |
Target:5' tcgCCGGGGCTGCGGCCGCCCGa 3'</td><td>-31.12</td><td>140.00</td></tr>
<tr><td>292</td><td>hsa-miR-3939</td><td>20~40</td><td>21</td><td>miRNA: 3' cuguAGGACACCAGACGCGCau 5'
| || ||| |||||||
Target:5' gagcTGCTCTGG-CTGCGCGcg 3'</td><td>-17.80</td><td>141.00</td><td>Show result profile</td></tr>
<tr><td>293</td><td>hsa-miR-3940</td><td>1533~1554</td><td>22</td><td>miRNA: 3' uuCACCCGACCCUAGGCCCGac 5'
| || || ||||||||
Target:5' ctGAGGACTCCTATCCGGGCag 3'</td><td>-17.50</td><td>140.00</td><td>Show result profile</td></tr>
<tr><td></td><td></td><td>1509~1531</td><td>23</td><td>miRNA: 3' gcGGUUCACUUCGGACCCCCGAu 5'
|||: |:| |||||||||
Target:5' gtCCAGACCAGGGCTGGGGGCTg 3'</td><td>-27.50</td><td>165.00</td><td></td></tr>
</table>

294	hsa-miR-3943	1479~1499	21	miRNA: 3' gcGGUUCACUUCGGACCCCCGau<br>5'<br>|||: || |:||||||| Target:5' tgCCAGCTG--TCTTGGGGGCca<br>3'	-25.20	141.00	Show result profile
		2076~2098	23	miRNA: 3' gcGGUUCACUUCGGACCCCCGAu<br>5'<br>:|: | |||||| |||||| Target:5' ttTCGTTTCAAGCCTTGGGGCTg<br>3'	-22.90	141.00	
295	hsa-miR-3944	127~147	21	miRNA: 3' ggCCUCGUCGUCCGGUCGGGCUu<br>5'<br>||:|| ||:| | |||||| Target:5' ccGGGGCTGCGGCC--GCCCGAg<br>3'	-37.50	140.00	Show result profile
296	hsa-miR-409-3p	669~700	32	miRNA: 3' uccCCAAGUGGCUC----------<br>GUUGUAAg 5'<br>||||:: | || ||||||| Target:5'<br>attGGTTTGGCAAGAACTTGTTTACAACATTt 3'	-10.50	145.00	Show result profile
297	hsa-miR-423-5p	1027~1051	25	miRNA: 3' uuucaGAGCGA-GA-<br>GACGGGGAGu 5'<br>||| || || | ||:|||| Target:5'<br>gcaccCTCCCTCCTGCCGCTCCTCt 3'	-19.80	142.00	Show result profile
298	hsa-miR-424	1061~1084	24	miRNA: 3' aaGUUUUGUACUU-A-ACGACGAc<br>5'<br>:|:||| ||:: | ||||||| Target:5' tcTAGAACCTGGGCTGTGCTGCTt<br>3'	-15.10	160.00	Show result profile
		1354~1380	27	miRNA: 3' aaGUUUUG-UAC----<br>UUAACGACGAc 5'<br>||:::| :|| || ||||||| Target:5'<br>aaCAGGGCTGTGTACAAAGTGCTGCTg 3'	-18.20	154.00	
299	hsa-miR-4252	1568~1586	19	miRNA: 3' accacgacuGAGUCACCGg 5'<br>:|||||||| Target:5' gttcccctcTTCAGTGGCc 3'	-16.60	146.00	Show result profile
300	hsa-miR-4254	2232~2259	28	miRNA: 3' cuCUACCACCUC-AUC-----<br>GAGGUCCg 5'<br>|| || |||| | | ||||||| Target:5' ggGAAGG-<br>GGAGCTCGGCACCCTCCAGGt 3'	-22.10	152.00	Show result profile
		1706~1730	25	miRNA: 3' cucuaccaccuCAUC--<br>GAGGUCCg 5'<br>||:| ||||||| Target:5'<br>agcccacagccGTGGCCCTCCAGGg 3'	-16.20	146.00	

<table>
<tr><td>301</td><td>hsa-miR-4257</td><td>2011~2030</td><td>20</td><td>miRNA: 3' gaGUCAGGGG--UGGAGACc 5'
            ||| ||:| |||||||
Target:5' ccCAGCCCTCCAACCTCTGg 3'</td><td>-23.20</td><td>158.00</td><td>Show result profile</td></tr>
<tr><td>302</td><td>hsa-miR-4258</td><td>1550~1567</td><td>18</td><td>miRNA: 3' gguUCCG-CCACCGCCCc 5'
             || | | |||||||
Target:5' ggcAGCCTGCTGGCGGGg 3'</td><td>-20.70</td><td>146.00</td><td>Show result profile</td></tr>
<tr><td rowspan="2">303</td><td rowspan="2">hsa-miR-4259</td><td>525~546</td><td>22</td><td>miRNA: 3' aggacugGGGAUCUGGGUUGAc
5'
                 :|| |||||||:||
Target:5' ccaaataTCCAAGACCCAGCTc
3'</td><td>-19.60</td><td>147.00</td><td rowspan="2">Show result profile</td></tr>
<tr><td>2018~2040</td><td>23</td><td>miRNA: 3' aggacUGGGGAUC-UGGGUUGac
5'
               |||:||:| ||||||
Target:5' ctccaACCTCTGGCTCCCAACcc
3'</td><td>-21.30</td><td>141.00</td></tr>
<tr><td rowspan="2">304</td><td rowspan="2">hsa-miR-4265</td><td>1697~1714</td><td>18</td><td>miRNA: 3' ggGUCUCGACUCGGGUGUc 5'
            | |:| |||||||||
Target:5' ttCTGGG-AGAGCCCACAg 3'</td><td>-23.30</td><td>152.00</td><td rowspan="2">Show result profile</td></tr>
<tr><td>544~562</td><td>19</td><td>miRNA: 3' ggGUCUCGACU-CGGGUGUc 5'
            || ||| || |||:|||
Target:5' ctCA-AGCAGAGGCCTACAc 3'</td><td>-14.20</td><td>140.00</td></tr>
<tr><td rowspan="2">305</td><td rowspan="2">hsa-miR-4267</td><td>2093~2106</td><td>14</td><td>miRNA: 3' caCGGUGGCUCGACCu 5'
            ||:: |||||||
Target:5' ggGCTG--GAGCTGGc 3'</td><td>-12.10</td><td>142.00</td><td rowspan="2">Show result profile</td></tr>
<tr><td>2127~2143</td><td>17</td><td>miRNA: 3' caCGGUGG-CUCGACCu 5'
            |: :|: |||||||
Target:5' cgGTGGCTGGAGCTGGg 3'</td><td>-17.00</td><td>142.00</td></tr>
<tr><td>306</td><td>hsa-miR-4268</td><td>2224~2244</td><td>21</td><td>miRNA: 3' guguaGGACUCUCCUCCUCGg 5'
               ||||:|| |:|||||
Target:5' agggcCCTGGGAAGGGGAGCt 3'</td><td>-23.20</td><td>152.00</td><td>Show result profile</td></tr>
<tr><td rowspan="2">307</td><td rowspan="2">hsa-miR-4269</td><td>422~441</td><td>20</td><td>miRNA: 3' cgGUCCC-GACAGACACGGACg
5'
            |:||| | | ||||||||
Target:5' ttCGGGGACAG--TGTGCCTGt
3'</td><td>-22.70</td><td>155.00</td><td rowspan="2">Show result profile</td></tr>
<tr><td>1761~1781</td><td>21</td><td>miRNA: 3' cgGUCCCGACAGACACGGACg 5'
            | |||| | |||||:|||
Target:5' ccCTGGGCAGCCTGTGTCTGg 3'</td><td>-25.10</td><td>155.00</td></tr>
<tr><td>308</td><td>hsa-miR-4271</td><td>1720~1738</td><td>19</td><td>miRNA: 3' ggGGUGGAAAAGAAGGGGg 5'
            || || |||||||
Target:5' gcCCTCCAGGGCTTCCCCg 3'</td><td>-21.00</td><td>145.00</td><td>Show result profile</td></tr>
</table>

<table>
<tr><td>309</td><td>hsa-miR-4273</td><td>438~460</td><td>23</td><td>miRNA: 3' gaCAGGUAG-----UCUCUUGUg 5'
||||||| :|||:|||
Target:5' ctGTCCATCTTAGAGGAGGACAa 3'</td><td>-21.20</td><td>144.00</td><td>Show result profile</td></tr>
<tr><td>310</td><td>hsa-miR-4276</td><td>2398~2415</td><td>18</td><td>miRNA: 3' cguGUAC-UCAGUGACUc 5'
| || |||||||||
Target:5' cacCCTGCAGTCACTGAg 3'</td><td>-17.80</td><td>154.00</td><td>Show result profile</td></tr>
<tr><td>311</td><td>hsa-miR-4277</td><td>980~998</td><td>19</td><td>miRNA: 3' cacaUGACACGAGUCUUGACg 5'
|| ||| ||||:|||
Target:5' tctcACCGTG--CAGAGCTGc 3'</td><td>-15.40</td><td>141.00</td><td>Show result profile</td></tr>
<tr><td>312</td><td>hsa-miR-4279</td><td>2707~2722</td><td>16</td><td>miRNA: 3' cuucggccCUCCUCUc 5'
|||||||
Target:5' ccttgtaaGAGGAGAg 3'</td><td>-13.90</td><td>140.00</td><td>Show result profile</td></tr>
<tr><td rowspan="2">313</td><td rowspan="2">hsa-miR-4280</td><td>982~1002</td><td>21</td><td>miRNA: 3' cgaGACGAGUCUUGAUGUGAg 5'
| |: ||||:||:||||
Target:5' tcaCCGTGCAGAGCTGCACTt 3'</td><td>-18.20</td><td>150.00</td><td rowspan="2">Show result profile</td></tr>
<tr><td>2519~2543</td><td>25</td><td>miRNA: 3' cgAGACG--A--GUCUUGAUGUGAg 5'
|:||: | | ||||||||:|
Target:5' atTTTGTAATGACTGAACTACATTt 3'</td><td>-13.50</td><td>143.00</td></tr>
<tr><td rowspan="2">314</td><td rowspan="2">hsa-miR-4283</td><td>1848~1864</td><td>17</td><td>miRNA: 3' uuugaGCGACUCGGGGu 5'
||||||||||:
Target:5' cctgaCGCTGAGCCCTc 3'</td><td>-21.90</td><td>144.00</td><td rowspan="2">Show result profile</td></tr>
<tr><td>1848~1864</td><td>17</td><td>miRNA: 3' uuugaGCGACUCGGGGu 5'
||||||||||:
Target:5' cctgaCGCTGAGCCCTc 3'</td><td>-21.90</td><td>144.00</td></tr>
<tr><td>315</td><td>hsa-miR-4285</td><td>109~130</td><td>22</td><td>miRNA: 3' uacuCAGCC----UGAGCGGCg 5'
||| | :|||||||
Target:5' acctGTCCGCTACGCTCGCCGg 3'</td><td>-14.70</td><td>144.00</td><td>Show result profile</td></tr>
<tr><td>316</td><td>hsa-miR-4287</td><td>2222~2243</td><td>22</td><td>miRNA: 3' uuUCACGGGA---GUUCCCUCu 5'
|| ||||| |:|||||
Target:5' ccAGGGCCCTGGGAAGGGGAGc 3'</td><td>-19.20</td><td>141.00</td><td>Show result profile</td></tr>
<tr><td>317</td><td>hsa-miR-4288</td><td>169~185</td><td>17</td><td>miRNA: 3' ccuuuGAGUCGUCUGuu 5'
||||||||||
Target:5' tcgccCTCAGCAGACtc 3'</td><td>-16.00</td><td>140.00</td><td>Show result profile</td></tr>
<tr><td>318</td><td>hsa-miR-429</td><td>872~898</td><td>27</td><td>miRNA: 3' ugccAAAAUGGU--CU---GUCAUAAu 5'
||||| :| || |||||||
Target:5' tttaTTTTAATATTGATGTCAGTATTt 3'</td><td>-7.70</td><td>156.00</td><td>Show result profile</td></tr>
</table>

<table>
<tr><td>319</td><td>hsa-miR-4290</td><td>2200~2219</td><td>20</td><td>miRNA: 3' cuccCUU-CUUUCCUCCCGu 5'
    ||| | |:|||||||
Target:5' caaaGAATGCAGGGAGGGCg 3'</td><td>-18.40</td><td>155.00</td><td>Show result profile</td></tr>
<tr><td>320</td><td>hsa-miR-4291</td><td>1367~1382</td><td>16</td><td>miRNA: 3' ucgacaAGGACGACUu 5'
      | |||||||
Target:5' acaaagTGCTGCTGAg 3'</td><td>-11.50</td><td>142.00</td><td>Show result profile</td></tr>
<tr><td rowspan="2">321</td><td rowspan="2">hsa-miR-4292</td><td>933~948</td><td>16</td><td>miRNA: 3' ggUUCCGGCCGGGUCCCc 5'
  ||| || |||||||
Target:5' gtAAGTCC--CCCAGGGg 3'</td><td>-22.20</td><td>152.00</td><td rowspan="2">Show result profile</td></tr>
<tr><td>2211~2228</td><td>18</td><td>miRNA: 3' ggUUCCGGCCGGGUCCCc 5'
  :||| |||:||||||
Target:5' ggGAGGGCGGTCCAGGGc 3'</td><td>-27.40</td><td>152.00</td></tr>
<tr><td>322</td><td>hsa-miR-4294</td><td>1527~1544</td><td>18</td><td>miRNA: 3' ggGAC-GACAUCUGAGGg 5'
  ||| ||| :||||||
Target:5' ggCTGCCTGAGGACTCCt 3'</td><td>-17.90</td><td>143.00</td><td>Show result profile</td></tr>
<tr><td>323</td><td>hsa-miR-4296</td><td>1698~1714</td><td>17</td><td>miRNA: 3' acucggaCUCGGGUGUa 5'
       |||||||||
Target:5' tctgggaGAGCCCACAg 3'</td><td>-18.00</td><td>150.00</td><td>Show result profile</td></tr>
<tr><td>324</td><td>hsa-miR-4297</td><td>1952~1966</td><td>15</td><td>miRNA: 3' guGUCUGUCCUUCCGu 5'
  ||| |||||||||
Target:5' ccCAG-CAGGAAGGCa 3'</td><td>-20.00</td><td>157.00</td><td>Show result profile</td></tr>
<tr><td rowspan="2">325</td><td rowspan="2">hsa-miR-4298</td><td>224~243</td><td>20</td><td>miRNA: 3' gaCGGAGGAGGAGGACAGGGUc
5'
  |::|| | ||||||||
Target:5' tgGTTTCGT--GGCTGTCCCAa
3'</td><td>-16.70</td><td>148.00</td><td rowspan="2">Show result profile</td></tr>
<tr><td>1620~1644</td><td>25</td><td>miRNA: 3' gaCGGAGGAG---
GAGGACAGGGuc 5'
  ||| |||| : :|||||||
Target:5'
gtGCCACCTCAGGTGTCTGTCCCtt 3'</td><td>-26.10</td><td>144.00</td></tr>
<tr><td>326</td><td>hsa-miR-4304</td><td>149~165</td><td>17</td><td>miRNA: 3' acGGGACCUGUACGGCc 5'
  |::|| |||||:||
Target:5' gaCTTTGAACATGTCGg 3'</td><td>-20.30</td><td>143.00</td><td>Show result profile</td></tr>
<tr><td rowspan="2">327</td><td rowspan="2">hsa-miR-4308</td><td>2211~2228</td><td>18</td><td>miRNA: 3' uuCUUCUUUGAGGUCCCu 5'
  ||:|: : |||||||
Target:5' ggGAGGGCGGTCCAGGGc 3'</td><td>-19.30</td><td>152.00</td><td rowspan="2">Show result profile</td></tr>
<tr><td>1714~1731</td><td>18</td><td>miRNA: 3' uucuucuuuGAGGUCCCu 5'
         ||||||||
Target:5' gccgtggccCTCCAGGGc 3'</td><td>-14.80</td><td>145.00</td></tr>
</table>

<table>
<tr><td rowspan="2">328</td><td rowspan="2">hsa-miR-4309</td><td>2254~2275</td><td>22</td><td>miRNA: 3' accUUAGG---A-UCUGAGGUc 5'
::||| | :|||||||
Target:5' ccaGGTCCGTGTGGGACTCCAg 3'</td><td>-18.60</td><td>143.00</td><td rowspan="2">Show result profile</td></tr>
<tr><td>2632~2651</td><td>20</td><td>miRNA: 3' accUUAGGAU--CUGAGGUc 5'
|||| || ||||:||
Target:5' gccAATCGTAGTGACTTCAg 3'</td><td>-13.10</td><td>141.00</td></tr>
<tr><td>329</td><td>hsa-miR-431*</td><td>622~646</td><td>25</td><td>miRNA: 3' ucuuCGGGA-CGUUC--UGCUGGAc 5'
||||| :||| :||||||
Target:5' ttgcGCCCTCATAAGCAGCGACCTt 3'</td><td>-18.10</td><td>144.00</td><td>Show result profile</td></tr>
<tr><td>330</td><td>hsa-miR-4312</td><td>1491~1506</td><td>16</td><td>miRNA: 3' acCCCUGUCCUUGUUCCGg 5'
|||:| |||||||
Target:5' tgGGGGC---CACAAGGCc 3'</td><td>-23.20</td><td>146.00</td><td>Show result profile</td></tr>
<tr><td>331</td><td>hsa-miR-4314</td><td>2496~2512</td><td>17</td><td>miRNA: 3' gacaGGGUAAAGGGUCUc 5'
|||: |||||||
Target:5' gtagCCCG-CTCCCAGAa 3'</td><td>-16.40</td><td>145.00</td><td>Show result profile</td></tr>
<tr><td>332</td><td>hsa-miR-4317</td><td>2797~2813</td><td>17</td><td>miRNA: 3' uuUGAGGGACCGUUACa 5'
| |: : |||||||
Target:5' agAATTGTGGGCAATGg 3'</td><td>-10.20</td><td>143.00</td><td>Show result profile</td></tr>
<tr><td>333</td><td>hsa-miR-432</td><td>1705~1730</td><td>26</td><td>miRNA: 3' ggUGGGU-----UACUGGAUGAGGUUCu 5'
:|||| :||:|| |||||:|
Target:5' gaGCCCACAGCCGTGGCC--CTCCAGGg 3'</td><td>-24.10</td><td>141.00</td><td>Show result profile</td></tr>
<tr><td rowspan="2">334</td><td rowspan="2">hsa-miR-432*</td><td>199~222</td><td>24</td><td>miRNA: 3' ucUGUACCUCC--UC-GGUAGGUc 5'
:|||||||| || ||| |||
Target:5' aaGCATGGAGGAAAGACCACCCAt 3'</td><td>-20.20</td><td>141.00</td><td rowspan="2">Show result profile</td></tr>
<tr><td>461~483</td><td>23</td><td>miRNA: 3' ucUGUACCUCC--UCGGUAG-GUc 5'
|| |||||| ||||||| ||
Target:5' ggAC-TGGAGGCCAGCCATCACAa 3'</td><td>-23.44</td><td>140.00</td></tr>
<tr><td>335</td><td>hsa-miR-4320</td><td>2822~2838</td><td>17</td><td>miRNA: 3' ucCUUCGAUGUCUUAGGg 5'
| | ||| |||||||
Target:5' ctGTATCTA-AGAATCCa 3'</td><td>-10.20</td><td>151.00</td><td>Show result profile</td></tr>
<tr><td>336</td><td>hsa-miR-4322</td><td>1684~1714</td><td>31</td><td>miRNA: 3' ggGGUGCGCGA----------CUCGGGUGUc 5'
||| |:||| |||||||||
Target:5' cgCCAGGTGCTTTTTCTGGGAGAGCCCACAg 3'</td><td>-29.60</td><td>157.00</td><td>Show result profile</td></tr>
</table>

337	hsa-miR-4323	2082~2098	17	miRNA: 3' agacUCCGACACCCCGAc 5'<br>|| || ||||||| Target:5' ttcaAGCCT-TGGGGCTg 3'	-16.80	149.00	Show result profile
		1880~1899	20	miRNA: 3' agacUCC-GA-CACCCCGAc 5'<br>||| || ||||||| Target:5' ggccAGGCCTCTTGGGGCTg 3'	-19.30	146.00	
338	hsa-miR-4325	2146~2163	18	miRNA: 3' agugACUCUGUUCACGUu 5'<br>||: |||||||| Target:5' cccgTGGCCCAAGTGCAg 3'	-13.00	150.00	Show result profile
339	hsa-miR-449a	548~574	27	miRNA: 3' ugGUC----GAU-<br>UGUUAUGUGACGGu 5'<br>||| ||| ||:|| :|||||| Target:5'<br>agCAGAGGCCTACACGATTTACTGCCa 3'	-19.80	150.00	Show result profile
340	hsa-miR-449b	548~574	27	miRNA: 3' cgGUC----GAU-<br>UGUUAUGUGACGGa 5'<br>||| ||| ||:|| :|||||| Target:5'<br>agCAGAGGCCTACACGATTTACTGCCa 3'	-17.50	150.00	Show result profile
341	hsa-miR-449c	551~575	25	miRNA: 3' ugUCGGCGAU-<br>CGUUAUGUGACGGau 5'<br>|| | ||| |:|| :|||||| Target:5' agAGGC-<br>CTACACGATTTACTGCCaa 3'	-15.80	142.00	Show result profile
342	hsa-miR-450b-5p	687~708	22	miRNA: 3' auAAGUCCUUGU-AUAACGUUUu<br>5'<br>||:| |||| | ||||||| Target:5' tgTTTA-CAACATTTTTGCAAAt<br>3'	-8.50	159.00	Show result profile
343	hsa-miR-450b-3p	478~498	21	miRNA: 3' auaccUACGUUUUACUAGGGUu<br>5'<br>|| |||| |||||:| Target:5' tcacaAT-CAAACAGATCCTAt<br>3'	-8.90	140.00	Show result profile
344	hsa-miR-452	1339~1360	22	miRNA: 3' agucaaaGGAGACGUUUGUCaa<br>5'<br>:||:||:|||||| Target:5' ccctggaTCTTTGTAAACAGgg<br>3'	-15.20	143.00	Show result profile
345	hsa-miR-485-5p	1644~1666	23	miRNA: 3' cuuaAGUAGUGC-CGGUCGGAGa<br>5'<br>|| ||| | | ||||||:<br>Target:5' tgtgTCCTCAGGAGGCAGCCTTg<br>3'	-14.90	142.00	Show result profile
		1537~1559	23	miRNA: 3' cuUAAG--UAGUGCCGGUCGGAga<br>5'<br>| || ||| ||| |||||| Target:5' ggACTCCTATC-CGGGCAGCCTgc	-19.00	141.00	

<table>
<tr><td></td><td></td><td></td><td></td><td>3'</td><td></td><td></td><td></td></tr>
<tr><td>346</td><td>hsa-miR-486-5p</td><td>2146~2165</td><td>20</td><td>miRNA: 3' gaGCCCCGUCGAGUCAUGUCCu 5'
|| ||| | |||:||||
Target:5' ccCGTGGC--CCAAGTGCAGGg 3'</td><td>-18.40</td><td>140.00</td><td>Show result profile</td></tr>
<tr><td>347</td><td>hsa-miR-486-3p</td><td>2093~2118</td><td>26</td><td>miRNA: 3' uagGACAU-GACU----CGACGGGGc 5'
||| | |||: |||||||:
Target:5' gggCTGGAGCTGGCCCTGCTGCCCTg 3'</td><td>-23.60</td><td>140.00</td><td>Show result profile</td></tr>
<tr><td>348</td><td>hsa-miR-489</td><td>873~893</td><td>21</td><td>miRNA: 3' cgacggcaUAUACACUACAGUg 5'
|||| ||||||||
Target:5' ttattttaATAT-TGATGTCAg 3'</td><td>-9.90</td><td>157.00</td><td>Show result profile</td></tr>
<tr><td>349</td><td>hsa-miR-490-3p</td><td>2240~2261</td><td>22</td><td>miRNA: 3' guCGUACCUCAGGAGGUCCAac 5'
|| || |||||||||
Target:5' gaGCTCGGCACCCTCCAGGTcc 3'</td><td>-21.40</td><td>140.00</td><td>Show result profile</td></tr>
<tr><td rowspan="2">350</td><td rowspan="2">hsa-miR-491-5p</td><td>2174~2195</td><td>22</td><td>miRNA: 3' ggaGUACCUUCCCAAGGGGUga 5'
|| || :||| ||||||
Target:5' gggCAGGGCGGGGCTCCCCAaa 3'</td><td>-21.20</td><td>147.00</td><td rowspan="2">Show result profile</td></tr>
<tr><td>1239~1260</td><td>22</td><td>miRNA: 3' ggaguaccuucccaAGGGGUGa 5'
|||||||
Target:5' ccagtgcccggcccTCCCCACc 3'</td><td>-13.80</td><td>140.00</td></tr>
<tr><td rowspan="2">351</td><td rowspan="2">hsa-miR-492</td><td>2237~2262</td><td>26</td><td>miRNA: 3' uuCUUAGAAC---AGGGCGUCCAGGa 5'
|:| || | ||: |||||||
Target:5' ggGGAGCTCGGCACCCTCCAGGTCCg 3'</td><td>-18.50</td><td>153.00</td><td rowspan="2">Show result profile</td></tr>
<tr><td>1483~1513</td><td>31</td><td>miRNA: 3' uucuUAGAA---CAGG-G---C-GUCCAGGa 5'
:|||| | || | | |||||||
Target:5' agctGTCTTGGGGGCCACAAGGCCAGGTCCa 3'</td><td>-21.70</td><td>143.00</td></tr>
<tr><td>352</td><td>hsa-miR-493*</td><td>2544~2565</td><td>22</td><td>miRNA: 3' uuACUUUCGGAUG-GUACAUGUu 5'
| | || :||| ||||||:|
Target:5' agTAATAG-TTACACATGTATAt 3'</td><td>-10.30</td><td>143.00</td><td>Show result profile</td></tr>
<tr><td></td><td></td><td>2603~2630</td><td>28</td><td>miRNA: 3' cucCAAAGGG---CA---CAUACAAAGu 5'
|| |:|: || |</td><td>-8.60</td><td>147.00</td><td></td></tr>
</table>

<table>
<tr><td rowspan="2">353</td><td rowspan="2">hsa-miR-494</td><td></td><td>21</td><td>|||||||
Target:5'
aacGTATTCTGAAGTAACGGATGTTTCt 3'</td><td></td><td></td><td rowspan="2">Show result profile</td></tr>
<tr><td>2419~2441</td><td>23</td><td>miRNA: 3' cuCCAAAGGGCACA-UACAAAgu
5'
||| | ||| | ||||||
Target:5' tcGGTCTGACGTCTGATGTTTgt
3'</td><td>-7.90</td><td>140.00</td></tr>
<tr><td>354</td><td>hsa-miR-496</td><td>849~869</td><td>21</td><td>miRNA: 3' cuCUAACCGGUACAUUAUGAGu
5'
|||| |::|||||| ||||
Target:5' ttGATT-GTTATGTAAAACTCg
3'</td><td>-16.10</td><td>147.00</td><td>Show result profile</td></tr>
<tr><td rowspan="2">355</td><td rowspan="2">hsa-miR-497</td><td>1063~1084</td><td>22</td><td>miRNA: 3' ugUUUGGUGUC-ACACGACGAc
5'
:|||| :| |||||||||
Target:5' taGAACCTGGGCTGTGCTGCTt
3'</td><td>-17.90</td><td>163.00</td><td rowspan="2">Show result profile</td></tr>
<tr><td>1360~1380</td><td>21</td><td>miRNA: 3' uguuugGUGUCACACGACGAc 5'
:||| ||||||||
Target:5' gctgtgTACAAAGTGCTGCTg 3'</td><td>-18.60</td><td>155.00</td></tr>
<tr><td rowspan="2">356</td><td rowspan="2">hsa-miR-497*</td><td>2423~2447</td><td>25</td><td>miRNA: 3' agAUUGUGG--UGU-
CACACCAAAc 5'
|:||::| |:: ||||||||
Target:5'
tcTGACGTCTGATGTTTGTGGTTTg 3'</td><td>-20.00</td><td>154.00</td><td rowspan="2">Show result profile</td></tr>
<tr><td>1792~1816</td><td>25</td><td>miRNA: 3' agAUUGUGGUG---
UCACACCAAAc 5'
|:| :|||| |||||||| |
Target:5'
ccTGAGGCCACCTGAGTGTGGTCTg 3'</td><td>-23.70</td><td>140.00</td></tr>
<tr><td>357</td><td>hsa-miR-498</td><td>2470~2491</td><td>22</td><td>miRNA: 3' cuUUUUGCGGGGGACCGAACUUu
5'
::||: :| ||||:|||||
Target:5' ggGGAAT-TCAGCTGGTTTGAAt
3'</td><td>-16.40</td><td>144.00</td><td>Show result profile</td></tr>
<tr><td>358</td><td>hsa-miR-500a*</td><td>2139~2163</td><td>25</td><td>miRNA: 3' guCUUAGG---
AACGGGUCCACGUa 5'
|::||| | ||||| |||||
Target:5'
ctGGGTCCCCGTGGCCCAAGTGCAg 3'</td><td>-20.30</td><td>140.00</td><td>Show result profile</td></tr>
<tr><td>359</td><td>hsa-miR-500b</td><td>1583~1600</td><td>18</td><td>miRNA: 3' ugGGUCCAUCGUUCCUAa 5'
|||||| ||:||||
Target:5' ggCCAGGTCACAGGGATg 3'</td><td>-18.50</td><td>148.00</td><td>Show result profile</td></tr>
<tr><td>360</td><td>hsa-miR-501-5p</td><td>182~205</td><td>24</td><td>miRNA: 3' agAGUGGGUCC-CU-GUUUCCUAa
5'
||:|||||| || |||| ||
Target:5' acTCGCCCAGGAGAGGAAAGCATg
3'</td><td>-24.29</td><td>140.00</td><td>Show result profile</td></tr>
</table>

<table>
<tr><td rowspan="3">361</td><td rowspan="3">hsa-miR-503</td><td>1353~1380</td><td>28</td><td>miRNA: 3' gacGUCUUGACA-AGG----
GCGACGAu 5'
|||::|||| | |
:||||||
Target:5'
aaaCAGGGCTGTGTACAAAGTGCTGCTg 3'</td><td>-22.50</td><td>146.00</td><td rowspan="3">Show result profile</td></tr>
<tr><td>2262~2284</td><td>23</td><td>miRNA: 3' gaCGUCUUGACAAGGGCGACGAu
5'
|::|:||| ||||||:|
Target:5' gtGTGGGACTCCAGCCGCTGTTg
3'</td><td>-21.60</td><td>145.00</td></tr>
<tr><td>1060~1084</td><td>25</td><td>miRNA: 3' gacGUCUU-GACAAGG-
GCGACGAu 5'
:|||| ||| |: :||||||
Target:5'
ttcTAGAACCTGGGCTGTGCTGCTt 3'</td><td>-17.50</td><td>144.00</td></tr>
<tr><td rowspan="4">362</td><td rowspan="4">hsa-miR-504</td><td>1~16</td><td>16</td><td>miRNA: 3' cuaucucacGUCUGGUCCCAGa
5'
|: :||||||||
Target:5' ------gccCGCGCCAGGGTCc
3'</td><td>-17.10</td><td>149.00</td><td rowspan="4">Show result profile</td></tr>
<tr><td>806~826</td><td>21</td><td>miRNA: 3' cuaucucACGUCUGGUCCCAGa
5'
| || | |||||||
Target:5' gcccgttTCCATA-CAGGGTCt
3'</td><td>-13.80</td><td>146.00</td></tr>
<tr><td>2147~2168</td><td>22</td><td>miRNA: 3' cuaucucacgUCUGGUCCCAGa
5'
|| |||||||
Target:5' ccgtggcccaAGTGCAGGGTCc
3'</td><td>-13.60</td><td>144.00</td></tr>
<tr><td>581~603</td><td>23</td><td>miRNA: 3' cuAUCUCACG-UCUGGUCCCAGa
5'
|:|||| | ||| ||||||
Target:5' agTGGAGTACGAGAAAAGGGTCc
3'</td><td>-20.50</td><td>140.00</td></tr>
<tr><td>363</td><td>hsa-miR-505*</td><td>2016~2035</td><td>20</td><td>miRNA: 3' uguAGUUAUGAAGGACCGAGGg
5'
|| | || | ||||||||
Target:5' cccTCCA-ACCT-CTGGCTCCc
3'</td><td>-18.90</td><td>153.00</td><td>Show result profile</td></tr>
<tr><td>364</td><td>hsa-miR-505</td><td>2266~2287</td><td>22</td><td>miRNA: 3' ucCUUUGGUCGUUCACAACUGc
5'
|| ||||| : |||||:|
Target:5' ggGACTCCAGCCGCTGTTGGCt
3'</td><td>-18.20</td><td>148.00</td><td>Show result profile</td></tr>
<tr><td>365</td><td>hsa-miR-506</td><td>2355~2379</td><td>25</td><td>miRNA: 3' agaUGAGUCUUC-C---
CACGGAAu 5'
|||| | :| | |||||||
Target:5'
gcgACTCTGCGGTGCCAGTGCCTTg 3'</td><td>-14.30</td><td>150.00</td><td>Show result profile</td></tr>
</table>

366	hsa-miR-509-5p	2387~2409	23	miRNA: 3' acuaaCGGUGAC---AGACGUCAu 5'<br>||:|| | ||||||| Target:5' ttccgGCTAC-GCACCCTGCAGTc 3'	-14.60	143.00	Show result profile
367	hsa-miR-509-3p	2613~2639	27	miRNA: 3' gaugggUGUCUGCA-----UGGUUAGu 5'<br>||:||:|| :|||||| Target:5' gaagtaACGGATGTTTCTCGCCAATCg 3'	-17.40	140.00	Show result profile
368	hsa-miR-509-5p	2387~2409	23	miRNA: 3' acuaaCGGUGAC---AGACGUCAu 5'<br>||:|| | ||||||| Target:5' ttccgGCTAC-GCACCCTGCAGTc 3'	-14.60	143.00	Show result profile
369	hsa-miR-509-3p	2613~2639	27	miRNA: 3' gaugggUGUCUGCA-----UGGUUAGu 5'<br>||:||:|| :|||||| Target:5' gaagtaACGGATGTTTCTCGCCAATCg 3'	-17.40	140.00	Show result profile
370	hsa-miR-509-3-5p	2385~2409	25	miRNA: 3' guacuaaCGGUGC---AGACGUCAu 5'<br>||:||| ||||||| Target:5' ttttccgGCTACGCACCCTGCAGTc 3'	-16.90	151.00	Show result profile
371	hsa-miR-509-3p	2613~2639	27	miRNA: 3' gaugggUGUCUGCA-----UGGUUAGu 5'<br>||:||:|| :|||||| Target:5' gaagtaACGGATGTTTCTCGCCAATCg 3'	-17.40	140.00	Show result profile
372	hsa-miR-510	1792~1809	18	miRNA: 3' cacuaaCGGUGAGAGGACUCAu 5'<br>|||| ||||||| Target:5' cctgagGCCA----CCTGAGTg 3'	-15.90	146.00	Show result profile
373	hsa-miR-515-5p	1684~1707	24	miRNA: 3' gucuUUCACGAAAGAAAACCUCUu 5'<br>|:|||||||:| | ||||| Target:5' cgccAGGTGCTTTTTCTGGGAGAg 3'	-19.80	152.00	Show result profile
374	hsa-miR-515-3p	1946~1969	24	miRNA: 3' uugcGAGGUU--UUCUUCCGUGag 5'<br>|||| | |:|||||||| Target:5' tcacCTCCCAGCAGGAAGGCACag 3'	-18.80	148.00	Show result profile
375	hsa-miR-515-5p	1684~1707	24	miRNA: 3' gucuUUCACGAAAGAAAACCUCUu 5'<br>|:|||||||:| | ||||| Target:5' cgccAGGTGCTTTTTCTGGGAGAg 3'	-19.80	152.00	Show result profile

<table>
<tr><td>376</td><td>hsa-miR-515-3p</td><td>1946~1969</td><td>24</td><td>miRNA: 3' uugcGAGGUU--UUCUUCCGUGag
5'
|||| | |:||||||||
Target:5' tcacCTCCCAGCAGGAAGGCACag
3'</td><td>-18.80</td><td>148.00</td><td>Show result profile</td></tr>
<tr><td rowspan="4">377</td><td rowspan="4">hsa-miR-516a-3p</td><td>310~327</td><td>18</td><td>miRNA: 3' ugGGAGACUUUCCUUCGu 5'
|| : |:|||||:||
Target:5' ctCCGTGGGAAGGAGGCt 3'</td><td>-17.10</td><td>140.00</td><td rowspan="4">Show result profile</td></tr>
<tr><td>1216~1233</td><td>18</td><td>miRNA: 3' ugggagACUUUCCUUCGu 5'
|||:||||:||
Target:5' tgaaagTGAGAGGAGGCg 3'</td><td>-16.70</td><td>140.00</td></tr>
<tr><td>310~327</td><td>18</td><td>miRNA: 3' ugGGAGACUUUCCUUCGu 5'
|| : |:|||||:||
Target:5' ctCCGTGGGAAGGAGGCt 3'</td><td>-17.10</td><td>140.00</td></tr>
<tr><td>1216~1233</td><td>18</td><td>miRNA: 3' ugggagACUUUCCUUCGu 5'
|||:||||:||
Target:5' tgaaagTGAGAGGAGGCg 3'</td><td>-16.70</td><td>140.00</td></tr>
<tr><td rowspan="4">378</td><td rowspan="4">hsa-miR-516b*</td><td>310~327</td><td>18</td><td>miRNA: 3' ugGGAGACUUUCCUUCGu 5'
|| : |:|||||:||
Target:5' ctCCGTGGGAAGGAGGCt 3'</td><td>-17.10</td><td>140.00</td><td rowspan="4">Show result profile</td></tr>
<tr><td>1216~1233</td><td>18</td><td>miRNA: 3' ugggagACUUUCCUUCGu 5'
|||:||||:||
Target:5' tgaaagTGAGAGGAGGCg 3'</td><td>-16.70</td><td>140.00</td></tr>
<tr><td>310~327</td><td>18</td><td>miRNA: 3' ugGGAGACUUUCCUUCGu 5'
|| : |:|||||:||
Target:5' ctCCGTGGGAAGGAGGCt 3'</td><td>-17.10</td><td>140.00</td></tr>
<tr><td>1216~1233</td><td>18</td><td>miRNA: 3' ugggagACUUUCCUUCGu 5'
|||:||||:||
Target:5' tgaaagTGAGAGGAGGCg 3'</td><td>-16.70</td><td>140.00</td></tr>
<tr><td rowspan="3">379</td><td rowspan="3">hsa-miR-517*</td><td>1151~1172</td><td>22</td><td>miRNA: 3' ucuGUCACGAAGGUAGAUCUCc
5'
| |||:| |::|:|||||
Target:5' tgtCTGTGTTGCTGTTTAGAGt
3'</td><td>-14.30</td><td>151.00</td><td rowspan="3">Show result profile</td></tr>
<tr><td>427~453</td><td>27</td><td>miRNA: 3' ucUGUCAC---GA-AGGUAG-
AUCUCc 5'
|||||| || ||||||
|||||
Target:5'
ggACAGTGTGCCTGTCCATCTTAGAGg 3'</td><td>-29.30</td><td>148.00</td></tr>
<tr><td>1151~1172</td><td>22</td><td>miRNA: 3' ucuGUCACGAAGGUAGAUCUCc
5'
| |||:| |::|:|||||
Target:5' tgtCTGTGTTGCTGTTTAGAGt
3'</td><td>-14.30</td><td>151.00</td></tr>
</table>

		427~453	27	miRNA: 3' ucUGUCAC---GA-AGGUAG-AUCUCc 5'<br>|||||| || |||||| ||||| Target:5' ggACAGTGTGCCTGTCCATCTTAGAGg 3'	-29.30	148.00	
380	hsa-miR-517b	1296~1317	22	miRNA: 3' uuGUGAGAU-UUCCCUACGUGCu 5'<br>|| |||: ||||||||| Target:5' tcCA-TCTGCTTCGGATGCACGc 3'	-17.10	151.00	Show result profile
381	hsa-miR-517*	1151~1172	22	miRNA: 3' ucuGUCACGAAGGUAGAUCUCc 5'<br>| |||:| |::|:||||| Target:5' tgtCTGTGTTGCTGTTTAGAGt 3'	-14.30	151.00	Show result profile
		427~453	27	miRNA: 3' ucUGUCAC---GA-AGGUAG-AUCUCc 5'<br>|||||| || |||||| ||||| Target:5' ggACAGTGTGCCTGTCCATCTTAGAGg 3'	-29.30	148.00	
382	hsa-miR-518a-3p	1675~1697	23	miRNA: 3' agGUCGUUU-CCCUUCGCGAAAg 5'<br>::||||| | |:|:||||| Target:5' cgTGGCAAACGCCAGGTGCTTTt 3'	-13.20	148.00	Show result profile
		1675~1697	23	miRNA: 3' agGUCGUUU-CCCUUCGCGAAAg 5'<br>::||||| | |:|:||||| Target:5' cgTGGCAAACGCCAGGTGCTTTt 3'	-13.20	148.00	
383	hsa-miR-518c*	1711~1731	21	miRNA: 3' gucuuuCACGAAGGGAGGUCUCu 5'<br>||| ||||||||:| Target:5' acagccGTG--GCCCTCCAGGGc 3'	-24.30	141.00	Show result profile
384	hsa-miR-518d-5p	1036~1057	22	miRNA: 3' gucuuucaCGAAGGGAGAUCUc 5'<br>||| |:||||||| Target:5' ctcctgccGCTCCTCTCTAGAa 3'	-14.50	158.00	Show result profile
385	hsa-miR-518d-3p	1676~1697	22	miRNA: 3' cgagGUUU-CCCUUCGCGAAAc 5'<br>|||| | |:|:||||| Target:5' gtggCAAACGCCAGGTGCTTTt 3'	-11.10	141.00	Show result profile
386	hsa-miR-518e*	1036~1057	22	miRNA: 3' gucuuucGCGAAGGGAGAUCUc 5'<br>|||| |:||||||| Target:5' ctcctgcCGCTCCTCTCTAGAa 3'	-18.10	163.00	Show result profile

<table>
<tr><td>387</td><td>hsa-miR-518f*</td><td>1036~1057</td><td>22</td><td>miRNA: 3' cucuuucaCGAAGGGAGAUCUc 5'
||| |:|||||||
Target:5' ctcctgccGCTCCTCTCTAGAa 3'</td><td>-14.50</td><td>158.00</td><td>Show result profile</td></tr>
<tr><td>388</td><td>hsa-miR-519a*</td><td>1036~1057</td><td>22</td><td>miRNA: 3' gucuuucGCGAAGGGAGAUCUc 5'
|||| |:|||||||
Target:5' ctcctgcCGCTCCTCTCTAGAa 3'</td><td>-18.10</td><td>163.00</td><td>Show result profile</td></tr>
<tr><td rowspan="2">389</td><td rowspan="2">hsa-miR-519a</td><td>982~1003</td><td>22</td><td>miRNA: 3' ugUGAGAUUUUCCUACGUGAAa 5'
|| |: |: | |||||||
Target:5' tcACCGTGCAGAGCTGCACTTg 3'</td><td>-10.00</td><td>152.00</td><td rowspan="2">Show result profile</td></tr>
<tr><td>982~1003</td><td>22</td><td>miRNA: 3' ugUGAGAUUUUCCUACGUGAAa 5'
|| |: |: | |||||||
Target:5' tcACCGTGCAGAGCTGCACTTg 3'</td><td>-10.00</td><td>152.00</td></tr>
<tr><td>390</td><td>hsa-miR-519b-5p</td><td>1036~1057</td><td>22</td><td>miRNA: 3' gucuuucGCGAAGGGAGAUCUc 5'
|||| |:|||||||
Target:5' ctcctgcCGCTCCTCTCTAGAa 3'</td><td>-18.10</td><td>163.00</td><td>Show result profile</td></tr>
<tr><td>391</td><td>hsa-miR-519b-3p</td><td>978~1003</td><td>26</td><td>miRNA: 3' uuGGAG----AUUUUCCUACGUGAAa 5'
:||| |: |: | |||||||
Target:5' ctTCTCACCGTGCAGAGCTGCACTTg 3'</td><td>-11.10</td><td>150.00</td><td>Show result profile</td></tr>
<tr><td>392</td><td>hsa-miR-519c-5p</td><td>1036~1057</td><td>22</td><td>miRNA: 3' gucuuucGCGAAGGGAGAUCUc 5'
|||| |:|||||||
Target:5' ctcctgcCGCTCCTCTCTAGAa 3'</td><td>-18.10</td><td>163.00</td><td>Show result profile</td></tr>
<tr><td>393</td><td>hsa-miR-519c-3p</td><td>978~1003</td><td>26</td><td>miRNA: 3' uaGGAG----AUUUUUCUACGUGAAa 5'
:||| |: |:|| |||||||
Target:5' ctTCTCACCGTGCAGAGCTGCACTTg 3'</td><td>-12.60</td><td>158.00</td><td>Show result profile</td></tr>
<tr><td>394</td><td>hsa-miR-519e*</td><td>1686~1707</td><td>22</td><td>miRNA: 3' cuUUCACGAGGGAAAACCUCUu 5'
|:|||||:::| | |||||
Target:5' ccAGGTGCTTTTTCTGGGAGAg 3'</td><td>-19.00</td><td>144.00</td><td>Show result profile</td></tr>
<tr><td>395</td><td>hsa-miR-520a-5p</td><td>947~967</td><td>21</td><td>miRNA: 3' ucuUUCAUGAAGGGAGACCUc 5'
:||| | || ||||||:
Target:5' ggcGAGTTCCTCGCTCTGGGa 3'</td><td>-15.50</td><td>146.00</td><td>Show result profile</td></tr>
</table>

<table>
<tr><td>396</td><td>hsa-miR-520c-5p</td><td>1036~1057</td><td>22</td><td>miRNA: 3' gucuuucaCGAAGGGAGAUCUc 5'
                  ||| |:|||||||
Target:5' ctcctgccGCTCCTCTCTAGAa 3'</td><td>-14.50</td><td>158.00</td><td>Show result profile</td></tr>
<tr><td>397</td><td>hsa-miR-520d-5p</td><td>1335~1354</td><td>20</td><td>miRNA: 3' cuuucccgaagGGAAACAUc 5'
                     :|||||||
Target:5' cttgccctggaTCTTTGTAa 3'</td><td>-7.90</td><td>141.00</td><td>Show result profile</td></tr>
<tr><td>398</td><td>hsa-miR-520d-3p</td><td>2834~2853</td><td>20</td><td>miRNA: 3' ugGGUGGUUUCUCUUCGUGAAa 5'
            ||||| | | |||||:||
Target:5' atCCACC-ATTA-AAGCATTTg 3'</td><td>-16.70</td><td>142.00</td><td>Show result profile</td></tr>
<tr><td rowspan="4">399</td><td rowspan="4">hsa-miR-521</td><td>2746~2767</td><td>22</td><td>miRNA: 3' ugUGAGAUUUCCCUUCACGCAa 5'
            | | ||:|| |||||:||
Target:5' ttAGTGGAAGGGAAAGTGTGTt 3'</td><td>-18.20</td><td>148.00</td><td rowspan="4">Show result profile</td></tr>
<tr><td>267~290</td><td>24</td><td>miRNA: 3' uguGAG-AUUU-CCCUUCACGCaa 5'
             ||| |:|| ||:||||||
Target:5' aacCTCATGAACTGGGAGTGCGcc 3'</td><td>-18.60</td><td>143.00</td></tr>
<tr><td>2746~2767</td><td>22</td><td>miRNA: 3' ugUGAGAUUUCCCUUCACGCAa 5'
            | | ||:|| |||||:||
Target:5' ttAGTGGAAGGGAAAGTGTGTt 3'</td><td>-18.20</td><td>148.00</td></tr>
<tr><td>267~290</td><td>24</td><td>miRNA: 3' uguGAG-AUUU-CCCUUCACGCaa 5'
             ||| |:|| ||:||||||
Target:5' aacCTCATGAACTGGGAGTGCGcc 3'</td><td>-18.60</td><td>143.00</td></tr>
<tr><td>400</td><td>hsa-miR-522*</td><td>1036~1057</td><td>22</td><td>miRNA: 3' gucuuucGCGAAGGGAGAUCUc 5'
                 |||| |:|||||||
Target:5' ctcctgcCGCTCCTCTCTAGAa 3'</td><td>-18.10</td><td>163.00</td><td>Show result profile</td></tr>
<tr><td>401</td><td>hsa-miR-523*</td><td>1036~1057</td><td>22</td><td>miRNA: 3' gucuuucGCGAAGGGAGAUCUc 5'
                 |||| |:|||||||
Target:5' ctcctgcCGCTCCTCTCTAGAa 3'</td><td>-18.10</td><td>163.00</td><td>Show result profile</td></tr>
<tr><td rowspan="2"></td><td rowspan="2"></td><td>2655~2673</td><td>19</td><td>miRNA: 3' cuCUUUCACGAAGGGAAACAUc 5'
            |||| || |:|:||||||
Target:5' acGAAA-TG--TTCTTTTGTAg 3'</td><td>-11.90</td><td>147.00</td><td rowspan="2"></td></tr>
<tr><td></td><td></td><td>miRNA: 3' cucUUUCACGAAGGGAAACAUc 5'</td><td></td><td></td></tr>
</table>

402	hsa-miR-524-5p	2506~2527	22	|:| ||: |: :|||||| Target:5' cccAGAATGTCTTATTTTGTAa 3'	-8.50	143.00	Show result profile
		1327~1354	28	miRNA: 3' cucuuuCACGAAGG------GAAACAUc 5'<br>|| :| || ||||||| Target:5' ggctctGTCTTGCCCTGGATCTTTGTAa 3'	-8.30	142.00	
		2725~2747	23	miRNA: 3' cuCUUU-CACGAAGGGAAACAuc 5'<br>|||| | | | ||||||| Target:5' ctGAAACGCGGTCACCTTTGTtt 3'	-10.70	140.00	
403	hsa-miR-524-3p	65~85	21	miRNA: 3' ugagGUUUCCCUUCGCGGaag 5'<br>|||||||||||||| Target:5' gagaCAAAGGGAAGCGCCgcc 3'	-27.20	145.00	Show result profile
404	hsa-miR-525-5p	947~967	21	miRNA: 3' ucuUUCACGUAGGGAGACCUc 5'<br>:||| | || ||||||:<br>Target:5' ggcGAGTTCCTCGCTCTGGGa 3'	-15.50	146.00	Show result profile
405	hsa-miR-525-3p	2357~2379	23	miRNA: 3' gcGAGAUUUC-CCUUCGCGGAAg 5'<br>||||: :| | ||:||||| Target:5' gaCTCTGCGGTGCCAGTGCCTTg 3'	-16.80	144.00	Show result profile
		64~85	22	miRNA: 3' gcgagaUUUCCCUUCGCGGaag 5'<br>||||||||||||| Target:5' cgagacAAAGGGAAGCGCCgcc 3'	-24.80	140.00	
406	hsa-miR-526a	1036~1057	22	miRNA: 3' gucuuucaCGAAGGGAGAUCUc 5'<br>||| |:||||||| Target:5' ctcctgccGCTCCTCTCTAGAa 3'	-14.50	158.00	Show result profile
		1036~1057	22	miRNA: 3' gucuuucaCGAAGGGAGAUCUc 5'<br>||| |:||||||| Target:5' ctcctgccGCTCCTCTCTAGAa 3'	-14.50	158.00	
407	hsa-miR-539	2611~2632	22	miRNA: 3' ugugUGGUU-CCUAUUAAAGAGg 5'<br>| :|| |||| :|||||| Target:5' ctgaAGTAACGGAT-GTTTCTCg 3'	-10.50	141.00	Show result profile
408	hsa-miR-541*	1755~1789	35	miRNA: 3' ucaCCCUGG--CUGUCG--------UCUUAGGAAa 5'<br>||| || |:|||| :||||:||| Target:5' agaGGGCCCTGGGCAGCCTGTGTCTGGAATTCTTc	-19.50	146.00	Show result profile

<table>
<tr><td></td><td></td><td></td><td></td><td>3'</td><td></td><td></td><td></td></tr>
<tr><td>409</td><td>hsa-miR-542-5p</td><td>2125~2150</td><td>26</td><td>miRNA: 3' agagCACUGUACU--A-CUAGGGGCu 5'
|||:| || |
|:||||||
Target:5' cccgGTGGCTGGAGCTGGGTCCCCGt 3'</td><td>-23.40</td><td>141.00</td><td>Show result profile</td></tr>
<tr><td>410</td><td>hsa-miR-543</td><td>397~417</td><td>21</td><td>miRNA: 3' uucuUCACGUGGCGCUUACAaa 5'
| | |||| |||||||
Target:5' cattATTTCACC-CGAATGTgt 3'</td><td>-14.30</td><td>141.00</td><td>Show result profile</td></tr>
<tr><td rowspan="3">411</td><td rowspan="3">hsa-miR-544b</td><td>1612~1633</td><td>22</td><td>miRNA: 3' aaucuUUACGUGUUGGAGUCCa 5'
|: |::| ||||||||
Target:5' tgcatAGGGTGCCACCTCAGGt 3'</td><td>-20.50</td><td>157.00</td><td rowspan="3">Show result profile</td></tr>
<tr><td>989~1012</td><td>24</td><td>miRNA: 3' aaUCUUUACGUG--UUGGAGUCca 5'
|||: ||||| ::||||||
Target:5' gcAGAGCTGCACTTGGCCTCAGct 3'</td><td>-18.70</td><td>150.00</td></tr>
<tr><td>1637~1656</td><td>20</td><td>miRNA: 3' aaucuuuACGUGUUGGAGUCCa 5'
||::: |||||||
Target:5' tgtccctTGTGT--CCTCAGGa 3'</td><td>-12.00</td><td>143.00</td></tr>
<tr><td>412</td><td>hsa-miR-548a-5p</td><td>859~880</td><td>22</td><td>miRNA: 3' ccAUUUUGAGCGUUAAUGAAAa 5'
|||||||||| |||:|||
Target:5' tgTAAAACTCGCTTTTATTTTa 3'</td><td>-18.90</td><td>168.00</td><td>Show result profile</td></tr>
<tr><td rowspan="4">413</td><td rowspan="4">hsa-miR-548aa</td><td>653~678</td><td>26</td><td>miRNA: 3' acCACGUUUUCAUUAAC--ACCAAAaa 5'
|| |||||| || |
||||||
Target:5' tcGT-CAAAAGGAAGGGATTGGTTTgg 3'</td><td>-9.30</td><td>148.00</td><td rowspan="4">Show result profile</td></tr>
<tr><td>2427~2448</td><td>22</td><td>miRNA: 3' acCACGUUUUCAUUAACACCAAAaa 5'
|| | :| || |||||||||
Target:5' acGT-CTGATGT--TTGTGGTTTgt 3'</td><td>-9.50</td><td>142.00</td></tr>
<tr><td>653~678</td><td>26</td><td>miRNA: 3' acCACGUUUUCAUUAAC--ACCAAAaa 5'
|| |||||| || |
||||||
Target:5' tcGT-CAAAAGGAAGGGATTGGTTTgg 3'</td><td>-9.30</td><td>148.00</td></tr>
<tr><td>2427~2448</td><td>22</td><td>miRNA: 3' acCACGUUUUCAUUAACACCAAAaa 5'
|| | :| || |||||||||</td><td>-9.50</td><td>142.00</td></tr>
</table>

<table>
<tr><td></td><td></td><td></td><td></td><td>Target:5' acGT-CTGATGT--
TTGTGGTTTgt 3'</td><td></td><td></td><td></td></tr>
<tr><td>414</td><td>hsa-miR-548b-5p</td><td>858~880</td><td>23</td><td>miRNA: 3' ccgGUUUUG-GUGUUAAUGAAAa 5'
:||||| |:| |||:|||
Target:5' atgTAAAACTCGCTTTTATTTTa 3'</td><td>-9.90</td><td>147.00</td><td>Show result profile</td></tr>
<tr><td>415</td><td>hsa-miR-548c-5p</td><td>859~880</td><td>22</td><td>miRNA: 3' ccGUUUUUGGCGUUAAUGAAAa 5'
:|||| :||| |||:|||
Target:5' tgTAAAACTCGCTTTTATTTTa 3'</td><td>-12.30</td><td>152.00</td><td>Show result profile</td></tr>
<tr><td>416</td><td>hsa-miR-548d-5p</td><td>859~880</td><td>22</td><td>miRNA: 3' ccGUUUUUGGUGUUAAUGAAAa 5'
:|||| :|:| |||:|||
Target:5' tgTAAAACTCGCTTTTATTTTa 3'</td><td>-10.40</td><td>148.00</td><td>Show result profile</td></tr>
<tr><td>417</td><td>hsa-miR-548d-3p</td><td>1170~1200</td><td>31</td><td>miRNA: 3' cguUUUCUUUGACA---------CCAAAAac 5'
||| |||||||
||||||
Target:5' agtAAATAAACTGTTTATATAAAGGTTTTgg 3'</td><td>-10.50</td><td>143.00</td><td>Show result profile</td></tr>
<tr><td>418</td><td>hsa-miR-548d-5p</td><td>859~880</td><td>22</td><td>miRNA: 3' ccGUUUUUGGUGUUAAUGAAAa 5'
:|||| :|:| |||:|||
Target:5' tgTAAAACTCGCTTTTATTTTa 3'</td><td>-10.40</td><td>148.00</td><td>Show result profile</td></tr>
<tr><td>419</td><td>hsa-miR-548d-3p</td><td>1170~1200</td><td>31</td><td>miRNA: 3' cguUUUCUUUGACA---------CCAAAAac 5'
||| |||||||
||||||
Target:5' agtAAATAAACTGTTTATATAAAGGTTTTgg 3'</td><td>-10.50</td><td>143.00</td><td>Show result profile</td></tr>
<tr><td rowspan="4">420</td><td rowspan="4">hsa-miR-548h</td><td>859~880</td><td>22</td><td>miRNA: 3' cuGUUUUUGGCGCUAAUGAAAa 5'
:|||| :||| |||:|||
Target:5' tgTAAAACTCGCTTTTATTTTa 3'</td><td>-9.60</td><td>152.00</td><td rowspan="4">Show result profile</td></tr>
<tr><td>859~880</td><td>22</td><td>miRNA: 3' cuGUUUUUGGCGCUAAUGAAAa 5'
:|||| :||| |||:|||
Target:5' tgTAAAACTCGCTTTTATTTTa 3'</td><td>-9.60</td><td>152.00</td></tr>
<tr><td>859~880</td><td>22</td><td>miRNA: 3' cuGUUUUUGGCGCUAAUGAAAa 5'
:|||| :||| |||:|||
Target:5' tgTAAAACTCGCTTTTATTTTa 3'</td><td>-9.60</td><td>152.00</td></tr>
<tr><td>859~880</td><td>22</td><td>miRNA: 3' cuGUUUUUGGCGCUAAUGAAAa 5'
:|||| :||| |||:|||</td><td>-9.60</td><td>152.00</td></tr>
</table>

<table>
<tr><td></td><td></td><td></td><td></td><td>Target:5' tgTAAAACTCGCTTTTATTTTa
3'</td><td></td><td></td><td></td></tr>
<tr><td rowspan="4">421</td><td rowspan="4">hsa-miR-548i</td><td>859~880</td><td>22</td><td>miRNA: 3' ccGUUUUAGGCGUUAAUGAAAa
5'
:|||| :||| |||:|||
Target:5' tgTAAAACTCGCTTTTATTTTa
3'</td><td>-12.60</td><td>152.00</td><td rowspan="4">Show result profile</td></tr>
<tr><td>859~880</td><td>22</td><td>miRNA: 3' ccGUUUUAGGCGUUAAUGAAAa
5'
:|||| :||| |||:|||
Target:5' tgTAAAACTCGCTTTTATTTTa
3'</td><td>-12.60</td><td>152.00</td></tr>
<tr><td>859~880</td><td>22</td><td>miRNA: 3' ccGUUUUAGGCGUUAAUGAAAa
5'
:|||| :||| |||:|||
Target:5' tgTAAAACTCGCTTTTATTTTa
3'</td><td>-12.60</td><td>152.00</td></tr>
<tr><td>859~880</td><td>22</td><td>miRNA: 3' ccGUUUUAGGCGUUAAUGAAAa
5'
:|||| :||| |||:|||
Target:5' tgTAAAACTCGCTTTTATTTTa
3'</td><td>-12.60</td><td>152.00</td></tr>
<tr><td>422</td><td>hsa-miR-548j</td><td>859~880</td><td>22</td><td>miRNA: 3' ugGUUUCUGGCGUUAAUGAAAa
5'
:||| :||| |||:|||
Target:5' tgTAAAACTCGCTTTTATTTTa
3'</td><td>-9.40</td><td>144.00</td><td>Show result profile</td></tr>
<tr><td>423</td><td>hsa-miR-548k</td><td>1678~1697</td><td>20</td><td>miRNA: 3' ucGUUUUAGGC-GUUCAUGAAAa
5'
|||| || ||:||:||||
Target:5' ggCAAA---CGCCAGGTGCTTTt
3'</td><td>-15.40</td><td>145.00</td><td>Show result profile</td></tr>
<tr><td>424</td><td>hsa-miR-548l</td><td>328~349</td><td>22</td><td>miRNA: 3' cuguuUUGGGCGUUUAUGAAAa
5'
|| |: |::||:||||
Target:5' tgtttAAACTACGGATGCTTTt
3'</td><td>-11.40</td><td>141.00</td><td>Show result profile</td></tr>
<tr><td>425</td><td>hsa-miR-548m</td><td>330~350</td><td>21</td><td>miRNA: 3' guuUUUGGUGUUUAUGGAAAc 5'
||||:||::||:|:|||
Target:5' tttAAACTACGGATGCTTTTc 3'</td><td>-15.30</td><td>146.00</td><td>Show result profile</td></tr>
<tr><td>426</td><td>hsa-miR-548n</td><td>329~350</td><td>22</td><td>miRNA: 3' uguuUUAGGUGUUAAUGAAAAc
5'
|| |:||:: |:|||||
Target:5' gtttAAACTACGGATGCTTTTc
3'</td><td>-10.10</td><td>146.00</td><td>Show result profile</td></tr>
<tr><td>427</td><td>hsa-miR-548s</td><td>988~1008</td><td>21</td><td>miRNA: 3' uuuUAUUGACGUCAAAACCGGua
5'
| |:||||| ||||||
Target:5' tgcAGAGCTGCA--CTTGGCCtc
3'</td><td>-15.40</td><td>140.00</td><td>Show result profile</td></tr>
</table>

<table>
<tr><td>428</td><td>hsa-miR-548t</td><td>330~350</td><td>21</td><td>miRNA: 3' guuUUUGGUGCUAGUGAAAAc 5'
||||:|||: ::|||||
Target:5' tttAAACTACGGATGCTTTTc 3'</td><td>-13.70</td><td>142.00</td><td>Show result profile</td></tr>
<tr><td>429</td><td>hsa-miR-548v</td><td>2482~2501</td><td>20</td><td>miRNA: 3' acCACGUUUUCAUUGACAUCGa
5'
|| ::|| || :||||||
Target:5' tgGTTTGAA--TATTTGTAGCc
3'</td><td>-10.90</td><td>140.00</td><td>Show result profile</td></tr>
<tr><td>430</td><td>hsa-miR-548w</td><td>858~880</td><td>23</td><td>miRNA: 3' ucCGUUUUUGGCGUCAAUGAAAa
5'
|:|||| :||| |||:|||
Target:5' atGTAAAACTCGCTTTTATTTTa
3'</td><td>-11.50</td><td>157.00</td><td>Show result profile</td></tr>
<tr><td>431</td><td>hsa-miR-551b*</td><td>211~230</td><td>20</td><td>miRNA: 3' ccagaGUGGGUGCGAACUAAAg
5'
|||||| :|||:|||
Target:5' aagacCACCCA--TTTGGTTTc
3'</td><td>-17.60</td><td>145.00</td><td>Show result profile</td></tr>
<tr><td rowspan="2">432</td><td rowspan="2">hsa-miR-552</td><td>723~743</td><td>21</td><td>miRNA: 3' aacagAUUGGUCAGUGGACaa 5'
|:||:|||||||||
Target:5' tacaaTGACTAGTCACCTGgg 3'</td><td>-21.20</td><td>152.00</td><td rowspan="2">Show result profile</td></tr>
<tr><td>91~115</td><td>25</td><td>miRNA: 3' aacagauuGGUCA----
GUGGACAa 5'
:|:|| |||||||
Target:5'
cgccccgcTCGGTCCTCCACCTGTc 3'</td><td>-12.30</td><td>143.00</td></tr>
<tr><td>433</td><td>hsa-miR-555</td><td>1321~1342</td><td>22</td><td>miRNA: 3' uaguCUCCAAGUC-GAAUGGGa
5'
|||| || | |||:|||
Target:5' ctgtGAGGCTCTGTCTTGCCCt
3'</td><td>-18.20</td><td>145.00</td><td>Show result profile</td></tr>
<tr><td>434</td><td>hsa-miR-557</td><td>971~994</td><td>24</td><td>miRNA: 3' ucUGUUCCGGGUG-GGCACGUUUg
5'
:|| | ::| | |||||||:|
Target:5' agGCATGCTTCTCACCGTGCAGAg
3'</td><td>-20.20</td><td>145.00</td><td>Show result profile</td></tr>
<tr><td>435</td><td>hsa-miR-566</td><td>741~761</td><td>21</td><td>miRNA: 3' caaCCCUAG--UGUCCGCGGg 5'
|||:|: :|:||||||
Target:5' gggGGGGTTGGGCGGGCGCCa 3'</td><td>-24.50</td><td>142.00</td><td>Show result profile</td></tr>
<tr><td>436</td><td>hsa-miR-572</td><td>28~48</td><td>21</td><td>miRNA: 3' acCCGGUG-GCGGCUCGCCUg 5'
|||::| ||| ||||||:
Target:5' ctGGCTGCGCGCGGAGCGGGc 3'</td><td>-29.00</td><td>150.00</td><td>Show result profile</td></tr>
<tr><td>437</td><td>hsa-miR-573</td><td>1108~1131</td><td>24</td><td>miRNA: 3' gacuAGUCAAUGUGUAGUGAAGuc
5'
||:||| ::|: ||||||
Target:5' catcTCGGTTCTGCGCCACTTCct
3'</td><td>-15.30</td><td>148.00</td><td>Show result profile</td></tr>
</table>

438	hsa-miR-575	997~1017	21	miRNA: 3' cgaGGACAG--GUUGACCGAg 5'<br>|:|| | ||:|||||| Target:5' gcaCTTGGCCTCAGCTGGCTg 3'	-18.20	142.00	Show result profile
439	hsa-miR-578	441~464	24	miRNA: 3' uguUAGGAU---CUCGUGUUCUUc 5'<br>|||:|| ||| |||||:| Target:5' tccATCTTAGAGGAGGACAAGGAc 3'	-14.40	150.00	Show result profile
		489~513	25	miRNA: 3' ugUUAGGAU----CUCGUGUUCUUc 5'<br>:|||||| || :|||:||| Target:5' caGATCCTATTAGGAATACAGGAAc 3'	-13.20	149.00	
		576~596	21	miRNA: 3' uguuaggAUCUCGUGUUCUUc 5'<br>|:|||:||:|||| Target:5' aacagagTGGAGTACGAGAAa 3'	-16.80	146.00	
440	hsa-miR-587	2564~2583	20	miRNA: 3' caCUGAGUAGUGGAUACCUUu 5'<br>|::| || || ||||||| Target:5' atGGTTAAT-ACATATGGAAa 3'	-12.60	158.00	Show result profile
		1002~1025	24	miRNA: 3' caCUGAGU--AGUGG-AUACCUUu 5'<br>| |||| | :|: ||||||| Target:5' tgGCCTCAGCTGGCTGTATGGAAa 3'	-12.50	153.00	
441	hsa-miR-588	1566~1588	23	miRNA: 3' caagauuGGGUAA--CACCGGUu 5'<br>||: || ||||||| Target:5' gggttccCCTCTTCAGTGGCCAg 3'	-17.80	148.00	Show result profile
442	hsa-miR-589	1098~1121	24	miRNA: 3' gaGUCUCGUC-UGCA-CCAAGAgu 5'<br>|||: ||| |: | |||||| Target:5' ccCAGGTCAGCATCTCGGTTCTgc 3'	-13.90	140.00	Show result profile
443	hsa-miR-593	539~557	19	miRNA: 3' ucuUUGGGGUCGUCUCUGu 5'<br>|:|:| |||||||:| Target:5' cccAGCTCAAGCAGAGGCc 3'	-17.40	148.00	Show result profile
444	hsa-miR-596	1303~1323	21	miRNA: 3' gggcuCCUCGGCCCGUCCGAa 5'<br>||| | ||||||| Target:5' gcttcGGATGCACGCAGGCTg 3'	-15.80	148.00	Show result profile
445	hsa-miR-602	2130~2153	24	miRNA: 3' ccCGGCGUCGA--CAGCGGGCACag 5'<br>||:| |||| ||| |||||| Target:5' tgGCTGGAGCTGGGTC-CCCGTGgc 3'	-28.80	150.00	Show result profile

446	hsa-miR-603	2750~2767	18	miRNA: 3' cgUUUUCAUUAACGUCACACAc 5'<br>:||:| || ||||||| Target:5' tgGAAGGGAA----AGTGTGTt 3'	-9.00	150.00	Show result profile
447	hsa-miR-608	2326~2351	26	miRNA: 3' ugccUCGACA-GGGUUGUGGUGGGGa 5'<br>:|| || ||| ||||||||| Target:5' agccGGCAGTGCCCCCCACCACCCCt 3'	-31.80	169.00	Show result profile
		1652~1677	26	miRNA: 3' ugCCU-CGACAGGGUUGUGGUGGGga 5'<br>||| || | |::: :||||||| Target:5' caGGAGGCAGCCTTGCTACCACCCgt 3'	-22.70	147.00	
		73~97	25	miRNA: 3' ugCCUCGACAG-GGUUGUGGUGGGGa 5'<br>| ||| | | ||: |:||:|||| Target:5' ggGAAGC-GCCGCCGCCGCCGCCCCg 3'	-27.30	146.00	
		1603~1630	28	miRNA: 3' ugcCUCGACA-GGGU--UGUGGUGGGGa 5'<br>| ||||| | :| :::|||||:| Target:5' gctGCGCTGTGCATAGGGTGCCACCTCa 3'	-21.60	144.00	
		2096~2126	31	miRNA: 3' ugCCUCGACAGGGUUG------UGGUGGGGa 5'<br>||||||| ||| :| : |||||| Target:5' ctGGAGCTGGCCCTGCTGCCCTGGCACCCCc 3'	-29.70	141.00	
448	hsa-miR-611	1~20	20	miRNA: 3' cagucugGGGCUC---CCCAGGAGCg 5'<br>|||| | ||||||||| Target:5' ------gCCCGCGCCAGGGTCCTCGg 3'	-26.10	160.00	Show result profile
		2669~2688	20	miRNA: 3' caGUC-UGGGGCUCCCCAGGAGCg 5'<br>:|| ||| | |||||||| Target:5' tgTAGTACCAC----GGTCCTCGg 3'	-18.70	151.00	
		87~109	23	miRNA: 3' cagucUGGGGCUCCCCAGGAGcg 5'<br>:||||| ||||||| Target:5' ccgccGCCCCGCTCGGTCCTCca 3'	-24.60	142.00	
	hsa-	168~192	25	miRNA: 3' uuccucGAGUCUUCGGGACGGGUCg 5'<br>||||| || |: |||||| Target:5' atcgccCTCAGCAGACTCGCCCAGg 3'	-22.10	143.00	

449	miR-612	1225~1250	26	miRNA: 3' uuCCUCGAGUCUUCGGG--ACGGGUCg 5'<br>|||| |:| ||| |||||:| Target:5' gaGGAG-GCGGCCTCCCAGTGCCCGGc 3'	-28.30	140.00	Show result profile
450	hsa-miR-615-5p	2251~2274	24	miRNA: 3' cuAGGCUCGUGGC---CCCUGGGGg 5'<br>||| || ||| |||||:|| Target:5' ccTCC-AGGTCCGTGTGGGACTCCa 3'	-24.30	143.00	Show result profile
451	hsa-miR-616*	1068~1090	23	miRNA: 3' uucagUGAC-UUCCCAAAACUCa 5'<br>:||| : | ||||||| Target:5' cctggGCTGTGCTGCTTTTGAGc 3'	-7.90	145.00	Show result profile
452	hsa-miR-616	706~732	27	miRNA: 3' gacGAGUUUGG-GAG----GUUACUGa 5'<br>|| ||::: ||| ||||||| Target:5' aatCTAAAGTTGCTCCATACAATGACt 3'	-15.40	153.00	Show result profile
453	hsa-miR-617	43~63	21	miRNA: 3' cggugGAAGUUUACCCUUCAGa 5'<br>|| |::| |||||||| Target:5' gcgggCTCCGGA-GGGAAGTCc 3'	-19.20	156.00	Show result profile
454	hsa-miR-619	2242~2260	19	miRNA: 3' ugACCCGUGUUUGUACAGGUCCAg 5'<br>| ||||| | ||||||| Target:5' gcTCGGCAC---C--CTCCAGGTc 3'	-20.40	147.00	Show result profile
		1698~1731	34	miRNA: 3' ugACCC------GUGUUUGUAC----AGGUCCag 5'<br>|||| ||||: |:|| |||||| Target:5' tcTGGGAGAGCCCACAGCCGTGGCCCTCCAGGgc 3'	-24.70	142.00	
455	hsa-miR-622	166~186	21	miRNA: 3' cgaGGUUGGAGUCGUCUGAca 5'<br>:|: ||||||||||| Target:5' ggaTCGCCCTCAGCAGACTcg 3'	-24.20	154.00	Show result profile
456	hsa-miR-623	130~151	22	miRNA: 3' uggGUUGUCGG-GGACGUUCCCUa 5'<br>| :|:||| || |:||||| Target:5' gggCTGCGGCCGCC--CGAGGGAc 3'	-19.80	140.00	Show result profile
		2361~2381	21	miRNA: 3' ucCAUUAUGGUUAUGGAACAc 5'<br>| ::|:|||:|:|||||| Target:5' ctGCGGTGCCAGTGCCTTGTt 3'	-19.90	155.00	

<table>
<tr><td rowspan="2">457</td><td rowspan="2">hsa-miR-624</td><td>2693~2713</td><td>21</td><td>miRNA: 3' ucCAUUAUGGUUAUGGAACAc 5'
| |: || : |||||||
Target:5' acGAAGGACGTGAACCTTGTa 3'</td><td>-8.60</td><td>147.00</td><td rowspan="2">Show result profile</td></tr>
<tr><td>627~649</td><td>23</td><td>miRNA: 3' uccAUUA-UGGUU-AUGGAACAc
5'
| || | ||: |||||||
Target:5' cccTCATAAGCAGCGACCTTGTg
3'</td><td>-14.00</td><td>146.00</td></tr>
<tr><td rowspan="2">458</td><td rowspan="2">hsa-miR-628-5p</td><td>875~896</td><td>22</td><td>miRNA: 3' ggAGAUCAUUUAUACAGUCGUa
5'
|:|| || |||||||:|
Target:5' atTTTAATATTGATGTCAGTAt
3'</td><td>-13.40</td><td>148.00</td><td rowspan="2">Show result profile</td></tr>
<tr><td>1089~1110</td><td>22</td><td>miRNA: 3' ggagaucauuuaUACAGUCGUa
5'
| |||||||
Target:5' gcctcagaccccAGGTCAGCAt
3'</td><td>-17.54</td><td>142.00</td></tr>
<tr><td>459</td><td>hsa-miR-629*</td><td>1687~1708</td><td>22</td><td>miRNA: 3' cgaCCCGAAUGCAACCCUCUUg
5'
| |||| : |||||||:
Target:5' cagGTGCTTTTTCTGGGAGAGc
3'</td><td>-20.70</td><td>143.00</td><td>Show result profile</td></tr>
<tr><td rowspan="4">460</td><td rowspan="4">hsa-miR-631</td><td>1489~1512</td><td>24</td><td>miRNA: 3' cgACUCC---AGACCCGGUCCAGa
5'
||:|| | |||||||||
Target:5' ctTGGGGGCCACAAGGCCAGGTCc
3'</td><td>-23.50</td><td>155.00</td><td rowspan="4">Show result profile</td></tr>
<tr><td>1571~1592</td><td>22</td><td>miRNA: 3' cgacuccAGAC-CCGGUCCAGa
5'
|| | |||||||||
Target:5' cccctctTCAGTGGCCAGGTCa
3'</td><td>-16.90</td><td>154.00</td></tr>
<tr><td>1084~1106</td><td>23</td><td>miRNA: 3' cgACUC-CAGAC-CCGGUCCAGa
5'
|||| || | |||||||
Target:5' ttTGAGCCTCAGACCCCAGGTCa
3'</td><td>-13.99</td><td>147.00</td></tr>
<tr><td>2237~2261</td><td>25</td><td>miRNA: 3' cgaCUCCAGACC-----
CGGUCCAGa 5'
||| || ||
|||||||
Target:5' gggGAGCTC-
GGCACCCTCCAGGTCc 3'</td><td>-17.10</td><td>145.00</td></tr>
<tr><td>461</td><td>hsa-miR-632</td><td>958~976</td><td>19</td><td>miRNA: 3' agGGUGUCCUUCGUCUGUg 5'
|: ::||| ||||:||
Target:5' cgCTCTGGGATGCAGGCAt 3'</td><td>-16.90</td><td>141.00</td><td>Show result profile</td></tr>
<tr><td>462</td><td>hsa-miR-634</td><td>1927~1947</td><td>21</td><td>miRNA: 3' caggUUUCAACCCCACGACCAa
5'
:||||| |||||| |||
Target:5' ctggGAAGTT-GGGTGCCGGTc
3'</td><td>-18.20</td><td>141.00</td><td>Show result profile</td></tr>
</table>

463	hsa-miR-635	2136~2159	24	miRNA: 3' ccuguaaCAAAGUCA-CGGGUUCa<br>5' ||	|| ||||||| Target:5' gagctggGTCCCCGTGGCCCAAGt<br>3'	-15.60	148.00	Show result profile
464	hsa-miR-637	2235~2258	24	miRNA: 3' ugCGUCUCGGGCUUUCGGGGGUCa<br>5' | :||||:||:	||:|||| Target:5' aaGGGGAGCTCGGCACCCTCCAGg<br>3'	-26.90	158.00	Show result profile
		1994~2016	23	miRNA: 3' ugcGUCUCGGGCUUUCGGGGGUCa<br>5' || |||||| : ||:||||| Target:5' ggaCACAGCCCG-GTGCTCCCAGc<br>3'	-29.70	156.00	
		920~946	27	miRNA: 3' ugcgucucGGGCU--UUC-<br>GGGGGUCa 5' 	::	:		
						 Target:5'<br>cttttataCTTGGGTAAGTCCCCCAGg 3'	-19.50	150.00	
		1700~1729	30	miRNA: 3' ugCGUCUCGGGCUUUC-------<br>GGGGGUCa 5' | |||||||		
	:				 Target:5' tgGGAGAGCCC-<br>ACAGCCGTGGCCCTCCAGg 3'	-26.80	145.00	
		2329~2356	28	miRNA: 3' ugCGUCUC--GGGCU--<br>UUCGGGGGUCa 5' ||||	|||	
	:				 Target:5'<br>cgGCAGTGCCCCCCACCACCCCTCCAGc 3'	-22.84	142.00	
		2108~2130	23	miRNA: 3' ugCGUCUCGGGCUUUCGGGGGUCa<br>5' ||	||:|:	|||||:| Target:5' ctGCTG-CCCTGGCACCCCCCGGt<br>3'	-25.00	141.00	
		153~179	27	miRNA: 3' ugCGUCU-CGGGCUUU--<br>CGGGGGUCa 5' 				:		::
			:			 Target:5'<br>ttGAACATGTCGGGGATCGCCCTCAGc 3'	-20.40	140.00	
		1934~1957	24	miRNA: 3' ugcgUCUCGGGCUUUCGGGGGUCa<br>5'<br>:| |||		|:||||| Target:5' gttgGGTGCCGGTCACCTCCCAGc<br>3'	-20.00	140.00	
		2492~2511	20	miRNA: 3' ugcgucUCGGGCUUUCGGGGGUCa<br>5' ||||| ||:|||||	-21.30	140.00	

<table>
<tr><td></td><td></td><td></td><td></td><td>Target:5' atttgtAGCCC----GCTCCCAGa
3'</td><td></td><td></td><td></td></tr>
<tr><td rowspan="2">465</td><td rowspan="2">hsa-miR-638</td><td>81~107</td><td>27</td><td>miRNA: 3' ucCGGCGGUGGGCGGGC--
GCUAGGGa 5'
||||||:|| ||||
||:|||:
Target:5'
ccGCCGCCGCCGCCCCGCTCGGTCCTc 3'</td><td>-36.40</td><td>153.00</td><td rowspan="2">Show result profile</td></tr>
<tr><td>769~793</td><td>25</td><td>miRNA: 3'
ucCGGCGGUGGGCGGGCGCUAGGGa 5'
||||||:| |: :|||:||:|
Target:5'
ttGCCGCCGCGGGTGTGCGGTCTCg 3'</td><td>-32.60</td><td>147.00</td></tr>
<tr><td rowspan="2">466</td><td rowspan="2">hsa-miR-639</td><td>621~643</td><td>23</td><td>miRNA: 3' uguCGCGAGCGUUGGCGUCGCUa
5'
|||| | || |||||||
Target:5' tttGCGCCCTCATAAGCAGCGAc
3'</td><td>-18.70</td><td>160.00</td><td rowspan="2">Show result profile</td></tr>
<tr><td>2623~2646</td><td>24</td><td>miRNA: 3' ugucgcGAGC-GUUGGCGUCGCUa
5'
|||| |||:||:||:||
Target:5' atgtttCTCGCCAATCGTAGTGAc
3'</td><td>-22.70</td><td>141.00</td></tr>
<tr><td>467</td><td>hsa-miR-641</td><td>2493~2519</td><td>27</td><td>miRNA: 3' cucCACUGAGAUAGG---
AUACAGAAa 5'
|| :| | |||
|||||||
Target:5'
tttGTAGCCCGCTCCCAGAATGTCTTa 3'</td><td>-7.80</td><td>149.00</td><td>Show result profile</td></tr>
<tr><td>468</td><td>hsa-miR-642b</td><td>399~419</td><td>21</td><td>miRNA: 3' cccAGGGAGAGGUUUACACAga
5'
|::| | ||:|||||||
Target:5' ttaTTTCAC-CCGAATGTGTac
3'</td><td>-14.50</td><td>142.00</td><td>Show result profile</td></tr>
<tr><td>469</td><td>hsa-miR-646</td><td>5~28</td><td>24</td><td>miRNA: 3' cggaGUCUC-----CGUCGACGAa
5'
|||:| | |||||||
Target:5' gcgcCAGGGTCCTCGGAGCTGCTc
3'</td><td>-17.70</td><td>147.00</td><td>Show result profile</td></tr>
<tr><td>470</td><td>hsa-miR-651</td><td>478~499</td><td>22</td><td>miRNA: 3' guUUUCAGUUCGAAUAGGAUuu
5'
| | |||| | ||||||
Target:5' tcACAATCAAACAGATCCTAtt
3'</td><td>-8.20</td><td>140.00</td><td>Show result profile</td></tr>
<tr><td rowspan="2">471</td><td rowspan="2">hsa-miR-652</td><td>273~293</td><td>21</td><td>miRNA: 3' gugUUGG-GAUCACCGCGGUAa
5'
|||: ||| |||||||
Target:5' atgAACTGGGAGT-GCGCCATt
3'</td><td>-15.60</td><td>149.00</td><td rowspan="2">Show result profile</td></tr>
<tr><td>743~763</td><td>21</td><td>miRNA: 3' guguugggaucaCCGCGGUAa 5'
||||||||
Target:5' gggggttgggcgGGCGCCATc 3'</td><td>-16.70</td><td>145.00</td></tr>
</table>

		1735~1753	19	miRNA: 3' guGUUGGGAUCACCGCGGUaa 5' |:|||||	|||||| Target:5' ccCGACCCT--TAGCGCCAgg 3'	-19.70	143.00	
472	hsa-miR-654-5p	1690~1714	25	miRNA: 3' cgUGUACAAGACGC---CGGGUGgu 5'<br>:|	|||||	|||||| Target:5' gtGCTTTTTCTGGGAGAGCCCACag 3'	-15.10	140.00	Show result profile
473	hsa-miR-655	2808~2831	24	miRNA: 3' uuUCUCCAAUUGGU-ACAUA-AUa 5' | :| ||||||| ||||| || Target:5' caATGGATTAACCACTGTATCTAa 3'	-10.64	140.00	Show result profile
474	hsa-miR-658	1839~1868	30	miRNA: 3' ugGUUGCCUGGA---UGA--AGGGAGGCGg 5' ||:|	||| :|| |||||:|| Target:5' agCAGCCGTCCTGACGCTGAGCCCTCTGCa 3'	-25.20	145.00	Show result profile
475	hsa-miR-659	506~529	24	miRNA: 3' accCCUGGGAGG--GACUUGGUUc 5' ||| |:||: |||||||| Target:5' acaGGAACTTCTAAATGAACCAAa 3'	-17.70	161.00	Show result profile
476	hsa-miR-661	1273~1294	22	miRNA: 3' ugcGCGUCCGGUCUCUGGGUCCGu 5' |||:||||||: |||||| Target:5' cacCGCGGGCCAGGA--CCAGGCt 3'	-33.60	148.00	Show result profile
		1081~1105	25	miRNA: 3' ugcgcgucCGGUCUCU-GGGUCCGu 5' ||| ||| ||||||:<br>Target:5' gcttttgaGCCTCAGACCCCAGGTc 3'	-18.90	140.00	
477	hsa-miR-663	78~100	23	miRNA: 3' cgccagGGC-GCCGCGGGGCGGa 5' ||| || ||||||||:<br>Target:5' gcgccgCCGCCGCCGCCCCGCTc 3'	-29.20	148.00	Show result profile
		1380~1401	22	miRNA: 3' cgCCAGGGCGCCGCGGGGCGga 5' |||::| || |||||| Target:5' gaGGTTTCTGTGCTCCCCGCat 3'	-23.60	140.00	
478	hsa-miR-663b	1482~1501	20	miRNA: 3' ggaGUCCGUGCCGGCCCGGUGg 5' |		:|| ||||||| Target:5' cagCTGTCTTGG--GGGCCACa 3'	-16.70	147.00	Show result profile

<table>
<tr><td>479</td><td>hsa-miR-665</td><td>1873~1895</td><td>23</td><td>miRNA: 3' ucCCCGG--AGU-CGGAGGACCa 5'
||||| || |||||:|||
Target:5' ttGGGCCGGCCAGGCCTCTTGGg 3'</td><td>-26.00</td><td>148.00</td><td>Show result profile</td></tr>
<tr><td rowspan="2">480</td><td rowspan="2">hsa-miR-671-5p</td><td>1713~1736</td><td>24</td><td>miRNA: 3' gaGGU-CGGGGAGGUCCCGAAGGa 5'
||: | ||||||||||||||
Target:5' agCCGTGGCCCTCCAGGGCTTCCc 3'</td><td>-38.60</td><td>185.00</td><td rowspan="2">Show result profile</td></tr>
<tr><td>1880~1902</td><td>23</td><td>miRNA: 3' gaGGUCGGGGAGGUCCCGAAGGa 5'
|||| ||: :::||||| ||
Target:5' ggCCAGGCCTCTTGGGGCTGCCt 3'</td><td>-28.00</td><td>141.00</td></tr>
<tr><td>481</td><td>hsa-miR-675</td><td>1385~1403</td><td>19</td><td>miRNA: 3' guGACACCCGGGAGAGGCGUGGu 5'
||||| ||| |||||:|
Target:5' ttCTGTG----CTCCCCGCATCt 3'</td><td>-20.20</td><td>147.00</td><td>Show result profile</td></tr>
<tr><td>482</td><td>hsa-miR-7-1*</td><td>2429~2450</td><td>22</td><td>miRNA: 3' auaccguCUGACACUAAACAAc 5'
| :||||:||||||
Target:5' gtctgatGTTTGTGGTTTGTTt 3'</td><td>-11.80</td><td>147.00</td><td>Show result profile</td></tr>
<tr><td>483</td><td>hsa-miR-711</td><td>2126~2147</td><td>22</td><td>miRNA: 3' gaaUGCAGAGAGGGACCCAGGg 5'
::| || ||||||||
Target:5' ccgGTGGCTGGAGCTGGGTCCc 3'</td><td>-21.30</td><td>147.00</td><td>Show result profile</td></tr>
<tr><td rowspan="2">484</td><td rowspan="2">hsa-miR-744</td><td>78~99</td><td>22</td><td>miRNA: 3' acgacaauCGGGAUCGGGGCGu 5'
||| |||||||
Target:5' gcgccgccGCCGCCGCCCCGCt 3'</td><td>-17.80</td><td>146.00</td><td rowspan="2">Show result profile</td></tr>
<tr><td>2091~2112</td><td>22</td><td>miRNA: 3' acgaCAAUCGGGAUCGGGGCGu 5'
| |:| ||:||||:||
Target:5' tgggGCTGGAGCTGGCCCTGCt 3'</td><td>-21.80</td><td>142.00</td></tr>
<tr><td>485</td><td>hsa-miR-758</td><td>1576~1596</td><td>21</td><td>miRNA: 3' ccAAUCACCUGGUCCAGUGUUu 5'
| ||||| ||||||||||:
Target:5' ctTCAGTGG-CCAGGTCACAGg 3'</td><td>-27.00</td><td>163.00</td><td>Show result profile</td></tr>
<tr><td rowspan="2"></td><td rowspan="2">hsa-</td><td>2093~2114</td><td>22</td><td>miRNA: 3' acacagucaaAGUGGGACGACg 5'
| :||||||||
Target:5' gggctggagcTGGCCCTGCTGc 3'</td><td>-18.70</td><td>148.00</td><td rowspan="2"></td></tr>
<tr><td></td><td></td><td>miRNA: 3' acacagucaaaGUGGGACGACg 5'</td><td></td><td></td></tr>
</table>

<table>
<tr><td rowspan="2">486</td><td rowspan="2">miR-761</td><td>1541~1562</td><td>22</td><td>|| |||||||
Target:5' tcctatccgggCAGCCTGCTGg 3'</td><td>-17.80</td><td>147.00</td><td rowspan="2">Show result profile</td></tr>
<tr><td>886~908</td><td>23</td><td>miRNA: 3' acACAGUC--AAAGUGGGACGACg 5'
|||||| ||||| ||||||
Target:5' gaTGTCAGTATTTCA-ACTGCTGt 3'</td><td>-21.20</td><td>145.00</td></tr>
<tr><td rowspan="2">487</td><td rowspan="2">hsa-miR-762</td><td>1996~2019</td><td>24</td><td>miRNA: 3' cgaGCCGGGGC-CG-GGGUCGGGg 5'
| |||| | || ||||||||
Target:5' acaCAGCCCGGTGCTCCCAGCCCt 3'</td><td>-30.10</td><td>163.00</td><td rowspan="2">Show result profile</td></tr>
<tr><td>1229~1253</td><td>25</td><td>miRNA: 3' cgaGCCG--GGGCCG-GGGUCGGGg 5'
|||| ||| |: |||:||||
Target:5' aggCGGCCTCCCAGTGCCCGGCCCt 3'</td><td>-30.40</td><td>149.00</td></tr>
<tr><td rowspan="2">488</td><td rowspan="2">hsa-miR-766</td><td>2082~2102</td><td>21</td><td>miRNA: 3' cgacUCCGACACCCCGACCUCa 5'
|| || |||||||||||
Target:5' ttcaAGCCT-TGGGGCTGGAGc 3'</td><td>-24.00</td><td>169.00</td><td rowspan="2">Show result profile</td></tr>
<tr><td>2118~2139</td><td>22</td><td>miRNA: 3' cgacuccgacaCCCCGACCUCa 5'
| ||||||||
Target:5' ggcaccccccgGTGGCTGGAGc 3'</td><td>-17.30</td><td>147.00</td></tr>
<tr><td>489</td><td>hsa-miR-769-3p</td><td>2145~2171</td><td>27</td><td>miRNA: 3' uuGGUUCUGGG---GCCU-CUAGGGUc 5'
||: |:||| :| | |:|||||
Target:5' ccCCGTGGCCCAAGTGCAGGGTCCCAa 3'</td><td>-21.60</td><td>141.00</td><td>Show result profile</td></tr>
<tr><td>490</td><td>hsa-miR-874</td><td>2162~2184</td><td>23</td><td>miRNA: 3' agCCAGGGAGC-CCGGUCCCGUc 5'
|||||| | || ||||||:
Target:5' agGGTCCCAAGAGGGCAGGGCGg 3'</td><td>-33.00</td><td>152.00</td><td>Show result profile</td></tr>
<tr><td rowspan="2">491</td><td rowspan="2">hsa-miR-876-5p</td><td>2566~2587</td><td>22</td><td>miRNA: 3' accACUAAGUGUUUCUUUAGGu 5'
| || ||:| :|||||:|
Target:5' ggtTAATACATATGGAAATTCa 3'</td><td>-11.70</td><td>147.00</td><td rowspan="2">Show result profile</td></tr>
<tr><td>43~64</td><td>22</td><td>miRNA: 3' acCACUAAGUGUUUCUUUAGGu 5'
| | | |: |::|||:|||
Target:5' gcGGGCTCCGGAGGGAAGTCCc 3'</td><td>-14.10</td><td>140.00</td></tr>
<tr><td>492</td><td>hsa-miR-</td><td>377~398</td><td>22</td><td>miRNA: 3' acUUA-AUGAAACAUUUGGUGGu 5'</td><td>-11.80</td><td>151.00</td><td>Show result profile</td></tr>
</table>

<table>
<tr><td></td><td>876-3p</td><td></td><td></td><td>||| || || :|||||||
Target:5' aaAATGTAAATT-CGAACCACCa
3'</td><td></td><td></td><td></td></tr>
<tr><td>493</td><td>hsa-miR-877</td><td>1116~1135</td><td>20</td><td>miRNA: 3' ggGACGCGGUAGAGGAGAUg 5'
|||||||| :||||:|:
Target:5' ttCTGCGCCACTTCCTTTGt 3'</td><td>-25.80</td><td>146.00</td><td>Show result profile</td></tr>
<tr><td>494</td><td>hsa-miR-877*</td><td>1212~1232</td><td>21</td><td>miRNA: 3' gacccuccUCCCUCUUCUCCu 5'
|| ||||:||||
Target:5' tcattgaaAGTGAGAGGAGGc 3'</td><td>-14.50</td><td>141.00</td><td>Show result profile</td></tr>
<tr><td>495</td><td>hsa-miR-885-3p</td><td>2096~2116</td><td>21</td><td>miRNA: 3' auaggUGAUGUGGGGCGACGGa
5'
:||: :|||:||||||
Target:5' ctggaGCTG-GCCCTGCTGCCc
3'</td><td>-22.20</td><td>144.00</td><td>Show result profile</td></tr>
<tr><td>496</td><td>hsa-miR-888*</td><td>872~895</td><td>24</td><td>miRNA: 3' aaGUGGGUUUCU--CCACAGUCAg
5'
:|::: || | | |||||||
Target:5' ttTATTTTAATATTGATGTCAGTa
3'</td><td>-8.30</td><td>150.00</td><td>Show result profile</td></tr>
<tr><td>497</td><td>hsa-miR-892a</td><td>1952~1971</td><td>20</td><td>miRNA: 3' gaugCGUCUUUCCUGUGUCAc 5'
||||:|| |:||||||
Target:5' cccaGCAGGAA-GGCACAGTg 3'</td><td>-25.10</td><td>152.00</td><td>Show result profile</td></tr>
<tr><td rowspan="3">498</td><td rowspan="3">hsa-miR-9</td><td>502~531</td><td>30</td><td>miRNA: 3' agUAUGUC------GAUCUA-
UUGGUUUcu 5'
|||||| ||| ||
|||||||
Target:5'
gaATACAGGAACTTCTAAATGAACCAAAta 3'</td><td>-9.40</td><td>151.00</td><td rowspan="3">Show result profile</td></tr>
<tr><td>502~531</td><td>30</td><td>miRNA: 3' agUAUGUC------GAUCUA-
UUGGUUUcu 5'
|||||| ||| ||
|||||||
Target:5'
gaATACAGGAACTTCTAAATGAACCAAAta 3'</td><td>-9.40</td><td>151.00</td></tr>
<tr><td>502~531</td><td>30</td><td>miRNA: 3' agUAUGUC------GAUCUA-
UUGGUUUcu 5'
|||||| ||| ||
|||||||
Target:5'
gaATACAGGAACTTCTAAATGAACCAAAta 3'</td><td>-9.40</td><td>151.00</td></tr>
<tr><td>499</td><td>hsa-miR-920</td><td>2400~2421</td><td>22</td><td>miRNA: 3' auGACGAAGGUG--UCGAGGGg
5'
|||| :||| |||||:|
Target:5' ccCTGCAGTCACTGAGCTCTCg
3'</td><td>-17.70</td><td>144.00</td><td>Show result profile</td></tr>
<tr><td>500</td><td>hsa-miR-92a-1*</td><td>2014~2040</td><td>27</td><td>miRNA: 3' ucguaACGUUGG----
CUAGGGUUGGa 5'
| ||||| |
||||||||
Target:5'
agcccTCCAACCTCTGGCTCCCAACCc 3'</td><td>-19.70</td><td>160.00</td><td>Show result profile</td></tr>
</table>

<table>
<tr><td rowspan="2">501</td><td rowspan="2">hsa-miR-92a-2*</td><td>1239~1261</td><td>23</td><td>miRNA: 3' caUUAC--GUUGUUUAGGGGUGGg 5'
          |:|| |::| ||||||||
Target:5' ccAGTGCCCGGC-CCTCCCCACCc 3'</td><td>-22.30</td><td>149.00</td><td rowspan="2">Show result profile</td></tr>
<tr><td>2326~2346</td><td>21</td><td>miRNA: 3' cauuaCGUUGUUUAGGGGUGGg 5'
             |||:: : |||||||
Target:5' agccgGCAGT-GCCCCCCACCa 3'</td><td>-19.50</td><td>144.00</td></tr>
<tr><td rowspan="2">502</td><td rowspan="2">hsa-miR-92b*</td><td>1618~1643</td><td>26</td><td>miRNA: 3' gugAC-GUGGCG--CA-GGGCAGGGa 5'
          || |||| | || :|:|||||
Target:5' gggTGCCACCTCAGGTGTCTGTCCCt 3'</td><td>-21.50</td><td>141.00</td><td rowspan="2">Show result profile</td></tr>
<tr><td>1828~1851</td><td>24</td><td>miRNA: 3' gugACGUGGCGCAG--GGCAGGGa 5'
          |||:|| | | ||||||:
Target:5' ctgTGCGCCTCAGCAGCCGTCCTg 3'</td><td>-19.60</td><td>141.00</td></tr>
<tr><td>503</td><td>hsa-miR-93</td><td>982~1004</td><td>23</td><td>miRNA: 3' gaUGG-ACGUGCUUGUCGUGAAac 5'
          ||| |||| ||:| ||||||
Target:5' tcACCGTGCA-GAGCTGCACTTgg 3'</td><td>-19.80</td><td>152.00</td><td>Show result profile</td></tr>
<tr><td rowspan="2">504</td><td rowspan="2">hsa-miR-93*</td><td>1821~1844</td><td>24</td><td>miRNA: 3' gcCCUUC-ACGAUC-GAGUCGUCa 5'
          |||:| ||: | ||||||||
Target:5' ggGGAGGCTGTGCGCCTCAGCAGc 3'</td><td>-24.50</td><td>160.00</td><td rowspan="2">Show result profile</td></tr>
<tr><td>163~182</td><td>20</td><td>miRNA: 3' gcCCUUCACGAUCGAGUCGUCa 5'
          ||:| || ||||||||
Target:5' cgGGGATCGC--CCTCAGCAGa 3'</td><td>-22.00</td><td>152.00</td></tr>
<tr><td>505</td><td>hsa-miR-933</td><td>16~40</td><td>25</td><td>miRNA: 3' cccUCUCCA-GAGG--GACGCGUGu 5'
          :||| | |||: ||||||:|
Target:5' ctcGGAGCTGCTCTGGCTGCGCGCg 3'</td><td>-23.60</td><td>145.00</td><td>Show result profile</td></tr>
<tr><td>506</td><td>hsa-miR-937</td><td>16~43</td><td>28</td><td>miRNA: 3' ccGUCUC--UCAGUCU----CGCGCCUa 5'
          |:||| : || |: |||||||
Target:5' ctCGGAGCTGCTCTGGCTGCGCGCGGAg 3'</td><td>-18.60</td><td>148.00</td><td>Show result profile</td></tr>
<tr><td></td><td></td><td>2164~2193</td><td>30</td><td>miRNA: 3' guGGGGGUCU--CG----GAGUCGAGGGGu 5'
          :||| ||| || | :|||||||
Target:5' ggTCCCAAGAGGGCAGGGCGGGGCTCCCCa 3'</td><td>-27.41</td><td>154.00</td><td></td></tr>
</table>

<table>
<tr><td rowspan="7">507</td><td rowspan="7">hsa-miR-939</td><td>1372~1398</td><td>27</td><td>miRNA: 3' gugGGGGUCUC---
GGAGUCGAGGGGu 5'
|: | ||| :||
|||||||
Target:5'
gtgCTGCTGAGGTTTCTGTGCTCCCCg 3'</td><td>-24.70</td><td>153.00</td><td rowspan="7">Show result profile</td></tr>
<tr><td>2007~2037</td><td>31</td><td>miRNA: 3' guGGGGGUC------UCGGAG-
UCGAGGGGu 5'
|:||||| | ||||
:||||||
Target:5'
tgCTCCCAGCCCTCCAACCTCTGGCTCCCaa 3'</td><td>-28.30</td><td>148.00</td></tr>
<tr><td>937~959</td><td>23</td><td>miRNA: 3' guGGGGGUCUCGGAGUCGAGGGGu
5'
|||||||:| | ||:|||:|
Target:5' gtCCCCCAGGGGC-GAGTTCCTCg
3'</td><td>-33.40</td><td>145.00</td></tr>
<tr><td>1706~1737</td><td>32</td><td>miRNA: 3' guGGGGGUC----UC-GGAG---
UCGAGGGGu 5'
||| ||| :| ||||
:|||:|||
Target:5'
agCCCACAGCCGTGGCCCTCCAGGGCTTCCCc 3'</td><td>-30.90</td><td>144.00</td></tr>
<tr><td>2317~2343</td><td>27</td><td>miRNA: 3' guGGGGGUCUCGG-AGU--
CGAGGGGu 5'
||||| ||||| || ||
||||
Target:5'
ggCCCCCCGAGCCGGCAGTGCCCCCCa 3'</td><td>-35.10</td><td>144.00</td></tr>
<tr><td>1277~1299</td><td>23</td><td>miRNA: 3' guggGGUCUCGGAGUCGAGGGGu
5'
||||: || :||||:||
Target:5' gcgggCCAGGACC-AGGCTCTCCa
3'</td><td>-23.90</td><td>142.00</td></tr>
<tr><td>1234~1258</td><td>25</td><td>miRNA: 3' guGGGGGUCUCGGAGUC--
GAGGGGu 5'
|:||||| ||| |:|
||||||
Target:5' gcCTCCCAGTGCC-
CGGCCCTCCCCa 3'</td><td>-34.10</td><td>141.00</td></tr>
<tr><td rowspan="3">508</td><td rowspan="3">hsa-miR-941</td><td>107~133</td><td>27</td><td>miRNA: 3' cgUGUACACGUG-UGU---
CGGCCCac 5'
|| ||| |:| ||:
||||||
Target:5'
ccACCTGTCCGCTACGCTCGCCGGGgc 3'</td><td>-22.40</td><td>141.00</td><td rowspan="3">Show result profile</td></tr>
<tr><td>107~133</td><td>27</td><td>miRNA: 3' cgUGUACACGUG-UGU---
CGGCCCac 5'
|| ||| |:| ||:
||||||
Target:5'
ccACCTGTCCGCTACGCTCGCCGGGgc 3'</td><td>-22.40</td><td>141.00</td></tr>
<tr><td></td><td></td><td>miRNA: 3' cgUGUACACGUG-UGU---</td><td></td><td></td></tr>
</table>

<table>
<tr><td rowspan="2"></td><td rowspan="2"></td><td>107~133</td><td>27</td><td>CGGCCCac 5'
|| ||| |:| ||:
||||||
Target:5'
ccACCTGTCCGCTACGCTCGCCGGGgc 3'</td><td>-22.40</td><td>141.00</td><td rowspan="2"></td></tr>
<tr><td>107~133</td><td>27</td><td>miRNA: 3' cgUGUACACGUG-UGU---
CGGCCCac 5'
|| ||| |:| ||:
||||||
Target:5'
ccACCTGTCCGCTACGCTCGCCGGGgc 3'</td><td>-22.40</td><td>141.00</td></tr>
<tr><td rowspan="3">509</td><td rowspan="3">hsa-miR-942</td><td>56~79</td><td>24</td><td>miRNA: 3' guguaccGGUUUUG--UCUCUUCu
5'
||:|:|| ||:||||
Target:5' ggaagtcCCGAGACAAAGGGAAGc
3'</td><td>-14.60</td><td>141.00</td><td rowspan="3">Show result profile</td></tr>
<tr><td>435~456</td><td>22</td><td>miRNA: 3' guGUACCGGUUUUGUCUCUUCu
5'
| || ||| :||||:||
Target:5' tgCCTGTCCATCTTAGAGGAGg
3'</td><td>-15.80</td><td>140.00</td></tr>
<tr><td>1211~1231</td><td>21</td><td>miRNA: 3' guGUACCGGUUUUG-UCUCUUCu
5'
||| | |||:: ||||:||
Target:5' atCATTG--AAAGTGAGAGGAGg
3'</td><td>-10.90</td><td>140.00</td></tr>
<tr><td>510</td><td>hsa-miR-96</td><td>2351~2373</td><td>23</td><td>miRNA: 3' ucGUU-UUUACACGAUCACGGUUu
5'
||: :| | ||| :||||||:
Target:5' tcCAGCGACTCTGC-GGTGCCAGt
3'</td><td>-15.90</td><td>140.00</td><td>Show result profile</td></tr>
<tr><td>511</td><td>hsa-miR-99a*</td><td>2043~2064</td><td>22</td><td>miRNA: 3' gucuGGGUAUCUUCGCUCGAAc
5'
||||| |||||||||
Target:5' tctcCCCATCCTAGCGAGCTTg
3'</td><td>-24.50</td><td>166.00</td><td>Show result profile</td></tr>
<tr><td>512</td><td>hsa-miR-99b*</td><td>2043~2064</td><td>22</td><td>miRNA: 3' gccuGGGUGUCUGUGCUCGAAc
5'
||||: :|||||||
Target:5' tctcCCCATCCTAGCGAGCTTg
3'</td><td>-20.90</td><td>150.00</td><td>Show result profile</td></tr>
</table>

miRNA Target Sites Table View

<table>
<tr><td>Target Sequence</td><td>>
AGATTGCAGAGGGAGACGTGGACGTGAGTGGAGCGGGGCGGTCCCCAGCACACTAGAGGAAGTCGTGCTACCCCCGCGGA
GTTGTCGTGTGTTCTGGATTCATTCCGGCACCACCATGTCGAAGGTTTCCTTTAAGATCACGCTGACGTCGGACCCACGG
CTGCCGTACAAAGTACTCAGTGTTCCTGAAAGTACACCTTTCACAGCAGTCTTAAAGTTTGCAGCAGAAGAATTTAAAGT
TCCTGCTGCAACAAGTGCAATTATTACCAATGATGGAATAGGAATAAATCCTGCACAGACTGCTGGAAATGTTTTTCTAA
AACATGGTTCAGAACTGCGGATTATTCCTAGAGATCGTGTTGGAAGTTGTTAATATCTGCTACTTGGAACATACGATTGC
CTTTCAGAATAAATATTGGTATTTTTTGTTGTTGTAAAATTGAAATCAGGCATTTAACATACTATGAAAACACCAGGAGT
CAATGATTAATGAAAGGTGACTCATCTGTCCCTTTTTGTTGTCCATACTCTTCCTATGAAGAGGGAATGCGTATGAATTA
AGGCTACTACTGTCACAGAAGATCATAGTCTTTGATGCTACCTCACAACACAAACAGGTAGTTCGTTGGGGGCAAATGAA
TTAGCCAACTGTTAACTGGAAGCTTTTGATAATTTTTTTTTTTAGAACAATTTGGAACATTAAAATTTACTGAATCGTAT
ATATTCATCTGAGATAAAAATATAAAAAGAATTATGGACCCTGGATGGCAATTTGCTTGATAGCATCTGATTTGCAGACT
CATAATTTGATTTTTAATTAAATATATAGGTTATGATGAAGTGAATAGACATATCAGTGAACAGTTAACTATATTAAATT
TTTATCATTTACTTTTTTTAAGATTCAGACCTCAGTTATATAAATTTCAGTTTAATATCAACCAAAAAATTAAAATTTTA
ATCTAACCCTTATGTGTATAAATTGGTGTCCCATACCAGCTTTTAATGGTGGACCTATAGAATCCAGTACTTTTAATGGT
GGGAATTTACAGTAGAAGCATCCTTTGCTGAGTTATACATTCCTTTATCAATCTCTTTTGATACAACATTTAAAACAAGT
AGCTTCAAGAAACCACTGGTGTTTTGAGGATAGTATTTCTAAATAGCATTCAGGAACAGAGTATTATTGCACAGATCTGA
AGATCAAAAAAAAGCTCAAGGAAATACAGATCGGAAGTGCTGATGAGTTATATTTATTGAAAACCCAACTTTTAAGGAAG
TGCTAAGATCAGTCACCCATGTGAATAAGAAGCCAGGAAAGGAAAGATGGGGAAGCCCAGATCACCAGGCTTCTATTAAG
GAGGAAAGCAACAGAGGAAACAGTGAAGGGGAACAGAAGGGGGTAGCAAAGTGTTACAGAAAAGCGGACTGGATAGACAA
AACTGCAGAAGGTGTATGTTGGGGAGAACTGAAAGGGAAAACAAAATACTTGACATAGTCTTAAGTAGAAGAAGGCAGTT
AGAGAAAACAAAGTATCTACTGGCCTTGTCAACATACAGACTTCAAAATACCCCTTATGAGAATCCAAAGAATGATGTGT
GTAAGGGAAGATTTTATTTGCCCTTCCGGAAGAAATCAGTATCTATGCAAATCTTGAAAGACGAAATCAAAGCCCATTAA
TGATTCAGAATCAGTGCTTGACCTCCTGTATTCTGAATGGTGAACTCTGGAAGCAGGGATTGTGTCTGGCTCTTTTTAGA
GCTGGAAATGTAGTGGCTTTCATTAAATACTTGCTGTAAAGTCTTTCTAAGACCAATTATTATCTTAGCATGTTTCAGTA
TCTTCTCTATCATAGGCCCTAAGTTCATTGGGGGAAAAAAAATAAGAAGATTCAACAGAATCAGCATTTGAAGTGTACCA
TTGGTAGTTGTTTATGAAATTACCAGATATTCATAAATGTGACAAATGAACAGCAGGATTATGAATTATCAAAGGAAAAA
GTATTTGCTGAGGTGAAAAAATCTGATGTTTGAGGAAGTTTTTATTTTTATTTATTTGTTTTGTTTTTTTTTTTTTGAGG
CAGACTCTCTGTCGCCAGGCTTCTCCTGCCTCAGCCTCACGAGTAGCTGGGACTACAGGCATGCACCACCACGCCCAGCT
AATTTTTGTATTTTTAGCAGAGACAGGGAGGAAGTTTTTATTTTTATAAACAAAATCTGATGTTCAGAGGCCCCGTTTCT
TACAATAAATGTTGAGTCTTAGTTAAGCAGGAATTTATGAACACCCATTTCCTGACTTTTTGCTTTAATTTAGATTTTTC
TCCATCCTGTTTCTAGCACAAAAATTTGCCTGCTGTGTTACAAAAATAAATTTATGTTGTCCTGTGCCATAAAGTGATAT
ATTTAATATTTTTATTCTTTGGTTTTGAACATTGTAAGTTCCTAAAAACATTTTATTAAACAAGTAGACATTTTGTTATT
AAAAAAATGTGATCTGTAATTTCTTTGTGCAGAATGATTTGAAGTATTGTATTCAGTTTACATGCGTTATTGGTTTATAA
TTAATATCTAATGAAAATACATGTTGTTATATTGTAAACCAAAAAAAAAAAAAAAAAA</td></tr>
</table>

<table>
<tr><th>No.</th><th>miRNA ID</th><th>Location</th><th>Len</th><th>Hybridization</th><th>Minimum Free Energy</th><th>Score</th><th>Profile</th></tr>
<tr><td rowspan="3">1</td><td rowspan="3">hsa-let-7a</td><td>578~605</td><td>28</td><td>miRNA: 3' uugaUA-UGUUGG-----
AUGAUGGAGu 5'
|| |:|::|
|:|||||||
Target:5'
gaagATCATAGTCTTTGATGCTACCTCa 3'</td><td>-15.60</td><td>150.00</td><td rowspan="3">Show result profile</td></tr>
<tr><td>578~605</td><td>28</td><td>miRNA: 3' uugaUA-UGUUGG-----
AUGAUGGAGu 5'
|| |:|::|
|:|||||||
Target:5'
gaagATCATAGTCTTTGATGCTACCTCa 3'</td><td>-15.60</td><td>150.00</td></tr>
<tr><td>578~605</td><td>28</td><td>miRNA: 3' uugaUA-UGUUGG-----
AUGAUGGAGu 5'
|| |:|::|
|:|||||||
Target:5'
gaagATCATAGTCTTTGATGCTACCTCa 3'</td><td>-15.60</td><td>150.00</td></tr>
<tr><td></td><td>hsa-let-</td><td></td><td></td><td>miRNA: 3' uugguGUGUUGG-----
AUGAUGGAGu 5'</td><td></td><td></td><td></td></tr>
</table>

<table>
<tr><td>2</td><td>7b</td><td>579~605</td><td>27</td><td>||:|::|
|:|||||||
Target:5'
aagatCATAGTCTTTGATGCTACCTCa 3'</td><td>-18.50</td><td>153.00</td><td>Show result profile</td></tr>
<tr><td>3</td><td>hsa-let-7c</td><td>578~605</td><td>28</td><td>miRNA: 3' uuggUA-UGUUGG-----
AUGAUGGAGu 5'
|| |:|::|
|:|||||||
Target:5'
gaagATCATAGTCTTTGATGCTACCTCa 3'</td><td>-17.22</td><td>150.00</td><td>Show result profile</td></tr>
<tr><td>4</td><td>hsa-let-7d</td><td>588~605</td><td>18</td><td>miRNA: 3' uuGAUACGUUGGAUGAUGGAGa
5'
|| || | |:|||||||
Target:5' gtCTTTG--A--TGCTACCTCa
3'</td><td>-12.70</td><td>148.00</td><td>Show result profile</td></tr>
<tr><td rowspan="2">5</td><td rowspan="2">hsa-let-7e</td><td>578~605</td><td>28</td><td>miRNA: 3' uugaUA-UGUUGGAG-----
GAUGGAGu 5'
|| |:|::||:
|||||||
Target:5'
gaagATCATAGTCTTTGATGCTACCTCa 3'</td><td>-15.10</td><td>150.00</td><td rowspan="2">Show result profile</td></tr>
<tr><td>2087~2113</td><td>27</td><td>miRNA: 3' uuGAUA--UGUU---
GGAGGAUGGAGu 5'
||:| ||:
:|||||:||||
Target:5'
ctCTGTCGCCAGGCTTCTCCTGCCTCa 3'</td><td>-21.80</td><td>142.00</td></tr>
<tr><td rowspan="2">6</td><td rowspan="2">hsa-let-7f</td><td>578~605</td><td>28</td><td>miRNA: 3' uugaUA-UGUUAG-----
AUGAUGGAGu 5'
|| |:|:||
|:|||||||
Target:5'
gaagATCATAGTCTTTGATGCTACCTCa 3'</td><td>-16.30</td><td>154.00</td><td rowspan="2">Show result profile</td></tr>
<tr><td>578~605</td><td>28</td><td>miRNA: 3' uugaUA-UGUUAG-----
AUGAUGGAGu 5'
|| |:|:||
|:|||||||
Target:5'
gaagATCATAGTCTTTGATGCTACCTCa 3'</td><td>-16.30</td><td>154.00</td></tr>
<tr><td>7</td><td>hsa-let-7g</td><td>586~605</td><td>20</td><td>miRNA: 3' uugACAUGUUUGAUGAUGGAGu
5'
| | ::| |:|||||||
Target:5' tagTCTTTGA--TGCTACCTCa
3'</td><td>-13.90</td><td>147.00</td><td>Show result profile</td></tr>
<tr><td>8</td><td>hsa-let-7i</td><td>579~605</td><td>27</td><td>miRNA: 3' uugUCGUGUUUG-----
AUGAUGGAGu 5'
| ||:|: |
|:|||||||
Target:5'
aagATCATAGTCTTTGATGCTACCTCa 3'</td><td>-16.60</td><td>151.00</td><td>Show result profile</td></tr>
<tr><td></td><td></td><td>1056~1084</td><td>29</td><td>miRNA: 3' uaUGUAUGAAG------AA-
AUGUAAGGu 5'
:||| |||: ||
||||||||
Target:5'
aaGCATCCTTTGCTGAGTTATACATTCCt 3'</td><td>-11.30</td><td>158.00</td><td></td></tr>
</table>

<table>
<tr><td rowspan="2">9</td><td rowspan="2">hsa-miR-1</td><td></td><td></td><td></td><td></td><td></td><td rowspan="2">Show result profile</td></tr>
<tr><td>1056~1084</td><td>29</td><td>miRNA: 3' uaUGUAUGAAG------AA-
AUGUAAGGu 5'
            :||| |||: ||
||||||||
Target:5'
aaGCATCCTTTGCTGAGTTATACATTCCt 3'</td><td>-11.30</td><td>158.00</td></tr>
<tr><td>10</td><td>hsa-miR-103-2*</td><td>1289~1314</td><td>26</td><td>miRNA: 3' guUCCGUCGUGACAU---
UUCUUCGa 5'
            || || | |||:
|||||||
Target:5'
tcAGTCACCCATGTGAATAAGAAGCc 3'</td><td>-13.00</td><td>157.00</td><td>Show result profile</td></tr>
<tr><td rowspan="2">11</td><td rowspan="2">hsa-miR-105</td><td>1892~1911</td><td>20</td><td>miRNA: 3' uggUGUCCUCAGACUCGUAAACu
5'
             ||| || || ||||||||
Target:5' tcaACA-GAATC--AGCATTTGa
3'</td><td>-12.20</td><td>159.00</td><td rowspan="2">Show result profile</td></tr>
<tr><td>1892~1911</td><td>20</td><td>miRNA: 3' uggUGUCCUCAGACUCGUAAACu
5'
             ||| || || ||||||||
Target:5' tcaACA-GAATC--AGCATTTGa
3'</td><td>-12.20</td><td>159.00</td></tr>
<tr><td>12</td><td>hsa-miR-1179</td><td>529~552</td><td>24</td><td>miRNA: 3' gguuGGUUACUU---UCUUACGaa
5'
              || ||||| :||||||
Target:5' tcttCCTATGAAGAGGGAATGCgt
3'</td><td>-13.60</td><td>141.00</td><td>Show result profile</td></tr>
<tr><td>13</td><td>hsa-miR-1182</td><td>1838~1861</td><td>24</td><td>miRNA: 3' caguGUAGGGAGGGU-UCUGGGAg
5'
              | ||:|| :|| ||:||||
Target:5' gtatCTTCTCTATCATAGGCCCTa
3'</td><td>-18.00</td><td>147.00</td><td>Show result profile</td></tr>
<tr><td rowspan="2">14</td><td rowspan="2">hsa-miR-1183</td><td>2108~2140</td><td>33</td><td>miRNA: 3' acGGGUGAGAGUGGU-AGUG----
-GAUGUCac 5'
            |:|| ||||| | | :|
||||||
Target:5'
gcCTCAGCCTCACGAGTAGCTGGGACTACAGgc
3'</td><td>-18.80</td><td>141.00</td><td rowspan="2">Show result profile</td></tr>
<tr><td>1024~1054</td><td>31</td><td>miRNA: 3' acggGUGAGAGUGGUAG----
UGGAUGUCAc 5'
              :|||:|:| :: :
|::||||||
Target:5'
ccagTACTTTTAATGGTGGGAATTTACAGTa 3'</td><td>-12.74</td><td>141.00</td></tr>
<tr><td>15</td><td>hsa-miR-1197</td><td>77~96</td><td>20</td><td>miRNA: 3' ucUUCAUCUGGUACACAGGAu 5'
            :||| | :|:|||||:||
Target:5' cgGAGTTG-TCGTGTGTTCTg 3'</td><td>-19.80</td><td>146.00</td><td>Show result profile</td></tr>
<tr><td>16</td><td>hsa-miR-1205</td><td>278~297</td><td>20</td><td>miRNA: 3' gagUUUCGUUUGGGACGUcu 5'
             ::| :|||:||||||
Target:5' ataGGAATAAATCCTGCAca 3'</td><td>-12.80</td><td>141.00</td><td>Show result profile</td></tr>
</table>

<table>
<tr><td>17</td><td>hsa-miR-1206</td><td>2261~2283</td><td>23</td><td>miRNA: 3' cgAAUUUGU--AGAUGUACUUGu 5'
||||:|| | :||||||
Target:5' agTTAAGCAGGAATTTATGAACa 3'</td><td>-12.30</td><td>141.00</td><td>Show result profile</td></tr>
<tr><td>18</td><td>hsa-miR-1207-5p</td><td>2090~2111</td><td>22</td><td>miRNA: 3' ggGGAGGGUCGGA-GGGACGGu 5'
:| || :||:| :||||||
Target:5' tgTCGCCAGGCTTCTCCTGCCt 3'</td><td>-23.10</td><td>143.00</td><td>Show result profile</td></tr>
<tr><td>19</td><td>hsa-miR-1208</td><td>1367~1386</td><td>20</td><td>miRNA: 3' aggcggacagacUUGUCACu 5'
|||||||
Target:5' agcaacagaggaAACAGTGa 3'</td><td>-9.72</td><td>140.00</td><td>Show result profile</td></tr>
<tr><td>20</td><td>hsa-miR-1236</td><td>2175~2197</td><td>23</td><td>miRNA: 3' gaCCUCUCUGUUCC-CCUUCucc 5'
| |||||||:|| |||||
Target:5' taGCAGAGACAGGGAGGAAGttt 3'</td><td>-23.32</td><td>140.00</td><td>Show result profile</td></tr>
<tr><td rowspan="2">21</td><td rowspan="2">hsa-miR-1237</td><td>1379~1401</td><td>23</td><td>miRNA: 3' gaccCCCUGCCUC--GUCUUCCu 5'
| || ||:| |||||||
Target:5' aacaGTGAAGGGGAACAGAAGGg 3'</td><td>-19.60</td><td>155.00</td><td rowspan="2">Show result profile</td></tr>
<tr><td>1426~1453</td><td>28</td><td>miRNA: 3' gacccCCUGCCU-------CGUCUUCCu 5'
|||: ||
||||||||
Target:5' ggactGGATAGACAAAACTGCAGAAGGt 3'</td><td>-19.60</td><td>148.00</td></tr>
<tr><td rowspan="3">22</td><td rowspan="3">hsa-miR-124*</td><td>2265~2285</td><td>21</td><td>miRNA: 3' uaguUCCAGGCGACACUUGUGc 5'
||| : :| |||||||
Target:5' aagcAGGAAT-TTATGAACACc 3'</td><td>-8.40</td><td>145.00</td><td rowspan="3">Show result profile</td></tr>
<tr><td>2265~2285</td><td>21</td><td>miRNA: 3' uaguUCCAGGCGACACUUGUGc 5'
||| : :| |||||||
Target:5' aagcAGGAAT-TTATGAACACc 3'</td><td>-8.40</td><td>145.00</td></tr>
<tr><td>2265~2285</td><td>21</td><td>miRNA: 3' uaguUCCAGGCGACACUUGUGc 5'
||| : :| |||||||
Target:5' aagcAGGAAT-TTATGAACACc 3'</td><td>-8.40</td><td>145.00</td></tr>
<tr><td>23</td><td>hsa-miR-1243</td><td>1010~1029</td><td>20</td><td>miRNA: 3' gugaGGAUAUUAACUAGGUCAa 5'
||||||: |||||||
Target:5' tggaCCTATAG--AATCCAGTa 3'</td><td>-15.60</td><td>158.00</td><td>Show result profile</td></tr>
<tr><td></td><td>hsa-</td><td></td><td></td><td>miRNA: 3' uaCAUCC---GGAAA-</td><td></td><td></td><td></td></tr>
</table>

24	miR-1245	119~143	25	UCUAGUGaa 5'<br>| ||| ||||| ||||||| Target:5'<br>tcGAAGGTTTCCTTTAAGATCACgc 3'	-14.80	147.00	Show result profile
25	hsa-miR-1248	1489~1516	28	miRNA: 3' aaAUCGUGUCACGAAU--<br>AUGUUCUUCCa 5'<br>|: ||:||| |||| || ||||||| Target:5' ctTGACATAGT-<br>CTTAAGTAGAAGAAGGc 3'	-18.60	178.00	Show result profile
		1338~1365	28	miRNA: 3' aaaUCGU-GUCACGAAUAU-<br>GUUCUUCCa 5'<br>| || ||| |||| || :|||:||| Target:5' cagATCACCAG-<br>GCTTCTATTAAGGAGGa 3'	-16.60	155.00	
		519~546	28	miRNA: 3' aaaucGUGUCACGA--<br>AUAUGUUCUUCCa 5'<br>||:| | :| |||:<br>||||:|| Target:5' ttgtcCATACTCTTCCTATG-<br>AAGAGGGa 3'	-9.80	143.00	
26	hsa-miR-1251	1505~1525	21	miRNA: 3' ucgcggaaaCCGUCGAUCUCa 5'<br>|||||:||||| Target:5' gtagaagaaGGCAGTTAGAGa 3'	-17.70	144.00	Show result profile
27	hsa-miR-1252	1065~1087	23	miRNA: 3' auuuACUUAA-GUUAAAGGAAGa<br>5'<br>|||:|| :| ||||||:<br>Target:5' ttgcTGAGTTATACATTCCTTTa<br>3'	-7.70	142.00	Show result profile
28	hsa-miR-1253	1827~1848	22	miRNA: 3' acGU-CCGACUAGAAGAAGAGa<br>5'<br>|| | :| | : ||||||| Target:5' agCATGTTTCAGTATCTTCTCt<br>3'	-12.00	147.00	Show result profile
29	hsa-miR-1257	872~893	22	miRNA: 3' ccAGUCUU--GGGUAGUAAGUGa<br>5'<br>|:| || :::||||||:|| Target:5' taTTA-AATTTTTATCATTTACt<br>3'	-10.80	140.00	Show result profile
30	hsa-miR-1260	1028~1045	18	miRNA: 3' accaccgucuCCACCCUa 5'<br>||||||| Target:5' tacttttaatGGTGGGAa 3'	-11.10	140.00	Show result profile
31	hsa-miR-1260b	1027~1045	19	miRNA: 3' uaccaccGUCACCACCCUa 5'<br>:| |||||||| Target:5' gtactttTAATGGTGGGAa 3'	-13.10	148.00	Show result profile
32	hsa-miR-1262	1277~1300	24	miRNA: 3' uaggAAGAUGUUUA---<br>AGUGGGUa 5'<br>| ||| |:|| ||||||| Target:5' gaagTGCTA-<br>AGATCAGTCACCCAt 3'	-13.30	153.00	Show result profile

33	hsa-miR-1264	1592~1615	24	miRNA: 3' uugUCCACG-AGUUUAUUCUGAAc 5'<br>| |||: | |:: ||||:|| Target:5' atgATGTGTGTAAGGGAAGATTTt 3'	-9.00	140.00	Show result profile
34	hsa-miR-1267	1877~1899	23	miRNA: 3' accccUAAUGU--GAAGUUGUCc 5'<br>|| | | |||||||| Target:5' aaaaaATAAGAAGATTCAACAGa 3'	-8.00	146.00	Show result profile
35	hsa-miR-1268	2140~2157	18	miRNA: 3' ggggGUGGUGGUGCGGGc 5'<br>|||||||||||| Target:5' catgCACCACCACGCCCa 3'	-28.30	170.00	Show result profile
36	hsa-miR-127-5p	1107~1128	22	miRNA: 3' uagucucgggagacUCGAAGUc 5'<br>||||||| Target:5' catttaaaacaagtAGCTTCAa 3'	-9.00	140.00	Show result profile
37	hsa-miR-1273	2075~2097	23	miRNA: 3' uuCUUUCUCAGAACGAAACAGCGGg 5'<br>||:: || || || ||||||| Target:5' ttGAGGCAGACT--CTCTGTCGCCa 3'	-16.90	163.00	Show result profile
38	hsa-miR-1274a	2172~2190	19	miRNA: 3' accgcgGACU-UGUCCCUg 5'<br>| || ||||||| Target:5' ttttagCAGAGACAGGGAg 3'	-12.40	144.00	Show result profile
39	hsa-miR-1276	1737~1756	20	miRNA: 3' acagaggugucCCGAGAAAu 5'<br>|||||||| Target:5' ggattgtgtctGGCTCTTTt 3'	-13.20	145.00	Show result profile
40	hsa-miR-1278	1005~1032	28	miRNA: 3' uauCUACU---AUA-CGU--GUCAUGAu 5'<br>|:||: ||| | | ||||||| Target:5' aatGGTGGACCTATAGAATCCAGTACTt 3'	-10.20	149.00	Show result profile
41	hsa-miR-1285	506~527	22	miRNA: 3' ucCAGAGUGAAACAACGGGUcu 5'<br>||| | :|||||||:||| Target:5' ctGTCCCTTTTTGTTGTCCAta 3'	-16.60	144.00	Show result profile
		506~527	22	miRNA: 3' ucCAGAGUGAAACAACGGGUcu 5'<br>||| | :|||||||:||| Target:5' ctGTCCCTTTTTGTTGTCCAta 3'	-16.60	144.00	
42	hsa-miR-1286	2367~2386	20	miRNA: 3' ucccGAGUAGAACCAGGACGu 5'<br>:|:|| || ||||||:<br>Target:5' taaaTTTATGTT-GTCCTGTg 3'	-12.30	140.00	Show result profile

<table>
<tr><td>43</td><td>hsa-miR-1288</td><td>25~45</td><td>21</td><td>miRNA: 3' agagGUCUAGUCCCGUCAGGu 5'
::|| |:||||:||||
Target:5' tgagTGGAGCGGGGCGGTCCc 3'</td><td>-21.90</td><td>149.00</td><td>Show result profile</td></tr>
<tr><td>44</td><td>hsa-miR-1294</td><td>587~607</td><td>21</td><td>miRNA: 3' ucuguuGUUACGGUUGGAGUGu 5'
::||||: |||||||
Target:5' agtcttTGATGCT-ACCTCACa 3'</td><td>-17.10</td><td>155.00</td><td>Show result profile</td></tr>
<tr><td>45</td><td>hsa-miR-1296</td><td>1841~1862</td><td>22</td><td>miRNA: 3' ccucuaccucggucCCGGGAUu 5'
|||||||
Target:5' tcttctctatcataGGCCCTAa 3'</td><td>-13.30</td><td>140.00</td><td>Show result profile</td></tr>
<tr><td rowspan="8">46</td><td rowspan="8">hsa-miR-1302</td><td>972~994</td><td>23</td><td>miRNA: 3' aaauCGUAUUCA--UACAGGGUu 5'
|:|||| | :|||||||
Target:5' atgtGTATAAATTGGTGTCCCAt 3'</td><td>-14.10</td><td>159.00</td><td rowspan="8">Show result profile</td></tr>
<tr><td>972~994</td><td>23</td><td>miRNA: 3' aaauCGUAUUCA--UACAGGGUu 5'
|:|||| | :|||||||
Target:5' atgtGTATAAATTGGTGTCCCAt 3'</td><td>-14.10</td><td>159.00</td></tr>
<tr><td>972~994</td><td>23</td><td>miRNA: 3' aaauCGUAUUCA--UACAGGGUu 5'
|:|||| | :|||||||
Target:5' atgtGTATAAATTGGTGTCCCAt 3'</td><td>-14.10</td><td>159.00</td></tr>
<tr><td>972~994</td><td>23</td><td>miRNA: 3' aaauCGUAUUCA--UACAGGGUu 5'
|:|||| | :|||||||
Target:5' atgtGTATAAATTGGTGTCCCAt 3'</td><td>-14.10</td><td>159.00</td></tr>
<tr><td>972~994</td><td>23</td><td>miRNA: 3' aaauCGUAUUCA--UACAGGGUu 5'
|:|||| | :|||||||
Target:5' atgtGTATAAATTGGTGTCCCAt 3'</td><td>-14.10</td><td>159.00</td></tr>
<tr><td>972~994</td><td>23</td><td>miRNA: 3' aaauCGUAUUCA--UACAGGGUu 5'
|:|||| | :|||||||
Target:5' atgtGTATAAATTGGTGTCCCAt 3'</td><td>-14.10</td><td>159.00</td></tr>
<tr><td>972~994</td><td>23</td><td>miRNA: 3' aaauCGUAUUCA--UACAGGGUu 5'
|:|||| | :|||||||
Target:5' atgtGTATAAATTGGTGTCCCAt 3'</td><td>-14.10</td><td>159.00</td></tr>
<tr><td>972~994</td><td>23</td><td>miRNA: 3' aaauCGUAUUCA--UACAGGGUu 5'</td><td>-14.10</td><td>159.00</td></tr>
</table>

<table>
<tr><td rowspan="4"></td><td rowspan="4"></td><td></td><td></td><td>|:|||| | :|||||||
Target:5' atgtGTATAAATTGGTGTCCCAt
3'</td><td></td><td></td><td rowspan="4"></td></tr>
<tr><td>972~994</td><td>23</td><td>miRNA: 3' aaauCGUAUUCA--UACAGGGUu
5'
|:|||| | :|||||||
Target:5' atgtGTATAAATTGGTGTCCCAt
3'</td><td>-14.10</td><td>159.00</td></tr>
<tr><td>972~994</td><td>23</td><td>miRNA: 3' aaauCGUAUUCA--UACAGGGUu
5'
|:|||| | :|||||||
Target:5' atgtGTATAAATTGGTGTCCCAt
3'</td><td>-14.10</td><td>159.00</td></tr>
<tr><td>972~994</td><td>23</td><td>miRNA: 3' aaauCGUAUUCA--UACAGGGUu
5'
|:|||| | :|||||||
Target:5' atgtGTATAAATTGGTGTCCCAt
3'</td><td>-14.10</td><td>159.00</td></tr>
<tr><td rowspan="3">47</td><td rowspan="3">hsa-miR-1304</td><td>1105~1129</td><td>25</td><td>miRNA: 3' guGUAGA--GUG-
ACAUCGGAGUUu 5'
|||:| || |||||:||||
Target:5'
aaCATTTAAAACAAGTAGCTTCAAg 3'</td><td>-17.50</td><td>146.00</td><td rowspan="3">Show result profile</td></tr>
<tr><td>2098~2121</td><td>24</td><td>miRNA: 3' guGUAGAGUGAC--A-
UCGGAGUuu 5'
| |||| ||| | |||||||
Target:5' ggCTTCTC-
CTGCCTCAGCCTCAcg 3'</td><td>-15.80</td><td>141.00</td></tr>
<tr><td>195~216</td><td>22</td><td>miRNA: 3' guGUAGAGUGACAUCGGAGUUu
5'
| |:|||| | ||:||:||
Target:5' caCCTTTCACAGCAGTCTTAAa
3'</td><td>-16.50</td><td>140.00</td></tr>
<tr><td>48</td><td>hsa-miR-130a*</td><td>1943~1963</td><td>21</td><td>miRNA: 3' cgUCUGUCAUCGUG-UUACACUu
5'
|||:| | ||: |||||||
Target:5' ccAGATATT--CATAAATGTGAc
3'</td><td>-11.80</td><td>156.00</td><td>Show result profile</td></tr>
<tr><td>49</td><td>hsa-miR-130b*</td><td>1363~1384</td><td>22</td><td>miRNA: 3' caucaCGUUGUC-CCUUUCUCa
5'
||||||| ||||| ||
Target:5' ggaaaGCAACAGAGGAAACAGt
3'</td><td>-17.60</td><td>140.00</td><td>Show result profile</td></tr>
<tr><td>50</td><td>hsa-miR-1323</td><td>2407~2428</td><td>22</td><td>miRNA: 3' ucuuuuACGGGGAGUCAAAACu
5'
| ::|:|::||||||
Target:5' tattttTATTCTTTGGTTTTGa
3'</td><td>-11.90</td><td>140.00</td><td>Show result profile</td></tr>
<tr><td>51</td><td>hsa-miR-134</td><td>1275~1297</td><td>23</td><td>miRNA: 3' ggggagACCAGU-UGGUCAGUGu
5'
|| | | |:|||||||
Target:5' aggaagTGCTAAGATCAGTCACc
3'</td><td>-13.70</td><td>152.00</td><td>Show result profile</td></tr>
</table>

<table>
<tr><td rowspan="2">52</td><td rowspan="2">hsa-miR-135a</td><td>1295~1317</td><td>23</td><td>miRNA: 3' aguGUAUCCUUAUUUUUCGGUau 5'
|||: ||||||:||||||
Target:5' accCATGTGAATAAGAAGCCAgg 3'</td><td>-18.80</td><td>164.00</td><td rowspan="2">Show result profile</td></tr>
<tr><td>1295~1317</td><td>23</td><td>miRNA: 3' aguGUAUCCUUAUUUUUCGGUau 5'
|||: ||||||:||||||
Target:5' accCATGTGAATAAGAAGCCAgg 3'</td><td>-18.80</td><td>164.00</td></tr>
<tr><td>53</td><td>hsa-miR-135b</td><td>1295~1317</td><td>23</td><td>miRNA: 3' aguGUAUCCUUACUUUUCGGUau 5'
|||: |||| |:||||||
Target:5' accCATGTGAATAAGAAGCCAgg 3'</td><td>-15.10</td><td>156.00</td><td>Show result profile</td></tr>
<tr><td rowspan="2">54</td><td rowspan="2">hsa-miR-138</td><td>979~1001</td><td>23</td><td>miRNA: 3' gccgGACUAAGUGUUGUGGUCGa 5'
:||:| : | |:||||||
Target:5' taaaTTGGTGTCCCATACCAGCt 3'</td><td>-16.30</td><td>143.00</td><td rowspan="2">Show result profile</td></tr>
<tr><td>979~1001</td><td>23</td><td>miRNA: 3' gccgGACUAAGUGUUGUGGUCGa 5'
:||:| : | |:||||||
Target:5' taaaTTGGTGTCCCATACCAGCt 3'</td><td>-16.30</td><td>143.00</td></tr>
<tr><td>55</td><td>hsa-miR-140-5p</td><td>1113~1138</td><td>26</td><td>miRNA: 3' gaUGGU-AUCCCA---UUUUGGUGAc 5'
|| | ||| | :||||||||
Target:5' aaACAAGTAGCTTCAAGAAACCACTg 3'</td><td>-13.90</td><td>152.00</td><td>Show result profile</td></tr>
<tr><td rowspan="2">56</td><td rowspan="2">hsa-miR-141*</td><td>1591~1613</td><td>23</td><td>miRNA: 3' agGUUGUG-ACAUGACCUUCUAc 5'
::|::: |||| |||||||
Target:5' aaTGATGTGTGTAAGGGAAGATt 3'</td><td>-12.80</td><td>156.00</td><td rowspan="2">Show result profile</td></tr>
<tr><td>640~665</td><td>26</td><td>miRNA: 3' agGUUG--UGACA--UGACCUUCUAc 5'
:|:| ||||| ||||||||
|
Target:5' atTAGCCAACTGTTAACTGGAAGCTt 3'</td><td>-18.62</td><td>140.00</td></tr>
<tr><td>57</td><td>hsa-miR-141</td><td>167~185</td><td>19</td><td>miRNA: 3' gguagaaAUGGUCUGUCACAAu 5'
|||: |||||||
Target:5' tacaaagTACT---CAGTGTTc 3'</td><td>-7.70</td><td>144.00</td><td>Show result profile</td></tr>
<tr><td>58</td><td>hsa-miR-143*</td><td>1173~1193</td><td>21</td><td>miRNA: 3' uggUCUCUACGUCGUGACGUGg 5'
| ||| |:| :|:|||||
Target:5' ggaACAGA-GTATTATTGCACa 3'</td><td>-15.50</td><td>142.00</td><td>Show result profile</td></tr>
</table>

<table>
<tr><td>59</td><td>hsa-miR-144</td><td>2576~2596</td><td>21</td><td>miRNA: 3' ucAUGUAGUA-GAUAUGACAu 5'
||||| | :||||:|||
Target:5' aaTACATGTTGTTATATTGTa 3'</td><td>-11.50</td><td>146.00</td><td>Show result profile</td></tr>
<tr><td rowspan="2">60</td><td rowspan="2">hsa-miR-145</td><td>1735~1767</td><td>33</td><td>miRNA: 3' ucCCUAA---GGACC-------CUUUUGACCUg 5'
||||| :||||
|:|:|||||
Target:5' agGGATTGTGTCTGGCTCTTTTTAGAGCTGGAa 3'</td><td>-22.20</td><td>141.00</td><td rowspan="2">Show result profile</td></tr>
<tr><td>639~661</td><td>23</td><td>miRNA: 3' ucccuaaggacccuuUUGACCUg 5'
|||||||
Target:5' aattagccaactgttAACTGGAa 3'</td><td>-11.30</td><td>140.00</td></tr>
<tr><td>61</td><td>hsa-miR-1468</td><td>1409~1429</td><td>21</td><td>miRNA: 3' guCGCUUUGUCCGUUUGCCUc 5'
|:| |||| ||:||||
Target:5' aaGTGTTACAGAAAAGCGGAc 3'</td><td>-18.80</td><td>143.00</td><td>Show result profile</td></tr>
<tr><td>62</td><td>hsa-miR-146a*</td><td>2209~2230</td><td>22</td><td>miRNA: 3' gacUUCUUGACUUAAAGUCUCc 5'
|| | |||| |||||||
Target:5' aacAAAATCTGATGTTCAGAGg 3'</td><td>-16.30</td><td>163.00</td><td>Show result profile</td></tr>
<tr><td>63</td><td>hsa-miR-148b*</td><td>314~337</td><td>24</td><td>miRNA: 3' cggacucacaUAUU--GUCUUGAa 5'
||:: |||||||
Target:5' tttctaaaacATGGTTCAGAACTg 3'</td><td>-7.30</td><td>142.00</td><td>Show result profile</td></tr>
<tr><td>64</td><td>hsa-miR-150*</td><td>1900~1921</td><td>22</td><td>miRNA: 3' gacagggggucc ggACAUGGUc 5'
|||||||
Target:5' atcagcatttgaagTGTACCAt 3'</td><td>-8.40</td><td>140.00</td><td>Show result profile</td></tr>
<tr><td>65</td><td>hsa-miR-151-3p</td><td>2518~2541</td><td>24</td><td>miRNA: 3' ggAGUUCCU--CG-AAGUCAGAUc 5'
| ||| | |: ||||||:||
Target:5' ttTGAAGTATTGTATTCAGTTTAc 3'</td><td>-8.00</td><td>141.00</td><td>Show result profile</td></tr>
<tr><td rowspan="2">66</td><td rowspan="2">hsa-miR-153</td><td>1629~1650</td><td>22</td><td>miRNA: 3' cuagugaaaaCACUGAUACGUu 5'
|| |||||||
Target:5' gaagaaatcaGTATCTATGCAa 3'</td><td>-10.10</td><td>144.00</td><td rowspan="2">Show result profile</td></tr>
<tr><td>1629~1650</td><td>22</td><td>miRNA: 3' cuagugaaaaCACUGAUACGUu 5'
|| |||||||
Target:5' gaagaaatcaGTATCTATGCAa 3'</td><td>-10.10</td><td>144.00</td></tr>
<tr><td></td><td>hsa-</td><td></td><td></td><td>miRNA: 3' aucUCGUCGUUUAUUACUAAGc</td><td></td><td></td><td></td></tr>
</table>

<table>
<tr><td>67</td><td>miR-15b*</td><td>1668~1687</td><td>20</td><td>5'
||| | | |||||||||
Target:5' caaAGC--CCATTAATGATTCa 3'</td><td>-9.40</td><td>159.00</td><td>Show result profile</td></tr>
<tr><td>68</td><td>hsa-miR-16-2*</td><td>400~419</td><td>20</td><td>miRNA: 3' auuucGUCGUGUCAUUAUAACc 5'
||| |:| |||||||
Target:5' cctttCAGAATA--AATATTGg 3'</td><td>-10.30</td><td>153.00</td><td>Show result profile</td></tr>
<tr><td>69</td><td>hsa-miR-17*</td><td>1428~1449</td><td>22</td><td>miRNA: 3' gauguucacggaagUGACGUCa 5'
|||||||
Target:5' actggatagacaaaACTGCAGa 3'</td><td>-11.70</td><td>140.00</td><td>Show result profile</td></tr>
<tr><td>70</td><td>hsa-miR-182</td><td>1526~1553</td><td>28</td><td>miRNA: 3' ucacacUCA-AGAUG---GUAACGGUUu 5'
||| ||||| | |||:|||
Target:5' aaacaaAGTATCTACTGGCCTTGTCAAc 3'</td><td>-9.90</td><td>146.00</td><td>Show result profile</td></tr>
<tr><td>71</td><td>hsa-miR-1827</td><td>2096~2113</td><td>18</td><td>miRNA: 3' uaaguuAGAUGACGGAGu 5'
||| |||||||
Target:5' caggctTCTCCTGCCTCa 3'</td><td>-15.10</td><td>152.00</td><td>Show result profile</td></tr>
<tr><td>72</td><td>hsa-miR-183</td><td>2365~2391</td><td>27</td><td>miRNA: 3' ucACUUAA----GAU-GGUCACGGUAu 5'
| |||| :|: || |||||||
Target:5' aaTAAATTTATGTTGTCCTGTGCCATa 3'</td><td>-14.60</td><td>154.00</td><td>Show result profile</td></tr>
<tr><td>73</td><td>hsa-miR-183*</td><td>1667~1688</td><td>22</td><td>miRNA: 3' aauacCGGGAAGCCAUUAAGUg 5'
|||| |: |:|||||
Target:5' tcaaaGCCCATTAATGATTCAg 3'</td><td>-10.20</td><td>141.00</td><td>Show result profile</td></tr>
<tr><td>74</td><td>hsa-miR-186</td><td>2400~2421</td><td>22</td><td>miRNA: 3' ucggguuuuccucuUAAGAAAc 5'
|||||||
Target:5' tatttaatatttttATTCTTTg 3'</td><td>-7.90</td><td>140.00</td><td>Show result profile</td></tr>
<tr><td>75</td><td>hsa-miR-18a</td><td>1043~1066</td><td>24</td><td>miRNA: 3' gaUAGACGUGAUCUACGU-GGAAu 5'
||:| || |||| ||| ||||
Target:5' gaATTTACAGTAGAAGCATCCTTt 3'</td><td>-14.00</td><td>145.00</td><td>Show result profile</td></tr>
<tr><td>76</td><td>hsa-miR-18b*</td><td>1444~1466</td><td>23</td><td>miRNA: 3' cgGUCUUCCCCGUA-AAUCCCgu 5'
||||||| |:|| ||:|||
Target:5' tgCAGAAGGTGTATGTTGGGGag 3'</td><td>-21.70</td><td>144.00</td><td>Show result profile</td></tr>
<tr><td>77</td><td>hsa-miR-190</td><td>836~856</td><td>21</td><td>miRNA: 3' uggaUUAUAUAGUUUGUAUAGu 5'
|:|: || |:|||||||
Target:5' atgaAGTGAAT-AGACATATCa</td><td>-11.00</td><td>157.00</td><td>Show result profile</td></tr>
</table>

<table>
<tr><td></td><td></td><td></td><td></td><td>3'</td><td></td><td></td><td></td></tr>
<tr><td>78</td><td>hsa-miR-190b</td><td>835~856</td><td>22</td><td>miRNA: 3' uugggUUA-UAGUUUGUAUAGu 5'
|:| | :|:|||||||
Target:5' gatgaAGTGAATAGACATATCa 3'</td><td>-10.90</td><td>152.00</td><td>Show result profile</td></tr>
<tr><td rowspan="2">79</td><td rowspan="2">hsa-miR-1913</td><td>20~42</td><td>23</td><td>miRNA: 3' accgucgUCGCCUC-CCCCGUCu 5'
||:|||| |||||:|
Target:5' ggacgtgAGTGGAGCGGGGCGGt 3'</td><td>-28.00</td><td>147.00</td><td rowspan="2">Show result profile</td></tr>
<tr><td>1386~1407</td><td>22</td><td>miRNA: 3' acCGUCGUCGCCUCCCCCGUCu 5'
| :| | | ||||||:||
Target:5' aaGGGGAACAGAAGGGGGTAGc 3'</td><td>-23.10</td><td>140.00</td></tr>
<tr><td>80</td><td>hsa-miR-192</td><td>814~834</td><td>21</td><td>miRNA: 3' ccgacAGUUAAGUAUCCAGUc 5'
| ||| :||||||:|
Target:5' ttaatTAAATATATAGGTTAt 3'</td><td>-8.20</td><td>144.00</td><td>Show result profile</td></tr>
<tr><td rowspan="2">81</td><td rowspan="2">hsa-miR-195*</td><td>401~419</td><td>19</td><td>miRNA: 3' ccucGUCGUGUCGGUUAUAACc 5'
||| |:| |||||||
Target:5' ctttCAGAATA---AATATTGg 3'</td><td>-11.00</td><td>151.00</td><td rowspan="2">Show result profile</td></tr>
<tr><td>2391~2412</td><td>22</td><td>miRNA: 3' ccUCGUCGUGUCGGUUAUAAcc 5'
||:: :|:| ::||||||
Target:5' aaAGTGATATATTTAATATTtt 3'</td><td>-7.50</td><td>140.00</td></tr>
<tr><td>82</td><td>hsa-miR-196a*</td><td>503~524</td><td>22</td><td>miRNA: 3' gaguccgucaAAGAACAACGGc 5'
||:||||||:|
Target:5' catctgtcccTTTTTGTTGTCc 3'</td><td>-9.90</td><td>140.00</td><td>Show result profile</td></tr>
<tr><td rowspan="4">83</td><td rowspan="4">hsa-miR-199a-3p</td><td>282~306</td><td>25</td><td>miRNA: 3' auUGGUUA-CAC--GUCUGAUGACa 5'
|: ||| || ||||||:|||
Target:5' gaATAAATCCTGCACAGACTGCTGg 3'</td><td>-15.60</td><td>146.00</td><td rowspan="4">Show result profile</td></tr>
<tr><td>555~573</td><td>19</td><td>miRNA: 3' auUGGUUACACGUCUGAUGACa 5'
|::|| | || |||||||
Target:5' gaATTAA-G-GC-TACTACTGt 3'</td><td>-12.70</td><td>145.00</td></tr>
<tr><td>282~306</td><td>25</td><td>miRNA: 3' auUGGUUA-CAC--GUCUGAUGACa 5'
|: ||| || ||||||:|||
Target:5' gaATAAATCCTGCACAGACTGCTGg 3'</td><td>-15.60</td><td>146.00</td></tr>
<tr><td></td><td></td><td></td><td></td><td></td></tr>
</table>

<table>
<tr><td></td><td></td><td>555~573</td><td>19</td><td>miRNA: 3' auUGGUUACACGUCUGAUGACa 5'
|::|| | || |||||||
Target:5' gaATTAA-G-GC-TACTACTGt 3'</td><td>-12.70</td><td>145.00</td><td></td></tr>
<tr><td rowspan="2">84</td><td rowspan="2">hsa-miR-199b-3p</td><td>282~306</td><td>25</td><td>miRNA: 3' auUGGUUA-CAC--GUCUGAUGACa 5'
|: ||| || ||||||:|||
Target:5' gaATAAATCCTGCACAGACTGCTGg 3'</td><td>-15.60</td><td>146.00</td><td rowspan="2">Show result profile</td></tr>
<tr><td>555~573</td><td>19</td><td>miRNA: 3' auUGGUUACACGUCUGAUGACa 5'
|::|| | || |||||||
Target:5' gaATTAA-G-GC-TACTACTGt 3'</td><td>-12.70</td><td>145.00</td></tr>
<tr><td>85</td><td>hsa-miR-19a</td><td>775~798</td><td>24</td><td>miRNA: 3' agucaaAACGUA-UCUAAACGUgu 5'
| |||| ||||||||
Target:5' gcttgaTAGCATCTGATTTGCAga 3'</td><td>-12.20</td><td>141.00</td><td>Show result profile</td></tr>
<tr><td>86</td><td>hsa-miR-19b-1*</td><td>1428~1452</td><td>25</td><td>miRNA: 3' cgACCUA-C-GUUUGGACGUUUUga 5'
||||| | |||| |||||:||
Target:5' acTGGATAGACAAAACTGCAGAAgg 3'</td><td>-19.30</td><td>145.00</td><td>Show result profile</td></tr>
<tr><td rowspan="2">87</td><td rowspan="2">hsa-miR-19b</td><td>775~798</td><td>24</td><td>miRNA: 3' agucaaAACGUA-CCUAAACGUgu 5'
| |||| ||||||||
Target:5' gcttgaTAGCATCTGATTTGCAga 3'</td><td>-12.00</td><td>141.00</td><td rowspan="2">Show result profile</td></tr>
<tr><td>775~798</td><td>24</td><td>miRNA: 3' agucaaAACGUA-CCUAAACGUgu 5'
| |||| ||||||||
Target:5' gcttgaTAGCATCTGATTTGCAga 3'</td><td>-12.00</td><td>141.00</td></tr>
<tr><td>88</td><td>hsa-miR-200a</td><td>167~185</td><td>19</td><td>miRNA: 3' uguagcaAUGGUCUGUCACAAu 5'
|||: |||||||
Target:5' tacaaagTACT---CAGTGTTc 3'</td><td>-8.80</td><td>144.00</td><td>Show result profile</td></tr>
<tr><td>89</td><td>hsa-miR-203</td><td>2435~2456</td><td>22</td><td>miRNA: 3' gaUCACCAGGA--UUUGUAAAGUg 5'
||| |||| ||||||||:|
Target:5' taAGT--TCCTAAAAACATTTTAt 3'</td><td>-12.10</td><td>154.00</td><td>Show result profile</td></tr>
<tr><td rowspan="2">90</td><td rowspan="2">hsa-miR-204</td><td>1452~1479</td><td>28</td><td>miRNA: 3' ucCGUA----UCCUACUG--UUUCCCUu 5'
|:|| :||| ||
|||||||
Target:5' gtGTATGTTGGGGAGAACTGAAAGGGAa 3'</td><td>-14.10</td><td>152.00</td><td rowspan="2">Show result profile</td></tr>
<tr><td></td><td></td><td>miRNA: 3' uccguaucCUACUGUUUCCCUu</td><td></td><td></td></tr>
</table>

		1~15	15	5'<br>||| :||:||||| Target:5' -------aGATTGCAGAGGGAg 3'	-15.40	142.00	
91	hsa-miR-2053	2543~2567	25	miRNA: 3' cauuuAUCUCCAA--AUUAAUUGUg 5'<br>|| |||| |||||||:| Target:5' tgcgtTATTGGTTTATAATTAATAt 3'	-7.20	148.00	Show result profile
92	hsa-miR-206	1056~1084	29	miRNA: 3' ggUGUGUGAAG-GA------AUGUAAGGu 5'<br>:||: |||: || |||||||| Target:5' aaGCATCCTTTGCTGAGTTATACATTCCt 3'	-11.30	154.00	Show result profile
93	hsa-miR-20a	1011~1034	24	miRNA: 3' gaUGGACGUGAUA-UUCGUGAAAu 5'<br>|||| :| || ||:||||| Target:5' ggACCTATAGAATCCAGTACTTTt 3'	-10.00	145.00	Show result profile
94	hsa-miR-20a*	1753~1774	22	miRNA: 3' gaAAUUCACGAGUAUUACGUCa 5'<br>|| || ||| : ||||:|| Target:5' ttTTTAGAGCTGGAAATGTAGt 3'	-12.90	148.00	Show result profile
95	hsa-miR-20b	1011~1034	24	miRNA: 3' gaUGGACGUGAUA-CUCGUGAAAc 5'<br>|||| :| || ||:||||| Target:5' ggACCTATAGAATCCAGTACTTTt 3'	-9.10	145.00	Show result profile
96	hsa-miR-20b*	2117~2139	23	miRNA: 3' gaccUUCA-CGGGUAUGAUGUCa 5'<br>:||| ||: : ||||||| Target:5' tcacGAGTAGCTGGGACTACAGg 3'	-13.50	154.00	Show result profile
97	hsa-miR-21*	1119~1144	26	miRNA: 3' ugUCGGG-----UAGCUGACCACAAc 5'<br>|||:: | | ||||||||| Target:5' gtAGCTTCAAGAAACCACTGGTGTTt 3'	-13.90	155.00	Show result profile
98	hsa-miR-211	1456~1479	24	miRNA: 3' ucCGCUUCCUACUG--UUUCCCUu 5'<br>|: ::||| || ||||||| Target:5' atGTTGGGGAGAACTGAAAGGGAa 3'	-13.90	154.00	Show result profile
		1~15	15	miRNA: 3' uccgcuucCUACUGUUUCCCUu 5'<br>||| :||:||||| Target:5' -------aGATTGCAGAGGGAg 3'	-15.40	142.00	
		2323~2343	21	miRNA: 3' cacuGUCUCGGUUCGUGUUUa 5'<br>| | :| ||||||||	-8.60	149.00	

<table>
<tr><td rowspan="2">99</td><td rowspan="2">hsa-miR-2113</td><td></td><td></td><td>Target:5' catcCTGTTTCTAGCACAAAa 3'</td><td></td><td></td><td rowspan="2">Show result profile</td></tr>
<tr><td>1173~1196</td><td>24</td><td>miRNA: 3' cacUGUCUC--GGU-UCGUGUUUa
5'
|||||| ::| |||||:|
Target:5' ggaACAGAGTATTATTGCACAGAt
3'</td><td>-16.60</td><td>140.00</td></tr>
<tr><td rowspan="2">100</td><td rowspan="2">hsa-miR-2115</td><td>637~664</td><td>28</td><td>miRNA: 3' aggUAGUC--CU--CAGU--
ACCUUCGa 5'
||:|| | ||:|
|||||||
Target:5'
tgaATTAGCCAACTGTTAACTGGAAGCt 3'</td><td>-14.70</td><td>149.00</td><td rowspan="2">Show result profile</td></tr>
<tr><td>1714~1735</td><td>22</td><td>miRNA: 3' aggUAGUCCUCAGUACCUUCGa
5'
|| : || : |||||||
Target:5' tgaATGGTGAACTCTGGAAGCa
3'</td><td>-9.40</td><td>147.00</td></tr>
<tr><td rowspan="2">101</td><td rowspan="2">hsa-miR-2115*</td><td>1698~1718</td><td>21</td><td>miRNA: 3' gaucGGAGGUACUUAAGACUac
5'
||||| || |||||||
Target:5' ttgaCCTCC-TGTATTCTGAat
3'</td><td>-17.70</td><td>149.00</td><td rowspan="2">Show result profile</td></tr>
<tr><td>1078~1103</td><td>26</td><td>miRNA: 3' gaucGGAGGUACUU----
AAGACUAc 5'
|||::|| ||
||:||||
Target:5'
cattCCTTTATCAATCTCTTTTGATa 3'</td><td>-9.40</td><td>144.00</td></tr>
<tr><td rowspan="2">102</td><td rowspan="2">hsa-miR-214</td><td>2335~2356</td><td>22</td><td>miRNA: 3' ugacggacagACACGGACGACa
5'
| |||||||||
Target:5' agcacaaaaaTTTGCCTGCTGt
3'</td><td>-16.29</td><td>152.00</td><td rowspan="2">Show result profile</td></tr>
<tr><td>228~249</td><td>22</td><td>miRNA: 3' ugacggacagaCACGGACGACa
5'
|| |||||||
Target:5' aagaatttaaaGTTCCTGCTGc
3'</td><td>-12.60</td><td>147.00</td></tr>
<tr><td>103</td><td>hsa-miR-215</td><td>814~834</td><td>21</td><td>miRNA: 3' cagacAGUUAAGUAUCCAGUa 5'
| ||| :||||||:|
Target:5' ttaatTAAATATATAGGTTAt 3'</td><td>-10.20</td><td>144.00</td><td>Show result profile</td></tr>
<tr><td>104</td><td>hsa-miR-22*</td><td>213~234</td><td>22</td><td>miRNA: 3' auUUCGAACGGUGACUUCUUGa
5'
|||:|||| :| ||||||:
Target:5' taAAGTTTGCAGCAGAAGAATt
3'</td><td>-17.50</td><td>160.00</td><td>Show result profile</td></tr>
<tr><td>105</td><td>hsa-miR-221</td><td>1753~1775</td><td>23</td><td>miRNA: 3' cuuugggUCGUCUGUUACAUCGa
5'
||| |: |||||||:
Target:5' tttttagAGCTGGAAATGTAGTg
3'</td><td>-11.70</td><td>144.00</td><td>Show result profile</td></tr>
</table>

<table>
<tr><td>106</td><td>hsa-miR-222</td><td>1754~1775</td><td>22</td><td>miRNA: 3' ugGGUCAUCGGUC--UACAUCGa 5'
::|| |||::| ||||||:
Target:5' ttTTAG-AGCTGGAAATGTAGTg 3'</td><td>-13.20</td><td>140.00</td><td>Show result profile</td></tr>
<tr><td>107</td><td>hsa-miR-223*</td><td>174~198</td><td>25</td><td>miRNA: 3' uuGAGUCGAACAG----UUUAUGUGc 5'
|||||: | || ||:|||||
Target:5' taCTCAGTGT-TCCTGAAAGTACACc 3'</td><td>-14.42</td><td>145.00</td><td>Show result profile</td></tr>
<tr><td>108</td><td>hsa-miR-224</td><td>1761~1780</td><td>20</td><td>miRNA: 3' uuGCCUUGGUGAUCACUGAAc 5'
:|||| :: |||||:|||
Target:5' gcTGGAAATG-TAGTGGCTTt 3'</td><td>-14.00</td><td>146.00</td><td>Show result profile</td></tr>
<tr><td>109</td><td>hsa-miR-2276</td><td>779~798</td><td>20</td><td>miRNA: 3' ggAGCGGAGACUGUGAACGUCu 5'
| || |||| |:||||||
Target:5' gaTAGCATCTG--ATTTGCAGa 3'</td><td>-15.90</td><td>148.00</td><td>Show result profile</td></tr>
<tr><td>110</td><td>hsa-miR-2355-5p</td><td>1445~1466</td><td>22</td><td>miRNA: 3' aacagguaACAUA-GACCCCUa 5'
||||| :||||||
Target:5' gcagaaggTGTATGTTGGGGAg 3'</td><td>-13.90</td><td>141.00</td><td>Show result profile</td></tr>
<tr><td rowspan="2">111</td><td rowspan="2">hsa-miR-23a</td><td>1945~1963</td><td>19</td><td>miRNA: 3' ccUUUAGGGACCGUUACACUa 5'
| ||:| | |||||||
Target:5' agATATTCAT--AAATGTGAc 3'</td><td>-8.90</td><td>147.00</td><td rowspan="2">Show result profile</td></tr>
<tr><td>2473~2493</td><td>21</td><td>miRNA: 3' ccuuuagggaccgUUACACUa 5'
|||||||
Target:5' ttgttattaaaaaAATGTGAt 3'</td><td>-7.60</td><td>140.00</td></tr>
<tr><td rowspan="2">112</td><td rowspan="2">hsa-miR-23b</td><td>1945~1963</td><td>19</td><td>miRNA: 3' ccauUAGGGACCGUUACACUa 5'
||:| | |||||||
Target:5' agatATTCAT--AAATGTGAc 3'</td><td>-7.20</td><td>145.00</td><td rowspan="2">Show result profile</td></tr>
<tr><td>2473~2493</td><td>21</td><td>miRNA: 3' ccauuagggaccgUUACACUa 5'
|||||||
Target:5' ttgttattaaaaaAATGTGAt 3'</td><td>-7.80</td><td>140.00</td></tr>
<tr><td>113</td><td>hsa-miR-23c</td><td>2473~2493</td><td>21</td><td>miRNA: 3' ccCAUUAGUGACCGUUACACUa 5'
|| ||:| |||||||
Target:5' ttGTTATTA-AAAAAATGTGAt 3'</td><td>-9.40</td><td>143.00</td><td>Show result profile</td></tr>
<tr><td>114</td><td>hsa-miR-25</td><td>241~262</td><td>22</td><td>miRNA: 3' agucUGGCUCUGUUCACGUUAc 5'
:|:| ||||||||||
Target:5' tcctGCTGCAACAAGTGCAATt 3'</td><td>-17.00</td><td>166.00</td><td>Show result profile</td></tr>
</table>

<table>
<tr><th></th><th></th><th></th><th></th><th></th><th></th><th></th><th></th></tr>
<tr><td>115</td><td>hsa-miR-26a</td><td>753~781</td><td>29</td><td>miRNA: 3' ucggaUAGGACCU-------AAUGAACUu 5'
| |||||| ||:|||||
Target:5' tatggACCCTGGATGGCAATTTGCTTGAt 3'</td><td>-14.81</td><td>141.00</td><td>Show result profile</td></tr>
<tr><td rowspan="2">116</td><td rowspan="2">hsa-miR-26a-1*</td><td>254~282</td><td>29</td><td>miRNA: 3' gcACGUUCAU--UGGU-----UCUUAUCc 5'
||||| || |||| :||||||
Target:5' agTGCAATTATTACCAATGATGGAATAGg 3'</td><td>-18.00</td><td>150.00</td><td rowspan="2">Show result profile</td></tr>
<tr><td>1122~1154</td><td>33</td><td>miRNA: 3' gcACGUUCAUUGGU-----------UCUUAUCc 5'
| |||| ||||| ||:||||
Target:5' gcTTCAAGAAACCACTGGTGTTTTGAGGATAGt 3'</td><td>-12.70</td><td>140.00</td></tr>
<tr><td>117</td><td>hsa-miR-26a</td><td>753~781</td><td>29</td><td>miRNA: 3' ucggaUAGGACCU-------AAUGAACUu 5'
| |||||| ||:|||||
Target:5' tatggACCCTGGATGGCAATTTGCTTGAt 3'</td><td>-14.81</td><td>141.00</td><td>Show result profile</td></tr>
<tr><td>118</td><td>hsa-miR-26a-2*</td><td>248~282</td><td>35</td><td>miRNA: 3' cuUUGUUCA--UUAGU-----------UCUUAUCc 5'
||||||| |||:| :||||||
Target:5' gcAACAAGTGCAATTATTACCAATGATGGAATAGg 3'</td><td>-15.30</td><td>142.00</td><td>Show result profile</td></tr>
<tr><td>119</td><td>hsa-miR-26b</td><td>1679~1702</td><td>24</td><td>miRNA: 3' uggaUAGGACUUA---AUGAACUu 5'
||:| |||| |:|||||
Target:5' aatgATTCAGAATCAGTGCTTGAc 3'</td><td>-8.00</td><td>145.00</td><td>Show result profile</td></tr>
<tr><td>120</td><td>hsa-miR-26b*</td><td>1376~1397</td><td>22</td><td>miRNA: 3' cucgguUCAUUACCUCUUGUCc 5'
|||:| ||:||||||
Target:5' ggaaacAGTGAAGGGGAACAGa 3'</td><td>-17.80</td><td>152.00</td><td>Show result profile</td></tr>
<tr><td>121</td><td>hsa-miR-27b*</td><td>1842~1867</td><td>26</td><td>miRNA: 3' caAGUGGU--UAGUC--GAUUCGAGa 5'
|| |:| || :| |||||:||
Target:5' ctTCTCTATCATAGGCCCTAAGTTCa 3'</td><td>-10.00</td><td>140.00</td><td>Show result profile</td></tr>
<tr><td rowspan="2">122</td><td rowspan="2">hsa-miR-28-5p</td><td>2423~2444</td><td>22</td><td>miRNA: 3' gaguuaUCUGACACUCGAGGAa 5'
| |:||| ||:||||
Target:5' ttttgaACATTGTAAGTTCCTa 3'</td><td>-11.80</td><td>144.00</td><td rowspan="2">Show result profile</td></tr>
<tr><td>223~245</td><td>23</td><td>miRNA: 3' gaGUUAUCUGACA-CUCGAGGAa 5'
||: ||| | | ||:||||
Target:5' agCAGAAGAATTTAAAGTTCCTg</td><td>-11.60</td><td>140.00</td></tr>
</table>

<table>
<tr><td></td><td></td><td></td><td></td><td>3'</td><td></td><td></td><td></td></tr>
<tr><td>123</td><td>hsa-miR-2909</td><td>1843~1863</td><td>21</td><td>miRNA: 3' gguucucuacAACCGGGAUUg 5'
                    | ||||||||
Target:5' ttctctatcaTAGGCCCTAAg 3'</td><td>-12.60</td><td>147.00</td><td>Show result profile</td></tr>
<tr><td>124</td><td>hsa-miR-296-3p</td><td>950~971</td><td>22</td><td>miRNA: 3' ccUCUCGGAGGUGGGUUGGGAg
5'
            | | ::|: |:|:||||||
Target:5' ttAAAATTTTAATCTAACCCTt
3'</td><td>-15.40</td><td>144.00</td><td>Show result profile</td></tr>
<tr><td rowspan="2">125</td><td rowspan="2">hsa-miR-297</td><td>1539~1559</td><td>21</td><td>miRNA: 3' guACGUGUACGUGUGUAUGUa 5'
            || | ||: |||||||
Target:5' acTGGCCTTGTCAACATACAg 3'</td><td>-9.20</td><td>151.00</td><td rowspan="2">Show result profile</td></tr>
<tr><td>376~396</td><td>21</td><td>miRNA: 3' guACG-UGUACGUGUGUAUGUa
5'
            ||| || || | ||||||:
Target:5' tcTGCTACTTGGA-ACATACGa
3'</td><td>-9.50</td><td>142.00</td></tr>
<tr><td>126</td><td>hsa-miR-299-5p</td><td>2581~2601</td><td>21</td><td>miRNA: 3' uaCAUACACCCUGCCAUUUGGu
5'
            || ||| |: |||||||
Target:5' atGT-TGTTATATTGTAAACCa
3'</td><td>-10.50</td><td>151.00</td><td>Show result profile</td></tr>
<tr><td rowspan="3">127</td><td rowspan="3">hsa-miR-29a*</td><td>1619~1640</td><td>22</td><td>miRNA: 3' gacuuguGGUUUUCUUUAGUCa
5'
                 ||::||||||||||
Target:5' tgcccttCCGGAAGAAATCAGt
3'</td><td>-19.70</td><td>167.00</td><td rowspan="3">Show result profile</td></tr>
<tr><td>427~450</td><td>24</td><td>miRNA: 3' gacuugugGUUUU--CUUUAGUCa
5'
                  :|||| ||||||||
Target:5' tgttgttgTAAAATTGAAATCAGg
3'</td><td>-9.60</td><td>156.00</td></tr>
<tr><td>1884~1905</td><td>22</td><td>miRNA: 3' gaCUUGUGGUUUUCUUUAGUCa
5'
            ||| |:: || :||||||
Target:5' aaGAAGATTCAACAGAATCAGc
3'</td><td>-10.40</td><td>144.00</td></tr>
<tr><td rowspan="3">128</td><td rowspan="3">hsa-miR-29b-1*</td><td>1287~1317</td><td>31</td><td>miRNA: 3' agAUUUGGUGG-------
UAUACUUUGGUCg 5'
            | |::|||| |||
|||:||||
Target:5'
gaTCAGTCACCCATGTGAATAAGAAGCCAGg 3'</td><td>-18.10</td><td>150.00</td><td rowspan="3">Show result profile</td></tr>
<tr><td>1617~1640</td><td>24</td><td>miRNA: 3' agauuuGGUGGUAUACUUUGGUCg
5'
                |: ||: | ||||:|||
Target:5' tttgccCTTCCGGAAGAAATCAGt
3'</td><td>-14.60</td><td>142.00</td></tr>
<tr><td></td><td></td><td></td><td></td><td></td></tr>
</table>

<table>
<tr><th></th><th></th><th></th><th></th><th></th><th></th><th></th><th></th></tr>
<tr><td></td><td></td><td>433~450</td><td>18</td><td>miRNA: 3' agAUUUGGUGGUAUACUUUGGUCg
5'
|||| || |||||:|||
Target:5' tgTAAA-----AT-TGAAATCAGg
3'</td><td>-8.90</td><td>140.00</td><td></td></tr>
<tr><td>129</td><td>hsa-miR-29b-2*</td><td>1288~1316</td><td>29</td><td>miRNA: 3' gauUCGGU-GGUACA------
CUUUGGUc 5'
||:|| ||||||
|||:|||
Target:5'
atcAGTCACCCATGTGAATAAGAAGCCAg 3'</td><td>-22.90</td><td>149.00</td><td>Show result profile</td></tr>
<tr><td></td><td></td><td>1115~1136</td><td>22</td><td>miRNA: 3' gaUUCGGUGGUACACUUUGGUc
5'
|||: :|: |||||||
Target:5' acAAGTAGCTTCAAGAAACCAc
3'</td><td>-9.40</td><td>148.00</td><td></td></tr>
<tr><td>130</td><td>hsa-miR-301a</td><td>1172~1194</td><td>23</td><td>miRNA: 3' cgaaacUGUUAUGAUAACGUGac
5'
| |:||:||||||||
Target:5' aggaacAGAGTATTATTGCACag
3'</td><td>-13.30</td><td>149.00</td><td>Show result profile</td></tr>
<tr><td>131</td><td>hsa-miR-301b</td><td>1172~1194</td><td>23</td><td>miRNA: 3' cgaaacUGUUAUAGUAACGUGac
5'
| |:|||:|||||||
Target:5' aggaacAGAGTATTATTGCACag
3'</td><td>-13.20</td><td>149.00</td><td>Show result profile</td></tr>
<tr><td>132</td><td>hsa-miR-302a*</td><td>2008~2035</td><td>28</td><td>miRNA: 3' ucgUUCA----UGUAGG-
UGCAAAUUCa 5'
|:|| | |||:
|:||||:||
Target:5'
ctgAGGTGAAAAAATCTGATGTTTGAGg 3'</td><td>-12.90</td><td>142.00</td><td>Show result profile</td></tr>
<tr><td>133</td><td>hsa-miR-302d</td><td>1888~1910</td><td>23</td><td>miRNA: 3' ugUGAGUUUGUACCU--
UCGUGAAu 5'
|:|| |||| | | ||||:||
Target:5' agATTC-AACA-
GAATCAGCATTTg 3'</td><td>-11.30</td><td>141.00</td><td>Show result profile</td></tr>
<tr><td>134</td><td>hsa-miR-3065-5p</td><td>409~432</td><td>24</td><td>miRNA: 3' aggUCGUAGUCACU--
AAAACAACu 5'
| :||::|| | ||||||||
Target:5' ataAATATTGGT-
ATTTTTTGTTGt 3'</td><td>-10.10</td><td>157.00</td><td>Show result profile</td></tr>
<tr><td></td><td></td><td>498~522</td><td>25</td><td>miRNA: 3' aggucGUAGUCA--
CUAAAACAACu 5'
|||| || ||||||||
Target:5'
tgactCATCTGTCCCTTTTTGTTGt 3'</td><td>-11.90</td><td>156.00</td><td></td></tr>
<tr><td>135</td><td>hsa-miR-30a*</td><td>1449~1475</td><td>27</td><td>miRNA: 3' cgaCGUUUGUAGGC-----
UGACUUUc 5'
|:: |::|: |
|||||||
Target:5'
aagGTGTATGTTGGGGAGAACTGAAAg 3'</td><td>-12.10</td><td>143.00</td><td>Show result profile</td></tr>
</table>

<table>
<tr><td rowspan="2">136</td><td rowspan="2">hsa-miR-30c</td><td>1907~1936</td><td>30</td><td>miRNA: 3' cgACUCUCACAU-----CC-U--ACAAAUGu 5'
||| |||||| || | ||||||:
Target:5' ttTGA-AGTGTACCATTGGTAGTTGTTTATg 3'</td><td>-10.90</td><td>142.00</td><td rowspan="2">Show result profile</td></tr>
<tr><td>1907~1936</td><td>30</td><td>miRNA: 3' cgACUCUCACAU-----CC-U--ACAAAUGu 5'
||| |||||| || | ||||||:
Target:5' ttTGA-AGTGTACCATTGGTAGTTGTTTATg 3'</td><td>-10.90</td><td>142.00</td></tr>
<tr><td rowspan="2">137</td><td rowspan="2">hsa-miR-30d*</td><td>641~662</td><td>22</td><td>miRNA: 3' cgUCGUUUGUAGACUGACUUUc 5'
||| ||| :| ||||:||
Target:5' ttAGCCAACTGTTAACTGGAAg 3'</td><td>-14.10</td><td>148.00</td><td rowspan="2">Show result profile</td></tr>
<tr><td>1449~1475</td><td>27</td><td>miRNA: 3' cgUCGUUUGUAGAC-----UGACUUUc 5'
:|:: |::|: | |||||||
Target:5' aaGGTGTATGTTGGGGAGAACTGAAAg 3'</td><td>-10.70</td><td>144.00</td></tr>
<tr><td>138</td><td>hsa-miR-30e*</td><td>1449~1475</td><td>27</td><td>miRNA: 3' cgaCAUUUGUAGGC-----UGACUUUc 5'
||: |::|: | |||||||
Target:5' aagGTGTATGTTGGGGAGAACTGAAAg 3'</td><td>-11.59</td><td>147.00</td><td>Show result profile</td></tr>
<tr><td rowspan="2">139</td><td rowspan="2">hsa-miR-3115</td><td>1002~1020</td><td>19</td><td>miRNA: 3' ugGUUGAUCAUUUGGGUAUa 5'
:|| |:||::|||:|||
Target:5' ttTAA-TGGTGGACCTATAg 3'</td><td>-13.00</td><td>145.00</td><td rowspan="2">Show result profile</td></tr>
<tr><td>786~805</td><td>20</td><td>miRNA: 3' ugGUUGAUCAUUUGGGUAUa 5'
::|:| | |:||:||||
Target:5' tcTGATTTGCAGACTCATAa 3'</td><td>-9.80</td><td>142.00</td></tr>
<tr><td>140</td><td>hsa-miR-3117</td><td>2239~2262</td><td>24</td><td>miRNA: 3' gaccGUGAUAU---ACUCAGGAUa 5'
|| || | ||||||:||
Target:5' cttaCAATAAATGTTGAGTCTTAg 3'</td><td>-9.80</td><td>141.00</td><td>Show result profile</td></tr>
<tr><td rowspan="3"></td><td rowspan="3"></td><td>1276~1297</td><td>22</td><td>miRNA: 3' ucUUAAAAGUAUUACGUCAGUGu 5'
|| | :| :|| |||||||
Target:5' ggAAGTGCTAAGAT-CAGTCACc 3'</td><td>-9.60</td><td>152.00</td><td rowspan="3"></td></tr>
<tr><td>1276~1297</td><td>22</td><td>miRNA: 3' ucUUAAAAGUAUUACGUCAGUGu 5'
|| | :| :|| |||||||
Target:5' ggAAGTGCTAAGAT-CAGTCACc 3'</td><td>-9.60</td><td>152.00</td></tr>
<tr><td>1276~1297</td><td>22</td><td>miRNA: 3' ucUUAAAAGUAUUACGUCAGUGu 5'
|| | :| :|| |||||||</td><td>-9.60</td><td>152.00</td></tr>
</table>

<table>
<tr><td rowspan="4">141</td><td rowspan="4">hsa-miR-3118</td><td></td><td></td><td>Target:5' ggAAGTGCTAAGAT-CAGTCACc 3'</td><td></td><td></td><td rowspan="4">Show result profile</td></tr>
<tr><td>1276~1297</td><td>22</td><td>miRNA: 3' ucUUAAAAGUAUUACGUCAGUGu 5'
|| | :| :|| |||||||
Target:5' ggAAGTGCTAAGAT-CAGTCACc 3'</td><td>-9.60</td><td>152.00</td></tr>
<tr><td>1276~1297</td><td>22</td><td>miRNA: 3' ucUUAAAAGUAUUACGUCAGUGu 5'
|| | :| :|| |||||||
Target:5' ggAAGTGCTAAGAT-CAGTCACc 3'</td><td>-9.60</td><td>152.00</td></tr>
<tr><td>1276~1297</td><td>22</td><td>miRNA: 3' ucUUAAAAGUAUUACGUCAGUGu 5'
|| | :| :|| |||||||
Target:5' ggAAGTGCTAAGAT-CAGTCACc 3'</td><td>-9.60</td><td>152.00</td></tr>
<tr><td rowspan="2">142</td><td rowspan="2">hsa-miR-3119</td><td>1200~1217</td><td>18</td><td>miRNA: 3' cggUAGUUUCAAUUUUCGGu 5'
|||||| ||||||:
Target:5' aagATCAAA--AAAAAGCTc 3'</td><td>-7.40</td><td>141.00</td><td rowspan="2">Show result profile</td></tr>
<tr><td>1200~1217</td><td>18</td><td>miRNA: 3' cggUAGUUUCAAUUUUCGGu 5'
|||||| ||||||:
Target:5' aagATCAAA--AAAAAGCTc 3'</td><td>-7.40</td><td>141.00</td></tr>
<tr><td rowspan="2">143</td><td rowspan="2">hsa-miR-3120</td><td>1774~1798</td><td>25</td><td>miRNA: 3' acgGACAG--A--UGUGAACGACAc 5'
|| || | |:|||||||||
Target:5' tggCTTTCATTAAATACTTGCTGTa 3'</td><td>-14.30</td><td>158.00</td><td rowspan="2">Show result profile</td></tr>
<tr><td>503~523</td><td>21</td><td>miRNA: 3' acGGACAGAUGUGAACGACAc 5'
:||||| : :|||:|||
Target:5' caTCTGTCCCTTTTTGTTGTc 3'</td><td>-14.30</td><td>143.00</td></tr>
<tr><td>144</td><td>hsa-miR-3125</td><td>115~134</td><td>20</td><td>miRNA: 3' agAGAGGUGUCGAAGGAGAu 5'
| || | :|:|||||:|
Target:5' caTGTCGAAGGTTTCCTTTa 3'</td><td>-13.20</td><td>142.00</td><td>Show result profile</td></tr>
<tr><td>145</td><td>hsa-miR-3126-5p</td><td>494~515</td><td>22</td><td>miRNA: 3' acgaagaccGUAGACAGGGAGu 5'
|||||||||||:
Target:5' aaggtgactCATCTGTCCCTTt 3'</td><td>-20.20</td><td>149.00</td><td>Show result profile</td></tr>
<tr><td rowspan="2">146</td><td rowspan="2">hsa-miR-3129</td><td>551~573</td><td>23</td><td>miRNA: 3' uuUGGUUAGAGAUG-UGAUGACg 5'
|: |||:   :| |||||||
Target:5' gtATGAATTAAGGCTACTACTGt 3'</td><td>-11.30</td><td>148.00</td><td rowspan="2">Show result profile</td></tr>
<tr><td>282~306</td><td>25</td><td>miRNA: 3' uuUGGUUAGAGAUG----UGAUGACg 5'
|: |||| ||:| |||:|||</td><td>-16.00</td><td>141.00</td></tr>
</table>

<table>
<tr><td></td><td></td><td></td><td></td><td>Target:5' gaATAAATC-
CTGCACAGACTGCTGg 3'</td><td></td><td></td><td></td></tr>
<tr><td>147</td><td>hsa-miR-3132</td><td>1830~1853</td><td>24</td><td>miRNA: 3' aggAGACUCGAGGAAGAGAUGGgu
5'
|:| ||: :||||||||:|
Target:5' atgTTTCAGTATCTTCTCTATCat
3'</td><td>-17.60</td><td>141.00</td><td>Show result profile</td></tr>
<tr><td>148</td><td>hsa-miR-3134</td><td>2305~2327</td><td>23</td><td>miRNA: 3' uuaUACAUCAGAAAAUAGGUAGu
5'
|| ||| :|||| ||||||
Target:5' ttaATTTAGATTTTTCTCCATCc
3'</td><td>-10.50</td><td>148.00</td><td>Show result profile</td></tr>
<tr><td>149</td><td>hsa-miR-3136</td><td>1273~1296</td><td>24</td><td>miRNA: 3' uuacugggAUGGAU-AAGUCAGUc
5'
|:|: | |||||||
Target:5' taaggaagTGCTAAGATCAGTCAc
3'</td><td>-8.40</td><td>143.00</td><td>Show result profile</td></tr>
<tr><td>150</td><td>hsa-miR-3138</td><td>507~527</td><td>21</td><td>miRNA: 3' ugAGGGAGAUGGAGUGACAGGUGu
5'
|||||:| |:::||||||:
Target:5' tgTCCCTTT---TTGTTGTCCATa
3'</td><td>-21.60</td><td>151.00</td><td>Show result profile</td></tr>
<tr><td rowspan="2">151</td><td rowspan="2">hsa-miR-3143</td><td>1391~1417</td><td>27</td><td>miRNA: 3' gcUUUCUUCGCGAAAUG--
UUACAAUa 5'
| ||||| | | :|
|:|||||
Target:5'
gaACAGAAGGGGGTAGCAAAGTGTTAc 3'</td><td>-12.90</td><td>145.00</td><td rowspan="2">Show result profile</td></tr>
<tr><td>2222~2255</td><td>34</td><td>miRNA: 3' gcuuUCUUC---GCGA--AAUG--
--UUACAAUa 5'
|||:| ||:| ||||
||||||:
Target:5'
gttcAGAGGCCCCGTTTCTTACAATAAATGTTGa
3'</td><td>-10.30</td><td>145.00</td></tr>
<tr><td>152</td><td>hsa-miR-3144-5p</td><td>26~47</td><td>22</td><td>miRNA: 3' gauauauagagaaaCCAGGGGa
5'
|||||||
Target:5' gagtggagcggggcGGTCCCCa
3'</td><td>-13.20</td><td>140.00</td><td>Show result profile</td></tr>
<tr><td rowspan="2">153</td><td rowspan="2">hsa-miR-3145</td><td>1937~1962</td><td>26</td><td>miRNA: 3' guUAA-GGUUUGUGAGU-
UUUAUAga 5'
||| |||:|:|:||| ||||:|
Target:5'
aaATTACCAGATATTCATAAATGTga 3'</td><td>-14.50</td><td>146.00</td><td rowspan="2">Show result profile</td></tr>
<tr><td>1515~1538</td><td>24</td><td>miRNA: 3' guUAAGGUUUGUGAGUUUUAUAGa
5'
| |: :|: | ||||:||||
Target:5' gcAGTTAGAGAAAACAAAGTATCt
3'</td><td>-13.40</td><td>142.00</td></tr>
<tr><td></td><td></td><td>1811~1832</td><td>22</td><td>miRNA: 3' gguaagAAAGAUAGGAUCGUAc
5'
|| :||||:||||||</td><td>-19.30</td><td>152.00</td><td></td></tr>
</table>

<table>
<tr><td rowspan="2">154</td><td rowspan="2">hsa-miR-3146</td><td></td><td></td><td>Target:5' gaccaaTTATTATCTTAGCATg
3'</td><td></td><td></td><td rowspan="2">Show result profile</td></tr>
<tr><td>2312~2340</td><td>29</td><td>miRNA: 3' ggUAAGAA-AGAUAG------
GAUCGUac 5'
|||:|| || |||
||||||
Target:5'
agATTTTTCTCCATCCTGTTTCTAGCAca 3'</td><td>-12.90</td><td>142.00</td></tr>
<tr><td>155</td><td>hsa-miR-3147</td><td>2137~2160</td><td>24</td><td>miRNA: 3' agUGUGGGAGG-
AGUGACGGGUUGg 5'
:||: | || ||| |||||:|
Target:5' agGCATGCACCACCAC-
GCCCAGCt 3'</td><td>-22.00</td><td>141.00</td><td>Show result profile</td></tr>
<tr><td>156</td><td>hsa-miR-3148</td><td>291~319</td><td>29</td><td>miRNA: 3' uuCGUGUGUGGUCA-------
AAAAAGGu 5'
||||| ||:: |
||||||:
Target:5'
ctGCACAGACTGCTGGAAATGTTTTTCTa 3'</td><td>-11.10</td><td>140.00</td><td>Show result profile</td></tr>
<tr><td>157</td><td>hsa-miR-3149</td><td>151~172</td><td>22</td><td>miRNA: 3' uaUGUGUGUGUAUAGGUAUGUUu
5'
|| |||: |: ||:|||||
Target:5' ggACCCACGGCTG-CCGTACAAa
3'</td><td>-16.50</td><td>144.00</td><td>Show result profile</td></tr>
<tr><td>158</td><td>hsa-miR-3152</td><td>2318~2340</td><td>23</td><td>miRNA: 3' aauaacgGGGAU-AAGAUUGUGu
5'
:|||: |||||:|||
Target:5' ttctccaTCCTGTTTCTAGCACa
3'</td><td>-17.50</td><td>143.00</td><td>Show result profile</td></tr>
<tr><td>159</td><td>hsa-miR-3154</td><td>495~517</td><td>23</td><td>miRNA: 3' agacgaGGGUUGA-GGGGAAGAc
5'
|:|| || :|||||:|
Target:5' aggtgaCTCATCTGTCCCTTTTt
3'</td><td>-14.60</td><td>140.00</td><td>Show result profile</td></tr>
<tr><td rowspan="2">160</td><td rowspan="2">hsa-miR-3158</td><td>1318~1340</td><td>23</td><td>miRNA: 3' caggacgUCU-CUCCUUCGGGaa
5'
||| |:||||||||
Target:5' aaaggaaAGATGGGGAAGCCCag
3'</td><td>-19.40</td><td>143.00</td><td rowspan="2">Show result profile</td></tr>
<tr><td>1318~1340</td><td>23</td><td>miRNA: 3' caggacgUCU-CUCCUUCGGGaa
5'
||| |:||||||||
Target:5' aaaggaaAGATGGGGAAGCCCag
3'</td><td>-19.40</td><td>143.00</td></tr>
<tr><td rowspan="2">161</td><td rowspan="2">hsa-miR-3180-</td><td>1711~1733</td><td>23</td><td>miRNA: 3'
gcugcaccCCGCCUCGCAGACCUUc 5'
||: || | |||||||
Target:5' ttctgaatGGT-GAAC-
TCTGGAAg 3'</td><td>-15.80</td><td>147.00</td><td rowspan="2">Show result profile</td></tr>
<tr><td>1711~1733</td><td>23</td><td>miRNA: 3'
gcugcaccCCGCCUCGCAGACCUUc 5'
||: || | |||||||
Target:5' ttctgaatGGT-GAAC-</td><td>-15.80</td><td>147.00</td></tr>
</table>

<table>
<tr><td></td><td>5p</td><td></td><td></td><td>TCTGGAAg 3'</td><td></td><td></td><td></td></tr>
<tr><td></td><td></td><td>1711~1733</td><td>23</td><td>miRNA: 3'
gcugcaccCCGCCUCGCAGACCUUc 5'
||: || | |||||||
Target:5' ttctgaatGGT-GAAC-
TCTGGAAg 3'</td><td>-15.80</td><td>147.00</td><td></td></tr>
<tr><td rowspan="3">162</td><td rowspan="3">hsa-miR-3182</td><td>562~582</td><td>21</td><td>miRNA: 3' cuGAUG-UGA---UGUCUUCg 5'
|||| ||| |||||||
Target:5' ggCTACTACTGTCACAGAAGa 3'</td><td>-15.70</td><td>155.00</td><td rowspan="3">Show result profile</td></tr>
<tr><td>1436~1452</td><td>17</td><td>miRNA: 3' cuGAUGUGAUGUCUUCg 5'
| | |||:||||||
Target:5' gaCAAAACTGCAGAAGg 3'</td><td>-15.10</td><td>143.00</td></tr>
<tr><td>1384~1400</td><td>17</td><td>miRNA: 3' cugaugugaUGUCUUCg 5'
|||||||
Target:5' tgaaggggaACAGAAGg 3'</td><td>-7.90</td><td>140.00</td></tr>
<tr><td>163</td><td>hsa-miR-3190</td><td>183~205</td><td>23</td><td>miRNA: 3' agaGACCGGCAGAUGGAAGGUGu
5'
||| || |||||:|||
Target:5' ttcCTGAAAGTACACCTTTCACa
3'</td><td>-18.40</td><td>144.00</td><td>Show result profile</td></tr>
<tr><td>164</td><td>hsa-miR-32</td><td>247~262</td><td>16</td><td>miRNA: 3' acGUUGAAUCAUUACACGUUAu
5'
|||| || |||||||
Target:5' tgCAAC-----AA-GTGCAATt
3'</td><td>-10.10</td><td>146.00</td><td>Show result profile</td></tr>
<tr><td>165</td><td>hsa-miR-32*</td><td>1845~1866</td><td>22</td><td>miRNA: 3' uuuAUAGUGUGUGUGAUUUAAc
5'
|||||:| :| ||||:||
Target:5' ctcTATCATAGGCCCTAAGTTc
3'</td><td>-11.60</td><td>155.00</td><td>Show result profile</td></tr>
<tr><td rowspan="2">166</td><td rowspan="2">hsa-miR-3202</td><td>1604~1628</td><td>25</td><td>miRNA: 3' uaaUUUCGAGAAGA---
GGGAAGGu 5'
:||| |:|| | |||||||
Target:5'
aggGAAGATTTTATTTGCCCTTCCg 3'</td><td>-16.20</td><td>159.00</td><td rowspan="2">Show result profile</td></tr>
<tr><td>1604~1628</td><td>25</td><td>miRNA: 3' uaaUUUCGAGAAGA---
GGGAAGGu 5'
:||| |:|| | |||||||
Target:5'
aggGAAGATTTTATTTGCCCTTCCg 3'</td><td>-16.20</td><td>159.00</td></tr>
<tr><td>167</td><td>hsa-miR-320a</td><td>983~1004</td><td>22</td><td>miRNA: 3' agcgggAGAGUUGGGUCGAAAa
5'
|| || ||||||||
Target:5' ttggtgTCCCATACCAGCTTTt
3'</td><td>-16.90</td><td>156.00</td><td>Show result profile</td></tr>
<tr><td></td><td></td><td>983~1004</td><td>22</td><td>miRNA: 3' aacgggAGAGUUGGGUCGAAAa
5'</td><td>-16.60</td><td>156.00</td><td></td></tr>
</table>

168	hsa-miR-320b			|| || |||||||| Target:5' ttggtgTCCCATACCAGCTTTt<br>3'			Show result profile
		983~1004	22	miRNA: 3' aacgggAGAGUUGGGUCGAAAa<br>5'<br>|| || |||||||| Target:5' ttggtgTCCCATACCAGCTTTt<br>3'	-16.60	156.00	
169	hsa-miR-320c	985~1004	20	miRNA: 3' ugggAGAGUUGGGUCGAAAa 5'<br>|| || |||||||| Target:5' ggtgTCCCATACCAGCTTTt 3'	-14.10	156.00	Show result profile
		985~1004	20	miRNA: 3' ugggAGAGUUGGGUCGAAAa 5'<br>|| || |||||||| Target:5' ggtgTCCCATACCAGCTTTt 3'	-14.10	156.00	
170	hsa-miR-320d	986~1004	19	miRNA: 3' aggAGAGUUGGGUCGAAAa 5'<br>|| || |||||||| Target:5' gtgTCCCATACCAGCTTTt 3'	-12.70	156.00	Show result profile
		986~1004	19	miRNA: 3' aggAGAGUUGGGUCGAAAa 5'<br>|| || |||||||| Target:5' gtgTCCCATACCAGCTTTt 3'	-12.70	156.00	
171	hsa-miR-320e	986~1003	18	miRNA: 3' ggaAGAGUUGGGUCGAAa 5'<br>|| || ||||||| Target:5' gtgTCCCATACCAGCTTt 3'	-11.30	151.00	Show result profile
172	hsa-miR-326	1326~1343	18	miRNA: 3' gacCUCCUUCCCGGGUCUcc 5'<br>|:|||| ||||||| Target:5' gatGGGGAA--GCCCAGAtc 3'	-18.20	141.00	Show result profile
173	hsa-miR-330-5p	1320~1343	24	miRNA: 3' cggaUUCUGUGUC--CGGGUCUcu<br>5'<br>||||:: :| ||||||| Target:5' aggaAAGATGGGGAAGCCCAGAtc<br>3'	-14.10	140.00	Show result profile
174	hsa-miR-335*	471~497	27	miRNA: 3' ccaGUCCUC-GUU----<br>AUUACUUUUu 5'<br>|||||| ||| ||||||||:<br>Target:5'<br>cacCAGGAGTCAATGATTAATGAAAGg 3'	-16.80	157.00	Show result profile
		444~471	28	miRNA: 3' ccAGUCC-----UCGUUAU--<br>UACUUUUu 5'<br>||||| | | ||| ||||||| Target:5' aaTCAGGCATTTAAC-<br>ATACTATGAAAAc 3'	-7.10	153.00	
175	hsa-miR-338-3p	1435~1463	29	miRNA: 3' guUGUUUUAGUG------AC-<br>UACGACCu 5'<br>|||||| ::| || |||:||| Target:5'	-13.00	142.00	Show result profile

<table>
<tr><td></td><td></td><td></td><td></td><td>agACAAAACTGCAGAAGGTGTATGTTGGg 3'</td><td></td><td></td><td></td></tr>
<tr><td>176</td><td>hsa-miR-339-5p</td><td>2165~2189</td><td>25</td><td>miRNA: 3' gcAC-UCGAGGACCUC-CUGUCCCu 5'
|| | :|:: | || |||||||
Target:5' ttTGTATTTTTAGCAGAGACAGGGa 3'</td><td>-16.10</td><td>153.00</td><td>Show result profile</td></tr>
<tr><td>177</td><td>hsa-miR-346</td><td>2064~2086</td><td>23</td><td>miRNA: 3' ucuccguccguacgcCCGUCUGu 5'
|||||||
Target:5' tttttttttttttgaGGCAGACt 3'</td><td>-11.50</td><td>140.00</td><td>Show result profile</td></tr>
<tr><td rowspan="2">178</td><td rowspan="2">hsa-miR-3605-5p</td><td>509~531</td><td>23</td><td>miRNA: 3' ccGAAGGAACGAUAGGUAGGAGu 5'
| |::|||:|:||||| |||
Target:5' tcCCTTTTTGTTGTCCATACTCt 3'</td><td>-18.10</td><td>149.00</td><td rowspan="2">Show result profile</td></tr>
<tr><td>2308~2330</td><td>23</td><td>miRNA: 3' ccgaaggAACGAUAGGUAGGAgu 5'
|| :| ||||||||
Target:5' atttagaTTTTTCTCCATCCTgt 3'</td><td>-10.50</td><td>140.00</td></tr>
<tr><td>179</td><td>hsa-miR-3606</td><td>1766~1786</td><td>21</td><td>miRNA: 3' uuaAUUUUAUCGGAAGUGAUu 5'
|: |:|:||:||||:||
Target:5' aaaTGTAGTGGCTTTCATTAa 3'</td><td>-15.30</td><td>150.00</td><td>Show result profile</td></tr>
<tr><td>180</td><td>hsa-miR-3607-5p</td><td>2518~2545</td><td>28</td><td>miRNA: 3' ugACU--A-AACG-AAGU--AGUGUACg 5'
||| | |||: ||||
|:|||||
Target:5' ttTGAAGTATTGTATTCAGTTTACATGc 3'</td><td>-12.00</td><td>144.00</td><td>Show result profile</td></tr>
<tr><td>181</td><td>hsa-miR-3607-3p</td><td>1037~1053</td><td>17</td><td>miRNA: 3' guaGUCUUUCGCAAAUGUCa 5'
::|:|| |||||||
Target:5' tggTGGGAA---TTTACAGt 3'</td><td>-7.80</td><td>146.00</td><td>Show result profile</td></tr>
<tr><td>182</td><td>hsa-miR-361-5p</td><td>650~672</td><td>23</td><td>miRNA: 3' cauggGACC-UCUAAGACUAUu 5'
|||| || ||:|||||
Target:5' tgttaaCTGGAAGCTTTTGATAa 3'</td><td>-14.40</td><td>148.00</td><td>Show result profile</td></tr>
<tr><td>183</td><td>hsa-miR-3613-3p</td><td>2055~2078</td><td>24</td><td>miRNA: 3' cuucccAACCCGAAAAAAAAAACa 5'
||| :||||||||||
Target:5' tttgttTTGTTTTTTTTTTTTTGa 3'</td><td>-7.60</td><td>170.00</td><td>Show result profile</td></tr>
<tr><td rowspan="2">184</td><td rowspan="2">hsa-miR-3614-3p</td><td>105~128</td><td>24</td><td>miRNA: 3' uuUUGUGGUUCUAGA-CUUCCGAu 5'
::||||| || | ||||:|
Target:5' ccGGCACCACCATGTCGAAGGTTt 3'</td><td>-16.30</td><td>149.00</td><td rowspan="2">Show result profile</td></tr>
<tr><td></td><td></td><td></td><td></td><td></td></tr>
</table>

<table>
<tr><td></td><td></td><td>1221~1242</td><td>22</td><td>miRNA: 3' uuUUGUGGUUCUAGACUUC-CGAu 5'
||:|| ||||| |||| |||
Target:5' gaAATAC--AGATCGGAAGTGCTg 3'</td><td>-16.50</td><td>141.00</td><td></td></tr>
<tr><td rowspan="2">185</td><td rowspan="2">hsa-miR-3619</td><td>2333~2356</td><td>24</td><td>miRNA: 3' cgaCGUG--GUCGGACGGACGACu 5'
|||| | ::|||||||||
Target:5' ctaGCACAAAAAATTTGCCTGCTGt 3'</td><td>-24.00</td><td>161.00</td><td rowspan="2">Show result profile</td></tr>
<tr><td>218~249</td><td>32</td><td>miRNA: 3' cgACGUGGUC-----GGA---C--GGACGACu 5'
|||| ||| ::| | |||||||
Target:5' ttTGCAGCAGAAGAATTTAAAGTTCCTGCTGc 3'</td><td>-18.70</td><td>146.00</td></tr>
<tr><td>186</td><td>hsa-miR-363</td><td>241~262</td><td>22</td><td>miRNA: 3' auGUCUACCUAUG-GCACGUUAa 5'
| | || | || |||||||
Target:5' tcCTGCTGCA-ACAAGTGCAATt 3'</td><td>-7.60</td><td>147.00</td><td>Show result profile</td></tr>
<tr><td>187</td><td>hsa-miR-3646</td><td>1599~1620</td><td>22</td><td>miRNA: 3' acccgaCCCGAGUAAAGUAAAa 5'
||| ||||:||||
Target:5' gtgtaaGGGAAGATTTTATTTg 3'</td><td>-8.70</td><td>140.00</td><td>Show result profile</td></tr>
<tr><td>188</td><td>hsa-miR-3648</td><td>60~81</td><td>22</td><td>miRNA: 3' gggAGC-CGCUAGGGGCGCCga 5'
||| || | ||||||||
Target:5' aagTCGTGCTACCCCCGCGGag 3'</td><td>-24.00</td><td>146.00</td><td>Show result profile</td></tr>
<tr><td>189</td><td>hsa-miR-3660</td><td>556~577</td><td>22</td><td>miRNA: 3' aguuUUACGA-GAGGACAGUca 5'
|| ||| || ||||||
Target:5' aattAAGGCTACTACTGTCAca 3'</td><td>-10.70</td><td>141.00</td><td>Show result profile</td></tr>
<tr><td>190</td><td>hsa-miR-3662</td><td>869~892</td><td>24</td><td>miRNA: 3' guaGUCAGUGAUGAGUAGUAAAag 5'
:| | | | :|:|||||||
Target:5' ctaTATTAAATTTTTATCATTTac 3'</td><td>-7.40</td><td>141.00</td><td>Show result profile</td></tr>
<tr><td>191</td><td>hsa-miR-3664</td><td>1164~1183</td><td>20</td><td>miRNA: 3' ugaGUACUCACUUC-UGUCUCAa 5'
||| | |:| |||||||
Target:5' tagCAT---TCAGGAACAGAGTa 3'</td><td>-9.60</td><td>148.00</td><td>Show result profile</td></tr>
<tr><td>192</td><td>hsa-miR-3667-3p</td><td>2177~2196</td><td>20</td><td>miRNA: 3' uuUCUGGGUACCUCUCCUUCca 5'
||| || |:|||||||
Target:5' gcAGAGACA--GGGAGGAAGtt 3'</td><td>-16.60</td><td>140.00</td><td>Show result profile</td></tr>
<tr><td></td><td>hsa-</td><td></td><td></td><td>miRNA: 3' auAUAAGGCAUAUGUAUAAGGCa 5'</td><td></td><td></td><td></td></tr>
</table>

<table>
<tr><td>193</td><td>miR-3669</td><td>88~108</td><td>21</td><td>|:|||:| || | ||||||
Target:5' tgTGTTCTGGATTC--ATTCCGg 3'</td><td>-15.70</td><td>140.00</td><td>Show result profile</td></tr>
<tr><td rowspan="2">194</td><td rowspan="2">hsa-miR-367*</td><td>1354~1375</td><td>22</td><td>miRNA: 3' ucucaacguauaaUCGUUGUCa 5'
||||||||
Target:5' tattaaggaggaaAGCAACAGa 3'</td><td>-10.70</td><td>145.00</td><td rowspan="2">Show result profile</td></tr>
<tr><td>206~228</td><td>23</td><td>miRNA: 3' ucUCA-ACGUAUAAUCGUUGUCa 5'
||| | | :|| |||:|||
Target:5' gcAGTCTTAAAGTTTGCAGCAGa 3'</td><td>-8.50</td><td>140.00</td></tr>
<tr><td>195</td><td>hsa-miR-367</td><td>240~262</td><td>23</td><td>miRNA: 3' aguGGUAACGAU-UUCACGUUAa 5'
|:: ||| | |||||||||
Target:5' ttcCTGCTGCAACAAGTGCAATt 3'</td><td>-14.60</td><td>163.00</td><td>Show result profile</td></tr>
<tr><td rowspan="3">196</td><td rowspan="3">hsa-miR-3672</td><td>1483~1504</td><td>22</td><td>miRNA: 3' uucUACAAAAUGUACUCAGAGUa 5'
|| || |||| |||||:|
Target:5' aaaATACTTGACAT-AGTCTTAa 3'</td><td>-8.60</td><td>147.00</td><td rowspan="3">Show result profile</td></tr>
<tr><td>193~215</td><td>23</td><td>miRNA: 3' uucuacAAAAUGUACUCAGAGUa 5'
||| ||| |||||:|
Target:5' tacaccTTTCACAGCAGTCTTAa 3'</td><td>-7.30</td><td>145.00</td></tr>
<tr><td>2232~2262</td><td>31</td><td>miRNA: 3' uucUACAAAAUGU--------ACUCAGAGUa 5'
:| | ||||| |||||||:|
Target:5' cccGTTTCTTACAATAAATGTTGAGTCTTAg 3'</td><td>-10.20</td><td>142.00</td></tr>
<tr><td rowspan="2">197</td><td rowspan="2">hsa-miR-3673</td><td>1064~1085</td><td>22</td><td>miRNA: 3' auAAGGCAU-AUAUGUAAGGua 5'
| |:| : ||||||||||
Target:5' ttTGCTGAGTTATACATTCCtt 3'</td><td>-13.90</td><td>143.00</td><td rowspan="2">Show result profile</td></tr>
<tr><td>90~108</td><td>19</td><td>miRNA: 3' auAAGGCAUAUAUGUAAGGUa 5'
|||:| || | ||||||:
Target:5' tgTTCTGGAT-T-CATTCCGg 3'</td><td>-14.40</td><td>141.00</td></tr>
<tr><td>198</td><td>hsa-miR-3675-5p</td><td>2212~2237</td><td>26</td><td>miRNA: 3' cuUUAG---AGAUGUCUUCGGGGUAu 5'
|||| | | ||||:|||||:|
Target:5' aaAATCTGATGTTCAGAGGCCCCGTt 3'</td><td>-24.10</td><td>157.00</td><td>Show result profile</td></tr>
<tr><td>199</td><td>hsa-miR-3675-</td><td>334~356</td><td>23</td><td>miRNA: 3' aacCCCCUCA-AGGAAUCUCUAc 5'
| ||| | |:| |||||||
Target:5' actGCGGATTATTCCTAGAGATc</td><td>-12.10</td><td>159.00</td><td>Show result profile</td></tr>
</table>

<table>
<tr><td></td><td>3p</td><td></td><td></td><td>3'</td><td></td><td></td><td></td></tr>
<tr><td>200</td><td>hsa-miR-3677</td><td>141~161</td><td>21</td><td>miRNA: 3' ccGGCACCGGUCUCGGGUGCUc 5'
|:| |:|:|| ||||||:
Target:5' cgCTGACGTCGGA-CCCACGGc 3'</td><td>-23.50</td><td>143.00</td><td>Show result profile</td></tr>
<tr><td>201</td><td>hsa-miR-3679-3p</td><td>1857~1877</td><td>21</td><td>miRNA: 3' cuacUUCUAAUGACCCCCCUUc 5'
||| | |:| |||||||
Target:5' ccctAAGTTCATT-GGGGGAAa 3'</td><td>-14.60</td><td>157.00</td><td>Show result profile</td></tr>
<tr><td>202</td><td>hsa-miR-3680*</td><td>1629~1652</td><td>24</td><td>miRNA: 3' ggaugagGGUCCCAG-UACGUUUu 5'
:||| || |||||||
Target:5' gaagaaaTCAGTATCTATGCAAAt 3'</td><td>-10.00</td><td>152.00</td><td>Show result profile</td></tr>
<tr><td>203</td><td>hsa-miR-3686</td><td>769~799</td><td>31</td><td>miRNA: 3' agUAAAUGAA-------AGA--GAAUGUCUa 5'
||||:||| ||| :||:||||
Target:5' caATTTGCTTGATAGCATCTGATTTGCAGAc 3'</td><td>-10.41</td><td>146.00</td><td>Show result profile</td></tr>
<tr><td>204</td><td>hsa-miR-3688</td><td>1790~1811</td><td>22</td><td>miRNA: 3' ucucacCGUUUCAGAAAGGUau 5'
|:||||||||||:|
Target:5' cttgctGTAAAGTCTTTCTAag 3'</td><td>-15.20</td><td>140.00</td><td>Show result profile</td></tr>
<tr><td>205</td><td>hsa-miR-369-3p</td><td>1170~1188</td><td>19</td><td>miRNA: 3' uuUCUAGUUGGUACAUAAUAa 5'
||: ||| : |||||||
Target:5' tcAGG--AACAGAGTATTATt 3'</td><td>-7.60</td><td>151.00</td><td>Show result profile</td></tr>
<tr><td>206</td><td>hsa-miR-370</td><td>1957~1978</td><td>22</td><td>miRNA: 3' ugguccaaggugggGUCGUCCg 5'
|||||||
Target:5' atgtgacaaatgaaCAGCAGGa 3'</td><td>-13.70</td><td>140.00</td><td>Show result profile</td></tr>
<tr><td>207</td><td>hsa-miR-371-5p</td><td>2016~2035</td><td>20</td><td>miRNA: 3' ucacggGGGUGUCAAACUCa 5'
:|:: |||||||
Target:5' aaaaaaTCTGATGTTTGAGg 3'</td><td>-9.90</td><td>142.00</td><td>Show result profile</td></tr>
<tr><td>208</td><td>hsa-miR-372</td><td>1888~1910</td><td>23</td><td>miRNA: 3' ugcGAGUUUACAGCGUCGUGAAa 5'
:|||| | |||||:||
Target:5' agaTTCAACAGAATCAGCATTTg 3'</td><td>-9.90</td><td>140.00</td><td>Show result profile</td></tr>
<tr><td>209</td><td>hsa-miR-373*</td><td>1128~1149</td><td>22</td><td>miRNA: 3' ccUUUCGCGGGGGUAAAACUCa 5'
||| | |: ::|||||||
Target:5' agAAACCACTGGTGTTTTGAGg 3'</td><td>-8.80</td><td>156.00</td><td>Show result profile</td></tr>
</table>

<table>
<tr><td rowspan="2">210</td><td rowspan="2">hsa-miR-373</td><td>1011~1033</td><td>23</td><td>miRNA: 3' ugUGGG-GUUUUAGCUUCGUGAAg 5'
|||: :|:|||| ||:||||
Target:5' ggACCTATAGAATC-CAGTACTTt 3'</td><td>-14.30</td><td>148.00</td><td rowspan="2">Show result profile</td></tr>
<tr><td>1888~1910</td><td>23</td><td>miRNA: 3' ugUGGG--GUUUUAGCUUCGUGAAg 5'
|::| ||:|||| ||||:||
Target:5' agATTCAACAGAATC--AGCATTTg 3'</td><td>-11.90</td><td>147.00</td></tr>
<tr><td>211</td><td>hsa-miR-374a</td><td>2572~2597</td><td>26</td><td>miRNA: 3' gugaaUA-GUCCAAC---AUAAUAUu 5'
|| || |||| ||||:||
Target:5' tgaaaATACATGTTGTTATATTGTAa 3'</td><td>-7.50</td><td>141.00</td><td>Show result profile</td></tr>
<tr><td>212</td><td>hsa-miR-374b*</td><td>366~384</td><td>19</td><td>miRNA: 3' uuACUAUUAUGUUGGACGAUuc 5'
|| |||| |:||||||
Target:5' gtTGTTAAT---ATCTGCTAct 3'</td><td>-7.40</td><td>141.00</td><td>Show result profile</td></tr>
<tr><td rowspan="3">213</td><td rowspan="3">hsa-miR-376a*</td><td>216~237</td><td>22</td><td>miRNA: 3' auGAGUAUCUUCCUCUUAGAUg 5'
:|:: || || |||||:||
Target:5' agTTTGCAGCAGAAGAATTTAa 3'</td><td>-9.50</td><td>148.00</td><td rowspan="3">Show result profile</td></tr>
<tr><td>2254~2278</td><td>25</td><td>miRNA: 3' augAGUAUC--UUCCU-CUUAGAUg 5'
|| ||| ||| | ||||:||
Target:5' gagTCTTAGTTAAGCAGGAATTTAt 3'</td><td>-7.80</td><td>145.00</td></tr>
<tr><td>1028~1050</td><td>23</td><td>miRNA: 3' auGAGUAUCUUC-CUCUUAGAUg 5'
||: || :| |:||||:||
Target:5' taCTTTTAATGGTGGGAATTTAc 3'</td><td>-13.40</td><td>140.00</td></tr>
<tr><td>214</td><td>hsa-miR-376b</td><td>1917~1938</td><td>22</td><td>miRNA: 3' uuGUACCUAAAAGGAGAUACUa 5'
||| |:| |: |:|||||
Target:5' acCATTGGTAGTTGTTTATGAa 3'</td><td>-8.30</td><td>144.00</td><td>Show result profile</td></tr>
<tr><td>215</td><td>hsa-miR-377</td><td>2339~2361</td><td>23</td><td>miRNA: 3' ugUUUUCAACGGA-AACACACUa 5'
|||| |||||| ||||| |
Target:5' caAAAATTTGCCTGCTGTGTTAc 3'</td><td>-11.82</td><td>144.00</td><td>Show result profile</td></tr>
<tr><td>216</td><td>hsa-miR-381</td><td>2151~2172</td><td>22</td><td>miRNA: 3' ugucucUCGAACGGGAACAUAu 5'
|||| :::||||||
Target:5' acgcccAGCTAATTTTTGTATt 3'</td><td>-9.80</td><td>140.00</td><td>Show result profile</td></tr>
<tr><td></td><td>hsa-</td><td></td><td></td><td>miRNA: 3' ugUACAGGUAUAA--UACGCAAu</td><td></td><td></td><td></td></tr>
</table>

217	miR-3912	[2527~2549]	23	5'<br>:| |:|| || ||||||| Target:5' ttGTATTCAGTTTACATGCGTTa<br>3'	-10.10	153.00	Show result profile
218	hsa-miR-3916	[104~134]	31	miRNA: 3' gacUCUUGGUCGGUAAAG------<br>AAGGAGAa 5'<br>:| |||| |||| || |||||:| Target:5' tccGGCACCA-<br>CCATGTCGAAGGTTTCCTTTa 3'	-15.20	148.00	Show result profile
219	hsa-miR-3918	[1842~1862]	21	miRNA: 3' ucAGAGGUAGACGCCGGGAca 5'<br>||||:||| |||||| Target:5' ctTCTCTATCATAGGCCCTaa 3'	-20.10	147.00	Show result profile
220	hsa-miR-3921	[2208~2230]	23	miRNA: 3' uguuccgUAUACCAUGAGUCUCu<br>5'<br>|| || |::|||||| Target:5' aaacaaaATCTGATGTTCAGAGg<br>3'	-13.40	144.00	Show result profile
221	hsa-miR-3928	[224~246]	23	miRNA: 3' cggCUUC-GAGGUUCCAAGGAgg<br>5'<br>|||| |::|| |||||| Target:5' gcaGAAGAATTTAAAGTTCCTgc<br>3'	-14.50	143.00	Show result profile
		[167~189]	23	miRNA: 3' cgGCUUCGAG-GUUCCAAGGAgg<br>5'<br>| |||: | ||: |||||| Target:5' taCAAAGTACTCAGTGTTCCTga<br>3'	-11.30	140.00	
222	hsa-miR-3929	[2094~2118]	25	miRNA: 3' ucacCAGAUGAG--<br>UGUAGUCGGAg 5'<br>| || ||| :| ||||||| Target:5'<br>gccaGGCTTCTCCTGCCTCAGCCTc 3'	-18.70	157.00	Show result profile
223	hsa-miR-3934	[172~201]	30	miRNA: 3' gacgGAGUCA-AAGG-------<br>UGUGGACu 5'<br>|||||| |||| |||||| Target:5'<br>agtaCTCAGTGTTCCTGAAAGTACACCTtt 3'	-16.91	142.00	Show result profile
224	hsa-miR-3935	[1520~1541]	22	miRNA: 3' caccgaccacgagCAUAGAUGu<br>5'<br>|||||||| Target:5' tagagaaaacaaaGTATCTACt<br>3'	-8.60	145.00	Show result profile
		[356~381]	26	miRNA: 3' cacCGACC----<br>ACGAGCAUAGAUGu 5'<br>|:||| ||:| |||||:| Target:5'<br>cgtGTTGGAAGTTGTTAATATCTGCt 3'	-13.20	141.00	
225	hsa-miR-	[1556~1577]	22	miRNA: 3' acguaGACGGUAUGUGGGGAAu<br>5'<br>|| | | |:|||||||	-15.60	157.00	Show result profile

<table>
<tr><td></td><td>3936</td><td></td><td></td><td>Target:5' acagaCTTCAAAATACCCCTTa 3'</td><td></td><td></td><td></td></tr>
<tr><td rowspan="3">226</td><td rowspan="3">hsa-miR-3938</td><td>529~549</td><td>21</td><td>miRNA: 3' ggcccaAUAGAUGUUCCCUUAa 5'
                ||| | :|||||||
Target:5' tcttccTAT-GAAGAGGGAATg 3'</td><td>-9.10</td><td>147.00</td><td rowspan="3">Show result profile</td></tr>
<tr><td>1460~1481</td><td>22</td><td>miRNA: 3' ggCCCAAUAGAUGUUCCCUUaa 5'
            ||| | ||: |||||||
Target:5' tgGGGAGAACTGAAAGGGAAaa 3'</td><td>-16.10</td><td>144.00</td></tr>
<tr><td>1590~1611</td><td>22</td><td>miRNA: 3' ggccCAAUAGAUGUUCCCUUaa 5'
              | |:| |::|||||||
Target:5' gaatGATGTGTGTAAGGGAAga 3'</td><td>-12.70</td><td>142.00</td></tr>
<tr><td>227</td><td>hsa-miR-3941</td><td>1579~1604</td><td>26</td><td>miRNA: 3' auacUAGGAGUC--A--ACACACAUu 5'
              |||| || |||||||||
Target:5' gagaATCCAAAGAATGATGTGTGTAa 3'</td><td>-13.60</td><td>154.00</td><td>Show result profile</td></tr>
<tr><td>228</td><td>hsa-miR-3942</td><td>1167~1192</td><td>26</td><td>miRNA: 3' uaAAGUCCAUUGUC-----AUAACGaa 5'
            |||||| ||||| ||||||
Target:5' caTTCAGG-AACAGAGTATTATTGCac 3'</td><td>-15.20</td><td>151.00</td><td>Show result profile</td></tr>
<tr><td rowspan="3">229</td><td rowspan="3">hsa-miR-410</td><td>2574~2593</td><td>20</td><td>miRNA: 3' uguccgGUAGACACAAUAUAa 5'
                ||| | ||||||||
Target:5' aaaataCATGT-TGTTATATt 3'</td><td>-7.90</td><td>154.00</td><td rowspan="3">Show result profile</td></tr>
<tr><td>1234~1254</td><td>21</td><td>miRNA: 3' ugUC-CGGUAGACACAAUAUAa 5'
            || ||:: || |||||||
Target:5' gaAGTGCTG-ATGAGTTATATt 3'</td><td>-8.50</td><td>150.00</td></tr>
<tr><td>902~922</td><td>21</td><td>miRNA: 3' uguccggUAGACACAAUAUAa 5'
                 | || |||||||
Target:5' gattcagACCTCAGTTATATa 3'</td><td>-8.40</td><td>146.00</td></tr>
<tr><td>230</td><td>hsa-miR-411*</td><td>2343~2364</td><td>22</td><td>miRNA: 3' ccAAUCACCUGGCACAAUGUau 5'
            || | :|:||||||||
Target:5' aaTTTGCCTGCTGTGTTACAaa 3'</td><td>-12.30</td><td>140.00</td><td>Show result profile</td></tr>
<tr><td>231</td><td>hsa-miR-423-5p</td><td>1607~1627</td><td>21</td><td>miRNA: 3' uuUCAGAGCGAGAGACGGGGAGu 5'
            || :| :| |:|||||:||
Target:5' gaAGATT--TTATTTGCCCTTCc 3'</td><td>-13.90</td><td>141.00</td><td>Show result profile</td></tr>
</table>

<table>
<tr><td rowspan="2">232</td><td rowspan="2">hsa-miR-4259</td><td>1250~1271</td><td>22</td><td>miRNA: 3' aggacuggggaUCUGGGUUGAc 5'
| ||||||||
Target:5' atatttattgaAAACCCAACTt 3'</td><td>-11.10</td><td>147.00</td><td rowspan="2">Show result profile</td></tr>
<tr><td>2139~2161</td><td>23</td><td>miRNA: 3' aggAC-UGGGGAUCUGGGUUGAc 5'
|| ||| | | :||||:||
Target:5' gcaTGCACCACCACGCCCAGCTa 3'</td><td>-15.50</td><td>143.00</td></tr>
<tr><td>233</td><td>hsa-miR-4262</td><td>1705~1721</td><td>17</td><td>miRNA: 3' gucCAUCAGACUUACag 5'
||| ||||||||
Target:5' cctGTATTCTGAATGgt 3'</td><td>-10.50</td><td>142.00</td><td>Show result profile</td></tr>
<tr><td>234</td><td>hsa-miR-4267</td><td>1751~1766</td><td>16</td><td>miRNA: 3' cacgguggCUCGACCu 5'
|||||||
Target:5' tctttttaGAGCTGGa 3'</td><td>-13.00</td><td>140.00</td><td>Show result profile</td></tr>
<tr><td>235</td><td>hsa-miR-4269</td><td>1732~1749</td><td>18</td><td>miRNA: 3' cgGUCCCGACAGACACGGACg 5'
||||| | ||||:|||
Target:5' agCAGGGAT---TGTGTCTGg 3'</td><td>-21.10</td><td>144.00</td><td>Show result profile</td></tr>
<tr><td>236</td><td>hsa-miR-4274</td><td>288~305</td><td>18</td><td>miRNA: 3' gucccCCUCCCUGACGAc 5'
| | |||||||
Target:5' atcctGCACAGACTGCTg 3'</td><td>-12.90</td><td>141.00</td><td>Show result profile</td></tr>
<tr><td rowspan="3">237</td><td rowspan="3">hsa-miR-4277</td><td>318~338</td><td>21</td><td>miRNA: 3' cacaUG-ACACGAGUCUUGACg 5'
|| || |:|||||||||
Target:5' taaaACATG-GTTCAGAACTGc 3'</td><td>-20.00</td><td>160.00</td><td rowspan="3">Show result profile</td></tr>
<tr><td>1452~1472</td><td>21</td><td>miRNA: 3' caCAUGACACGAGUCUUGACg 5'
|||: || |||||||
Target:5' gtGTATGTTGGGGAGAACTGa 3'</td><td>-13.40</td><td>151.00</td></tr>
<tr><td>1745~1765</td><td>21</td><td>miRNA: 3' cacaUGACACGAGUCUUGACg 5'
:|| | :|:|||:|||
Target:5' tctgGCTCTTTTTAGAGCTGg 3'</td><td>-14.70</td><td>141.00</td></tr>
<tr><td>238</td><td>hsa-miR-429</td><td>1138~1158</td><td>21</td><td>miRNA: 3' ugcCAAAAUGGUCUGUCAUAAu 5'
|||||: :||:||||||
Target:5' ggtGTTTTG-AGGATAGTATTt 3'</td><td>-13.80</td><td>150.00</td><td>Show result profile</td></tr>
<tr><td>239</td><td>hsa-miR-4291</td><td>235~250</td><td>16</td><td>miRNA: 3' ucgaCAAGGACGACuu 5'
||||||||||
Target:5' taaaGTTCCTGCTGca 3'</td><td>-16.40</td><td>140.00</td><td>Show result profile</td></tr>
<tr><td>240</td><td>hsa-miR-4298</td><td>973~994</td><td>22</td><td>miRNA: 3' gacggaggaggaggACAGGGUc 5'
|||||||
Target:5' tgtgtataaattggTGTCCCAt</td><td>-10.60</td><td>140.00</td><td>Show result profile</td></tr>
</table>

<table>
<tr><td></td><td></td><td></td><td></td><td>3'</td><td></td><td></td><td></td></tr>
<tr><td rowspan="2">241</td><td rowspan="2">hsa-miR-4299</td><td>2083~2099</td><td>17</td><td>miRNA: 3' cgGAGAGUACAGUGGUCg 5'
          ||||| ||||:||||
Target:5' gaCTCTC-TGTCGCCAGg 3'</td><td>-22.70</td><td>151.00</td><td rowspan="2">Show result profile</td></tr>
<tr><td>1332~1349</td><td>18</td><td>miRNA: 3' cggaGAGUACAGUGGUCg 5'
            | || |||||||
Target:5' gaagCCCAGATCACCAGg 3'</td><td>-13.60</td><td>146.00</td></tr>
<tr><td>242</td><td>hsa-miR-4307</td><td>1511~1532</td><td>22</td><td>miRNA: 3' ccUUUGUC---CUUUUUUGUaa
5'
          |::||| ||:||||||
Target:5' gaAGGCAGTTAGAGAAAACAaa
3'</td><td>-9.90</td><td>141.00</td><td>Show result profile</td></tr>
<tr><td>243</td><td>hsa-miR-4311</td><td>1080~1099</td><td>20</td><td>miRNA: 3' guGUGAGUCG--AGAGAAAg 5'
          | :|:| | |||||||
Target:5' ttCCTTTATCAATCTCTTTt 3'</td><td>-7.60</td><td>146.00</td><td>Show result profile</td></tr>
<tr><td>244</td><td>hsa-miR-4316</td><td>2104~2121</td><td>18</td><td>miRNA: 3' guGGUCGA-UCGGAGUGg 5'
          |:: || ||||||||
Target:5' tcCTGCCTCAGCCTCACg 3'</td><td>-16.10</td><td>151.00</td><td>Show result profile</td></tr>
<tr><td rowspan="3">245</td><td rowspan="3">hsa-miR-4320</td><td>1009~1026</td><td>18</td><td>miRNA: 3' ucCUUCGAUGUCUUAGGg 5'
          |:| |||:|||||||
Target:5' gtGGACCTATAGAATCCa 3'</td><td>-17.40</td><td>164.00</td><td rowspan="3">Show result profile</td></tr>
<tr><td>1569~1587</td><td>19</td><td>miRNA: 3' uccuucGAUG-UCUUAGGg 5'
              :||: |||||||
Target:5' taccccTTATGAGAATCCa 3'</td><td>-9.00</td><td>144.00</td></tr>
<tr><td>1884~1904</td><td>21</td><td>miRNA: 3' ucCUUC---GAUGUCUUAGgg 5'
          |||| | ||||||||
Target:5' aaGAAGATTCAACAGAATCag 3'</td><td>-12.40</td><td>140.00</td></tr>
<tr><td>246</td><td>hsa-miR-4325</td><td>243~260</td><td>18</td><td>miRNA: 3' agUGACUCUGUUCACGUu 5'
          :||| |||||||||
Target:5' ctGCTGCAACAAGTGCAa 3'</td><td>-16.60</td><td>160.00</td><td>Show result profile</td></tr>
<tr><td>247</td><td>hsa-miR-4326</td><td>2174~2195</td><td>22</td><td>miRNA: 3' cagaccCUCUGU--CUCCUUgu
5'
              |||||| ||||||
Target:5' ttagcaGAGACAGGGAGGAAgt
3'</td><td>-16.20</td><td>140.00</td><td>Show result profile</td></tr>
<tr><td>248</td><td>hsa-miR-4328</td><td>1425~1446</td><td>22</td><td>miRNA: 3' uuagGACC---C--UUUUGACc
5'
            |||| | |||||||
Target:5' cggaCTGGATAGACAAAACTGc
3'</td><td>-8.10</td><td>143.00</td><td>Show result profile</td></tr>
<tr><td>249</td><td>hsa-miR-4330</td><td>716~734</td><td>19</td><td>miRNA: 3' cguuccgaGACUAGACUCc 5'
                :| ||||||||
Target:5' cgtatataTTCATCTGAGa 3'</td><td>-7.70</td><td>143.00</td><td>Show result profile</td></tr>
</table>

<table>
<tr><td></td><td></td><td></td><td></td><td></td><td></td><td></td><td></td></tr>
<tr><td>250</td><td>hsa-miR-449c</td><td>373~403</td><td>31</td><td>miRNA: 3' ugUCGGCGAU----CGU-UAUG-UGACGGAu 5'
| |:|||| | | ||||
|:|||||
Target:5' atATCTGCTACTTGGAACATACGATTGCCTt 3'</td><td>-15.60</td><td>149.00</td><td>Show result profile</td></tr>
<tr><td rowspan="2">251</td><td rowspan="2">hsa-miR-450b-5p</td><td>1167~1194</td><td>28</td><td>miRNA: 3' auAAGUCCUUGU------AUAACGUUUu 5'
||||||||||
||||||| |
Target:5' caTTCAGGAACAGAGTATTATTGCACAg 3'</td><td>-18.50</td><td>150.00</td><td rowspan="2">Show result profile</td></tr>
<tr><td>2576~2599</td><td>24</td><td>miRNA: 3' auAAGUCC--UUGUAUAACGUUUu 5'
| || | : :||||||:|||
Target:5' aaTACATGTTGTTATATTGTAAAc 3'</td><td>-7.20</td><td>142.00</td></tr>
<tr><td>252</td><td>hsa-miR-452</td><td>1364~1385</td><td>22</td><td>miRNA: 3' agucaaaggagaCGUUUGUCAa 5'
| |||||||
Target:5' gaaagcaacagaGGAAACAGTg 3'</td><td>-7.40</td><td>142.00</td><td>Show result profile</td></tr>
<tr><td>253</td><td>hsa-miR-452*</td><td>2494~2518</td><td>25</td><td>miRNA: 3' gugAAUGAAGAA--ACGUC-UACUc 5'
| |:||||| ||||| ||||
Target:5' ctgTAATTTCTTTGTGCAGAATGAt 3'</td><td>-12.10</td><td>141.00</td><td>Show result profile</td></tr>
<tr><td>254</td><td>hsa-miR-454</td><td>1172~1194</td><td>23</td><td>miRNA: 3' ugggauaUUCGUUAUAACGUGau 5'
:||:| ||||||||
Target:5' aggaacaGAGTATTATTGCACag 3'</td><td>-11.30</td><td>144.00</td><td>Show result profile</td></tr>
<tr><td>255</td><td>hsa-miR-466</td><td>957~979</td><td>23</td><td>miRNA: 3' uacacacaacgcacAUACACAUa 5'
||||||||
Target:5' tttaatctaaccctTATGTGTAt 3'</td><td>-7.60</td><td>145.00</td><td>Show result profile</td></tr>
<tr><td>256</td><td>hsa-miR-485-5p</td><td>2098~2119</td><td>22</td><td>miRNA: 3' cuuAAGUAGUGCCG-GUCGGAGa 5'
||| || :| | |||||||
Target:5' ggcTTC-TCCTGCCTCAGCCTCa 3'</td><td>-14.60</td><td>154.00</td><td>Show result profile</td></tr>
<tr><td>257</td><td>hsa-miR-486-3p</td><td>492~513</td><td>22</td><td>miRNA: 3' uaggaCA-UGACUCGACGGGGc 5'
|| ||| | |||:|||
Target:5' gaaagGTGACTCATCTGTCCCt 3'</td><td>-11.80</td><td>140.00</td><td>Show result profile</td></tr>
<tr><td></td><td></td><td>183~204</td><td>22</td><td>miRNA: 3' cuGGUUCUUUAU-CGGAAAGUu 5'
|| : ||:|| |||||||
Target:5' ttCCTGAAAGTACACCTTTCAc 3'</td><td>-9.80</td><td>155.00</td><td></td></tr>
</table>

<table>
<tr><td rowspan="2">258</td><td rowspan="2">hsa-miR-488</td><td></td><td></td><td></td><td></td><td></td><td rowspan="2">Show result profile</td></tr>
<tr><td>381~407</td><td>27</td><td>miRNA: 3' cuGGUUCUU-UAU-----
CGGAAAGUu 5'
|: :||| |||
||||||||
Target:5'
taCTTGGAACATACGATTGCCTTTCAg 3'</td><td>-12.60</td><td>155.00</td></tr>
<tr><td>259</td><td>hsa-miR-494</td><td>1816~1837</td><td>22</td><td>miRNA: 3' cuccAAAGGGCACAUACAAAGu
5'
|| :|: | |||||||
Target:5' attaTTATCTTAGCATGTTTCa
3'</td><td>-7.10</td><td>150.00</td><td>Show result profile</td></tr>
<tr><td>260</td><td>hsa-miR-495</td><td>1908~1933</td><td>26</td><td>miRNA: 3' uuCUUCACGUGGUA--CA--
AACAAa 5'
||||||:||||| ||
|||||
Target:5'
ttGAAGTGTACCATTGGTAGTTGTTt 3'</td><td>-20.00</td><td>146.00</td><td>Show result profile</td></tr>
<tr><td>261</td><td>hsa-miR-496</td><td>1769~1793</td><td>25</td><td>miRNA: 3' cucUAACCG---
GUACAUUAUGAGu 5'
| |||| ||| ||||||:
Target:5'
tgtAGTGGCTTTCATTAAATACTTg 3'</td><td>-8.00</td><td>143.00</td><td>Show result profile</td></tr>
<tr><td rowspan="4">262</td><td rowspan="4">hsa-miR-499-5p</td><td>192~215</td><td>24</td><td>miRNA: 3' uuUGU----
AGUGACGUUCAGAAUu 5'
||| |||| || |||||||
Target:5' gtACACCTTTCACAGC-
AGTCTTAa 3'</td><td>-13.10</td><td>160.00</td><td rowspan="4">Show result profile</td></tr>
<tr><td>2240~2262</td><td>23</td><td>miRNA: 3' uuUG-UAGUGAC-GUUCAGAAUu
5'
|| || | || ::|||||||
Target:5' ttACAATAAATGTTGAGTCTTAg
3'</td><td>-8.60</td><td>155.00</td></tr>
<tr><td>1482~1504</td><td>23</td><td>miRNA: 3' uuUGUAGUGAC-GU-UCAGAAUu
5'
| || :|| || |||||||
Target:5' caAAATACTTGACATAGTCTTAa
3'</td><td>-9.60</td><td>151.00</td></tr>
<tr><td>1785~1807</td><td>23</td><td>miRNA: 3' uuUGU-AGUGACG-UUCAGAAuu
5'
|:| |::|||: |||||||
Target:5' aaATACTTGCTGTAAAGTCTTtc
3'</td><td>-10.90</td><td>143.00</td></tr>
<tr><td>263</td><td>hsa-miR-500a</td><td>1720~1742</td><td>23</td><td>miRNA: 3' agagUGGGUCCAUCGUUCCUAAu
5'
||:| || ||||:|||||
Target:5' gtgaACTCTGGAAGCAGGGATTg
3'</td><td>-22.30</td><td>159.00</td><td>Show result profile</td></tr>
<tr><td>264</td><td>hsa-miR-500b</td><td>1724~1741</td><td>18</td><td>miRNA: 3' ugGGUCCAUCGUUCCUAa 5'
:| || ||||:||||
Target:5' acTCTGGAAGCAGGGATt 3'</td><td>-19.90</td><td>144.00</td><td>Show result profile</td></tr>
</table>

<table>
<tr><td>265</td><td>hsa-miR-502-5p</td><td>1720~1740</td><td>21</td><td>miRNA: 3' auCGUGGGUCUAUCGUUCCUa 5'
| ||:| |: ||||:|||
Target:5' gtGAACTCTGGAAGCAGGGAt 3'</td><td>-18.10</td><td>147.00</td><td>Show result profile</td></tr>
<tr><td>266</td><td>hsa-miR-503</td><td>225~251</td><td>27</td><td>miRNA: 3' gaCGUCUUGA----CAAGGGCGACGau 5'
| ||||:|
|||||:|||||
Target:5' caGAAGAATTTAAAGTTCCTGCTGCaa 3'</td><td>-20.40</td><td>143.00</td><td>Show result profile</td></tr>
<tr><td>267</td><td>hsa-miR-507</td><td>242~262</td><td>21</td><td>miRNA: 3' aaGUGAGGUUUUCCACGUUuu 5'
::|| ||| | ||||||
Target:5' ccTGCTGCAACAAGTGCAAtt 3'</td><td>-9.50</td><td>143.00</td><td>Show result profile</td></tr>
<tr><td rowspan="2">268</td><td rowspan="2">hsa-miR-512-5p</td><td>1054~1074</td><td>21</td><td>miRNA: 3' cuUUCACGGGAGUUCCGACUCAc 5'
||| :|||: |||||||
Target:5' agAAGCATCCTT--TGCTGAGTt 3'</td><td>-15.00</td><td>153.00</td><td rowspan="2">Show result profile</td></tr>
<tr><td>1054~1074</td><td>21</td><td>miRNA: 3' cuUUCACGGGAGUUCCGACUCAc 5'
||| :|||: |||||||
Target:5' agAAGCATCCTT--TGCTGAGTt 3'</td><td>-15.00</td><td>153.00</td></tr>
<tr><td rowspan="6">269</td><td rowspan="6">hsa-miR-513a-3p</td><td>2254~2277</td><td>24</td><td>miRNA: 3' ggaAGAGUC-UUUCCACUUUAAAu 5'
|||:|| ||| |:|||||
Target:5' gagTCTTAGTTAAGCAGGAATTTa 3'</td><td>-11.10</td><td>148.00</td><td rowspan="6">Show result profile</td></tr>
<tr><td>1026~1049</td><td>24</td><td>miRNA: 3' ggAAGAGUCUUUCCA-CUUUAAAu 5'
| ||: || ||| |:|||||
Target:5' agTACTTTTAATGGTGGGAATTTa 3'</td><td>-10.50</td><td>145.00</td></tr>
<tr><td>2170~2198</td><td>29</td><td>miRNA: 3' ggAAGA--GUCU---UUCC-ACUUUAAAu 5'
||:| |||| |:||
|||:|||
Target:5' atTTTTAGCAGAGACAGGGAGGAAGTTTt 3'</td><td>-13.70</td><td>143.00</td></tr>
<tr><td>2254~2277</td><td>24</td><td>miRNA: 3' ggaAGAGUC-UUUCCACUUUAAAu 5'
|||:|| ||| |:|||||
Target:5' gagTCTTAGTTAAGCAGGAATTTa 3'</td><td>-11.10</td><td>148.00</td></tr>
<tr><td>1026~1049</td><td>24</td><td>miRNA: 3' ggAAGAGUCUUUCCA-CUUUAAAu 5'
| ||: || ||| |:|||||
Target:5' agTACTTTTAATGGTGGGAATTTa 3'</td><td>-10.50</td><td>145.00</td></tr>
<tr><td></td><td></td><td>miRNA: 3' ggAAGA--GUCU---UUCC-ACUUUAAAu 5'</td><td></td><td></td></tr>
</table>

		2170~2198	29	||:| |||| |:|| |||:||| Target:5' atTTTTAGCAGAGACAGGGAGGAAGTTTt 3'	-13.70	143.00	
270	hsa-miR-515-5p	1446~1466	21	miRNA: 3' guCUUUCACGAAAGAAAACCUCUu 5' |||:||| | | ||||:|| Target:5' caGAAGGTG---TATGTTGGGGAg 3'	-19.70	147.00	Show result profile
271	hsa-miR-515-3p	428~455	28	miRNA: 3' uuGCGAGGUUUU--CU----UCCGUGAg 5' :|:| :|||| || |||||:| Target:5' gtTGTTGTAAAATTGAAATCAGGCATTt 3'	-11.30	140.00	Show result profile
272	hsa-miR-515-5p	1446~1466	21	miRNA: 3' guCUUUCACGAAAGAAAACCUCUu 5' |||:||| | | ||||:|| Target:5' caGAAGGTG---TATGTTGGGGAg 3'	-19.70	147.00	Show result profile
273	hsa-miR-515-3p	428~455	28	miRNA: 3' uuGCGAGGUUUU--CU----UCCGUGAg 5' :|:| :|||| || |||||:| Target:5' gtTGTTGTAAAATTGAAATCAGGCATTt 3'	-11.30	140.00	Show result profile
274	hsa-miR-517*	1737~1762	26	miRNA: 3' ucUGUCAC-GA---AGGUAGAUCUCc 5' |: ||| || ||: |:||||| Target:5' ggATTGTGTCTGGCTCTTTTTAGAGc 3'	-12.00	140.00	Show result profile
		1737~1762	26	miRNA: 3' ucUGUCAC-GA---AGGUAGAUCUCc 5' |: ||| || ||: |:||||| Target:5' ggATTGTGTCTGGCTCTTTTTAGAGc 3'	-12.00	140.00	
275	hsa-miR-517b	2127~2148	22	miRNA: 3' uugugaGAUUUCCCUACGUGcu 5' ||| ||| |||||| Target:5' ctgggaCTACAGGCATGCACca 3'	-12.60	144.00	Show result profile
276	hsa-miR-517*	1737~1762	26	miRNA: 3' ucUGUCAC-GA---AGGUAGAUCUCc 5' |: ||| || ||: |:||||| Target:5' ggATTGTGTCTGGCTCTTTTTAGAGc 3'	-12.00	140.00	Show result profile
277	hsa-miR-519e	434~455	22	miRNA: 3' uugugaGAUUUUCCUCCGUGAa 5' :|:||| |||||:| Target:5' gtaaaaTTGAAATCAGGCATTt 3'	-11.20	140.00	Show result profile

<table>
<tr><td>278</td><td>hsa-miR-520a-5p</td><td>1712~1732</td><td>21</td><td>miRNA: 3' ucuUUCAUGAAGGGAGACCUc 5'
:| |: | |||||||
Target:5' tctGAATGGTGAACTCTGGAa 3'</td><td>-12.30</td><td>142.00</td><td>Show result profile</td></tr>
<tr><td>279</td><td>hsa-miR-520b</td><td>1013~1033</td><td>21</td><td>miRNA: 3' ggGAGAUUUUCCUUCGUGAAa 5'
|| ||:|| ||:||||
Target:5' acCTATAGAATCCAGTACTTt 3'</td><td>-12.00</td><td>143.00</td><td>Show result profile</td></tr>
<tr><td>280</td><td>hsa-miR-520c-3p</td><td>1012~1033</td><td>22</td><td>miRNA: 3' ugGGAGAUUUUCCUUCGUGAAa
5'
||| ||:|| ||:||||
Target:5' gaCCTATAGAATCCAGTACTTt
3'</td><td>-12.00</td><td>148.00</td><td>Show result profile</td></tr>
<tr><td>281</td><td>hsa-miR-522</td><td>1903~1924</td><td>22</td><td>miRNA: 3' ugugAGAUUUCCCUUGGUAAaa
5'
|:|:||| | ||||||
Target:5' agcaTTTGAAGTGTACCATTgg
3'</td><td>-10.50</td><td>146.00</td><td>Show result profile</td></tr>
<tr><td>282</td><td>hsa-miR-525-5p</td><td>1715~1732</td><td>18</td><td>miRNA: 3' ucUUUCACGUAGGGAGACCUc 5'
| :||| | |||||||
Target:5' gaATGGTGAA---CTCTGGAa 3'</td><td>-13.20</td><td>148.00</td><td>Show result profile</td></tr>
<tr><td>283</td><td>hsa-miR-526b</td><td>118~138</td><td>21</td><td>miRNA: 3' uguCUUUCACGAAGGGAGUUCUc
5'
|||:|| ||||:|:||||
Target:5' gtcGAAGGT--TTCCTTTAAGAt
3'</td><td>-16.30</td><td>144.00</td><td>Show result profile</td></tr>
<tr><td>284</td><td>hsa-miR-526b*</td><td>1013~1034</td><td>22</td><td>miRNA: 3' cgGAGAUUUUCCUUCGUGAAAg
5'
|| ||:|| ||:|||||
Target:5' acCTATAGAATCCAGTACTTTt
3'</td><td>-13.00</td><td>148.00</td><td>Show result profile</td></tr>
<tr><td>285</td><td>hsa-miR-539</td><td>1139~1162</td><td>24</td><td>miRNA: 3' uguguGGUUCCUAU--UAAAGAgg
5'
:::|||||| ||||||
Target:5' gtgttTTGAGGATAGTATTTCTaa
3'</td><td>-11.70</td><td>143.00</td><td>Show result profile</td></tr>
<tr><td>286</td><td>hsa-miR-541*</td><td>1038~1066</td><td>29</td><td>miRNA: 3' ucACCC-UGGCUGUCG---
UCUUAGGAAa 5'
|||| |:: ||||: ||
||||||
Target:5'
ggTGGGAATTTACAGTAGAAGCATCCTTt 3'</td><td>-26.40</td><td>143.00</td><td>Show result profile</td></tr>
<tr><td>287</td><td>hsa-miR-542-3p</td><td>558~577</td><td>20</td><td>miRNA: 3' aaagUCAAUAGUUAGACAGUGu
5'
:| || :| |||||||
Target:5' ttaaGGCTACTA--CTGTCACa
3'</td><td>-12.00</td><td>146.00</td><td>Show result profile</td></tr>
<tr><td></td><td></td><td>1426~1451</td><td>26</td><td>miRNA: 3' cuUGAACGAU---UUUU-
ACGUCUUa 5'
||| | || ||||
|||||||
Target:5'</td><td>-10.20</td><td>164.00</td><td></td></tr>
</table>

<table>
<tr><td>288</td><td>hsa-miR-544</td><td></td><td></td><td>ggACTGGATAGACAAAACTGCAGAAg 3'</td><td></td><td></td><td>Show result profile</td></tr>
<tr><td></td><td></td><td>2497~2515</td><td>19</td><td>miRNA: 3' cuUGAACGAUUUUUACGUCUUa 5'
|:|| :| :|||||||
Target:5' taATTTCTT---TGTGCAGAAt 3'</td><td>-10.80</td><td>145.00</td><td></td></tr>
<tr><td>289</td><td>hsa-miR-544b</td><td>897~917</td><td>21</td><td>miRNA: 3' aaucUUUACGUGUUGGAGUCca 5'
|:|| || :|||||||
Target:5' tttaAGATTCA-GACCTCAGtt 3'</td><td>-11.50</td><td>141.00</td><td>Show result profile</td></tr>
<tr><td>290</td><td>hsa-miR-545*</td><td>688~713</td><td>26</td><td>miRNA: 3' agUAGAU----UAUUUGUAAAUGACu 5'
||:|: || ||
||||||||
Target:5' caATTTGGAACATTAAAATTTACTGa 3'</td><td>-8.60</td><td>162.00</td><td>Show result profile</td></tr>
<tr><td></td><td></td><td>1236~1260</td><td>25</td><td>miRNA: 3' aguaGAUUAUU---UGUAAAUGACu 5'
||:||:| |:|||||:||
Target:5' agtgCTGATGAGTTATATTTATTGa 3'</td><td>-13.70</td><td>150.00</td><td></td></tr>
<tr><td></td><td></td><td>1990~2011</td><td>22</td><td>miRNA: 3' aguagaUUAUUUGUAAAUGACu 5'
|| ||::||||:|||
Target:5' caaaggAAAAAGTATTTGCTGa 3'</td><td>-11.40</td><td>148.00</td><td></td></tr>
<tr><td></td><td></td><td>1043~1071</td><td>29</td><td>miRNA: 3' agUAGAU----UAUUUGU---AAAUGACu 5'
||:|| | ||:||
|||:|||
Target:5' gaATTTACAGTAGAAGCATCCTTTGCTGa 3'</td><td>-9.50</td><td>142.00</td><td></td></tr>
<tr><td>291</td><td>hsa-miR-545</td><td>1990~2011</td><td>22</td><td>miRNA: 3' cgugugUUAUUUACAAACGACu 5'
|| ||:| |||||||
Target:5' caaaggAAAAAGTATTTGCTGa 3'</td><td>-10.00</td><td>160.00</td><td>Show result profile</td></tr>
<tr><td></td><td></td><td>1045~1071</td><td>27</td><td>miRNA: 3' cguGUGUUAUUUAC-----AAACGACu 5'
:|||:||:| |
|||||||
Target:5' attTACAGTAGAAGCATCCTTTGCTGa 3'</td><td>-13.10</td><td>159.00</td><td></td></tr>
<tr><td></td><td></td><td>402~432</td><td>31</td><td>miRNA: 3' cguGUGUUAUUUA----C-----AAACGACu 5'
|| ||||||| |
||||:||
Target:5' tttCAGAATAAATATTGGTATTTTTTGTTGt 3'</td><td>-7.70</td><td>141.00</td><td></td></tr>
<tr><td></td><td></td><td>2518~2541</td><td>24</td><td>miRNA: 3' cguUUUCAUUAAC---GGUCAAAac 5'
:|||| |||| :|||||</td><td>-8.60</td><td>142.00</td><td></td></tr>
</table>

<table>
<tr><td rowspan="3">292</td><td rowspan="3">hsa-miR-548a-3p</td><td></td><td></td><td>Target:5' tttGAAGT-
ATTGTATTCAGTTTac 3'</td><td></td><td></td><td rowspan="3">Show result profile</td></tr>
<tr><td>2518~2541</td><td>24</td><td>miRNA: 3' cguUUUCAUUAAC---
GGUCAAAac 5'
:|||| |||| :||||||
Target:5' tttGAAGT-
ATTGTATTCAGTTTac 3'</td><td>-8.60</td><td>142.00</td></tr>
<tr><td>2518~2541</td><td>24</td><td>miRNA: 3' cguUUUCAUUAAC---
GGUCAAAac 5'
:|||| |||| :||||||
Target:5' tttGAAGT-
ATTGTATTCAGTTTac 3'</td><td>-8.60</td><td>142.00</td></tr>
<tr><td>293</td><td>hsa-miR-548e</td><td>2517~2541</td><td>25</td><td>miRNA: 3' acguUUUCAU--CA-
GAGUCAAAaa 5'
:||||| || :|||||||
Target:5'
atttGAAGTATTGTATTCAGTTTac 3'</td><td>-8.60</td><td>144.00</td><td>Show result profile</td></tr>
<tr><td rowspan="4">294</td><td rowspan="4">hsa-miR-548i</td><td>2486~2507</td><td>22</td><td>miRNA: 3' ccguuUUAGGCGUUAAUGAAAa
5'
:|||:|:|||| ||||
Target:5' aatgtGATCTGTAATTTCTTTg
3'</td><td>-11.20</td><td>141.00</td><td rowspan="4">Show result profile</td></tr>
<tr><td>2486~2507</td><td>22</td><td>miRNA: 3' ccguuUUAGGCGUUAAUGAAAa
5'
:|||:|:|||| ||||
Target:5' aatgtGATCTGTAATTTCTTTg
3'</td><td>-11.20</td><td>141.00</td></tr>
<tr><td>2486~2507</td><td>22</td><td>miRNA: 3' ccguuUUAGGCGUUAAUGAAAa
5'
:|||:|:|||| ||||
Target:5' aatgtGATCTGTAATTTCTTTg
3'</td><td>-11.20</td><td>141.00</td></tr>
<tr><td>2486~2507</td><td>22</td><td>miRNA: 3' ccguuUUAGGCGUUAAUGAAAa
5'
:|||:|:|||| ||||
Target:5' aatgtGATCTGTAATTTCTTTg
3'</td><td>-11.20</td><td>141.00</td></tr>
<tr><td>295</td><td>hsa-miR-548k</td><td>1016~1034</td><td>19</td><td>miRNA: 3' ucGUUUUAGGCGUUCAUGAAAa
5'
:|:||||| ||||||||
Target:5' taTAGAATCC---AGTACTTTt
3'</td><td>-14.80</td><td>165.00</td><td>Show result profile</td></tr>
<tr><td rowspan="2">296</td><td rowspan="2">hsa-miR-548l</td><td>1016~1034</td><td>19</td><td>miRNA: 3' cuGUUUUGGGCGUUUAUGAAAa
5'
:|:||:|| |:||||||
Target:5' taTAGAATCC---AGTACTTTt
3'</td><td>-13.00</td><td>145.00</td><td rowspan="2">Show result profile</td></tr>
<tr><td>1475~1493</td><td>19</td><td>miRNA: 3' cugUUUUGGGCGUUUAUGAAaa
5'
||||| ||||||||
Target:5' gggAAAAC---AAAATACTTga
3'</td><td>-8.80</td><td>140.00</td></tr>
</table>

<table>
<tr><td rowspan="2">297</td><td rowspan="2">hsa-miR-548m</td><td>384~405</td><td>22</td><td>miRNA: 3' guUUUUG-GUGUUUAUGGAAAc 5'
::||| :||:| |:|||||
Target:5' ttGGAACATACGATTGCCTTTc 3'</td><td>-11.00</td><td>147.00</td><td rowspan="2">Show result profile</td></tr>
<tr><td>1017~1035</td><td>19</td><td>miRNA: 3' guUUUUGGUGUUUAUGGAAAc 5'
|:||:| | |:|||:|||
Target:5' atAGAATC-C-AGTACTTTTa 3'</td><td>-7.90</td><td>141.00</td></tr>
<tr><td>298</td><td>hsa-miR-548n</td><td>1017~1035</td><td>19</td><td>miRNA: 3' ugUUUUAGGUGUUAAUGAAAAc 5'
|:||||| | |||||||
Target:5' atAGAATCC---AGTACTTTTa 3'</td><td>-12.00</td><td>161.00</td><td>Show result profile</td></tr>
<tr><td rowspan="2">299</td><td rowspan="2">hsa-miR-548p</td><td>2285~2305</td><td>21</td><td>miRNA: 3' uuUCAUUGACGUCAAAAACGAu 5'
| | ||| | ||||||||
Target:5' ccATTTCCTG-ACTTTTTGCTt 3'</td><td>-8.80</td><td>155.00</td><td rowspan="2">Show result profile</td></tr>
<tr><td>410~431</td><td>22</td><td>miRNA: 3' uuUCAUUGACGUCAAAAACGAu 5'
| || : |:| ||||||:|
Target:5' taAATATTGGTATTTTTTGTTg 3'</td><td>-7.20</td><td>144.00</td></tr>
<tr><td rowspan="3">300</td><td rowspan="3">hsa-miR-548u</td><td>574~594</td><td>21</td><td>miRNA: 3' gcGUUUUCAUUAACGUCAGAAAc 5'
||:||| || :|||||||
Target:5' caCAGAAG--ATCATAGTCTTTg 3'</td><td>-15.30</td><td>161.00</td><td rowspan="3">Show result profile</td></tr>
<tr><td>1480~1504</td><td>25</td><td>miRNA: 3' gcGUUUUCAUUAAC---GUCAGAAac 5'
||||| || ||| :||||||
Target:5' aaCAAAA-TACTTGACATAGTCTTaa 3'</td><td>-8.20</td><td>148.00</td></tr>
<tr><td>185~215</td><td>31</td><td>miRNA: 3' gcGUUUUCAU-U---AA----CGUCAGAAac 5'
::|||||| | ||
||||||||
Target:5' ccTGAAAGTACACCTTTCACAGCAGTCTTaa 3'</td><td>-13.40</td><td>143.00</td></tr>
<tr><td>301</td><td>hsa-miR-548x</td><td>2181~2200</td><td>20</td><td>miRNA: 3' acUUUCAUUAACGUCAAAAAu 5'
| || :| | |||||||
Target:5' agACAGGGA-GGAAGTTTTTa 3'</td><td>-7.80</td><td>146.00</td><td>Show result profile</td></tr>
<tr><td>302</td><td>hsa-miR-548y</td><td>2176~2205</td><td>30</td><td>miRNA: 3' ccGUUUUUGUCACU--------AAUGAAAa 5'
||:|:|||| ||
|||:|||
Target:5' agCAGAGACAGGGAGGAAGTTTTTATTTTt 3'</td><td>-13.80</td><td>146.00</td><td>Show result profile</td></tr>
<tr><td></td><td></td><td></td><td></td><td>miRNA: 3' ucuCGA--GUAG-GUAUCAACAGu</td><td></td><td></td><td></td></tr>
</table>

<table>
<tr><td rowspan="2">303</td><td rowspan="2">hsa-miR-549</td><td>64~87</td><td>24</td><td>5'
||| | | |: |||||||
Target:5' cgtGCTACCCCCGCGGAGTTGTCg 3'</td><td>-15.40</td><td>144.00</td><td rowspan="2">Show result profile</td></tr>
<tr><td>1913~1933</td><td>21</td><td>miRNA: 3' ucucgaGUAGGUAUCAACAGu 5'
|||: :|||||||:
Target:5' gtgtacCATTGGTAGTTGTTt 3'</td><td>-13.10</td><td>143.00</td></tr>
<tr><td rowspan="2">304</td><td rowspan="2">hsa-miR-551b*</td><td>792~814</td><td>23</td><td>miRNA: 3' ccagagUGGGUG-CGAACUAAAg 5'
||:||: :|||||||
Target:5' ttgcagACTCATAATTTGATTTt 3'</td><td>-10.20</td><td>152.00</td><td rowspan="2">Show result profile</td></tr>
<tr><td>2534~2557</td><td>24</td><td>miRNA: 3' ccaGAGUGGGUGCG--AACUAAAg 5'
:|:|| ::||: |||:|||
Target:5' cagTTTACATGCGTTATTGGTTTa 3'</td><td>-11.90</td><td>141.00</td></tr>
<tr><td>305</td><td>hsa-miR-561</td><td>198~221</td><td>24</td><td>miRNA: 3' ugAAGU-UCCU-AGAAUUUGAAAc 5'
|||| || | ||||||| |||
Target:5' ctTTCACAGCAGTCTTAAAGTTTg 3'</td><td>-12.20</td><td>144.00</td><td>Show result profile</td></tr>
<tr><td>306</td><td>hsa-miR-562</td><td>362~386</td><td>25</td><td>miRNA: 3' cgUUUACCA--UGU---CGAUGAAa 5'
||:| || |:| |||||||
Target:5' ggAAGTTGTTAATATCTGCTACTTg 3'</td><td>-7.10</td><td>152.00</td><td>Show result profile</td></tr>
<tr><td rowspan="3">307</td><td rowspan="3">hsa-miR-567</td><td>374~395</td><td>22</td><td>miRNA: 3' caAGAC-AGGACCUUCUUGUAUGa 5'
|||| | || | ||||||||
Target:5' taTCTGCTACTTG--GAACATACg 3'</td><td>-14.40</td><td>161.00</td><td rowspan="3">Show result profile</td></tr>
<tr><td>1537~1558</td><td>22</td><td>miRNA: 3' caaGACAGGACCUUCUUGUAUGa 5'
||| ||| | |||||||
Target:5' ctaCTGGCCTTG-TCAACATACa 3'</td><td>-12.80</td><td>155.00</td></tr>
<tr><td>439~463</td><td>25</td><td>miRNA: 3' caagacaGGACCUU--CUUGUAUGa 5'
:| || | |||||||
Target:5' attgaaaTCAGGCATTTAACATACt 3'</td><td>-8.00</td><td>142.00</td></tr>
<tr><td>308</td><td>hsa-miR-577</td><td>1600~1620</td><td>21</td><td>miRNA: 3' guccauggUUAUAAAAUAGAu 5'
|| |||||||:|
Target:5' tgtaagggAAGATTTTATTTg 3'</td><td>-7.70</td><td>141.00</td><td>Show result profile</td></tr>
<tr><td rowspan="2">309</td><td rowspan="2">hsa-miR-</td><td>1948~1970</td><td>23</td><td>miRNA: 3' uuagcgccaaAUAUGGUUUACUu 5'
|:|: |||||||
Target:5' tattcataaaTGTGACAAATGAa 3'</td><td>-8.50</td><td>149.00</td><td rowspan="2">Show result profile</td></tr>
<tr><td></td><td></td><td></td><td></td><td></td></tr>
</table>

<table>
<tr><td></td><td>579</td><td>618~640</td><td>23</td><td>miRNA: 3' uuagcgcCAAAUAUGGUUUACUu 5'
||| : : |||||||
Target:5' gtagttcGTTGGGGGCAAATGAa 3'</td><td>-12.50</td><td>148.00</td><td></td></tr>
<tr><td>310</td><td>hsa-miR-586</td><td>530~553</td><td>24</td><td>miRNA: 3' ccuGGAU--UUUUAUGUUACGUAu 5'
|||| ||:| : |||||:|
Target:5' cttCCTATGAAGAGGGAATGCGTa 3'</td><td>-12.50</td><td>145.00</td><td>Show result profile</td></tr>
<tr><td rowspan="2">311</td><td rowspan="2">hsa-miR-589*</td><td>74~97</td><td>24</td><td>miRNA: 3' agacCCUUGGCCGUAAACAAGACu 5'
|||:::| |:| |||||||
Target:5' ccgcGGAGTTGTCGTGTGTTCTGg 3'</td><td>-21.30</td><td>168.00</td><td rowspan="2">Show result profile</td></tr>
<tr><td>2041~2064</td><td>24</td><td>miRNA: 3' agacccuuggccGUAAACAAGACu 5'
:|||||||:||
Target:5' tttatttttattTATTTGTTTTGt 3'</td><td>-9.30</td><td>140.00</td></tr>
<tr><td>312</td><td>hsa-miR-590-3p</td><td>1923~1942</td><td>20</td><td>miRNA: 3' ugAUCGAAUAUGUAUUUUAAu 5'
||| ||:| :||:|||||
Target:5' ggTAG-TTGTTTATGAAATTa 3'</td><td>-9.10</td><td>150.00</td><td>Show result profile</td></tr>
<tr><td>313</td><td>hsa-miR-592</td><td>1085~1106</td><td>22</td><td>miRNA: 3' ugUAG-UAGCGUAUAACUGUGUu 5'
||| ||| | | ||||:|||
Target:5' ttATCAATCTC-TTTTGATACAa 3'</td><td>-12.40</td><td>147.00</td><td>Show result profile</td></tr>
<tr><td>314</td><td>hsa-miR-593</td><td>2167~2185</td><td>19</td><td>miRNA: 3' ucuUUGGGGUCGUCUCUGu 5'
| ::::|||||||||
Target:5' tgtATTTTTAGCAGAGACa 3'</td><td>-17.80</td><td>156.00</td><td>Show result profile</td></tr>
<tr><td>315</td><td>hsa-miR-609</td><td>2268~2287</td><td>20</td><td>miRNA: 3' ucucuacucUCUUUGUGGGa 5'
| :|||||||
Target:5' caggaatttATGAACACCCa 3'</td><td>-9.40</td><td>143.00</td><td>Show result profile</td></tr>
<tr><td>316</td><td>hsa-miR-613</td><td>1064~1084</td><td>21</td><td>miRNA: 3' ccguuuCUUCCU-UGUAAGGa 5'
||: | |||||||
Target:5' tttgctGAGTTATACATTCCt 3'</td><td>-11.60</td><td>142.00</td><td>Show result profile</td></tr>
<tr><td rowspan="2">317</td><td rowspan="2">hsa-miR-616*</td><td>1130~1149</td><td>20</td><td>miRNA: 3' uucaGUGACUUCCCAAAACUCa 5'
||||| | |||||||||
Target:5' aaacCACTG--GTGTTTTGAGg 3'</td><td>-15.80</td><td>162.00</td><td rowspan="2">Show result profile</td></tr>
<tr><td>649~671</td><td>23</td><td>miRNA: 3' uuCAG-UGACUUCCCAAAACUca 5'
||: ||||:| | |||||||
Target:5' ctGTTAACTGGAAGCTTTTGAta 3'</td><td>-8.60</td><td>148.00</td></tr>
</table>

<table>
<tr><td>318</td><td>hsa-miR-616</td><td>253~275</td><td>23</td><td>miRNA: 3' gaCGAGUUUGGGA-GGUUACUGa 5'
|: ||| : :| |||||||:
Target:5' aaGTGCAATTATTACCAATGATg 3'</td><td>-9.90</td><td>140.00</td><td>Show result profile</td></tr>
<tr><td rowspan="3">319</td><td rowspan="3">hsa-miR-617</td><td>47~65</td><td>19</td><td>miRNA: 3' cgGUG-GAAGUUUACCCUUCAGa 5'
||| || |:| |||||||
Target:5' agCACACT--AGA--GGAAGTCg 3'</td><td>-11.90</td><td>148.00</td><td rowspan="3">Show result profile</td></tr>
<tr><td>1219~1240</td><td>22</td><td>miRNA: 3' cgguggAAGUUUACCCUUCAga 5'
| ||:|| ||||||
Target:5' aggaaaTACAGATCGGAAGTgc 3'</td><td>-11.00</td><td>140.00</td></tr>
<tr><td>1264~1283</td><td>20</td><td>miRNA: 3' cgGUGGAAGUUUACCCUUCAga 5'
|| |||: || ||||||
Target:5' ccCAACTTTTAA--GGAAGTgc 3'</td><td>-12.60</td><td>140.00</td></tr>
<tr><td>320</td><td>hsa-miR-622</td><td>283~303</td><td>21</td><td>miRNA: 3' cgaggUUGGA-GUCGUCUGACa 5'
| ||| || |||||||
Target:5' aataaATCCTGCA-CAGACTGc 3'</td><td>-14.20</td><td>151.00</td><td>Show result profile</td></tr>
<tr><td>321</td><td>hsa-miR-624*</td><td>404~424</td><td>21</td><td>miRNA: 3' acUUGUGUUCCAUGACCAUGAu 5'
|: |:|| ||:|||||:|
Target:5' tcAGAATAA-ATATTGGTATTt 3'</td><td>-12.10</td><td>143.00</td><td>Show result profile</td></tr>
<tr><td rowspan="3">322</td><td rowspan="3">hsa-miR-627</td><td>888~908</td><td>21</td><td>miRNA: 3' aggaGAAAAGAAUCUCUGAGUg 5'
|||||:||| |||:|||
Target:5' tttaCTTTTTTTA-AGATTCAg 3'</td><td>-13.40</td><td>157.00</td><td rowspan="3">Show result profile</td></tr>
<tr><td>783~803</td><td>21</td><td>miRNA: 3' aggAGAAAAGAAUCUCUGAGUg 5'
||| | ||: |||||||
Target:5' gcaTCTGAT-TTGCAGACTCAt 3'</td><td>-13.30</td><td>154.00</td></tr>
<tr><td>2066~2089</td><td>24</td><td>miRNA: 3' agGAGAAAAGAAUC--UCUGAGug 5'
:|:||||:| || ||||||
Target:5' ttTTTTTTTTTGAGGCAGACTCtc 3'</td><td>-12.70</td><td>150.00</td></tr>
<tr><td rowspan="2">323</td><td rowspan="2">hsa-miR-629*</td><td>1447~1469</td><td>23</td><td>miRNA: 3' cgacCCGAAUGCAA-CCCUCUUg 5'
||: ||:||| |||||||
Target:5' agaaGGTGTATGTTGGGGAGAAc 3'</td><td>-22.10</td><td>166.00</td><td rowspan="2">Show result profile</td></tr>
<tr><td></td><td></td><td></td><td></td><td></td></tr>
</table>

<table>
<tr><td></td><td></td><td>1852~1877</td><td>26</td><td>miRNA: 3' cgaCCCGAAU----
GCAACCCUCUUg 5'
|| | || |
|||||:|||
Target:5'
ataGGCCCTAAGTTCATTGGGGGAAa 3'</td><td>-13.20</td><td>141.00</td><td></td></tr>
<tr><td>324</td><td>hsa-miR-637</td><td>26~49</td><td>24</td><td>miRNA: 3' ugCGUCUCGGGCUUUCGGGGGUCa
5'
|::|||| |: :|:||||||
Target:5' gaGTGGAGCGGGGCGGTCCCCAGc
3'</td><td>-24.80</td><td>154.00</td><td>Show result profile</td></tr>
<tr><td rowspan="2">325</td><td rowspan="2">hsa-miR-642a</td><td>1~15</td><td>15</td><td>miRNA: 3' guucuguguaAACCUCUCCCUg
5'
||| |||||||
Target:5' -------agaTTGCAGAGGGAg
3'</td><td>-12.40</td><td>152.00</td><td rowspan="2">Show result profile</td></tr>
<tr><td>526~547</td><td>22</td><td>miRNA: 3' guucuguGUAAACCUCUCCCUg
5'
| | || |||||||
Target:5' tactcttCCTATGAAGAGGGAa
3'</td><td>-12.20</td><td>151.00</td></tr>
<tr><td>326</td><td>hsa-miR-648</td><td>37~55</td><td>19</td><td>miRNA: 3' ugGUCACGGGACGUGUGAa 5'
|:|| ||| |||||||
Target:5' ggCGGTCCCCAGCACACTa 3'</td><td>-19.80</td><td>165.00</td><td>Show result profile</td></tr>
<tr><td>327</td><td>hsa-miR-649</td><td>809~833</td><td>25</td><td>miRNA: 3' cuGAGAACUUGU---
UGUGUCCAAa 5'
:|:|| | :| |:|:|||||
Target:5'
gaTTTTTAATTAAATATATAGGTTa 3'</td><td>-9.80</td><td>140.00</td><td>Show result profile</td></tr>
<tr><td>328</td><td>hsa-miR-654-3p</td><td>830~853</td><td>24</td><td>miRNA: 3' uuccACUAC--CAGUCGUCUGUAu
5'
||||| || | :||||||
Target:5' gttaTGATGAAGTGAATAGACATa
3'</td><td>-14.10</td><td>148.00</td><td>Show result profile</td></tr>
<tr><td>329</td><td>hsa-miR-655</td><td>1691~1714</td><td>24</td><td>miRNA: 3' uuUCUCCAAUUG--GUACAUAAua
5'
|| | ||:|| | ||||||
Target:5' tcAGTGCTTGACCTCCTGTATTct
3'</td><td>-8.70</td><td>142.00</td><td>Show result profile</td></tr>
<tr><td rowspan="2">330</td><td rowspan="2">hsa-miR-664</td><td>617~642</td><td>26</td><td>miRNA: 3' acAUC---
CGACCCCUAUUUACUUAu 5'
||| |:|||||:
||||||||
Target:5'
ggTAGTTCGTTGGGGGCAAATGAATt 3'</td><td>-24.20</td><td>177.00</td><td rowspan="2">Show result profile</td></tr>
<tr><td>825~847</td><td>23</td><td>miRNA: 3' acAUCCGACCCCUA-UUUACUUAu
5'
||||:| ||| ||:|||||
Target:5' taTAGGTT-ATGATGAAGTGAATa
3'</td><td>-13.40</td><td>148.00</td></tr>
<tr><td></td><td></td><td>109~131</td><td>23</td><td>miRNA: 3' gaGGUCGGGGAGGUCCCGAAGGa
5'
||| | : || | ||:||||</td><td>-19.00</td><td>145.00</td><td></td></tr>
</table>

<table>
<tr><td rowspan="3">331</td><td rowspan="3">hsa-
miR-
671-5p</td><td></td><td></td><td>Target:5' caCCACCATGTCGAAGGTTTCCt
3'</td><td></td><td></td><td rowspan="3">Show result profile</td></tr>
<tr><td>1330~1355</td><td>26</td><td>miRNA: 3' gaggUCGGG----
GAGGUCCCGAAGGa 5'
||||| | |||
||||||:
Target:5' gggaAGCCCAGATCACCA-
GGCTTCTa 3'</td><td>-19.70</td><td>144.00</td></tr>
<tr><td>2078~2105</td><td>28</td><td>miRNA: 3' gagGUCGGGGAGGU--C---
CCGAAGGa 5'
||| |:|||:: |
||||||:
Target:5'
aggCAGACTCTCTGTCGCCAGGCTTCTc 3'</td><td>-19.10</td><td>142.00</td></tr>
<tr><td rowspan="3">332</td><td rowspan="3">hsa-
miR-7</td><td>165~187</td><td>23</td><td>miRNA: 3' uguUGUUUUAGUGA-UCAGAAGGu
5'
|||||:| ||| ||| ||||
Target:5' cgtACAAAGT-ACTCAGTGTTCCt
3'</td><td>-14.10</td><td>143.00</td><td rowspan="3">Show result profile</td></tr>
<tr><td>165~187</td><td>23</td><td>miRNA: 3' uguUGUUUUAGUGA-UCAGAAGGu
5'
|||||:| ||| ||| ||||
Target:5' cgtACAAAGT-ACTCAGTGTTCCt
3'</td><td>-14.10</td><td>143.00</td></tr>
<tr><td>165~187</td><td>23</td><td>miRNA: 3' uguUGUUUUAGUGA-UCAGAAGGu
5'
|||||:| ||| ||| ||||
Target:5' cgtACAAAGT-ACTCAGTGTTCCt
3'</td><td>-14.10</td><td>143.00</td></tr>
<tr><td rowspan="2">333</td><td rowspan="2">hsa-
miR-
708</td><td>2422~2444</td><td>23</td><td>miRNA: 3' gggucgaUCUAACAUUCGAGGAa
5'
| ||||||||:||||
Target:5' gttttgaACATTGTAAGTTCCTa
3'</td><td>-15.40</td><td>156.00</td><td rowspan="2">Show result profile</td></tr>
<tr><td>220~245</td><td>26</td><td>miRNA: 3' ggGUCG--AUC-
UAACAUUCGAGGAa 5'
|||| || |||
|||:||||
Target:5'
tgCAGCAGAAGAATTTAAAGTTCCTg 3'</td><td>-13.20</td><td>147.00</td></tr>
<tr><td rowspan="2">334</td><td rowspan="2">hsa-
miR-
761</td><td>225~249</td><td>25</td><td>miRNA: 3' acacagUCAAAGU---
GGGACGACg 5'
| ||| | :|||||||
Target:5'
cagaagAATTTAAAGTTCCTGCTGc 3'</td><td>-15.30</td><td>148.00</td><td rowspan="2">Show result profile</td></tr>
<tr><td>2337~2356</td><td>20</td><td>miRNA: 3' acacagUCAAAGUGGGACGACg
5'
| ||| |||||||
Target:5' cacaaaAATTT--GCCTGCTGt
3'</td><td>-12.80</td><td>144.00</td></tr>
<tr><td>335</td><td>hsa-
miR-</td><td>762~790</td><td>29</td><td>miRNA: 3' ucCUCCUGUU---C--AC--
UCGUGGACg 5'
|| |:||| | ||</td><td>-15.70</td><td>140.00</td><td>Show result profile</td></tr>
</table>

<table>
<tr><td></td><td>764</td><td></td><td></td><td>||||:|||
Target:5'
tgGATGGCAATTTGCTTGATAGCATCTGa 3'</td><td></td><td></td><td></td></tr>
<tr><td>336</td><td>hsa-miR-802</td><td>2460~2481</td><td>22</td><td>miRNA: 3' ugUUCCUACUUAGAAACAAUGAc
5'
||| | || :|||||||:|
Target:5' acAAGTA-GACATTTTGTTATTa
3'</td><td>-9.00</td><td>148.00</td><td>Show result profile</td></tr>
<tr><td rowspan="3">337</td><td rowspan="3">hsa-miR-873</td><td>226~246</td><td>21</td><td>miRNA: 3' uccUCUGAGUGUUCAAGGACg 5'
||| |: |||||||||
Target:5' agaAGAATTTAAAGTTCCTGc 3'</td><td>-17.80</td><td>162.00</td><td rowspan="3">Show result profile</td></tr>
<tr><td>169~189</td><td>21</td><td>miRNA: 3' uccUC-UGAGUGUUCAAGGACg
5'
|| ||||| : |||||||
Target:5' caaAGTACTCA-GTGTTCCTGa
3'</td><td>-16.40</td><td>157.00</td></tr>
<tr><td>2425~2445</td><td>21</td><td>miRNA: 3' ucCUCUG-AGUGUUCAAGGAcg
5'
|| || |:::||||||||
Target:5' ttGA-ACATTGTAAGTTCCTaa
3'</td><td>-11.40</td><td>142.00</td></tr>
<tr><td>338</td><td>hsa-miR-876-5p</td><td>1618~1639</td><td>22</td><td>miRNA: 3' accacuAAGUGUUUCUUUAGgu
5'
||| :||||||||
Target:5' ttgcccTTCCGGAAGAAATCag
3'</td><td>-9.00</td><td>140.00</td><td>Show result profile</td></tr>
<tr><td rowspan="2">339</td><td rowspan="2">hsa-miR-877</td><td>1066~1088</td><td>23</td><td>miRNA: 3' ggGACGCGGUA---GAGGAGAUg
5'
||| |::|| :||||:||
Target:5' tgCTGAGTTATACATTCCTTTAt
3'</td><td>-14.30</td><td>142.00</td><td rowspan="2">Show result profile</td></tr>
<tr><td>1831~1850</td><td>20</td><td>miRNA: 3' ggGACGCGGUAGAGGAGAUg 5'
:| | :||||:|||||
Target:5' tgTTTCAGTATCTTCTCTAt 3'</td><td>-13.90</td><td>142.00</td></tr>
<tr><td rowspan="2">340</td><td rowspan="2">hsa-miR-877*</td><td>1381~1403</td><td>23</td><td>miRNA: 3' gaCCCUCCUCC--CUCUUCUCCu
5'
| || |:|| |||||:||
Target:5' caGTGAAGGGGAACAGAAGGGGg
3'</td><td>-17.90</td><td>141.00</td><td rowspan="2">Show result profile</td></tr>
<tr><td>525~545</td><td>21</td><td>miRNA: 3' gacccuccucccuCUUCUCCu 5'
|||||||
Target:5' atactcttcctatGAAGAGGg 3'</td><td>-10.40</td><td>140.00</td></tr>
<tr><td>341</td><td>hsa-miR-892b</td><td>1292~1318</td><td>27</td><td>miRNA: 3' agaUGGGU---CU--
UUCCUCGGUCac 5'
||||| || |||
||||||
Target:5'
gtcACCCATGTGAATAAGAAGCCAGga 3'</td><td>-17.90</td><td>145.00</td><td>Show result profile</td></tr>
</table>

<table>
<tr><td rowspan="10">342</td><td rowspan="10">hsa-miR-9</td><td>2575~2605</td><td>31</td><td>miRNA: 3' agUAUGU----CGAUCUA----
UUGGUUUCu 5'
||||| |:|| ||
|||||||
Target:5'
aaATACATGTTGTTATATTGTAAACCAAAaa 3'</td><td>-8.10</td><td>145.00</td><td rowspan="10">Show result profile</td></tr>
<tr><td>923~948</td><td>26</td><td>miRNA: 3' aguAUGUCGA--UCUA-
UUGGUUUCu 5'
| |||:| | || |||||||
Target:5'
aatTTCAGTTTAATATCAACCAAAaa 3'</td><td>-8.20</td><td>142.00</td></tr>
<tr><td>1511~1534</td><td>24</td><td>miRNA: 3' agUAUGUCGAUCU-AUUGGUUUCu
5'
| :|||:|||| || |||||
Target:5' gaAGGCAGTTAGAGAAAACAAAGt
3'</td><td>-15.50</td><td>141.00</td></tr>
<tr><td>1966~1995</td><td>30</td><td>miRNA: 3' aguaUGUCGAUCUA-----U--
UGGUUUCu 5'
||||| :||| |
|:|||||
Target:5'
atgaACAGCAGGATTATGAATTATCAAAGg 3'</td><td>-14.10</td><td>141.00</td></tr>
<tr><td>2575~2605</td><td>31</td><td>miRNA: 3' agUAUGU----CGAUCUA----
UUGGUUUCu 5'
||||| |:|| ||
|||||||
Target:5'
aaATACATGTTGTTATATTGTAAACCAAAaa 3'</td><td>-8.10</td><td>145.00</td></tr>
<tr><td>923~948</td><td>26</td><td>miRNA: 3' aguAUGUCGA--UCUA-
UUGGUUUCu 5'
| |||:| | || |||||||
Target:5'
aatTTCAGTTTAATATCAACCAAAaa 3'</td><td>-8.20</td><td>142.00</td></tr>
<tr><td>1511~1534</td><td>24</td><td>miRNA: 3' agUAUGUCGAUCU-AUUGGUUUCu
5'
| :|||:|||| || |||||
Target:5' gaAGGCAGTTAGAGAAAACAAAGt
3'</td><td>-15.50</td><td>141.00</td></tr>
<tr><td>1966~1995</td><td>30</td><td>miRNA: 3' aguaUGUCGAUCUA-----U--
UGGUUUCu 5'
||||| :||| |
|:|||||
Target:5'
atgaACAGCAGGATTATGAATTATCAAAGg 3'</td><td>-14.10</td><td>141.00</td></tr>
<tr><td>2575~2605</td><td>31</td><td>miRNA: 3' agUAUGU----CGAUCUA----
UUGGUUUCu 5'
||||| |:|| ||
|||||||
Target:5'
aaATACATGTTGTTATATTGTAAACCAAAaa 3'</td><td>-8.10</td><td>145.00</td></tr>
<tr><td>923~948</td><td>26</td><td>miRNA: 3' aguAUGUCGA--UCUA-
UUGGUUUCu 5'
| |||:| | || |||||||
Target:5'
aatTTCAGTTTAATATCAACCAAAaa 3'</td><td>-8.20</td><td>142.00</td></tr>
</table>

<table>
<tr><td rowspan="2"></td><td rowspan="2"></td><td>1511~1534</td><td>24</td><td><pre>miRNA: 3' agUAUGUCGAUCU-AUUGGUUUCu
5'
            | :|||:|||| || |||||
Target:5' gaAGGCAGTTAGAGAAAACAAAGt
3'</pre></td><td>-15.50</td><td>141.00</td><td rowspan="2"></td></tr>
<tr><td>1966~1995</td><td>30</td><td><pre>miRNA: 3' aguaUGUCGAUCUA-----U--
UGGUUUCu 5'
              ||||| :||| |
|:|||||
Target:5'
atgaACAGCAGGATTATGAATTATCAAAGg 3'</pre></td><td>-14.10</td><td>141.00</td></tr>
<tr><td rowspan="2">343</td><td rowspan="2">hsa-miR-922</td><td>1049~1071</td><td>23</td><td><pre>miRNA: 3' cugCAUCAGGAUAAGAGACGACg
5'
             |||| || ||:|||||
Target:5' acaGTAGAAGCATCCTTTGCTGa
3'</pre></td><td>-13.40</td><td>144.00</td><td rowspan="2">Show result profile</td></tr>
<tr><td>360~383</td><td>24</td><td><pre>miRNA: 3' cugCAUCAG-GAUAAGAGACGAcg
5'
             | |||: :|| | ||||||
Target:5' ttgGAAGTTGTTAATATCTGCTac
3'</pre></td><td>-9.50</td><td>140.00</td></tr>
<tr><td rowspan="2">344</td><td rowspan="2">hsa-miR-92a</td><td>241~262</td><td>22</td><td><pre>miRNA: 3' ugucCGGCCCUGUUCACGUUAu
5'
              ||:| |||||||||||
Target:5' tcctGCTGCAACAAGTGCAATt
3'</pre></td><td>-19.90</td><td>170.00</td><td rowspan="2">Show result profile</td></tr>
<tr><td>241~262</td><td>22</td><td><pre>miRNA: 3' ugucCGGCCCUGUUCACGUUAu
5'
              ||:| |||||||||||
Target:5' tcctGCTGCAACAAGTGCAATt
3'</pre></td><td>-19.90</td><td>170.00</td></tr>
<tr><td>345</td><td>hsa-miR-92b</td><td>241~262</td><td>22</td><td><pre>miRNA: 3' ccucCGGCCCUGCUCACGUUAu
5'
              ||:| || ||||||||
Target:5' tcctGCTGCAACAAGTGCAATt
3'</pre></td><td>-14.40</td><td>162.00</td><td>Show result profile</td></tr>
<tr><td>346</td><td>hsa-miR-93</td><td>1011~1034</td><td>24</td><td><pre>miRNA: 3' gaUGGACGUGCUU--
GUCGUGAAAc 5'
            |||| :| ||| |||:|||||
Target:5' ggACCTATA-
GAATCCAGTACTTTt 3'</pre></td><td>-14.10</td><td>154.00</td><td>Show result profile</td></tr>
<tr><td>347</td><td>hsa-miR-934</td><td>2449~2470</td><td>22</td><td><pre>miRNA: 3' ggucacagaggucaUCAUCUGu
5'
                    |||||||
Target:5' cattttattaaacaAGTAGACa
3'</pre></td><td>-11.20</td><td>140.00</td><td>Show result profile</td></tr>
<tr><td>348</td><td>hsa-miR-936</td><td>1522~1543</td><td>22</td><td><pre>miRNA: 3' gacgcuaaggagggAGAUGACa
5'
                    |||||||
Target:5' gagaaaacaaagtaTCTACTGg
3'</pre></td><td>-7.90</td><td>140.00</td><td>Show result profile</td></tr>
<tr><td></td><td></td><td></td><td></td><td></td><td></td><td></td><td></td></tr>
</table>

<table>
<tr><td>349</td><td>hsa-miR-940</td><td>2092~2112</td><td>21</td><td>miRNA: 3' ccccucgcccccGGGACGGAa 5'
:|||||||
Target:5' tcgccaggcttcTCCTGCCTc 3'</td><td>-14.80</td><td>141.00</td><td>Show result profile</td></tr>
<tr><td rowspan="2">350</td><td rowspan="2">hsa-miR-944</td><td>469~489</td><td>21</td><td>miRNA: 3' gaGUAGGCUACAUGUUAUUAAa 5'
|| | |: || ||||:|||
Target:5' aaCACCAGGAGT-CAATGATTa 3'</td><td>-9.20</td><td>143.00</td><td rowspan="2">Show result profile</td></tr>
<tr><td>2497~2520</td><td>24</td><td>miRNA: 3' gaguAGGCUACAUG--UUAUUAAa 5'
|:| |||:| |||:|||
Target:5' taatTTCTTTGTGCAGAATGATTt 3'</td><td>-7.60</td><td>140.00</td></tr>
<tr><td>351</td><td>hsa-miR-98</td><td>578~605</td><td>28</td><td>miRNA: 3' uugUUA-UGUU-GA----AUGAUGGAGu 5'
:|| |:|: || |:|||||||
Target:5' gaaGATCATAGTCTTTGATGCTACCTCa 3'</td><td>-15.50</td><td>149.00</td><td>Show result profile</td></tr>
</table>

RESULTS

POSITION	SEQUENCE OF TARGET	NAME OF MIRNA	SEQUENCE OF MIRNA	FREE ENERGY	LINK (SEC.STRUCTURE .ps)
1292	TCTTGCATAAATACTGGAAATTGCACATGGTA	hsa-miR-96-3p MIMA	aaucaugugcagugccaauaug	-23.29	image
823	CACTCCTGGACTGTGACTTTCAGTGGGAGATG	hsa-miR-141-5p MIM	caucuuccaguacaguguugga	-23.26	image
170	AGGCAAAACCTTCAACTGAGGACTTGGGGGAT	hsa-miR-141-5p MIM	caucuuccaguacaguguugga	-20.21	image

Results in .CSV format (Right click and 'Save as')

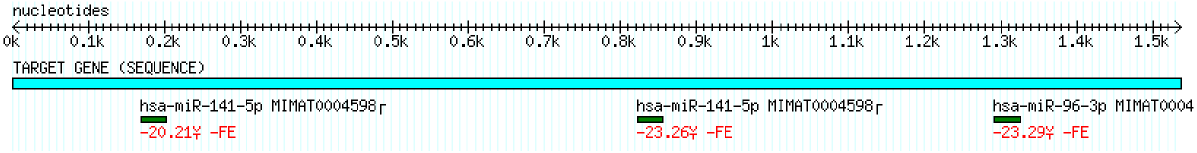

RESULTS

POSITION	SEQUENCE OF TARGET	NAME OF MIRNA	SEQUENCE OF MIRNA	FREE ENERGY	LINK (SEC.STRUCTURE .ps)
354	AATTGATGTGTTCCAACAGCAGACGGGAGGTG	hsa-miR-141-5p MIM	caucuuccaguacaguguugga	-23.52	image
315	CTATTGTGAACGACAGTTGGAAATGGAGGATG	hsa-miR-141-5p MIM	caucuuccaguacaguguugga	-23.1	image

Results in .CSV format (Right click and 'Save as')

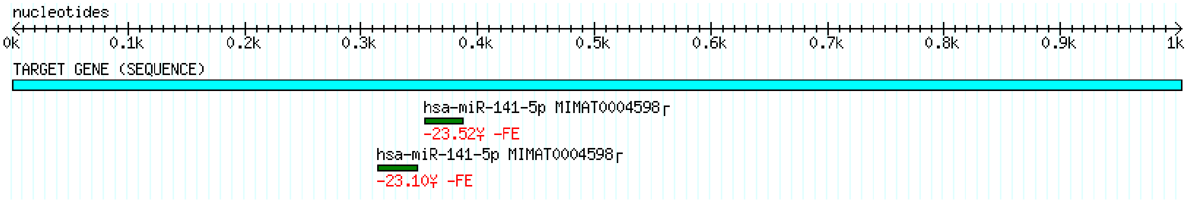

RESULTS

POSITION	SEQUENCE OF TARGET	NAME OF MIRNA	SEQUENCE OF MIRNA	FREE ENERGY	LINK (SEC.STRUCTURE .ps)
501	CATCCTCGCATTGCTGTTGAATGGTGAGCACG	hsa-miR-200c-5p MI	cgucuuacccagcaguguuugg	-26.61	image
408	CATCGACGTGTTCCAGCAGCAGACGGGAGGTG	hsa-miR-141-5p MIM	caucuuccaguacaguguugga	-23.24	image
1686	TTCCTGTTTGCTGTATGGGCTCGGGTGGGATG	hsa-miR-200b-5p MI	caucuuacugggcagcauugga	-21	image
1686	TTCCTGTTTGCTGTATGGGCTCGGGTGGGATG	hsa-miR-200a-5p MI	caucuuaccggacagugcugga	-20.79	image

Results in .CSV format (Right click and 'Save as')

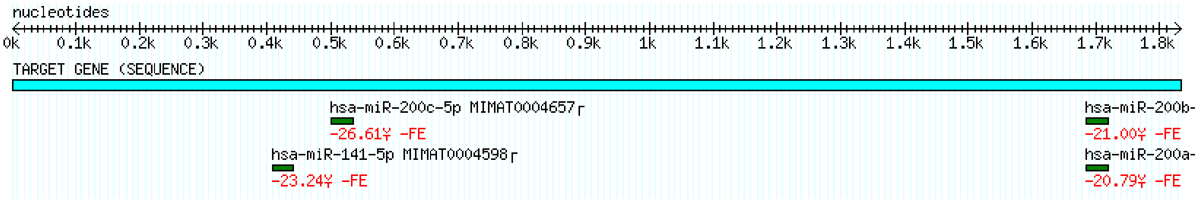

RESULTS

POSITION	SEQUENCE OF TARGET	NAME OF MIRNA	SEQUENCE OF MIRNA	FREE ENERGY	LINK (SEC.STRUCTURE .ps)
312	GCAGTTTTACCCGAAAGCTAATATCGTTGCCT	hsa-miR-34c-5p MIM	aggcaguguaguuagcugauugc	-21.73	image
1728	AGTGGGGCCCAAACAAGCTGAAGATGCTGCCA	hsa-miR-34c-5p MIM	aggcaguguaguuagcugauugc	-21.6	image
687	ATCTCCTGACAGAGCTGACCCTGAAGCTGCCT	hsa-miR-34c-5p MIM	aggcaguguaguuagcugauugc	-21.37	image

Results in .CSV format (Right click and 'Save as')

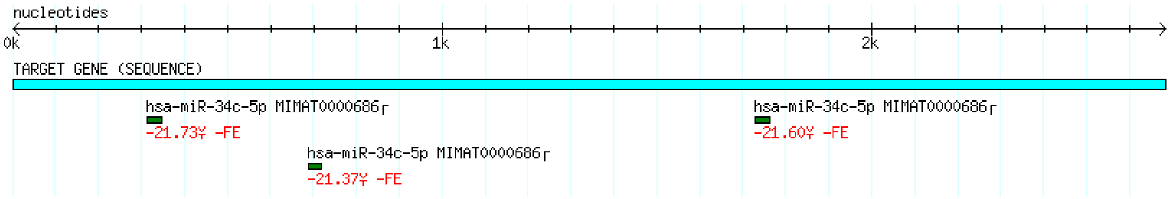

RESULTS

POSITION	SEQUENCE OF TARGET	NAME OF MIRNA	SEQUENCE OF MIRNA	FREE ENERGY	LINK (SEC.STRUCTURE .ps)
94	GGAAACCTCCGGGACCTGTGGCTGGAGAGGTG	hsa-miR-141-5p MIM	caucuuccaguacaguguugga	-25.98	image
1714	CACAGGGATGGAGCTGCGCTGTGCATAGGGTG	hsa-miR-200a-5p MI	caucuuaccggacagugcugga	-21.05	image
1574	GGCATCGAGACCCTGGCAACTGCACCGGTGCC	hsa-miR-34c-5p MIM	aggcaguguaguuagcugauugc	-20.01	image

Results in .CSV format (Right click and 'Save as')

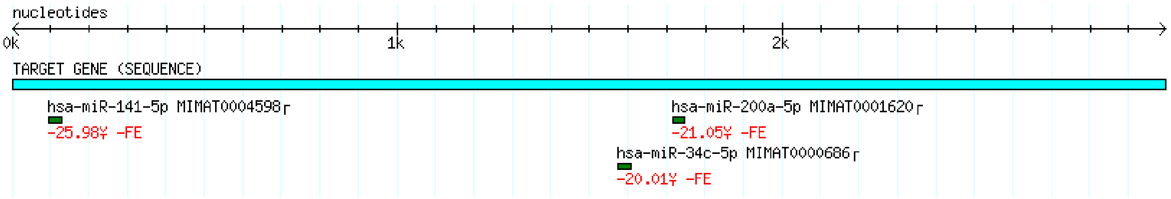

miRNA Target Sites Table View

<table>
<tr><td>Target Sequence</td><td>>
GCCCGCGCCAGGGTCCTCGGAGCTGCTCTGGCTGCGCGCGGAGCGGGCTCCGGAGGGAAGTCCCGAGACAAAGGGAAGCG
CCGCCGCCGCCGCCCCGCTCGGTCCTCCACCTGTCCGCTACGCTCGCCGGGGCTGCGGCCGCCCGAGGGACTTTGAACAT
GTCGGGGATCGCCCTCAGCAGACTCGCCCAGGAGAGGAAAGCATGGAGGAAAGACCACCCATTTGGTTTCGTGGCTGTCC
CAACAAAAAATCCCGATGGCACGATGAACCTCATGAACTGGGAGTGCGCCATTCCAGGAAAGAAAGGGACTCCGTGGGAA
GGAGGCTTGTTTAAACTACGGATGCTTTTCAAAGATGATTATCCATCTTCGCCACCAAAATGTAAATTCGAACCACCATT
ATTTCACCCGAATGTGTACCCTTCGGGGACAGTGTGCCTGTCCATCTTAGAGGAGGACAAGGACTGGAGGCCAGCCATCA
CAATCAAACAGATCCTATTAGGAATACAGGAACTTCTAAATGAACCAAATATCCAAGACCCAGCTCAAGCAGAGGCCTAC
ACGATTTACTGCCAAAACAGAGTGGAGTACGAGAAAAGGGTCCGAGCACAAGCCAAGAAGTTTGCGCCCTCATAAGCAGC
GACCTTGTGGCATCGTCAAAAGGAAGGGATTGGTTTGGCAAGAACTTGTTTACAACATTTTTGCAAATCTAAAGTTGCTC
CATACAATGACTAGTCACCTGGGGGGGTTGGGCGGGCGCCATCTTCCATTGCCGCCGCGGGTGTGCGGTCTCGATTCGCT
GAATTGCCCGTTTCCATACAGGGTCTCTTCCTTCGGTCTTTTGTATTTTTGATTGTTATGTAAAACTCGCTTTTATTTTA
ATATTGATGTCAGTATTTCAACTGCTGTAAAATTATAAACTTTTATACTTGGGTAAGTCCCCCAGGGGCGAGTTCCTCGC
TCTGGGATGCAGGCATGCTTCTCACCGTGCAGAGCTGCACTTGGCCTCAGCTGGCTGTATGGAAATGCACCCTCCCTCCT
GCCGCTCCTCTCTAGAACCTTCTAGAACCTGGGCTGTGCTGCTTTTGAGCCTCAGACCCCAGGTCAGCATCTCGGTTCTG
CGCCACTTCCTTTGTGTTTATATGGCGTTTTGTCTGTGTTGCTGTTTAGAGTAAATAAACTGTTTATATAAAGGTTTTGG
TTGCATTATTATCATTGAAAGTGAGAGGAGGCGGCCTCCCAGTGCCCGGCCCTCCCCACCCACCTGCAGCCCCACCGCGG
GCCAGGACCAGGCTCTCCATCTGCTTCGGATGCACGCAGGCTGTGAGGCTCTGTCTTGCCCTGGATCTTTGTAAACAGGG
CTGTGTACAAAGTGCTGCTGAGGTTTCTGTGCTCCCCGCATCTGCGGGCTGTAGAGCGCTGGGCAGCTAAGATCTGCATA
GGTCGGGATTGGCATCGAGACCCTGGCAACTGCACCGGTGCCAGCTGTCTTGGGGGCCACAAGGCCAGGTCCAGACCAGG
GCTGGGGGCTGCCTGAGGACTCCTATCCGGGCAGCCTGCTGGCGGGGGTTCCCCTCTTCAGTGGCCAGGTCACAGGGATG
GAGCTGCGCTGTGCATAGGGTGCCACCTCAGGTGTCTGTCCCTTGTGTCCTCAGGAGGCAGCCTTGCTACCACCCGTGGC
AAACGCCAGGTGCTTTTTCTGGGAGAGCCCACAGCCGTGGCCCTCCAGGGCTTCCCCGACCCTTAGCGCCAGGTAGAGGG
CCCTGGGCAGCCTGTGTCTGGAATTCTTCGTCCTGAGGCCACCTGAGTGTGGTCTGTCCTGGGGAGGCTGTGCGCCTCAG
CAGCCGTCCTGACGCTGAGCCCTCTGCAAAGGTTGGGCCGGCCAGGCCTCTTGGGGCTGCCTGAGCCACTGCAGGAAGTG
GCCTGGCTGGGAAGTTGGGTGCCGGTCACCTCCCAGCAGGAAGGCACAGTGGACAGAGATGGGAAGCCCTGGGGGACACA
GCCCGGTGCTCCCAGCCCTCCAACCTCTGGCTCCCAACCCAGTCTCCCCATCCTAGCGAGCTTGGCCCTCCTCAGTTTCG
TTTCAAGCCTTGGGGCTGGAGCTGGCCCTGCTGCCCTGGCACCCCCCGGTGGCTGGAGCTGGGTCCCCGTGGCCCAAGTG
CAGGGTCCCAAGAGGGCAGGGCGGGGCTCCCCAAAGGAGCAAAGAATGCAGGGAGGGCGGTCCAGGGCCCTGGGAAGGGG
AGCTCGGCACCCTCCAGGTCCGTGTGGGACTCCAGCCGCTGTTGGCTGGGAATCGAAGTTAGAGGTGACTTCCAAAGGCC
CCCCGAGCCGGCAGTGCCCCCCACCACCCCTCCAGCGACTCTGCGGTGCCAGTGCCTTGTTGGCTTTTCCGGCTACGCAC
CCTGCAGTCACTGAGCTCTCGGTCTGACGTCTGATGTTTGTGGTTTGTTTATAACACGGGGCCTTACCTGGGGAATTCAG
CTGGTTTGAATATTTGTAGCCCGCTCCCAGAATGTCTTATTTTGTAATGACTGAACTACATTTAGTAATAGTTACACATG
TATATGGTTAATACATATGGAAATTCAATATATTTTGTAGTTAACGTATTCTGAAGTAACGGATGTTTCTCGCCAATCGT
AGTGACTTCAGCTAACGAAATGTTCTTTTGTAGTACCACGGTCCTCGGCCTAACGAAGGACGTGAACCTTGTAAGAGGAG
AGCTCTGAAACGCGGTCACCTTTGTTTAGTGGAAGGGAAAGTGTGTTCCCGGCATGAGGTGCCTCGGAATTAGTAAAGAA
TTGTGGGCAATGGATTAACCACTGTATCTAAGAATCCACCATTAAAGCATTTGCACAGACAAAAAAAAAAA</td></tr>
</table>

<table>
<tr><th>No.</th><th>miRNA ID</th><th>Location</th><th>Len</th><th>Hybridization</th><th>Minimum Free Energy</th><th>Score</th><th>Profile</th></tr>
<tr><td>1</td><td>hsa-let-7a-2*</td><td>1592~1616</td><td>25</td><td><pre>miRNA: 3' ccUUUCGAUCCUC----CGACAUGUc 5'
            |::| | |||| |||||:||
Target:5' acAGGGAT-GGAGCTGCGCTGTGCAt 3'</pre></td><td>-15.80</td><td>141.00</td><td>?</td></tr>
<tr><td>2</td><td>hsa-let-7f-2*</td><td>1349~1368</td><td>20</td><td><pre>miRNA: 3' ccuuUCUGUCAUCUGACAUAUc 5'
              | |||| |:||||:||
Target:5' ttgtAAACAG--GGCTGTGTAc 3'</pre></td><td>-11.70</td><td>142.00</td><td>?</td></tr>
<tr><td>3</td><td>hsa-let-7i*</td><td>607~629</td><td>23</td><td><pre>miRNA: 3' ucGUUCCG-UCAUCGAACGCGuc 5'
            |||| | || ||:||||||
Target:5' caCAAGCCAAGAAGTTTGCGCcc 3'</pre></td><td>-17.70</td><td>152.00</td><td>?</td></tr>
</table>

<table>
<tr><td rowspan="2">4</td><td rowspan="2">hsa-miR-103</td><td>1355~1379</td><td>25</td><td>miRNA: 3' agUAUCGGGACAUG--UUACGACGa 5'
| :||: ||||| |:||||||
Target:5' acAGGGCTGTGTACAAAGTGCTGCt 3'</td><td>-23.50</td><td>155.00</td><td rowspan="2"></td></tr>
<tr><td>1060~1083</td><td>24</td><td>miRNA: 3' aguAUC-GGGACAUGUUACGACGa 5'
||| |||| :| :||||||
Target:5' ttcTAGAACCTGGGCTGTGCTGCt 3'</td><td>-18.50</td><td>148.00</td></tr>
<tr><td>5</td><td>hsa-miR-103-as</td><td>1342~1366</td><td>25</td><td>miRNA: 3' ucgUCGUAACA--UGUCCCGAUACu 5'
| | |||| ||||||||:||
Target:5' tggATCTTTGTAAACAGGGCTGTGt 3'</td><td>-20.10</td><td>158.00</td><td></td></tr>
<tr><td>6</td><td>hsa-miR-103-2*</td><td>2177~2201</td><td>25</td><td>miRNA: 3' guUCCGUCGUGA--CAUUUCUUCGa 5'
:|||:| :|| ||||:|||
Target:5' caGGGCGGGGCTCCCCAAAGGAGCa 3'</td><td>-21.50</td><td>143.00</td><td></td></tr>
<tr><td rowspan="2">7</td><td rowspan="2">hsa-miR-103</td><td>1355~1379</td><td>25</td><td>miRNA: 3' agUAUCGGGACAUG--UUACGACGa 5'
| :||: ||||| |:||||||
Target:5' acAGGGCTGTGTACAAAGTGCTGCt 3'</td><td>-23.50</td><td>155.00</td><td rowspan="2"></td></tr>
<tr><td>1060~1083</td><td>24</td><td>miRNA: 3' aguAUC-GGGACAUGUUACGACGa 5'
||| |||| :| :||||||
Target:5' ttcTAGAACCTGGGCTGTGCTGCt 3'</td><td>-18.50</td><td>148.00</td></tr>
<tr><td>8</td><td>hsa-miR-103-as</td><td>1342~1366</td><td>25</td><td>miRNA: 3' ucgUCGUAACA--UGUCCCGAUACu 5'
| | |||| ||||||||:||
Target:5' tggATCTTTGTAAACAGGGCTGTGt 3'</td><td>-20.10</td><td>158.00</td><td></td></tr>
<tr><td rowspan="2">9</td><td rowspan="2">hsa-miR-105</td><td>2833~2854</td><td>22</td><td>miRNA: 3' ugguGUCCUCAGACUCGUAAACu 5'
|| | |: ||||||||
Target:5' aatcCACCA-TTAAAGCATTTGc 3'</td><td>-10.96</td><td>146.00</td><td rowspan="2"></td></tr>
<tr><td>2833~2854</td><td>22</td><td>miRNA: 3' ugguGUCCUCAGACUCGUAAACu 5'
|| | |: ||||||||
Target:5' aatcCACCA-TTAAAGCATTTGc 3'</td><td>-10.96</td><td>146.00</td></tr>
<tr><td>10</td><td>hsa-miR-106b*</td><td>409~437</td><td>29</td><td>miRNA: 3' cgUCGUUCAUGGG-------UGUCACGCc 5'
| :: |||||| ||||||:|
Target:5' cgAATGTGTACCCTTCGGGGACAGTGTGc 3'</td><td>-16.91</td><td>140.00</td><td></td></tr>
<tr><td rowspan="2">11</td><td rowspan="2">hsa-miR-107</td><td>1355~1379</td><td>25</td><td>miRNA: 3' acUAUCGGGACAUG--UUACGACGa 5'
| :||: ||||| |:||||||
Target:5' acAGGGCTGTGTACAAAGTGCTGCt 3'</td><td>-21.60</td><td>155.00</td><td rowspan="2"></td></tr>
<tr><td>1060~1083</td><td>24</td><td>miRNA: 3' acuAUC-GGGACAUGUUACGACGa 5'
[illegible]</td><td>-18.10</td><td>148.00</td></tr>
</table>

<table>
<tr><td></td><td></td><td></td><td></td><td>||| |||| ·| ·||||||
Target:5' ttcTAGAACCTGGGCTGTGCTGCt 3'</td><td></td><td></td><td></td></tr>
<tr><td>12</td><td>hsa-miR-10a</td><td>798~825</td><td>28</td><td>miRNA: 3' guGUUUAA---GCCUAG--AUGUCCCAu 5'
::|||| || || ||||||||
Target:5' gcTGAATTGCCCGTTTCCATACAGGGTc 3'</td><td>-12.40</td><td>159.00</td><td></td></tr>
<tr><td>13</td><td>hsa-miR-10b</td><td>798~825</td><td>28</td><td>miRNA: 3' guGUUUAA---GCCAAG--AUGUCCCAu 5'
::|||| || ||| ||||||||
Target:5' gcTGAATTGCCCGTTTCCATACAGGGTc 3'</td><td>-13.40</td><td>167.00</td><td></td></tr>
<tr><td>14</td><td>hsa-miR-1178</td><td>2790~2810</td><td>21</td><td>miRNA: 3' gaUCCCUUCUUGUCACUCGUu 5'
|| |||||: |||:|||
Target:5' ttAGTAAAGAATTGTGGGCAa 3'</td><td>-16.80</td><td>151.00</td><td></td></tr>
<tr><td>15</td><td>hsa-miR-1179</td><td>2187~2211</td><td>25</td><td>miRNA: 3' gguuGGUUAC----UUUCUUACGaa 5'
|||| | |||||||||
Target:5' ctccCCAAAGGAGCAAAGAATGCag 3'</td><td>-12.00</td><td>143.00</td><td></td></tr>
<tr><td>16</td><td>hsa-miR-1182</td><td>1443~1465</td><td>23</td><td>miRNA: 3' caguguagggagGGUUCUGGGAg 5'
:|:|||||||
Target:5' tcgggattggcaTCGAGACCCTg 3'</td><td>-15.82</td><td>147.00</td><td></td></tr>
<tr><td rowspan="2">17</td><td rowspan="2">hsa-miR-1183</td><td>1244~1271</td><td>28</td><td>miRNA: 3' acGGGUGAGAGUGGU-AGUGGAUGUCac 5'
|| :| ||| ||| |||||:|||
Target:5' gcCCGGCCCTCCCCACCCACCTGCAGcc 3'</td><td>-28.60</td><td>145.00</td><td rowspan="2"></td></tr>
<tr><td>2381~2409</td><td>29</td><td>miRNA: 3' acgGGUGAGAGUGGU-AGU-GGAUGUCAc 5'
|: :|| :|:| || |||:||||
Target:5' tggCTTTTCCGGCTACGCACCCTGCAGTc 3'</td><td>-18.60</td><td>140.00</td></tr>
<tr><td>18</td><td>hsa-miR-1197</td><td>1632~1652</td><td>21</td><td>miRNA: 3' ucuucaucuGGUACACAGGAu 5'
|| ||||||||
Target:5' gtgtctgtcCCTTGTGTCCTc 3'</td><td>-13.30</td><td>152.00</td><td></td></tr>
<tr><td>19</td><td>hsa-miR-1200</td><td>1636~1657</td><td>22</td><td>miRNA: 3' cuccgagucuuaccGAGUCCUc 5'
|||||||
Target:5' ctgtcccttgtgtcCTCAGGAg 3'</td><td>-13.90</td><td>140.00</td><td></td></tr>
<tr><td rowspan="2">20</td><td rowspan="2">hsa-miR-1203</td><td>29~54</td><td>26</td><td>miRNA: 3' cuCGACGU-AG----GACCGAGGCCc 5'
|||||: | | ||||||||
Target:5' tgGCTGCGCGCGGAGCGGGCTCCGGa 3'</td><td>-24.60</td><td>153.00</td><td rowspan="2"></td></tr>
<tr><td>1292~1310</td><td>19</td><td>miRNA: 3' cucGACGUAGGACCGAGGCCc 5'
|| ||||: |||:|||
Target:5' ggtCTCCATCT-CCTTCCCa 3'</td><td>-15.40</td><td>142.00</td></tr>
</table>

<table>
<tr><td rowspan="2"></td><td rowspan="2"></td><td></td><td></td><td>Target:5' [illegible]</td><td></td><td></td><td rowspan="2"></td></tr>
<tr><td>946~966</td><td>21</td><td>miRNA: 3' cucgacguAGGACCGAGGCCc 5'
|||| ||||:||
Target:5' gggcgagtTCCTCGCTCTGGg 3'</td><td>-24.70</td><td>141.00</td></tr>
<tr><td rowspan="2">21</td><td rowspan="2">hsa-miR-1205</td><td>2391~2408</td><td>18</td><td>miRNA: 3' gaGUUUCGUUUGGGACGUCu 5'
| | || |||||||||
Target:5' ggCTACGC--ACCCTGCAGt 3'</td><td>-18.70</td><td>154.00</td><td rowspan="2"></td></tr>
<tr><td>1250~1270</td><td>21</td><td>miRNA: 3' gaguuucGUUUG-GGACGUCu 5'
|| | |||||||
Target:5' ccctcccCACCCACCTGCAGc 3'</td><td>-12.73</td><td>141.00</td></tr>
<tr><td rowspan="2">22</td><td rowspan="2">hsa-miR-1206</td><td>262~279</td><td>18</td><td>miRNA: 3' cgaAUUUGUAGAUGUACUUGu 5'
|:||| || |||||||
Target:5' cgaTGAAC--CT-CATGAACt 3'</td><td>-9.30</td><td>153.00</td><td rowspan="2"></td></tr>
<tr><td>506~526</td><td>21</td><td>miRNA: 3' cgaaUUUGUAGAUGUACUUGu 5'
:||| |||| ||||||
Target:5' acagGAACTTCTAAATGAACc 3'</td><td>-10.70</td><td>141.00</td></tr>
<tr><td>23</td><td>hsa-miR-1207-3p</td><td>1471~1488</td><td>18</td><td>miRNA: 3' cuuuacUCCCGGUCGACu 5'
:| ||||||||
Target:5' tgcaccGGTGCCAGCTGt 3'</td><td>-16.20</td><td>148.00</td><td></td></tr>
<tr><td>24</td><td>hsa-miR-122*</td><td>1128~1149</td><td>22</td><td>miRNA: 3' auaAAUCACACUAU-UACCGCAa 5'
|| |||| || |||||||
Target:5' tccTTTGTGT-TTATATGGCGTt 3'</td><td>-14.00</td><td>158.00</td><td></td></tr>
<tr><td>25</td><td>hsa-miR-1224-5p</td><td>1636~1654</td><td>19</td><td>miRNA: 3' ggUGGAGGGCUCAGGAGUg 5'
::| |::| |||||||
Target:5' ctGTCCCTTGTGTCCTCAg 3'</td><td>-17.60</td><td>153.00</td><td></td></tr>
<tr><td rowspan="2">26</td><td rowspan="2">hsa-miR-1225-3p</td><td>2170~2190</td><td>21</td><td>miRNA: 3' gaCCCCCGCCGUGUCCCCGAGu 5'
| |||| | :| |||||||
Target:5' aaGAGGGCAGGGC-GGGGCTCc 3'</td><td>-24.00</td><td>159.00</td><td rowspan="2"></td></tr>
<tr><td>1547~1572</td><td>26</td><td>miRNA: 3' gaCCC--CCGCCG--UGUCCCCGAGu 5'
||| | | || :|:||||:||
Target:5' ccGGGCAGCCTGCTGGCGGGGGTTCc 3'</td><td>-22.60</td><td>140.00</td></tr>
<tr><td></td><td></td><td>607~633</td><td>27</td><td>miRNA: 3' ggGGUAGG-UC--CGGACGUACGGGAGUg 5'
| | || || |::||| |||||||
Target:5' caCAAGCCAAGAAGTTTGC--GCCCTCAt 3'</td><td>-19.70</td><td>158.00</td><td></td></tr>
</table>

<table>
<tr><td rowspan="7">27</td><td rowspan="7">hsa-miR-1226*</td><td>2046~2072</td><td>27</td><td>miRNA: 3' ggGGUAGGUCCGGACGUA-CGGGAGug 5'
|||||| || || | ||||||
Target:5' ccCCATCCTAGCGAGCTTGGCCCTCct 3'</td><td>-30.40</td><td>152.00</td><td rowspan="7">?</td></tr>
<tr><td>155~178</td><td>24</td><td>miRNA: 3' gggGUAGGUCCGGACGUA-CGGGAGUg 5'
||| : || | || |||||||
Target:5' gaaCATGTCGG---GGATCGCCCTCAg 3'</td><td>-17.70</td><td>152.00</td></tr>
<tr><td>983~1010</td><td>28</td><td>miRNA: 3' ggGGUAGGUCCGGACGU--ACGGGAGUg 5'
||:| ||| ||||| || |||||
Target:5' caCCGTGCAGAGCTGCACTTGGCCTCAg 3'</td><td>-26.50</td><td>150.00</td></tr>
<tr><td>1621~1654</td><td>34</td><td>miRNA: 3' ggGGUAG-GUC--CGGAC-----GUACGGGAGUg 5'
||| | ||| |:||| ::||:|||||
Target:5' tgCCACCTCAGGTGTCTGTCCCTTGTGTCCTCAg 3'</td><td>-25.30</td><td>150.00</td></tr>
<tr><td>1842~1866</td><td>25</td><td>miRNA: 3' ggGGUAGGUCCG-GACGUACGGGAGug 5'
||:||| | | ||| | ||||||
Target:5' agCCGTCCTGACGCTG-A-GCCCTCtg 3'</td><td>-25.10</td><td>146.00</td></tr>
<tr><td>1996~2022</td><td>27</td><td>miRNA: 3' gggGUAGGUCCG-GACGUACGGGAGug 5'
|| || ||: || | ||||||
Target:5' acaCAGCCCGGTGCTCCCAGCCCTCca 3'</td><td>-20.70</td><td>143.00</td></tr>
<tr><td>1233~1256</td><td>24</td><td>miRNA: 3' ggGGUAGGUC-CGGACGUACGGGAGug 5'
|| |||| ||| | ||||||
Target:5' ggCCTCCCAGTGCCCG---GCCCTCcc 3'</td><td>-26.60</td><td>141.00</td></tr>
<tr><td>28</td><td>hsa-miR-1226</td><td>2468~2487</td><td>20</td><td>miRNA: 3' gaUCCCUUGUGUCCCGACCAcu 5'
:|||||: || ||||||
Target:5' ctGGGGAATTCA--GCTGGTtt 3'</td><td>-20.20</td><td>144.00</td><td>?</td></tr>
<tr><td>29</td><td>hsa-miR-1228*</td><td>78~97</td><td>20</td><td>miRNA: 3' guguGUGGACGGGGGCGGGug 5'
|:|| ||| ||||||
Target:5' gcgcCGCC-GCCGCCGCCCcg 3'</td><td>-30.30</td><td>140.00</td><td>?</td></tr>
<tr><td>30</td><td>hsa-miR-1228</td><td>767~786</td><td>20</td><td>miRNA: 3' ccccCCGCUCCGUCCACACu 5'
| || ||:||||||
Target:5' cattGCCGCCGCGGGTGTGc 3'</td><td>-16.60</td><td>140.00</td><td>?</td></tr>
<tr><td rowspan="2">31</td><td rowspan="2">hsa-miR-1233</td><td>2170~2190</td><td>21</td><td>miRNA: 3' gaCGCCC-UCCUGUCCCGAGu 5'
| ||| |||:|:||||||
Target:5' aaGAGGGCAGGGCGGGGCTCc 3'</td><td>-27.90</td><td>154.00</td><td rowspan="2">?</td></tr>
<tr><td></td><td></td><td>miRNA: 3' gaCGCCC-UCCUGUCCCGAGu 5'</td><td></td><td></td></tr>
</table>

<table>
<tr><td></td><td></td><td>2170~2190</td><td>21</td><td>miRNA: 3' gaCGCCC-UCCUGUCCCGAGu 5'
| ||| |||:|:||||||
Target:5' aaGAGGGCAGGGCGGGGCTCc 3'</td><td>-27.90</td><td>154.00</td><td></td></tr>
<tr><td>32</td><td>hsa-miR-1234</td><td>1868~1890</td><td>23</td><td>miRNA: 3' cacCCCACCC-ACCAGUCCGGcu 5'
|| |||| || ||||||
Target:5' aaaGGTTGGGCCGGCCAGGCCtc 3'</td><td>-21.10</td><td>143.00</td><td></td></tr>
<tr><td>33</td><td>hsa-miR-1236</td><td>60~81</td><td>22</td><td>miRNA: 3' gaccuCUCUGUUCCCCUUCUCc 5'
||||||| |||||| |
Target:5' gtcccGAGACAAAGGGAAGCGc 3'</td><td>-17.82</td><td>145.00</td><td></td></tr>
<tr><td rowspan="3">34</td><td rowspan="3">hsa-miR-124</td><td>2358~2379</td><td>22</td><td>miRNA: 3' ccguAAGUGGCG--CACGGAAu 5'
| |: :|| |||||||
Target:5' actcTGCGGTGCCAGTGCCTTg 3'</td><td>-16.00</td><td>146.00</td><td rowspan="3"></td></tr>
<tr><td>2358~2379</td><td>22</td><td>miRNA: 3' ccguAAGUGGCG--CACGGAAu 5'
| |: :|| |||||||
Target:5' actcTGCGGTGCCAGTGCCTTg 3'</td><td>-16.00</td><td>146.00</td></tr>
<tr><td>2358~2379</td><td>22</td><td>miRNA: 3' ccguAAGUGGCG--CACGGAAu 5'
| |: :|| |||||||
Target:5' actcTGCGGTGCCAGTGCCTTg 3'</td><td>-16.00</td><td>146.00</td></tr>
<tr><td rowspan="6">35</td><td rowspan="6">hsa-miR-1244</td><td>2515~2541</td><td>27</td><td>miRNA: 3' uuGGUAGAGUAUGUUUGG-UUGAUGaa 5'
::||:|::|| :||: ||||||
Target:5' tcTTATTTTGTAATGACTGAACTACat 3'</td><td>-8.80</td><td>148.00</td><td rowspan="6"></td></tr>
<tr><td>883~907</td><td>25</td><td>miRNA: 3' uuggUAGAGUAUGUUUGGUUGAUGAa 5'
|| ||| ::| :|||||:||
Target:5' attgATGTCA-GTATTTCAACTGCTg 3'</td><td>-12.80</td><td>145.00</td></tr>
<tr><td>2515~2541</td><td>27</td><td>miRNA: 3' uuGGUAGAGUAUGUUUGG-UUGAUGaa 5'
::||:|::|| :||: ||||||
Target:5' tcTTATTTTGTAATGACTGAACTACat 3'</td><td>-8.80</td><td>148.00</td></tr>
<tr><td>883~907</td><td>25</td><td>miRNA: 3' uuggUAGAGUAUGUUUGGUUGAUGAa 5'
|| ||| ::| :|||||:||
Target:5' attgATGTCA-GTATTTCAACTGCTg 3'</td><td>-12.80</td><td>145.00</td></tr>
<tr><td>2515~2541</td><td>27</td><td>miRNA: 3' uuGGUAGAGUAUGUUUGG-UUGAUGaa 5'
::||:|::|| :||: ||||||
Target:5' tcTTATTTTGTAATGACTGAACTACat 3'</td><td>-8.80</td><td>148.00</td></tr>
<tr><td>883~907</td><td>25</td><td>miRNA: 3' uuggUAGAGUAUGUUUGGUUGAUGAa 5'</td><td>12.80</td><td>145.00</td></tr>
</table>

<table>
<tr><td></td><td></td><td>[illegible]</td><td>[illegible]</td><td>[illegible]
|| ||| ::| :|||||:||
Target:5' attgATGTCA-GTATTTCAACTGCTg 3'</td><td>[illegible]</td><td>[illegible]</td><td></td></tr>
<tr><td>36</td><td>hsa-miR-1246</td><td>2821~2840</td><td>20</td><td>miRNA: 3' ggACG-AGGUUUUUAGGUaa 5'
||: ||:||:||||||
Target:5' acTGTATCTAAGAATCCAcc 3'</td><td>-16.50</td><td>145.00</td><td>?</td></tr>
<tr><td>37</td><td>hsa-miR-1247</td><td>738~757</td><td>20</td><td>miRNA: 3' agGCCCCUGCUUGCCCUGCCCa 5'
:|||| |::: ||:||||
Target:5' ccTGGGG--GGGTTGGGCGGGc 3'</td><td>-28.80</td><td>140.00</td><td>?</td></tr>
<tr><td>38</td><td>hsa-miR-1248</td><td>2148~2177</td><td>30</td><td>miRNA: 3' aaAUCGUG-UCACG-AAUA-UGUUCUUCCa 5'
|:|| | ||||| :| |||||:||
Target:5' cgTGGCCCAAGTGCAGGGTCCCAAGAGGGc 3'</td><td>-18.30</td><td>145.00</td><td>?</td></tr>
<tr><td>39</td><td>hsa-miR-1249</td><td>2200~2220</td><td>21</td><td>miRNA: 3' acUUCUUCCCCCCCUUCCCGCa 5'
||||| | ||:||||||
Target:5' caAAGAA-TGCAGGGAGGGCGg 3'</td><td>-19.10</td><td>147.00</td><td>?</td></tr>
<tr><td rowspan="2">40</td><td rowspan="2">hsa-miR-1251</td><td>2084~2102</td><td>19</td><td>miRNA: 3' ucgCGGAAACCGUCGAUCUCa 5'
||||| || |||:|||
Target:5' caaGCCTTGGG--GCTGGAGc 3'</td><td>-19.10</td><td>146.00</td><td rowspan="2">?</td></tr>
<tr><td>2275~2305</td><td>31</td><td>miRNA: 3' ucGCGGAAACCG----------UCGAUCUCa 5'
|||: ||||| ||:|||||
Target:5' gcCGCTGTTGGCTGGGAATCGAAGTTAGAGg 3'</td><td>-18.30</td><td>141.00</td></tr>
<tr><td>41</td><td>hsa-miR-1252</td><td>816~835</td><td>20</td><td>miRNA: 3' auuuaCUUA-AGUUAAAGGAAGa 5'
|::| || |||||||
Target:5' atacaGGGTCTC---TTCCTTCg 3'</td><td>-7.80</td><td>142.00</td><td>?</td></tr>
<tr><td rowspan="3">42</td><td rowspan="3">hsa-miR-1254</td><td>2235~2260</td><td>26</td><td>miRNA: 3' ugacgucCGAG--GUCGAAGGUCCGa 5'
|||| || | ||||||:
Target:5' aaggggaGCTCGGCACCCTCCAGGTc 3'</td><td>-17.90</td><td>143.00</td><td rowspan="3">?</td></tr>
<tr><td>1532~1553</td><td>22</td><td>miRNA: 3' ugACGUCCGAGGUCGAAGGUCCGa 5'
|| :| |||| : |||:|||
Target:5' ccTGAGGACTCC--TATCCGGGCa 3'</td><td>-22.60</td><td>142.00</td></tr>
<tr><td>278~300</td><td>23</td><td>miRNA: 3' ugaCGUC-CGAGGUCGAAGGUCCga 5'
| || || ||| |||||||
Target:5' ctgGGAGTGCGCCA--TTCCAGGaa 3'</td><td>-18.70</td><td>141.00</td></tr>
<tr><td></td><td>hsa-[illegible]</td><td>716~742</td><td>27</td><td>miRNA: 3' ccGAGG---GUUCUUGG--AGUGGACa 5'
|||| ||| :||: |||||||
Target:5' tgCTCCATACAATGACTAGTCACCTGg 3'</td><td>-19.00</td><td>162.00</td><td></td></tr>
</table>

<table>
<tr><th></th><th></th><th></th><th></th><th></th><th></th><th></th><th></th></tr>
<tr><td rowspan="2">43</td><td rowspan="2">hsa-
miR-
125a-
3p</td><td>[illegible]</td><td>[illegible]</td><td>[illegible]</td><td>[illegible]</td><td>[illegible]</td><td rowspan="2">?</td></tr>
<tr><td>2449~2471</td><td>23</td><td>miRNA: 3' ccgaggGU-UCUUGGAGUGGACa 5'
                || :|::|||:|||||
Target:5' ttataaCACGGGGCCTTACCTGg 3'</td><td>-19.60</td><td>144.00</td></tr>
<tr><td>44</td><td>hsa-
miR-
126*</td><td>2530~2553</td><td>24</td><td>miRNA: 3' gcGCAUGGUUU---UCAUUAUUAc 5'
            :| ||:| | |||||||:|
Target:5' acTGAACTACATTTAGTAATAGTt 3'</td><td>-7.60</td><td>143.00</td><td>?</td></tr>
<tr><td>45</td><td>hsa-
miR-
1260</td><td>1967~1985</td><td>19</td><td>miRNA: 3' acCACC-GUCUCCACCCUa 5'
            |||| ||||| |||||
Target:5' caGTGGACAGAGATGGGAa 3'</td><td>-20.60</td><td>140.00</td><td>?</td></tr>
<tr><td>46</td><td>hsa-
miR-
1261</td><td>346~366</td><td>21</td><td>miRNA: 3' uucGGUUUCGG--AAUAGGUa 5'
             :||||| : |||||||
Target:5' tttTCAAAGATGATTATCCAt 3'</td><td>-13.40</td><td>154.00</td><td>?</td></tr>
<tr><td>47</td><td>hsa-
miR-
1262</td><td>170~191</td><td>22</td><td>miRNA: 3' uaGGAAGAUGUUUAAGUGGGUa 5'
            || || :||:| ||:||||
Target:5' cgCCCTCAGCAGACTCGCCCAg 3'</td><td>-17.90</td><td>152.00</td><td>?</td></tr>
<tr><td>48</td><td>hsa-
miR-
1263</td><td>1604~1626</td><td>23</td><td>miRNA: 3' ugaGUCAUACG-GUCCCAUGGUa 5'
             |: |:||| :|||||:|||
Target:5' ctgCGCTGTGCATAGGGTGCCAc 3'</td><td>-22.10</td><td>151.00</td><td>?</td></tr>
<tr><td rowspan="3">49</td><td rowspan="3">hsa-
miR-
1266</td><td>1774~1798</td><td>25</td><td>miRNA: 3' ucgGGAC---AAGAUGUCGGGACUCc 5'
             :||| |||| | |:||||||
Target:5' gtgTCTGGAATTCTTC-GTCCTGAGg 3'</td><td>-22.40</td><td>147.00</td><td rowspan="3">?</td></tr>
<tr><td>2213~2235</td><td>23</td><td>miRNA: 3' ucgggaCAAGAUGUCGGGACUCc 5'
                | || | :||||||:|
Target:5' gagggcGGTCCAGGGCCCTGGGa 3'</td><td>-18.30</td><td>141.00</td></tr>
<tr><td>1746~1768</td><td>23</td><td>miRNA: 3' ucgGGACAAGAUGUCGGGACUCc 5'
             || | | | :||||||:|
Target:5' gcgCCAGGTAGAGGGCCCTGGGc 3'</td><td>-23.00</td><td>140.00</td></tr>
<tr><td rowspan="2">50</td><td rowspan="2">hsa-
miR-
1267</td><td>163~182</td><td>20</td><td>miRNA: 3' acCCCUAAUGUGAAGUUGUCc 5'
            ||||| :| | |||:|||
Target:5' cgGGGATCGC-CCTCAGCAGa 3'</td><td>-18.50</td><td>146.00</td><td rowspan="2">?</td></tr>
<tr><td>1820~1844</td><td>25</td><td>miRNA: 3' acCCCU---A-AUGUGAAGUUGUCc 5'
            |||| | |:|:| |||:|||
Target:5' tgGGGAGGCTGTGCGCCTCAGCAGc 3'</td><td>-22.20</td><td>143.00</td></tr>
</table>

<table>
<tr><td rowspan="2">51</td><td rowspan="2">hsa-miR-1271</td><td>1603~1627</td><td>25</td><td>miRNA: 3' acuCACGA-ACG-AU-CCACGGUuc 5'
   | ||| ||| || |||||||
Target:5' gctGCGCTGTGCATAGGGTGCCAcc 3'</td><td>-14.90</td><td>143.00</td><td rowspan="2">?</td></tr>
<tr><td>2352~2373</td><td>22</td><td>miRNA: 3' acUCAC-GA-ACGAUCCACGGUUc 5'
  || | || ||| |||||||:
Target:5' ccAGCGACTCTGC--GGTGCCAGt 3'</td><td>-14.30</td><td>140.00</td></tr>
<tr><td>52</td><td>hsa-miR-1272</td><td>1188~1216</td><td>29</td><td>miRNA: 3' aaagUCUUAAA-CGACG--GUAGUAGUAg 5'
    ||: ||| |:||| :||:|||||
Target:5' ataaAGGTTTTGGTTGCATTATTATCATt 3'</td><td>-15.10</td><td>156.00</td><td>?</td></tr>
<tr><td rowspan="2">53</td><td rowspan="2">hsa-miR-1273e</td><td>526~552</td><td>27</td><td>miRNA: 3' aggUGAAGGACC-----CAAGUUCGUu 5'
   |: ||| | | |||||||
Target:5' caaATATCCAAGACCCAGCTCAAGCAg 3'</td><td>-14.00</td><td>143.00</td><td rowspan="2">?</td></tr>
<tr><td>2071~2090</td><td>20</td><td>miRNA: 3' agGUGAAGGACCCAAGUUCGuu 5'
  || ||:| | |||||||
Target:5' ctCAGTTTC--GTTTCAAGCct 3'</td><td>-10.90</td><td>140.00</td></tr>
<tr><td>54</td><td>hsa-miR-1274a</td><td>1580~1599</td><td>20</td><td>miRNA: 3' accgCGGACU--UGUCCCUg 5'
    ||| |: |||||||
Target:5' agtgGCCAGGTCACAGGGAt 3'</td><td>-18.90</td><td>148.00</td><td>?</td></tr>
<tr><td>55</td><td>hsa-miR-1274b</td><td>1345~1361</td><td>17</td><td>miRNA: 3' accgcgggcUUGUCCCu 5'
         |||||||
Target:5' atctttgtaAACAGGGc 3'</td><td>-10.00</td><td>140.00</td><td>?</td></tr>
<tr><td>56</td><td>hsa-miR-1275</td><td>929~945</td><td>17</td><td>miRNA: 3' cugucggagAGGGGGUg 5'
         |||||||
Target:5' ttgggtaagTCCCCCAg 3'</td><td>-12.90</td><td>140.00</td><td>?</td></tr>
<tr><td>57</td><td>hsa-miR-1281</td><td>1215~1234</td><td>20</td><td>miRNA: 3' ccCUCUC---CUCCUCCGCu 5'
  || || |||||||||
Target:5' ttGAAAGTGAGAGGAGGCGg 3'</td><td>-20.50</td><td>155.00</td><td>?</td></tr>
<tr><td>58</td><td>hsa-miR-1282</td><td>1667~1686</td><td>20</td><td>miRNA: 3' uucgucuuuuCCGUUUGCu 5'
          ||||||||
Target:5' ctaccacccgtGGCAAACGc 3'</td><td>-10.90</td><td>145.00</td><td>?</td></tr>
<tr><td rowspan="2"></td><td rowspan="2"></td><td>2655~2674</td><td>20</td><td>miRNA: 3' ucUUUCGCGAAAGGAAACAUCu 5'
  :|| :| |||:|||||||
Target:5' acGAAATG--TTCTTTTGTAGt 3'</td><td>-14.20</td><td>160.00</td><td rowspan="2"></td></tr>
<tr><td></td><td></td><td></td><td></td><td></td></tr>
</table>

<table>
<tr><td rowspan="5">59</td><td rowspan="5">hsa-miR-1283</td><td>2475~2500</td><td>26</td><td>miRNA: 3' ucuuUCG-CGAAA---GGAAACAUCu 5'
||| | ||| : |||||||
Target:5' attcAGCTGGTTTGAATATTTGTAGc 3'</td><td>-8.00</td><td>150.00</td><td rowspan="5"></td></tr>
<tr><td>2580~2601</td><td>22</td><td>miRNA: 3' ucUUUCGCGAAAGGAAACAUCu 5'
|| | | | :|||||||
Target:5' gaAATTCAATATATTTTGTAGt 3'</td><td>-7.10</td><td>148.00</td></tr>
<tr><td>2655~2674</td><td>20</td><td>miRNA: 3' ucUUUCGCGAAAGGAAACAUCu 5'
:|| :| |||:|||||||
Target:5' acGAAATG--TTCTTTTGTAGt 3'</td><td>-14.20</td><td>160.00</td></tr>
<tr><td>2475~2500</td><td>26</td><td>miRNA: 3' ucuuUCG-CGAAA---GGAAACAUCu 5'
||| | ||| : |||||||
Target:5' attcAGCTGGTTTGAATATTTGTAGc 3'</td><td>-8.00</td><td>150.00</td></tr>
<tr><td>2580~2601</td><td>22</td><td>miRNA: 3' ucUUUCGCGAAAGGAAACAUCu 5'
|| | | | :|||||||
Target:5' gaAATTCAATATATTTTGTAGt 3'</td><td>-7.10</td><td>148.00</td></tr>
<tr><td>60</td><td>hsa-miR-1286</td><td>1102~1122</td><td>21</td><td>miRNA: 3' ucccgaGUAGAACCAGGACGu 5'
||||| |||:||||
Target:5' ggtcagCATCTCGGTTCTGCg 3'</td><td>-21.60</td><td>151.00</td><td></td></tr>
<tr><td>61</td><td>hsa-miR-1288</td><td>2205~2224</td><td>20</td><td>miRNA: 3' agAGGUCUAGUCCCGUCAGGu 5'
| |||: |||||:||||
Target:5' aaTGCAGG-GAGGGCGGTCCa 3'</td><td>-26.00</td><td>146.00</td><td></td></tr>
<tr><td rowspan="5">62</td><td rowspan="5">hsa-miR-1289</td><td>2249~2274</td><td>26</td><td>miRNA: 3' uuuuACGUCUAAGGA---CCUGAGGu 5'
| |||:| | | |||||||
Target:5' acccTCCAGGTCCGTGTGGGACTCCa 3'</td><td>-20.90</td><td>155.00</td><td rowspan="5"></td></tr>
<tr><td>1519~1544</td><td>26</td><td>miRNA: 3' uuuuACGUCU-AAGGA--CCUGAGGu 5'
|| :|: | ||| |||||||
Target:5' gggcTGGGGGCTGCCTGAGGACTCCt 3'</td><td>-17.50</td><td>153.00</td></tr>
<tr><td>281~314</td><td>34</td><td>miRNA: 3' uuUUACGU-CUAAGG----------ACCUGAGGu 5'
|:|||: ||||| ||||||
Target:5' ggAGTGCGCCATTCCAGGAAAGAAAGGGACTCCg 3'</td><td>-21.70</td><td>147.00</td></tr>
<tr><td>2249~2274</td><td>26</td><td>miRNA: 3' uuuuACGUCUAAGGA---CCUGAGGu 5'
| |||:| | | |||||||
Target:5' acccTCCAGGTCCGTGTGGGACTCCa 3'</td><td>-20.90</td><td>155.00</td></tr>
<tr><td>1519~1544</td><td>26</td><td>miRNA: 3' uuuuACGUCU-AAGGA--CCUGAGGu 5'
[illegible]</td><td>-17.50</td><td>153.00</td></tr>
</table>

<table>
<tr><td></td><td></td><td></td><td></td><td>|| :|: | ||| |||||||
Target:5' gggcTGGGGGCTGCCTGAGGACTCCt 3'</td><td></td><td></td><td></td></tr>
<tr><td></td><td></td><td>281~314</td><td>34</td><td>miRNA: 3' uuUUACGU-CUAAGG----------ACCUGAGGu 5'
|:|||: ||||| |||||||
Target:5' ggAGTGCGCCATTCCAGGAAAGAAAGGGACTCCg 3'</td><td>-21.70</td><td>147.00</td><td></td></tr>
<tr><td>63</td><td>hsa-miR-1290</td><td>2819~2838</td><td>20</td><td>miRNA: 3' aggGAC-UAGGUUUUUAGGu 5'
||| |||:||:|||||
Target:5' ccaCTGTATCTAAGAATCCa 3'</td><td>-19.60</td><td>152.00</td><td></td></tr>
<tr><td></td><td></td><td>232~254</td><td>23</td><td>miRNA: 3' aggGACUAGG-----UUUUUAGGu 5'
||| ||| ||||||||
Target:5' tggCTG-TCCCAACAAAAAATCCc 3'</td><td>-10.90</td><td>151.00</td><td></td></tr>
<tr><td>64</td><td>hsa-miR-1291</td><td>803~826</td><td>24</td><td>miRNA: 3' ugACGACCAGAAGUCAGUCCCGGu 5'
||| ||:| || |||||:|
Target:5' atTGCCCGTTTCCATACAGGGTCt 3'</td><td>-18.10</td><td>150.00</td><td></td></tr>
<tr><td></td><td></td><td>2205~2230</td><td>26</td><td>miRNA: 3' ugACG-ACCAGAAGUCA-GUCCCGGu 5'
||| || |:|| |||||||
Target:5' aaTGCAGGGAGGGCGGTCCAGGGCCc 3'</td><td>-22.90</td><td>150.00</td><td></td></tr>
<tr><td></td><td></td><td>1337~1363</td><td>27</td><td>miRNA: 3' ugacGACC-AGAAGUCA---GUCCCGGu 5'
|||| ||||: || ||||||:
Target:5' tgccCTGGATCTTT-GTAAACAGGGCTg 3'</td><td>-19.50</td><td>147.00</td><td></td></tr>
<tr><td></td><td></td><td>2440~2464</td><td>25</td><td>miRNA: 3' ugaCGACCAGA-AGUCAGUCCCGGu 5'
|:| ||:| | | |:||||
Target:5' gtgGTTTGTTTATAACACGGGGCCt 3'</td><td>-16.46</td><td>141.00</td><td></td></tr>
<tr><td></td><td></td><td>1502~1524</td><td>23</td><td>miRNA: 3' ugacGACCAGAAGUC-AGUCCCGGu 5'
| |||| ||| ||||||:
Target:5' aggcCAGGTC--CAGACCAGGGCTg 3'</td><td>-18.30</td><td>140.00</td><td></td></tr>
<tr><td>65</td><td>hsa-miR-1292</td><td>789~816</td><td>28</td><td>miRNA: 3' gucGC-AGACGGCCU--UGGGCAAGGGu 5'
|| |: ||:| | :||||||:||
Target:5' tctCGATTCGCTGAATTGCCCGTTTCCa 3'</td><td>-24.30</td><td>148.00</td><td></td></tr>
<tr><td>66</td><td>hsa-miR-1293</td><td>2329~2350</td><td>22</td><td>miRNA: 3' cgUGUUUAGAGGUCUGGUGGGu 5'
:||: | || |||||||
Target:5' cgGCAGTGCCCCCCACCACCCc 3'</td><td>-16.70</td><td>152.00</td><td></td></tr>
<tr><td></td><td></td><td>200~221</td><td>22</td><td>miRNA: 3' cguguuuagaggUCUGGUGGGu 5'
|||||||||
Target:5' aggatggaggaaAGACCACCCa 3'</td><td>-23.50</td><td>150.00</td><td></td></tr>
</table>

<table>
<tr><td></td><td></td><td></td><td></td><td>Target:5' [illegible] 3'</td><td></td><td></td><td></td></tr>
<tr><td></td><td></td><td>1655~1676</td><td>22</td><td>miRNA: 3' cguguUUAGAGGUCUGGUGGGu 5'
|: ||: |||||||
Target:5' gaggcAGCCTTGCTACCACCCg 3'</td><td>-16.10</td><td>145.00</td><td></td></tr>
<tr><td>67</td><td>hsa-miR-1294</td><td>256~275</td><td>20</td><td>miRNA: 3' ucuguUGUUACGGUUGGAGUGu 5'
||:||| |||||||:
Target:5' atggcACGATG--AACCTCATg 3'</td><td>-16.10</td><td>145.00</td><td></td></tr>
<tr><td>68</td><td>hsa-miR-1295</td><td>2673~2693</td><td>21</td><td>miRNA: 3' agUGGGUCUAGACGCCGGAUu 5'
||| |:|| |||||||
Target:5' gtACCACGGTCCTCGGCCTAa 3'</td><td>-22.80</td><td>159.00</td><td></td></tr>
<tr><td rowspan="3">69</td><td rowspan="3">hsa-miR-1296</td><td>2210~2233</td><td>24</td><td>miRNA: 3' ccUCUACCUC--GGUCCCGGGAUu 5'
:|| || | ||||||||||:
Target:5' agGGAGGGCGGTCCAGGGCCCTGg 3'</td><td>-32.60</td><td>154.00</td><td rowspan="3"></td></tr>
<tr><td>2092~2111</td><td>20</td><td>miRNA: 3' ccUCUACCUCGGUCCCGGGAUu 5'
:| ||||||: ||||||:
Target:5' ggGGCTGGAGCT--GGCCCTGc 3'</td><td>-26.20</td><td>148.00</td></tr>
<tr><td>1749~1766</td><td>18</td><td>miRNA: 3' ccUCUACCUCGGUCCCGGGAUu 5'
||:| || ||||||||:
Target:5' ccAGGT--AG--AGGGCCCTGg 3'</td><td>-19.00</td><td>140.00</td></tr>
<tr><td>70</td><td>hsa-miR-1299</td><td>1045~1067</td><td>23</td><td>miRNA: 3' agGGAGUGUGUCUU--AAGGUCUu 5'
|||| | :|||| |||:|||
Target:5' ctCCTCTC-TAGAACCTTCTAGAa 3'</td><td>-16.90</td><td>149.00</td><td></td></tr>
<tr><td rowspan="2">71</td><td rowspan="2">hsa-miR-1301</td><td>969~1000</td><td>32</td><td>miRNA: 3' cuUCAGU-----GAG-GGUCCG--UCGACGUu 5'
|| || ||| ||: || |||||||
Target:5' gcAGGCATGCTTCTCACCGTGCAGAGCTGCAc 3'</td><td>-17.50</td><td>156.00</td><td rowspan="2"></td></tr>
<tr><td>1586~1609</td><td>24</td><td>miRNA: 3' cuUCAGUGAGGGUCCGUCGACGUu 5'
:||||| | | ||||||:
Target:5' caGGTCACAGGGATGGAGCTGCGc 3'</td><td>-18.60</td><td>142.00</td></tr>
<tr><td rowspan="2"></td><td rowspan="2"></td><td>222~243</td><td>22</td><td>miRNA: 3' aaAUCGUAUUCAU--ACAGGGUu 5'
|:|: | ||: |||||||
Target:5' ttTGGT-TTCGTGGCTGTCCCAa 3'</td><td>-13.70</td><td>144.00</td><td rowspan="2"></td></tr>
<tr><td>222~243</td><td>22</td><td>miRNA: 3' aaAUCGUAUUCAU--ACAGGGUu 5'
|:|: | ||: |||||||
Target:5' ttTGGT-TTCGTGGCTGTCCCAa 3'</td><td>-13.70</td><td>144.00</td></tr>
</table>

72	hsa-miR-1302	222~243	22	miRNA: 3' aaAUCGUAUUCAU--ACAGGGUu 5'<br>|:|: | ||: ||||||| Target:5' ttTGGT-TTCGTGGCTGTCCCAa 3'	-13.70	144.00	
		222~243	22	miRNA: 3' aaAUCGUAUUCAU--ACAGGGUu 5'<br>|:|: | ||: ||||||| Target:5' ttTGGT-TTCGTGGCTGTCCCAa 3'	-13.70	144.00	
		222~243	22	miRNA: 3' aaAUCGUAUUCAU--ACAGGGUu 5'<br>|:|: | ||: ||||||| Target:5' ttTGGT-TTCGTGGCTGTCCCAa 3'	-13.70	144.00	
		222~243	22	miRNA: 3' aaAUCGUAUUCAU--ACAGGGUu 5'<br>|:|: | ||: ||||||| Target:5' ttTGGT-TTCGTGGCTGTCCCAa 3'	-13.70	144.00	
		222~243	22	miRNA: 3' aaAUCGUAUUCAU--ACAGGGUu 5'<br>|:|: | ||: ||||||| Target:5' ttTGGT-TTCGTGGCTGTCCCAa 3'	-13.70	144.00	
		222~243	22	miRNA: 3' aaAUCGUAUUCAU--ACAGGGUu 5'<br>|:|: | ||: ||||||| Target:5' ttTGGT-TTCGTGGCTGTCCCAa 3'	-13.70	144.00	
		222~243	22	miRNA: 3' aaAUCGUAUUCAU--ACAGGGUu 5'<br>|:|: | ||: ||||||| Target:5' ttTGGT-TTCGTGGCTGTCCCAa 3'	-13.70	144.00	
		222~243	22	miRNA: 3' aaAUCGUAUUCAU--ACAGGGUu 5'<br>|:|: | ||: ||||||| Target:5' ttTGGT-TTCGTGGCTGTCCCAa 3'	-13.70	144.00	
		222~243	22	miRNA: 3' aaAUCGUAUUCAU--ACAGGGUu 5'<br>|:|: | ||: ||||||| Target:5' ttTGGT-TTCGTGGCTGTCCCAa 3'	-13.70	144.00	
73	hsa-miR-1304	990~1011	22	miRNA: 3' gugUAGA-GUGACAUCGGAGUUu 5'<br>| || |||| |:||||||:<br>Target:5' cagAGCTGCACT-TGGCCTCAGc 3'	-16.70	146.00	
74	hsa-miR-1307	110~132	23	miRNA: 3' guGCUGGC--UGCGGUGCGGCUCa 5'<br>:| ||| ||||: |||||:| Target:5' ccTGTCCGCTACGCT-CGCCGGGg 3'	-24.10	145.00	

<table>
<tr><td></td><td></td><td>1839~1860</td><td>22</td><td>miRNA: 3' guGCUGGCUGCGGUGCGGCUCa 5'
| :||| | : ||||:|||
Target:5' agCAGCCGTCCTGACGCTGAGc 3'</td><td>-19.30</td><td>144.00</td><td></td></tr>
<tr><td>75</td><td>hsa-miR-130b*</td><td>281~305</td><td>25</td><td>miRNA: 3' caUCACGUUGU----CCCUUUCUca 5'
|||||: || |||||||
Target:5' ggAGTGCGCCATTCCAGGAAAGAaa 3'</td><td>-13.90</td><td>141.00</td><td>?</td></tr>
<tr><td>76</td><td>hsa-miR-1321</td><td>1020~1038</td><td>19</td><td>miRNA: 3' uagUGUAAGU-GGAGGGAc 5'
| || || |||||||
Target:5' tggAAATGCACCCTCCCTc 3'</td><td>-13.00</td><td>151.00</td><td>?</td></tr>
<tr><td>77</td><td>hsa-miR-1324</td><td>1134~1157</td><td>24</td><td>miRNA: 3' cuuucACGUAUCUUAAGACAGACc 5'
| :||:| :||:||||||
Target:5' gtgttTATATGGCGTTTTGTCTGt 3'</td><td>-14.20</td><td>151.00</td><td>?</td></tr>
<tr><td rowspan="3">78</td><td rowspan="3">hsa-miR-134</td><td>2384~2412</td><td>29</td><td>miRNA: 3' ggGGAGACCAGU---UG----GUCAGUGu 5'
::|| || :| || |||||||
Target:5' ctTTTCCGGCTACGCACCCTGCAGTCACt 3'</td><td>-15.00</td><td>146.00</td><td rowspan="3">?</td></tr>
<tr><td>2720~2740</td><td>21</td><td>miRNA: 3' gggGAGACCAGUUG-GUCAGUGu 5'
||||| ||| |:|||||
Target:5' gagCTCTG--AAACGCGGTCACc 3'</td><td>-18.10</td><td>143.00</td></tr>
<tr><td>2022~2047</td><td>26</td><td>miRNA: 3' ggGGAGACC-A--GUU-GGUCAGUGu 5'
||||||| | ||| |||||| |
Target:5' aaCCTCTGGCTCCCAACCCAGTCTCc 3'</td><td>-23.00</td><td>142.00</td></tr>
<tr><td>79</td><td>hsa-miR-135b*</td><td>2377~2407</td><td>31</td><td>miRNA: 3' ggguACCGAAAA--UC-------GGGAUGUa 5'
|||||||| :| ||||:||
Target:5' ttgtTGGCTTTTCCGGCTACGCACCCTGCAg 3'</td><td>-18.80</td><td>140.00</td><td>?</td></tr>
<tr><td>80</td><td>hsa-miR-136</td><td>565~588</td><td>24</td><td>miRNA: 3' agGUAGUAGUUUUGU-UUACCUCa 5'
:| :: ||||||| |:|||||
Target:5' ttTACTGCCAAAACAGAGTGGAGt 3'</td><td>-16.00</td><td>153.00</td><td>?</td></tr>
<tr><td rowspan="2">81</td><td rowspan="2">hsa-miR-140-5p</td><td>2798~2824</td><td>27</td><td>miRNA: 3' gaUGGUAUCCCAUU------UUGGUGAc 5'
|:::| ||| || |||||||
Target:5' gaATTGT-GGGCAATGGATTAACCACTg 3'</td><td>-14.70</td><td>149.00</td><td rowspan="2">?</td></tr>
<tr><td>372~398</td><td>27</td><td>miRNA: 3' gaUGGUAUCCCAUU-----UUGGUGac 5'
|||| | |||| ||||||
Target:5' ccACCAAAATGTAAATTCGAACCACca 3'</td><td>-10.40</td><td>140.00</td></tr>
<tr><td>82</td><td>hsa-miR-[illegible]</td><td>410~437</td><td>28</td><td>miRNA: 3' ggUAGAAAUGG------UCUGUCACAau 5'
|| | |||| :||||||||</td><td>-12.50</td><td>142.00</td><td>?</td></tr>
</table>

<table>
<tr><td></td><td>141</td><td></td><td></td><td>Target:5' gaATGTGTACCCTTCGGGGACAGTGTgc 3'</td><td></td><td></td><td></td></tr>
<tr><td rowspan="3">83</td><td rowspan="3">hsa-miR-143*</td><td>1453~1476</td><td>24</td><td>miRNA: 3' ugguCUCUACG-UCG-UGACGUGg 5'
|||| | :|| |||||||
Target:5' catcGAGACCCTGGCAACTGCACc 3'</td><td>-20.30</td><td>154.00</td><td rowspan="3"></td></tr>
<tr><td>980~1001</td><td>22</td><td>miRNA: 3' uggucucUACGUCGUGACGUGg 5'
:||||| :|||||
Target:5' tctcaccGTGCAGAGCTGCACt 3'</td><td>-20.00</td><td>147.00</td></tr>
<tr><td>1890~1915</td><td>26</td><td>miRNA: 3' ugGUCUCUACG---UC-GUGACGUgg 5'
::|:| ||| || |||||||
Target:5' ctTGGGGCTGCCTGAGCCACTGCAgg 3'</td><td>-13.90</td><td>140.00</td></tr>
<tr><td rowspan="2">84</td><td rowspan="2">hsa-miR-145*</td><td>485~507</td><td>23</td><td>miRNA: 3' ucUUGUCAUA--AAGGUCCUUAgg 5'
||||| || | ::||||||
Target:5' caAACAG-ATCCTATTAGGAATac 3'</td><td>-10.60</td><td>141.00</td><td rowspan="2"></td></tr>
<tr><td>2272~2295</td><td>24</td><td>miRNA: 3' ucUUGUC-AUAA-AGGUCCUUAGg 5'
|:| | |:|| |::||||||
Target:5' ccAGCCGCTGTTGGCTGGGAATCg 3'</td><td>-13.40</td><td>140.00</td></tr>
<tr><td rowspan="2">85</td><td rowspan="2">hsa-miR-146b-3p</td><td>1341~1362</td><td>22</td><td>miRNA: 3' ggUCUUGACUCAGGUGUCCCGu 5'
:|| || || |||||||
Target:5' ctGGATCTTTGTAAACAGGGCt 3'</td><td>-19.30</td><td>156.00</td><td rowspan="2"></td></tr>
<tr><td>2155~2183</td><td>29</td><td>miRNA: 3' ggUCUUGACUCAGG-------UGUCCCGu 5'
|| :| |:|||| :|||||
Target:5' caAGTGCAGGGTCCCAAGAGGGCAGGGCg 3'</td><td>-20.31</td><td>140.00</td></tr>
<tr><td>86</td><td>hsa-miR-147</td><td>802~821</td><td>20</td><td>miRNA: 3' cgucuucGUAAAGGUGUGUg 5'
|:||||||:|||
Target:5' aattgcccGTTTCCATACAg 3'</td><td>-15.00</td><td>145.00</td><td></td></tr>
<tr><td>87</td><td>hsa-miR-1470</td><td>35~58</td><td>24</td><td>miRNA: 3' gccccaCGUGCCC---GCCUCCCg 5'
| :|||| |||||||
Target:5' cgcgcgGAGCGGGCTCCGGAGGGa 3'</td><td>-27.40</td><td>151.00</td><td></td></tr>
<tr><td>88</td><td>hsa-miR-148a*</td><td>663~688</td><td>26</td><td>miRNA: 3' ucagCCUCA-CAGA--G-UCUUGAAa 5'
||| | ||:| | |||||||
Target:5' gaagGGATTGGTTTGGCAAGAACTTg 3'</td><td>-11.40</td><td>152.00</td><td></td></tr>
<tr><td></td><td></td><td>1237~1257</td><td>21</td><td>miRNA: 3' cguGUCGGGGGCAGGGAGGGa 5'
|||: |||| |||||||
Target:5' tccCAGTGCCCGGCCCTCCCc 3'</td><td>-30.40</td><td>170.00</td><td></td></tr>
</table>

<table>
<tr><td rowspan="3">89</td><td rowspan="3">hsa-miR-149*</td><td>1702~1728</td><td>27</td><td>miRNA: 3' cgUGUCGGG----GGCA--GGGAGGga 5'
            | ||||| |||| ||||||
Target:5' ggAGAGCCCACAGCCGTGGCCCTCCag 3'</td><td>-26.50</td><td>143.00</td><td rowspan="3"></td></tr>
<tr><td>1022~1041</td><td>20</td><td>miRNA: 3' cguguCGGGGGCAGGGAGGGa 5'
               || ||| |||||||:
Target:5' gaaatGCACCC-TCCCTCCTg 3'</td><td>-19.80</td><td>143.00</td></tr>
<tr><td>2003~2023</td><td>21</td><td>miRNA: 3' cguguCGGGGGCAGGGAGGga 5'
               ||:||| ||||||
Target:5' ccggtGCTCCCAGCCCTCCaa 3'</td><td>-25.30</td><td>140.00</td></tr>
<tr><td>90</td><td>hsa-miR-151-5p</td><td>940~960</td><td>21</td><td>miRNA: 3' ugaucugaCACUCGAGGAGCu 5'
                  | |||:||||||
Target:5' ccccagggGCGAGTTCCTCGc 3'</td><td>-19.10</td><td>141.00</td><td></td></tr>
<tr><td>91</td><td>hsa-miR-152</td><td>1324~1344</td><td>21</td><td>miRNA: 3' ggUUCAAGACAGUACGUGACu 5'
            |:| |||||| ||| |||
Target:5' tgAGGCTCTGTCTTGCCCTGg 3'</td><td>-17.20</td><td>143.00</td><td></td></tr>
<tr><td rowspan="2">92</td><td rowspan="2">hsa-miR-15a</td><td>1062~1084</td><td>23</td><td>miRNA: 3' gugUUUGGUAAU--ACACGACGAu 5'
             :|||| | : |||||||||
Target:5' ctaGAACC-TGGGCTGTGCTGCTt 3'</td><td>-17.50</td><td>156.00</td><td rowspan="2"></td></tr>
<tr><td>1354~1380</td><td>27</td><td>miRNA: 3' guGU-UUGGUA-A---UACACGACGAu 5'
            || ::|::| | | ||||||||
Target:5' aaCAGGGCTGTGTACAAAGTGCTGCTg 3'</td><td>-15.50</td><td>148.00</td></tr>
<tr><td>93</td><td>hsa-miR-15a*</td><td>1902~1925</td><td>24</td><td>miRNA: 3' acUCCGU--CGUGUUAUACCGGAc 5'
            || || ||| :| :||||||
Target:5' tgAGCCACTGCAGGAAGTGGCCTg 3'</td><td>-21.10</td><td>146.00</td><td></td></tr>
<tr><td rowspan="2">94</td><td rowspan="2">hsa-miR-15b</td><td>1061~1084</td><td>24</td><td>miRNA: 3' acAUUUGGUACU--ACACGACGAu 5'
            ||:| | ||: |||||||||
Target:5' tcTAGAACCTGGGCTGTGCTGCTt 3'</td><td>-19.60</td><td>166.00</td><td rowspan="2"></td></tr>
<tr><td>1350~1380</td><td>31</td><td>miRNA: 3' acAUUU-----GGUAC----UACACGACGAu 5'
            |||| |::|| | ||||||||
Target:5' tgTAAACAGGGCTGTGTACAAAGTGCTGCTg 3'</td><td>-17.90</td><td>154.00</td></tr>
<tr><td></td><td></td><td>1356~1380</td><td>25</td><td>miRNA: 3' gcgguuAUAAAUG---CACGACGAu 5'
                |:| ||| ||||||||
Target:5' cagggcTGTGTACAAAGTGCTGCTg 3'</td><td>-14.60</td><td>156.00</td><td></td></tr>
</table>

<table>
<tr><td rowspan="3">95</td><td rowspan="3">hsa-miR-16</td><td>1060~1084</td><td>25</td><td>miRNA: 3' gcGGUUAUAAAU---GCACGACGAu 5'
|:|: | |:   :||||||||
Target:5' ttCTAGAACCTGGGCTGTGCTGCTt 3'</td><td>-14.60</td><td>148.00</td><td rowspan="3"></td></tr>
<tr><td>1356~1380</td><td>25</td><td>miRNA: 3' gcgguuAUAAAUG---CACGACGAu 5'
|:| ||| ||||||||
Target:5' cagggcTGTGTACAAAGTGCTGCTg 3'</td><td>-14.60</td><td>156.00</td></tr>
<tr><td>1060~1084</td><td>25</td><td>miRNA: 3' gcGGUUAUAAAU---GCACGACGAu 5'
|:|: | |:   :||||||||
Target:5' ttCTAGAACCTGGGCTGTGCTGCTt 3'</td><td>-14.60</td><td>148.00</td></tr>
<tr><td>96</td><td>hsa-miR-17*</td><td>1891~1915</td><td>25</td><td>miRNA: 3' gauguucACGGA---AGUGACGUCa 5'
||||| ||||||||
Target:5' ttggggcTGCCTGAGCCACTGCAGg 3'</td><td>-19.90</td><td>155.00</td><td></td></tr>
<tr><td rowspan="2">97</td><td rowspan="2">hsa-miR-181a-2*</td><td>2729~2753</td><td>25</td><td>miRNA: 3' ccaUGUCAGUUG---CCAGUCACCa 5'
:|:|||| | | |:|||||
Target:5' aacGCGGTCACCTTTGTTTAGTGGa 3'</td><td>-16.60</td><td>143.00</td><td rowspan="2"></td></tr>
<tr><td>1564~1585</td><td>22</td><td>miRNA: 3' ccaugucaguugccAGUCACCa 5'
|||||||
Target:5' gggggttcccctctTCAGTGGc 3'</td><td>-14.60</td><td>140.00</td></tr>
<tr><td>98</td><td>hsa-miR-182*</td><td>1040~1060</td><td>21</td><td>miRNA: 3' aucaaccguucAGAUCUUGGu 5'
|||||||||
Target:5' tgccgctcctcTCTAGAACCt 3'</td><td>-11.40</td><td>150.00</td><td></td></tr>
<tr><td>99</td><td>hsa-miR-183</td><td>1464~1485</td><td>22</td><td>miRNA: 3' ucacUUAAGAUGGUCACGGUau 5'
|| | |||:||||||
Target:5' tggcAACTGCACCGGTGCCAgc 3'</td><td>-16.10</td><td>142.00</td><td></td></tr>
<tr><td rowspan="2">100</td><td rowspan="2">hsa-miR-184</td><td>1769~1794</td><td>26</td><td>miRNA: 3' ugGGA-AUAG---UCAAGAGGCAGGu 5'
||| |:|| | ||||:|||||
Target:5' agCCTGTGTCTGGAATTCTTCGTCCt 3'</td><td>-20.50</td><td>152.00</td><td rowspan="2"></td></tr>
<tr><td>1621~1642</td><td>22</td><td>miRNA: 3' ugGGAAUAGUCAAGAGGCAGGu 5'
|| |||| | ||:||||
Target:5' tgCCACCTCAGGTGTCTGTCCc 3'</td><td>-16.90</td><td>144.00</td></tr>
<tr><td rowspan="2">101</td><td rowspan="2">hsa-miR-185*</td><td>1987~2006</td><td>20</td><td>miRNA: 3' cuGGUCUCCUUUCGGUCGGGga 5'
|::|:||| | ||||||
Target:5' ccCTGGGGGACA--CAGCCCgg 3'</td><td>-20.90</td><td>140.00</td><td rowspan="2"></td></tr>
<tr><td>1253~1274</td><td>22</td><td>miRNA: 3' cuggucuccuuucgGUCGGGGa 5'
|||||||</td><td>-13.64</td><td>140.00</td></tr>
</table>

<table>
<tr><td></td><td></td><td></td><td></td><td>Target:5' tccccacccacctgCAGCCCCa 3'</td><td></td><td></td><td></td></tr>
<tr><td>102</td><td>hsa-miR-187*</td><td>2478~2501</td><td>24</td><td>miRNA: 3' cggGCCCAGG---ACACAACAUCGg 5'
| |||:: | | |||||||
Target:5' cagCTGGTTTGAATAT-TTGTAGCc 3'</td><td>-15.20</td><td>146.00</td><td></td></tr>
<tr><td>103</td><td>hsa-miR-188-5p</td><td>649~671</td><td>23</td><td>miRNA: 3' ggGAGGUGG--UACGUUCCCUAc 5'
| :|::| | | |||||||
Target:5' ggCATCGTCAAAAGGAAGGGATt 3'</td><td>-14.10</td><td>149.00</td><td></td></tr>
<tr><td>104</td><td>hsa-miR-188-3p</td><td>2244~2270</td><td>27</td><td>miRNA: 3' acguuUGGGACGU------ACACCCUc 5'
||||| || |||||||
Target:5' tcggcACCCTCCAGGTCCGTGTGGGAc 3'</td><td>-18.90</td><td>154.00</td><td></td></tr>
<tr><td>105</td><td>hsa-miR-18a*</td><td>2157~2180</td><td>24</td><td>miRNA: 3' gguCUUCCUCGUG-AAUCCCGUCa 5'
| |||: | | |||||||
Target:5' agtGCAGGGTCCCAAGAGGGCAGg 3'</td><td>-17.20</td><td>148.00</td><td></td></tr>
<tr><td>106</td><td>hsa-miR-1908</td><td>81~100</td><td>20</td><td>miRNA: 3' cugguuaGCGGCAGGGGCGGc 5'
||||| ||||||:
Target:5' ccgccgcCGCCG-CCCCGCTc 3'</td><td>-27.30</td><td>141.00</td><td></td></tr>
<tr><td>107</td><td>hsa-miR-1909</td><td>1021~1043</td><td>23</td><td>miRNA: 3' gccacuCGUGGG-CCGGGGACGc 5'
|||||| |:||||
Target:5' ggaaatGCACCCTCCCTCCTGCc 3'</td><td>-20.80</td><td>140.00</td><td></td></tr>
<tr><td>108</td><td>hsa-miR-190b</td><td>140~164</td><td>25</td><td>miRNA: 3' uuGGGUUAU----AGUUUGUAUAGu 5'
|||:| : |::||||:||
Target:5' cgCCCGAGGGACTTTGAACATGTCg 3'</td><td>-15.50</td><td>141.00</td><td></td></tr>
<tr><td>109</td><td>hsa-miR-191*</td><td>2143~2164</td><td>22</td><td>miRNA: 3' ccccuGCUUUAGGUUCGCGUCg 5'
|| :: |||||:||||
Target:5' gtcccCGTGGCCCAAGTGCAGg 3'</td><td>-18.90</td><td>145.00</td><td></td></tr>
<tr><td>110</td><td>hsa-miR-1910</td><td>448~467</td><td>20</td><td>miRNA: 3' ucCGCCGUCCGUGUCCUGACc 5'
| || :| || |||||||
Target:5' taGAGGAGGACA-AGGACTGg 3'</td><td>-21.80</td><td>154.00</td><td></td></tr>
<tr><td>111</td><td>hsa-miR-1911*</td><td>1492~1511</td><td>20</td><td>miRNA: 3' ccUCUGGUGUUACGGACCAc 5'
:|:|||||| ||| |||
Target:5' ggGGGCCACAAGGCCAGGTc 3'</td><td>-24.10</td><td>142.00</td><td></td></tr>
<tr><td>[illegible]</td><td>hsa-[illegible]</td><td>1599~1620</td><td>22</td><td>miRNA: 3' guCUUCACCCGGCCCGUGUCCc 5'
||: || ||:| |||:|||
Target:5' tgGAGCTGCGCTGTGCATAGGg 3'</td><td>-23.40</td><td>152.00</td><td></td></tr>
</table>

<table>
<tr><td>112</td><td>miR-1914</td><td>1576~1597</td><td>22</td><td>miRNA: 3' gucuUCACCCGGCCCGUGUCCc 5'
||||| | || ||||||
Target:5' cttcAGTGGCCAGGTCACAGGg 3'</td><td>-25.80</td><td>142.00</td><td>?</td></tr>
<tr><td>113</td><td>hsa-miR-1914*</td><td>2327~2353</td><td>27</td><td>miRNA: 3' ggaggGUCAC------GCCCUGGGGAGg 5'
||||| | |||||||
Target:5' gccggCAGTGCCCCCCACCACCCCTCc 3'</td><td>-24.60</td><td>145.00</td><td>?</td></tr>
<tr><td rowspan="6">114</td><td rowspan="6">hsa-miR-1915</td><td>1745~1768</td><td>24</td><td>miRNA: 3' ggGCGGCGCA----GCGGGACCCc 5'
|||| || |||||||
Target:5' agCGCCAGGTAGAGGGCCCTGGGc 3'</td><td>-23.10</td><td>152.00</td><td rowspan="6">?</td></tr>
<tr><td>2213~2235</td><td>23</td><td>miRNA: 3' gggCGGCGCAG----CGGGACCCc 5'
| || ||| |||||||
Target:5' gagGGCG-GTCCAGGGCCCTGGGa 3'</td><td>-21.20</td><td>150.00</td></tr>
<tr><td>1975~1994</td><td>20</td><td>miRNA: 3' gggCGGCGCAGCGGGACCCc 5'
| :| | ||||||||
Target:5' agaGATGGGAAGCCCTGGGg 3'</td><td>-23.60</td><td>149.00</td></tr>
<tr><td>1327~1346</td><td>20</td><td>miRNA: 3' ggGCGGCGCAGCGGGACCcc 5'
| |:|: |:|||||||
Target:5' ggCTCTGTCTTGCCCTGGat 3'</td><td>-19.90</td><td>142.00</td></tr>
<tr><td>2102~2121</td><td>20</td><td>miRNA: 3' gggCGGCGCAGCGGGACCcc 5'
||| :| :|||||||
Target:5' ctgGCCCTGCTGCCCTGGca 3'</td><td>-22.20</td><td>141.00</td></tr>
<tr><td>948~967</td><td>20</td><td>miRNA: 3' gggcggcGCAGCGGGACCCc 5'
| ||||:|||||
Target:5' gcgagttCCTCGCTCTGGGa 3'</td><td>-19.70</td><td>141.00</td></tr>
<tr><td>115</td><td>hsa-miR-192*</td><td>1427~1455</td><td>29</td><td>miRNA: 3' gacaCUGGA--UA-----CCUUAACCGUc 5'
||:|| || ||:|||||||
Target:5' ctaaGATCTGCATAGGTCGGGATTGGCAt 3'</td><td>-16.40</td><td>156.00</td><td>?</td></tr>
<tr><td>116</td><td>hsa-miR-193a-5p</td><td>521~542</td><td>22</td><td>miRNA: 3' aguagagcgggcGUUUCUGGGu 5'
| |||||||
Target:5' tgaaccaaatatCCAAGACCCa 3'</td><td>-13.30</td><td>142.00</td><td>?</td></tr>
<tr><td>117</td><td>hsa-miR-193a-3p</td><td>457~476</td><td>20</td><td>miRNA: 3' ugacCCUGAAACAUCCGGUCaa 5'
|||| || |||||||
Target:5' acaaGGAC--TGGAGGCCAGcc 3'</td><td>-20.00</td><td>142.00</td><td>?</td></tr>
</table>

<table>
<tr><td rowspan="1">118</td><td rowspan="1">hsa-miR-193b*</td><td>1080~1101</td><td>22</td><td>miRNA: 3' aguAGAGC-GGGAGUUUUGGGGc 5'
|:|:| |||| |:|||||
Target:5' tgcTTTTGAGCCTC-AGACCCCa 3'</td><td>-19.50</td><td>142.00</td><td></td></tr>
<tr><td rowspan="3">119</td><td rowspan="3">hsa-miR-195</td><td>1064~1084</td><td>21</td><td>miRNA: 3' cgguuauaaaGACACGACGAu 5'
||||||||||
Target:5' agaacctgggCTGTGCTGCTt 3'</td><td>-18.40</td><td>155.00</td><td rowspan="3"></td></tr>
<tr><td>1144~1164</td><td>21</td><td>miRNA: 3' cgGUUAUAAAGACACGACGAu 5'
|: | | ||||||:||||
Target:5' ggCGTTTTGTCTGTGTTGCTg 3'</td><td>-18.60</td><td>151.00</td></tr>
<tr><td>1360~1380</td><td>21</td><td>miRNA: 3' cgguuauaaagaCACGACGAu 5'
||||||||
Target:5' gctgtgtacaaaGTGCTGCTg 3'</td><td>-13.50</td><td>145.00</td></tr>
<tr><td>120</td><td>hsa-miR-196a*</td><td>1140~1164</td><td>25</td><td>miRNA: 3' gaguCCGUCAA--AGA-ACAACGGc 5'
|||: || ||| ||||||:
Target:5' atatGGCGTTTTGTCTGTGTTGCTg 3'</td><td>-16.80</td><td>144.00</td><td></td></tr>
<tr><td>121</td><td>hsa-miR-196b*</td><td>219~241</td><td>23</td><td>miRNA: 3' cuuccGUCACAGCA-CGACAGcu 5'
::|| |||| ||||||
Target:5' ccattTGGTTTCGTGGCTGTccc 3'</td><td>-13.50</td><td>141.00</td><td></td></tr>
<tr><td rowspan="4">122</td><td rowspan="4">hsa-miR-1972</td><td>1902~1926</td><td>25</td><td>miRNA: 3' acUCGGUGAC--AC-GGACCGGACu 5'
|||||||| | |||||||
Target:5' tgAGCCACTGCAGGAAGTGGCCTGg 3'</td><td>-29.60</td><td>158.00</td><td rowspan="4"></td></tr>
<tr><td>1795~1817</td><td>23</td><td>miRNA: 3' acUCGGU-GACACGGACCGGACu 5'
:|||| ||| |: |||:|||
Target:5' gaGGCCACCTGAGTGTGGTCTGt 3'</td><td>-23.30</td><td>152.00</td></tr>
<tr><td>1902~1926</td><td>25</td><td>miRNA: 3' acUCGGUGAC--AC-GGACCGGACu 5'
|||||||| | |||||||
Target:5' tgAGCCACTGCAGGAAGTGGCCTGg 3'</td><td>-29.60</td><td>158.00</td></tr>
<tr><td>1795~1817</td><td>23</td><td>miRNA: 3' acUCGGU-GACACGGACCGGACu 5'
:|||| ||| |: |||:|||
Target:5' gaGGCCACCTGAGTGTGGTCTGt 3'</td><td>-23.30</td><td>152.00</td></tr>
<tr><td rowspan="2">123</td><td rowspan="2">hsa-miR-19a*</td><td>846~867</td><td>22</td><td>miRNA: 3' acaucacguUGAUACGUUUUGa 5'
::||||:|||||
Target:5' tttttgattGTTATGTAAAACt 3'</td><td>-10.80</td><td>141.00</td><td rowspan="2"></td></tr>
<tr><td>892~914</td><td>23</td><td>miRNA: 3' acAUCACGUUGAU-ACGUUUUGa 5'
[illegible]</td><td>-12.60</td><td>140.00</td></tr>
</table>

<table>
<tr><td></td><td></td><td></td><td></td><td>|| | |||||: ||:||||:
Target:5' agTATTTCAACTGCTGTAAAATt 3'</td><td></td><td></td><td></td></tr>
<tr><td>124</td><td>hsa-miR-19a</td><td>2834~2857</td><td>24</td><td>miRNA: 3' agucaaaacGUAUC-UAAACGUGu 5'
:| || ||||||||
Target:5' atccaccatTAAAGCATTTGCACa 3'</td><td>-10.30</td><td>150.00</td><td>?</td></tr>
<tr><td rowspan="2">125</td><td rowspan="2">hsa-miR-19b</td><td>2835~2857</td><td>23</td><td>miRNA: 3' agucaaaacgUACCUAAACGUGu 5'
| | ||||||||
Target:5' tccaccattaAAGCATTTGCACa 3'</td><td>-9.70</td><td>149.00</td><td rowspan="2">?</td></tr>
<tr><td>2835~2857</td><td>23</td><td>miRNA: 3' agucaaaacgUACCUAAACGUGu 5'
| | ||||||||
Target:5' tccaccattaAAGCATTTGCACa 3'</td><td>-9.70</td><td>149.00</td></tr>
<tr><td>126</td><td>hsa-miR-200b</td><td>875~898</td><td>24</td><td>miRNA: 3' aguAGUAAUGGU--CCGUCAUAAu 5'
|:| ||::: | |||||||
Target:5' attTTAATATTGATGTCAGTATTt 3'</td><td>-8.60</td><td>153.00</td><td>?</td></tr>
<tr><td>127</td><td>hsa-miR-200c</td><td>874~898</td><td>25</td><td>miRNA: 3' agguAGUAAUGG--GCCGUCAUAAu 5'
|:| ||:: :| |||||||
Target:5' tattTTAATATTGATGTCAGTATTt 3'</td><td>-10.40</td><td>153.00</td><td>?</td></tr>
<tr><td rowspan="2">128</td><td rowspan="2">hsa-miR-202*</td><td>1424~1444</td><td>21</td><td>miRNA: 3' guuucUUCAUAUACGUAUCCuu 5'
||| || ||||||||
Target:5' cagctAAG-ATCTGCATAGGtc 3'</td><td>-12.50</td><td>144.00</td><td rowspan="2">?</td></tr>
<tr><td>1596~1621</td><td>26</td><td>miRNA: 3' guUUCUUC-AU---AUACGUAUCCUu 5'
| |:|| |: |:||||||||:
Target:5' ggATGGAGCTGCGCTGTGCATAGGGt 3'</td><td>-15.10</td><td>144.00</td></tr>
<tr><td rowspan="2">129</td><td rowspan="2">hsa-miR-204</td><td>57~77</td><td>21</td><td>miRNA: 3' uccguaucCUACUGUUUCCCUu 5'
|| ||||||||||
Target:5' gaagtcccGA-GACAAAGGGAa 3'</td><td>-16.60</td><td>157.00</td><td rowspan="2">?</td></tr>
<tr><td>287~310</td><td>24</td><td>miRNA: 3' uccGUA---UCCUACUGUUUCCCUu 5'
||| |||| | |||||||
Target:5' cgcCATTCCAGGA-AAGAAAGGGAc 3'</td><td>-11.50</td><td>154.00</td></tr>
<tr><td>130</td><td>hsa-miR-20a*</td><td>2191~2212</td><td>22</td><td>miRNA: 3' gaaaUUCACGAGUAUUACGUCa 5'
|:| || | |||||||
Target:5' ccaaAGGAGCAAAGAATGCAGg 3'</td><td>-9.90</td><td>154.00</td><td>?</td></tr>
<tr><td></td><td>hsa-</td><td>319~342</td><td>24</td><td>miRNA: 3' gaCCUUC--ACGGGUAUGAUGUCa 5'
|||:| ||:::| |||||:|
Target:5' aaGGAGGCTTGTTTAAACTACGGa 3'</td><td>-17.60</td><td>150.00</td><td></td></tr>
</table>

131	hsa-miR-20b*						?
		1891~1915	25	miRNA: 3' gaCCUUCACGGGU---AUGAUGUCa 5'<br>||:: ||||:: |||:||| Target:5' ttGGGGCTGCCTGAGCCACTGCAGg 3'	-18.60	140.00	
132	hsa-miR-211	53~77	25	miRNA: 3' ucCGCUUC----CUACUGUUUCCCUu 5'<br>| |||| || |||||||||| Target:5' gaGGGAAGTCCCGA-GACAAAGGGAa 3'	-23.00	165.00	?
		290~310	21	miRNA: 3' uccgcuUCCUACUGUUUCCCUu 5'<br>|||| | ||||||| Target:5' cattccAGGA-AAGAAAGGGAc 3'	-11.30	151.00	
		580~602	23	miRNA: 3' ucCGCUUCCUACU-GUUUCCCuu 5'<br>|:|:|| | || |||||| Target:5' gaGTGGAGTACGAGAAAAGGGtc 3'	-14.40	140.00	
		2215~2242	28	miRNA: 3' ucCGCU---UCCU---ACUGUUUCCCUu 5'<br>|||: |||: ||: ||:|||| Target:5' ggGCGGTCCAGGGCCCTGGGAAGGGGAg 3'	-24.80	140.00	
133	hsa-miR-2113	1352~1372	21	miRNA: 3' cacUGUCUCGGUUCGUGUUUa 5'<br>||||:||:: |:||||| Target:5' taaACAGGGCTGTGTACAAAg 3'	-17.20	154.00	?
		592~613	22	miRNA: 3' cacUGUCUC-GGUUCGUGUUUa 5'<br>| ||:| ||:|||||||:<br>Target:5' agaAAAGGGTCCGAGCACAAGc 3'	-18.90	150.00	
134	hsa-miR-2114	284~312	29	miRNA: 3' cugGCG--AAG-----UUCCUUCCCUGAu 5'<br>||| ||| ||| |||||||| Target:5' gtgCGCCATTCCAGGAAAGAAAGGGACTc 3'	-18.40	161.00	?
		651~672	22	miRNA: 3' cuGGCGAAGUUCCUUCCCUGAu 5'<br>:||: ||||||||||:| Target:5' caTCGTCAAAAGGAAGGGATTg 3'	-24.50	152.00	
		128~153	26	miRNA: 3' cuggCGAAGUU--C--CUUCCCUGAu 5'<br>||| |:: | :||||||| Target:5' cgggGCTGCGGCCGCCCGAGGGACTt 3'	-20.00	142.00	
135	hsa-miR-2114*	1311~1332	22	miRNA: 3' uucaGGGAACGA-ACUCCGAGc 5'<br>| | ||| |||||||| Target:5' tgcaCGCAGGCTGTGAGGCTCt 3'	-17.00	153.00	?

<table>
<tr><td>136</td><td>hsa-miR-2116</td><td>2816~2836</td><td>21</td><td><pre>miRNA: 3' ucUGGAGG-AUACGAUUCUUGg 5'
            ||| |: ||| |||||||:
Target:5' taACCACTGTAT-CTAAGAATc 3'</pre></td><td>-14.60</td><td>146.00</td><td>?</td></tr>
<tr><td rowspan="2">137</td><td rowspan="2">hsa-miR-214</td><td>1538~1562</td><td>25</td><td><pre>miRNA: 3' ugacGGACAGAC---ACGGACGACa 5'
              ||| || | ||||||||
Target:5' gactCCTATCCGGGCAGCCTGCTGg 3'</pre></td><td>-19.30</td><td>154.00</td><td rowspan="2">?</td></tr>
<tr><td>2091~2114</td><td>24</td><td><pre>miRNA: 3' ugaCGGAC--AGACACGGACGACa 5'
             | ||| ||| |||||||
Target:5' tggGGCTGGAGCTGGCCCTGCTGc 3'</pre></td><td>-20.40</td><td>153.00</td></tr>
<tr><td>138</td><td>hsa-miR-216a</td><td>1367~1386</td><td>20</td><td><pre>miRNA: 3' agUGUCAACGGUCGACUCUAAu 5'
            | || |||: ||||||:||
Target:5' acAAAG-TGCT-GCTGAGGTTt 3'</pre></td><td>-18.10</td><td>146.00</td><td>?</td></tr>
<tr><td>139</td><td>hsa-miR-219-1-3p</td><td>777~798</td><td>22</td><td><pre>miRNA: 3' gcCCUGCAGGUCUGAGUUGAGa 5'
            ||::|| |:| |||:|:||
Target:5' gcGGGTGTGCGGTCTCGATTCg 3'</pre></td><td>-25.40</td><td>140.00</td><td>?</td></tr>
<tr><td>140</td><td>hsa-miR-22</td><td>1407~1429</td><td>23</td><td><pre>miRNA: 3' ugucaagaaguUGA-CCGUCGAa 5'
                     :|| |||||||
Target:5' ggctgtagagcGCTGGGCAGCTa 3'</pre></td><td>-12.50</td><td>143.00</td><td>?</td></tr>
<tr><td>141</td><td>hsa-miR-222*</td><td>1360~1382</td><td>23</td><td><pre>miRNA: 3' uccUAGAUGUGAC-CGAUGACUc 5'
             :| |||| | |||:||||
Target:5' gctGTGTACAAAGTGCTGCTGAg 3'</pre></td><td>-17.80</td><td>143.00</td><td>?</td></tr>
<tr><td>142</td><td>hsa-miR-223*</td><td>1004~1031</td><td>28</td><td><pre>miRNA: 3' uuGAGUCGA---ACA---GUUUAUGUGc 5'
            ||||||| ||| ||||:|||
Target:5' gcCTCAGCTGGCTGTATGGAAATGCACc 3'</pre></td><td>-15.70</td><td>152.00</td><td>?</td></tr>
<tr><td rowspan="2">143</td><td rowspan="2">hsa-miR-224</td><td>2630~2649</td><td>20</td><td><pre>miRNA: 3' uugccUUGGUGAUCACUGAAc 5'
               ||:|: |||||||||
Target:5' tcgccAATCG-TAGTGACTTc 3'</pre></td><td>-14.10</td><td>159.00</td><td rowspan="2">?</td></tr>
<tr><td>2292~2312</td><td>21</td><td><pre>miRNA: 3' uuGCCUUGGUGAUCACUGAAc 5'
             || |::| :|||||||
Target:5' atCGAAGTTAGAGGTGACTTc 3'</pre></td><td>-12.80</td><td>155.00</td></tr>
<tr><td>144</td><td>hsa-miR-2277-5p</td><td>1~9</td><td>9</td><td><pre>miRNA: 3' cugaccgucgcgaguCGGGCGCGa 5'
                         ||||||||
Target:5' ---------------GCCCGCGCc 3'</pre></td><td>-15.80</td><td>145.00</td><td>?</td></tr>
<tr><td></td><td>hsa-</td><td></td><td></td><td></td><td></td><td></td><td></td></tr>
</table>

145	miR-2278	8~30	23	miRNA: 3' ggUCCGUUGUGUGU-GACGAGAg 5'<br>||| | |: | ||||||| Target:5' ccAGGGTCCTCGGAGCTGCTCTg 3'	-18.80	148.00	
146	hsa-miR-2355-5p	1805~1826	22	miRNA: 3' aaCAGGUAACAUA-GACCCCUa 5'<br>|| :: | |:| ||||||| Target:5' gaGTGTGGTCTGTCCTGGGGAg 3'	-14.30	151.00	
		2455~2475	21	miRNA: 3' aacagguaacAUAGACCCCUa 5'<br>|| ||||||| Target:5' cacggggcctTACCTGGGGAa 3'	-12.60	147.00	
147	hsa-miR-2355-3p	438~461	24	miRNA: 3' uagAGGU---UUGUCGUUCCUGUUa 5'<br>|||| | || :||||||| Target:5' ctgTCCATCTTAGAG-GAGGACAAg 3'	-15.90	150.00	
148	hsa-miR-24	1840~1862	23	miRNA: 3' gacaaGGACGACU-UGACUCGGu 5'<br>| | |||| :||||||| Target:5' gcagcCGTCCTGACGCTGAGCCc 3'	-19.00	157.00	
		1885~1908	24	miRNA: 3' gacaAGGA---CGACUUGACUCGGu 5'<br>||:| |||| ||||||| Target:5' ggccTCTTGGGGCTG-CCTGAGCCa 3'	-19.30	153.00	
		2396~2418	23	miRNA: 3' gacaaGGACGAC-UUGACUCGGu 5'<br>||||| | |||||||:<br>Target:5' cgcacCCTGCAGTCACTGAGCTc 3'	-18.10	145.00	
		1840~1862	23	miRNA: 3' gacaaGGACGACU-UGACUCGGu 5'<br>| | |||| :||||||| Target:5' gcagcCGTCCTGACGCTGAGCCc 3'	-19.00	157.00	
		1885~1908	24	miRNA: 3' gacaAGGA---CGACUUGACUCGGu 5'<br>||:| |||| ||||||| Target:5' ggccTCTTGGGGCTG-CCTGAGCCa 3'	-19.30	153.00	
		2396~2418	23	miRNA: 3' gacaaGGACGAC-UUGACUCGGu 5'<br>||||| | |||||||:<br>Target:5' cgcacCCTGCAGTCACTGAGCTc 3'	-18.10	145.00	
149	hsa-miR-25*	356~374	19	miRNA: 3' guUAACGGGUUCAGAGGCGGa 5'<br>||| :||| |||:|||| Target:5' tgATTATCCA--TCTTCGCCa 3'	-16.70	147.00	
		920~941	22	miRNA: 3' acGAGUGUUCGUCGAUUCGGGa 5'<br>:|:|:| ::| ||||:|| Target:5' ctTTTATACTTGGGTAAGTCCc 3'	-11.00	140.00	

<table>
<tr><td rowspan="2">150</td><td rowspan="2">hsa-miR-27a*</td><td></td><td></td><td>Target:5' [illegible] 3'</td><td></td><td></td><td rowspan="2"></td></tr>
<tr><td>1834~1863</td><td>30</td><td>miRNA: 3' acGAGUGUUCG-------U-CGAUUCGGGa 5'
|||| ||| | |||:|||||
Target:5' gcCTCAGCAGCCGTCCTGACGCTGAGCCCt 3'</td><td>-21.50</td><td>140.00</td></tr>
<tr><td rowspan="2">151</td><td rowspan="2">hsa-miR-27b*</td><td>2395~2419</td><td>25</td><td>miRNA: 3' caaGUG----GUUAGUCGAUUCGAGa 5'
||| ||:||| ||:|||||
Target:5' acgCACCCTGCAGTCA-CTGAGCTCt 3'</td><td>-20.70</td><td>148.00</td><td rowspan="2"></td></tr>
<tr><td>105~126</td><td>22</td><td>miRNA: 3' caaGUGGUUAGUCGAUUCGAGa 5'
|||| :|| |||| ||||
Target:5' ctcCACCTGTCCGCTACGCTCg 3'</td><td>-16.80</td><td>143.00</td></tr>
<tr><td rowspan="2">152</td><td rowspan="2">hsa-miR-2861</td><td>1494~1513</td><td>20</td><td>miRNA: 3' ggCGG-GUGGCGGUCCGGGg 5'
||| || ||||||:||
Target:5' ggGCCACAAGGCCAGGTCCa 3'</td><td>-25.30</td><td>145.00</td><td rowspan="2"></td></tr>
<tr><td>2245~2262</td><td>18</td><td>miRNA: 3' ggCGGGUGGCGGUCCGGGg 5'
|| | || |||||:||
Target:5' cgGCAC-CCTCCAGGTCCg 3'</td><td>-20.70</td><td>140.00</td></tr>
<tr><td rowspan="2">153</td><td rowspan="2">hsa-miR-296-3p</td><td>1725~1744</td><td>20</td><td>miRNA: 3' ccUCUCGGAGGUGGGUUGGGAg 5'
||:||:|| |||:|||||
Target:5' ccAGGGCTTC--CCCGACCCTt 3'</td><td>-26.40</td><td>156.00</td><td rowspan="2"></td></tr>
<tr><td>1996~2020</td><td>25</td><td>miRNA: 3' ccUCUCGG---AGGUGGGUUGGGAg 5'
| |||| | | ||||:||||
Target:5' acACAGCCCGGTGCTCCCAGCCCTc 3'</td><td>-25.30</td><td>148.00</td></tr>
<tr><td>154</td><td>hsa-miR-299-3p</td><td>1378~1402</td><td>25</td><td>miRNA: 3' uuCGCCAAAUGGUA----GGGUGUAu 5'
| ||||| |::| |||:|||
Target:5' ctGAGGTTT-CTGTGCTCCCCGCATc 3'</td><td>-12.80</td><td>141.00</td><td></td></tr>
<tr><td>155</td><td>hsa-miR-300</td><td>1628~1649</td><td>22</td><td>miRNA: 3' ucUCUCUCAGAC-GGGAACAUAu 5'
|| | ||||| |||||||:|
Target:5' tcAG-GTGTCTGTCCCTTGTGTc 3'</td><td>-20.40</td><td>155.00</td><td></td></tr>
<tr><td>156</td><td>hsa-miR-302a</td><td>2825~2853</td><td>29</td><td>miRNA: 3' aguGGUUUU------GUACCUUCGUGAAu 5'
|:||:| ||| |||||:||
Target:5' tatCTAAGAATCCACCATTAAAGCATTTg 3'</td><td>-12.20</td><td>142.00</td><td></td></tr>
<tr><td>157</td><td>hsa-miR-302b</td><td>2825~2853</td><td>29</td><td>miRNA: 3' gauGAUUUU------GUACCUUCGUGAAu 5'
||||:| ||| |||||:||
Target:5' tatCTAAGAATCCACCATTAAAGCATTTg 3'</td><td>-10.70</td><td>146.00</td><td></td></tr>
</table>

<table>
<tr><td>158</td><td>hsa-miR-302c</td><td>2831~2853</td><td>23</td><td>miRNA: 3' ggUGACUUUGUACCUUCGUGAAu 5'
            | | | ||| |||||:||
Target:5' agAATCCACCATTAAAGCATTTg 3'</td><td>-12.90</td><td>141.00</td><td></td></tr>
<tr><td>159</td><td>hsa-miR-302d</td><td>2831~2853</td><td>23</td><td>miRNA: 3' ugUGAGUUUGUACCUUCGUGAAu 5'
            | || | ||| |||||:||
Target:5' agAATCCACCATTAAAGCATTTg 3'</td><td>-9.20</td><td>149.00</td><td></td></tr>
<tr><td>160</td><td>hsa-miR-30a</td><td>672~694</td><td>23</td><td>miRNA: 3' gaAGGUCAGCUCCU--ACAAAUGu 5'
            |:::| | || | |||||||
Target:5' ggTTTGG-CAAGAACTTGTTTACa 3'</td><td>-7.70</td><td>149.00</td><td></td></tr>
<tr><td>161</td><td>hsa-miR-30a*</td><td>1200~1221</td><td>22</td><td>miRNA: 3' cgACGU--UUGUAGGCUGACUUUc 5'
            |||| | :||| |:|||||
Target:5' gtTGCATTATTATC--ATTGAAAg 3'</td><td>-14.50</td><td>142.00</td><td></td></tr>
<tr><td rowspan="2">162</td><td rowspan="2">hsa-miR-30b</td><td>667~694</td><td>28</td><td>miRNA: 3' ucgACUCACAUC---CU---ACAAAUGu 5'
             | :|| |:| || |||||||
Target:5' ggaTTGGTTTGGCAAGAACTTGTTTACa 3'</td><td>-8.09</td><td>147.00</td><td rowspan="2"></td></tr>
<tr><td>2429~2453</td><td>25</td><td>miRNA: 3' ucGACU-C--ACAUCCUACAAAUGu 5'
            |||| | |||:| ||||||:
Target:5' gtCTGATGTTTGTGGTTTGTTTATa 3'</td><td>-13.90</td><td>146.00</td></tr>
<tr><td rowspan="2">163</td><td rowspan="2">hsa-miR-30b*</td><td>1936~1956</td><td>21</td><td>miRNA: 3' cuUCAUUUGUAGGUGGAGGGUc 5'
            :|| : |: :|||||||||
Target:5' tgGGT-GCCGGTCACCTCCCAg 3'</td><td>-24.60</td><td>155.00</td><td rowspan="2"></td></tr>
<tr><td>1224~1242</td><td>19</td><td>miRNA: 3' cuUCAUUUGUAGGUGGAGGGUc 5'
            || |::|:   :|||||||
Target:5' agAGGAGGCG---GCCTCCCAg 3'</td><td>-21.90</td><td>149.00</td></tr>
<tr><td rowspan="2">164</td><td rowspan="2">hsa-miR-30c</td><td>663~694</td><td>32</td><td>miRNA: 3' cgaCUCU---CACAUC---CU---ACAAAUGu 5'
             |:|| || |:| || |||||||
Target:5' gaaGGGATTGGTTTGGCAAGAACTTGTTTACa 3'</td><td>-10.80</td><td>148.00</td><td rowspan="2"></td></tr>
<tr><td>2430~2453</td><td>24</td><td>miRNA: 3' cgACU-CUCACAUCCUACAAAUGu 5'
            ||| | |||:| ||||||:
Target:5' tcTGATGTTTGTGGTTTGTTTATa 3'</td><td>-16.00</td><td>145.00</td></tr>
<tr><td>165</td><td>hsa-miR-30c-1*</td><td>2027~2050</td><td>24</td><td>miRNA: 3' ccucauuuGUUGGG--AGAGGGuc 5'
                  |||||| ||||||
Target:5' ctggctcccAACCCAGTCTCCCca 3'</td><td>-16.90</td><td>140.00</td><td></td></tr>
</table>

<table>
<tr><td rowspan="2">166</td><td rowspan="2">hsa-miR-30c</td><td>663~694</td><td>32</td><td>miRNA: 3' cgaCUCU---CACAUC---CU---ACAAAUGu 5'
|:|| || |:| || |||||||
Target:5' gaaGGGATTGGTTTGGCAAGAACTTGTTTACa 3'</td><td>-10.80</td><td>148.00</td><td rowspan="2"></td></tr>
<tr><td>2430~2453</td><td>24</td><td>miRNA: 3' cgACU-CUCACAUCCUACAAAUGu 5'
||| | |||:| ||||||:
Target:5' tcTGATGTTTGTGGTTTGTTTATa 3'</td><td>-16.00</td><td>145.00</td></tr>
<tr><td>167</td><td>hsa-miR-30d</td><td>667~694</td><td>28</td><td>miRNA: 3' gaaGGUCAGCCC---CU---ACAAAUGu 5'
:::||: || || |||||||
Target:5' ggaTTGGTTTGGCAAGAACTTGTTTACa 3'</td><td>-8.80</td><td>147.00</td><td></td></tr>
<tr><td>168</td><td>hsa-miR-30e</td><td>672~694</td><td>23</td><td>miRNA: 3' gaAGGUCAGUUCCU--ACAAAUGu 5'
|:::| |||| | |||||||
Target:5' ggTTTGG-CAAGAACTTGTTTACa 3'</td><td>-10.70</td><td>157.00</td><td></td></tr>
<tr><td>169</td><td>hsa-miR-31</td><td>1319~1341</td><td>23</td><td>miRNA: 3' ucGAUACGGUCG---UAGAACGGa 5'
||:|| :|| :|||||||
Target:5' ggCTGTG-AGGCTCTGTCTTGCCc 3'</td><td>-19.20</td><td>150.00</td><td></td></tr>
<tr><td rowspan="7"></td><td rowspan="7"></td><td>2390~2412</td><td>23</td><td>miRNA: 3' ucuuaaaaGUAUUACGUCAGUGu 5'
|| |||||||||
Target:5' cggctacgCACCCTGCAGTCACt 3'</td><td>-13.50</td><td>151.00</td><td rowspan="7"></td></tr>
<tr><td>711~739</td><td>29</td><td>miRNA: 3' ucUUAA--AAGUA--UUAC--GUCAGUGu 5'
|:|| | ||| |||| :||||||
Target:5' aaAGTTGCTCCATACAATGACTAGTCACc 3'</td><td>-8.90</td><td>147.00</td></tr>
<tr><td>2719~2740</td><td>22</td><td>miRNA: 3' ucUUAAAAGUAUUACGUCAGUGu 5'
|: | | | || ||:||||
Target:5' agAGCTCTGA-AACGCGGTCACc 3'</td><td>-10.41</td><td>140.00</td></tr>
<tr><td>2390~2412</td><td>23</td><td>miRNA: 3' ucuuaaaaGUAUUACGUCAGUGu 5'
|| |||||||||
Target:5' cggctacgCACCCTGCAGTCACt 3'</td><td>-13.50</td><td>151.00</td></tr>
<tr><td>711~739</td><td>29</td><td>miRNA: 3' ucUUAA--AAGUA--UUAC--GUCAGUGu 5'
|:|| | ||| |||| :||||||
Target:5' aaAGTTGCTCCATACAATGACTAGTCACc 3'</td><td>-8.90</td><td>147.00</td></tr>
<tr><td>2719~2740</td><td>22</td><td>miRNA: 3' ucUUAAAAGUAUUACGUCAGUGu 5'
|: | | | || ||:||||
Target:5' agAGCTCTGA-AACGCGGTCACc 3'</td><td>-10.41</td><td>140.00</td></tr>
<tr><td>2390~2412</td><td>23</td><td>miRNA: 3' ucuuaaaaGUAUUACGUCAGUGu 5'
[illegible]</td><td>-13.50</td><td>151.00</td></tr>
</table>

				|| ||||||||| Target:5' cggctacgCACCCTGCAGTCACt 3'			
		711~739	29	miRNA: 3' ucUUAA--AAGUA--UUAC--GUCAGUGu 5' |:||	||| |||| :|||||| Target:5' aaAGTTGCTCCATACAATGACTAGTCACc 3'	-8.90	147.00	
170	hsa-miR-3118	2719~2740	22	miRNA: 3' ucUUAAAAGUAUUACGUCAGUGu 5' |:			|| ||:||||| Target:5' agAGCTCTGA-AACGCGGTCACc 3'	-10.41	140.00	
		2390~2412	23	miRNA: 3' ucuuaaaaGUAUUACGUCAGUGu 5' || ||||||||| Target:5' cggctacgCACCCTGCAGTCACt 3'	-13.50	151.00	
		711~739	29	miRNA: 3' ucUUAA--AAGUA--UUAC--GUCAGUGu 5' |:||	||| |||| :|||||| Target:5' aaAGTTGCTCCATACAATGACTAGTCACc 3'	-8.90	147.00	
		2719~2740	22	miRNA: 3' ucUUAAAAGUAUUACGUCAGUGu 5' |:			|| ||:||||| Target:5' agAGCTCTGA-AACGCGGTCACc 3'	-10.41	140.00	
		2390~2412	23	miRNA: 3' ucuuaaaaGUAUUACGUCAGUGu 5' || ||||||||| Target:5' cggctacgCACCCTGCAGTCACt 3'	-13.50	151.00	
		711~739	29	miRNA: 3' ucUUAA--AAGUA--UUAC--GUCAGUGu 5' |:||	||| |||| :|||||| Target:5' aaAGTTGCTCCATACAATGACTAGTCACc 3'	-8.90	147.00	
		2719~2740	22	miRNA: 3' ucUUAAAAGUAUUACGUCAGUGu 5' |:			|| ||:||||| Target:5' agAGCTCTGA-AACGCGGTCACc 3'	-10.41	140.00	
		2390~2412	23	miRNA: 3' ucuuaaaaGUAUUACGUCAGUGu 5' || ||||||||| Target:5' cggctacgCACCCTGCAGTCACt 3'	-13.50	151.00	
		711~739	29	miRNA: 3' ucUUAA--AAGUA--UUAC--GUCAGUGu 5' |:||	||| |||| :|||||| Target:5' aaAGTTGCTCCATACAATGACTAGTCACc 3'	-8.90	147.00	
		2719~2740	22	miRNA: 3' ucUUAAAAGUAUUACGUCAGUGu 5' |:			|| ||:||||| Target:5' agAGCTCTGA-AACGCGGTCACc 3'	-10.41	140.00	

<table>
<tr><td>171</td><td>hsa-miR-3120</td><td>1147~1166</td><td>20</td><td>miRNA: 3' acGGACAGAUGUGAACGACAc 5'
::|||||::: |||||||
Target:5' gtTTTGTCTGTG-TTGCTGTt 3'</td><td>-15.80</td><td>162.00</td><td>?</td></tr>
<tr><td rowspan="2">172</td><td rowspan="2">hsa-miR-3122</td><td>219~244</td><td>26</td><td>miRNA: 3' uucuGGCAGGAG-A---ACAGGGUUg 5'
::| ::|| | ||||||||
Target:5' ccatTTGGTTTCGTGGCTGTCCCAAc 3'</td><td>-17.80</td><td>146.00</td><td rowspan="2">?</td></tr>
<tr><td>2151~2172</td><td>22</td><td>miRNA: 3' uucuggcaggagaaCAGGGUUg 5'
|||||||
Target:5' ggcccaagtgcaggGTCCCAAg 3'</td><td>-13.40</td><td>140.00</td></tr>
<tr><td>173</td><td>hsa-miR-3125</td><td>1115~1134</td><td>20</td><td>miRNA: 3' agAGAGGUGUCGAAGGAGAu 5'
||| |:| ||||||:|
Target:5' gtTCTGCGCCACTTCCTTTg 3'</td><td>-19.50</td><td>146.00</td><td>?</td></tr>
<tr><td>174</td><td>hsa-miR-3126-5p</td><td>1624~1645</td><td>22</td><td>miRNA: 3' acGAAGACCGUAGACAGGGAGu 5'
| || ||::|||||||||:
Target:5' caCCTCAGGTGTCTGTCCCTTg 3'</td><td>-21.10</td><td>160.00</td><td>?</td></tr>
<tr><td>175</td><td>hsa-miR-3126-3p</td><td>1671~1692</td><td>22</td><td>miRNA: 3' agacaCACUGCCUACGGUCUAc 5'
|||:| | |||||:|
Target:5' cacccGTGGCAAACGCCAGGTg 3'</td><td>-17.70</td><td>141.00</td><td>?</td></tr>
<tr><td>176</td><td>hsa-miR-3127</td><td>2213~2234</td><td>22</td><td>miRNA: 3' gaaggGUAAGGUGUUCGGGACUa 5'
|: |||| ::||||||:
Target:5' gagggCGGTCCA-GGGCCCTGGg 3'</td><td>-19.50</td><td>141.00</td><td>?</td></tr>
<tr><td>177</td><td>hsa-miR-3129</td><td>887~908</td><td>22</td><td>miRNA: 3' uuUGGUUAGAGAUGUGAUGACg 5'
::||:| |:| |||:|||
Target:5' atGTCAGTATTTCAACTGCTGt 3'</td><td>-12.80</td><td>144.00</td><td>?</td></tr>
<tr><td rowspan="2">178</td><td rowspan="2">hsa-miR-3130-5p</td><td>2127~2145</td><td>19</td><td>miRNA: 3' ccgACGUGGCCUCUGACCCAu 5'
|| :|:||| :||||||
Target:5' cggTG-GCTGGA-GCTGGGTc 3'</td><td>-21.50</td><td>140.00</td><td rowspan="2">?</td></tr>
<tr><td>2127~2145</td><td>19</td><td>miRNA: 3' ccgACGUGGCCUCUGACCCAu 5'
|| :|:||| :||||||
Target:5' cggTG-GCTGGA-GCTGGGTc 3'</td><td>-21.50</td><td>140.00</td></tr>
<tr><td></td><td></td><td>1~20</td><td>20</td><td>miRNA: 3' uucCGGGAAGGUGGU--CAGGAGCu 5'
|||| |:||| |||||||
Target:5' ---GCCC--GCGCCAGGGTCCTCGg 3'</td><td>-26.00</td><td>158.00</td><td></td></tr>
</table>

<table>
<tr><th></th><th></th><th></th><th></th><th></th><th></th><th></th><th></th></tr>
<tr><td rowspan="2">179</td><td rowspan="2">hsa-miR-3131</td><td>2659~2688</td><td>30</td><td>miRNA: 3' uucCGGGAAG-----GUGGU--CAGGAGCu 5'
|::|||:   :|||| |||||||
Target:5' aatGTTCTTTTGTAGTACCACGGTCCTCGg 3'</td><td>-22.70</td><td>158.00</td><td rowspan="2">?</td></tr>
<tr><td>86~109</td><td>24</td><td>miRNA: 3' uucCGGGAAGGUG-GUCAGGAGcu 5'
||| ||:| |:||||||
Target:5' gccGCCGCCCCGCTCGGTCCTCca 3'</td><td>-21.20</td><td>140.00</td></tr>
<tr><td>180</td><td>hsa-miR-3134</td><td>346~368</td><td>23</td><td>miRNA: 3' uuauacaUCAGAAAAUAGGUAGu 5'
|| : |||||||||
Target:5' ttttcaaAGATGATTATCCATCt 3'</td><td>-9.90</td><td>152.00</td><td>?</td></tr>
<tr><td>181</td><td>hsa-miR-3138</td><td>91~119</td><td>29</td><td>miRNA: 3' ugaGGGAGA---UGGA-GU-GACAGGUGu 5'
||| || ||| || ||||||:|
Target:5' cgcCCCGCTCGGTCCTCCACCTGTCCGCt 3'</td><td>-24.40</td><td>145.00</td><td>?</td></tr>
<tr><td>182</td><td>hsa-miR-3140</td><td>2300~2320</td><td>21</td><td>miRNA: 3' ugauggACUUAAGGGUUUUCGa 5'
||| || |||||:||
Target:5' tagaggTGACTT-CCAAAGGCc 3'</td><td>-11.20</td><td>143.00</td><td>?</td></tr>
<tr><td>183</td><td>hsa-miR-3141</td><td>1831~1851</td><td>21</td><td>miRNA: 3' agGAGGAG--GUGGGCGGGAg 5'
| |||| || |||:|||
Target:5' tgCGCCTCAGCAGCCGTCCTg 3'</td><td>-21.40</td><td>143.00</td><td>?</td></tr>
<tr><td>184</td><td>hsa-miR-3144-5p</td><td>2128~2149</td><td>22</td><td>miRNA: 3' gauauauagagaAACCAGGGGa 5'
| |||||||
Target:5' ggtggctggagcTGGGTCCCCg 3'</td><td>-15.50</td><td>142.00</td><td>?</td></tr>
<tr><td>185</td><td>hsa-miR-3145</td><td>511~534</td><td>24</td><td>miRNA: 3' guuAAGGUUUGUGAGUUUUAUAGa 5'
|||:|||:: | |||||||
Target:5' aacTTCTAAATGAACCAAATATCc 3'</td><td>-7.80</td><td>169.00</td><td>?</td></tr>
<tr><td rowspan="3">186</td><td rowspan="3">hsa-miR-3147</td><td>2674~2695</td><td>22</td><td>miRNA: 3' aguGUGGGAGGAGUGACGGGUUGg 5'
||| |||||: |||:|||
Target:5' tacCACGGTCCTCG--GCCTAACg 3'</td><td>-20.30</td><td>149.00</td><td rowspan="3">?</td></tr>
<tr><td>1229~1251</td><td>23</td><td>miRNA: 3' agUGUGGGAGGAGUGACGGGUUGg 5'
:|: ||||| || |||||::|
Target:5' agGCGGCCTCC-CAGTGCCCGGCc 3'</td><td>-33.00</td><td>141.00</td></tr>
<tr><td>1254~1276</td><td>23</td><td>miRNA: 3' aguGUGGGAGGAGUGACGGGUUGg 5'
||||| ||| :| |||| ||
Target:5' cccCACCCACCT-GCAGCCCCACc 3'</td><td>-24.00</td><td>140.00</td></tr>
<tr><td></td><td>hsa-</td><td></td><td></td><td>miRNA: 3' [illegible]</td><td></td><td></td><td></td></tr>
</table>

<table>
<tr><td>187</td><td>miR-
3148</td><td>1677~1701</td><td>25</td><td>[illegible]
||| ||:||| || ||||||:
Target:5' tgGCAAACGCCAGGTGCTTTTTCTg 3'</td><td>-16.90</td><td>154.00</td><td>?</td></tr>
<tr><td rowspan="2">188</td><td rowspan="2">hsa-
miR-
3149</td><td>705~728</td><td>24</td><td>miRNA: 3' uaUGUGUGUGUAU-AGGUAUGUUu 5'
|: :| | |: |||||||||
Target:5' aaATCTAAAGTTGCTCCATACAAt 3'</td><td>-9.40</td><td>153.00</td><td rowspan="2">?</td></tr>
<tr><td>800~822</td><td>23</td><td>miRNA: 3' uauguGUGUGUAUAGGUAUGUUu 5'
::| |:| ||||||||:
Target:5' tgaatTGCCCGTTTCCATACAGg 3'</td><td>-13.70</td><td>146.00</td></tr>
<tr><td rowspan="3">189</td><td rowspan="3">hsa-
miR-
3150</td><td>2034~2051</td><td>18</td><td>miRNA: 3' ggUUGGAGCUCCUAGAGGGGUc 5'
|||| | || ||||||||
Target:5' ccAACC-C-AG--TCTCCCCAt 3'</td><td>-21.10</td><td>154.00</td><td rowspan="3">?</td></tr>
<tr><td>2175~2194</td><td>20</td><td>miRNA: 3' gguuggaGCUCCUAGAGGGGUc 5'
||:|| |||||||
Target:5' ggcagggCGGGG--CTCCCCAa 3'</td><td>-21.20</td><td>151.00</td></tr>
<tr><td>1238~1259</td><td>22</td><td>miRNA: 3' gguuggagcuCCUAGAGGGGUc 5'
|| |||||||
Target:5' cccagtgcccGGCCCTCCCCAc 3'</td><td>-19.20</td><td>144.00</td></tr>
<tr><td>190</td><td>hsa-
miR-
3151</td><td>1256~1276</td><td>21</td><td>miRNA: 3' uggacuaGGGUAACGGGGUGg 5'
||:: |||||||
Target:5' ccacccaCCTGCAGCCCCACc 3'</td><td>-21.10</td><td>146.00</td><td>?</td></tr>
<tr><td>191</td><td>hsa-
miR-
3153</td><td>2370~2391</td><td>22</td><td>miRNA: 3' uuUACAGGGAUGAGCGAAAGGGg 5'
:|| ||:|::| |||||:||
Target:5' caGTG-CCTTGTTGGCTTTTCCg 3'</td><td>-20.50</td><td>152.00</td><td>?</td></tr>
<tr><td>192</td><td>hsa-
miR-
3155</td><td>2043~2065</td><td>23</td><td>miRNA: 3' ucAAGGGU--GA-CGUCUCGGACc 5'
| |||| || || ||||:||
Target:5' tcTCCCCATCCTAGC-GAGCTTGg 3'</td><td>-20.20</td><td>140.00</td><td>?</td></tr>
<tr><td rowspan="3">193</td><td rowspan="3">hsa-
miR-
3156</td><td>1326~1350</td><td>25</td><td>miRNA: 3' acaGAGGGUGAA---GGUCUAGAAa 5'
|||: ||| |::||||||
Target:5' aggCTCTGTCTTGCCCTGGATCTTt 3'</td><td>-15.10</td><td>143.00</td><td rowspan="3">?</td></tr>
<tr><td>1326~1350</td><td>25</td><td>miRNA: 3' acaGAGGGUGAA---GGUCUAGAAa 5'
|||: ||| |::||||||
Target:5' aggCTCTGTCTTGCCCTGGATCTTt 3'</td><td>-15.10</td><td>143.00</td></tr>
<tr><td>1326~1350</td><td>25</td><td>miRNA: 3' acaGAGGGUGAA---GGUCUAGAAa 5'
|||: ||| |::||||||
Target:5' aggCTCTGTCTTGCCCTGGATCTTt 3'</td><td>-15.10</td><td>143.00</td></tr>
</table>

<table>
<tr><td rowspan="3">194</td><td rowspan="3">hsa-miR-3157</td><td>1906~1931</td><td>26</td><td>miRNA: 3' ucUGACG----UGAUCGGACCGACUu 5'
||||| | |:||||||||||:
Target:5' ccACTGCAGGAAGTGGCCTGGCTGGg 3'</td><td>-26.50</td><td>158.00</td><td rowspan="3"></td></tr>
<tr><td>992~1019</td><td>28</td><td>miRNA: 3' ucUGACGUG-AUCG-----GACCGACuu 5'
:|||||| |:|| |||||||
Target:5' gaGCTGCACTTGGCCTCAGCTGGCTGta 3'</td><td>-23.90</td><td>148.00</td></tr>
<tr><td>2108~2137</td><td>30</td><td>miRNA: 3' ucUGACGUGAUC------GG--ACCGACUu 5'
:|||| ||:| || ||||||:
Target:5' ctGCTGCCCTGGCACCCCCCGGTGGCTGGa 3'</td><td>-22.20</td><td>140.00</td></tr>
<tr><td rowspan="2">195</td><td rowspan="2">hsa-miR-3158</td><td>1968~1991</td><td>24</td><td>miRNA: 3' caggacGUCUCU--CCUUCGGGAa 5'
|||||| |||||||||
Target:5' agtggaCAGAGATGGGAAGCCCTg 3'</td><td>-23.90</td><td>170.00</td><td rowspan="2"></td></tr>
<tr><td>1968~1991</td><td>24</td><td>miRNA: 3' caggacGUCUCU--CCUUCGGGAa 5'
|||||| |||||||||
Target:5' agtggaCAGAGATGGGAAGCCCTg 3'</td><td>-23.90</td><td>170.00</td></tr>
<tr><td>196</td><td>hsa-miR-3162</td><td>1027~1055</td><td>29</td><td>miRNA: 3' gagGGGUGGGAAGAU------GAGGGAUu 5'
||| |||| ||: |||:|||
Target:5' gcaCCCTCCCTCCTGCCGCTCCTCTCTAg 3'</td><td>-23.60</td><td>146.00</td><td></td></tr>
<tr><td rowspan="2">197</td><td rowspan="2">hsa-miR-3163</td><td>860~882</td><td>23</td><td>miRNA: 3' cagaaUGA-CGGGAGUAAAAUau 5'
||| ||::|:||||||
Target:5' gtaaaACTCGCTTTTATTTTAat 3'</td><td>-10.40</td><td>145.00</td><td rowspan="2"></td></tr>
<tr><td>2506~2526</td><td>21</td><td>miRNA: 3' cagaaUGACGGGAGUAAAAUAu 5'
| || :||:|||||:|
Target:5' cccagAATG-TCTTATTTTGTa 3'</td><td>-9.40</td><td>140.00</td></tr>
<tr><td>198</td><td>hsa-miR-3165</td><td>2820~2841</td><td>22</td><td>miRNA: 3' acuccaguguaaCGUAGGUGGa 5'
| |||||||
Target:5' cactgtatctaaGAATCCACCa 3'</td><td>-9.79</td><td>142.00</td><td></td></tr>
<tr><td>199</td><td>hsa-miR-3166</td><td>1757~1782</td><td>26</td><td>miRNA: 3' auCCGGUCAUCCGU---A-ACAGACgc 5'
|||| |:|||| | ||||||
Target:5' agGGCC-CTGGGCAGCCTGTGTCTGga 3'</td><td>-23.10</td><td>140.00</td><td></td></tr>
<tr><td>200</td><td>hsa-miR-3169</td><td>86~107</td><td>22</td><td>miRNA: 3' gauacaCGGUUCGUGUCAGGAu 5'
||| || |:|||||
Target:5' gccgccGCCCCGCTCGGTCCTc 3'</td><td>-17.10</td><td>140.00</td><td></td></tr>
</table>

<table>
<tr><td>201</td><td>hsa-miR-3171</td><td>2557~2578</td><td>22</td><td>miRNA: 3' cuAUAUAUGUCUAAGGUAUGUAga 5'
            |:||||::| |: ||||||
Target:5' caTGTATATGG--TTAATACATat 3'</td><td>-11.60</td><td>146.00</td><td></td></tr>
<tr><td>202</td><td>hsa-miR-3175</td><td>2029~2050</td><td>22</td><td>miRNA: 3' ugcagugacgcaagAGAGGGGc 5'
                        |||||||
Target:5' ggctcccaacccagTCTCCCCa 3'</td><td>-13.70</td><td>140.00</td><td></td></tr>
<tr><td rowspan="2">203</td><td rowspan="2">hsa-miR-3176</td><td>1490~1509</td><td>20</td><td>miRNA: 3' ggCCAUCAG-GGUCCGGUCa 5'
            || :| | | |||||||
Target:5' ttGGGGGCCACAAGGCCAGg 3'</td><td>-18.40</td><td>149.00</td><td rowspan="2"></td></tr>
<tr><td>457~475</td><td>19</td><td>miRNA: 3' ggccaucagggUCCGGUCa 5'
                     |||||||
Target:5' acaaggactggAGGCCAGc 3'</td><td>-15.01</td><td>140.00</td></tr>
<tr><td>204</td><td>hsa-miR-3177</td><td>1812~1834</td><td>23</td><td>miRNA: 3' ugcACAGGGGUC--ACGGCACGu 5'
             |||||: :| ||:||||
Target:5' gtcTGTCCTGGGGAGGCTGTGCg 3'</td><td>-22.50</td><td>140.00</td><td></td></tr>
<tr><td rowspan="2">205</td><td rowspan="2">hsa-miR-3180-5p</td><td>1758~1784</td><td>27</td><td>miRNA: 3' gcugcacCCCGCC--UCGCAGACCUUc 5'
                 |||| | |:||||||||
Target:5' gggccctGGGCAGCCTGTGTCTGGAAt 3'</td><td>-22.60</td><td>160.00</td><td rowspan="2"></td></tr>
<tr><td>939~968</td><td>30</td><td>miRNA: 3' gcugcaCCCCGCCUC-----GC-AGACCUUc 5'
                ||||| ||| || |||||:|
Target:5' cccccaGGGGC-GAGTTCCTCGCTCTGGGAt 3'</td><td>-22.00</td><td>142.00</td></tr>
<tr><td>206</td><td>hsa-miR-3180-3p</td><td>76~97</td><td>22</td><td>miRNA: 3' ccgGAGGCCUUCGAGGCGGGGu 5'
             | ||| : | |||||||
Target:5' aagCGCCGCCGCCGCCGCCCCg 3'</td><td>-26.30</td><td>151.00</td><td></td></tr>
<tr><td rowspan="2">207</td><td rowspan="2">hsa-miR-3180-5p</td><td>1758~1784</td><td>27</td><td>miRNA: 3' gcugcacCCCGCC--UCGCAGACCUUc 5'
                 |||| | |:||||||||
Target:5' gggccctGGGCAGCCTGTGTCTGGAAt 3'</td><td>-22.60</td><td>160.00</td><td rowspan="2"></td></tr>
<tr><td>939~968</td><td>30</td><td>miRNA: 3' gcugcaCCCCGCCUC-----GC-AGACCUUc 5'
                ||||| ||| || |||||:|
Target:5' cccccaGGGGC-GAGTTCCTCGCTCTGGGAt 3'</td><td>-22.00</td><td>142.00</td></tr>
<tr><td>208</td><td>hsa-miR-3180-3p</td><td>76~97</td><td>22</td><td>miRNA: 3' ccgGAGGCCUUCGAGGCGGGGu 5'
             | ||| : | |||||||
Target:5' aagCGCCGCCGCCGCCGCCCCg 3'</td><td>-26.30</td><td>151.00</td><td></td></tr>
</table>

<table>
<tr><td rowspan="2">209</td><td rowspan="2">hsa-miR-3180-5p</td><td>1758~1784</td><td>27</td><td>miRNA: 3' gcugcacCCCGCC--UCGCAGACCUUc 5'
|||| | |:||||||||
Target:5' gggccctGGGCAGCCTGTGTCTGGAAt 3'</td><td>-22.60</td><td>160.00</td><td rowspan="2"></td></tr>
<tr><td>939~968</td><td>30</td><td>miRNA: 3' gcugcaCCCCGCCUC-----GC-AGACCUUc 5'
||||| ||| || |||||:|
Target:5' cccccaGGGGC-GAGTTCCTCGCTCTGGGAt 3'</td><td>-22.00</td><td>142.00</td></tr>
<tr><td>210</td><td>hsa-miR-3180-3p</td><td>76~97</td><td>22</td><td>miRNA: 3' ccgGAGGCCUUCGAGGCGGGGu 5'
| ||| : | |||||||
Target:5' aagCGCCGCCGCCGCCGCCCCg 3'</td><td>-26.30</td><td>151.00</td><td></td></tr>
<tr><td rowspan="2">211</td><td rowspan="2">hsa-miR-3180</td><td>79~97</td><td>19</td><td>miRNA: 3' gaGGCCUUCGAGGCGGGGu 5'
||| : | |||||||
Target:5' cgCCGCCGCCGCCGCCCCg 3'</td><td>-24.00</td><td>149.00</td><td rowspan="2"></td></tr>
<tr><td>79~97</td><td>19</td><td>miRNA: 3' gaGGCCUUCGAGGCGGGGu 5'
||| : | |||||||
Target:5' cgCCGCCGCCGCCGCCCCg 3'</td><td>-24.00</td><td>149.00</td></tr>
<tr><td>212</td><td>hsa-miR-3183</td><td>173~198</td><td>26</td><td>miRNA: 3' aggcUCG-CUGA---GGCUCUCUCCg 5'
||| |||| || :||||||
Target:5' cctcAGCAGACTCGCCCAGGAGAGGa 3'</td><td>-21.10</td><td>146.00</td><td></td></tr>
<tr><td>213</td><td>hsa-miR-3188</td><td>2071~2092</td><td>22</td><td>miRNA: 3' ggGGCAUAGGCGUGUUUCGGAGa 5'
| || |:||: : ||||||:
Target:5' ctCAGT-TTCGTTTCAAGCCTTg 3'</td><td>-13.10</td><td>140.00</td><td></td></tr>
<tr><td>214</td><td>hsa-miR-3189</td><td>2142~2160</td><td>19</td><td>miRNA: 3' gauGGGGUAGUCUGGGUUCcc 5'
||||:| |:||||||
Target:5' ggtCCCCGT--GGCCCAAGtg 3'</td><td>-23.70</td><td>142.00</td><td></td></tr>
<tr><td>215</td><td>hsa-miR-3190</td><td>749~770</td><td>22</td><td>miRNA: 3' agagaCCGGCAGAUGGAAGGUGu 5'
|| || | |:||||||:
Target:5' tgggcGGGCG-CCATCTTCCATt 3'</td><td>-17.70</td><td>141.00</td><td></td></tr>
<tr><td>216</td><td>hsa-miR-3191</td><td>2129~2149</td><td>21</td><td>miRNA: 3' gaCAGACCGGUCGAUGCAGGGGu 5'
| |||| ||||: ||||||
Target:5' gtGGCTGG--AGCTGGGTCCCCg 3'</td><td>-27.60</td><td>141.00</td><td></td></tr>
<tr><td rowspan="2"></td><td rowspan="2"></td><td>1221~1243</td><td>23</td><td>miRNA: 3' aaggugacgaUGUUGGAGGGUCu 5'
:|::||||||||
Target:5' gtgagaggagGCGGCCTCCCAGt 3'</td><td>-22.40</td><td>153.00</td><td rowspan="2"></td></tr>
<tr><td>1934~1957</td><td>24</td><td>miRNA: 3' aaggugAC-GAUGUUGGAGGGUCu 5'
|| | .. |||||||||</td><td>-19.30</td><td>153.00</td></tr>
</table>

<table>
<tr><td></td><td></td><td></td><td></td><td>[illegible]
Target:5' gttgggTGCCGGTCACCTCCCAGc 3'</td><td></td><td></td><td></td></tr>
<tr><td rowspan="3">217</td><td rowspan="3">hsa-miR-3192</td><td>1994~2016</td><td>23</td><td>miRNA: 3' aagGUGACGAUGUUG-GAGGGUCu 5'
||| || |::: |||||||
Target:5' ggaCACAGC-CCGGTGCTCCCAGc 3'</td><td>-17.10</td><td>151.00</td><td rowspan="3"></td></tr>
<tr><td>2488~2511</td><td>24</td><td>miRNA: 3' aagGUGACGAUGUUG---GAGGGUCu 5'
:|:| |::|:| |||||||
Target:5' gaaTATT--TGTAGCCCGCTCCCAGa 3'</td><td>-16.20</td><td>148.00</td></tr>
<tr><td>1045~1066</td><td>22</td><td>miRNA: 3' aaGGUGACGAUGUUGGAGGGUCu 5'
|| || ||| |||||:|:||
Target:5' ctCCTCT-CTAGAACCTTCTAGa 3'</td><td>-23.80</td><td>144.00</td></tr>
<tr><td rowspan="2">218</td><td rowspan="2">hsa-miR-3193</td><td>1301~1321</td><td>21</td><td>miRNA: 3' ugaGGAGUCUAGGAUGCGUCCu 5'
|:||:||| | |||||||
Target:5' ctgCTTCGGATGC-ACGCAGGc 3'</td><td>-24.40</td><td>166.00</td><td rowspan="2"></td></tr>
<tr><td>952~974</td><td>23</td><td>miRNA: 3' ugAGGAGUCUAGG--AUGCGUCCu 5'
||||| | ||: |:|||||
Target:5' gtTCCTC-GCTCTGGGATGCAGGc 3'</td><td>-20.90</td><td>141.00</td></tr>
<tr><td>219</td><td>hsa-miR-3194</td><td>2117~2138</td><td>22</td><td>miRNA: 3' guCG-GGAGGACCACCGACCgg 5'
|| || || |||||||||
Target:5' tgGCACCCCCCGGTGGCTGGag 3'</td><td>-28.60</td><td>151.00</td><td></td></tr>
<tr><td rowspan="3">220</td><td rowspan="3">hsa-miR-3196</td><td>80~97</td><td>18</td><td>miRNA: 3' cucCGGGGACGGCGGGGc 5'
||| | ||||||||
Target:5' gccGCCGCCGCCGCCCCg 3'</td><td>-29.30</td><td>159.00</td><td rowspan="3"></td></tr>
<tr><td>1032~1049</td><td>18</td><td>miRNA: 3' cuccGGGGACGGCGGGGc 5'
|:||||||||:||
Target:5' ctccCTCCTGCCGCTCCt 3'</td><td>-25.10</td><td>150.00</td></tr>
<tr><td>129~146</td><td>18</td><td>miRNA: 3' cuCCGGGGACGGCGGGgc 5'
|||: | |||||||
Target:5' ggGGCTGCGGCCGCCCga 3'</td><td>-25.70</td><td>140.00</td></tr>
<tr><td rowspan="2">221</td><td rowspan="2">hsa-miR-3197</td><td>1811~1839</td><td>29</td><td>miRNA: 3' gcGGAAAGG----CUCGG--ACGCGGAGg 5'
:|| ||| ||| | ||||||||
Target:5' ggTCTGTCCTGGGGAGGCTGTGCGCCTCa 3'</td><td>-24.80</td><td>161.00</td><td rowspan="2"></td></tr>
<tr><td>2761~2786</td><td>26</td><td>miRNA: 3' gcggaAAGGCUCGGA---CGCGGAGg 5'
|||| :|| | |:|||||
Target:5' gtgtgTTCCCGGCATGAGGTGCCTCg 3'</td><td>-20.50</td><td>142.00</td></tr>
</table>

<table>
<tr><td rowspan="2">222</td><td rowspan="2">hsa-miR-3198</td><td>2251~2275</td><td>25</td><td>miRNA: 3' agAGGUAAGGG---GUCCUGAGGUg 5'
|||| :|| ::||||||||
Target:5' ccTCCAGGTCCGTGTGGGACTCCAg 3'</td><td>-25.00</td><td>160.00</td><td rowspan="2"></td></tr>
<tr><td>1524~1545</td><td>22</td><td>miRNA: 3' agagguAAGGGGUCCUGAGGug 5'
| ||: ||||||||
Target:5' gggggcTGCCTGAGGACTCCta 3'</td><td>-19.70</td><td>140.00</td></tr>
<tr><td rowspan="2">223</td><td rowspan="2">hsa-miR-3199</td><td>2025~2047</td><td>23</td><td>miRNA: 3' uuGAAAGAGGAUUCCGUCAGGGa 5'
|| |||| || |||||:|
Target:5' ctCTGGCTCCCAACCCAGTCTCc 3'</td><td>-16.70</td><td>149.00</td><td rowspan="2"></td></tr>
<tr><td>2025~2047</td><td>23</td><td>miRNA: 3' uuGAAAGAGGAUUCCGUCAGGGa 5'
|| |||| || |||||:|
Target:5' ctCTGGCTCCCAACCCAGTCTCc 3'</td><td>-16.70</td><td>149.00</td></tr>
<tr><td rowspan="2">224</td><td rowspan="2">hsa-miR-3200-5p</td><td>1613~1634</td><td>22</td><td>miRNA: 3' uggAACACGCGGAAGAGUCUAa 5'
| | |:||| |||||:|
Target:5' gcaTAGGGTGCCACCTCAGGTg 3'</td><td>-16.20</td><td>143.00</td><td rowspan="2"></td></tr>
<tr><td>1071~1098</td><td>28</td><td>miRNA: 3' uggaACACG-CGGAA-----GAGUCUaa 5'
||||| ||:|| ||||||
Target:5' gggcTGTGCTGCTTTTGAGCCTCAGAcc 3'</td><td>-15.30</td><td>142.00</td></tr>
<tr><td rowspan="2">225</td><td rowspan="2">hsa-miR-323-5p</td><td>195~220</td><td>26</td><td>miRNA: 3' cgCUUGCGCGGUGC----CUGGUGGa 5'
||| || : : | |||||||
Target:5' agGAAAGCATGGAGGAAAGACCACCc 3'</td><td>-15.90</td><td>146.00</td><td rowspan="2"></td></tr>
<tr><td>153~174</td><td>22</td><td>miRNA: 3' cgCUUGCGCGGUGCCUGGUGGa 5'
|||| :|:|: |||:|:||
Target:5' ttGAACATGTCGGGGATCGCCc 3'</td><td>-27.20</td><td>140.00</td></tr>
<tr><td>226</td><td>hsa-miR-324-5p</td><td>946~971</td><td>26</td><td>miRNA: 3' ugUGGUUACGG---GAUCCCCUACGc 5'
:| |:| || || |||||||
Target:5' ggGCGAGTTCCTCGCTCTGGGATGCa 3'</td><td>-18.40</td><td>153.00</td><td></td></tr>
<tr><td rowspan="2">227</td><td rowspan="2">hsa-miR-328</td><td>2209~2231</td><td>23</td><td>miRNA: 3' ugCCUUCCCGUC-UCUCCCGGuc 5'
||:|||||:| ||||||
Target:5' caGGGAGGGCGGTCCAGGGCCct 3'</td><td>-29.50</td><td>148.00</td><td rowspan="2"></td></tr>
<tr><td>1741~1764</td><td>24</td><td>miRNA: 3' ugccUUCCCGUC--UCUCCCGGuc 5'
|: | ||| ||||||||
Target:5' ccttAGCGCCAGGTAGAGGGCCct 3'</td><td>-18.50</td><td>140.00</td></tr>
</table>

<table>
<tr><td rowspan="2">228</td><td rowspan="2">hsa-miR-330-3p</td><td>1066~1087</td><td>22</td><td>miRNA: 3' agaGACGUCCGGCAC-ACGAAAcg 5'
             ||| |||:||| ||||||
Target:5' aacCTG--GGCTGTGCTGCTTTtg 3'</td><td>-18.70</td><td>148.00</td><td rowspan="2"></td></tr>
<tr><td>2358~2380</td><td>23</td><td>miRNA: 3' agAGACGUC-CGGCACACGAAACg 5'
            |||||:| ||| |||| |||
Target:5' acTCTGCGGTGCC-AGTGCCTTGt 3'</td><td>-25.00</td><td>140.00</td></tr>
<tr><td>229</td><td>hsa-miR-331-3p</td><td>926~949</td><td>24</td><td>miRNA: 3' aaGAUCCUAUCC---GGGUCCCCg 5'
            || ||:|| | ||||||||
Target:5' taCTTGGGTAAGTCCCCCAGGGGc 3'</td><td>-25.70</td><td>163.00</td><td></td></tr>
<tr><td>230</td><td>hsa-miR-339-5p</td><td>2160~2182</td><td>23</td><td>miRNA: 3' gcaCUCGAGGACCUCCUGUCCCu 5'
             |:|: || ||||:|||||
Target:5' gcaGGGTCCCAAGAGGGCAGGGc 3'</td><td>-25.10</td><td>152.00</td><td></td></tr>
<tr><td>231</td><td>hsa-miR-33b*</td><td>130~153</td><td>24</td><td>miRNA: 3' ccCGACG-UGAC-GGCUCCGUGAc 5'
            ||||| :| | |||||| |||
Target:5' ggGCTGCGGCCGCCCGAGGGACTt 3'</td><td>-28.30</td><td>140.00</td><td></td></tr>
<tr><td rowspan="2">232</td><td rowspan="2">hsa-miR-340*</td><td>1213~1235</td><td>23</td><td>miRNA: 3' cgauaUUUCA-UUGACUCUGCCu 5'
               ||||| |: |||:|||
Target:5' cattgAAAGTGAGAGGAGGCGGc 3'</td><td>-16.70</td><td>141.00</td><td rowspan="2"></td></tr>
<tr><td>1961~1983</td><td>23</td><td>miRNA: 3' cgauaUUUCA-UUGACUCUGCCu 5'
               | ||| :|| ||||:||
Target:5' aaggcACAGTGGACAGAGATGGg 3'</td><td>-14.60</td><td>141.00</td></tr>
<tr><td>233</td><td>hsa-miR-342-5p</td><td>2106~2126</td><td>21</td><td>miRNA: 3' aguuagugucuAUCGUGGGGa 5'
                     |:|||||||
Target:5' ccctgctgcccTGGCACCCCc 3'</td><td>-14.40</td><td>146.00</td><td></td></tr>
<tr><td>234</td><td>hsa-miR-34a</td><td>1893~1914</td><td>22</td><td>miRNA: 3' ugUUGGUCGAUUCUGUGACGgu 5'
            ::|:: ||:|| ||||||
Target:5' ggGGCTGCCTGAGCCACTGCag 3'</td><td>-16.60</td><td>144.00</td><td></td></tr>
<tr><td>235</td><td>hsa-miR-34b*</td><td>1879~1903</td><td>25</td><td>miRNA: 3' guuaGUC--GAUUACUGUGACGGAu 5'
              ||| || ||: :||||||
Target:5' cggcCAGGCCTCTTGGGGCTGCCTg 3'</td><td>-20.60</td><td>141.00</td><td></td></tr>
<tr><td>236</td><td>hsa-miR-34c-5p</td><td>545~574</td><td>30</td><td>miRNA: 3' cgUUAGUC----GAU-UG--AUGUGACGGa 5'
            || ||| ||| || | :||||||
Target:5' tcAAGCAGAGGCCTACACGATTTACTGCCa 3'</td><td>-14.91</td><td>141.00</td><td></td></tr>
<tr><td>[illegible]</td><td>hsa-miR-[illegible]</td><td>[illegible]</td><td>[illegible]</td><td>miRNA: 3' ccgaaggaAC-GAUAGGUAGGAGu 5'</td><td>[illegible]</td><td>[illegible]</td><td></td></tr>
</table>

237	3605-5p	348~371	24	|| :||||||||:|| Target:5' ttcaaagaTGATTATCCATCTTCg 3'	-17.00	147.00	?
238	hsa-miR-3605-3p	186~209	24	miRNA: 3' gaucUCCUGUCCAUU-GUGCCUCc 5' |||| ||| || ||:|||| Target:5' gcccAGGAGAGGAAAGCATGGAGg 3'	-26.00	155.00	?
239	hsa-miR-3607-3p	677~696	20	miRNA: 3' guagUCUUUCGCAAAUGUca 5' |||| :||||||| Target:5' ggcaAGAACTTGTTTACAac 3'	-8.50	140.00	?
240	hsa-miR-361-3p	720~746	27	miRNA: 3' uuUAGUCUUAGU-----GUGGACCCCCu 5' ||	|||	|||||||||| Target:5' ccAT-ACAATGACTAGTCACCTGGGGGg 3'	-23.10	160.00	?
		1977~1996	20	miRNA: 3' uuUAGUCUUAGUGUGGACCCCCu 5' || :|||	|||||||| Target:5' agATGGGAAGC---CCTGGGGGa 3'	-22.80	158.00	
		1508~1529	22	miRNA: 3' uuuaGUCUUAGUGUGGACCCCCu 5' |||	|| : ||||||| Target:5' ggtcCAG-ACCAGGGCTGGGGGc 3'	-20.80	154.00	
		2453~2475	23	miRNA: 3' uuuaGUCUUAGUGUGGACCCCcu 5' |:|::	:|||||||| Target:5' aacaCGGGGCCTTACCTGGGGaa 3'	-23.50	143.00	
241	hsa-miR-3614-5p	2138~2161	24	miRNA: 3' cccgUCGGAAGU-CUAGGUUCACc 5'<br>:|:| |: |: ||||||| Target:5' gctgGGTCCCCGTGGCCCAAGTGc 3'	-17.90	147.00	?
242	hsa-miR-3615	130~150	21	miRNA: 3' cuCGGCGCUCCUCGGCUCUCu 5' ||:|||: : |||||:| Target:5' ggGCTGCGGCCGCCCGAGGGa 3'	-26.60	143.00	?
243	hsa-miR-3617	2499~2519	21	miRNA: 3' ggguaGAACGUUGAUACAGAAa 5' || ||: ||||||| Target:5' gcccgCTCCCAG-AATGTCTTa 3'	-14.60	144.00	?
244	hsa-miR-3619	1541~1562	22	miRNA: 3' cgacguGGUCGGACGGACGACu 5' ||:|	|||||||| Target:5' tcctatCCGGGCAGCCTGCTGg 3'	-22.90	160.00	?
		2092~2114	23	miRNA: 3' cgaCGUGGUCGGAC-GGACGACu 5' || |||:	||||||| Target:5' gggGCTGGAGCTGGCCCTGCTGc 3'	-21.30	151.00	

<table>
<tr><td></td><td></td><td>1469~1488</td><td>20</td><td>miRNA: 3' cgACGUGGUCGGACGGACGACu 5'
||||||:| |||| ||||
Target:5' acTGCACCGG--TGCCAGCTGt 3'</td><td>-27.00</td><td>144.00</td><td></td></tr>
<tr><td>245</td><td>hsa-miR-362-5p</td><td>1576~1600</td><td>25</td><td>miRNA: 3' ugAGU-GUGGAUCCAAGGUUCCUAa 5'
||| :: | |||| ||:||||
Target:5' ctTCAGTGGCCAGGTCACAGGGATg 3'</td><td>-16.90</td><td>146.00</td><td></td></tr>
<tr><td>246</td><td>hsa-miR-3620</td><td>1600~1623</td><td>24</td><td>miRNA: 3' gacCCACGCCCUAC--GUCCCACu 5'
| |||| :|| :||||||
Target:5' ggaGCTGCGCTGTGCATAGGGTGc 3'</td><td>-18.30</td><td>141.00</td><td></td></tr>
<tr><td rowspan="2">247</td><td rowspan="2">hsa-miR-3622a-3p</td><td>1609~1635</td><td>27</td><td>miRNA: 3' ugucCGUACCCUCC-----AGUCCACu 5'
|||| ||: | |||||||
Target:5' ctgtGCATAGGGTGCCACCTCAGGTGt 3'</td><td>-17.30</td><td>154.00</td><td rowspan="2"></td></tr>
<tr><td>1922~1942</td><td>21</td><td>miRNA: 3' uguCCGUACCCUCCAGUCCACu 5'
||| ||||| ||::||||
Target:5' cctGGC-TGGGAAGTTGGGTGc 3'</td><td>-22.40</td><td>142.00</td></tr>
<tr><td>248</td><td>hsa-miR-3622b-3p</td><td>1610~1635</td><td>26</td><td>miRNA: 3' gucCGUG-CCC----UCGAGUCCACu 5'
|||: ||| | ||||||||
Target:5' tgtGCATAGGGTGCCACCTCAGGTGt 3'</td><td>-20.80</td><td>156.00</td><td></td></tr>
<tr><td>249</td><td>hsa-miR-3646</td><td>2057~2084</td><td>28</td><td>miRNA: 3' accCGACCC-----GAGU-AAAGUAAAa 5'
||| || |||| ||||:|||
Target:5' cgaGCTTGGCCCTCCTCAGTTTCGTTTc 3'</td><td>-11.90</td><td>147.00</td><td></td></tr>
<tr><td>250</td><td>hsa-miR-3648</td><td>2131~2154</td><td>24</td><td>miRNA: 3' ggGAGC-CG--CUAGGGGCGCCGa 5'
|| | || |:||||||:|||
Target:5' ggCTGGAGCTGGGTCCCCGTGGCc 3'</td><td>-27.40</td><td>149.00</td><td></td></tr>
<tr><td rowspan="2">251</td><td rowspan="2">hsa-miR-365*</td><td>40~65</td><td>26</td><td>miRNA: 3' ugUCG-ACGGGGACU---UUCAGGGa 5'
||| ||:|| || |||||||
Target:5' ggAGCGGGCTCCGGAGGGAAGTCCCg 3'</td><td>-24.80</td><td>160.00</td><td rowspan="2"></td></tr>
<tr><td>919~942</td><td>24</td><td>miRNA: 3' ugucGACGGGGACU--UUCAGGGa 5'
:| : |:||: |||||||
Target:5' acttTTATACTTGGGTAAGTCCCc 3'</td><td>-14.20</td><td>148.00</td></tr>
<tr><td>252</td><td>hsa-miR-3651</td><td>1387~1413</td><td>27</td><td>miRNA: 3' agUAC-AUGGUCGCUG--GCCCGAUAc 5'
:|| | || || | ||||||:|
Target:5' ctGTGCTCCCCGCATCTGCGGGCTGTa 3'</td><td>-16.80</td><td>140.00</td><td></td></tr>
</table>

<table>
<tr><td rowspan="2">253</td><td rowspan="2">hsa-miR-3652</td><td>2261~2278</td><td>18</td><td>miRNA: 3' aggagugugGAGGUCGGc 5'
||||||||
Target:5' cgtgtgggaCTCCAGCCg 3'</td><td>-18.90</td><td>145.00</td><td rowspan="2"></td></tr>
<tr><td>2341~2358</td><td>18</td><td>miRNA: 3' aggaGUGUGGAGGUCGGc 5'
||| ||||||||
Target:5' ccacCACCCCTCCAGCga 3'</td><td>-19.80</td><td>142.00</td></tr>
<tr><td>254</td><td>hsa-miR-3657</td><td>1980~2000</td><td>21</td><td>miRNA: 3' uuagugguuauuaCCCUGUGu 5'
|||||||
Target:5' tgggaagccctggGGGACACa 3'</td><td>-15.50</td><td>140.00</td><td></td></tr>
<tr><td rowspan="4">255</td><td rowspan="4">hsa-miR-3661</td><td>1076~1106</td><td>31</td><td>miRNA: 3' guCGAC--AGGCUC--A-----GGGUCCAGu 5'
|||| |::||| | ||||||||
Target:5' gtGCTGCTTTTGAGCCTCAGACCCCAGGTCa 3'</td><td>-22.80</td><td>156.00</td><td rowspan="4"></td></tr>
<tr><td>1482~1512</td><td>31</td><td>miRNA: 3' guCGACAGG---CUCAG------GGUCCAGu 5'
||||||: |:| | |||||||
Target:5' caGCTGTCTTGGGGGCCACAAGGCCAGGTCc 3'</td><td>-27.20</td><td>154.00</td></tr>
<tr><td>1571~1592</td><td>22</td><td>miRNA: 3' gucGACAGGCUCA-GGGUCCAGu 5'
|| |:| ||| |||||||
Target:5' cccCTCTTC-AGTGGCCAGGTCa 3'</td><td>-16.80</td><td>154.00</td></tr>
<tr><td>2240~2261</td><td>22</td><td>miRNA: 3' gucgacaggcucaGGGUCCAGu 5'
:|||||||
Target:5' gagctcggcacccTCCAGGTCc 3'</td><td>-16.60</td><td>141.00</td></tr>
<tr><td rowspan="2">256</td><td rowspan="2">hsa-miR-3663-5p</td><td>1499~1520</td><td>22</td><td>miRNA: 3' ggcUCGUGGUGC--GUCUGGUCg 5'
|| :||| | ||||||||
Target:5' acaAG-GCCAGGTCCAGACCAGg 3'</td><td>-18.30</td><td>155.00</td><td rowspan="2"></td></tr>
<tr><td>1265~1286</td><td>22</td><td>miRNA: 3' ggcUCGUGGU-GCGUCUGGUCg 5'
||| ||| |||:|:||||
Target:5' tgcAGCCCCACCGCGGGCCAGg 3'</td><td>-25.20</td><td>142.00</td></tr>
<tr><td>257</td><td>hsa-miR-3663-3p</td><td>1984~2012</td><td>29</td><td>miRNA: 3' cgCGGG----CC-G-GACACACCACGAGu 5'
|||| || | | | |||||||
Target:5' aaGCCCTGGGGGACACAGCCCGGTGCTCc 3'</td><td>-23.70</td><td>143.00</td><td></td></tr>
<tr><td>258</td><td>hsa-miR-3664</td><td>561~584</td><td>24</td><td>miRNA: 3' ugaguACUCAC--UUCUGUCUCAa 5'
| | || || |||||||
Target:5' acgatTTACTGCCAAAACAGAGTg 3'</td><td>-7.70</td><td>151.00</td><td></td></tr>
</table>

<table>
<tr><td rowspan="3">259</td><td rowspan="3">hsa-miR-3665</td><td>1249~1268</td><td>20</td><td>miRNA: 3' gcGGCGGG--GCGUGGACGa 5'
          || ||| | |||||||
Target:5' gcCCTCCCCACCCACCTGCa 3'</td><td>-21.00</td><td>154.00</td><td rowspan="3"></td></tr>
<tr><td>1389~1406</td><td>18</td><td>miRNA: 3' gcgGCGGGGCGUGGACGa 5'
           | |||||||:||||
Target:5' gtgCTCCCCGCATCTGCg 3'</td><td>-27.80</td><td>151.00</td></tr>
<tr><td>98~115</td><td>18</td><td>miRNA: 3' gcGGCGGGGCGUGGACGa 5'
          | |:||: ||||||:
Target:5' ctCGGTCCTCCACCTGTc 3'</td><td>-18.50</td><td>140.00</td></tr>
<tr><td>260</td><td>hsa-miR-3667-5p</td><td>807~828</td><td>22</td><td>miRNA: 3' ugGAAGAGGAGUUACCCAGAaa 5'
          | |:||| :| ||||||
Target:5' ccCGTTTCCATACAGGGTCTct 3'</td><td>-15.00</td><td>140.00</td><td></td></tr>
<tr><td rowspan="3">261</td><td rowspan="3">hsa-miR-3667-3p</td><td>646~668</td><td>23</td><td>miRNA: 3' uuuCUG-GGUACCUCUCCUUCCa 5'
           |:| :|:| | |||||||
Target:5' tgtGGCATCGTCAAAAGGAAGGg 3'</td><td>-13.20</td><td>151.00</td><td rowspan="3"></td></tr>
<tr><td>1947~1965</td><td>19</td><td>miRNA: 3' uuucuGGGUACCUCUCCUUCCa 5'
               |||| : |||||||
Target:5' cacctCCCA---GCAGGAAGGc 3'</td><td>-15.50</td><td>146.00</td></tr>
<tr><td>182~202</td><td>21</td><td>miRNA: 3' uuucUGGGUACCUCUCCUUCCa 5'
              :|||| ||||||||| |
Target:5' actcGCCCA-GGAGAGGAAAGc 3'</td><td>-21.70</td><td>141.00</td></tr>
<tr><td>262</td><td>hsa-miR-3669</td><td>2589~2614</td><td>26</td><td>miRNA: 3' auAUAAGGCAU--A-UGUAUAAGGCa 5'
          ||||::||| | ||:|||||:|
Target:5' taTATTTTGTAGTTAACGTATTCTGa 3'</td><td>-18.50</td><td>159.00</td><td></td></tr>
<tr><td>263</td><td>hsa-miR-3670</td><td>2395~2419</td><td>25</td><td>miRNA: 3' aucucuuccUGUC-GACACUCGAGa 5'
                   :||| | |||||||
Target:5' acgcaccctGCAGTCACTGAGCTCt 3'</td><td>-14.30</td><td>147.00</td><td></td></tr>
<tr><td>264</td><td>hsa-miR-3673</td><td>274~297</td><td>24</td><td>miRNA: 3' auaaGGCAUAUAU---GUAAGGUa 5'
              |:| : :|: |||||||
Target:5' tgaaCTGGGAGTGCGCCATTCCAg 3'</td><td>-9.20</td><td>141.00</td><td></td></tr>
<tr><td>265</td><td>hsa-miR-3675-5p</td><td>702~724</td><td>23</td><td>miRNA: 3' cuuuagAGAUGUCUUCGGGGUAu 5'
                |||| || ||:||||
Target:5' tgcaaaTCTAAAGTTGCTCCATa 3'</td><td>-16.00</td><td>145.00</td><td></td></tr>
<tr><td>266</td><td>hsa-miR-3678</td><td>1843~1869</td><td>27</td><td>miRNA: 3' ggcCAGGCAUG---UUU--GAGACGUc 5'
           |||| || :|: |||||||</td><td>-16.90</td><td>149.00</td><td></td></tr>
</table>

<table>
<tr><td></td><td>[illegible]
3p</td><td></td><td></td><td>[illegible]
Target:5' gccGTCCTGACGCTGAGCCCTCTGCAa 3'</td><td></td><td></td><td></td></tr>
<tr><td>267</td><td>hsa-miR-3679-5p</td><td>1633~1653</td><td>21</td><td>miRNA: 3' agGGGAAGGGACGGUAUAGGAGu 5'
:|: ||||| ::|:|||||
Target:5' tgTCTGTCCCT--TGTGTCCTCa 3'</td><td>-19.90</td><td>145.00</td><td>?</td></tr>
<tr><td>268</td><td>hsa-miR-3680*</td><td>842~865</td><td>24</td><td>miRNA: 3' ggAUGAGGG-UCCCAGUACGUUUu 5'
||:|::: | ||:|||:|||
Target:5' tgTATTTTTGATTGTTATGTAAAa 3'</td><td>-10.60</td><td>145.00</td><td>?</td></tr>
<tr><td rowspan="2">269</td><td rowspan="2">hsa-miR-3681*</td><td>2806~2827</td><td>22</td><td>miRNA: 3' ucaUCACCUACUUCGUGACAca 5'
| ||||| || ||||||
Target:5' ggcAATGGATTAACCACTGTat 3'</td><td>-12.00</td><td>151.00</td><td rowspan="2">?</td></tr>
<tr><td>1591~1614</td><td>24</td><td>miRNA: 3' ucaUCACCUACUU---CGUGACACa 5'
|| |||||:| ||:|||||
Target:5' cacAG-GGATGGAGCTGCGCTGTGc 3'</td><td>-20.90</td><td>150.00</td></tr>
<tr><td>270</td><td>hsa-miR-3688</td><td>792~818</td><td>27</td><td>miRNA: 3' ucUCACCG-UUUCA---G-AAAGGUAu 5'
| | || :|| | | |||||||
Target:5' cgATTCGCTGAATTGCCCGTTTCCATa 3'</td><td>-9.20</td><td>144.00</td><td>?</td></tr>
<tr><td rowspan="2">271</td><td rowspan="2">hsa-miR-3689a-3p</td><td>1933~1956</td><td>24</td><td>miRNA: 3' uggugCUAUAGU--GUGGAGGGUc 5'
|:|: |: |||||||||
Target:5' agttgGGTGCCGGTCACCTCCCAg 3'</td><td>-23.30</td><td>155.00</td><td rowspan="2">?</td></tr>
<tr><td>1221~1242</td><td>22</td><td>miRNA: 3' uggugcuauagugUGGAGGGUc 5'
:|||||||
Target:5' gtgagaggaggcgGCCTCCCAg 3'</td><td>-18.10</td><td>141.00</td></tr>
<tr><td rowspan="2">272</td><td rowspan="2">hsa-miR-3689b*</td><td>1933~1956</td><td>24</td><td>miRNA: 3' uggugUUAUAGU--GUGGAGGGUc 5'
::|: |: |||||||||
Target:5' agttgGGTGCCGGTCACCTCCCAg 3'</td><td>-21.00</td><td>151.00</td><td rowspan="2">?</td></tr>
<tr><td>1221~1242</td><td>22</td><td>miRNA: 3' ugguguuauagugUGGAGGGUc 5'
:|||||||
Target:5' gtgagaggaggcgGCCTCCCAg 3'</td><td>-17.20</td><td>141.00</td></tr>
<tr><td>273</td><td>hsa-miR-3690</td><td>2205~2227</td><td>23</td><td>miRNA: 3' gaaacagaugcgacCCAGGUCCa 5'
||||||||
Target:5' aatgcagggagggcGGTCCAGGg 3'</td><td>-15.51</td><td>145.00</td><td>?</td></tr>
<tr><td>274</td><td>hsa-miR-3692*</td><td>1937~1960</td><td>24</td><td>miRNA: 3' guCAUAGGU--GAGGACUGGUCGUCc 5'
||: ||: | ||| |||||||
Target:5' ggGTG-CCGGTCACCT-CCCAGCAGg 3'</td><td>-23.50</td><td>150.00</td><td>?</td></tr>
</table>

<table>
<tr><td>275</td><td>hsa-miR-3692</td><td>2248~2270</td><td>23</td><td>miRNA: 3' ugaaGACGUCACAGUCACACCUUg 5'
|| ||| ||| ||||||:|
Target:5' caccCTCCAG-GTCCGTGTGGGAc 3'</td><td>-22.40</td><td>155.00</td><td></td></tr>
<tr><td rowspan="3">276</td><td rowspan="3">hsa-miR-370</td><td>1940~1961</td><td>22</td><td>miRNA: 3' ugGUCCAAGGUGGGGUCGUCCg 5'
| ||| | :|||||||||
Target:5' tgCCGGTCACCTCCCAGCAGGa 3'</td><td>-28.00</td><td>164.00</td><td rowspan="3"></td></tr>
<tr><td>161~183</td><td>23</td><td>miRNA: 3' ugGUCCAAGGUGGG-GUCGUCcg 5'
|:|| :|:||| ||||||
Target:5' gtCGGGGATCGCCCTCAGCAGac 3'</td><td>-27.60</td><td>144.00</td></tr>
<tr><td>1822~1845</td><td>24</td><td>miRNA: 3' uggUCC-A-AGGUGGGGUCGUCcg 5'
||| | | |:||:||||||
Target:5' gggAGGCTGTGCGCCTCAGCAGcc 3'</td><td>-23.20</td><td>143.00</td></tr>
<tr><td rowspan="2">277</td><td rowspan="2">hsa-miR-3714</td><td>1065~1086</td><td>22</td><td>miRNA: 3' uguccccUCGUGACGACGGAAg 5'
|:|| ||||||:||
Target:5' gaacctGGGCTGTGCTGCTTTt 3'</td><td>-16.30</td><td>144.00</td><td rowspan="2"></td></tr>
<tr><td>1472~1492</td><td>21</td><td>miRNA: 3' uguccCCUCGUGACGACGGAAg 5'
|| || | ||||:|||
Target:5' gcaccGGTGC-CAGCTGTCTTg 3'</td><td>-14.30</td><td>140.00</td></tr>
<tr><td>278</td><td>hsa-miR-373*</td><td>1071~1090</td><td>20</td><td>miRNA: 3' ccuuuCGCGGGGGUAAAACUCa 5'
|:||: | |||||||
Target:5' gggctGTGCTGC--TTTTGAGc 3'</td><td>-14.40</td><td>149.00</td><td></td></tr>
<tr><td>279</td><td>hsa-miR-373</td><td>2831~2853</td><td>23</td><td>miRNA: 3' ugUGGGGUUUUAGCUUCGUGAAg 5'
| :||| ||: |||||:||
Target:5' agAATCCACCATTAAAGCATTTg 3'</td><td>-10.80</td><td>149.00</td><td></td></tr>
<tr><td>280</td><td>hsa-miR-376c</td><td>1140~1160</td><td>21</td><td>miRNA: 3' ugcACCUUAAAGGAGAUACAa 5'
||| :|||: |||:|||
Target:5' ataTGGCGTTTTGTCTGTGTt 3'</td><td>-15.10</td><td>150.00</td><td></td></tr>
<tr><td>281</td><td>hsa-miR-377*</td><td>2004~2028</td><td>25</td><td>miRNA: 3' cuUAAGUGGUUC---CCGUUGGAGa 5'
:| | || || |||||||
Target:5' cgGTGCTCCCAGCCCTCCAACCTCt 3'</td><td>-14.00</td><td>144.00</td><td></td></tr>
<tr><td>282</td><td>hsa-miR-378*</td><td>1425~1449</td><td>25</td><td>miRNA: 3' uguGUCCUGGACCU---CAGUCCUc 5'
:| ||:||| | |||:|||
Target:5' agcTAAGATCTGCATAGGTCGGGAt 3'</td><td>-15.90</td><td>143.00</td><td></td></tr>
</table>

283	hsa-miR-381	1628~1649	22	miRNA: 3' ugUCUCUCGAACGGGAACAUAu 5'<br>||: : || ||||||:| Target:5' tcAGGTGTCTGTCCCTTGTGTc 3'	-16.80	144.00	
284	hsa-miR-3909	2155~2178	24	miRNA: 3' ucUGACGUCC--GGGAUCUCCUGu 5'<br>| |||||| ||| |||||:| Target:5' caAGTGCAGGGTCCCAAGAGGGCa 3'	-26.70	158.00	
		1977~1998	22	miRNA: 3' ucUGACGUCCGGGAUCUCCUGu 5'<br>|: | | |||||:|:|||| Target:5' agATGGGAAGCCCTGGGGGACa 3'	-26.20	140.00	
285	hsa-miR-3913	2149~2172	24	miRNA: 3' ucuguaGUUCUAGU--CAGGGUUu 5'<br>|||| || ||||||| Target:5' gtggccCAAGTGCAGGGTCCCAAg 3'	-15.00	154.00	
		224~244	21	miRNA: 3' ucuguAGUUCUAGUCAGGGUUu 5'<br>||: |: | ||||||| Target:5' tggttTCGTGG-CTGTCCCAAc 3'	-12.30	148.00	
		2149~2172	24	miRNA: 3' ucuguaGUUCUAGU--CAGGGUUu 5'<br>|||| || ||||||| Target:5' gtggccCAAGTGCAGGGTCCCAAg 3'	-15.00	154.00	
		224~244	21	miRNA: 3' ucuguAGUUCUAGUCAGGGUUu 5'<br>||: |: | ||||||| Target:5' tggttTCGTGG-CTGTCCCAAc 3'	-12.30	148.00	
286	hsa-miR-3917	588~607	20	miRNA: 3' ggguggacgagUCAGGCUCg 5'<br>:||||||| Target:5' tacgagaaaagGGTCCGAGc 3'	-15.84	141.00	
287	hsa-miR-3918	2093~2111	19	miRNA: 3' ucaGAGGUAGACGCCGGGACa 5'<br>|| | || ||||||| Target:5' gggCTGGAGCT--GGCCCTGc 3'	-16.90	146.00	
		2217~2233	17	miRNA: 3' ucagAGGUAGACGCCGGGACa 5'<br>|||| | ||||||| Target:5' gcggTCCA---G-GGCCCTGg 3'	-16.50	145.00	
		1744~1766	23	miRNA: 3' ucaGAGGUAGA-C-GCCGGGACa 5'<br>| ||| | | ||||||| Target:5' tagCGCCAGGTAGAGGGCCCTGg 3'	-16.30	142.00	
		[illegible]	[illegible]	miRNA: 3' uuUCUCAGUUCAGUUCCGGUCu 5'	[illegible]	[illegible]	

<table>
<tr><td rowspan="2">288</td><td rowspan="2">hsa-miR-3922</td><td>1491~1509</td><td>19</td><td>   :|:| || |||||||||
Target:5' tgGGGGCCA---CAAGGCCAGg 3'</td><td>-19.60</td><td>157.00</td><td rowspan="2"></td></tr>
<tr><td>450~475</td><td>26</td><td>miRNA: 3' uuUCU-CAGUUC---AGUUCCGGUCu 5'
            :|| | |||| | :|||||||
Target:5' gaGGAGGACAAGGACTGGAGGCCAGc 3'</td><td>-20.00</td><td>156.00</td></tr>
<tr><td>289</td><td>hsa-miR-3924</td><td>2544~2578</td><td>35</td><td>miRNA: 3' ucAUCGUCA--GUGUA-----------UAUGUAUa 5'
            || :||| ||||| |||||||
Target:5' agTAATAGTTACACATGTATATGGTTAATACATAt 3'</td><td>-16.15</td><td>150.00</td><td></td></tr>
<tr><td rowspan="2">290</td><td rowspan="2">hsa-miR-3928</td><td>931~959</td><td>29</td><td>miRNA: 3' cggcUUC--GAGGUUC-----CAAGGAGg 5'
              ||| | |||:| |||||||
Target:5' gggtAAGTCCCCCAGGGGCGAGTTCCTCg 3'</td><td>-17.40</td><td>152.00</td><td rowspan="2"></td></tr>
<tr><td>2610~2632</td><td>23</td><td>miRNA: 3' cgGCUUCG-AGGUUCCAAGGAGg 5'
            :||||: | :| |||:|||
Target:5' tcTGAAGTAACGGATGTTTCTCg 3'</td><td>-16.20</td><td>140.00</td></tr>
<tr><td rowspan="2">291</td><td rowspan="2">hsa-miR-3937</td><td>1513~1536</td><td>24</td><td>miRNA: 3' gggGGUAACGA-UGUCGGCGGACa 5'
             ||| ||| : :||:|||||
Target:5' agaCCAGGGCTGGGGGCTGCCTGa 3'</td><td>-24.20</td><td>144.00</td><td rowspan="2"></td></tr>
<tr><td>124~146</td><td>23</td><td>miRNA: 3' gggGGUAACGAUGUCGGCGGACa 5'
             ||: |||:|:|||||| |
Target:5' tcgCCGGGGCTGCGGCCGCCCGa 3'</td><td>-31.12</td><td>140.00</td></tr>
<tr><td>292</td><td>hsa-miR-3939</td><td>20~40</td><td>21</td><td>miRNA: 3' cuguAGGACACCAGACGCGCau 5'
              | || ||| |||||||
Target:5' gagcTGCTCTGG-CTGCGCGcg 3'</td><td>-17.80</td><td>141.00</td><td></td></tr>
<tr><td>293</td><td>hsa-miR-3940</td><td>1533~1554</td><td>22</td><td>miRNA: 3' uuCACCCGACCCUAGGCCCGac 5'
            | || || ||||||||
Target:5' ctGAGGACTCCTATCCGGGCag 3'</td><td>-17.50</td><td>140.00</td><td></td></tr>
<tr><td rowspan="3">294</td><td rowspan="3">hsa-miR-3943</td><td>1509~1531</td><td>23</td><td>miRNA: 3' gcGGUUCACUUCGGACCCCCGAu 5'
            |||: |:| |||||||||
Target:5' gtCCAGACCAGGGCTGGGGGCTg 3'</td><td>-27.50</td><td>165.00</td><td rowspan="3"></td></tr>
<tr><td>1479~1499</td><td>21</td><td>miRNA: 3' gcGGUUCACUUCGGACCCCCGau 5'
            |||: || |:|||||||
Target:5' tgCCAGCTG--TCTTGGGGGCca 3'</td><td>-25.20</td><td>141.00</td></tr>
<tr><td>2076~2098</td><td>23</td><td>miRNA: 3' gcGGUUCACUUCGGACCCCCGAu 5'
            :|: | |||||| ||||||
Target:5' ttTCGTTTCAAGCCTTGGGGCTg 3'</td><td>-22.90</td><td>141.00</td></tr>
</table>

<table>
<tr><td></td><td></td><td></td><td></td><td>[illegible]</td><td></td><td></td><td></td></tr>
<tr><td>295</td><td>hsa-miR-3944</td><td>127~147</td><td>21</td><td>miRNA: 3' ggCCUCGUCGUCCGGUCGGGCUu 5'
||:|| ||:| | ||||||
Target:5' ccGGGGCTGCGGCC--GCCCGAg 3'</td><td>-37.50</td><td>140.00</td><td></td></tr>
<tr><td>296</td><td>hsa-miR-409-3p</td><td>669~700</td><td>32</td><td>miRNA: 3' uccCCAAGUGGCUC----------GUUGUAAg 5'
||||:: | || |||||||
Target:5' attGGTTTGGCAAGAACTTGTTTACAACATTt 3'</td><td>-10.50</td><td>145.00</td><td></td></tr>
<tr><td>297</td><td>hsa-miR-423-5p</td><td>1027~1051</td><td>25</td><td>miRNA: 3' uuucaGAGCGA-GA-GACGGGGAGu 5'
||| || || | ||:||||
Target:5' gcaccCTCCCTCCTGCCGCTCCTCt 3'</td><td>-19.80</td><td>142.00</td><td></td></tr>
<tr><td rowspan="2">298</td><td rowspan="2">hsa-miR-424</td><td>1061~1084</td><td>24</td><td>miRNA: 3' aaGUUUUGUACUU-A-ACGACGAc 5'
:|:||| ||:: | |||||||
Target:5' tcTAGAACCTGGGCTGTGCTGCTt 3'</td><td>-15.10</td><td>160.00</td><td rowspan="2"></td></tr>
<tr><td>1354~1380</td><td>27</td><td>miRNA: 3' aaGUUUUG-UAC----UUAACGACGAc 5'
||:::| :|| || |||||||
Target:5' aaCAGGGCTGTGTACAAAGTGCTGCTg 3'</td><td>-18.20</td><td>154.00</td></tr>
<tr><td>299</td><td>hsa-miR-4252</td><td>1568~1586</td><td>19</td><td>miRNA: 3' accacgacuGAGUCACCGg 5'
:||||||||
Target:5' gttcccctcTTCAGTGGCc 3'</td><td>-16.60</td><td>146.00</td><td></td></tr>
<tr><td rowspan="2">300</td><td rowspan="2">hsa-miR-4254</td><td>2232~2259</td><td>28</td><td>miRNA: 3' cuCUACCACCUC-AUC-----GAGGUCCg 5'
|| || |||| | | |||||||
Target:5' ggGAAGG-GGAGCTCGGCACCCTCCAGGt 3'</td><td>-22.10</td><td>152.00</td><td rowspan="2"></td></tr>
<tr><td>1706~1730</td><td>25</td><td>miRNA: 3' cucuaccaccuCAUC--GAGGUCCg 5'
||:| |||||||
Target:5' agcccacagccGTGGCCCTCCAGGg 3'</td><td>-16.20</td><td>146.00</td></tr>
<tr><td>301</td><td>hsa-miR-4257</td><td>2011~2030</td><td>20</td><td>miRNA: 3' gaGUCAGGGG--UGGAGACc 5'
||| ||:| |||||||
Target:5' ccCAGCCCTCCAACCTCTGg 3'</td><td>-23.20</td><td>158.00</td><td></td></tr>
<tr><td>302</td><td>hsa-miR-4258</td><td>1550~1567</td><td>18</td><td>miRNA: 3' gguUCCG-CCACCGCCCc 5'
|| | | |||||||
Target:5' ggcAGCCTGCTGGCGGGg 3'</td><td>-20.70</td><td>146.00</td><td></td></tr>
<tr><td rowspan="2">303</td><td rowspan="2">hsa-miR-</td><td>525~546</td><td>22</td><td>miRNA: 3' aggacugGGGAUCUGGGUUGAc 5'
:|| |||||||:||
Target:5' ccaaataTCCAAGACCCAGCTc 3'</td><td>-19.60</td><td>147.00</td><td rowspan="2"></td></tr>
<tr><td></td><td></td><td></td><td></td><td></td></tr>
</table>

	4259	2018~2040	23	miRNA: 3' aggacUGGGGAUC-UGGGUUGac 5'<br>|||:||:| |||||| Target:5' ctccaACCTCTGGCTCCCAACcc 3'	-21.30	141.00	
304	hsa-miR-4265	1697~1714	18	miRNA: 3' ggGUCUCGACUCGGGUGUc 5'<br>| |:| ||||||||| Target:5' ttCTGGG-AGAGCCCACAg 3'	-23.30	152.00	
		544~562	19	miRNA: 3' ggGUCUCGACU-CGGGUGUc 5'<br>|| ||| || |||:||| Target:5' ctCA-AGCAGAGGCCTACAc 3'	-14.20	140.00	
305	hsa-miR-4267	2093~2106	14	miRNA: 3' caCGGUGGCUCGACCu 5'<br>||:: ||||||| Target:5' ggGCTG--GAGCTGGc 3'	-12.10	142.00	
		2127~2143	17	miRNA: 3' caCGGUGG-CUCGACCu 5'<br>|: :|: ||||||| Target:5' cgGTGGCTGGAGCTGGg 3'	-17.00	142.00	
306	hsa-miR-4268	2224~2244	21	miRNA: 3' guguaGGACUCUCCUCCUCGg 5'<br>||||:|| |:||||| Target:5' agggcCCTGGGAAGGGGAGCt 3'	-23.20	152.00	
307	hsa-miR-4269	422~441	20	miRNA: 3' cgGUCCC-GACAGACACGGACg 5'<br>|:||| | | |||||||| Target:5' ttCGGGGACAG--TGTGCCTGt 3'	-22.70	155.00	
		1761~1781	21	miRNA: 3' cgGUCCCGACAGACACGGACg 5'<br>| |||| | |||||:||| Target:5' ccCTGGGCAGCCTGTGTCTGg 3'	-25.10	155.00	
308	hsa-miR-4271	1720~1738	19	miRNA: 3' ggGGUGGAAAAGAAGGGGg 5'<br>|| || ||||||| Target:5' gcCCTCCAGGGCTTCCCCg 3'	-21.00	145.00	
309	hsa-miR-4273	438~460	23	miRNA: 3' gaCAGGUAG-----UCUCUUGUg 5'<br>||||||| :|||:||| Target:5' ctGTCCATCTTAGAGGAGGACAa 3'	-21.20	144.00	
310	hsa-miR-4276	2398~2415	18	miRNA: 3' cguGUAC-UCAGUGACUc 5'<br>| || ||||||||| Target:5' cacCCTGCAGTCACTGAg 3'	-17.80	154.00	
	hsa-			[illegible]			

311	miR-4277	980~998	19	miRNA: 3' cacaUGACACGAGUCUUGACg 5'<br>|| ||| ||||:||| Target:5' tctcACCGTG--CAGAGCTGc 3'	-15.40	141.00	
312	hsa-miR-4279	2707~2722	16	miRNA: 3' cuucggccCUCCUCUc 5'<br>||||||| Target:5' ccttgtaaGAGGAGAg 3'	-13.90	140.00	
313	hsa-miR-4280	982~1002	21	miRNA: 3' cgaGACGAGUCUUGAUGUGAg 5'<br>| |: ||||:||:|||| Target:5' tcaCCGTGCAGAGCTGCACTt 3'	-18.20	150.00	
		2519~2543	25	miRNA: 3' cgAGACG--A--GUCUUGAUGUGAg 5'<br>|:||: | | ||||||||:| Target:5' atTTTGTAATGACTGAACTACATTt 3'	-13.50	143.00	
314	hsa-miR-4283	1848~1864	17	miRNA: 3' uuugaGCGACUCGGGGu 5'<br>||||||||||:<br>Target:5' cctgaCGCTGAGCCCTc 3'	-21.90	144.00	
		1848~1864	17	miRNA: 3' uuugaGCGACUCGGGGu 5'<br>||||||||||:<br>Target:5' cctgaCGCTGAGCCCTc 3'	-21.90	144.00	
315	hsa-miR-4285	109~130	22	miRNA: 3' uacuCAGCC----UGAGCGGCg 5'<br>||| | :||||||| Target:5' acctGTCCGCTACGCTCGCCGg 3'	-14.70	144.00	
316	hsa-miR-4287	2222~2243	22	miRNA: 3' uuUCACGGGA---GUUCCCUCu 5'<br>|| ||||| |:||||| Target:5' ccAGGGCCCTGGGAAGGGGAGc 3'	-19.20	141.00	
317	hsa-miR-4288	169~185	17	miRNA: 3' ccuuuGAGUCGUCUGuu 5'<br>|||||||||| Target:5' tcgccCTCAGCAGACtc 3'	-16.00	140.00	
318	hsa-miR-429	872~898	27	miRNA: 3' ugccAAAAUGGU--CU---GUCAUAAu 5'<br>||||| :| || ||||||| Target:5' tttaTTTTAATATTGATGTCAGTATTt 3'	-7.70	156.00	
319	hsa-miR-4290	2200~2219	20	miRNA: 3' cuccCUU-CUUUCCUCCCGu 5'<br>||| | |:||||||| Target:5' caaaGAATGCAGGGAGGGCg 3'	-18.40	155.00	
320	hsa-miR-[illegible]	1367~1382	16	miRNA: 3' ucgacaAGGACGACUu 5'<br>| ||||||| [illegible]	-11.50	142.00	

<table>
<tr><td></td><td>4291</td><td></td><td></td><td>Target:5' acaaagTGCTGCTGAg 3'</td><td></td><td></td><td></td></tr>
<tr><td rowspan="2">321</td><td rowspan="2">hsa-miR-4292</td><td>933~948</td><td>16</td><td>miRNA: 3' ggUUCCGGCCGGGUCCCc 5'
||| || |||||||
Target:5' gtAAGTCC--CCCAGGGg 3'</td><td>-22.20</td><td>152.00</td><td rowspan="2"></td></tr>
<tr><td>2211~2228</td><td>18</td><td>miRNA: 3' ggUUCCGGCCGGGUCCCc 5'
:||| |||:||||||
Target:5' ggGAGGGCGGTCCAGGGc 3'</td><td>-27.40</td><td>152.00</td></tr>
<tr><td>322</td><td>hsa-miR-4294</td><td>1527~1544</td><td>18</td><td>miRNA: 3' ggGAC-GACAUCUGAGGg 5'
||| ||| :||||||
Target:5' ggCTGCCTGAGGACTCCt 3'</td><td>-17.90</td><td>143.00</td><td></td></tr>
<tr><td>323</td><td>hsa-miR-4296</td><td>1698~1714</td><td>17</td><td>miRNA: 3' acucggaCUCGGGUGUa 5'
|||||||||
Target:5' tctgggaGAGCCCACAg 3'</td><td>-18.00</td><td>150.00</td><td></td></tr>
<tr><td>324</td><td>hsa-miR-4297</td><td>1952~1966</td><td>15</td><td>miRNA: 3' guGUCUGUCCUUCCGu 5'
||| |||||||||
Target:5' ccCAG-CAGGAAGGCa 3'</td><td>-20.00</td><td>157.00</td><td></td></tr>
<tr><td rowspan="2">325</td><td rowspan="2">hsa-miR-4298</td><td>224~243</td><td>20</td><td>miRNA: 3' gaCGGAGGAGGAGGACAGGGUc 5'
|::|| | ||||||||
Target:5' tgGTTTCGT--GGCTGTCCCAa 3'</td><td>-16.70</td><td>148.00</td><td rowspan="2"></td></tr>
<tr><td>1620~1644</td><td>25</td><td>miRNA: 3' gaCGGAGGAG---GAGGACAGGGuc 5'
||| |||| : :|||||||
Target:5' gtGCCACCTCAGGTGTCTGTCCCtt 3'</td><td>-26.10</td><td>144.00</td></tr>
<tr><td>326</td><td>hsa-miR-4304</td><td>149~165</td><td>17</td><td>miRNA: 3' acGGGACCUGUACGGCc 5'
|::|| |||||:||
Target:5' gaCTTTGAACATGTCGg 3'</td><td>-20.30</td><td>143.00</td><td></td></tr>
<tr><td rowspan="2">327</td><td rowspan="2">hsa-miR-4308</td><td>2211~2228</td><td>18</td><td>miRNA: 3' uuCUUCUUUGAGGUCCCu 5'
||:|: : |||||||
Target:5' ggGAGGGCGGTCCAGGGc 3'</td><td>-19.30</td><td>152.00</td><td rowspan="2"></td></tr>
<tr><td>1714~1731</td><td>18</td><td>miRNA: 3' uucuucuuuGAGGUCCCu 5'
||||||||
Target:5' gccgtggccCTCCAGGGc 3'</td><td>-14.80</td><td>145.00</td></tr>
<tr><td></td><td>hsa-</td><td>2254~2275</td><td>22</td><td>miRNA: 3' accUUAGG---A-UCUGAGGUc 5'
::||| | :|||||||
Target:5' ccaGGTCCGTGTGGGACTCCAg 3'</td><td>-18.60</td><td>143.00</td><td></td></tr>
</table>

<table>
<tr><td>328</td><td>miR-4309</td><td>2632~2651</td><td>20</td><td>miRNA: 3' accUUAGGAU--CUGAGGUc 5'
|||| || ||||:||
Target:5' gccAATCGTAGTGACTTCAg 3'</td><td>-13.10</td><td>141.00</td><td></td></tr>
<tr><td>329</td><td>hsa-miR-431*</td><td>622~646</td><td>25</td><td>miRNA: 3' ucuuCGGGA-CGUUC--UGCUGGAc 5'
||||| :||| :||||||
Target:5' ttgcGCCCTCATAAGCAGCGACCTt 3'</td><td>-18.10</td><td>144.00</td><td></td></tr>
<tr><td>330</td><td>hsa-miR-4312</td><td>1491~1506</td><td>16</td><td>miRNA: 3' acCCCUGUCCUUGUUCCGg 5'
|||:| |||||||
Target:5' tgGGGGC---CACAAGGCc 3'</td><td>-23.20</td><td>146.00</td><td></td></tr>
<tr><td>331</td><td>hsa-miR-4314</td><td>2496~2512</td><td>17</td><td>miRNA: 3' gacaGGGUAAAGGGUCUc 5'
|||: |||||||
Target:5' gtagCCCG-CTCCCAGAa 3'</td><td>-16.40</td><td>145.00</td><td></td></tr>
<tr><td>332</td><td>hsa-miR-4317</td><td>2797~2813</td><td>17</td><td>miRNA: 3' uuUGAGGGACCGUUACa 5'
| |: : |||||||
Target:5' agAATTGTGGGCAATGg 3'</td><td>-10.20</td><td>143.00</td><td></td></tr>
<tr><td>333</td><td>hsa-miR-432</td><td>1705~1730</td><td>26</td><td>miRNA: 3' ggUGGGU-----UACUGGAUGAGGUUCu 5'
:|||| :||:|| |||||:|
Target:5' gaGCCCACAGCCGTGGCC--CTCCAGGg 3'</td><td>-24.10</td><td>141.00</td><td></td></tr>
<tr><td rowspan="2">334</td><td rowspan="2">hsa-miR-432*</td><td>199~222</td><td>24</td><td>miRNA: 3' ucUGUACCUCC--UC-GGUAGGUc 5'
:|||||||| || ||| |||
Target:5' aaGCATGGAGGAAAGACCACCCAt 3'</td><td>-20.20</td><td>141.00</td><td rowspan="2"></td></tr>
<tr><td>461~483</td><td>23</td><td>miRNA: 3' ucUGUACCUCC--UCGGUAG-GUc 5'
|| |||||| ||||||| ||
Target:5' ggAC-TGGAGGCCAGCCATCACAa 3'</td><td>-23.44</td><td>140.00</td></tr>
<tr><td>335</td><td>hsa-miR-4320</td><td>2822~2838</td><td>17</td><td>miRNA: 3' ucCUUCGAUGUCUUAGGg 5'
| | ||| |||||||
Target:5' ctGTATCTA-AGAATCCa 3'</td><td>-10.20</td><td>151.00</td><td></td></tr>
<tr><td>336</td><td>hsa-miR-4322</td><td>1684~1714</td><td>31</td><td>miRNA: 3' ggGGUGCGCGA----------CUCGGGUGUc 5'
||| |:||| |||||||||
Target:5' cgCCAGGTGCTTTTTCTGGGAGAGCCCACAg 3'</td><td>-29.60</td><td>157.00</td><td></td></tr>
<tr><td rowspan="2">337</td><td rowspan="2">hsa-miR-4323</td><td>2082~2098</td><td>17</td><td>miRNA: 3' agacUCCGACACCCCGAc 5'
|| || |||||||
Target:5' ttcaAGCCT-TGGGGCTg 3'</td><td>-16.80</td><td>149.00</td><td rowspan="2"></td></tr>
<tr><td></td><td></td><td></td><td></td><td></td></tr>
</table>

<table>
<tr><td></td><td></td><td>1880~1899</td><td>20</td><td>miRNA: 3' agacUCC-GA-CACCCCGAc 5'
||| || |||||||
Target:5' ggccAGGCCTCTTGGGGCTg 3'</td><td>-19.30</td><td>146.00</td><td></td></tr>
<tr><td>338</td><td>hsa-miR-4325</td><td>2146~2163</td><td>18</td><td>miRNA: 3' agugACUCUGUUCACGUu 5'
||: ||||||||
Target:5' cccgTGGCCCAAGTGCAg 3'</td><td>-13.00</td><td>150.00</td><td></td></tr>
<tr><td>339</td><td>hsa-miR-449a</td><td>548~574</td><td>27</td><td>miRNA: 3' ugGUC----GAU-UGUUAUGUGACGGu 5'
||| ||| ||:|| :||||||
Target:5' agCAGAGGCCTACACGATTTACTGCCa 3'</td><td>-19.80</td><td>150.00</td><td></td></tr>
<tr><td>340</td><td>hsa-miR-449b</td><td>548~574</td><td>27</td><td>miRNA: 3' cgGUC----GAU-UGUUAUGUGACGGa 5'
||| ||| ||:|| :||||||
Target:5' agCAGAGGCCTACACGATTTACTGCCa 3'</td><td>-17.50</td><td>150.00</td><td></td></tr>
<tr><td>341</td><td>hsa-miR-449c</td><td>551~575</td><td>25</td><td>miRNA: 3' ugUCGGCGAU-CGUUAUGUGACGGau 5'
|| | ||| |:|| :||||||
Target:5' agAGGC-CTACACGATTTACTGCCaa 3'</td><td>-15.80</td><td>142.00</td><td></td></tr>
<tr><td>342</td><td>hsa-miR-450b-5p</td><td>687~708</td><td>22</td><td>miRNA: 3' auAAGUCCUUGU-AUAACGUUUu 5'
||:| |||| | |||||||
Target:5' tgTTTA-CAACATTTTTGCAAAt 3'</td><td>-8.50</td><td>159.00</td><td></td></tr>
<tr><td>343</td><td>hsa-miR-450b-3p</td><td>478~498</td><td>21</td><td>miRNA: 3' auaccUACGUUUUACUAGGGUu 5'
|| |||| |||||:|
Target:5' tcacaAT-CAAACAGATCCTAt 3'</td><td>-8.90</td><td>140.00</td><td></td></tr>
<tr><td>344</td><td>hsa-miR-452</td><td>1339~1360</td><td>22</td><td>miRNA: 3' agucaaaGGAGACGUUUGUCaa 5'
:||:||:||||||
Target:5' ccctggaTCTTTGTAAACAGgg 3'</td><td>-15.20</td><td>143.00</td><td></td></tr>
<tr><td rowspan="2">345</td><td rowspan="2">hsa-miR-485-5p</td><td>1644~1666</td><td>23</td><td>miRNA: 3' cuuaAGUAGUGC-CGGUCGGAGa 5'
|| ||| | | |||||:
Target:5' tgtgTCCTCAGGAGGCAGCCTTg 3'</td><td>-14.90</td><td>142.00</td><td rowspan="2"></td></tr>
<tr><td>1537~1559</td><td>23</td><td>miRNA: 3' cuUAAG--UAGUGCCGGUCGGAga 5'
| || ||| ||| ||||||
Target:5' ggACTCCTATC-CGGGCAGCCTgc 3'</td><td>-19.00</td><td>141.00</td></tr>
<tr><td>346</td><td>hsa-miR-486-5p</td><td>2146~2165</td><td>20</td><td>miRNA: 3' gaGCCCCGUCGAGUCAUGUCCu 5'
|| ||| | |||:||||
Target:5' ccCGTGGC--CCAAGTGCAGGg 3'</td><td>-18.40</td><td>140.00</td><td></td></tr>
<tr><td>[illegible]</td><td>hsa-[illegible]</td><td>[illegible]</td><td>[illegible]</td><td>miRNA: 3' uagGACAU-GACU----CGACGGGGc 5'
[illegible]</td><td>[illegible]</td><td>[illegible]</td><td></td></tr>
</table>

<table>
<tr><td>347</td><td>miR-486-3p</td><td>2093~2118</td><td>26</td><td>miRNA: 3' [illegible] 5'
||| | |||: |||||||:
Target:5' gggCTGGAGCTGGCCCTGCTGCCCTg 3'</td><td>-23.60</td><td>140.00</td><td>?</td></tr>
<tr><td>348</td><td>hsa-miR-489</td><td>873~893</td><td>21</td><td>miRNA: 3' cgacggcaUAUACACUACAGUg 5'
|||| ||||||||
Target:5' ttattttaATAT-TGATGTCAg 3'</td><td>-9.90</td><td>157.00</td><td>?</td></tr>
<tr><td>349</td><td>hsa-miR-490-3p</td><td>2240~2261</td><td>22</td><td>miRNA: 3' guCGUACCUCAGGAGGUCCAac 5'
|| || |||||||||
Target:5' gaGCTCGGCACCCTCCAGGTcc 3'</td><td>-21.40</td><td>140.00</td><td>?</td></tr>
<tr><td rowspan="2">350</td><td rowspan="2">hsa-miR-491-5p</td><td>2174~2195</td><td>22</td><td>miRNA: 3' ggaGUACCUUCCCAAGGGGUga 5'
|| || :||| ||||||
Target:5' gggCAGGGCGGGGCTCCCCAaa 3'</td><td>-21.20</td><td>147.00</td><td rowspan="2">?</td></tr>
<tr><td>1239~1260</td><td>22</td><td>miRNA: 3' ggaguaccuucccaAGGGGUGa 5'
|||||||
Target:5' ccagtgcccggcccTCCCCACc 3'</td><td>-13.80</td><td>140.00</td></tr>
<tr><td rowspan="2">351</td><td rowspan="2">hsa-miR-492</td><td>2237~2262</td><td>26</td><td>miRNA: 3' uuCUUAGAAC---AGGGCGUCCAGGa 5'
|:| || | ||: |||||||
Target:5' ggGGAGCTCGGCACCCTCCAGGTCCg 3'</td><td>-18.50</td><td>153.00</td><td rowspan="2">?</td></tr>
<tr><td>1483~1513</td><td>31</td><td>miRNA: 3' uucuUAGAA---CAGG-G---C-GUCCAGGa 5'
:|||| | || | | |||||||
Target:5' agctGTCTTGGGGGCCACAAGGCCAGGTCCa 3'</td><td>-21.70</td><td>143.00</td></tr>
<tr><td>352</td><td>hsa-miR-493*</td><td>2544~2565</td><td>22</td><td>miRNA: 3' uuACUUUCGGAUG-GUACAUGUu 5'
| | || :||| ||||||:|
Target:5' agTAATAG-TTACACATGTATAt 3'</td><td>-10.30</td><td>143.00</td><td>?</td></tr>
<tr><td rowspan="2">353</td><td rowspan="2">hsa-miR-494</td><td>2603~2630</td><td>28</td><td>miRNA: 3' cucCAAAGGG---CA---CAUACAAAGu 5'
|| |:|: || | |||||||
Target:5' aacGTATTCTGAAGTAACGGATGTTTCt 3'</td><td>-8.60</td><td>147.00</td><td rowspan="2">?</td></tr>
<tr><td>2419~2441</td><td>23</td><td>miRNA: 3' cuCCAAAGGGCACA-UACAAAgu 5'
||| | ||| | ||||||
Target:5' tcGGTCTGACGTCTGATGTTTgt 3'</td><td>-7.90</td><td>140.00</td></tr>
<tr><td>354</td><td>hsa-miR-496</td><td>849~869</td><td>21</td><td>miRNA: 3' cuCUAACCGGUACAUUAUGAGu 5'
|||| |::|||||| ||||
Target:5' ttGATT-GTTATGTAAAACTCg 3'</td><td>-16.10</td><td>147.00</td><td>?</td></tr>
<tr><td></td><td>[illegible]</td><td>1063~1084</td><td>22</td><td>miRNA: 3' ugUUUGGUGUC-ACACGACGAc 5'
:|||| :| |||||||||
Target:5' taGAACCTGGGCTGTGCTGCTt 3'</td><td>-17.90</td><td>163.00</td><td></td></tr>
</table>

355	hsa-miR-497						
		[1360~1380]	21	miRNA: 3' uguuugGUGUCACACGACGAc 5'<br>:||| |||||||| Target:5' gctgtgTACAAAGTGCTGCTg 3'	-18.60	155.00	
356	hsa-miR-497*	[2423~2447]	25	miRNA: 3' agAUUGUGG--UGU-CACACCAAAc 5'<br>|:||::| |:: |||||||| Target:5' tcTGACGTCTGATGTTTGTGGTTTg 3'	-20.00	154.00	
		[1792~1816]	25	miRNA: 3' agAUUGUGGUG---UCACACCAAAc 5'<br>|:| :|||| |||||||| | Target:5' ccTGAGGCCACCTGAGTGTGGTCTg 3'	-23.70	140.00	
357	hsa-miR-498	[2470~2491]	22	miRNA: 3' cuUUUUGCGGGGGACCGAACUUu 5'<br>::||: :| ||||:||||| Target:5' ggGGAAT-TCAGCTGGTTTGAAt 3'	-16.40	144.00	
358	hsa-miR-500a*	[2139~2163]	25	miRNA: 3' guCUUAGG---AACGGGUCCACGUa 5'<br>|::||| | ||||| ||||| Target:5' ctGGGTCCCCGTGGCCCAAGTGCAg 3'	-20.30	140.00	
359	hsa-miR-500b	[1583~1600]	18	miRNA: 3' ugGGUCCAUCGUUCCUAa 5'<br>|||||| ||:|||| Target:5' ggCCAGGTCACAGGGATg 3'	-18.50	148.00	
360	hsa-miR-501-5p	[182~205]	24	miRNA: 3' agAGUGGGUCC-CU-GUUUCCUAa 5'<br>||:|||||| || |||| || Target:5' acTCGCCCAGGAGAGGAAAGCATg 3'	-24.29	140.00	
361	hsa-miR-503	[1353~1380]	28	miRNA: 3' gacGUCUUGACA-AGG----GCGACGAu 5'<br>|||::|||| | | :|||||| Target:5' aaaCAGGGCTGTGTACAAAGTGCTGCTg 3'	-22.50	146.00	
		[2262~2284]	23	miRNA: 3' gaCGUCUUGACAAGGGCGACGAu 5'<br>|::|:||| ||||||:| Target:5' gtGTGGGACTCCAGCCGCTGTTg 3'	-21.60	145.00	
		[1060~1084]	25	miRNA: 3' gacGUCUU-GACAAGG-GCGACGAu 5'<br>:|||| ||| |: :|||||| Target:5' ttcTAGAACCTGGGCTGTGCTGCTt 3'	-17.50	144.00	
		[1~16]	16	miRNA: 3' cuaucucacGUCUGGUCCCAGa 5'<br>|: :|||||||| Target:5' ------gccCGCGCCAGGGTCc 3'	-17.10	149.00	

<table>
<tr><td rowspan="3">362</td><td rowspan="3">hsa-miR-504</td><td>806~826</td><td>21</td><td>miRNA: 3' cuaucucACGUCUGGUCCCAGa 5'
| || | |||||||
Target:5' gcccgttTCCATA-CAGGGTCt 3'</td><td>-13.80</td><td>146.00</td><td rowspan="3"></td></tr>
<tr><td>2147~2168</td><td>22</td><td>miRNA: 3' cuaucucacgUCUGGUCCCAGa 5'
|| |||||||
Target:5' ccgtggcccaAGTGCAGGGTCc 3'</td><td>-13.60</td><td>144.00</td></tr>
<tr><td>581~603</td><td>23</td><td>miRNA: 3' cuAUCUCACG-UCUGGUCCCAGa 5'
|:|||| | ||| ||||||
Target:5' agTGGAGTACGAGAAAAGGGTCc 3'</td><td>-20.50</td><td>140.00</td></tr>
<tr><td>363</td><td>hsa-miR-505*</td><td>2016~2035</td><td>20</td><td>miRNA: 3' uguAGUUAUGAAGGACCGAGGg 5'
|| | || | ||||||||
Target:5' cccTCCA-ACCT-CTGGCTCCc 3'</td><td>-18.90</td><td>153.00</td><td></td></tr>
<tr><td>364</td><td>hsa-miR-505</td><td>2266~2287</td><td>22</td><td>miRNA: 3' ucCUUUGGUCGUUCACAACUGc 5'
|| ||||| : |||||:|
Target:5' ggGACTCCAGCCGCTGTTGGCt 3'</td><td>-18.20</td><td>148.00</td><td></td></tr>
<tr><td>365</td><td>hsa-miR-506</td><td>2355~2379</td><td>25</td><td>miRNA: 3' agaUGAGUCUUC-C---CACGGAAu 5'
|||| | :| | |||||||
Target:5' gcgACTCTGCGGTGCCAGTGCCTTg 3'</td><td>-14.30</td><td>150.00</td><td></td></tr>
<tr><td>366</td><td>hsa-miR-509-5p</td><td>2387~2409</td><td>23</td><td>miRNA: 3' acuaaCGGUGAC---AGACGUCAu 5'
||:|| | |||||||
Target:5' ttccgGCTAC-GCACCCTGCAGTc 3'</td><td>-14.60</td><td>143.00</td><td></td></tr>
<tr><td>367</td><td>hsa-miR-509-3p</td><td>2613~2639</td><td>27</td><td>miRNA: 3' gaugggUGUCUGCA-----UGGUUAGu 5'
||:||:|| :||||||
Target:5' gaagtaACGGATGTTTCTCGCCAATCg 3'</td><td>-17.40</td><td>140.00</td><td></td></tr>
<tr><td>368</td><td>hsa-miR-509-5p</td><td>2387~2409</td><td>23</td><td>miRNA: 3' acuaaCGGUGAC---AGACGUCAu 5'
||:|| | |||||||
Target:5' ttccgGCTAC-GCACCCTGCAGTc 3'</td><td>-14.60</td><td>143.00</td><td></td></tr>
<tr><td>369</td><td>hsa-miR-509-3p</td><td>2613~2639</td><td>27</td><td>miRNA: 3' gaugggUGUCUGCA-----UGGUUAGu 5'
||:||:|| :||||||
Target:5' gaagtaACGGATGTTTCTCGCCAATCg 3'</td><td>-17.40</td><td>140.00</td><td></td></tr>
<tr><td>370</td><td>hsa-miR-509-3-5p</td><td>2385~2409</td><td>25</td><td>miRNA: 3' guacuaaCGGUGC---AGACGUCAu 5'
||:||| |||||||
Target:5' ttttccgGCTACGCACCCTGCAGTc 3'</td><td>-16.90</td><td>151.00</td><td></td></tr>
<tr><td>371</td><td>hsa-miR</td><td>2613~2639</td><td>27</td><td>miRNA: 3' gaugggUGUCUGCA-----UGGUUAGu 5'</td><td>-17.40</td><td>140.00</td><td></td></tr>
</table>

<table>
<tr><td>371</td><td>miR-509-3p</td><td>2613~2639</td><td>27</td><td>[illegible]
||:||:|| :||||||
Target:5' gaagtaACGGATGTTTCTCGCCAATCg 3'</td><td>-17.40</td><td>140.00</td><td></td></tr>
<tr><td>372</td><td>hsa-miR-510</td><td>1792~1809</td><td>18</td><td>miRNA: 3' cacuaaCGGUGAGAGGACUCAu 5'
|||| |||||||
Target:5' cctgagGCCA----CCTGAGTg 3'</td><td>-15.90</td><td>146.00</td><td></td></tr>
<tr><td>373</td><td>hsa-miR-515-5p</td><td>1684~1707</td><td>24</td><td>miRNA: 3' gucuUUCACGAAAGAAAACCUCUu 5'
|:|||||||:| | |||||
Target:5' cgccAGGTGCTTTTTCTGGGAGAg 3'</td><td>-19.80</td><td>152.00</td><td></td></tr>
<tr><td>374</td><td>hsa-miR-515-3p</td><td>1946~1969</td><td>24</td><td>miRNA: 3' uugcGAGGUU--UUCUUCCGUGag 5'
|||| | |:||||||||
Target:5' tcacCTCCCAGCAGGAAGGCACag 3'</td><td>-18.80</td><td>148.00</td><td></td></tr>
<tr><td>375</td><td>hsa-miR-515-5p</td><td>1684~1707</td><td>24</td><td>miRNA: 3' gucuUUCACGAAAGAAAACCUCUu 5'
|:|||||||:| | |||||
Target:5' cgccAGGTGCTTTTTCTGGGAGAg 3'</td><td>-19.80</td><td>152.00</td><td></td></tr>
<tr><td>376</td><td>hsa-miR-515-3p</td><td>1946~1969</td><td>24</td><td>miRNA: 3' uugcGAGGUU--UUCUUCCGUGag 5'
|||| | |:||||||||
Target:5' tcacCTCCCAGCAGGAAGGCACag 3'</td><td>-18.80</td><td>148.00</td><td></td></tr>
<tr><td rowspan="4">377</td><td rowspan="4">hsa-miR-516a-3p</td><td>310~327</td><td>18</td><td>miRNA: 3' ugGGAGACUUUCCUUCGu 5'
|| : |:|||||:||
Target:5' ctCCGTGGGAAGGAGGCt 3'</td><td>-17.10</td><td>140.00</td><td rowspan="4"></td></tr>
<tr><td>1216~1233</td><td>18</td><td>miRNA: 3' ugggagACUUUCCUUCGu 5'
|||:||||:||
Target:5' tgaaagTGAGAGGAGGCg 3'</td><td>-16.70</td><td>140.00</td></tr>
<tr><td>310~327</td><td>18</td><td>miRNA: 3' ugGGAGACUUUCCUUCGu 5'
|| : |:|||||:||
Target:5' ctCCGTGGGAAGGAGGCt 3'</td><td>-17.10</td><td>140.00</td></tr>
<tr><td>1216~1233</td><td>18</td><td>miRNA: 3' ugggagACUUUCCUUCGu 5'
|||:||||:||
Target:5' tgaaagTGAGAGGAGGCg 3'</td><td>-16.70</td><td>140.00</td></tr>
<tr><td rowspan="2"></td><td rowspan="2"></td><td>310~327</td><td>18</td><td>miRNA: 3' ugGGAGACUUUCCUUCGu 5'
|| : |:|||||:||
Target:5' ctCCGTGGGAAGGAGGCt 3'</td><td>-17.10</td><td>140.00</td><td rowspan="2"></td></tr>
<tr><td>1216~1233</td><td>18</td><td>miRNA: 3' ugggagACUUUCCUUCGu 5'
|||:||||:||
[illegible]</td><td>-16.70</td><td>140.00</td></tr>
</table>

378	hsa-miR-516b*			Target:5' tgaaagTGAGAGGAGGCg 3'			
		310~327	18	miRNA: 3' ugGGAGACUUUCCUUCGu 5'<br>|| : |:|||||:|| Target:5' ctCCGTGGGAAGGAGGCt 3'	-17.10	140.00	
		1216~1233	18	miRNA: 3' ugggagACUUUCCUUCGu 5'<br>|||:||||:|| Target:5' tgaaagTGAGAGGAGGCg 3'	-16.70	140.00	
379	hsa-miR-517*	1151~1172	22	miRNA: 3' ucuGUCACGAAGGUAGAUCUCc 5'<br>| |||:| |::|:||||| Target:5' tgtCTGTGTTGCTGTTTAGAGt 3'	-14.30	151.00	
		427~453	27	miRNA: 3' ucUGUCAC---GA-AGGUAG-AUCUCc 5'<br>|||||| || |||||| ||||| Target:5' ggACAGTGTGCCTGTCCATCTTAGAGg 3'	-29.30	148.00	
		1151~1172	22	miRNA: 3' ucuGUCACGAAGGUAGAUCUCc 5'<br>| |||:| |::|:||||| Target:5' tgtCTGTGTTGCTGTTTAGAGt 3'	-14.30	151.00	
		427~453	27	miRNA: 3' ucUGUCAC---GA-AGGUAG-AUCUCc 5'<br>|||||| || |||||| ||||| Target:5' ggACAGTGTGCCTGTCCATCTTAGAGg 3'	-29.30	148.00	
380	hsa-miR-517b	1296~1317	22	miRNA: 3' uuGUGAGAU-UUCCCUACGUGCu 5'<br>|| |||: ||||||||| Target:5' tcCA-TCTGCTTCGGATGCACGc 3'	-17.10	151.00	
381	hsa-miR-517*	1151~1172	22	miRNA: 3' ucuGUCACGAAGGUAGAUCUCc 5'<br>| |||:| |::|:||||| Target:5' tgtCTGTGTTGCTGTTTAGAGt 3'	-14.30	151.00	
		427~453	27	miRNA: 3' ucUGUCAC---GA-AGGUAG-AUCUCc 5'<br>|||||| || |||||| ||||| Target:5' ggACAGTGTGCCTGTCCATCTTAGAGg 3'	-29.30	148.00	
382	hsa-miR-518a-3p	1675~1697	23	miRNA: 3' agGUCGUUU-CCCUUCGCGAAAg 5'<br>::||||| | |:|:||||| Target:5' cgTGGCAAACGCCAGGTGCTTTt 3'	-13.20	148.00	
		1675~1697	23	miRNA: 3' agGUCGUUU-CCCUUCGCGAAAg 5'<br>::||||| | |:|:||||| Target:5' cgTGGCAAACGCCAGGTGCTTTt 3'	-13.20	148.00	

<table>
<tr><th></th><th></th><th></th><th></th><th></th><th></th><th></th><th></th></tr>
<tr><td>383</td><td>hsa-miR-518c*</td><td>1711~1731</td><td>21</td><td>miRNA: 3' gucuuuCACGAAGGGAGGUCUCu 5'
                ||| ||||||||:|
Target:5' acagccGTG--GCCCTCCAGGGc 3'</td><td>-24.30</td><td>141.00</td><td></td></tr>
<tr><td>384</td><td>hsa-miR-518d-5p</td><td>1036~1057</td><td>22</td><td>miRNA: 3' gucuuucaCGAAGGGAGAUCUc 5'
                  ||| |:|||||||
Target:5' ctcctgccGCTCCTCTCTAGAa 3'</td><td>-14.50</td><td>158.00</td><td></td></tr>
<tr><td>385</td><td>hsa-miR-518d-3p</td><td>1676~1697</td><td>22</td><td>miRNA: 3' cgagGUUU-CCCUUCGCGAAAc 5'
              |||| | |:|:|||||
Target:5' gtggCAAACGCCAGGTGCTTTt 3'</td><td>-11.10</td><td>141.00</td><td></td></tr>
<tr><td>386</td><td>hsa-miR-518e*</td><td>1036~1057</td><td>22</td><td>miRNA: 3' gucuuucGCGAAGGGAGAUCUc 5'
                 |||| |:|||||||
Target:5' ctcctgcCGCTCCTCTCTAGAa 3'</td><td>-18.10</td><td>163.00</td><td></td></tr>
<tr><td>387</td><td>hsa-miR-518f*</td><td>1036~1057</td><td>22</td><td>miRNA: 3' cucuuucaCGAAGGGAGAUCUc 5'
                  ||| |:|||||||
Target:5' ctcctgccGCTCCTCTCTAGAa 3'</td><td>-14.50</td><td>158.00</td><td></td></tr>
<tr><td>388</td><td>hsa-miR-519a*</td><td>1036~1057</td><td>22</td><td>miRNA: 3' gucuuucGCGAAGGGAGAUCUc 5'
                 |||| |:|||||||
Target:5' ctcctgcCGCTCCTCTCTAGAa 3'</td><td>-18.10</td><td>163.00</td><td></td></tr>
<tr><td rowspan="2">389</td><td rowspan="2">hsa-miR-519a</td><td>982~1003</td><td>22</td><td>miRNA: 3' ugUGAGAUUUUCCUACGUGAAa 5'
            || |: |: | |||||||
Target:5' tcACCGTGCAGAGCTGCACTTg 3'</td><td>-10.00</td><td>152.00</td><td rowspan="2"></td></tr>
<tr><td>982~1003</td><td>22</td><td>miRNA: 3' ugUGAGAUUUUCCUACGUGAAa 5'
            || |: |: | |||||||
Target:5' tcACCGTGCAGAGCTGCACTTg 3'</td><td>-10.00</td><td>152.00</td></tr>
<tr><td>390</td><td>hsa-miR-519b-5p</td><td>1036~1057</td><td>22</td><td>miRNA: 3' gucuuucGCGAAGGGAGAUCUc 5'
                 |||| |:|||||||
Target:5' ctcctgcCGCTCCTCTCTAGAa 3'</td><td>-18.10</td><td>163.00</td><td></td></tr>
<tr><td>391</td><td>hsa-miR-519b-3p</td><td>978~1003</td><td>26</td><td>miRNA: 3' uuGGAG----AUUUUCCUACGUGAAa 5'
            :||| |: |: | |||||||
Target:5' ctTCTCACCGTGCAGAGCTGCACTTg 3'</td><td>-11.10</td><td>150.00</td><td></td></tr>
<tr><td>392</td><td>hsa-miR-519c-5p</td><td>1036~1057</td><td>22</td><td>miRNA: 3' gucuuucGCGAAGGGAGAUCUc 5'
                 |||| |:|||||||
Target:5' ctcctgcCGCTCCTCTCTAGAa 3'</td><td>-18.10</td><td>163.00</td><td></td></tr>
<tr><td></td><td>hsa</td><td></td><td></td><td></td><td></td><td></td><td></td></tr>
</table>

<table>
<tr><td>393</td><td>hsa-miR-519c-3p</td><td>978~1003</td><td>26</td><td>miRNA: 3' uaGGAG----AUUUUUCUACGUGAAa 5'
:||| |: |:|| |||||||
Target:5' ctTCTCACCGTGCAGAGCTGCACTTg 3'</td><td>-12.60</td><td>158.00</td><td></td></tr>
<tr><td>394</td><td>hsa-miR-519e*</td><td>1686~1707</td><td>22</td><td>miRNA: 3' cuUUCACGAGGGAAAACCUCUu 5'
|:|||||:::| | |||||
Target:5' ccAGGTGCTTTTTCTGGGAGAg 3'</td><td>-19.00</td><td>144.00</td><td></td></tr>
<tr><td>395</td><td>hsa-miR-520a-5p</td><td>947~967</td><td>21</td><td>miRNA: 3' ucuUUCAUGAAGGGAGACCUc 5'
:||| | || ||||||:
Target:5' ggcGAGTTCCTCGCTCTGGGa 3'</td><td>-15.50</td><td>146.00</td><td></td></tr>
<tr><td>396</td><td>hsa-miR-520c-5p</td><td>1036~1057</td><td>22</td><td>miRNA: 3' gucuuucaCGAAGGGAGAUCUc 5'
||| |:|||||||
Target:5' ctcctgccGCTCCTCTCTAGAa 3'</td><td>-14.50</td><td>158.00</td><td></td></tr>
<tr><td>397</td><td>hsa-miR-520d-5p</td><td>1335~1354</td><td>20</td><td>miRNA: 3' cuuucccgaagGGAAACAUc 5'
:|||||||
Target:5' cttgccctggaTCTTTGTAa 3'</td><td>-7.90</td><td>141.00</td><td></td></tr>
<tr><td>398</td><td>hsa-miR-520d-3p</td><td>2834~2853</td><td>20</td><td>miRNA: 3' ugGGUGGUUUCUCUUCGUGAAa 5'
||||| | | |||||:||
Target:5' atCCACC-ATTA-AAGCATTTg 3'</td><td>-16.70</td><td>142.00</td><td></td></tr>
<tr><td rowspan="4">399</td><td rowspan="4">hsa-miR-521</td><td>2746~2767</td><td>22</td><td>miRNA: 3' ugUGAGAUUUCCCUUCACGCAa 5'
| | ||:|| |||||:||
Target:5' ttAGTGGAAGGGAAAGTGTGTt 3'</td><td>-18.20</td><td>148.00</td><td rowspan="4"></td></tr>
<tr><td>267~290</td><td>24</td><td>miRNA: 3' uguGAG-AUUU-CCCUUCACGCaa 5'
||| |:|| ||:||||||
Target:5' aacCTCATGAACTGGGAGTGCGcc 3'</td><td>-18.60</td><td>143.00</td></tr>
<tr><td>2746~2767</td><td>22</td><td>miRNA: 3' ugUGAGAUUUCCCUUCACGCAa 5'
| | ||:|| |||||:||
Target:5' ttAGTGGAAGGGAAAGTGTGTt 3'</td><td>-18.20</td><td>148.00</td></tr>
<tr><td>267~290</td><td>24</td><td>miRNA: 3' uguGAG-AUUU-CCCUUCACGCaa 5'
||| |:|| ||:||||||
Target:5' aacCTCATGAACTGGGAGTGCGcc 3'</td><td>-18.60</td><td>143.00</td></tr>
<tr><td>400</td><td>hsa-miR-522*</td><td>1036~1057</td><td>22</td><td>miRNA: 3' gucuuucGCGAAGGGAGAUCUc 5'
|||| |:|||||||
Target:5' ctcctgcCGCTCCTCTCTAGAa 3'</td><td>-18.10</td><td>163.00</td><td></td></tr>
<tr><td>401</td><td>hsa-miR-</td><td>1036~1057</td><td>22</td><td>miRNA: 3' gucuuucGCGAAGGGAGAUCUc 5'
|||| |:|||||||</td><td>-18.10</td><td>163.00</td><td></td></tr>
</table>

<table>
<tr><td></td><td>523*</td><td></td><td></td><td>Target:5' ctcctgcCGCTCCTCTCTAGAa 3'</td><td></td><td></td><td></td></tr>
<tr><td rowspan="4">402</td><td rowspan="4">hsa-miR-524-5p</td><td>2655~2673</td><td>19</td><td>miRNA: 3' cuCUUUCACGAAGGGAAACAUc 5'
             |||| || |:|:||||||
Target:5' acGAAA-TG--TTCTTTTGTAg 3'</td><td>-11.90</td><td>147.00</td><td rowspan="4"></td></tr>
<tr><td>2506~2527</td><td>22</td><td>miRNA: 3' cucUUUCACGAAGGGAAACAUc 5'
              |:| ||: |: :||||||
Target:5' cccAGAATGTCTTATTTTGTAa 3'</td><td>-8.50</td><td>143.00</td></tr>
<tr><td>1327~1354</td><td>28</td><td>miRNA: 3' cucuuuCACGAAGG------GAAACAUc 5'
                 || :| || |||||||
Target:5' ggctctGTCTTGCCCTGGATCTTTGTAa 3'</td><td>-8.30</td><td>142.00</td></tr>
<tr><td>2725~2747</td><td>23</td><td>miRNA: 3' cuCUUU-CACGAAGGGAAACAuc 5'
             |||| | | | |||||||
Target:5' ctGAAACGCGGTCACCTTTGTtt 3'</td><td>-10.70</td><td>140.00</td></tr>
<tr><td>403</td><td>hsa-miR-524-3p</td><td>65~85</td><td>21</td><td>miRNA: 3' ugagGUUUCCCUUCGCGGaag 5'
               ||||||||||||||
Target:5' gagaCAAAGGGAAGCGCCgcc 3'</td><td>-27.20</td><td>145.00</td><td></td></tr>
<tr><td>404</td><td>hsa-miR-525-5p</td><td>947~967</td><td>21</td><td>miRNA: 3' ucuUUCACGUAGGGAGACCUc 5'
              :||| | || ||||||:
Target:5' ggcGAGTTCCTCGCTCTGGGa 3'</td><td>-15.50</td><td>146.00</td><td></td></tr>
<tr><td rowspan="2">405</td><td rowspan="2">hsa-miR-525-3p</td><td>2357~2379</td><td>23</td><td>miRNA: 3' gcGAGAUUUC-CCUUCGCGGAAg 5'
             ||||: :| | ||:|||||
Target:5' gaCTCTGCGGTGCCAGTGCCTTg 3'</td><td>-16.80</td><td>144.00</td><td rowspan="2"></td></tr>
<tr><td>64~85</td><td>22</td><td>miRNA: 3' gcgagaUUUCCCUUCGCGGaag 5'
                 |||||||||||||
Target:5' cgagacAAAGGGAAGCGCCgcc 3'</td><td>-24.80</td><td>140.00</td></tr>
<tr><td rowspan="2">406</td><td rowspan="2">hsa-miR-526a</td><td>1036~1057</td><td>22</td><td>miRNA: 3' gucuuucaCGAAGGGAGAUCUc 5'
                   ||| |:|||||||
Target:5' ctcctgccGCTCCTCTCTAGAa 3'</td><td>-14.50</td><td>158.00</td><td rowspan="2"></td></tr>
<tr><td>1036~1057</td><td>22</td><td>miRNA: 3' gucuuucaCGAAGGGAGAUCUc 5'
                   ||| |:|||||||
Target:5' ctcctgccGCTCCTCTCTAGAa 3'</td><td>-14.50</td><td>158.00</td></tr>
<tr><td>407</td><td>hsa-miR-539</td><td>2611~2632</td><td>22</td><td>miRNA: 3' ugugUGGUU-CCUAUUAAAGAGg 5'
               | :|| |||| :||||||
Target:5' ctgaAGTAACGGAT-GTTTCTCg 3'</td><td>-10.50</td><td>141.00</td><td></td></tr>
</table>

408	hsa-miR-541*	1755~1789	35	miRNA: 3' ucaCCCUGG--CUGUCG--------UCUUAGGAAa 5'<br>||| || |:|||| :||||:||| Target:5' agaGGGCCCTGGGCAGCCTGTGTCTGGAATTCTTc 3'	-19.50	146.00	
409	hsa-miR-542-5p	2125~2150	26	miRNA: 3' agagCACUGUACU--A-CUAGGGGCu 5'<br>|||:| || | |:|||||| Target:5' cccgGTGGCTGGAGCTGGGTCCCCGt 3'	-23.40	141.00	
410	hsa-miR-543	397~417	21	miRNA: 3' uucuUCACGUGGCGCUUACAaa 5'<br>| | |||| ||||||| Target:5' cattATTTCACC-CGAATGTgt 3'	-14.30	141.00	
411	hsa-miR-544b	1612~1633	22	miRNA: 3' aaucuUUACGUGUUGGAGUCCa 5'<br>|: |::| |||||||| Target:5' tgcatAGGGTGCCACCTCAGGt 3'	-20.50	157.00	
		989~1012	24	miRNA: 3' aaUCUUUACGUG--UUGGAGUCca 5'<br>|||: ||||| ::|||||| Target:5' gcAGAGCTGCACTTGGCCTCAGct 3'	-18.70	150.00	
		1637~1656	20	miRNA: 3' aaucuuuACGUGUUGGAGUCCa 5'<br>||::: ||||||| Target:5' tgtccctTGTGT--CCTCAGGa 3'	-12.00	143.00	
412	hsa-miR-548a-5p	859~880	22	miRNA: 3' ccAUUUUGAGCGUUAAUGAAAa 5'<br>|||||||||| |||:||| Target:5' tgTAAAACTCGCTTTTATTTTa 3'	-18.90	168.00	
413	hsa-miR-548aa	653~678	26	miRNA: 3' acCACGUUUUCAUUAAC--ACCAAAaa 5'<br>|| |||||| || | |||||| Target:5' tcGT-CAAAAGGAAGGGATTGGTTTgg 3'	-9.30	148.00	
		2427~2448	22	miRNA: 3' acCACGUUUUCAUUAACACCAAAaa 5'<br>|| | :| || ||||||||| Target:5' acGT-CTGATGT--TTGTGGTTTgt 3'	-9.50	142.00	
		653~678	26	miRNA: 3' acCACGUUUUCAUUAAC--ACCAAAaa 5'<br>|| |||||| || | |||||| Target:5' tcGT-CAAAAGGAAGGGATTGGTTTgg 3'	-9.30	148.00	
		2427~2448	22	miRNA: 3' acCACGUUUUCAUUAACACCAAAaa 5'<br>|| | :| || ||||||||| Target:5' acGT-CTGATGT--TTGTGGTTTgt 3'	-9.50	142.00	

<table>
<tr><td>414</td><td>hsa-miR-548b-5p</td><td>858~880</td><td>23</td><td>miRNA: 3' ccgGUUUUG-GUGUUAAUGAAAa 5'
:||||| |:| |||:|||
Target:5' atgTAAAACTCGCTTTTATTTTa 3'</td><td>-9.90</td><td>147.00</td><td></td></tr>
<tr><td>415</td><td>hsa-miR-548c-5p</td><td>859~880</td><td>22</td><td>miRNA: 3' ccGUUUUUGGCGUUAAUGAAAa 5'
:|||| :||| |||:|||
Target:5' tgTAAAACTCGCTTTTATTTTa 3'</td><td>-12.30</td><td>152.00</td><td></td></tr>
<tr><td>416</td><td>hsa-miR-548d-5p</td><td>859~880</td><td>22</td><td>miRNA: 3' ccGUUUUUGGUGUUAAUGAAAa 5'
:|||| :|:| |||:|||
Target:5' tgTAAAACTCGCTTTTATTTTa 3'</td><td>-10.40</td><td>148.00</td><td></td></tr>
<tr><td>417</td><td>hsa-miR-548d-3p</td><td>1170~1200</td><td>31</td><td>miRNA: 3' cguUUUCUUUGACA---------CCAAAAac 5'
||| ||||||| ||||||
Target:5' agtAAATAAACTGTTTATATAAAGGTTTTgg 3'</td><td>-10.50</td><td>143.00</td><td></td></tr>
<tr><td>418</td><td>hsa-miR-548d-5p</td><td>859~880</td><td>22</td><td>miRNA: 3' ccGUUUUUGGUGUUAAUGAAAa 5'
:|||| :|:| |||:|||
Target:5' tgTAAAACTCGCTTTTATTTTa 3'</td><td>-10.40</td><td>148.00</td><td></td></tr>
<tr><td>419</td><td>hsa-miR-548d-3p</td><td>1170~1200</td><td>31</td><td>miRNA: 3' cguUUUCUUUGACA---------CCAAAAac 5'
||| ||||||| ||||||
Target:5' agtAAATAAACTGTTTATATAAAGGTTTTgg 3'</td><td>-10.50</td><td>143.00</td><td></td></tr>
<tr><td rowspan="4">420</td><td rowspan="4">hsa-miR-548h</td><td>859~880</td><td>22</td><td>miRNA: 3' cuGUUUUUGGCGCUAAUGAAAa 5'
:|||| :||| |||:|||
Target:5' tgTAAAACTCGCTTTTATTTTa 3'</td><td>-9.60</td><td>152.00</td><td rowspan="4"></td></tr>
<tr><td>859~880</td><td>22</td><td>miRNA: 3' cuGUUUUUGGCGCUAAUGAAAa 5'
:|||| :||| |||:|||
Target:5' tgTAAAACTCGCTTTTATTTTa 3'</td><td>-9.60</td><td>152.00</td></tr>
<tr><td>859~880</td><td>22</td><td>miRNA: 3' cuGUUUUUGGCGCUAAUGAAAa 5'
:|||| :||| |||:|||
Target:5' tgTAAAACTCGCTTTTATTTTa 3'</td><td>-9.60</td><td>152.00</td></tr>
<tr><td>859~880</td><td>22</td><td>miRNA: 3' cuGUUUUUGGCGCUAAUGAAAa 5'
:|||| :||| |||:|||
Target:5' tgTAAAACTCGCTTTTATTTTa 3'</td><td>-9.60</td><td>152.00</td></tr>
<tr><td rowspan="2"></td><td rowspan="2"></td><td>859~880</td><td>22</td><td>miRNA: 3' ccGUUUUAGGCGUUAAUGAAAa 5'
:|||| :||| |||:|||
Target:5' tgTAAAACTCGCTTTTATTTTa 3'</td><td>-12.60</td><td>152.00</td><td rowspan="2"></td></tr>
<tr><td></td><td></td><td>miRNA: 3' ccGUUUUAGGCGUUAAUGAAAa 5'</td><td></td><td></td></tr>
</table>

<table>
<tr><td rowspan="3">421</td><td rowspan="3">hsa-miR-548i</td><td>859~880</td><td>22</td><td>:|||| :||| |||:|||
Target:5' tgTAAAACTCGCTTTTATTTTa 3'</td><td>-12.60</td><td>152.00</td><td rowspan="3"></td></tr>
<tr><td>859~880</td><td>22</td><td>miRNA: 3' ccGUUUUAGGCGUUAAUGAAAa 5'
:|||| :||| |||:|||
Target:5' tgTAAAACTCGCTTTTATTTTa 3'</td><td>-12.60</td><td>152.00</td></tr>
<tr><td>859~880</td><td>22</td><td>miRNA: 3' ccGUUUUAGGCGUUAAUGAAAa 5'
:|||| :||| |||:|||
Target:5' tgTAAAACTCGCTTTTATTTTa 3'</td><td>-12.60</td><td>152.00</td></tr>
<tr><td>422</td><td>hsa-miR-548j</td><td>859~880</td><td>22</td><td>miRNA: 3' ugGUUUCUGGCGUUAAUGAAAa 5'
:||| :||| |||:|||
Target:5' tgTAAAACTCGCTTTTATTTTa 3'</td><td>-9.40</td><td>144.00</td><td></td></tr>
<tr><td>423</td><td>hsa-miR-548k</td><td>1678~1697</td><td>20</td><td>miRNA: 3' ucGUUUUAGGC-GUUCAUGAAAa 5'
|||| || ||:||:||||
Target:5' ggCAAA---CGCCAGGTGCTTTt 3'</td><td>-15.40</td><td>145.00</td><td></td></tr>
<tr><td>424</td><td>hsa-miR-548l</td><td>328~349</td><td>22</td><td>miRNA: 3' cuguuUUGGGCGUUUAUGAAAa 5'
|| |: |::||:||||
Target:5' tgtttAAACTACGGATGCTTTt 3'</td><td>-11.40</td><td>141.00</td><td></td></tr>
<tr><td>425</td><td>hsa-miR-548m</td><td>330~350</td><td>21</td><td>miRNA: 3' guuUUUGGUGUUUAUGGAAAc 5'
||||:||::||:|:|||
Target:5' tttAAACTACGGATGCTTTTc 3'</td><td>-15.30</td><td>146.00</td><td></td></tr>
<tr><td>426</td><td>hsa-miR-548n</td><td>329~350</td><td>22</td><td>miRNA: 3' uguuUUAGGUGUUAAUGAAAAc 5'
|| |:||:: |:|||||
Target:5' gtttAAACTACGGATGCTTTTc 3'</td><td>-10.10</td><td>146.00</td><td></td></tr>
<tr><td>427</td><td>hsa-miR-548s</td><td>988~1008</td><td>21</td><td>miRNA: 3' uuuUAUUGACGUCAAAACCGGua 5'
| |:||||| ||||||
Target:5' tgcAGAGCTGCA--CTTGGCCtc 3'</td><td>-15.40</td><td>140.00</td><td></td></tr>
<tr><td>428</td><td>hsa-miR-548t</td><td>330~350</td><td>21</td><td>miRNA: 3' guuUUUGGUGCUAGUGAAAAc 5'
||||:|||: ::|||||
Target:5' tttAAACTACGGATGCTTTTc 3'</td><td>-13.70</td><td>142.00</td><td></td></tr>
<tr><td>429</td><td>hsa-miR-548v</td><td>2482~2501</td><td>20</td><td>miRNA: 3' acCACGUUUUCAUUGACAUCGa 5'
|| ::|| || :||||||
Target:5' tgGTTTGAA--TATTTGTAGCc 3'</td><td>-10.90</td><td>140.00</td><td></td></tr>
<tr><td>430</td><td>hsa-miR-548w</td><td>858~880</td><td>23</td><td>miRNA: 3' ucCGUUUUUGGCGUCAAUGAAAa 5'
|:|||| :||| |||:|||
Target:5' atGTAAAACTCGCTTTTATTTTa 3'</td><td>-11.50</td><td>157.00</td><td></td></tr>
</table>

<table>
<tr><td>431</td><td>hsa-miR-551b*</td><td>211~230</td><td>20</td><td>miRNA: 3' ccagaGUGGGUGCGAACUAAAg 5'
     |||||| :|||:|||
Target:5' aagacCACCCA--TTTGGTTTc 3'</td><td>-17.60</td><td>145.00</td><td></td></tr>
<tr><td rowspan="2">432</td><td rowspan="2">hsa-miR-552</td><td>723~743</td><td>21</td><td>miRNA: 3' aacagAUUGGUCAGUGGACaa 5'
     |:||:|||||||||
Target:5' tacaaTGACTAGTCACCTGgg 3'</td><td>-21.20</td><td>152.00</td><td rowspan="2"></td></tr>
<tr><td>91~115</td><td>25</td><td>miRNA: 3' aacagauuGGUCA----GUGGACAa 5'
        :|:|| |||||||
Target:5' cgccccgcTCGGTCCTCCACCTGTc 3'</td><td>-12.30</td><td>143.00</td></tr>
<tr><td>433</td><td>hsa-miR-555</td><td>1321~1342</td><td>22</td><td>miRNA: 3' uaguCUCCAAGUC-GAAUGGGa 5'
    |||| || | |||:|||
Target:5' ctgtGAGGCTCTGTCTTGCCCt 3'</td><td>-18.20</td><td>145.00</td><td></td></tr>
<tr><td>434</td><td>hsa-miR-557</td><td>971~994</td><td>24</td><td>miRNA: 3' ucUGUUCCGGGUG-GGCACGUUUg 5'
  :|| | ::| | |||||||:|
Target:5' agGCATGCTTCTCACCGTGCAGAg 3'</td><td>-20.20</td><td>145.00</td><td></td></tr>
<tr><td>435</td><td>hsa-miR-566</td><td>741~761</td><td>21</td><td>miRNA: 3' caaCCCUAG--UGUCCGCGGg 5'
   |||:|: :|:||||||
Target:5' gggGGGGTTGGGCGGGCGCCa 3'</td><td>-24.50</td><td>142.00</td><td></td></tr>
<tr><td>436</td><td>hsa-miR-572</td><td>28~48</td><td>21</td><td>miRNA: 3' acCCGGUG-GCGGCUCGCCUg 5'
  |||::| ||| ||||||:
Target:5' ctGGCTGCGCGCGGAGCGGGc 3'</td><td>-29.00</td><td>150.00</td><td></td></tr>
<tr><td>437</td><td>hsa-miR-573</td><td>1108~1131</td><td>24</td><td>miRNA: 3' gacuAGUCAAUGUGUAGUGAAGuc 5'
    ||:||| ::|: ||||||
Target:5' catcTCGGTTCTGCGCCACTTCct 3'</td><td>-15.30</td><td>148.00</td><td></td></tr>
<tr><td>438</td><td>hsa-miR-575</td><td>997~1017</td><td>21</td><td>miRNA: 3' cgaGGACAG--GUUGACCGAg 5'
   |:|| | ||:||||||
Target:5' gcaCTTGGCCTCAGCTGGCTg 3'</td><td>-18.20</td><td>142.00</td><td></td></tr>
<tr><td rowspan="2">439</td><td rowspan="2">hsa-miR-578</td><td>441~464</td><td>24</td><td>miRNA: 3' uguUAGGAU---CUCGUGUUCUUc 5'
   |||:|| ||| |||||:|
Target:5' tccATCTTAGAGGAGGACAAGGAc 3'</td><td>-14.40</td><td>150.00</td><td rowspan="2"></td></tr>
<tr><td>489~513</td><td>25</td><td>miRNA: 3' ugUUAGGAU----CUCGUGUUCUUc 5'
  :|||||| || :|||:|||
Target:5' caGATCCTATTAGGAATACAGGAAc 3'</td><td>-13.20</td><td>149.00</td></tr>
</table>

<table>
<tr><td></td><td></td><td>576~596</td><td>21</td><td>miRNA: 3' uguuaggAUCUCGUGUUCUUc 5'
|:|||:||:||||
Target:5' aacagagTGGAGTACGAGAAa 3'</td><td>-16.80</td><td>146.00</td><td></td></tr>
<tr><td rowspan="2">440</td><td rowspan="2">hsa-miR-587</td><td>2564~2583</td><td>20</td><td>miRNA: 3' caCUGAGUAGUGGAUACCUUu 5'
|::| || || |||||||
Target:5' atGGTTAAT-ACATATGGAAa 3'</td><td>-12.60</td><td>158.00</td><td rowspan="2">?</td></tr>
<tr><td>1002~1025</td><td>24</td><td>miRNA: 3' caCUGAGU--AGUGG-AUACCUUu 5'
| |||| | :|: |||||||
Target:5' tgGCCTCAGCTGGCTGTATGGAAa 3'</td><td>-12.50</td><td>153.00</td></tr>
<tr><td>441</td><td>hsa-miR-588</td><td>1566~1588</td><td>23</td><td>miRNA: 3' caagauuGGGUAA--CACCGGUu 5'
||: || |||||||
Target:5' gggttccCCTCTTCAGTGGCCAg 3'</td><td>-17.80</td><td>148.00</td><td>?</td></tr>
<tr><td>442</td><td>hsa-miR-589</td><td>1098~1121</td><td>24</td><td>miRNA: 3' gaGUCUCGUC-UGCA-CCAAGAgu 5'
|||: ||| |: | ||||||
Target:5' ccCAGGTCAGCATCTCGGTTCTgc 3'</td><td>-13.90</td><td>140.00</td><td>?</td></tr>
<tr><td>443</td><td>hsa-miR-593</td><td>539~557</td><td>19</td><td>miRNA: 3' ucuUUGGGGUCGUCUCUGu 5'
|:|:| |||||||:|
Target:5' cccAGCTCAAGCAGAGGCc 3'</td><td>-17.40</td><td>148.00</td><td>?</td></tr>
<tr><td>444</td><td>hsa-miR-596</td><td>1303~1323</td><td>21</td><td>miRNA: 3' gggcuCCUCGGCCCGUCCGAa 5'
||| | |||||||
Target:5' gcttcGGATGCACGCAGGCTg 3'</td><td>-15.80</td><td>148.00</td><td>?</td></tr>
<tr><td>445</td><td>hsa-miR-602</td><td>2130~2153</td><td>24</td><td>miRNA: 3' ccCGGCGUCGA--CAGCGGGCACag 5'
||:| |||| ||| ||||||
Target:5' tgGCTGGAGCTGGGTC-CCCGTGgc 3'</td><td>-28.80</td><td>150.00</td><td>?</td></tr>
<tr><td>446</td><td>hsa-miR-603</td><td>2750~2767</td><td>18</td><td>miRNA: 3' cgUUUUCAUUAACGUCACACAc 5'
:||:| || |||||||
Target:5' tgGAAGGGAA----AGTGTGTt 3'</td><td>-9.00</td><td>150.00</td><td>?</td></tr>
<tr><td rowspan="3"></td><td rowspan="3">hsa-</td><td>2326~2351</td><td>26</td><td>miRNA: 3' ugccUCGACA-GGGUUGUGGUGGGGa 5'
:|| || ||| |||||||||
Target:5' agccGGCAGTGCCCCCCACCACCCCt 3'</td><td>-31.80</td><td>169.00</td><td rowspan="3"></td></tr>
<tr><td>1652~1677</td><td>26</td><td>miRNA: 3' ugCCU-CGACAGGGUUGUGGUGGGga 5'
||| || | |::: :|||||||
Target:5' caGGAGGCAGCCTTGCTACCACCCgt 3'</td><td>-22.70</td><td>147.00</td></tr>
<tr><td></td><td></td><td></td><td></td><td></td></tr>
</table>

<table>
<tr><td rowspan="3">447</td><td rowspan="3">hsa-
miR-
608</td><td>73~97</td><td>25</td><td>miRNA: 3' ugCCUCGACAG-GGUUGUGGUGGGGa 5'
| ||| | | ||: |:||:||||
Target:5' ggGAAGC-GCCGCCGCCGCCGCCCCg 3'</td><td>-27.30</td><td>146.00</td><td rowspan="3"></td></tr>
<tr><td>1603~1630</td><td>28</td><td>miRNA: 3' ugcCUCGACA-GGGU--UGUGGUGGGGa 5'
| ||||| | :| :::|||||:|
Target:5' gctGCGCTGTGCATAGGGTGCCACCTCa 3'</td><td>-21.60</td><td>144.00</td></tr>
<tr><td>2096~2126</td><td>31</td><td>miRNA: 3' ugCCUCGACAGGGUUG------UGGUGGGGa 5'
||||||| ||| :| : ||||||
Target:5' ctGGAGCTGGCCCTGCTGCCCTGGCACCCCc 3'</td><td>-29.70</td><td>141.00</td></tr>
<tr><td rowspan="3">448</td><td rowspan="3">hsa-
miR-
611</td><td>1~20</td><td>20</td><td>miRNA: 3' cagucugGGGCUC---CCCAGGAGCg 5'
|||| | |||||||||
Target:5' ------gCCCGCGCCAGGGTCCTCGg 3'</td><td>-26.10</td><td>160.00</td><td rowspan="3"></td></tr>
<tr><td>2669~2688</td><td>20</td><td>miRNA: 3' caGUC-UGGGGCUCCCCAGGAGCg 5'
:|| ||| | ||||||||
Target:5' tgTAGTACCAC----GGTCCTCGg 3'</td><td>-18.70</td><td>151.00</td></tr>
<tr><td>87~109</td><td>23</td><td>miRNA: 3' cagucUGGGGCUCCCCAGGAGcg 5'
:||||| |||||||
Target:5' ccgccGCCCCGCTCGGTCCTCca 3'</td><td>-24.60</td><td>142.00</td></tr>
<tr><td rowspan="2">449</td><td rowspan="2">hsa-
miR-
612</td><td>168~192</td><td>25</td><td>miRNA: 3' uuccucGAGUCUUCGGGACGGGUCg 5'
||||| || |: ||||||
Target:5' atcgccCTCAGCAGACTCGCCCAGg 3'</td><td>-22.10</td><td>143.00</td><td rowspan="2"></td></tr>
<tr><td>1225~1250</td><td>26</td><td>miRNA: 3' uuCCUCGAGUCUUCGGG--ACGGGUCg 5'
|||| |:| ||| |||||:|
Target:5' gaGGAG-GCGGCCTCCCAGTGCCCGGc 3'</td><td>-28.30</td><td>140.00</td></tr>
<tr><td>450</td><td>hsa-
miR-
615-5p</td><td>2251~2274</td><td>24</td><td>miRNA: 3' cuAGGCUCGUGGC---CCCUGGGGg 5'
||| || ||| |||||:||
Target:5' ccTCC-AGGTCCGTGTGGGACTCCa 3'</td><td>-24.30</td><td>143.00</td><td></td></tr>
<tr><td>451</td><td>hsa-
miR-
616*</td><td>1068~1090</td><td>23</td><td>miRNA: 3' uucagUGAC-UUCCCAAAACUCa 5'
:||| : | |||||||
Target:5' cctggGCTGTGCTGCTTTTGAGc 3'</td><td>-7.90</td><td>145.00</td><td></td></tr>
<tr><td>452</td><td>hsa-
miR-
616</td><td>706~732</td><td>27</td><td>miRNA: 3' gacGAGUUUGG-GAG----GUUACUGa 5'
|| ||::: ||| |||||||
Target:5' aatCTAAAGTTGCTCCATACAATGACt 3'</td><td>-15.40</td><td>153.00</td><td></td></tr>
<tr><td>453</td><td>hsa-
miR-
617</td><td>43~63</td><td>21</td><td>miRNA: 3' cggugGAAGUUUACCCUUCAGa 5'
|| |::| ||||||||</td><td>-19.20</td><td>156.00</td><td></td></tr>
</table>

<table>
<tr><td></td><td>617</td><td></td><td></td><td>Target:5' gcgggCTCCGGA-GGGAAGTCc 3'</td><td></td><td></td><td></td></tr>
<tr><td rowspan="2">454</td><td rowspan="2">hsa-miR-619</td><td>2242~2260</td><td>19</td><td>miRNA: 3' ugACCCGUGUUUGUACAGGUCCAg 5'
  | ||||| | |||||||
Target:5' gcTCGGCAC---C--CTCCAGGTc 3'</td><td>-20.40</td><td>147.00</td><td rowspan="2"></td></tr>
<tr><td>1698~1731</td><td>34</td><td>miRNA: 3' ugACCC------GUGUUUGUAC----AGGUCCag 5'
  |||| ||||: |:|| ||||||
Target:5' tcTGGGAGAGCCCACAGCCGTGGCCCTCCAGGgc 3'</td><td>-24.70</td><td>142.00</td></tr>
<tr><td>455</td><td>hsa-miR-622</td><td>166~186</td><td>21</td><td>miRNA: 3' cgaGGUUGGAGUCGUCUGAca 5'
   :|: ||||||||||||
Target:5' ggaTCGCCCTCAGCAGACTcg 3'</td><td>-24.20</td><td>154.00</td><td></td></tr>
<tr><td>456</td><td>hsa-miR-623</td><td>130~151</td><td>22</td><td>miRNA: 3' uggGUUGUCGG-GGACGUUCCCUa 5'
   | :|:||| || |:|||||
Target:5' gggCTGCGGCCGCC--CGAGGGAc 3'</td><td>-19.80</td><td>140.00</td><td></td></tr>
<tr><td rowspan="3">457</td><td rowspan="3">hsa-miR-624</td><td>2361~2381</td><td>21</td><td>miRNA: 3' ucCAUUAUGGUUAUGGAACAc 5'
  | ::|:|||:|:||||||
Target:5' ctGCGGTGCCAGTGCCTTGTt 3'</td><td>-19.90</td><td>155.00</td><td rowspan="3"></td></tr>
<tr><td>2693~2713</td><td>21</td><td>miRNA: 3' ucCAUUAUGGUUAUGGAACAc 5'
  | |: || : |||||||
Target:5' acGAAGGACGTGAACCTTGTa 3'</td><td>-8.60</td><td>147.00</td></tr>
<tr><td>627~649</td><td>23</td><td>miRNA: 3' uccAUUA-UGGUU-AUGGAACAc 5'
   | || | ||: |||||||
Target:5' cccTCATAAGCAGCGACCTTGTg 3'</td><td>-14.00</td><td>146.00</td></tr>
<tr><td rowspan="2">458</td><td rowspan="2">hsa-miR-628-5p</td><td>875~896</td><td>22</td><td>miRNA: 3' ggAGAUCAUUUAUACAGUCGUa 5'
  |:|| || |||||||:|
Target:5' atTTTAATATTGATGTCAGTAt 3'</td><td>-13.40</td><td>148.00</td><td rowspan="2"></td></tr>
<tr><td>1089~1110</td><td>22</td><td>miRNA: 3' ggagaucauuuaUACAGUCGUa 5'
            | |||||||
Target:5' gcctcagaccccAGGTCAGCAt 3'</td><td>-17.54</td><td>142.00</td></tr>
<tr><td>459</td><td>hsa-miR-629*</td><td>1687~1708</td><td>22</td><td>miRNA: 3' cgaCCCGAAUGCAACCCUCUUg 5'
   | |||| : |||||||:
Target:5' cagGTGCTTTTTCTGGGAGAGc 3'</td><td>-20.70</td><td>143.00</td><td></td></tr>
<tr><td></td><td></td><td>1489~1512</td><td>24</td><td>miRNA: 3' cgACUCC---AGACCCGGUCCAGa 5'
  ||:|| | |||||||||
Target:5' ctTGGGGGCCACAAGGCCAGGTCc 3'</td><td>-23.50</td><td>155.00</td><td></td></tr>
</table>

<table>
<tr><td rowspan="3">460</td><td rowspan="3">hsa-miR-631</td><td>1571~1592</td><td>22</td><td>miRNA: 3' cgacuccAGAC-CCGGUCCAGa 5'
                 || | |||||||||
Target:5' cccctctTCAGTGGCCAGGTCa 3'</td><td>-16.90</td><td>154.00</td><td rowspan="3"></td></tr>
<tr><td>1084~1106</td><td>23</td><td>miRNA: 3' cgACUC-CAGAC-CCGGUCCAGa 5'
            |||| || | |||||||
Target:5' ttTGAGCCTCAGACCCCAGGTCa 3'</td><td>-13.99</td><td>147.00</td></tr>
<tr><td>2237~2261</td><td>25</td><td>miRNA: 3' cgaCUCCAGACC-----CGGUCCAGa 5'
             ||| || || |||||||
Target:5' gggGAGCTC-GGCACCCTCCAGGTCc 3'</td><td>-17.10</td><td>145.00</td></tr>
<tr><td>461</td><td>hsa-miR-632</td><td>958~976</td><td>19</td><td>miRNA: 3' agGGUGUCCUUCGUCUGUg 5'
            |: ::||| ||||:||
Target:5' cgCTCTGGGATGCAGGCAt 3'</td><td>-16.90</td><td>141.00</td><td></td></tr>
<tr><td>462</td><td>hsa-miR-634</td><td>1927~1947</td><td>21</td><td>miRNA: 3' caggUUUCAACCCCACGACCAa 5'
              :||||| |||||| |||
Target:5' ctggGAAGTT-GGGTGCCGGTc 3'</td><td>-18.20</td><td>141.00</td><td></td></tr>
<tr><td>463</td><td>hsa-miR-635</td><td>2136~2159</td><td>24</td><td>miRNA: 3' ccuguaaCAAAGUCA-CGGGUUCa 5'
                 || | || |||||||
Target:5' gagctggGTCCCCGTGGCCCAAGt 3'</td><td>-15.60</td><td>148.00</td><td></td></tr>
<tr><td rowspan="5">464</td><td rowspan="5">hsa-miR-637</td><td>2235~2258</td><td>24</td><td>miRNA: 3' ugCGUCUCGGGCUUUCGGGGGUCa 5'
            | :||||:||: | ||:||||
Target:5' aaGGGGAGCTCGGCACCCTCCAGg 3'</td><td>-26.90</td><td>158.00</td><td rowspan="5"></td></tr>
<tr><td>1994~2016</td><td>23</td><td>miRNA: 3' ugcGUCUCGGGCUUUCGGGGGUCa 5'
             || |||||| : ||:|||||
Target:5' ggaCACAGCCCG-GTGCTCCCAGc 3'</td><td>-29.70</td><td>156.00</td></tr>
<tr><td>920~946</td><td>27</td><td>miRNA: 3' ugcgucucGGGCU--UUC-GGGGGUCa 5'
                  |::|: ||| |||||||
Target:5' cttttataCTTGGGTAAGTCCCCCAGg 3'</td><td>-19.50</td><td>150.00</td></tr>
<tr><td>1700~1729</td><td>30</td><td>miRNA: 3' ugCGUCUCGGGCUUUC-------GGGGGUCa 5'
            | ||||||| | || ||:||||
Target:5' tgGGAGAGCCC-ACAGCCGTGGCCCTCCAGg 3'</td><td>-26.80</td><td>145.00</td></tr>
<tr><td>2329~2356</td><td>28</td><td>miRNA: 3' ugCGUCUC--GGGCU--UUCGGGGGUCa 5'
            |||| | ||| | | ||:||||
Target:5' cgGCAGTGCCCCCCACCACCCCTCCAGc 3'</td><td>-22.84</td><td>142.00</td></tr>
</table>

<table>
<tr><td rowspan="4"></td><td rowspan="4"></td><td>2108~2130</td><td>23</td><td>miRNA: 3' ugCGUCUCGGGCUUUCGGGGGUCa 5'
            || | ||:|: | |||||:|
Target:5' ctGCTG-CCCTGGCACCCCCCGGt 3'</td><td>-25.00</td><td>141.00</td><td rowspan="4"></td></tr>
<tr><td>153~179</td><td>27</td><td>miRNA: 3' ugCGUCU-CGGGCUUU--CGGGGGUCa 5'
            | | | |:| |::| ||||:|||
Target:5' ttGAACATGTCGGGGATCGCCCTCAGc 3'</td><td>-20.40</td><td>140.00</td></tr>
<tr><td>1934~1957</td><td>24</td><td>miRNA: 3' ugcgUCUCGGGCUUUCGGGGGUCa 5'
              :| ||| | | |:|||||
Target:5' gttgGGTGCCGGTCACCTCCCAGc 3'</td><td>-20.00</td><td>140.00</td></tr>
<tr><td>2492~2511</td><td>20</td><td>miRNA: 3' ugcgucUCGGGCUUUCGGGGGUCa 5'
                ||||| ||:|||||
Target:5' atttgtAGCCC----GCTCCCAGa 3'</td><td>-21.30</td><td>140.00</td></tr>
<tr><td rowspan="2">465</td><td rowspan="2">hsa-miR-638</td><td>81~107</td><td>27</td><td>miRNA: 3' ucCGGCGGUGGGCGGGC--GCUAGGGa 5'
            ||||||:|| |||| ||:|||:
Target:5' ccGCCGCCGCCGCCCCGCTCGGTCCTc 3'</td><td>-36.40</td><td>153.00</td><td rowspan="2">?</td></tr>
<tr><td>769~793</td><td>25</td><td>miRNA: 3' ucCGGCGGUGGGCGGGCGCUAGGGa 5'
            ||||||:| |: :|||:||:|
Target:5' ttGCCGCCGCGGGTGTGCGGTCTCg 3'</td><td>-32.60</td><td>147.00</td></tr>
<tr><td rowspan="2">466</td><td rowspan="2">hsa-miR-639</td><td>621~643</td><td>23</td><td>miRNA: 3' uguCGCGAGCGUUGGCGUCGCUa 5'
             |||| | || |||||||
Target:5' tttGCGCCCTCATAAGCAGCGAc 3'</td><td>-18.70</td><td>160.00</td><td rowspan="2">?</td></tr>
<tr><td>2623~2646</td><td>24</td><td>miRNA: 3' ugucgcGAGC-GUUGGCGUCGCUa 5'
                |||| |||:||:||:||
Target:5' atgtttCTCGCCAATCGTAGTGAc 3'</td><td>-22.70</td><td>141.00</td></tr>
<tr><td>467</td><td>hsa-miR-641</td><td>2493~2519</td><td>27</td><td>miRNA: 3' cucCACUGAGAUAGG---AUACAGAAa 5'
             || :| | ||| |||||||
Target:5' tttGTAGCCCGCTCCCAGAATGTCTTa 3'</td><td>-7.80</td><td>149.00</td><td>?</td></tr>
<tr><td>468</td><td>hsa-miR-642b</td><td>399~419</td><td>21</td><td>miRNA: 3' cccAGGGAGAGGUUUACACAga 5'
             |::| | ||:|||||||
Target:5' ttaTTTCAC-CCGAATGTGTac 3'</td><td>-14.50</td><td>142.00</td><td>?</td></tr>
<tr><td>469</td><td>hsa-miR-646</td><td>5~28</td><td>24</td><td>miRNA: 3' cggaGUCUC-----CGUCGACGAa 5'
              |||:| | |||||||
Target:5' gcgcCAGGGTCCTCGGAGCTGCTc 3'</td><td>-17.70</td><td>147.00</td><td>?</td></tr>
<tr><td></td><td>hsa-</td><td></td><td></td><td></td><td></td><td></td><td></td></tr>
</table>

<table>
<tr><td>470</td><td>miR-651</td><td>478~499</td><td>22</td><td>miRNA: 3' guUUUCAGUUCGAAUAGGAUuu 5'
  | | |||| | ||||||
Target:5' tcACAATCAAACAGATCCTAtt 3'</td><td>-8.20</td><td>140.00</td><td></td></tr>
<tr><td>471</td><td>hsa-miR-652</td><td>273~293</td><td>21</td><td>miRNA: 3' gugUUGG-GAUCACCGCGGUAa 5'
   |||: ||| |||||||
Target:5' atgAACTGGGAGT-GCGCCATt 3'</td><td>-15.60</td><td>149.00</td><td></td></tr>
<tr><td></td><td></td><td>743~763</td><td>21</td><td>miRNA: 3' guguugggaucaCCGCGGUAa 5'
            ||||||||
Target:5' gggggttgggcgGGCGCCATc 3'</td><td>-16.70</td><td>145.00</td><td></td></tr>
<tr><td></td><td></td><td>1735~1753</td><td>19</td><td>miRNA: 3' guGUUGGGAUCACCGCGGUaa 5'
  |:||||| | ||||||
Target:5' ccCGACCCT--TAGCGCCAgg 3'</td><td>-19.70</td><td>143.00</td><td></td></tr>
<tr><td>472</td><td>hsa-miR-654-5p</td><td>1690~1714</td><td>25</td><td>miRNA: 3' cgUGUACAAGACGC---CGGGUGgu 5'
  :| | ||||| | ||||||
Target:5' gtGCTTTTTCTGGGAGAGCCCACag 3'</td><td>-15.10</td><td>140.00</td><td></td></tr>
<tr><td>473</td><td>hsa-miR-655</td><td>2808~2831</td><td>24</td><td>miRNA: 3' uuUCUCCAAUUGGU-ACAUA-AUa 5'
  | :| ||||||| ||||| ||
Target:5' caATGGATTAACCACTGTATCTAa 3'</td><td>-10.64</td><td>140.00</td><td></td></tr>
<tr><td>474</td><td>hsa-miR-658</td><td>1839~1868</td><td>30</td><td>miRNA: 3' ugGUUGCCUGGA---UGA--AGGGAGGCGg 5'
  ||:| | ||| :|| |||||:||
Target:5' agCAGCCGTCCTGACGCTGAGCCCTCTGCa 3'</td><td>-25.20</td><td>145.00</td><td></td></tr>
<tr><td>475</td><td>hsa-miR-659</td><td>506~529</td><td>24</td><td>miRNA: 3' accCCUGGGAGG--GACUUGGUUc 5'
   ||| |:||: ||||||||
Target:5' acaGGAACTTCTAAATGAACCAAa 3'</td><td>-17.70</td><td>161.00</td><td></td></tr>
<tr><td>476</td><td>hsa-miR-661</td><td>1273~1294</td><td>22</td><td>miRNA: 3' ugcGCGUCCGGUCUCUGGGUCCGu 5'
   |||:||||||: ||||||
Target:5' cacCGCGGGCCAGGA--CCAGGCt 3'</td><td>-33.60</td><td>148.00</td><td></td></tr>
<tr><td></td><td></td><td>1081~1105</td><td>25</td><td>miRNA: 3' ugcgcgucCGGUCUCU-GGGUCCGu 5'
        ||| ||| ||||||:
Target:5' gcttttgaGCCTCAGACCCCAGGTc 3'</td><td>-18.90</td><td>140.00</td><td></td></tr>
<tr><td>477</td><td>hsa-miR-663</td><td>78~100</td><td>23</td><td>miRNA: 3' cgccagGGC-GCCGCGGGGCGGa 5'
      ||| || ||||||||:
Target:5' gcgccgCCGCCGCCGCCCCGCTc 3'</td><td>-29.20</td><td>148.00</td><td></td></tr>
<tr><td></td><td></td><td>1380~1401</td><td>22</td><td>miRNA: 3' cgCCAGGGCGCCGCGGGGCGga 5'
  |||::| || ||||||
[illegible]</td><td>-23.60</td><td>140.00</td><td></td></tr>
</table>

<table>
<tr><td></td><td></td><td></td><td></td><td>Target:5' gaGGTTTCTGTGCTCCCCGCat 3'</td><td></td><td></td><td></td></tr>
<tr><td>478</td><td>hsa-miR-663b</td><td>1482~1501</td><td>20</td><td>miRNA: 3' ggaGUCCGUGCCGGCCCGGUGg 5'
| | | :|| |||||||
Target:5' cagCTGTCTTGG--GGGCCACa 3'</td><td>-16.70</td><td>147.00</td><td>?</td></tr>
<tr><td>479</td><td>hsa-miR-665</td><td>1873~1895</td><td>23</td><td>miRNA: 3' ucCCCGG--AGU-CGGAGGACCa 5'
||||| || |||||:|||
Target:5' ttGGGCCGGCCAGGCCTCTTGGg 3'</td><td>-26.00</td><td>148.00</td><td>?</td></tr>
<tr><td rowspan="2">480</td><td rowspan="2">hsa-miR-671-5p</td><td>1713~1736</td><td>24</td><td>miRNA: 3' gaGGU-CGGGGAGGUCCCGAAGGa 5'
||: | ||||||||||||||
Target:5' agCCGTGGCCCTCCAGGGCTTCCc 3'</td><td>-38.60</td><td>185.00</td><td rowspan="2">?</td></tr>
<tr><td>1880~1902</td><td>23</td><td>miRNA: 3' gaGGUCGGGGAGGUCCCGAAGGa 5'
|||| ||: :::||||| ||
Target:5' ggCCAGGCCTCTTGGGGCTGCCt 3'</td><td>-28.00</td><td>141.00</td></tr>
<tr><td>481</td><td>hsa-miR-675</td><td>1385~1403</td><td>19</td><td>miRNA: 3' guGACACCCGGGAGAGGCGUGGu 5'
||||| ||| |||||:|
Target:5' ttCTGTG----CTCCCCGCATCt 3'</td><td>-20.20</td><td>147.00</td><td>?</td></tr>
<tr><td>482</td><td>hsa-miR-7-1*</td><td>2429~2450</td><td>22</td><td>miRNA: 3' auaccguCUGACACUAAACAAc 5'
| :||||:||||||
Target:5' gtctgatGTTTGTGGTTTGTTt 3'</td><td>-11.80</td><td>147.00</td><td>?</td></tr>
<tr><td>483</td><td>hsa-miR-711</td><td>2126~2147</td><td>22</td><td>miRNA: 3' gaaUGCAGAGAGGGACCCAGGg 5'
::| || |||||||
Target:5' ccgGTGGCTGGAGCTGGGTCCc 3'</td><td>-21.30</td><td>147.00</td><td>?</td></tr>
<tr><td rowspan="2">484</td><td rowspan="2">hsa-miR-744</td><td>78~99</td><td>22</td><td>miRNA: 3' acgacaauCGGGAUCGGGGCGu 5'
||| |||||||
Target:5' gcgccgccGCCGCCGCCCCGCt 3'</td><td>-17.80</td><td>146.00</td><td rowspan="2">?</td></tr>
<tr><td>2091~2112</td><td>22</td><td>miRNA: 3' acgaCAAUCGGGAUCGGGGCGu 5'
| |:| ||:||||:||
Target:5' tgggGCTGGAGCTGGCCCTGCt 3'</td><td>-21.80</td><td>142.00</td></tr>
<tr><td>485</td><td>hsa-miR-758</td><td>1576~1596</td><td>21</td><td>miRNA: 3' ccAAUCACCUGGUCCAGUGUUu 5'
| ||||| ||||||||||:
Target:5' ctTCAGTGG-CCAGGTCACAGg 3'</td><td>-27.00</td><td>163.00</td><td>?</td></tr>
<tr><td></td><td></td><td>2093~2114</td><td>22</td><td>miRNA: 3' acacagucaaAGUGGGACGACg 5'
| :||||||||
Target:5' gggctggagcTGGCCCTGCTGc 3'</td><td>-18.70</td><td>148.00</td><td></td></tr>
</table>

<table>
<tr><td rowspan="2">486</td><td rowspan="2">hsa-miR-761</td><td>1541~1562</td><td>22</td><td><pre>miRNA: 3' acacagucaaaGUGGGACGACg 5'
                     || |||||||
Target:5' tcctatccgggCAGCCTGCTGg 3'</pre></td><td>-17.80</td><td>147.00</td><td rowspan="2">?</td></tr>
<tr><td>886~908</td><td>23</td><td><pre>miRNA: 3' acACAGUC--AAAGUGGGACGACg 5'
            |||||| ||||| ||||||
Target:5' gaTGTCAGTATTTCA-ACTGCTGt 3'</pre></td><td>-21.20</td><td>145.00</td></tr>
<tr><td rowspan="2">487</td><td rowspan="2">hsa-miR-762</td><td>1996~2019</td><td>24</td><td><pre>miRNA: 3' cgaGCCGGGGC-CG-GGUCGGGg 5'
             | |||| | || ||||||||
Target:5' acaCAGCCCGGTGCTCCCAGCCCt 3'</pre></td><td>-30.10</td><td>163.00</td><td rowspan="2">?</td></tr>
<tr><td>1229~1253</td><td>25</td><td><pre>miRNA: 3' cgaGCCG--GGGCCG-GGUCGGGg 5'
             |||| ||| |: |||:||||
Target:5' aggCGGCCTCCCAGTGCCCGGCCCt 3'</pre></td><td>-30.40</td><td>149.00</td></tr>
<tr><td rowspan="2">488</td><td rowspan="2">hsa-
miR-766</td><td>2082~2102</td><td>21</td><td><pre>miRNA: 3' cgacUCCGACACCCCGACCUCa 5'
              || || |||||||||||
Target:5' ttcaAGCCT-TGGGGCTGGAGc 3'</pre></td><td>-24.00</td><td>169.00</td><td rowspan="2">?</td></tr>
<tr><td>2118~2139</td><td>22</td><td><pre>miRNA: 3' cgacuccgacaCCCCGACCUCa 5'
                     | ||||||||
Target:5' ggcacccccgGTGGCTGGAGc 3'</pre></td><td>-17.30</td><td>147.00</td></tr>
<tr><td>489</td><td>hsa-miR-769-3p</td><td>2145~2171</td><td>27</td><td><pre>miRNA: 3' uuGGUUCUGGG---GCCU-CUAGGGUc 5'
            ||: |:||| :| | |:|||||
Target:5' ccCCGTGGCCCAAGTGCAGGGTCCCAa 3'</pre></td><td>-21.60</td><td>141.00</td><td>?</td></tr>
<tr><td>490</td><td>hsa-miR-874</td><td>2162~2184</td><td>23</td><td><pre>miRNA: 3' agCCAGGGAGC-CCGGUCCCGUc 5'
            |||||| | || ||||||:
Target:5' agGGTCCCAAGAGGGCAGGGCGg 3'</pre></td><td>-33.00</td><td>152.00</td><td>?</td></tr>
<tr><td rowspan="2">491</td><td rowspan="2">hsa-miR-876-5p</td><td>2566~2587</td><td>22</td><td><pre>miRNA: 3' accACUAAGUGUUUCUUUAGGu 5'
             | || ||:| :|||||:|
Target:5' ggtTAATACATATGGAAATTCa 3'</pre></td><td>-11.70</td><td>147.00</td><td rowspan="2">?</td></tr>
<tr><td>43~64</td><td>22</td><td><pre>miRNA: 3' acCACUAAGUGUUUCUUUAGGu 5'
            | | | |: |::|||:|||
Target:5' gcGGGCTCCGGAGGGAAGTCCc 3'</pre></td><td>-14.10</td><td>140.00</td></tr>
<tr><td>492</td><td>hsa-miR-876-3p</td><td>377~398</td><td>22</td><td><pre>miRNA: 3' acUUA-AUGAAACAUUUGGUGGu 5'
            ||| || || :|||||||
Target:5' aaAATGTAAATT-CGAACCACCa 3'</pre></td><td>-11.80</td><td>151.00</td><td>?</td></tr>
</table>

493	hsa-miR-877	1116~1135	20	miRNA: 3' ggGACGCGGUAGAGGAGAUg 5'<br>|||||||| :||||:|:<br>Target:5' ttCTGCGCCACTTCCTTTGt 3'	-25.80	146.00	
494	hsa-miR-877*	1212~1232	21	miRNA: 3' gacccuccUCCCUCUUCUCCu 5'<br>|| ||||:|||| Target:5' tcattgaaAGTGAGAGGAGGc 3'	-14.50	141.00	
495	hsa-miR-885-3p	2096~2116	21	miRNA: 3' auaggUGAUGUGGGGCGACGGa 5'<br>:||: :|||:|||||| Target:5' ctggaGCTG-GCCCTGCTGCCc 3'	-22.20	144.00	
496	hsa-miR-888*	872~895	24	miRNA: 3' aaGUGGGUUUCU--CCACAGUCAg 5'<br>:|::: || | | ||||||| Target:5' ttTATTTTAATATTGATGTCAGTa 3'	-8.30	150.00	
497	hsa-miR-892a	1952~1971	20	miRNA: 3' gaugCGUCUUUCCUGUGUCAc 5'<br>||||:|| |:|||||| Target:5' cccaGCAGGAA-GGCACAGTg 3'	-25.10	152.00	
498	hsa-miR-9	502~531	30	miRNA: 3' agUAUGUC------GAUCUA-UUGGUUUcu 5'<br>|||||| ||| || ||||||| Target:5' gaATACAGGAACTTCTAAATGAACCAAAta 3'	-9.40	151.00	
		502~531	30	miRNA: 3' agUAUGUC------GAUCUA-UUGGUUUcu 5'<br>|||||| ||| || ||||||| Target:5' gaATACAGGAACTTCTAAATGAACCAAAta 3'	-9.40	151.00	
		502~531	30	miRNA: 3' agUAUGUC------GAUCUA-UUGGUUUcu 5'<br>|||||| ||| || ||||||| Target:5' gaATACAGGAACTTCTAAATGAACCAAAta 3'	-9.40	151.00	
499	hsa-miR-920	2400~2421	22	miRNA: 3' auGACGAAGGUG--UCGAGGGg 5'<br>|||| :||| |||||:| Target:5' ccCTGCAGTCACTGAGCTCTCg 3'	-17.70	144.00	
500	hsa-miR-92a-1*	2014~2040	27	miRNA: 3' ucguaACGUUGG----CUAGGGUUGGa 5'<br>| ||||| | |||||||| Target:5' agcccTCCAACCTCTGGCTCCCAACCc 3'	-19.70	160.00	
501	hsa-miR-92a-2*	1239~1261	23	miRNA: 3' caUUAC--GUUGUUUAGGGGUGGg 5'<br>|:|| |::| |||||||| Target:5' ccAGTGCCCGGC-CCTCCCCACCc 3'	-22.30	149.00	
		[illegible]	[illegible]	miRNA: 3' cauuaCGUUGUUUAGGGGUGGg 5'	[illegible]	[illegible]	

<table>
<tr><td></td><td></td><td>2326~2346</td><td>21</td><td>|||:: : |||||||
Target:5' agccgGCAGT-GCCCCCCACCa 3'</td><td>-19.50</td><td>144.00</td><td></td></tr>
<tr><td rowspan="2">502</td><td rowspan="2">hsa-miR-92b*</td><td>1618~1643</td><td>26</td><td>miRNA: 3' gugAC-GUGGCG--CA-GGGCAGGGa 5'
|| |||| | || :|:|||||
Target:5' gggTGCCACCTCAGGTGTCTGTCCCt 3'</td><td>-21.50</td><td>141.00</td><td rowspan="2"></td></tr>
<tr><td>1828~1851</td><td>24</td><td>miRNA: 3' gugACGUGGCGCAG--GGCAGGGa 5'
|||:|| | | ||||||:
Target:5' ctgTGCGCCTCAGCAGCCGTCCTg 3'</td><td>-19.60</td><td>141.00</td></tr>
<tr><td>503</td><td>hsa-miR-93</td><td>982~1004</td><td>23</td><td>miRNA: 3' gaUGG-ACGUGCUUGUCGUGAAac 5'
||| |||| ||:| ||||||
Target:5' tcACCGTGCA-GAGCTGCACTTgg 3'</td><td>-19.80</td><td>152.00</td><td></td></tr>
<tr><td rowspan="2">504</td><td rowspan="2">hsa-miR-93*</td><td>1821~1844</td><td>24</td><td>miRNA: 3' gcCCUUC-ACGAUC-GAGUCGUCa 5'
|||:| ||: | ||||||||
Target:5' ggGGAGGCTGTGCGCCTCAGCAGc 3'</td><td>-24.50</td><td>160.00</td><td rowspan="2"></td></tr>
<tr><td>163~182</td><td>20</td><td>miRNA: 3' gcCCUUCACGAUCGAGUCGUCa 5'
||:| || ||||||||
Target:5' cgGGGATCGC--CCTCAGCAGa 3'</td><td>-22.00</td><td>152.00</td></tr>
<tr><td>505</td><td>hsa-miR-933</td><td>16~40</td><td>25</td><td>miRNA: 3' cccUCUCCA-GAGG--GACGCGUGu 5'
:||| | |||: ||||||:|
Target:5' ctcGGAGCTGCTCTGGCTGCGCGCg 3'</td><td>-23.60</td><td>145.00</td><td></td></tr>
<tr><td>506</td><td>hsa-miR-937</td><td>16~43</td><td>28</td><td>miRNA: 3' ccGUCUC--UCAGUCU----CGCGCCUa 5'
|:||| : || |: |||||||
Target:5' ctCGGAGCTGCTCTGGCTGCGCGCGGAg 3'</td><td>-18.60</td><td>148.00</td><td></td></tr>
<tr><td rowspan="4"></td><td rowspan="4"></td><td>2164~2193</td><td>30</td><td>miRNA: 3' guGGGGGUCU--CG----GAGUCGAGGGGu 5'
:||| ||| || | :|||||||
Target:5' ggTCCCAAGAGGGCAGGGCGGGGCTCCCCa 3'</td><td>-27.41</td><td>154.00</td><td rowspan="4"></td></tr>
<tr><td>1372~1398</td><td>27</td><td>miRNA: 3' gugGGGGUCUC---GGAGUCGAGGGGu 5'
|: | ||| :|| |||||||
Target:5' gtgCTGCTGAGGTTTCTGTGCTCCCCg 3'</td><td>-24.70</td><td>153.00</td></tr>
<tr><td>2007~2037</td><td>31</td><td>miRNA: 3' guGGGGGUC------UCGGAG-UCGAGGGgu 5'
|:||||| | |||| :||||||
Target:5' tgCTCCCAGCCCTCCAACCTCTGGCTCCCaa 3'</td><td>-28.30</td><td>148.00</td></tr>
<tr><td>937~959</td><td>23</td><td>miRNA: 3' guGGGGGUCUCGGAGUCGAGGGGu 5'
|||||||:| | ||:|||:|
Target:5' gtCCCCCAGGGGC-GAGTTCCTCg 3'</td><td>-33.40</td><td>145.00</td></tr>
</table>

<table>
<tr><td rowspan="4">507</td><td rowspan="4">hsa-miR-939</td><td>1706~1737</td><td>32</td><td>miRNA: 3' guGGGGGUC----UC-GGAG---UCGAGGGGu 5'
            ||| ||| :| |||| :|||:|||
Target:5' agCCCACAGCCGTGGCCCTCCAGGGCTTCCCc 3'</td><td>-30.90</td><td>144.00</td></tr>
<tr><td>2317~2343</td><td>27</td><td>miRNA: 3' guGGGGGUCUCGG-AGU--CGAGGGGu 5'
            ||||| ||||| || || ||||
Target:5' ggCCCCCCGAGCCGGCAGTGCCCCCCa 3'</td><td>-35.10</td><td>144.00</td></tr>
<tr><td>1277~1299</td><td>23</td><td>miRNA: 3' gugggGGUCUCGGAGUCGAGGGGu 5'
               ||||: || :||||:||
Target:5' gcgggCCAGGACC-AGGCTCTCCa 3'</td><td>-23.90</td><td>142.00</td></tr>
<tr><td>1234~1258</td><td>25</td><td>miRNA: 3' guGGGGGUCUCGGAGUC--GAGGGGu 5'
            |:||||| ||| |:| ||||||
Target:5' gcCTCCCAGTGCC-CGGCCCTCCCCa 3'</td><td>-34.10</td><td>141.00</td></tr>
<tr><td rowspan="4">508</td><td rowspan="4">hsa-miR-941</td><td>107~133</td><td>27</td><td>miRNA: 3' cgUGUACACGUG-UGU---CGGCCCac 5'
            || ||| |:| ||: ||||||
Target:5' ccACCTGTCCGCTACGCTCGCCGGGgc 3'</td><td>-22.40</td><td>141.00</td></tr>
<tr><td>107~133</td><td>27</td><td>miRNA: 3' cgUGUACACGUG-UGU---CGGCCCac 5'
            || ||| |:| ||: ||||||
Target:5' ccACCTGTCCGCTACGCTCGCCGGGgc 3'</td><td>-22.40</td><td>141.00</td></tr>
<tr><td>107~133</td><td>27</td><td>miRNA: 3' cgUGUACACGUG-UGU---CGGCCCac 5'
            || ||| |:| ||: ||||||
Target:5' ccACCTGTCCGCTACGCTCGCCGGGgc 3'</td><td>-22.40</td><td>141.00</td></tr>
<tr><td>107~133</td><td>27</td><td>miRNA: 3' cgUGUACACGUG-UGU---CGGCCCac 5'
            || ||| |:| ||: ||||||
Target:5' ccACCTGTCCGCTACGCTCGCCGGGgc 3'</td><td>-22.40</td><td>141.00</td></tr>
<tr><td rowspan="3">509</td><td rowspan="3">hsa-miR-942</td><td>56~79</td><td>24</td><td>miRNA: 3' guguaccGGUUUUG--UCUCUUCu 5'
                 ||:|:|| ||:||||
Target:5' ggaagtcCCGAGACAAAGGGAAGc 3'</td><td>-14.60</td><td>141.00</td></tr>
<tr><td>435~456</td><td>22</td><td>miRNA: 3' guGUACCGGUUUUGUCUCUUCu 5'
            | || ||| :||||:||
Target:5' tgCCTGTCCATCTTAGAGGAGg 3'</td><td>-15.80</td><td>140.00</td></tr>
<tr><td>1211~1231</td><td>21</td><td>miRNA: 3' guGUACCGGUUUUG-UCUCUUCu 5'
            ||| | |||:: ||||:||
Target:5' atCATTG--AAAGTGAGAGGAGg 3'</td><td>-10.90</td><td>140.00</td></tr>
</table>

<table>
<tr><td>510</td><td>hsa-miR-96</td><td>2351~2373</td><td>23</td><td><pre>miRNA: 3' ucGUU-UUUACACGAUCACGGUUu 5'
            ||: :| | ||| :||||||:
Target:5' tcCAGCGACTCTGC-GGTGCCAGt 3'</pre></td><td>-15.90</td><td>140.00</td><td>?</td></tr>
<tr><td>511</td><td>hsa-miR-99a*</td><td>2043~2064</td><td>22</td><td><pre>miRNA: 3' gucuGGGUAUCUUCGCUCGAAc 5'
              ||||| |||||||||
Target:5' tctcCCCATCCTAGCGAGCTTg 3'</pre></td><td>-24.50</td><td>166.00</td><td>?</td></tr>
<tr><td>512</td><td>hsa-miR-99b*</td><td>2043~2064</td><td>22</td><td><pre>miRNA: 3' gccuGGGUGUCUGUGCUCGAAc 5'
              ||||:   :|||||||
Target:5' tctcCCCATCCTAGCGAGCTTg 3'</pre></td><td>-20.90</td><td>150.00</td><td>?</td></tr>
</table>

RESULTS

POSITION	SEQUENCE OF TARGET	NAME OF MIRNA	SEQUENCE OF MIRNA	FREE ENERGY	LINK (SEC.STRUCTURE .ps)
744	AAAAGAATTATGGACCCTGGATGGCAATTTGC	hsa-miR-183-3p MIM	gugaauuaccgaagggccauaa	-20.7	image

Results in .CSV format (Right click and 'Save as')

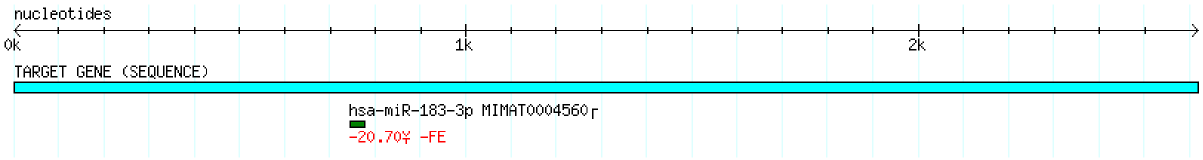

miRNA Target Sites Table View

<table>
<tr><td>Target Sequence</td><td>>
AGATTGCAGAGGGAGACGTGGACGTGAGTGGAGCGGGGCGGTCCCCAGCACACTAGAGGAAGTCGTGCTACCCCCGCGGA
GTTGTCGTGTGTTCTGGATTCATTCCGGCACCACCATGTCGAAGGTTTCCTTTAAGATCACGCTGACGTCGGACCCACGG
CTGCCGTACAAAGTACTCAGTGTTCCTGAAAGTACACCTTTCACAGCAGTCTTAAAGTTTGCAGCAGAAGAATTTAAAGT
TCCTGCTGCAACAAGTGCAATTATTACCAATGATGGAATAGGAATAAATCCTGCACAGACTGCTGGAAATGTTTTTCTAA
AACATGGTTCAGAACTGCGGATTATTCCTAGAGATCGTGTTGGAAGTTGTTAATATCTGCTACTTGGAACATACGATTGC
CTTTCAGAATAAATATTGGTATTTTTTGTTGTTGTAAAATTGAAATCAGGCATTTAACATACTATGAAAACACCAGGAGT
CAATGATTAATGAAAGGTGACTCATCTGTCCCTTTTTGTTGTCCATACTCTTCCTATGAAGAGGGAATGCGTATGAATTA
AGGCTACTACTGTCACAGAAGATCATAGTCTTTGATGCTACCTCACAACACAAACAGGTAGTTCGTTGGGGGCAAATGAA
TTAGCCAACTGTTAACTGGAAGCTTTTGATAATTTTTTTTTTTAGAACAATTTGGAACATTAAAATTTACTGAATCGTAT
ATATTCATCTGAGATAAAAATATAAAAAGAATTATGGACCCTGGATGGCAATTTGCTTGATAGCATCTGATTTGCAGACT
CATAATTTGATTTTTAATTAAATATATAGGTTATGATGAAGTGAATAGACATATCAGTGAACAGTTAACTATATTAAATT
TTTATCATTTACTTTTTTTAAGATTCAGACCTCAGTTATATAAATTTCAGTTTAATATCAACCAAAAAATTAAAATTTTA
ATCTAACCCTTATGTGTATAAATTGGTGTCCCATACCAGCTTTTAATGGTGGACCTATAGAATCCAGTACTTTTAATGGT
GGGAATTTACAGTAGAAGCATCCTTTGCTGAGTTATACATTCCTTTATCAATCTCTTTTGATACAACATTTAAAACAAGT
AGCTTCAAGAAACCACTGGTGTTTTGAGGATAGTATTTCTAAATAGCATTCAGGAACAGAGTATTATTGCACAGATCTGA
AGATCAAAAAAAAAGCTCAAGGAAATACAGATCGGAAGTGCTGATGAGTTATATTTATTGAAAACCCAACTTTTAAGGAAG
TGCTAAGATCAGTCACCCATGTGAATAAGAAGCCAGGAAAGGAAAGATGGGGAAGCCCAGATCACCAGGCTTCTATTAAG
GAGGAAAGCAACAGAGGAAACAGTGAAGGGGAACAGAAGGGGGTAGCAAAGTGTTACAGAAAAGCGGACTGGATAGACAA
AACTGCAGAAGGTGTATGTTGGGGAGAACTGAAAGGGAAAACAAAATACTTGACATAGTCTTAAGTAGAAGAAGGCAGTT
AGAGAAAACAAAGTATCTACTGGCCTTGTCAACATACAGACTTCAAAATACCCCTTATGAGAATCCAAAGAATGATGTGT
GTAAGGGAAGATTTTATTTGCCCTTCCGGAAGAAATCAGTATCTATGCAAATCTTGAAAGACGAAATCAAAGCCCATTAA
TGATTCAGAATCAGTGCTTGACCTCCTGTATTCTGAATGGTGAACTCTGGAAGCAGGGATTGTGTCTGGCTCTTTTTAGA
GCTGGAAATGTAGTGGCTTTCATTAAATACTTGCTGTAAAGTCTTTCTAAGACCAATTATTATCTTAGCATGTTTCAGTA
TCTTCTCTATCATAGGCCCTAAGTTCATTGGGGGAAAAAAAATAAGAAGATTCAACAGAATCAGCATTTGAAGTGTACCA
TTGGTAGTTGTTTATGAAATTACCAGATATTCATAAATGTGACAAATGAACAGCAGGATTATGAATTATCAAAGGAAAAA
GTATTTGCTGAGGTGAAAAAATCTGATGTTTGAGGAAGTTTTTATTTTTATTTATTTGTTTTGTTTTTTTTTTTTTGAGG
CAGACTCTCTGTCGCCAGGCTTCTCCTGCCTCAGCCTCACGAGTAGCTGGGACTACAGGCATGCACCACCACGCCCAGCT
AATTTTTGTATTTTTAGCAGAGACAGGGAGGAAGTTTTTATTTTTATAAACAAAATCTGATGTTCAGAGGCCCCGTTTCT
TACAATAAATGTTGAGTCTTAGTTAAGCAGGAATTTATGAACACCCATTTCCTGACTTTTTGCTTTAATTTAGATTTTTC
TCCATCCTGTTTCTAGCACAAAAATTTGCCTGCTGTGTTACAAAAATAAATTTATGTTGTCCTGTGCCATAAAGTGATAT
ATTTAATATTTTTATTCTTTGGTTTTGAACATTGTAAGTTCCTAAAAACATTTTATTAAACAAGTAGACATTTTGTTATT
AAAAAAATGTGATCTGTAATTTCTTTGTGCAGAATGATTTGAAGTATTGTATTCAGTTTACATGCGTTATTGGTTTATAA
TTAATATCTAATGAAAATACATGTTGTTATATTGTAAACCAAAAAAAAAAAAAAAAAA</td></tr>
</table>

<table>
<tr><th>No.</th><th>miRNA ID</th><th>Location</th><th>Len</th><th>Hybridization</th><th>Minimum Free Energy</th><th>Score</th><th>Profile</th></tr>
<tr><td rowspan="3">1</td><td rowspan="3">hsa-let-7a</td><td>578~605</td><td>28</td><td>miRNA: 3' uugaUA-UGUUGG-----AUGAUGGAGu 5'
              || |:|::| |:|||||||
Target:5' gaagATCATAGTCTTTGATGCTACCTCa 3'</td><td>-15.60</td><td>150.00</td><td rowspan="3"></td></tr>
<tr><td>578~605</td><td>28</td><td>miRNA: 3' uugaUA-UGUUGG-----AUGAUGGAGu 5'
              || |:|::| |:|||||||
Target:5' gaagATCATAGTCTTTGATGCTACCTCa 3'</td><td>-15.60</td><td>150.00</td></tr>
<tr><td>578~605</td><td>28</td><td>miRNA: 3' uugaUA-UGUUGG-----AUGAUGGAGu 5'
              || |:|::| |:|||||||
Target:5' gaagATCATAGTCTTTGATGCTACCTCa 3'</td><td>-15.60</td><td>150.00</td></tr>
<tr><td>2</td><td>hsa-let-7b</td><td>579~605</td><td>27</td><td>miRNA: 3' uugguGUGUUGG-----AUGAUGGAGu 5'
               ||:|::| |:|||||||
Target:5' aagatCATAGTCTTTGATGCTACCTCa 3'</td><td>-18.50</td><td>153.00</td><td></td></tr>
</table>

<table>
<tr><td>3</td><td>hsa-let-7c</td><td>578~605</td><td>28</td><td>miRNA: 3' uuggUA-UGUUGG-----AUGAUGGAGu 5'
    || |:|::| |:|||||||
Target:5' gaagATCATAGTCTTTGATGCTACCTCa 3'</td><td>-17.22</td><td>150.00</td><td></td></tr>
<tr><td>4</td><td>hsa-let-7d</td><td>588~605</td><td>18</td><td>miRNA: 3' uuGAUACGUUGGAUGAUGGAGa 5'
  || || | |:|||||||
Target:5' gtCTTTG--A--TGCTACCTCa 3'</td><td>-12.70</td><td>148.00</td><td></td></tr>
<tr><td rowspan="2">5</td><td rowspan="2">hsa-let-7e</td><td>578~605</td><td>28</td><td>miRNA: 3' uugaUA-UGUUGGAG-----GAUGGAGu 5'
    || |:|::||: |||||||
Target:5' gaagATCATAGTCTTTGATGCTACCTCa 3'</td><td>-15.10</td><td>150.00</td><td rowspan="2"></td></tr>
<tr><td>2087~2113</td><td>27</td><td>miRNA: 3' uuGAUA--UGUU---GGAGGAUGGAGu 5'
  ||:| ||:   :|||||:||||
Target:5' ctCTGTCGCCAGGCTTCTCCTGCCTCa 3'</td><td>-21.80</td><td>142.00</td></tr>
<tr><td rowspan="2">6</td><td rowspan="2">hsa-let-7f</td><td>578~605</td><td>28</td><td>miRNA: 3' uugaUA-UGUUAG-----AUGAUGGAGu 5'
    || |:|:|| |:|||||||
Target:5' gaagATCATAGTCTTTGATGCTACCTCa 3'</td><td>-16.30</td><td>154.00</td><td rowspan="2"></td></tr>
<tr><td>578~605</td><td>28</td><td>miRNA: 3' uugaUA-UGUUAG-----AUGAUGGAGu 5'
    || |:|:|| |:|||||||
Target:5' gaagATCATAGTCTTTGATGCTACCTCa 3'</td><td>-16.30</td><td>154.00</td></tr>
<tr><td>7</td><td>hsa-let-7g</td><td>586~605</td><td>20</td><td>miRNA: 3' uugACAUGUUUGAUGAUGGAGu 5'
   | | ::| |:|||||||
Target:5' tagTCTTTGA--TGCTACCTCa 3'</td><td>-13.90</td><td>147.00</td><td></td></tr>
<tr><td>8</td><td>hsa-let-7i</td><td>579~605</td><td>27</td><td>miRNA: 3' uugUCGUGUUUG-----AUGAUGGAGu 5'
   | ||:|: | |:|||||||
Target:5' aagATCATAGTCTTTGATGCTACCTCa 3'</td><td>-16.60</td><td>151.00</td><td></td></tr>
<tr><td rowspan="2">9</td><td rowspan="2">hsa-miR-1</td><td>1056~1084</td><td>29</td><td>miRNA: 3' uaUGUAUGAAG------AA-AUGUAAGGu 5'
  :||| |||: || ||||||||
Target:5' aaGCATCCTTTGCTGAGTTATACATTCCt 3'</td><td>-11.30</td><td>158.00</td><td rowspan="2"></td></tr>
<tr><td>1056~1084</td><td>29</td><td>miRNA: 3' uaUGUAUGAAG------AA-AUGUAAGGu 5'
  :||| |||: || ||||||||
Target:5' aaGCATCCTTTGCTGAGTTATACATTCCt 3'</td><td>-11.30</td><td>158.00</td></tr>
<tr><td>10</td><td>hsa-miR-103-2*</td><td>1289~1314</td><td>26</td><td>miRNA: 3' guUCCGUCGUGACAU---UUCUUCGa 5'
  || || | |||: |||||||
Target:5' tcAGTCACCCATGTGAATAAGAAGCc 3'</td><td>-13.00</td><td>157.00</td><td></td></tr>
<tr><td></td><td></td><td>[illegible]</td><td>[illegible]</td><td>miRNA: 3' uggUGUCCUCAGACUCGUAAACu 5'</td><td>[illegible]</td><td>[illegible]</td><td></td></tr>
</table>

11	hsa-miR-105	1892~1911	20	[illegible]<br>||| || || |||||||| Target:5' tcaACA-GAATC--AGCATTTGa 3'	-12.20	159.00	
		1892~1911	20	miRNA: 3' uggUGUCCUCAGACUCGUAAACu 5'<br>||| || || |||||||| Target:5' tcaACA-GAATC--AGCATTTGa 3'	-12.20	159.00	
12	hsa-miR-1179	529~552	24	miRNA: 3' gguuGGUUACUU---UCUUACGaa 5'<br>|| ||||| :|||||| Target:5' tcttCCTATGAAGAGGGAATGCgt 3'	-13.60	141.00	
13	hsa-miR-1182	1838~1861	24	miRNA: 3' caguGUAGGGAGGGU-UCUGGGAg 5'<br>| ||:|| :|| ||:|||| Target:5' gtatCTTCTCTATCATAGGCCCTa 3'	-18.00	147.00	
14	hsa-miR-1183	2108~2140	33	miRNA: 3' acGGGUGAGAGUGGU-AGUG-----GAUGUCac 5'<br>|:|| ||||| | | :| |||||| Target:5' gcCTCAGCCTCACGAGTAGCTGGGACTACAGgc 3'	-18.80	141.00	
		1024~1054	31	miRNA: 3' acggGUGAGAGUGGUAG----UGGAUGUCAc 5'<br>:|||:|:| :: : |::|||||| Target:5' ccagTACTTTTAATGGTGGGAATTTACAGTa 3'	-12.74	141.00	
15	hsa-miR-1197	77~96	20	miRNA: 3' ucUUCAUCUGGUACACAGGAu 5'<br>:||| | :|:|||||:|| Target:5' cgGAGTTG-TCGTGTGTTCTg 3'	-19.80	146.00	
16	hsa-miR-1205	278~297	20	miRNA: 3' gagUUUCGUUUGGGACGUcu 5'<br>::| :|||:|||||| Target:5' ataGGAATAAATCCTGCAca 3'	-12.80	141.00	
17	hsa-miR-1206	2261~2283	23	miRNA: 3' cgAAUUUGU--AGAUGUACUUGu 5'<br>||||:|| | :|||||| Target:5' agTTAAGCAGGAATTTATGAACa 3'	-12.30	141.00	
18	hsa-miR-1207-5p	2090~2111	22	miRNA: 3' ggGGAGGGUCGGA-GGGACGGu 5'<br>:| || :||:| :|||||| Target:5' tgTCGCCAGGCTTCTCCTGCCt 3'	-23.10	143.00	
19	hsa-miR-1208	1367~1386	20	miRNA: 3' aggcggacagacUUGUCACu 5'<br>||||||| Target:5' agcaacagaggaAACAGTGa 3'	-9.72	140.00	
20	hsa-miR-	2175~2197	23	miRNA: 3' gaCCUCUCUGUUCC-CCUUCucc 5'<br>| |||||||:|| |||||	-23.32	140.00	

<table>
<tr><td></td><td>1236</td><td></td><td></td><td>Target:5' taGCAGAGACAGGGAGGAAGttt 3'</td><td></td><td></td><td></td></tr>
<tr><td rowspan="2">21</td><td rowspan="2">hsa-miR-1237</td><td>1379~1401</td><td>23</td><td>miRNA: 3' gacccCCCUGCCUC--GUCUUCCu 5'
| || ||:| |||||||
Target:5' aacaGTGAAGGGGAACAGAAGGg 3'</td><td>-19.60</td><td>155.00</td><td rowspan="2"></td></tr>
<tr><td>1426~1453</td><td>28</td><td>miRNA: 3' gacccCCUGCCU-------CGUCUUCCu 5'
|||: || ||||||||
Target:5' ggactGGATAGACAAAACTGCAGAAGGt 3'</td><td>-19.60</td><td>148.00</td></tr>
<tr><td rowspan="3">22</td><td rowspan="3">hsa-miR-124*</td><td>2265~2285</td><td>21</td><td>miRNA: 3' uaguUCCAGGCGACACUUGUGc 5'
||| : :| |||||||
Target:5' aagcAGGAAT-TTATGAACACc 3'</td><td>-8.40</td><td>145.00</td><td rowspan="3"></td></tr>
<tr><td>2265~2285</td><td>21</td><td>miRNA: 3' uaguUCCAGGCGACACUUGUGc 5'
||| : :| |||||||
Target:5' aagcAGGAAT-TTATGAACACc 3'</td><td>-8.40</td><td>145.00</td></tr>
<tr><td>2265~2285</td><td>21</td><td>miRNA: 3' uaguUCCAGGCGACACUUGUGc 5'
||| : :| |||||||
Target:5' aagcAGGAAT-TTATGAACACc 3'</td><td>-8.40</td><td>145.00</td></tr>
<tr><td>23</td><td>hsa-miR-1243</td><td>1010~1029</td><td>20</td><td>miRNA: 3' gugaGGAUAUUAACUAGGUCAa 5'
||||||: |||||||
Target:5' tggaCCTATAG--AATCCAGTa 3'</td><td>-15.60</td><td>158.00</td><td></td></tr>
<tr><td>24</td><td>hsa-miR-1245</td><td>119~143</td><td>25</td><td>miRNA: 3' uaCAUCC---GGAAA-UCUAGUGaa 5'
| ||| ||||| |||||||
Target:5' tcGAAGGTTTCCTTTAAGATCACgc 3'</td><td>-14.80</td><td>147.00</td><td></td></tr>
<tr><td rowspan="3">25</td><td rowspan="3">hsa-miR-1248</td><td>1489~1516</td><td>28</td><td>miRNA: 3' aaAUCGUGUCACGAAU--AUGUUCUUCCa 5'
|: ||:||| |||| || |||||||
Target:5' ctTGACATAGT-CTTAAGTAGAAGAAGGc 3'</td><td>-18.60</td><td>178.00</td><td rowspan="3"></td></tr>
<tr><td>1338~1365</td><td>28</td><td>miRNA: 3' aaaUCGU-GUCACGAAUAU-GUUCUUCCa 5'
| || ||| |||| || :|||:|||
Target:5' cagATCACCAG-GCTTCTATTAAGGAGGa 3'</td><td>-16.60</td><td>155.00</td></tr>
<tr><td>519~546</td><td>28</td><td>miRNA: 3' aaaucGUGUCACGA--AUAUGUUCUUCCa 5'
||:| | :| |||: ||||:||
Target:5' ttgtcCATACTCTTCCTATG-AAGAGGGa 3'</td><td>-9.80</td><td>143.00</td></tr>
<tr><td>26</td><td>hsa-miR-1251</td><td>1505~1525</td><td>21</td><td>miRNA: 3' ucgcggaaaCCGUCGAUCUCa 5'
|||||:|||||
Target:5' gtagaagaaGGCAGTTAGAGa 3'</td><td>-17.70</td><td>144.00</td><td></td></tr>
</table>

<table>
<tr><td>27</td><td>hsa-miR-1252</td><td>1065~1087</td><td>23</td><td>miRNA: 3' auuuACUUAA-GUUAAAGGAAGa 5'
|||:|| :| ||||||:
Target:5' ttgcTGAGTTATACATTCCTTTa 3'</td><td>-7.70</td><td>142.00</td><td></td></tr>
<tr><td>28</td><td>hsa-miR-1253</td><td>1827~1848</td><td>22</td><td>miRNA: 3' acGU-CCGACUAGAAGAAGAGa 5'
|| | :| | : |||||||
Target:5' agCATGTTTCAGTATCTTCTCt 3'</td><td>-12.00</td><td>147.00</td><td></td></tr>
<tr><td>29</td><td>hsa-miR-1257</td><td>872~893</td><td>22</td><td>miRNA: 3' ccAGUCUU--GGGUAGUAAGUGa 5'
|:| || :::||||||:||
Target:5' taTTA-AATTTTTATCATTTACt 3'</td><td>-10.80</td><td>140.00</td><td></td></tr>
<tr><td>30</td><td>hsa-miR-1260</td><td>1028~1045</td><td>18</td><td>miRNA: 3' accaccgucuCCACCCUa 5'
|||||||
Target:5' tacttttaatGGTGGGAa 3'</td><td>-11.10</td><td>140.00</td><td></td></tr>
<tr><td>31</td><td>hsa-miR-1260b</td><td>1027~1045</td><td>19</td><td>miRNA: 3' uaccaccGUCACCACCCUa 5'
:| ||||||||
Target:5' gtactttTAATGGTGGGAa 3'</td><td>-13.10</td><td>148.00</td><td></td></tr>
<tr><td>32</td><td>hsa-miR-1262</td><td>1277~1300</td><td>24</td><td>miRNA: 3' uaggAAGAUGUUUA---AGUGGGUa 5'
| ||| |:|| |||||||
Target:5' gaagTGCTA-AGATCAGTCACCCAt 3'</td><td>-13.30</td><td>153.00</td><td></td></tr>
<tr><td>33</td><td>hsa-miR-1264</td><td>1592~1615</td><td>24</td><td>miRNA: 3' uugUCCACG-AGUUUAUUCUGAAc 5'
| |||: | |:: ||||:||
Target:5' atgATGTGTGTAAGGGAAGATTTt 3'</td><td>-9.00</td><td>140.00</td><td></td></tr>
<tr><td>34</td><td>hsa-miR-1267</td><td>1877~1899</td><td>23</td><td>miRNA: 3' accccUAAUGU--GAAGUUGUCc 5'
|| | | ||||||||
Target:5' aaaaaATAAGAAGATTCAACAGa 3'</td><td>-8.00</td><td>146.00</td><td></td></tr>
<tr><td>35</td><td>hsa-miR-1268</td><td>2140~2157</td><td>18</td><td>miRNA: 3' ggggGUGGUGGUGCGGGc 5'
|||||||||||||
Target:5' catgCACCACCACGCCCa 3'</td><td>-28.30</td><td>170.00</td><td></td></tr>
<tr><td>36</td><td>hsa-miR-127-5p</td><td>1107~1128</td><td>22</td><td>miRNA: 3' uagucucgggagacUCGAAGUc 5'
|||||||
Target:5' catttaaaacaagtAGCTTCAa 3'</td><td>-9.00</td><td>140.00</td><td></td></tr>
<tr><td>37</td><td>hsa-miR-1273</td><td>2075~2097</td><td>23</td><td>miRNA: 3' uuCUUUCUCAGAACGAAACAGCGGg 5'
||:: || || || |||||||
Target:5' ttGAGGCAGACT--CTCTGTCGCCa 3'</td><td>-16.90</td><td>163.00</td><td></td></tr>
</table>

<table>
<tr><td>38</td><td>hsa-miR-1274a</td><td>2172~2190</td><td>19</td><td>miRNA: 3' accgcgGACU-UGUCCCUg 5'
      | || |||||||
Target:5' ttttagCAGAGACAGGGAg 3'</td><td>-12.40</td><td>144.00</td><td></td></tr>
<tr><td>39</td><td>hsa-miR-1276</td><td>1737~1756</td><td>20</td><td>miRNA: 3' acagaggugucCCGAGAAAu 5'
           ||||||||
Target:5' ggattgtgtctGGCTCTTTt 3'</td><td>-13.20</td><td>145.00</td><td></td></tr>
<tr><td>40</td><td>hsa-miR-1278</td><td>1005~1032</td><td>28</td><td>miRNA: 3' uauCUACU---AUA-CGU--GUCAUGAu 5'
   |:||: ||| | | |||||||
Target:5' aatGGTGGACCTATAGAATCCAGTACTt 3'</td><td>-10.20</td><td>149.00</td><td></td></tr>
<tr><td rowspan="2">41</td><td rowspan="2">hsa-miR-1285</td><td>506~527</td><td>22</td><td>miRNA: 3' ucCAGAGUGAAACAACGGGUcu 5'
  ||| | :|||||||:|||
Target:5' ctGTCCCTTTTTGTTGTCCAta 3'</td><td>-16.60</td><td>144.00</td><td rowspan="2"></td></tr>
<tr><td>506~527</td><td>22</td><td>miRNA: 3' ucCAGAGUGAAACAACGGGUcu 5'
  ||| | :|||||||:|||
Target:5' ctGTCCCTTTTTGTTGTCCAta 3'</td><td>-16.60</td><td>144.00</td></tr>
<tr><td>42</td><td>hsa-miR-1286</td><td>2367~2386</td><td>20</td><td>miRNA: 3' ucccGAGUAGAACCAGGACGu 5'
    :|:|| || ||||||:
Target:5' taaaTTTATGTT-GTCCTGTg 3'</td><td>-12.30</td><td>140.00</td><td></td></tr>
<tr><td>43</td><td>hsa-miR-1288</td><td>25~45</td><td>21</td><td>miRNA: 3' agagGUCUAGUCCCGUCAGGu 5'
    ::|| |:||||:||||
Target:5' tgagTGGAGCGGGGCGGTCCc 3'</td><td>-21.90</td><td>149.00</td><td></td></tr>
<tr><td>44</td><td>hsa-miR-1294</td><td>587~607</td><td>21</td><td>miRNA: 3' ucuguuGUUACGGUUGGAGUGu 5'
      ::||||: |||||||
Target:5' agtcttTGATGCT-ACCTCACa 3'</td><td>-17.10</td><td>155.00</td><td></td></tr>
<tr><td>45</td><td>hsa-miR-1296</td><td>1841~1862</td><td>22</td><td>miRNA: 3' ccucuaccucggucCCGGGAUu 5'
              |||||||
Target:5' tcttctctatcataGGCCCTAa 3'</td><td>-13.30</td><td>140.00</td><td></td></tr>
<tr><td rowspan="3"></td><td rowspan="3"></td><td>972~994</td><td>23</td><td>miRNA: 3' aaauCGUAUUCA--UACAGGGUu 5'
    |:|||| | :|||||||
Target:5' atgtGTATAAATTGGTGTCCCAt 3'</td><td>-14.10</td><td>159.00</td><td rowspan="3"></td></tr>
<tr><td>972~994</td><td>23</td><td>miRNA: 3' aaauCGUAUUCA--UACAGGGUu 5'
    |:|||| | :|||||||
Target:5' atgtGTATAAATTGGTGTCCCAt 3'</td><td>-14.10</td><td>159.00</td></tr>
<tr><td>972~994</td><td>23</td><td>miRNA: 3' aaauCGUAUUCA--UACAGGGUu 5'
    |:|||| | :|||||||</td><td>-14.10</td><td>159.00</td></tr>
</table>

<table>
<tr><td></td><td></td><td></td><td></td><td>[illegible]
Target:5' atgtGTATAAATTGGTGTCCCAt 3'</td><td></td><td></td><td></td></tr>
<tr><td rowspan="8">46</td><td rowspan="8">hsa-miR-1302</td><td>972~994</td><td>23</td><td>miRNA: 3' aaauCGUAUUCA--UACAGGGUu 5'
|:|||| | :|||||||
Target:5' atgtGTATAAATTGGTGTCCCAt 3'</td><td>-14.10</td><td>159.00</td><td rowspan="8"></td></tr>
<tr><td>972~994</td><td>23</td><td>miRNA: 3' aaauCGUAUUCA--UACAGGGUu 5'
|:|||| | :|||||||
Target:5' atgtGTATAAATTGGTGTCCCAt 3'</td><td>-14.10</td><td>159.00</td></tr>
<tr><td>972~994</td><td>23</td><td>miRNA: 3' aaauCGUAUUCA--UACAGGGUu 5'
|:|||| | :|||||||
Target:5' atgtGTATAAATTGGTGTCCCAt 3'</td><td>-14.10</td><td>159.00</td></tr>
<tr><td>972~994</td><td>23</td><td>miRNA: 3' aaauCGUAUUCA--UACAGGGUu 5'
|:|||| | :|||||||
Target:5' atgtGTATAAATTGGTGTCCCAt 3'</td><td>-14.10</td><td>159.00</td></tr>
<tr><td>972~994</td><td>23</td><td>miRNA: 3' aaauCGUAUUCA--UACAGGGUu 5'
|:|||| | :|||||||
Target:5' atgtGTATAAATTGGTGTCCCAt 3'</td><td>-14.10</td><td>159.00</td></tr>
<tr><td>972~994</td><td>23</td><td>miRNA: 3' aaauCGUAUUCA--UACAGGGUu 5'
|:|||| | :|||||||
Target:5' atgtGTATAAATTGGTGTCCCAt 3'</td><td>-14.10</td><td>159.00</td></tr>
<tr><td>972~994</td><td>23</td><td>miRNA: 3' aaauCGUAUUCA--UACAGGGUu 5'
|:|||| | :|||||||
Target:5' atgtGTATAAATTGGTGTCCCAt 3'</td><td>-14.10</td><td>159.00</td></tr>
<tr><td>972~994</td><td>23</td><td>miRNA: 3' aaauCGUAUUCA--UACAGGGUu 5'
|:|||| | :|||||||
Target:5' atgtGTATAAATTGGTGTCCCAt 3'</td><td>-14.10</td><td>159.00</td></tr>
<tr><td rowspan="3">47</td><td rowspan="3">hsa-miR-1304</td><td>1105~1129</td><td>25</td><td>miRNA: 3' guGUAGA--GUG-ACAUCGGAGUUu 5'
|||:| || |||||:||||
Target:5' aaCATTTAAAACAAGTAGCTTCAAg 3'</td><td>-17.50</td><td>146.00</td><td rowspan="3"></td></tr>
<tr><td>2098~2121</td><td>24</td><td>miRNA: 3' guGUAGAGUGAC--A-UCGGAGUuu 5'
| |||| ||| | |||||||
Target:5' ggCTTCTC-CTGCCTCAGCCTCAcg 3'</td><td>-15.80</td><td>141.00</td></tr>
<tr><td>195~216</td><td>22</td><td>miRNA: 3' guGUAGAGUGACAUCGGAGUUu 5'
| |:|||| | ||:||:||
Target:5' caCCTTTCACAGCAGTCTTAAa 3'</td><td>-16.50</td><td>140.00</td></tr>
</table>

<table>
<tr><td>48</td><td>hsa-miR-130a*</td><td>1943~1963</td><td>21</td><td>miRNA: 3' cgUCUGUCAUCGUG-UUACACUu 5'
            |||:| | ||: |||||||
Target:5' ccAGATATT--CATAAATGTGAc 3'</td><td>-11.80</td><td>156.00</td><td></td></tr>
<tr><td>49</td><td>hsa-miR-130b*</td><td>1363~1384</td><td>22</td><td>miRNA: 3' caucaCGUUGUC-CCUUUCUCa 5'
               ||||||| ||||| ||
Target:5' ggaaaGCAACAGAGGAAACAGt 3'</td><td>-17.60</td><td>140.00</td><td></td></tr>
<tr><td>50</td><td>hsa-miR-1323</td><td>2407~2428</td><td>22</td><td>miRNA: 3' ucuuuuACGGGGAGUCAAAACu 5'
                | ::|:|::||||||
Target:5' tattttTATTCTTTGGTTTTGa 3'</td><td>-11.90</td><td>140.00</td><td></td></tr>
<tr><td>51</td><td>hsa-miR-134</td><td>1275~1297</td><td>23</td><td>miRNA: 3' ggggagACCAGU-UGGUCAGUGu 5'
                || | | |:|||||||
Target:5' aggaagTGCTAAGATCAGTCACc 3'</td><td>-13.70</td><td>152.00</td><td></td></tr>
<tr><td rowspan="2">52</td><td rowspan="2">hsa-miR-135a</td><td>1295~1317</td><td>23</td><td>miRNA: 3' aguGUAUCCUUAUUUUUCGGUau 5'
             |||: ||||||:||||||
Target:5' accCATGTGAATAAGAAGCCAgg 3'</td><td>-18.80</td><td>164.00</td><td rowspan="2"></td></tr>
<tr><td>1295~1317</td><td>23</td><td>miRNA: 3' aguGUAUCCUUAUUUUUCGGUau 5'
             |||: ||||||:||||||
Target:5' accCATGTGAATAAGAAGCCAgg 3'</td><td>-18.80</td><td>164.00</td></tr>
<tr><td>53</td><td>hsa-miR-135b</td><td>1295~1317</td><td>23</td><td>miRNA: 3' aguGUAUCCUUACUUUUCGGUau 5'
             |||: |||| |:||||||
Target:5' accCATGTGAATAAGAAGCCAgg 3'</td><td>-15.10</td><td>156.00</td><td></td></tr>
<tr><td rowspan="2">54</td><td rowspan="2">hsa-miR-138</td><td>979~1001</td><td>23</td><td>miRNA: 3' gccgGACUAAGUGUUGUGGUCGa 5'
              :||:| : | |:||||||
Target:5' taaaTTGGTGTCCCATACCAGCt 3'</td><td>-16.30</td><td>143.00</td><td rowspan="2"></td></tr>
<tr><td>979~1001</td><td>23</td><td>miRNA: 3' gccgGACUAAGUGUUGUGGUCGa 5'
              :||:| : | |:||||||
Target:5' taaaTTGGTGTCCCATACCAGCt 3'</td><td>-16.30</td><td>143.00</td></tr>
<tr><td>55</td><td>hsa-miR-140-5p</td><td>1113~1138</td><td>26</td><td>miRNA: 3' gaUGGU-AUCCCA---UUUUGGUGAc 5'
            || | ||| | :||||||||
Target:5' aaACAAGTAGCTTCAAGAAACCACTg 3'</td><td>-13.90</td><td>152.00</td><td></td></tr>
<tr><td>56</td><td>hsa-miR-[illegible]</td><td>1591~1613</td><td>23</td><td>miRNA: 3' agGUUGUG-ACAUGACCUUCUAc 5'
            ::|::: |||| |||||||
Target:5' aaTGATGTGTGTAAGGGAAGATt 3'</td><td>-12.80</td><td>156.00</td><td></td></tr>
</table>

	141*	640~665	26	miRNA: 3' agGUUG--UGACA--UGACCUUCUAc 5'<br>:|:| ||||| |||||||| | Target:5' atTAGCCAACTGTTAACTGGAAGCTt 3'	-18.62	140.00	
57	hsa-miR-141	167~185	19	miRNA: 3' gguagaaAUGGUCUGUCACAAu 5'<br>|||: ||||||| Target:5' tacaaagTACT---CAGTGTTc 3'	-7.70	144.00	
58	hsa-miR-143*	1173~1193	21	miRNA: 3' uggUCUCUACGUCGUGACGUGg 5'<br>| ||| |:| :|:||||| Target:5' ggaACAGA-GTATTATTGCACa 3'	-15.50	142.00	
59	hsa-miR-144	2576~2596	21	miRNA: 3' ucAUGUAGUA-GAUAUGACAu 5'<br>||||| | :||||:||| Target:5' aaTACATGTTGTTATATTGTa 3'	-11.50	146.00	
60	hsa-miR-145	1735~1767	33	miRNA: 3' ucCCUAA---GGACC-------CUUUUGACCUg 5'<br>||||| :|||| |:|:||||| Target:5' agGGATTGTGTCTGGCTCTTTTTAGAGCTGGAa 3'	-22.20	141.00	
		639~661	23	miRNA: 3' ucccuaaggacccuuUUGACCUg 5'<br>||||||| Target:5' aattagccaactgttAACTGGAa 3'	-11.30	140.00	
61	hsa-miR-1468	1409~1429	21	miRNA: 3' guCGCUUUGUCCGUUUGCCUc 5'<br>|:| |||| ||:|||| Target:5' aaGTGTTACAGAAAAGCGGAc 3'	-18.80	143.00	
62	hsa-miR-146a*	2209~2230	22	miRNA: 3' gacUUCUUGACUUAAAGUCUCc 5'<br>|| | |||| ||||||| Target:5' aacAAAATCTGATGTTCAGAGg 3'	-16.30	163.00	
63	hsa-miR-148b*	314~337	24	miRNA: 3' cggacucacaUAUU--GUCUUGAa 5'<br>||:: ||||||| Target:5' tttctaaaacATGGTTCAGAACTg 3'	-7.30	142.00	
64	hsa-miR-150*	1900~1921	22	miRNA: 3' gacagggggucggACAUGGUc 5'<br>||||||| Target:5' atcagcatttgaagTGTACCAt 3'	-8.40	140.00	
65	hsa-miR-151-3p	2518~2541	24	miRNA: 3' ggAGUUCCU--CG-AAGUCAGAUc 5'<br>| ||| | |: ||||||:|| Target:5' ttTGAAGTATTGTATTCAGTTTAc 3'	-8.00	141.00	
				[illegible]			

<table>
<tr><td rowspan="2">66</td><td rowspan="2">hsa-miR-153</td><td>1629~1650</td><td>22</td><td>miRNA: 3' cuagugaaaaCACUGAUACGUu 5'
|| |||||||
Target:5' gaagaaatcaGTATCTATGCAa 3'</td><td>-10.10</td><td>144.00</td><td rowspan="2"></td></tr>
<tr><td>1629~1650</td><td>22</td><td>miRNA: 3' cuagugaaaaCACUGAUACGUu 5'
|| |||||||
Target:5' gaagaaatcaGTATCTATGCAa 3'</td><td>-10.10</td><td>144.00</td></tr>
<tr><td>67</td><td>hsa-miR-15b*</td><td>1668~1687</td><td>20</td><td>miRNA: 3' aucUCGUCGUUUAUUACUAAGc 5'
||| | | |||||||||
Target:5' caaAGC--CCATTAATGATTCa 3'</td><td>-9.40</td><td>159.00</td><td></td></tr>
<tr><td>68</td><td>hsa-miR-16-2*</td><td>400~419</td><td>20</td><td>miRNA: 3' auuucGUCGUGUCAUUAUAACc 5'
||| |:| |||||||
Target:5' cctttCAGAATA--AATATTGg 3'</td><td>-10.30</td><td>153.00</td><td></td></tr>
<tr><td>69</td><td>hsa-miR-17*</td><td>1428~1449</td><td>22</td><td>miRNA: 3' gauguucacggaagUGACGUCa 5'
|||||||
Target:5' actggatagacaaaACTGCAGa 3'</td><td>-11.70</td><td>140.00</td><td></td></tr>
<tr><td>70</td><td>hsa-miR-182</td><td>1526~1553</td><td>28</td><td>miRNA: 3' ucacacUCA-AGAUG---GUAACGGUUu 5'
||| ||||| | |||:|||
Target:5' aaacaaAGTATCTACTGGCCTTGTCAAc 3'</td><td>-9.90</td><td>146.00</td><td></td></tr>
<tr><td>71</td><td>hsa-miR-1827</td><td>2096~2113</td><td>18</td><td>miRNA: 3' uaaguuAGAUGACGGAGu 5'
||| |||||||
Target:5' caggctTCTCCTGCCTCa 3'</td><td>-15.10</td><td>152.00</td><td></td></tr>
<tr><td>72</td><td>hsa-miR-183</td><td>2365~2391</td><td>27</td><td>miRNA: 3' ucACUUAA----GAU-GGUCACGGUAu 5'
| |||| :|: || |||||||
Target:5' aaTAAATTTATGTTGTCCTGTGCCATa 3'</td><td>-14.60</td><td>154.00</td><td></td></tr>
<tr><td>73</td><td>hsa-miR-183*</td><td>1667~1688</td><td>22</td><td>miRNA: 3' aauacCGGGAAGCCAUUAAGUg 5'
|||| |: |:||||
Target:5' tcaaaGCCCATTAATGATTCAg 3'</td><td>-10.20</td><td>141.00</td><td></td></tr>
<tr><td>74</td><td>hsa-miR-186</td><td>2400~2421</td><td>22</td><td>miRNA: 3' ucggguuuuccucuUAAGAAAc 5'
|||||||
Target:5' tatttaatattttATTCTTTg 3'</td><td>-7.90</td><td>140.00</td><td></td></tr>
<tr><td>75</td><td>hsa-miR-18a</td><td>1043~1066</td><td>24</td><td>miRNA: 3' gaUAGACGUGAUCUACGU-GGAAu 5'
||:| || |||| ||| ||||
Target:5' gaATTTACAGTAGAAGCATCCTTt 3'</td><td>-14.00</td><td>145.00</td><td></td></tr>
<tr><td>76</td><td>hsa-miR-18b*</td><td>1444~1466</td><td>23</td><td>miRNA: 3' cgGUCUUCCCCGUA-AAUCCCgu 5'
||||||| |:|| ||:|||
Target:5' tgCAGAAGGTGTATGTTGGGGag 3'</td><td>-21.70</td><td>144.00</td><td></td></tr>
</table>

<table>
<tr><td>77</td><td>hsa-miR-190</td><td>836~856</td><td>21</td><td>miRNA: 3' uggaUUAUAUAGUUUGUAUAGu 5'
|:|: || |:|||||||
Target:5' atgaAGTGAAT-AGACATATCa 3'</td><td>-11.00</td><td>157.00</td><td></td></tr>
<tr><td>78</td><td>hsa-miR-190b</td><td>835~856</td><td>22</td><td>miRNA: 3' uugggUUA-UAGUUUGUAUAGu 5'
|:| | :|:|||||||
Target:5' gatgaAGTGAATAGACATATCa 3'</td><td>-10.90</td><td>152.00</td><td></td></tr>
<tr><td rowspan="2">79</td><td rowspan="2">hsa-miR-1913</td><td>20~42</td><td>23</td><td>miRNA: 3' accgucgUCGCCUC-CCCCGUCu 5'
||:|||| |||||:|
Target:5' ggacgtgAGTGGAGCGGGGCGGt 3'</td><td>-28.00</td><td>147.00</td><td rowspan="2"></td></tr>
<tr><td>1386~1407</td><td>22</td><td>miRNA: 3' acCGUCGUCGCCUCCCCCGUCu 5'
| :| | | ||||||:||
Target:5' aaGGGGAACAGAAGGGGGTAGc 3'</td><td>-23.10</td><td>140.00</td></tr>
<tr><td>80</td><td>hsa-miR-192</td><td>814~834</td><td>21</td><td>miRNA: 3' ccgacAGUUAAGUAUCCAGUc 5'
| ||| :||||||:|
Target:5' ttaatTAAATATATAGGTTAt 3'</td><td>-8.20</td><td>144.00</td><td></td></tr>
<tr><td rowspan="2">81</td><td rowspan="2">hsa-miR-195*</td><td>401~419</td><td>19</td><td>miRNA: 3' ccucGUCGUGUCGGUUAUAACc 5'
||| |:| |||||||
Target:5' ctttCAGAATA---AATATTGg 3'</td><td>-11.00</td><td>151.00</td><td rowspan="2"></td></tr>
<tr><td>2391~2412</td><td>22</td><td>miRNA: 3' ccUCGUCGUGUCGGUUAUAAcc 5'
||:: :|:| ::||||||
Target:5' aaAGTGATATATTTAATATTtt 3'</td><td>-7.50</td><td>140.00</td></tr>
<tr><td>82</td><td>hsa-miR-196a*</td><td>503~524</td><td>22</td><td>miRNA: 3' gaguccgucaAAGAACAACGGc 5'
||:||||||:|
Target:5' catctgtcccTTTTTGTTGTCc 3'</td><td>-9.90</td><td>140.00</td><td></td></tr>
<tr><td rowspan="3">83</td><td rowspan="3">hsa-miR-199a-3p</td><td>282~306</td><td>25</td><td>miRNA: 3' auUGGUUA-CAC--GUCUGAUGACa 5'
|: ||| || ||||||:|||
Target:5' gaATAAATCCTGCACAGACTGCTGg 3'</td><td>-15.60</td><td>146.00</td><td rowspan="3"></td></tr>
<tr><td>555~573</td><td>19</td><td>miRNA: 3' auUGGUUACACGUCUGAUGACa 5'
|::|| | || |||||||
Target:5' gaATTAA-G-GC-TACTACTGt 3'</td><td>-12.70</td><td>145.00</td></tr>
<tr><td>282~306</td><td>25</td><td>miRNA: 3' auUGGUUA-CAC--GUCUGAUGACa 5'
|: ||| || ||||||:|||
Target:5' gaATAAATCCTGCACAGACTGCTGg 3'</td><td>-15.60</td><td>146.00</td></tr>
</table>

<table>
<tr><td></td><td></td><td>555~573</td><td>19</td><td>miRNA: 3' auUGGUUACACGUCUGAUGACa 5'
            |::|| | || |||||||
Target:5' gaATTAA-G-GC-TACTACTGt 3'</td><td>-12.70</td><td>145.00</td><td></td></tr>
<tr><td rowspan="2">84</td><td rowspan="2">hsa-miR-199b-3p</td><td>282~306</td><td>25</td><td>miRNA: 3' auUGGUUA-CAC--GUCUGAUGACa 5'
            |: ||| || ||||||:|||
Target:5' gaATAAATCCTGCACAGACTGCTGg 3'</td><td>-15.60</td><td>146.00</td><td rowspan="2">?</td></tr>
<tr><td>555~573</td><td>19</td><td>miRNA: 3' auUGGUUACACGUCUGAUGACa 5'
            |::|| | || |||||||
Target:5' gaATTAA-G-GC-TACTACTGt 3'</td><td>-12.70</td><td>145.00</td></tr>
<tr><td>85</td><td>hsa-miR-19a</td><td>775~798</td><td>24</td><td>miRNA: 3' agucaaAACGUA-UCUAAACGUgu 5'
                | |||| ||||||||
Target:5' gcttgaTAGCATCTGATTTGCAga 3'</td><td>-12.20</td><td>141.00</td><td>?</td></tr>
<tr><td>86</td><td>hsa-miR-19b-1*</td><td>1428~1452</td><td>25</td><td>miRNA: 3' cgACCUA-C-GUUUGGACGUUUUga 5'
            ||||| | |||| |||||:||
Target:5' acTGGATAGACAAAACTGCAGAAgg 3'</td><td>-19.30</td><td>145.00</td><td>?</td></tr>
<tr><td rowspan="2">87</td><td rowspan="2">hsa-miR-19b</td><td>775~798</td><td>24</td><td>miRNA: 3' agucaaAACGUA-CCUAAACGUgu 5'
                | |||| ||||||||
Target:5' gcttgaTAGCATCTGATTTGCAga 3'</td><td>-12.00</td><td>141.00</td><td rowspan="2">?</td></tr>
<tr><td>775~798</td><td>24</td><td>miRNA: 3' agucaaAACGUA-CCUAAACGUgu 5'
                | |||| ||||||||
Target:5' gcttgaTAGCATCTGATTTGCAga 3'</td><td>-12.00</td><td>141.00</td></tr>
<tr><td>88</td><td>hsa-miR-200a</td><td>167~185</td><td>19</td><td>miRNA: 3' uguagcaAUGGUCUGUCACAAu 5'
                 |||: |||||||
Target:5' tacaaagTACT---CAGTGTTc 3'</td><td>-8.80</td><td>144.00</td><td>?</td></tr>
<tr><td>89</td><td>hsa-miR-203</td><td>2435~2456</td><td>22</td><td>miRNA: 3' gaUCACCAGGA--UUUGUAAAGUg 5'
            ||| |||| ||||||||:|
Target:5' taAGT--TCCTAAAAACATTTTAt 3'</td><td>-12.10</td><td>154.00</td><td>?</td></tr>
<tr><td rowspan="2">90</td><td rowspan="2">hsa-miR-204</td><td>1452~1479</td><td>28</td><td>miRNA: 3' ucCGUA----UCCUACUG--UUUCCCUu 5'
            |:|| :||| || |||||||
Target:5' gtGTATGTTGGGGAGAACTGAAAGGGAa 3'</td><td>-14.10</td><td>152.00</td><td rowspan="2">?</td></tr>
<tr><td>1~15</td><td>15</td><td>miRNA: 3' uccguaucCUACUGUUUCCCUu 5'
                  ||| :||:|||||
Target:5' -------aGATTGCAGAGGGAg 3'</td><td>-15.40</td><td>142.00</td></tr>
<tr><td></td><td>hsa-</td><td></td><td></td><td></td><td></td><td></td><td></td></tr>
</table>

<table>
<tr><td>91</td><td>miR-2053</td><td>2543~2567</td><td>25</td><td><pre>miRNA: 3' cauuuAUCUCCAA--AUUAAUUGUg 5'
               || |||| |||||||:|
Target:5' tgcgtTATTGGTTTATAATTAATAt 3'</pre></td><td>-7.20</td><td>148.00</td><td>?</td></tr>
<tr><td>92</td><td>hsa-miR-206</td><td>1056~1084</td><td>29</td><td><pre>miRNA: 3' ggUGUGUGAAG-GA------AUGUAAGGu 5'
            :||: |||: || ||||||||
Target:5' aaGCATCCTTTGCTGAGTTATACATTCCt 3'</pre></td><td>-11.30</td><td>154.00</td><td>?</td></tr>
<tr><td>93</td><td>hsa-miR-20a</td><td>1011~1034</td><td>24</td><td><pre>miRNA: 3' gaUGGACGUGAUA-UUCGUGAAAu 5'
            |||| :| || ||:|||||
Target:5' ggACCTATAGAATCCAGTACTTTt 3'</pre></td><td>-10.00</td><td>145.00</td><td>?</td></tr>
<tr><td>94</td><td>hsa-miR-20a*</td><td>1753~1774</td><td>22</td><td><pre>miRNA: 3' gaAAUUCACGAGUAUUACGUCa 5'
            || || ||| : ||||:||
Target:5' ttTTTAGAGCTGGAAATGTAGt 3'</pre></td><td>-12.90</td><td>148.00</td><td>?</td></tr>
<tr><td>95</td><td>hsa-miR-20b</td><td>1011~1034</td><td>24</td><td><pre>miRNA: 3' gaUGGACGUGAUA-CUCGUGAAAc 5'
            |||| :| || ||:|||||
Target:5' ggACCTATAGAATCCAGTACTTTt 3'</pre></td><td>-9.10</td><td>145.00</td><td>?</td></tr>
<tr><td>96</td><td>hsa-miR-20b*</td><td>2117~2139</td><td>23</td><td><pre>miRNA: 3' gaccUUCA-CGGGUAUGAUGUCa 5'
              :||| ||: : |||||||
Target:5' tcacGAGTAGCTGGGACTACAGg 3'</pre></td><td>-13.50</td><td>154.00</td><td>?</td></tr>
<tr><td>97</td><td>hsa-miR-21*</td><td>1119~1144</td><td>26</td><td><pre>miRNA: 3' ugUCGGG-----UAGCUGACCACAAc 5'
            |||:: | | |||||||||
Target:5' gtAGCTTCAAGAAACCACTGGTGTTt 3'</pre></td><td>-13.90</td><td>155.00</td><td>?</td></tr>
<tr><td rowspan="2">98</td><td rowspan="2">hsa-miR-211</td><td>1456~1479</td><td>24</td><td><pre>miRNA: 3' ucCGCUUCCUACUG--UUUCCCUu 5'
            |: ::||| || |||||||
Target:5' atGTTGGGGAGAACTGAAAGGGAa 3'</pre></td><td>-13.90</td><td>154.00</td><td rowspan="2">?</td></tr>
<tr><td>1~15</td><td>15</td><td><pre>miRNA: 3' uccgcuucCUACUGUUUCCCUu 5'
                  ||| :||:|||||
Target:5' -------aGATTGCAGAGGGAg 3'</pre></td><td>-15.40</td><td>142.00</td></tr>
<tr><td rowspan="2">99</td><td rowspan="2">hsa-miR-2113</td><td>2323~2343</td><td>21</td><td><pre>miRNA: 3' cacuGUCUCGGUUCGUGUUUa 5'
              | | :| ||||||||
Target:5' catcCTGTTTCTAGCACAAAa 3'</pre></td><td>-8.60</td><td>149.00</td><td rowspan="2">?</td></tr>
<tr><td>1173~1196</td><td>24</td><td><pre>miRNA: 3' cacUGUCUC--GGU-UCGUGUUUa 5'
             |||||| ::| |||||:|
Target:5' ggaACAGAGTATTATTGCACAGAt 3'</pre></td><td>-16.60</td><td>140.00</td></tr>
<tr><td></td><td></td><td>637~664</td><td>28</td><td><pre>miRNA: 3' aggUAGUC--CU--CAGU--ACCUUCGa 5'
             ||:|| | ||:| |||||||</pre></td><td>-14.70</td><td>149.00</td><td></td></tr>
</table>

<table>
<tr><td rowspan="2">100</td><td rowspan="2">hsa-miR-2115</td><td></td><td></td><td>Target:5' tgaATTAGCCAACTGTTAACTGGAAGCt 3'</td><td></td><td></td><td rowspan="2"></td></tr>
<tr><td>1714~1735</td><td>22</td><td>miRNA: 3' aggUAGUCCUCAGUACCUUCGa 5'
|| : || : |||||||
Target:5' tgaATGGTGAACTCTGGAAGCa 3'</td><td>-9.40</td><td>147.00</td></tr>
<tr><td rowspan="2">101</td><td rowspan="2">hsa-miR-2115*</td><td>1698~1718</td><td>21</td><td>miRNA: 3' gaucGGAGGUACUUAAGACUac 5'
||||| || |||||||
Target:5' ttgaCCTCC-TGTATTCTGAat 3'</td><td>-17.70</td><td>149.00</td><td rowspan="2"></td></tr>
<tr><td>1078~1103</td><td>26</td><td>miRNA: 3' gaucGGAGGUACUU----AAGACUAc 5'
|||::|| || ||:||||
Target:5' cattCCTTTATCAATCTCTTTTGATa 3'</td><td>-9.40</td><td>144.00</td></tr>
<tr><td rowspan="2">102</td><td rowspan="2">hsa-miR-214</td><td>2335~2356</td><td>22</td><td>miRNA: 3' ugacggacagACACGGACGACa 5'
| |||||||||
Target:5' agcacaaaaaTTTGCCTGCTGt 3'</td><td>-16.29</td><td>152.00</td><td rowspan="2"></td></tr>
<tr><td>228~249</td><td>22</td><td>miRNA: 3' ugacggacagaCACGGACGACa 5'
|| |||||||
Target:5' aagaatttaaaGTTCCTGCTGc 3'</td><td>-12.60</td><td>147.00</td></tr>
<tr><td>103</td><td>hsa-miR-215</td><td>814~834</td><td>21</td><td>miRNA: 3' cagacAGUUAAGUAUCCAGUa 5'
| ||| :||||||:|
Target:5' ttaatTAAATATATAGGTTAt 3'</td><td>-10.20</td><td>144.00</td><td></td></tr>
<tr><td>104</td><td>hsa-miR-22*</td><td>213~234</td><td>22</td><td>miRNA: 3' auUUCGAACGGUGACUUCUUGa 5'
|||:|||| :| ||||||:
Target:5' taAAGTTTGCAGCAGAAGAATt 3'</td><td>-17.50</td><td>160.00</td><td></td></tr>
<tr><td>105</td><td>hsa-miR-221</td><td>1753~1775</td><td>23</td><td>miRNA: 3' cuuugggUCGUCUGUUACAUCGa 5'
||| |: |||||||:
Target:5' tttttagAGCTGGAAATGTAGTg 3'</td><td>-11.70</td><td>144.00</td><td></td></tr>
<tr><td>106</td><td>hsa-miR-222</td><td>1754~1775</td><td>22</td><td>miRNA: 3' ugGGUCAUCGGUC--UACAUCGa 5'
::|| |||::| ||||||:
Target:5' ttTTAG-AGCTGGAAATGTAGTg 3'</td><td>-13.20</td><td>140.00</td><td></td></tr>
<tr><td>107</td><td>hsa-miR-223*</td><td>174~198</td><td>25</td><td>miRNA: 3' uuGAGUCGAACAG----UUUAUGUGc 5'
|||||: | || ||:|||||
Target:5' taCTCAGTGT-TCCTGAAAGTACACc 3'</td><td>-14.42</td><td>145.00</td><td></td></tr>
<tr><td>108</td><td>hsa-miR-224</td><td>1761~1780</td><td>20</td><td>miRNA: 3' uuGCCUUGGUGAUCACUGAAc 5'
:|||| :: |||||:|||
Target:5' gcTGGAAATG-TAGTGGCTTt 3'</td><td>-14.00</td><td>146.00</td><td></td></tr>
</table>

<table>
<tr><td>109</td><td>hsa-miR-2276</td><td>779~798</td><td>20</td><td>miRNA: 3' ggAGCGGAGACUGUGAACGUCu 5'
            | || |||| |:||||||
Target:5' gaTAGCATCTG--ATTTGCAGa 3'</td><td>-15.90</td><td>148.00</td><td></td></tr>
<tr><td>110</td><td>hsa-miR-2355-5p</td><td>1445~1466</td><td>22</td><td>miRNA: 3' aacagguaACAUA-GACCCCUa 5'
                  ||||| :||||||
Target:5' gcagaaggTGTATGTTGGGGAg 3'</td><td>-13.90</td><td>141.00</td><td></td></tr>
<tr><td rowspan="2">111</td><td rowspan="2">hsa-miR-23a</td><td>1945~1963</td><td>19</td><td>miRNA: 3' ccUUUAGGGACCGUUACACUa 5'
            | ||:| | |||||||
Target:5' agATATTCAT--AAATGTGAc 3'</td><td>-8.90</td><td>147.00</td><td rowspan="2"></td></tr>
<tr><td>2473~2493</td><td>21</td><td>miRNA: 3' ccuuuagggaccgUUACACUa 5'
                       |||||||
Target:5' ttgttattaaaaaAATGTGAt 3'</td><td>-7.60</td><td>140.00</td></tr>
<tr><td rowspan="2">112</td><td rowspan="2">hsa-miR-23b</td><td>1945~1963</td><td>19</td><td>miRNA: 3' ccauUAGGGACCGUUACACUa 5'
              ||:| | |||||||
Target:5' agatATTCAT--AAATGTGAc 3'</td><td>-7.20</td><td>145.00</td><td rowspan="2"></td></tr>
<tr><td>2473~2493</td><td>21</td><td>miRNA: 3' ccauuagggaccgUUACACUa 5'
                       |||||||
Target:5' ttgttattaaaaaAATGTGAt 3'</td><td>-7.80</td><td>140.00</td></tr>
<tr><td>113</td><td>hsa-miR-23c</td><td>2473~2493</td><td>21</td><td>miRNA: 3' ccCAUUAGUGACCGUUACACUa 5'
            || ||:| |||||||
Target:5' ttGTTATTA-AAAAAATGTGAt 3'</td><td>-9.40</td><td>143.00</td><td></td></tr>
<tr><td>114</td><td>hsa-miR-25</td><td>241~262</td><td>22</td><td>miRNA: 3' agucUGGCUCUGUUCACGUUAc 5'
              :|:| |||||||||||
Target:5' tcctGCTGCAACAAGTGCAATt 3'</td><td>-17.00</td><td>166.00</td><td></td></tr>
<tr><td>115</td><td>hsa-miR-26a</td><td>753~781</td><td>29</td><td>miRNA: 3' ucggaUAGGACCU-------AAUGAACUu 5'
               | |||||| ||:|||||
Target:5' tatggACCCTGGATGGCAATTTGCTTGAt 3'</td><td>-14.81</td><td>141.00</td><td></td></tr>
<tr><td rowspan="2">116</td><td rowspan="2">hsa-miR-26a-1*</td><td>254~282</td><td>29</td><td>miRNA: 3' gcACGUUCAU--UGGU-----UCUUAUCc 5'
            ||||| || |||| :||||||
Target:5' agTGCAATTATTACCAATGATGGAATAGg 3'</td><td>-18.00</td><td>150.00</td><td rowspan="2"></td></tr>
<tr><td>1122~1154</td><td>33</td><td>miRNA: 3' gcACGUUCAUUGGU-----------UCUUAUCc 5'
            | |||| ||||| ||:||||
Target:5' gcTTCAAGAAACCACTGGTGTTTTGAGGATAGt 3'</td><td>-12.70</td><td>140.00</td></tr>
<tr><td></td><td>hsa-</td><td></td><td></td><td>[illegible]</td><td></td><td></td><td></td></tr>
</table>

<table>
<tr><td>117</td><td>miR-26a</td><td>753~781</td><td>29</td><td>miRNA: 3' [illegible] 5'
| |||||| ||:|||||
Target:5' tatggACCCTGGATGGCAATTTGCTTGAt 3'</td><td>-14.81</td><td>141.00</td><td></td></tr>
<tr><td>118</td><td>hsa-miR-26a-2*</td><td>248~282</td><td>35</td><td>miRNA: 3' cuUUGUUCA--UUAGU-----------UCUUAUCc 5'
||||||| |||:| :||||||
Target:5' gcAACAAGTGCAATTATTACCAATGATGGAATAGg 3'</td><td>-15.30</td><td>142.00</td><td></td></tr>
<tr><td>119</td><td>hsa-miR-26b</td><td>1679~1702</td><td>24</td><td>miRNA: 3' uggaUAGGACUUA---AUGAACUu 5'
||:| |||| |:|||||
Target:5' aatgATTCAGAATCAGTGCTTGAc 3'</td><td>-8.00</td><td>145.00</td><td></td></tr>
<tr><td>120</td><td>hsa-miR-26b*</td><td>1376~1397</td><td>22</td><td>miRNA: 3' cucgguUCAUUACCUCUUGUCc 5'
|||:| ||:||||||
Target:5' ggaaacAGTGAAGGGGAACAGa 3'</td><td>-17.80</td><td>152.00</td><td></td></tr>
<tr><td>121</td><td>hsa-miR-27b*</td><td>1842~1867</td><td>26</td><td>miRNA: 3' caAGUGGU--UAGUC--GAUUCGAGa 5'
|| |:| || :| |||||:||
Target:5' ctTCTCTATCATAGGCCCTAAGTTCa 3'</td><td>-10.00</td><td>140.00</td><td></td></tr>
<tr><td rowspan="2">122</td><td rowspan="2">hsa-miR-28-5p</td><td>2423~2444</td><td>22</td><td>miRNA: 3' gaguuaUCUGACACUCGAGGAa 5'
| |:||| ||:||||
Target:5' ttttgaACATTGTAAGTTCCTa 3'</td><td>-11.80</td><td>144.00</td><td rowspan="2"></td></tr>
<tr><td>223~245</td><td>23</td><td>miRNA: 3' gaGUUAUCUGACA-CUCGAGGAa 5'
||: ||| | | ||:||||
Target:5' agCAGAAGAATTTAAAGTTCCTg 3'</td><td>-11.60</td><td>140.00</td></tr>
<tr><td>123</td><td>hsa-miR-2909</td><td>1843~1863</td><td>21</td><td>miRNA: 3' gguucucuacAACCGGGAUUg 5'
| ||||||||
Target:5' ttctctatcaTAGGCCCTAAg 3'</td><td>-12.60</td><td>147.00</td><td></td></tr>
<tr><td>124</td><td>hsa-miR-296-3p</td><td>950~971</td><td>22</td><td>miRNA: 3' ccUCUCGGAGGUGGGUUGGGAg 5'
| | ::|: |:|:||||||
Target:5' ttAAAATTTTAATCTAACCCTt 3'</td><td>-15.40</td><td>144.00</td><td></td></tr>
<tr><td rowspan="2">125</td><td rowspan="2">hsa-miR-297</td><td>1539~1559</td><td>21</td><td>miRNA: 3' guACGUGUACGUGUGUAUGUa 5'
|| | ||: |||||||
Target:5' acTGGCCTTGTCAACATACAg 3'</td><td>-9.20</td><td>151.00</td><td rowspan="2"></td></tr>
<tr><td>376~396</td><td>21</td><td>miRNA: 3' guACG-UGUACGUGUGUAUGUa 5'
||| || || | ||||||:
Target:5' tcTGCTACTTGGA-ACATACGa 3'</td><td>-9.50</td><td>142.00</td></tr>
<tr><td>126</td><td>hsa-miR-299-5p</td><td>2581~2601</td><td>21</td><td>miRNA: 3' uaCAUACACCCUGCCAUUUGGu 5'
|| ||| |: |||||||
Target:5' atGT-TGTTATATTGTAAACCa 3'</td><td>-10.50</td><td>151.00</td><td></td></tr>
</table>

<table>
<tr><td rowspan="3">127</td><td rowspan="3">hsa-miR-29a*</td><td>1619~1640</td><td>22</td><td>miRNA: 3' gacuuguGGUUUUCUUUAGUCa 5'
                 ||::||||||||||
Target:5' tgcccttCCGGAAGAAATCAGt 3'</td><td>-19.70</td><td>167.00</td><td rowspan="3"></td></tr>
<tr><td>427~450</td><td>24</td><td>miRNA: 3' gacuugugGUUUU--CUUUAGUCa 5'
                  :|||| ||||||||
Target:5' tgttgttgTAAAATTGAAATCAGg 3'</td><td>-9.60</td><td>156.00</td></tr>
<tr><td>1884~1905</td><td>22</td><td>miRNA: 3' gaCUUGUGGUUUUCUUUAGUCa 5'
            ||| |:: || :||||||
Target:5' aaGAAGATTCAACAGAATCAGc 3'</td><td>-10.40</td><td>144.00</td></tr>
<tr><td rowspan="3">128</td><td rowspan="3">hsa-miR-29b-1*</td><td>1287~1317</td><td>31</td><td>miRNA: 3' agAUUUGGUGG-------UAUACUUUGGUCg 5'
            | |::|||| ||| |||:||||
Target:5' gaTCAGTCACCCATGTGAATAAGAAGCCAGg 3'</td><td>-18.10</td><td>150.00</td><td rowspan="3"></td></tr>
<tr><td>1617~1640</td><td>24</td><td>miRNA: 3' agauuuGGUGGUAUACUUUGGUCg 5'
                |: ||: | ||||:|||
Target:5' tttgccCTTCCGGAAGAAATCAGt 3'</td><td>-14.60</td><td>142.00</td></tr>
<tr><td>433~450</td><td>18</td><td>miRNA: 3' agAUUUGGUGGUAUACUUUGGUCg 5'
            |||| || |||||:|||
Target:5' tgTAAA-----AT-TGAAATCAGg 3'</td><td>-8.90</td><td>140.00</td></tr>
<tr><td rowspan="2">129</td><td rowspan="2">hsa-miR-29b-2*</td><td>1288~1316</td><td>29</td><td>miRNA: 3' gauUCGGU-GGUACA------CUUUGGUc 5'
             ||:|| |||||| |||:|||
Target:5' atcAGTCACCCATGTGAATAAGAAGCCAg 3'</td><td>-22.90</td><td>149.00</td><td rowspan="2"></td></tr>
<tr><td>1115~1136</td><td>22</td><td>miRNA: 3' gaUUCGGUGGUACACUUUGGUc 5'
            |||: :|: |||||||
Target:5' acAAGTAGCTTCAAGAAACCAc 3'</td><td>-9.40</td><td>148.00</td></tr>
<tr><td>130</td><td>hsa-miR-301a</td><td>1172~1194</td><td>23</td><td>miRNA: 3' cgaaacUGUUAUGAUAACGUGac 5'
                | |:||:||||||||
Target:5' aggaacAGAGTATTATTGCACag 3'</td><td>-13.30</td><td>149.00</td><td></td></tr>
<tr><td>131</td><td>hsa-miR-301b</td><td>1172~1194</td><td>23</td><td>miRNA: 3' cgaaacUGUUAUAGUAACGUGac 5'
                | |:|||:|||||||
Target:5' aggaacAGAGTATTATTGCACag 3'</td><td>-13.20</td><td>149.00</td><td></td></tr>
<tr><td>132</td><td>hsa-miR-302a*</td><td>2008~2035</td><td>28</td><td>miRNA: 3' ucgUUCA----UGUAGG-UGCAAAUUCa 5'
             |:|| | |||: |:||||:||
Target:5' ctgAGGTGAAAAAATCTGATGTTTGAGg 3'</td><td>-12.90</td><td>142.00</td><td></td></tr>
</table>

133	hsa-miR-302d	1888~1910	23	miRNA: 3' ugUGAGUUUGUACCU--UCGUGAAu 5'<br>|:|| |||| | | ||||:|| Target:5' agATTC-AACA-GAATCAGCATTTg 3'	-11.30	141.00	?
134	hsa-miR-3065-5p	409~432	24	miRNA: 3' aggUCGUAGUCACU--AAAACAACu 5'<br>| :||::|| | |||||||| Target:5' ataAATATTGGT-ATTTTTTGTTGt 3'	-10.10	157.00	?
		498~522	25	miRNA: 3' aggucGUAGUCA--CUAAAACAACu 5'<br>|||| || |||||||| Target:5' tgactCATCTGTCCCTTTTTGTTGt 3'	-11.90	156.00	
135	hsa-miR-30a*	1449~1475	27	miRNA: 3' cgaCGUUUGUAGGC-----UGACUUUc 5'<br>|:: |::|: | ||||||| Target:5' aagGTGTATGTTGGGGAGAACTGAAAg 3'	-12.10	143.00	?
136	hsa-miR-30c	1907~1936	30	miRNA: 3' cgACUCUCACAU-----CC-U--ACAAAUGu 5'<br>||| |||||| || | ||||||:<br>Target:5' ttTGA-AGTGTACCATTGGTAGTTGTTTATg 3'	-10.90	142.00	?
		1907~1936	30	miRNA: 3' cgACUCUCACAU-----CC-U--ACAAAUGu 5'<br>||| |||||| || | ||||||:<br>Target:5' ttTGA-AGTGTACCATTGGTAGTTGTTTATg 3'	-10.90	142.00	
137	hsa-miR-30d*	641~662	22	miRNA: 3' cgUCGUUUGUAGACUGACUUUc 5'<br>||| ||| :| ||||:|| Target:5' ttAGCCAACTGTTAACTGGAAg 3'	-14.10	148.00	?
		1449~1475	27	miRNA: 3' cgUCGUUUGUAGAC-----UGACUUUc 5'<br>:|:: |::|: | ||||||| Target:5' aaGGTGTATGTTGGGGAGAACTGAAAg 3'	-10.70	144.00	
138	hsa-miR-30e*	1449~1475	27	miRNA: 3' cgaCAUUUGUAGGC-----UGACUUUc 5'<br>||: |::|: | ||||||| Target:5' aagGTGTATGTTGGGGAGAACTGAAAg 3'	-11.59	147.00	?
139	hsa-miR-3115	1002~1020	19	miRNA: 3' ugGUUGAUCAUUUGGGUAUa 5'<br>:|| |:||::|||:||| Target:5' ttTAA-TGGTGGACCTATAg 3'	-13.00	145.00	?
		786~805	20	miRNA: 3' ugGUUGAUCAUUUGGGUAUa 5'<br>::|:| | |:||:|||| Target:5' tcTGATTTGCAGACTCATAa 3'	-9.80	142.00	
140	hsa-miR-	2239~2262	24	miRNA: 3' gaccGUGAUAU---ACUCAGGAUa 5'<br>[illegible]	-9.80	141.00	?

<table>
<tr><td></td><td>3117</td><td></td><td></td><td>|| || | |||||||:||
Target:5' cttaCAATAAATGTTGAGTCTTAg 3'</td><td></td><td></td><td></td></tr>
<tr><td rowspan="6">141</td><td rowspan="6">hsa-miR-3118</td><td>1276~1297</td><td>22</td><td>miRNA: 3' ucUUAAAAGUAUUACGUCAGUGu 5'
|| | :| :|| |||||||
Target:5' ggAAGTGCTAAGAT-CAGTCACc 3'</td><td>-9.60</td><td>152.00</td><td rowspan="6">?</td></tr>
<tr><td>1276~1297</td><td>22</td><td>miRNA: 3' ucUUAAAAGUAUUACGUCAGUGu 5'
|| | :| :|| |||||||
Target:5' ggAAGTGCTAAGAT-CAGTCACc 3'</td><td>-9.60</td><td>152.00</td></tr>
<tr><td>1276~1297</td><td>22</td><td>miRNA: 3' ucUUAAAAGUAUUACGUCAGUGu 5'
|| | :| :|| |||||||
Target:5' ggAAGTGCTAAGAT-CAGTCACc 3'</td><td>-9.60</td><td>152.00</td></tr>
<tr><td>1276~1297</td><td>22</td><td>miRNA: 3' ucUUAAAAGUAUUACGUCAGUGu 5'
|| | :| :|| |||||||
Target:5' ggAAGTGCTAAGAT-CAGTCACc 3'</td><td>-9.60</td><td>152.00</td></tr>
<tr><td>1276~1297</td><td>22</td><td>miRNA: 3' ucUUAAAAGUAUUACGUCAGUGu 5'
|| | :| :|| |||||||
Target:5' ggAAGTGCTAAGAT-CAGTCACc 3'</td><td>-9.60</td><td>152.00</td></tr>
<tr><td>1276~1297</td><td>22</td><td>miRNA: 3' ucUUAAAAGUAUUACGUCAGUGu 5'
|| | :| :|| |||||||
Target:5' ggAAGTGCTAAGAT-CAGTCACc 3'</td><td>-9.60</td><td>152.00</td></tr>
<tr><td rowspan="2">142</td><td rowspan="2">hsa-miR-3119</td><td>1200~1217</td><td>18</td><td>miRNA: 3' cggUAGUUUCAAUUUUCGGu 5'
|||||| ||||||:
Target:5' aagATCAAA--AAAAAGCTc 3'</td><td>-7.40</td><td>141.00</td><td rowspan="2">?</td></tr>
<tr><td>1200~1217</td><td>18</td><td>miRNA: 3' cggUAGUUUCAAUUUUCGGu 5'
|||||| ||||||:
Target:5' aagATCAAA--AAAAAGCTc 3'</td><td>-7.40</td><td>141.00</td></tr>
<tr><td rowspan="2">143</td><td rowspan="2">hsa-miR-3120</td><td>1774~1798</td><td>25</td><td>miRNA: 3' acgGACAG--A--UGUGAACGACAc 5'
|| || | |:|||||||||
Target:5' tggCTTTCATTAAATACTTGCTGTa 3'</td><td>-14.30</td><td>158.00</td><td rowspan="2">?</td></tr>
<tr><td>503~523</td><td>21</td><td>miRNA: 3' acGGACAGAUGUGAACGACAc 5'
:||||| : :|||:|||
Target:5' caTCTGTCCCTTTTTGTTGTc 3'</td><td>-14.30</td><td>143.00</td></tr>
<tr><td>144</td><td>hsa-miR-3125</td><td>115~134</td><td>20</td><td>miRNA: 3' agAGAGGUGUCGAAGGAGAu 5'
| || | :|:|||||:|
Target:5' caTGTCGAAGGTTTCCTTTa 3'</td><td>-13.20</td><td>142.00</td><td>?</td></tr>
</table>

<table>
<tr><td>145</td><td>hsa-miR-3126-5p</td><td>494~515</td><td>22</td><td>miRNA: 3' acgaagaccGUAGACAGGGAGu 5'
          |||||||||||:
Target:5' aaggtgactCATCTGTCCCTTt 3'</td><td>-20.20</td><td>149.00</td><td></td></tr>
<tr><td rowspan="2">146</td><td rowspan="2">hsa-miR-3129</td><td>551~573</td><td>23</td><td>miRNA: 3' uuUGGUUAGAGAUG-UGAUGACg 5'
  |: |||:   :| |||||||
Target:5' gtATGAATTAAGGCTACTACTGt 3'</td><td>-11.30</td><td>148.00</td><td rowspan="2"></td></tr>
<tr><td>282~306</td><td>25</td><td>miRNA: 3' uuUGGUUAGAGAUG----UGAUGACg 5'
  |: |||| ||:| |||:|||
Target:5' gaATAAATC-CTGCACAGACTGCTGg 3'</td><td>-16.00</td><td>141.00</td></tr>
<tr><td>147</td><td>hsa-miR-3132</td><td>1830~1853</td><td>24</td><td>miRNA: 3' aggAGACUCGAGGAAGAGAUGGgu 5'
   |:| ||: :||||||||:|
Target:5' atgTTTCAGTATCTTCTCTATCat 3'</td><td>-17.60</td><td>141.00</td><td></td></tr>
<tr><td>148</td><td>hsa-miR-3134</td><td>2305~2327</td><td>23</td><td>miRNA: 3' uuaUACAUCAGAAAAUAGGUAGu 5'
   || ||| :|||| ||||||
Target:5' ttaATTTAGATTTTTCTCCATCc 3'</td><td>-10.50</td><td>148.00</td><td></td></tr>
<tr><td>149</td><td>hsa-miR-3136</td><td>1273~1296</td><td>24</td><td>miRNA: 3' uuacugggAUGGAU-AAGUCAGUc 5'
        |:|: | |||||||
Target:5' taaggaagTGCTAAGATCAGTCAc 3'</td><td>-8.40</td><td>143.00</td><td></td></tr>
<tr><td>150</td><td>hsa-miR-3138</td><td>507~527</td><td>21</td><td>miRNA: 3' ugAGGGAGAUGGAGUGACAGGUGu 5'
  |||||:| |:::||||||:
Target:5' tgTCCCTTT---TTGTTGTCCATa 3'</td><td>-21.60</td><td>151.00</td><td></td></tr>
<tr><td rowspan="2">151</td><td rowspan="2">hsa-miR-3143</td><td>1391~1417</td><td>27</td><td>miRNA: 3' gcUUUCUUCGCGAAAUG--UUACAAUa 5'
  | ||||| | | :| |:|||||
Target:5' gaACAGAAGGGGGTAGCAAAGTGTTAc 3'</td><td>-12.90</td><td>145.00</td><td rowspan="2"></td></tr>
<tr><td>2222~2255</td><td>34</td><td>miRNA: 3' gcuuUCUUC---GCGA--AAUG----UUACAAUa 5'
    |||:| ||:| |||| ||||||:
Target:5' gttcAGAGGCCCCGTTTCTTACAATAAATGTTGa 3'</td><td>-10.30</td><td>145.00</td></tr>
<tr><td>152</td><td>hsa-miR-3144-5p</td><td>26~47</td><td>22</td><td>miRNA: 3' gauauauagagaaaCCAGGGGa 5'
              |||||||
Target:5' gagtggagcggggcGGTCCCCa 3'</td><td>-13.20</td><td>140.00</td><td></td></tr>
<tr><td rowspan="2">153</td><td rowspan="2">hsa-miR-3145</td><td>1937~1962</td><td>26</td><td>miRNA: 3' guUAA-GGUUUGUGAGU-UUUAUAga 5'
  ||| |||:|:|:||| ||||:|
Target:5' aaATTACCAGATATTCATAAATGTga 3'</td><td>-14.50</td><td>146.00</td><td rowspan="2"></td></tr>
<tr><td></td><td></td><td></td><td></td><td></td></tr>
</table>

		1515~1538	24	miRNA: 3' guUAAGGUUUGUGAGUUUUAUAGa 5'<br>| |: :|: | ||||:|||| Target:5' gcAGTTAGAGAAAACAAAGTATCt 3'	-13.40	142.00	
154	hsa-miR-3146	1811~1832	22	miRNA: 3' gguaagAAAGAUAGGAUCGUAc 5'<br>|| :||||:|||||| Target:5' gaccaaTTATTATCTTAGCATg 3'	-19.30	152.00	
		2312~2340	29	miRNA: 3' ggUAAGAA-AGAUAG------GAUCGUac 5'<br>|||:|| || ||| |||||| Target:5' agATTTTTCTCCATCCTGTTTCTAGCAca 3'	-12.90	142.00	
155	hsa-miR-3147	2137~2160	24	miRNA: 3' agUGUGGGAGG-AGUGACGGGUUGg 5'<br>:||: | || ||| |||||:| Target:5' agGCATGCACCACCAC-GCCCAGCt 3'	-22.00	141.00	
156	hsa-miR-3148	291~319	29	miRNA: 3' uuCGUGUGUGGUCA-------AAAAAGGu 5'<br>||||| ||:: | |||||:<br>Target:5' ctGCACAGACTGCTGGAAATGTTTTTCTa 3'	-11.10	140.00	
157	hsa-miR-3149	151~172	22	miRNA: 3' uaUGUGUGUGUAUAGGUAUGUUu 5'<br>|| |||: |: ||:||||| Target:5' ggACCCACGGCTG-CCGTACAAa 3'	-16.50	144.00	
158	hsa-miR-3152	2318~2340	23	miRNA: 3' aauaacgGGGAU-AAGAUUGUGu 5'<br>:|||: |||||:||| Target:5' ttctccaTCCTGTTTCTAGCACa 3'	-17.50	143.00	
159	hsa-miR-3154	495~517	23	miRNA: 3' agacgaGGGUUGA-GGGGAAGAc 5'<br>|:|| || :|||||:| Target:5' aggtgaCTCATCTGTCCCTTTTt 3'	-14.60	140.00	
160	hsa-miR-3158	1318~1340	23	miRNA: 3' caggacgUCU-CUCCUUCGGGaa 5'<br>||| |:|||||||| Target:5' aaaggaaAGATGGGGAAGCCCag 3'	-19.40	143.00	
		1318~1340	23	miRNA: 3' caggacgUCU-CUCCUUCGGGaa 5'<br>||| |:|||||||| Target:5' aaaggaaAGATGGGGAAGCCCag 3'	-19.40	143.00	
		1711~1733	23	miRNA: 3' gcugcaccCCGCCUCGCAGACCUUc 5'<br>||: || | ||||||| Target:5' ttctgaatGGT-GAAC-TCTGGAAg 3'	-15.80	147.00	
161	hsa-miR-3180-	1711~1733	23	miRNA: 3' gcugcaccCCGCCUCGCAGACCUUc 5'<br>||: || | |||||||	-15.80	147.00	

<table>
<tr><td></td><td>5p</td><td></td><td></td><td>Target:5' ttctgaatGGT-GAAC-TCTGGAAg 3'</td><td></td><td></td><td></td></tr>
<tr><td></td><td></td><td>1711~1733</td><td>23</td><td>miRNA: 3' gcugcaccCCGCCUCGCAGACCUUc 5'
||: || | |||||||
Target:5' ttctgaatGGT-GAAC-TCTGGAAg 3'</td><td>-15.80</td><td>147.00</td><td></td></tr>
<tr><td rowspan="3">162</td><td rowspan="3">hsa-miR-3182</td><td>562~582</td><td>21</td><td>miRNA: 3' cuGAUG-UGA---UGUCUUCg 5'
|||| ||| |||||||
Target:5' ggCTACTACTGTCACAGAAGa 3'</td><td>-15.70</td><td>155.00</td><td rowspan="3"></td></tr>
<tr><td>1436~1452</td><td>17</td><td>miRNA: 3' cuGAUGUGAUGUCUUCg 5'
| | |||:||||||
Target:5' gaCAAAACTGCAGAAGg 3'</td><td>-15.10</td><td>143.00</td></tr>
<tr><td>1384~1400</td><td>17</td><td>miRNA: 3' cugaugugaUGUCUUCg 5'
|||||||
Target:5' tgaaggggaACAGAAGg 3'</td><td>-7.90</td><td>140.00</td></tr>
<tr><td>163</td><td>hsa-miR-3190</td><td>183~205</td><td>23</td><td>miRNA: 3' agaGACCGGCAGAUGGAAGGUGu 5'
||| || |||||:|||
Target:5' ttcCTGAAAGTACACCTTTCACa 3'</td><td>-18.40</td><td>144.00</td><td></td></tr>
<tr><td>164</td><td>hsa-miR-32</td><td>247~262</td><td>16</td><td>miRNA: 3' acGUUGAAUCAUUACACGUUAu 5'
|||| || |||||||
Target:5' tgCAAC-----AA-GTGCAATt 3'</td><td>-10.10</td><td>146.00</td><td></td></tr>
<tr><td>165</td><td>hsa-miR-32*</td><td>1845~1866</td><td>22</td><td>miRNA: 3' uuuAUAGUGUGUGUGAUUUAAc 5'
|||||:| :| ||||:||
Target:5' ctcTATCATAGGCCCTAAGTTc 3'</td><td>-11.60</td><td>155.00</td><td></td></tr>
<tr><td rowspan="2">166</td><td rowspan="2">hsa-miR-3202</td><td>1604~1628</td><td>25</td><td>miRNA: 3' uaaUUUCGAGAAGA---GGGAAGGu 5'
:||| |:|| | |||||||
Target:5' aggGAAGATTTTATTTGCCCTTCCg 3'</td><td>-16.20</td><td>159.00</td><td rowspan="2"></td></tr>
<tr><td>1604~1628</td><td>25</td><td>miRNA: 3' uaaUUUCGAGAAGA---GGGAAGGu 5'
:||| |:|| | |||||||
Target:5' aggGAAGATTTTATTTGCCCTTCCg 3'</td><td>-16.20</td><td>159.00</td></tr>
<tr><td>167</td><td>hsa-miR-320a</td><td>983~1004</td><td>22</td><td>miRNA: 3' agcgggAGAGUUGGGUCGAAAa 5'
|| || ||||||||
Target:5' ttggtgTCCCATACCAGCTTTt 3'</td><td>-16.90</td><td>156.00</td><td></td></tr>
<tr><td>[illegible]</td><td>hsa-[illegible]</td><td>983~1004</td><td>22</td><td>miRNA: 3' aacgggAGAGUUGGGUCGAAAa 5'
|| || ||||||||
Target:5' ttggtgTCCCATACCAGCTTTt 3'</td><td>-16.60</td><td>156.00</td><td></td></tr>
</table>

<table>
<tr><td>168</td><td>miR-320b</td><td>983~1004</td><td>22</td><td><pre>miRNA: 3' aacgggAGAGUUGGGUCGAAAa 5'
                || || ||||||||
Target:5' ttggtgTCCCATACCAGCTTTt 3'</pre></td><td>-16.60</td><td>156.00</td><td></td></tr>
<tr><td rowspan="2">169</td><td rowspan="2">hsa-miR-320c</td><td>985~1004</td><td>20</td><td><pre>miRNA: 3' ugggAGAGUUGGGUCGAAAa 5'
              || || ||||||||
Target:5' ggtgTCCCATACCAGCTTTt 3'</pre></td><td>-14.10</td><td>156.00</td><td rowspan="2"></td></tr>
<tr><td>985~1004</td><td>20</td><td><pre>miRNA: 3' ugggAGAGUUGGGUCGAAAa 5'
              || || ||||||||
Target:5' ggtgTCCCATACCAGCTTTt 3'</pre></td><td>-14.10</td><td>156.00</td></tr>
<tr><td rowspan="2">170</td><td rowspan="2">hsa-miR-320d</td><td>986~1004</td><td>19</td><td><pre>miRNA: 3' aggAGAGUUGGGUCGAAAa 5'
             || || ||||||||
Target:5' gtgTCCCATACCAGCTTTt 3'</pre></td><td>-12.70</td><td>156.00</td><td rowspan="2"></td></tr>
<tr><td>986~1004</td><td>19</td><td><pre>miRNA: 3' aggAGAGUUGGGUCGAAAa 5'
             || || ||||||||
Target:5' gtgTCCCATACCAGCTTTt 3'</pre></td><td>-12.70</td><td>156.00</td></tr>
<tr><td>171</td><td>hsa-miR-320e</td><td>986~1003</td><td>18</td><td><pre>miRNA: 3' ggaAGAGUUGGGUCGAAa 5'
             || || |||||||
Target:5' gtgTCCCATACCAGCTTt 3'</pre></td><td>-11.30</td><td>151.00</td><td></td></tr>
<tr><td>172</td><td>hsa-miR-326</td><td>1326~1343</td><td>18</td><td><pre>miRNA: 3' gacCUCCUUCCCGGGUCUcc 5'
             |:|||| |||||||
Target:5' gatGGGGAA--GCCCAGAtc 3'</pre></td><td>-18.20</td><td>141.00</td><td></td></tr>
<tr><td>173</td><td>hsa-miR-330-5p</td><td>1320~1343</td><td>24</td><td><pre>miRNA: 3' cggaUUCUGUGUC--CGGGUCUcu 5'
              ||||:: :| |||||||
Target:5' aggaAAGATGGGGAAGCCCAGAtc 3'</pre></td><td>-14.10</td><td>140.00</td><td></td></tr>
<tr><td rowspan="2">174</td><td rowspan="2">hsa-miR-335*</td><td>471~497</td><td>27</td><td><pre>miRNA: 3' ccaGUCCUC-GUU----AUUACUUUUu 5'
             |||||| ||| ||||||||:
Target:5' cacCAGGAGTCAATGATTAATGAAAGg 3'</pre></td><td>-16.80</td><td>157.00</td><td rowspan="2"></td></tr>
<tr><td>444~471</td><td>28</td><td><pre>miRNA: 3' ccAGUCC-----UCGUUAU--UACUUUUu 5'
            ||||| | | ||| |||||||
Target:5' aaTCAGGCATTTAAC-ATACTATGAAAAc 3'</pre></td><td>-7.10</td><td>153.00</td></tr>
<tr><td>175</td><td>hsa-miR-338-3p</td><td>1435~1463</td><td>29</td><td><pre>miRNA: 3' guUGUUUUAGUG------AC-UACGACCu 5'
            |||||| ::| || |||:|||
Target:5' agACAAAACTGCAGAAGGTGTATGTTGGg 3'</pre></td><td>-13.00</td><td>142.00</td><td></td></tr>
<tr><td></td><td>hsa-</td><td></td><td></td><td></td><td></td><td></td><td></td></tr>
</table>

<table>
<tr><td>176</td><td>miR-339-5p</td><td>2165~2189</td><td>25</td><td>miRNA: 3' gcAC-UCGAGGACCUC-CUGUCCCu 5'
|| | :|:: | || |||||||
Target:5' ttTGTATTTTTAGCAGAGACAGGGa 3'</td><td>-16.10</td><td>153.00</td><td></td></tr>
<tr><td>177</td><td>hsa-miR-346</td><td>2064~2086</td><td>23</td><td>miRNA: 3' ucuccguccguacgcCCGUCUGu 5'
|||||||
Target:5' tttttttttttttgaGGCAGACt 3'</td><td>-11.50</td><td>140.00</td><td></td></tr>
<tr><td rowspan="2">178</td><td rowspan="2">hsa-miR-3605-5p</td><td>509~531</td><td>23</td><td>miRNA: 3' ccGAAGGAACGAUAGGUAGGAGu 5'
| |::|||:|:||||| |||
Target:5' tcCCTTTTTGTTGTCCATACTCt 3'</td><td>-18.10</td><td>149.00</td><td rowspan="2"></td></tr>
<tr><td>2308~2330</td><td>23</td><td>miRNA: 3' ccgaaggAACGAUAGGUAGGAgu 5'
|| :| ||||||||
Target:5' atttagaTTTTTCTCCATCCTgt 3'</td><td>-10.50</td><td>140.00</td></tr>
<tr><td>179</td><td>hsa-miR-3606</td><td>1766~1786</td><td>21</td><td>miRNA: 3' uuaAUUUUAUCGGAAGUGAUu 5'
|: |:|:||:||||:||
Target:5' aaaTGTAGTGGCTTTCATTAa 3'</td><td>-15.30</td><td>150.00</td><td></td></tr>
<tr><td>180</td><td>hsa-miR-3607-5p</td><td>2518~2545</td><td>28</td><td>miRNA: 3' ugACU--A-AACG-AAGU--AGUGUACg 5'
||| | |||: |||| |:|||||
Target:5' ttTGAAGTATTGTATTCAGTTTACATGc 3'</td><td>-12.00</td><td>144.00</td><td></td></tr>
<tr><td>181</td><td>hsa-miR-3607-3p</td><td>1037~1053</td><td>17</td><td>miRNA: 3' guaGUCUUUCGCAAAUGUCa 5'
::|:|| |||||||
Target:5' tggTGGGAA---TTTACAGt 3'</td><td>-7.80</td><td>146.00</td><td></td></tr>
<tr><td>182</td><td>hsa-miR-361-5p</td><td>650~672</td><td>23</td><td>miRNA: 3' caugggGACC-UCUAAGACUAUu 5'
|||| || ||:|||||
Target:5' tgttaaCTGGAAGCTTTTGATAa 3'</td><td>-14.40</td><td>148.00</td><td></td></tr>
<tr><td>183</td><td>hsa-miR-3613-3p</td><td>2055~2078</td><td>24</td><td>miRNA: 3' cuucccAACCCGAAAAAAAAAACa 5'
||| :|||||||||||
Target:5' tttgttTTGTTTTTTTTTTTTTGa 3'</td><td>-7.60</td><td>170.00</td><td></td></tr>
<tr><td rowspan="2">184</td><td rowspan="2">hsa-miR-3614-3p</td><td>105~128</td><td>24</td><td>miRNA: 3' uuUUGUGGUUCUAGA-CUUCCGAu 5'
::||||| || | |||||:|
Target:5' ccGGCACCACCATGTCGAAGGTTt 3'</td><td>-16.30</td><td>149.00</td><td rowspan="2"></td></tr>
<tr><td>1221~1242</td><td>22</td><td>miRNA: 3' uuUUGUGGUUCUAGACUUC-CGAu 5'
||:|| ||||| |||| |||
Target:5' gaAATAC--AGATCGGAAGTGCTg 3'</td><td>-16.50</td><td>141.00</td></tr>
<tr><td></td><td></td><td>2333~2356</td><td>24</td><td>miRNA: 3' cgaCGUG--GUCGGACGGACGACu 5'
|||| | ::|||||||||</td><td>-24.00</td><td>161.00</td><td></td></tr>
</table>

<table>
<tr><td></td><td></td><td></td><td></td><td><pre>Target:5' ctaGCACAAAAATTTGCCTGCTGt 3'</pre></td><td></td><td></td><td></td></tr>
<tr><td>185</td><td>hsa-miR-3619</td><td>218~249</td><td>32</td><td><pre>miRNA: 3' cgACGUGGUC-----GGA---C--GGACGACu 5'
            |||| ||| ::| | |||||||
Target:5' ttTGCAGCAGAAGAATTTAAAGTTCCTGCTGc 3'</pre></td><td>-18.70</td><td>146.00</td><td></td></tr>
<tr><td>186</td><td>hsa-miR-363</td><td>241~262</td><td>22</td><td><pre>miRNA: 3' auGUCUACCUAUG-GCACGUUAa 5'
            | | || | || |||||||
Target:5' tcCTGCTGCA-ACAAGTGCAATt 3'</pre></td><td>-7.60</td><td>147.00</td><td></td></tr>
<tr><td>187</td><td>hsa-miR-3646</td><td>1599~1620</td><td>22</td><td><pre>miRNA: 3' acccgaCCCGAGUAAAGUAAAa 5'
                ||| ||||:||||
Target:5' gtgtaaGGGAAGATTTTATTTg 3'</pre></td><td>-8.70</td><td>140.00</td><td></td></tr>
<tr><td>188</td><td>hsa-miR-3648</td><td>60~81</td><td>22</td><td><pre>miRNA: 3' gggAGC-CGCUAGGGGCGCCga 5'
             ||| || | ||||||||
Target:5' aagTCGTGCTACCCCCGCGGag 3'</pre></td><td>-24.00</td><td>146.00</td><td></td></tr>
<tr><td>189</td><td>hsa-miR-3660</td><td>556~577</td><td>22</td><td><pre>miRNA: 3' aguuUUACGA-GAGGACAGUca 5'
              || ||| || ||||||
Target:5' aattAAGGCTACTACTGTCAca 3'</pre></td><td>-10.70</td><td>141.00</td><td></td></tr>
<tr><td>190</td><td>hsa-miR-3662</td><td>869~892</td><td>24</td><td><pre>miRNA: 3' guaGUCAGUGAUGAGUAGUAAAag 5'
             :| | | | :|:|||||||
Target:5' ctaTATTAAATTTTTATCATTTac 3'</pre></td><td>-7.40</td><td>141.00</td><td></td></tr>
<tr><td>191</td><td>hsa-miR-3664</td><td>1164~1183</td><td>20</td><td><pre>miRNA: 3' ugaGUACUCACUUC-UGUCUCAa 5'
             ||| | |:| |||||||
Target:5' tagCAT---TCAGGAACAGAGTa 3'</pre></td><td>-9.60</td><td>148.00</td><td></td></tr>
<tr><td>192</td><td>hsa-miR-3667-3p</td><td>2177~2196</td><td>20</td><td><pre>miRNA: 3' uuUCUGGGUACCUCUCCUUCca 5'
            ||| || |:|||||||
Target:5' gcAGAGACA--GGGAGGAAGtt 3'</pre></td><td>-16.60</td><td>140.00</td><td></td></tr>
<tr><td>193</td><td>hsa-miR-3669</td><td>88~108</td><td>21</td><td><pre>miRNA: 3' auAUAAGGCAUAUGUAUAAGGCa 5'
            |:|||:| || | ||||||
Target:5' tgTGTTCTGGATTC--ATTCCGg 3'</pre></td><td>-15.70</td><td>140.00</td><td></td></tr>
<tr><td rowspan="2">194</td><td rowspan="2">hsa-miR-367*</td><td>1354~1375</td><td>22</td><td><pre>miRNA: 3' ucucaacguauaaUCGUUGUCa 5'
                       ||||||||
Target:5' tattaaggaggaaAGCAACAGa 3'</pre></td><td>-10.70</td><td>145.00</td><td rowspan="2"></td></tr>
<tr><td>206~228</td><td>23</td><td><pre>miRNA: 3' ucUCA-ACGUAUAAUCGUUGUCa 5'
            ||| | | :|| |||:|||
Target:5' gcAGTCTTAAAGTTTGCAGCAGa 3'</pre></td><td>-8.50</td><td>140.00</td></tr>
</table>

<table>
<tr><td>195</td><td>hsa-miR-367</td><td>240~262</td><td>23</td><td>miRNA: 3' aguGGUAACGAU-UUCACGUUAa 5'
|:: ||| | |||||||||
Target:5' ttcCTGCTGCAACAAGTGCAATt 3'</td><td>-14.60</td><td>163.00</td><td>?</td></tr>
<tr><td rowspan="3">196</td><td rowspan="3">hsa-miR-3672</td><td>1483~1504</td><td>22</td><td>miRNA: 3' uucUACAAAAUGUACUCAGAGUa 5'
|| || |||| |||||:|
Target:5' aaaATACTTGACAT-AGTCTTAa 3'</td><td>-8.60</td><td>147.00</td><td rowspan="3">?</td></tr>
<tr><td>193~215</td><td>23</td><td>miRNA: 3' uucuacAAAAUGUACUCAGAGUa 5'
||| ||| |||||:|
Target:5' tacaccTTTCACAGCAGTCTTAa 3'</td><td>-7.30</td><td>145.00</td></tr>
<tr><td>2232~2262</td><td>31</td><td>miRNA: 3' uucUACAAAAUGU--------ACUCAGAGUa 5'
:| | ||||| |||||||:|
Target:5' cccGTTTCTTACAATAAATGTTGAGTCTTAg 3'</td><td>-10.20</td><td>142.00</td></tr>
<tr><td rowspan="2">197</td><td rowspan="2">hsa-miR-3673</td><td>1064~1085</td><td>22</td><td>miRNA: 3' auAAGGCAU-AUAUGUAAGGua 5'
| |:| : ||||||||||
Target:5' ttTGCTGAGTTATACATTCCtt 3'</td><td>-13.90</td><td>143.00</td><td rowspan="2">?</td></tr>
<tr><td>90~108</td><td>19</td><td>miRNA: 3' auAAGGCAUAUAUGUAAGGUa 5'
|||:| || | ||||||:
Target:5' tgTTCTGGAT-T-CATTCCGg 3'</td><td>-14.40</td><td>141.00</td></tr>
<tr><td>198</td><td>hsa-miR-3675-5p</td><td>2212~2237</td><td>26</td><td>miRNA: 3' cuUUAG---AGAUGUCUUCGGGGUAu 5'
|||| | | ||||:|||||:|
Target:5' aaAATCTGATGTTCAGAGGCCCCGTt 3'</td><td>-24.10</td><td>157.00</td><td>?</td></tr>
<tr><td>199</td><td>hsa-miR-3675-3p</td><td>334~356</td><td>23</td><td>miRNA: 3' aacCCCCUCA-AGGAAUCUCUAc 5'
| ||| | |:| |||||||
Target:5' actGCGGATTATTCCTAGAGATc 3'</td><td>-12.10</td><td>159.00</td><td>?</td></tr>
<tr><td>200</td><td>hsa-miR-3677</td><td>141~161</td><td>21</td><td>miRNA: 3' ccGGCACCGGUCUCGGGUGCUc 5'
|:| |:|:|| ||||||:
Target:5' cgCTGACGTCGGA-CCCACGGc 3'</td><td>-23.50</td><td>143.00</td><td>?</td></tr>
<tr><td>201</td><td>hsa-miR-3679-3p</td><td>1857~1877</td><td>21</td><td>miRNA: 3' cuacUUCUAAUGACCCCCCUUc 5'
||| | |:| |||||||
Target:5' ccctAAGTTCATT-GGGGGAAa 3'</td><td>-14.60</td><td>157.00</td><td>?</td></tr>
<tr><td>202</td><td>hsa-miR-3680*</td><td>1629~1652</td><td>24</td><td>miRNA: 3' ggaugagGGUCCCAG-UACGUUUu 5'
:||| || |||||||
Target:5' gaagaaaTCAGTATCTATGCAAAt 3'</td><td>-10.00</td><td>152.00</td><td>?</td></tr>
<tr><td></td><td>hsa-</td><td></td><td></td><td>miRNA: 3' agUAAAUGAA-------AGA--GAAUGUCUa 5'</td><td></td><td></td><td></td></tr>
</table>

<table>
<tr><td>203</td><td>miR-3686</td><td>769~799</td><td>31</td><td>miRNA: 3' [illegible]
||||:||| ||| :||:||||
Target:5' caATTTGCTTGATAGCATCTGATTTGCAGAc 3'</td><td>-10.41</td><td>146.00</td><td></td></tr>
<tr><td>204</td><td>hsa-miR-3688</td><td>1790~1811</td><td>22</td><td>miRNA: 3' ucucacCGUUUCAGAAAGGUau 5'
|:||||||||||:|
Target:5' cttgctGTAAAGTCTTTCTAag 3'</td><td>-15.20</td><td>140.00</td><td></td></tr>
<tr><td>205</td><td>hsa-miR-369-3p</td><td>1170~1188</td><td>19</td><td>miRNA: 3' uuUCUAGUUGGUACAUAAUAa 5'
||: ||| : |||||||
Target:5' tcAGG--AACAGAGTATTATt 3'</td><td>-7.60</td><td>151.00</td><td></td></tr>
<tr><td>206</td><td>hsa-miR-370</td><td>1957~1978</td><td>22</td><td>miRNA: 3' ugguccaaggugggGUCGUCCg 5'
|||||||
Target:5' atgtgacaaatgaaCAGCAGGa 3'</td><td>-13.70</td><td>140.00</td><td></td></tr>
<tr><td>207</td><td>hsa-miR-371-5p</td><td>2016~2035</td><td>20</td><td>miRNA: 3' ucacggGGGUGUCAAACUCa 5'
:|:: |||||||
Target:5' aaaaaaTCTGATGTTTGAGg 3'</td><td>-9.90</td><td>142.00</td><td></td></tr>
<tr><td>208</td><td>hsa-miR-372</td><td>1888~1910</td><td>23</td><td>miRNA: 3' ugcGAGUUUACAGCGUCGUGAAa 5'
:|||| | |||||:||
Target:5' agaTTCAACAGAATCAGCATTTg 3'</td><td>-9.90</td><td>140.00</td><td></td></tr>
<tr><td>209</td><td>hsa-miR-373*</td><td>1128~1149</td><td>22</td><td>miRNA: 3' ccUUUCGCGGGGGUAAAACUCa 5'
||| | |: ::|||||||
Target:5' agAAACCACTGGTGTTTTGAGg 3'</td><td>-8.80</td><td>156.00</td><td></td></tr>
<tr><td rowspan="2">210</td><td rowspan="2">hsa-miR-373</td><td>1011~1033</td><td>23</td><td>miRNA: 3' ugUGGG-GUUUUAGCUUCGUGAAg 5'
|||: :|:|||| ||:||||
Target:5' ggACCTATAGAATC-CAGTACTTt 3'</td><td>-14.30</td><td>148.00</td><td rowspan="2"></td></tr>
<tr><td>1888~1910</td><td>23</td><td>miRNA: 3' ugUGGG--GUUUUAGCUUCGUGAAg 5'
|::| ||:|||| ||||:||
Target:5' agATTCAACAGAATC--AGCATTTg 3'</td><td>-11.90</td><td>147.00</td></tr>
<tr><td>211</td><td>hsa-miR-374a</td><td>2572~2597</td><td>26</td><td>miRNA: 3' gugaaUA-GUCCAAC---AUAAUAUu 5'
|| || |||| ||||:||
Target:5' tgaaaATACATGTTGTTATATTGTAa 3'</td><td>-7.50</td><td>141.00</td><td></td></tr>
<tr><td>212</td><td>hsa-miR-374b*</td><td>366~384</td><td>19</td><td>miRNA: 3' uuACUAUUAUGUUGGACGAUuc 5'
|| |||| |:||||||
Target:5' gtTGTTAAT---ATCTGCTAct 3'</td><td>-7.40</td><td>141.00</td><td></td></tr>
<tr><td></td><td></td><td>216~237</td><td>22</td><td>miRNA: 3' auGAGUAUCUUCCUCUUAGAUg 5'
:|:: || || |||||:||
Target:5' agTTTGCAGCAGAAGAATTTAa 3'</td><td>-9.50</td><td>148.00</td><td></td></tr>
</table>

<table>
<tr><td></td><td></td><td></td><td></td><td>[illegible]</td><td></td><td></td><td></td></tr>
<tr><td rowspan="2">213</td><td rowspan="2">hsa-miR-376a*</td><td>2254~2278</td><td>25</td><td>miRNA: 3' augAGUAUC--UUCCU-CUUAGAUg 5'
|| ||| ||| | ||||:||
Target:5' gagTCTTAGTTAAGCAGGAATTTAt 3'</td><td>-7.80</td><td>145.00</td><td rowspan="2"></td></tr>
<tr><td>1028~1050</td><td>23</td><td>miRNA: 3' auGAGUAUCUUC-CUCUUAGAUg 5'
||: || :| |:||||:||
Target:5' taCTTTTAATGGTGGGAATTTAc 3'</td><td>-13.40</td><td>140.00</td></tr>
<tr><td>214</td><td>hsa-miR-376b</td><td>1917~1938</td><td>22</td><td>miRNA: 3' uuGUACCUAAAAGGAGAUACUa 5'
||| |:| |: |:|||||
Target:5' acCATTGGTAGTTGTTTATGAa 3'</td><td>-8.30</td><td>144.00</td><td></td></tr>
<tr><td>215</td><td>hsa-miR-377</td><td>2339~2361</td><td>23</td><td>miRNA: 3' ugUUUUCAACGGA-AACACACUa 5'
|||| |||||| ||||| |
Target:5' caAAAATTTGCCTGCTGTGTTAc 3'</td><td>-11.82</td><td>144.00</td><td></td></tr>
<tr><td>216</td><td>hsa-miR-381</td><td>2151~2172</td><td>22</td><td>miRNA: 3' ugucucUCGAACGGGAACAUAu 5'
|||| :::||||||
Target:5' acgcccAGCTAATTTTTGTATt 3'</td><td>-9.80</td><td>140.00</td><td></td></tr>
<tr><td>217</td><td>hsa-miR-3912</td><td>2527~2549</td><td>23</td><td>miRNA: 3' ugUACAGGUAUAA--UACGCAAu 5'
:| |:|| || |||||||
Target:5' ttGTATTCAGTTTACATGCGTTa 3'</td><td>-10.10</td><td>153.00</td><td></td></tr>
<tr><td>218</td><td>hsa-miR-3916</td><td>104~134</td><td>31</td><td>miRNA: 3' gacUCUUGGUCGGUAAAG------AAGGAGAa 5'
:| |||| |||| || |||||:|
Target:5' tccGGCACCA-CCATGTCGAAGGTTTCCTTTa 3'</td><td>-15.20</td><td>148.00</td><td></td></tr>
<tr><td>219</td><td>hsa-miR-3918</td><td>1842~1862</td><td>21</td><td>miRNA: 3' ucAGAGGUAGACGCCGGGAca 5'
||||:||| ||||||
Target:5' ctTCTCTATCATAGGCCCTaa 3'</td><td>-20.10</td><td>147.00</td><td></td></tr>
<tr><td>220</td><td>hsa-miR-3921</td><td>2208~2230</td><td>23</td><td>miRNA: 3' uguuccgUAUACCAUGAGUCUCu 5'
|| || |::||||||
Target:5' aaacaaaATCTGATGTTCAGAGg 3'</td><td>-13.40</td><td>144.00</td><td></td></tr>
<tr><td rowspan="2">221</td><td rowspan="2">hsa-miR-3928</td><td>224~246</td><td>23</td><td>miRNA: 3' cggCUUC-GAGGUUCCAAGGAgg 5'
|||| |::|| ||||||
Target:5' gcaGAAGAATTTAAAGTTCCTgc 3'</td><td>-14.50</td><td>143.00</td><td rowspan="2"></td></tr>
<tr><td>167~189</td><td>23</td><td>miRNA: 3' cgGCUUCGAG-GUUCCAAGGAgg 5'
| |||: | ||: ||||||
Target:5' taCAAAGTACTCAGTGTTCCTga 3'</td><td>-11.30</td><td>140.00</td></tr>
</table>

<table>
<tr><td>222</td><td>hsa-miR-3929</td><td>2094~2118</td><td>25</td><td>miRNA: 3' ucacCAGAUGAG--UGUAGUCGGAg 5'
| || ||| :| |||||||
Target:5' gccaGGCTTCTCCTGCCTCAGCCTc 3'</td><td>-18.70</td><td>157.00</td><td></td></tr>
<tr><td>223</td><td>hsa-miR-3934</td><td>172~201</td><td>30</td><td>miRNA: 3' gacgGAGUCA-AAGG-------UGUGGAcu 5'
|||||| |||| ||||||
Target:5' agtaCTCAGTGTTCCTGAAAGTACACCTtt 3'</td><td>-16.91</td><td>142.00</td><td></td></tr>
<tr><td rowspan="2">224</td><td rowspan="2">hsa-miR-3935</td><td>1520~1541</td><td>22</td><td>miRNA: 3' caccgaccacgagCAUAGAUGu 5'
||||||||
Target:5' tagagaaaacaaaGTATCTACt 3'</td><td>-8.60</td><td>145.00</td><td rowspan="2"></td></tr>
<tr><td>356~381</td><td>26</td><td>miRNA: 3' cacCGACC----ACGAGCAUAGAUGu 5'
|:||| ||:| |||||:|
Target:5' cgtGTTGGAAGTTGTTAATATCTGCt 3'</td><td>-13.20</td><td>141.00</td></tr>
<tr><td>225</td><td>hsa-miR-3936</td><td>1556~1577</td><td>22</td><td>miRNA: 3' acguaGACGGUAUGUGGGGAAu 5'
|| | | |:|||||||
Target:5' acagaCTTCAAAATACCCCTTa 3'</td><td>-15.60</td><td>157.00</td><td></td></tr>
<tr><td rowspan="3">226</td><td rowspan="3">hsa-miR-3938</td><td>529~549</td><td>21</td><td>miRNA: 3' ggcccaAUAGAUGUUCCCUUAa 5'
||| | :|||||||
Target:5' tcttccTAT-GAAGAGGGAATg 3'</td><td>-9.10</td><td>147.00</td><td rowspan="3"></td></tr>
<tr><td>1460~1481</td><td>22</td><td>miRNA: 3' ggCCCAAUAGAUGUUCCCUUaa 5'
||| | ||: ||||||
Target:5' tgGGGAGAACTGAAAGGGAAaa 3'</td><td>-16.10</td><td>144.00</td></tr>
<tr><td>1590~1611</td><td>22</td><td>miRNA: 3' ggccCAAUAGAUGUUCCCUUaa 5'
| |:| |::|||||||
Target:5' gaatGATGTGTGTAAGGGAAga 3'</td><td>-12.70</td><td>142.00</td></tr>
<tr><td>227</td><td>hsa-miR-3941</td><td>1579~1604</td><td>26</td><td>miRNA: 3' auacUAGGAGUC--A--ACACACAUu 5'
|||| || | ||||||||
Target:5' gagaATCCAAAGAATGATGTGTGTAa 3'</td><td>-13.60</td><td>154.00</td><td></td></tr>
<tr><td>228</td><td>hsa-miR-3942</td><td>1167~1192</td><td>26</td><td>miRNA: 3' uaAAGUCCAUUGUC-----AUAACGaa 5'
|||||| ||||| ||||||
Target:5' caTTCAGG-AACAGAGTATTATTGCac 3'</td><td>-15.20</td><td>151.00</td><td></td></tr>
<tr><td rowspan="2"></td><td rowspan="2">hsa-</td><td>2574~2593</td><td>20</td><td>miRNA: 3' uguccgGUAGACACAAUAUAa 5'
||| | ||||||||
Target:5' aaaataCATGT-TGTTATATt 3'</td><td>-7.90</td><td>154.00</td><td rowspan="2"></td></tr>
<tr><td></td><td></td><td>[illegible]</td><td></td><td></td></tr>
</table>

<table>
<tr><td rowspan="2">229</td><td rowspan="2">miR-410</td><td>1234~1254</td><td>21</td><td>miRNA: 3' ugUC-CGGUAGACACAAUAUAa 5'
  || ||:: || |||||||
Target:5' gaAGTGCTG-ATGAGTTATATt 3'</td><td>-8.50</td><td>150.00</td><td rowspan="2">?</td></tr>
<tr><td>902~922</td><td>21</td><td>miRNA: 3' uguccggUAGACACAAUAUAa 5'
       | || |||||||
Target:5' gattcagACCTCAGTTATATa 3'</td><td>-8.40</td><td>146.00</td></tr>
<tr><td>230</td><td>hsa-miR-411*</td><td>2343~2364</td><td>22</td><td>miRNA: 3' ccAAUCACCUGGCACAAUGUau 5'
  || | :|:||||||||
Target:5' aaTTTGCCTGCTGTGTTACAaa 3'</td><td>-12.30</td><td>140.00</td><td>?</td></tr>
<tr><td>231</td><td>hsa-miR-423-5p</td><td>1607~1627</td><td>21</td><td>miRNA: 3' uuUCAGAGCGAGAGACGGGGAGu 5'
  || :| :| |:|||||:||
Target:5' gaAGATT--TTATTTGCCCTTCc 3'</td><td>-13.90</td><td>141.00</td><td>?</td></tr>
<tr><td rowspan="2">232</td><td rowspan="2">hsa-miR-4259</td><td>1250~1271</td><td>22</td><td>miRNA: 3' aggacuggggaUCUGGGUUGAc 5'
           | ||||||||
Target:5' atatttattgaAAACCCAACTt 3'</td><td>-11.10</td><td>147.00</td><td rowspan="2">?</td></tr>
<tr><td>2139~2161</td><td>23</td><td>miRNA: 3' aggAC-UGGGGAUCUGGGUUGAc 5'
   || ||| | | :||||:||
Target:5' gcaTGCACCACCACGCCCAGCTa 3'</td><td>-15.50</td><td>143.00</td></tr>
<tr><td>233</td><td>hsa-miR-4262</td><td>1705~1721</td><td>17</td><td>miRNA: 3' gucCAUCAGACUUACag 5'
   ||| ||||||||
Target:5' cctGTATTCTGAATGgt 3'</td><td>-10.50</td><td>142.00</td><td>?</td></tr>
<tr><td>234</td><td>hsa-miR-4267</td><td>1751~1766</td><td>16</td><td>miRNA: 3' cacgguggCUCGACCu 5'
        |||||||
Target:5' tctttttaGAGCTGGa 3'</td><td>-13.00</td><td>140.00</td><td>?</td></tr>
<tr><td>235</td><td>hsa-miR-4269</td><td>1732~1749</td><td>18</td><td>miRNA: 3' cgGUCCCGACAGACACGGACg 5'
  ||||| | ||||:|||
Target:5' agCAGGGAT---TGTGTCTGg 3'</td><td>-21.10</td><td>144.00</td><td>?</td></tr>
<tr><td>236</td><td>hsa-miR-4274</td><td>288~305</td><td>18</td><td>miRNA: 3' gucccCCUCCCUGACGAc 5'
     | | |||||||
Target:5' atcctGCACAGACTGCTg 3'</td><td>-12.90</td><td>141.00</td><td>?</td></tr>
<tr><td rowspan="2">237</td><td rowspan="2">hsa-miR-4277</td><td>318~338</td><td>21</td><td>miRNA: 3' cacaUG-ACACGAGUCUUGACg 5'
    || || |:|||||||||
Target:5' taaaACATG-GTTCAGAACTGc 3'</td><td>-20.00</td><td>160.00</td><td rowspan="2">?</td></tr>
<tr><td>1452~1472</td><td>21</td><td>miRNA: 3' caCAUGACACGAGUCUUGACg 5'
  |||: || |||||||
Target:5' gtGTATGTTGGGGAGAACTGa 3'</td><td>-13.40</td><td>151.00</td></tr>
</table>

<table>
<tr><td></td><td></td><td>1745~1765</td><td>21</td><td>miRNA: 3' cacaUGACACGAGUCUUGACg 5'
:|| | :|:|||:|||
Target:5' tctgGCTCTTTTTAGAGCTGg 3'</td><td>-14.70</td><td>141.00</td><td></td></tr>
<tr><td>238</td><td>hsa-miR-429</td><td>1138~1158</td><td>21</td><td>miRNA: 3' ugcCAAAAUGGUCUGUCAUAAu 5'
|||||: :||:||||||
Target:5' ggtGTTTTG-AGGATAGTATTt 3'</td><td>-13.80</td><td>150.00</td><td></td></tr>
<tr><td>239</td><td>hsa-miR-4291</td><td>235~250</td><td>16</td><td>miRNA: 3' ucgaCAAGGACGACuu 5'
||||||||||
Target:5' taaaGTTCCTGCTGca 3'</td><td>-16.40</td><td>140.00</td><td></td></tr>
<tr><td>240</td><td>hsa-miR-4298</td><td>973~994</td><td>22</td><td>miRNA: 3' gacggaggaggaggACAGGGUc 5'
|||||||
Target:5' tgtgtataaattggTGTCCCAt 3'</td><td>-10.60</td><td>140.00</td><td></td></tr>
<tr><td>241</td><td>hsa-miR-4299</td><td>2083~2099</td><td>17</td><td>miRNA: 3' cgGAGAGUACAGUGGUCg 5'
||||| ||||:||||
Target:5' gaCTCTC-TGTCGCCAGg 3'</td><td>-22.70</td><td>151.00</td><td></td></tr>
<tr><td></td><td></td><td>1332~1349</td><td>18</td><td>miRNA: 3' cggaGAGUACAGUGGUCg 5'
| || |||||||
Target:5' gaagCCCAGATCACCAGg 3'</td><td>-13.60</td><td>146.00</td><td></td></tr>
<tr><td>242</td><td>hsa-miR-4307</td><td>1511~1532</td><td>22</td><td>miRNA: 3' ccUUUGUC---CUUUUUUGUaa 5'
|::||| ||:||||||
Target:5' gaAGGCAGTTAGAGAAAACAaa 3'</td><td>-9.90</td><td>141.00</td><td></td></tr>
<tr><td>243</td><td>hsa-miR-4311</td><td>1080~1099</td><td>20</td><td>miRNA: 3' guGUGAGUCG--AGAGAAAg 5'
| :|:| | |||||||
Target:5' ttCCTTTATCAATCTCTTTt 3'</td><td>-7.60</td><td>146.00</td><td></td></tr>
<tr><td>244</td><td>hsa-miR-4316</td><td>2104~2121</td><td>18</td><td>miRNA: 3' guGGUCGA-UCGGAGUGg 5'
|:: || ||||||||
Target:5' tcCTGCCTCAGCCTCACg 3'</td><td>-16.10</td><td>151.00</td><td></td></tr>
<tr><td>245</td><td>hsa-miR-4320</td><td>1009~1026</td><td>18</td><td>miRNA: 3' ucCUUCGAUGUCUUAGGg 5'
|:| |||:|||||||
Target:5' gtGGACCTATAGAATCCa 3'</td><td>-17.40</td><td>164.00</td><td></td></tr>
<tr><td></td><td></td><td>1569~1587</td><td>19</td><td>miRNA: 3' uccuucGAUG-UCUUAGGg 5'
:||: ||||||
Target:5' taccccTTATGAGAATCCa 3'</td><td>-9.00</td><td>144.00</td><td></td></tr>
</table>

<table>
<tr><td></td><td></td><td>1884~1904</td><td>21</td><td>miRNA: 3' ucCUUC---GAUGUCUUAGgg 5'
|||| | ||||||||
Target:5' aaGAAGATTCAACAGAATCag 3'</td><td>-12.40</td><td>140.00</td><td></td></tr>
<tr><td>246</td><td>hsa-miR-4325</td><td>243~260</td><td>18</td><td>miRNA: 3' agUGACUCUGUUCACGUu 5'
:||| |||||||||
Target:5' ctGCTGCAACAAGTGCAa 3'</td><td>-16.60</td><td>160.00</td><td></td></tr>
<tr><td>247</td><td>hsa-miR-4326</td><td>2174~2195</td><td>22</td><td>miRNA: 3' cagaccCUCUGU--CUCCUUgu 5'
|||||| ||||||
Target:5' ttagcaGAGACAGGGAGGAAgt 3'</td><td>-16.20</td><td>140.00</td><td></td></tr>
<tr><td>248</td><td>hsa-miR-4328</td><td>1425~1446</td><td>22</td><td>miRNA: 3' uuagGACC---C--UUUUGACc 5'
|||| | |||||||
Target:5' cggaCTGGATAGACAAAACTGc 3'</td><td>-8.10</td><td>143.00</td><td></td></tr>
<tr><td>249</td><td>hsa-miR-4330</td><td>716~734</td><td>19</td><td>miRNA: 3' cguuccgaGACUAGACUCc 5'
:| |||||||
Target:5' cgtatataTTCATCTGAGa 3'</td><td>-7.70</td><td>143.00</td><td></td></tr>
<tr><td>250</td><td>hsa-miR-449c</td><td>373~403</td><td>31</td><td>miRNA: 3' ugUCGGCGAU----CGU-UAUG-UGACGGAu 5'
| |:|||| | | |||| |:|||||
Target:5' atATCTGCTACTTGGAACATACGATTGCCTt 3'</td><td>-15.60</td><td>149.00</td><td></td></tr>
<tr><td rowspan="2">251</td><td rowspan="2">hsa-miR-450b-5p</td><td>1167~1194</td><td>28</td><td>miRNA: 3' auAAGUCCUUGU------AUAACGUUUu 5'
|||||||||| ||||||| |
Target:5' caTTCAGGAACAGAGTATTATTGCACAg 3'</td><td>-18.50</td><td>150.00</td><td rowspan="2"></td></tr>
<tr><td>2576~2599</td><td>24</td><td>miRNA: 3' auAAGUCC--UUGUAUAACGUUUu 5'
| || | : :||||||:|||
Target:5' aaTACATGTTGTTATATTGTAAAc 3'</td><td>-7.20</td><td>142.00</td></tr>
<tr><td>252</td><td>hsa-miR-452</td><td>1364~1385</td><td>22</td><td>miRNA: 3' agucaaaggagaCGUUUGUCAa 5'
| |||||||
Target:5' gaaagcaacagaGGAAACAGTg 3'</td><td>-7.40</td><td>142.00</td><td></td></tr>
<tr><td>253</td><td>hsa-miR-452*</td><td>2494~2518</td><td>25</td><td>miRNA: 3' gugAAUGAAGAA--ACGUC-UACUc 5'
| |:||||| ||||| ||||
Target:5' ctgTAATTTCTTTGTGCAGAATGAt 3'</td><td>-12.10</td><td>141.00</td><td></td></tr>
<tr><td>254</td><td>hsa-miR-454</td><td>1172~1194</td><td>23</td><td>miRNA: 3' ugggauaUUCGUUAUAACGUGau 5'
:||:| ||||||||
Target:5' aggaacaGAGTATTATTGCACag 3'</td><td>-11.30</td><td>144.00</td><td></td></tr>
<tr><td>255</td><td>hsa-[illegible]</td><td>957~979</td><td>23</td><td>miRNA: 3' uacacacaacgcacAUACACAUa 5'
[illegible]</td><td>-7.60</td><td>145.00</td><td></td></tr>
</table>

<table>
<tr><td>[illegible]</td><td>[illegible] 466</td><td>[illegible]</td><td>[illegible]</td><td>[illegible]
              ||||||||
Target:5' tttaatctaaccctTATGTGTAt 3'</td><td>[illegible]</td><td>[illegible]</td></tr>
<tr><td>256</td><td>hsa-miR-485-5p</td><td>2098~2119</td><td>22</td><td>miRNA: 3' cuuAAGUAGUGCCG-GUCGGAGa 5'
             ||| || :| | |||||||
Target:5' ggcTTC-TCCTGCCTCAGCCTCa 3'</td><td>-14.60</td><td>154.00</td></tr>
<tr><td>257</td><td>hsa-miR-486-3p</td><td>492~513</td><td>22</td><td>miRNA: 3' uaggaCA-UGACUCGACGGGGc 5'
               || ||| | |||:|||
Target:5' gaaagGTGACTCATCTGTCCCt 3'</td><td>-11.80</td><td>140.00</td></tr>
<tr><td rowspan="2">258</td><td rowspan="2">hsa-miR-488</td><td>183~204</td><td>22</td><td>miRNA: 3' cuGGUUCUUUAU-CGGAAAGUu 5'
            || : ||:|| |||||||
Target:5' ttCCTGAAAGTACACCTTTCAc 3'</td><td>-9.80</td><td>155.00</td></tr>
<tr><td>381~407</td><td>27</td><td>miRNA: 3' cuGGUUCUU-UAU-----CGGAAAGUu 5'
            |: :||| ||| ||||||||
Target:5' taCTTGGAACATACGATTGCCTTTCAg 3'</td><td>-12.60</td><td>155.00</td></tr>
<tr><td>259</td><td>hsa-miR-494</td><td>1816~1837</td><td>22</td><td>miRNA: 3' cuccAAAGGGCACAUACAAAGu 5'
              || :|: | |||||||
Target:5' attaTTATCTTAGCATGTTTCa 3'</td><td>-7.10</td><td>150.00</td></tr>
<tr><td>260</td><td>hsa-miR-495</td><td>1908~1933</td><td>26</td><td>miRNA: 3' uuCUUCACGUGGUA--CA--AACAAa 5'
            ||||||:||||| || |||||
Target:5' ttGAAGTGTACCATTGGTAGTTGTTt 3'</td><td>-20.00</td><td>146.00</td></tr>
<tr><td>261</td><td>hsa-miR-496</td><td>1769~1793</td><td>25</td><td>miRNA: 3' cucUAACCG---GUACAUUAUGAGu 5'
             | |||| ||| ||||||:
Target:5' tgtAGTGGCTTTCATTAAATACTTg 3'</td><td>-8.00</td><td>143.00</td></tr>
<tr><td rowspan="4">262</td><td rowspan="4">hsa-miR-499-5p</td><td>192~215</td><td>24</td><td>miRNA: 3' uuUGU----AGUGACGUUCAGAAUu 5'
            ||| |||| || |||||||
Target:5' gtACACCTTTCACAGC-AGTCTTAa 3'</td><td>-13.10</td><td>160.00</td></tr>
<tr><td>2240~2262</td><td>23</td><td>miRNA: 3' uuUG-UAGUGAC-GUUCAGAAUu 5'
            || || | || ::|||||||
Target:5' ttACAATAAATGTTGAGTCTTAg 3'</td><td>-8.60</td><td>155.00</td></tr>
<tr><td>1482~1504</td><td>23</td><td>miRNA: 3' uuUGUAGUGAC-GU-UCAGAAUu 5'
            | || :|| || |||||||
Target:5' caAAATACTTGACATAGTCTTAa 3'</td><td>-9.60</td><td>151.00</td></tr>
<tr><td>1785~1807</td><td>23</td><td>miRNA: 3' uuUGU-AGUGACG-UUCAGAAuu 5'
            |:| |::|||: |||||||
Target:5' aaATACTTGCTGTAAAGTCTTtc 3'</td><td>-10.90</td><td>143.00</td></tr>
</table>

<table>
<tr><td rowspan="1">263</td><td>hsa-miR-500a</td><td>1720~1742</td><td>23</td><td><pre>miRNA: 3' agagUGGGUCCAUCGUUCCUAAu 5'
              ||:| || ||||:|||||
Target:5' gtgaACTCTGGAAGCAGGGATTg 3'</pre></td><td>-22.30</td><td>159.00</td><td></td></tr>
<tr><td>264</td><td>hsa-miR-500b</td><td>1724~1741</td><td>18</td><td><pre>miRNA: 3' ugGGUCCAUCGUUCCUAa 5'
            :| || ||||:||||
Target:5' acTCTGGAAGCAGGGATt 3'</pre></td><td>-19.90</td><td>144.00</td><td></td></tr>
<tr><td>265</td><td>hsa-miR-502-5p</td><td>1720~1740</td><td>21</td><td><pre>miRNA: 3' auCGUGGGUCUAUCGUUCCUa 5'

            | ||:| |: ||||:|||
Target:5' gtGAACTCTGGAAGCAGGGAt 3'</pre></td><td>-18.10</td><td>147.00</td><td></td></tr>
<tr><td>266</td><td>hsa-miR-503</td><td>225~251</td><td>27</td><td><pre>miRNA: 3' gaCGUCUUGA----CAAGGGCGACGau 5'
            | ||||:| |||||:|||||
Target:5' caGAAGAATTTAAAGTTCCTGCTGCaa 3'</pre></td><td>-20.40</td><td>143.00</td><td></td></tr>
<tr><td>267</td><td>hsa-miR-507</td><td>242~262</td><td>21</td><td><pre>miRNA: 3' aaGUGAGGUUUUCCACGUUuu 5'
            ::|| ||| | ||||||
Target:5' ccTGCTGCAACAAGTGCAAtt 3'</pre></td><td>-9.50</td><td>143.00</td><td></td></tr>
<tr><td rowspan="2">268</td><td rowspan="2">hsa-miR-512-5p</td><td>1054~1074</td><td>21</td><td><pre>miRNA: 3' cuUUCACGGGAGUUCCGACUCAc 5'
            ||| :|||: |||||||
Target:5' agAAGCATCCTT--TGCTGAGTt 3'</pre></td><td>-15.00</td><td>153.00</td><td rowspan="2"></td></tr>
<tr><td>1054~1074</td><td>21</td><td><pre>miRNA: 3' cuUUCACGGGAGUUCCGACUCAc 5'
            ||| :|||: |||||||
Target:5' agAAGCATCCTT--TGCTGAGTt 3'</pre></td><td>-15.00</td><td>153.00</td></tr>
<tr><td rowspan="4">269</td><td rowspan="4">hsa-miR-513a-3p</td><td>2254~2277</td><td>24</td><td><pre>miRNA: 3' ggaAGAGUC-UUUCCACUUUAAAu 5'
             |||:|| ||| |:|||||
Target:5' gagTCTTAGTTAAGCAGGAATTTa 3'</pre></td><td>-11.10</td><td>148.00</td><td rowspan="4"></td></tr>
<tr><td>1026~1049</td><td>24</td><td><pre>miRNA: 3' ggAAGAGUCUUUCCA-CUUUAAAu 5'
            | ||: || ||| |:|||||
Target:5' agTACTTTTAATGGTGGGAATTTa 3'</pre></td><td>-10.50</td><td>145.00</td></tr>
<tr><td>2170~2198</td><td>29</td><td><pre>miRNA: 3' ggAAGA--GUCU---UUCC-ACUUUAAAu 5'
            ||:| |||| |:|| |||:|||
Target:5' atTTTTAGCAGAGACAGGGAGGAAGTTTt 3'</pre></td><td>-13.70</td><td>143.00</td></tr>
<tr><td>2254~2277</td><td>24</td><td><pre>miRNA: 3' ggaAGAGUC-UUUCCACUUUAAAu 5'
             |||:|| ||| |:|||||
Target:5' gagTCTTAGTTAAGCAGGAATTTa 3'</pre></td><td>-11.10</td><td>148.00</td></tr>
</table>

<table>
<tr><td></td><td></td><td>1026~1049</td><td>24</td><td>miRNA: 3' ggAAGAGUCUUUCCA-CUUUAAAu 5'
            | ||: || ||| |:|||||
Target:5' agTACTTTTAATGGTGGGAATTTa 3'</td><td>-10.50</td><td>145.00</td><td></td></tr>
<tr><td></td><td></td><td>2170~2198</td><td>29</td><td>miRNA: 3' ggAAGA--GUCU---UUCC-ACUUUAAAu 5'
            ||:| |||| |:|| |||:|||
Target:5' atTTTTAGCAGAGACAGGGAGGAAGTTTt 3'</td><td>-13.70</td><td>143.00</td><td></td></tr>
<tr><td>270</td><td>hsa-miR-515-5p</td><td>1446~1466</td><td>21</td><td>miRNA: 3' guCUUUCACGAAAGAAAACCUCUu 5'
            |||:||| | | ||||:||
Target:5' caGAAGGTG---TATGTTGGGGAg 3'</td><td>-19.70</td><td>147.00</td><td></td></tr>
<tr><td>271</td><td>hsa-miR-515-3p</td><td>428~455</td><td>28</td><td>miRNA: 3' uuGCGAGGUUUU--CU----UCCGUGAg 5'
            :|:| :|||| || |||||:|
Target:5' gtTGTTGTAAAATTGAAATCAGGCATTt 3'</td><td>-11.30</td><td>140.00</td><td></td></tr>
<tr><td>272</td><td>hsa-miR-515-5p</td><td>1446~1466</td><td>21</td><td>miRNA: 3' guCUUUCACGAAAGAAAACCUCUu 5'
            |||:||| | | ||||:||
Target:5' caGAAGGTG---TATGTTGGGGAg 3'</td><td>-19.70</td><td>147.00</td><td></td></tr>
<tr><td>273</td><td>hsa-miR-515-3p</td><td>428~455</td><td>28</td><td>miRNA: 3' uuGCGAGGUUUU--CU----UCCGUGAg 5'
            :|:| :|||| || |||||:|
Target:5' gtTGTTGTAAAATTGAAATCAGGCATTt 3'</td><td>-11.30</td><td>140.00</td><td></td></tr>
<tr><td>274</td><td>hsa-miR-517*</td><td>1737~1762</td><td>26</td><td>miRNA: 3' ucUGUCAC-GA---AGGUAGAUCUCc 5'
            |: ||| || ||: |:|||||
Target:5' ggATTGTGTCTGGCTCTTTTTAGAGc 3'</td><td>-12.00</td><td>140.00</td><td></td></tr>
<tr><td></td><td></td><td>1737~1762</td><td>26</td><td>miRNA: 3' ucUGUCAC-GA---AGGUAGAUCUCc 5'
            |: ||| || ||: |:|||||
Target:5' ggATTGTGTCTGGCTCTTTTTAGAGc 3'</td><td>-12.00</td><td>140.00</td><td></td></tr>
<tr><td>275</td><td>hsa-miR-517b</td><td>2127~2148</td><td>22</td><td>miRNA: 3' uugugaGAUUUCCCUACGUGcu 5'
                ||| ||| ||||||
Target:5' ctgggaCTACAGGCATGCACca 3'</td><td>-12.60</td><td>144.00</td><td></td></tr>
<tr><td>276</td><td>hsa-miR-517*</td><td>1737~1762</td><td>26</td><td>miRNA: 3' ucUGUCAC-GA---AGGUAGAUCUCc 5'
            |: ||| || ||: |:|||||
Target:5' ggATTGTGTCTGGCTCTTTTTAGAGc 3'</td><td>-12.00</td><td>140.00</td><td></td></tr>
<tr><td>277</td><td>hsa-miR-519e</td><td>434~455</td><td>22</td><td>miRNA: 3' uugugaGAUUUUCCUCCGUGAa 5'
                :|:||| |||||:|
Target:5' gtaaaaTTGAAATCAGGCATTt 3'</td><td>-11.20</td><td>140.00</td><td></td></tr>
<tr><td>[illegible]</td><td>hsa-miR-[illegible]</td><td>[illegible]</td><td>[illegible]</td><td>miRNA: 3' ucuUUCAUGAAGGGAGACCUc 5'</td><td>[illegible]</td><td>[illegible]</td><td></td></tr>
</table>

<table>
<tr><th></th><th></th><th></th><th></th><th></th><th></th><th></th><th></th></tr>
<tr><td>278</td><td>520a-5p</td><td>1712~1732</td><td>21</td><td>:| |: | |||||||
Target:5' tctGAATGGTGAACTCTGGAa 3'</td><td>-12.50</td><td>142.00</td><td></td></tr>
<tr><td>279</td><td>hsa-miR-520b</td><td>1013~1033</td><td>21</td><td>miRNA: 3' ggGAGAUUUUCCUUCGUGAAa 5'
|| ||:|| ||:||||
Target:5' acCTATAGAATCCAGTACTTt 3'</td><td>-12.00</td><td>143.00</td><td></td></tr>
<tr><td>280</td><td>hsa-miR-520c-3p</td><td>1012~1033</td><td>22</td><td>miRNA: 3' ugGGAGAUUUUCCUUCGUGAAa 5'
||| ||:|| ||:||||
Target:5' gaCCTATAGAATCCAGTACTTt 3'</td><td>-12.00</td><td>148.00</td><td></td></tr>
<tr><td>281</td><td>hsa-miR-522</td><td>1903~1924</td><td>22</td><td>miRNA: 3' ugugAGAUUUCCCUUGGUAAaa 5'
|:|:||| | ||||||
Target:5' agcaTTTGAAGTGTACCATTgg 3'</td><td>-10.50</td><td>146.00</td><td></td></tr>
<tr><td>282</td><td>hsa-miR-525-5p</td><td>1715~1732</td><td>18</td><td>miRNA: 3' ucUUUCACGUAGGGAGACCUc 5'
| :||| | |||||||
Target:5' gaATGGTGAA---CTCTGGAa 3'</td><td>-13.20</td><td>148.00</td><td></td></tr>
<tr><td>283</td><td>hsa-miR-526b</td><td>118~138</td><td>21</td><td>miRNA: 3' uguCUUUCACGAAGGGAGUUCUc 5'
|||:|| ||||:|:||||
Target:5' gtcGAAGGT--TTCCTTTAAGAt 3'</td><td>-16.30</td><td>144.00</td><td></td></tr>
<tr><td>284</td><td>hsa-miR-526b*</td><td>1013~1034</td><td>22</td><td>miRNA: 3' cgGAGAUUUUCCUUCGUGAAAg 5'
|| ||:|| ||:|||||
Target:5' acCTATAGAATCCAGTACTTTt 3'</td><td>-13.00</td><td>148.00</td><td></td></tr>
<tr><td>285</td><td>hsa-miR-539</td><td>1139~1162</td><td>24</td><td>miRNA: 3' uguguGGUUCCUAU--UAAAGAgg 5'
:::|||||| ||||||
Target:5' gtgttTTGAGGATAGTATTTCTaa 3'</td><td>-11.70</td><td>143.00</td><td></td></tr>
<tr><td>286</td><td>hsa-miR-541*</td><td>1038~1066</td><td>29</td><td>miRNA: 3' ucACCC-UGGCUGUCG---UCUUAGGAAa 5'
|||| |:: ||||: || ||||||
Target:5' ggTGGGAATTTACAGTAGAAGCATCCTTt 3'</td><td>-26.40</td><td>143.00</td><td></td></tr>
<tr><td>287</td><td>hsa-miR-542-3p</td><td>558~577</td><td>20</td><td>miRNA: 3' aaagUCAAUAGUUAGACAGUGu 5'
:| || :| |||||||
Target:5' ttaaGGCTACTA--CTGTCACa 3'</td><td>-12.00</td><td>146.00</td><td></td></tr>
<tr><td rowspan="2">288</td><td rowspan="2">hsa-miR-544</td><td>1426~1451</td><td>26</td><td>miRNA: 3' cuUGAACGAU---UUUU-ACGUCUUa 5'
||| | || |||| |||||||
Target:5' ggACTGGATAGACAAAACTGCAGAAg 3'</td><td>-10.20</td><td>164.00</td><td rowspan="2"></td></tr>
<tr><td>2497~2515</td><td>19</td><td>miRNA: 3' cuUGAACGAUUUUUACGUCUUa 5'
|:|| :| :|||||||
Target:5' taATTTCTT---TGTGCAGAAt 3'</td><td>-10.80</td><td>145.00</td></tr>
</table>

<table>
<tr><td>289</td><td>hsa-miR-544b</td><td>897~917</td><td>21</td><td>miRNA: 3' aaucUUUACGUGUUGGAGUCca 5'
|:|| || :|||||||
Target:5' tttaAGATTCA-GACCTCAGtt 3'</td><td>-11.50</td><td>141.00</td><td></td></tr>
<tr><td rowspan="5">290</td><td rowspan="5">hsa-miR-545*</td><td>688~713</td><td>26</td><td>miRNA: 3' agUAGAU----UAUUUGUAAAUGACu 5'
||:|: || || ||||||||
Target:5' caATTTGGAACATTAAAATTTACTGa 3'</td><td>-8.60</td><td>162.00</td><td rowspan="5"></td></tr>
<tr><td>1236~1260</td><td>25</td><td>miRNA: 3' aguaGAUUAUU---UGUAAAUGACu 5'
||:||:| |:|||||:||
Target:5' agtgCTGATGAGTTATATTTATTGa 3'</td><td>-13.70</td><td>150.00</td></tr>
<tr><td>1990~2011</td><td>22</td><td>miRNA: 3' aguagaUUAUUUGUAAAUGACu 5'
|| ||::||||:|||
Target:5' caaaggAAAAAGTATTTGCTGa 3'</td><td>-11.40</td><td>148.00</td></tr>
<tr><td>1043~1071</td><td>29</td><td>miRNA: 3' agUAGAU----UAUUUGU---AAAUGACu 5'
||:|| | ||:|| |||:|||
Target:5' gaATTTACAGTAGAAGCATCCTTTGCTGa 3'</td><td>-9.50</td><td>142.00</td></tr>
<tr><td rowspan="3">291</td><td rowspan="3">hsa-miR-545</td><td>1990~2011</td><td>22</td><td>miRNA: 3' cgugugUUAUUUACAAACGACu 5'
|| ||:| |||||||
Target:5' caaaggAAAAAGTATTTGCTGa 3'</td><td>-10.00</td><td>160.00</td><td rowspan="3"></td></tr>
<tr><td>1045~1071</td><td>27</td><td>miRNA: 3' cguGUGUUAUUUAC-----AAACGACu 5'
:|||:||:| | |||||||
Target:5' attTACAGTAGAAGCATCCTTTGCTGa 3'</td><td>-13.10</td><td>159.00</td></tr>
<tr><td>402~432</td><td>31</td><td>miRNA: 3' cguGUGUUAUUUA----C-----AAACGACu 5'
|| ||||||| | ||||:||
Target:5' tttCAGAATAAATATTGGTATTTTTTGTTGt 3'</td><td>-7.70</td><td>141.00</td></tr>
<tr><td rowspan="3">292</td><td rowspan="3">hsa-miR-548a-3p</td><td>2518~2541</td><td>24</td><td>miRNA: 3' cguUUUCAUUAAC---GGUCAAAac 5'
:|||| |||| :||||||
Target:5' tttGAAGT-ATTGTATTCAGTTTac 3'</td><td>-8.60</td><td>142.00</td><td rowspan="3"></td></tr>
<tr><td>2518~2541</td><td>24</td><td>miRNA: 3' cguUUUCAUUAAC---GGUCAAAac 5'
:|||| |||| :||||||
Target:5' tttGAAGT-ATTGTATTCAGTTTac 3'</td><td>-8.60</td><td>142.00</td></tr>
<tr><td>2518~2541</td><td>24</td><td>miRNA: 3' cguUUUCAUUAAC---GGUCAAAac 5'
:|||| |||| :||||||
Target:5' tttGAAGT-ATTGTATTCAGTTTac 3'</td><td>-8.60</td><td>142.00</td></tr>
</table>

<table>
<tr><td>293</td><td>hsa-miR-548e</td><td>2517~2541</td><td>25</td><td>miRNA: 3' acguUUUCAU--CA-GAGUCAAAaa 5'
:||||| || :|||||||
Target:5' atttGAAGTATTGTATTCAGTTTac 3'</td><td>-8.60</td><td>144.00</td><td></td></tr>
<tr><td rowspan="4">294</td><td rowspan="4">hsa-miR-548i</td><td>2486~2507</td><td>22</td><td>miRNA: 3' ccguuUUAGGCGUUAAUGAAAa 5'
:|||:|:|||| ||||
Target:5' aatgtGATCTGTAATTTCTTTg 3'</td><td>-11.20</td><td>141.00</td><td rowspan="4"></td></tr>
<tr><td>2486~2507</td><td>22</td><td>miRNA: 3' ccguuUUAGGCGUUAAUGAAAa 5'
:|||:|:|||| ||||
Target:5' aatgtGATCTGTAATTTCTTTg 3'</td><td>-11.20</td><td>141.00</td></tr>
<tr><td>2486~2507</td><td>22</td><td>miRNA: 3' ccguuUUAGGCGUUAAUGAAAa 5'
:|||:|:|||| ||||
Target:5' aatgtGATCTGTAATTTCTTTg 3'</td><td>-11.20</td><td>141.00</td></tr>
<tr><td>2486~2507</td><td>22</td><td>miRNA: 3' ccguuUUAGGCGUUAAUGAAAa 5'
:|||:|:|||| ||||
Target:5' aatgtGATCTGTAATTTCTTTg 3'</td><td>-11.20</td><td>141.00</td></tr>
<tr><td>295</td><td>hsa-miR-548k</td><td>1016~1034</td><td>19</td><td>miRNA: 3' ucGUUUUAGGCGUUCAUGAAAa 5'
:|:||||| ||||||||
Target:5' taTAGAATCC---AGTACTTTt 3'</td><td>-14.80</td><td>165.00</td><td></td></tr>
<tr><td rowspan="2">296</td><td rowspan="2">hsa-miR-548l</td><td>1016~1034</td><td>19</td><td>miRNA: 3' cuGUUUUGGGCGUUUAUGAAAa 5'
:|:||:|| |:||||||
Target:5' taTAGAATCC---AGTACTTTt 3'</td><td>-13.00</td><td>145.00</td><td rowspan="2"></td></tr>
<tr><td>1475~1493</td><td>19</td><td>miRNA: 3' cugUUUUGGGCGUUUAUGAAaa 5'
||||| ||||||||
Target:5' gggAAAAC---AAAATACTTga 3'</td><td>-8.80</td><td>140.00</td></tr>
<tr><td rowspan="2">297</td><td rowspan="2">hsa-miR-548m</td><td>384~405</td><td>22</td><td>miRNA: 3' guUUUUG-GUGUUUAUGGAAAc 5'
::||| :||:| |:|||||
Target:5' ttGGAACATACGATTGCCTTTc 3'</td><td>-11.00</td><td>147.00</td><td rowspan="2"></td></tr>
<tr><td>1017~1035</td><td>19</td><td>miRNA: 3' guUUUUGGUGUUUAUGGAAAc 5'
|:||:| | |:|||:|||
Target:5' atAGAATC-C-AGTACTTTTa 3'</td><td>-7.90</td><td>141.00</td></tr>
<tr><td>298</td><td>hsa-miR-548n</td><td>1017~1035</td><td>19</td><td>miRNA: 3' ugUUUUAGGUGUUAAUGAAAAc 5'
|:||||| | |||||||
Target:5' atAGAATCC---AGTACTTTTa 3'</td><td>-12.00</td><td>161.00</td><td></td></tr>
<tr><td></td><td></td><td>2285~2305</td><td>21</td><td>miRNA: 3' uuUCAUUGACGUCAAAAACGAu 5'
[illegible]</td><td>-8.80</td><td>155.00</td><td></td></tr>
</table>

<table>
<tr><td rowspan="2">299</td><td rowspan="2">hsa-miR-548p</td><td></td><td></td><td> | | ||| | ||||||||
Target:5' ccATTTCCTG-ACTTTTTGCTt 3'</td><td></td><td></td><td rowspan="2"></td></tr>
<tr><td>410~431</td><td>22</td><td>miRNA: 3' uuUCAUUGACGUCAAAAACGAu 5'
  | || : |:| ||||||:|
Target:5' taAATATTGGTATTTTTTGTTg 3'</td><td>-7.20</td><td>144.00</td></tr>
<tr><td rowspan="3">300</td><td rowspan="3">hsa-miR-548u</td><td>574~594</td><td>21</td><td>miRNA: 3' gcGUUUUCAUUAACGUCAGAAAc 5'
  ||:||| || :|||||||
Target:5' caCAGAAG--ATCATAGTCTTTg 3'</td><td>-15.30</td><td>161.00</td><td rowspan="3"></td></tr>
<tr><td>1480~1504</td><td>25</td><td>miRNA: 3' gcGUUUUCAUUAAC---GUCAGAAac 5'
  ||||| || ||| :||||||
Target:5' aaCAAAA-TACTTGACATAGTCTTaa 3'</td><td>-8.20</td><td>148.00</td></tr>
<tr><td>185~215</td><td>31</td><td>miRNA: 3' gcGUUUUCAU-U---AA----CGUCAGAAac 5'
  ::|||||| | || ||||||||
Target:5' ccTGAAAGTACACCTTTCACAGCAGTCTTaa 3'</td><td>-13.40</td><td>143.00</td></tr>
<tr><td>301</td><td>hsa-miR-548x</td><td>2181~2200</td><td>20</td><td>miRNA: 3' acUUUCAUUAACGUCAAAAAu 5'
  | || :| | |||||||
Target:5' agACAGGGA-GGAAGTTTTTa 3'</td><td>-7.80</td><td>146.00</td><td></td></tr>
<tr><td>302</td><td>hsa-miR-548y</td><td>2176~2205</td><td>30</td><td>miRNA: 3' ccGUUUUUGUCACU--------AAUGAAAa 5'
  ||:|:|||| || |||:|||
Target:5' agCAGAGACAGGGAGGAAGTTTTTATTTTt 3'</td><td>-13.80</td><td>146.00</td><td></td></tr>
<tr><td rowspan="2">303</td><td rowspan="2">hsa-miR-549</td><td>64~87</td><td>24</td><td>miRNA: 3' ucuCGA--GUAG-GUAUCAACAGu 5'
   ||| | | |: |||||||
Target:5' cgtGCTACCCCCGCGGAGTTGTCg 3'</td><td>-15.40</td><td>144.00</td><td rowspan="2"></td></tr>
<tr><td>1913~1933</td><td>21</td><td>miRNA: 3' ucucgaGUAGGUAUCAACAGu 5'
      |||: :|||||||:
Target:5' gtgtacCATTGGTAGTTGTTt 3'</td><td>-13.10</td><td>143.00</td></tr>
<tr><td rowspan="2">304</td><td rowspan="2">hsa-miR-551b*</td><td>792~814</td><td>23</td><td>miRNA: 3' ccagagUGGGUG-CGAACUAAAg 5'
      ||:||: :|||||||
Target:5' ttgcagACTCATAATTTGATTTt 3'</td><td>-10.20</td><td>152.00</td><td rowspan="2"></td></tr>
<tr><td>2534~2557</td><td>24</td><td>miRNA: 3' ccaGAGUGGGUGCG--AACUAAAg 5'
   :|:|| ::||: |||:|||
Target:5' cagTTTACATGCGTTATTGGTTTa 3'</td><td>-11.90</td><td>141.00</td></tr>
<tr><td>305</td><td>hsa-miR-561</td><td>198~221</td><td>24</td><td>miRNA: 3' ugAAGU-UCCU-AGAAUUUGAAAc 5'
  |||| || | ||||||| |||
Target:5' ctTTCACAGCAGTCTTAAAGTTTg 3'</td><td>-12.20</td><td>144.00</td><td></td></tr>
</table>

<table>
<tr><td>306</td><td>hsa-miR-562</td><td>362~386</td><td>25</td><td>miRNA: 3' cgUUUACCA--UGU---CGAUGAAa 5'
||:| || |:| |||||||
Target:5' ggAAGTTGTTAATATCTGCTACTTg 3'</td><td>-7.10</td><td>152.00</td><td></td></tr>
<tr><td rowspan="3">307</td><td rowspan="3">hsa-miR-567</td><td>374~395</td><td>22</td><td>miRNA: 3' caAGAC-AGGACCUUCUUGUAUGa 5'
|||| | || | ||||||||
Target:5' taTCTGCTACTTG--GAACATACg 3'</td><td>-14.40</td><td>161.00</td><td rowspan="3"></td></tr>
<tr><td>1537~1558</td><td>22</td><td>miRNA: 3' caaGACAGGACCUUCUUGUAUGa 5'
||| ||| | |||||||
Target:5' ctaCTGGCCTTG-TCAACATACa 3'</td><td>-12.80</td><td>155.00</td></tr>
<tr><td>439~463</td><td>25</td><td>miRNA: 3' caagacaGGACCUU--CUUGUAUGa 5'
:| || | |||||||
Target:5' attgaaaTCAGGCATTTAACATACt 3'</td><td>-8.00</td><td>142.00</td></tr>
<tr><td>308</td><td>hsa-miR-577</td><td>1600~1620</td><td>21</td><td>miRNA: 3' guccauggUUAUAAAAUAGAu 5'
|| |||||||:|
Target:5' tgtaagggAAGATTTTATTTg 3'</td><td>-7.70</td><td>141.00</td><td></td></tr>
<tr><td rowspan="2">309</td><td rowspan="2">hsa-miR-579</td><td>1948~1970</td><td>23</td><td>miRNA: 3' uuagcgccaaAUAUGGUUUACUu 5'
|:|: |||||||
Target:5' tattcataaaTGTGACAAATGAa 3'</td><td>-8.50</td><td>149.00</td><td rowspan="2"></td></tr>
<tr><td>618~640</td><td>23</td><td>miRNA: 3' uuagcgcCAAAUAUGGUUUACUu 5'
||| : : |||||||
Target:5' gtagttcGTTGGGGGCAAATGAa 3'</td><td>-12.50</td><td>148.00</td></tr>
<tr><td>310</td><td>hsa-miR-586</td><td>530~553</td><td>24</td><td>miRNA: 3' ccuGGAU--UUUUAUGUUACGUAu 5'
|||| ||:| : |||||:|
Target:5' cttCCTATGAAGAGGGAATGCGTa 3'</td><td>-12.50</td><td>145.00</td><td></td></tr>
<tr><td rowspan="2">311</td><td rowspan="2">hsa-miR-589*</td><td>74~97</td><td>24</td><td>miRNA: 3' agacCCUUGGCCGUAAACAAGACu 5'
|||:::| |:| |||||||
Target:5' ccgcGGAGTTGTCGTGTGTTCTGg 3'</td><td>-21.30</td><td>168.00</td><td rowspan="2"></td></tr>
<tr><td>2041~2064</td><td>24</td><td>miRNA: 3' agacccuuggccGUAAACAAGACu 5'
:|||||||:||
Target:5' tttatttttattTATTTGTTTTGt 3'</td><td>-9.30</td><td>140.00</td></tr>
<tr><td>312</td><td>hsa-miR-590-3p</td><td>1923~1942</td><td>20</td><td>miRNA: 3' ugAUCGAAUAUGUAUUUUAAu 5'
||| ||:| :||:|||||
Target:5' ggTAG-TTGTTTATGAAATTa 3'</td><td>-9.10</td><td>150.00</td><td></td></tr>
<tr><td></td><td>hsa-</td><td></td><td></td><td></td><td></td><td></td><td></td></tr>
</table>

<table>
<tr><td>313</td><td>hsa-miR-592</td><td>1085~1106</td><td>22</td><td>miRNA: 3' ugUAG-UAGCGUAUAACUGUGUu 5'
  ||| ||| | | ||||:|||
Target:5' ttATCAATCTC-TTTTGATACAa 3'</td><td>-12.40</td><td>147.00</td><td>?</td></tr>
<tr><td>314</td><td>hsa-miR-593</td><td>2167~2185</td><td>19</td><td>miRNA: 3' ucuUUGGGGUCGUCUCUGu 5'
   | ::::|||||||||
Target:5' tgtATTTTTAGCAGAGACa 3'</td><td>-17.80</td><td>156.00</td><td>?</td></tr>
<tr><td>315</td><td>hsa-miR-609</td><td>2268~2287</td><td>20</td><td>miRNA: 3' ucucuacucUCUUUGUGGGa 5'
         | :|||||||
Target:5' caggaatttATGAACACCCa 3'</td><td>-9.40</td><td>143.00</td><td>?</td></tr>
<tr><td>316</td><td>hsa-miR-613</td><td>1064~1084</td><td>21</td><td>miRNA: 3' ccguuuCUUCCU-UGUAAGGa 5'
      ||: | |||||||
Target:5' tttgctGAGTTATACATTCCt 3'</td><td>-11.60</td><td>142.00</td><td>?</td></tr>
<tr><td rowspan="2">317</td><td rowspan="2">hsa-miR-616*</td><td>1130~1149</td><td>20</td><td>miRNA: 3' uucaGUGACUUCCCAAAACUCa 5'
    ||||| | ||||||||
Target:5' aaacCACTG--GTGTTTTGAGg 3'</td><td>-15.80</td><td>162.00</td><td rowspan="2">?</td></tr>
<tr><td>649~671</td><td>23</td><td>miRNA: 3' uuCAG-UGACUUCCCAAAACUca 5'
  ||: |||:| | ||||||
Target:5' ctGTTAACTGGAAGCTTTTGAta 3'</td><td>-8.60</td><td>148.00</td></tr>
<tr><td>318</td><td>hsa-miR-616</td><td>253~275</td><td>23</td><td>miRNA: 3' gaCGAGUUUGGGA-GGUUACUGa 5'
  |: ||| : :| |||||||:
Target:5' aaGTGCAATTATTACCAATGATg 3'</td><td>-9.90</td><td>140.00</td><td>?</td></tr>
<tr><td rowspan="3">319</td><td rowspan="3">hsa-miR-617</td><td>47~65</td><td>19</td><td>miRNA: 3' cgGUG-GAAGUUUACCCUUCAGa 5'
  ||| || |:| |||||||
Target:5' agCACACT--AGA--GGAAGTCg 3'</td><td>-11.90</td><td>148.00</td><td rowspan="3">?</td></tr>
<tr><td>1219~1240</td><td>22</td><td>miRNA: 3' cgguggAAGUUUACCCUUCAga 5'
      | ||:|| ||||||
Target:5' aggaaaTACAGATCGGAAGTgc 3'</td><td>-11.00</td><td>140.00</td></tr>
<tr><td>1264~1283</td><td>20</td><td>miRNA: 3' cgGUGGAAGUUUACCCUUCAga 5'
  || |||: || ||||||
Target:5' ccCAACTTTTAA--GGAAGTgc 3'</td><td>-12.60</td><td>140.00</td></tr>
<tr><td>320</td><td>hsa-miR-622</td><td>283~303</td><td>21</td><td>miRNA: 3' cgaggUUGGA-GUCGUCUGACa 5'
     | ||| || |||||||
Target:5' aataaATCCTGCA-CAGACTGc 3'</td><td>-14.20</td><td>151.00</td><td>?</td></tr>
<tr><td>321</td><td>hsa-miR-[illegible]</td><td>404~424</td><td>21</td><td>miRNA: 3' acUUGUGUUCCAUGACCAUGAu 5'
  |: |:|| ||:|||||:|</td><td>-12.10</td><td>143.00</td><td>?</td></tr>
</table>

<table>
<tr><td></td><td>624*</td><td></td><td></td><td>Target:5' tcAGAATAA-ATATTGGTATTt 3'</td><td></td><td></td><td></td></tr>
<tr><td rowspan="3">322</td><td rowspan="3">hsa-miR-627</td><td>888~908</td><td>21</td><td>miRNA: 3' aggaGAAAAGAAUCUCUGAGUg 5'
|||||:||| |||:|||
Target:5' tttaCTTTTTTTA-AGATTCAg 3'</td><td>-13.40</td><td>157.00</td><td rowspan="3">?</td></tr>
<tr><td>783~803</td><td>21</td><td>miRNA: 3' aggAGAAAAGAAUCUCUGAGUg 5'
||| | ||: |||||||
Target:5' gcaTCTGAT-TTGCAGACTCAt 3'</td><td>-13.30</td><td>154.00</td></tr>
<tr><td>2066~2089</td><td>24</td><td>miRNA: 3' agGAGAAAAGAAUC--UCUGAGug 5'
:|:||||:| || ||||||
Target:5' ttTTTTTTTTTGAGGCAGACTCtc 3'</td><td>-12.70</td><td>150.00</td></tr>
<tr><td rowspan="2">323</td><td rowspan="2">hsa-miR-629*</td><td>1447~1469</td><td>23</td><td>miRNA: 3' cgacCCGAAUGCAA-CCCUCUUg 5'
||: ||:||| |||||||
Target:5' agaaGGTGTATGTTGGGGAGAAc 3'</td><td>-22.10</td><td>166.00</td><td rowspan="2">?</td></tr>
<tr><td>1852~1877</td><td>26</td><td>miRNA: 3' cgaCCCGAAU----GCAACCCUCUUg 5'
|| | || | |||||:|||
Target:5' ataGGCCCTAAGTTCATTGGGGGAAa 3'</td><td>-13.20</td><td>141.00</td></tr>
<tr><td>324</td><td>hsa-miR-637</td><td>26~49</td><td>24</td><td>miRNA: 3' ugCGUCUCGGGCUUUCGGGGGUCa 5'
|::|||| |: :|:||||||
Target:5' gaGTGGAGCGGGGCGGTCCCCAGc 3'</td><td>-24.80</td><td>154.00</td><td>?</td></tr>
<tr><td rowspan="2">325</td><td rowspan="2">hsa-miR-642a</td><td>1~15</td><td>15</td><td>miRNA: 3' guucuguguaAACCUCUCCCUg 5'
||| |||||||
Target:5' -------agaTTGCAGAGGGAg 3'</td><td>-12.40</td><td>152.00</td><td rowspan="2">?</td></tr>
<tr><td>526~547</td><td>22</td><td>miRNA: 3' guucuguGUAAACCUCUCCCUg 5'
| | || |||||||
Target:5' tactcttCCTATGAAGAGGGAa 3'</td><td>-12.20</td><td>151.00</td></tr>
<tr><td>326</td><td>hsa-miR-648</td><td>37~55</td><td>19</td><td>miRNA: 3' ugGUCACGGGACGUGUGAa 5'
|:|| ||| |||||||
Target:5' ggCGGTCCCCAGCACACTa 3'</td><td>-19.80</td><td>165.00</td><td>?</td></tr>
<tr><td>327</td><td>hsa-miR-649</td><td>809~833</td><td>25</td><td>miRNA: 3' cuGAGAACUUGU---UGUGUCCAAa 5'
:|:|| | :| |:|:|||||
Target:5' gaTTTTTAATTAAATATATAGGTTa 3'</td><td>-9.80</td><td>140.00</td><td>?</td></tr>
<tr><td>328</td><td>hsa-miR-654-3p</td><td>830~853</td><td>24</td><td>miRNA: 3' uuccACUAC--CAGUCGUCUGUAu 5'
||||| || | :||||||
Target:5' gttaTGATGAAGTGAATAGACATa 3'</td><td>-14.10</td><td>148.00</td><td>?</td></tr>
</table>

<table>
<tr><td rowspan="1">329</td><td rowspan="1">hsa-miR-655</td><td>1691~1714</td><td>24</td><td>miRNA: 3' uuUCUCCAAUUG--GUACAUAAua 5'
|| | ||:|| | ||||||
Target:5' tcAGTGCTTGACCTCCTGTATTct 3'</td><td>-8.70</td><td>142.00</td><td rowspan="1"></td></tr>
<tr><td rowspan="2">330</td><td rowspan="2">hsa-miR-664</td><td>617~642</td><td>26</td><td>miRNA: 3' acAUC---CGACCCCUAUUUACUUAu 5'
||| |:|||||: ||||||||
Target:5' ggTAGTTCGTTGGGGGCAAATGAATt 3'</td><td>-24.20</td><td>177.00</td><td rowspan="2"></td></tr>
<tr><td>825~847</td><td>23</td><td>miRNA: 3' acAUCCGACCCCUA-UUUACUUAu 5'
||||:| ||| ||:|||||
Target:5' taTAGGTT-ATGATGAAGTGAATa 3'</td><td>-13.40</td><td>148.00</td></tr>
<tr><td rowspan="3">331</td><td rowspan="3">hsa-miR-671-5p</td><td>109~131</td><td>23</td><td>miRNA: 3' gaGGUCGGGGAGGUCCCGAAGGa 5'
||| | : || | ||:||||
Target:5' caCCACCATGTCGAAGGTTTCCt 3'</td><td>-19.00</td><td>145.00</td><td rowspan="3"></td></tr>
<tr><td>1330~1355</td><td>26</td><td>miRNA: 3' gaggUCGGG----GAGGUCCCGAAGGa 5'
||||| | ||| ||||||:
Target:5' gggaAGCCCAGATCACCA-GGCTTCTa 3'</td><td>-19.70</td><td>144.00</td></tr>
<tr><td>2078~2105</td><td>28</td><td>miRNA: 3' gagGUCGGGGAGGU--C---CCGAAGGa 5'
||| |:|||:: | ||||||:
Target:5' aggCAGACTCTCTGTCGCCAGGCTTCTc 3'</td><td>-19.10</td><td>142.00</td></tr>
<tr><td rowspan="3">332</td><td rowspan="3">hsa-miR-7</td><td>165~187</td><td>23</td><td>miRNA: 3' uguUGUUUUAGUGA-UCAGAAGGu 5'
|||||:| ||| ||| ||||
Target:5' cgtACAAAGT-ACTCAGTGTTCCt 3'</td><td>-14.10</td><td>143.00</td><td rowspan="3"></td></tr>
<tr><td>165~187</td><td>23</td><td>miRNA: 3' uguUGUUUUAGUGA-UCAGAAGGu 5'
|||||:| ||| ||| ||||
Target:5' cgtACAAAGT-ACTCAGTGTTCCt 3'</td><td>-14.10</td><td>143.00</td></tr>
<tr><td>165~187</td><td>23</td><td>miRNA: 3' uguUGUUUUAGUGA-UCAGAAGGu 5'
|||||:| ||| ||| ||||
Target:5' cgtACAAAGT-ACTCAGTGTTCCt 3'</td><td>-14.10</td><td>143.00</td></tr>
<tr><td rowspan="2">333</td><td rowspan="2">hsa-miR-708</td><td>2422~2444</td><td>23</td><td>miRNA: 3' gggucgaUCUAACAUUCGAGGAa 5'
| ||||||||:||||
Target:5' gttttgaACATTGTAAGTTCCTa 3'</td><td>-15.40</td><td>156.00</td><td rowspan="2"></td></tr>
<tr><td>220~245</td><td>26</td><td>miRNA: 3' ggGUCG--AUC-UAACAUUCGAGGAa 5'
|||| || ||| |||:||||
Target:5' tgCAGCAGAAGAATTTAAAGTTCCTg 3'</td><td>-13.20</td><td>147.00</td></tr>
</table>

<table>
<tr><td rowspan="2">334</td><td rowspan="2">hsa-miR-761</td><td>225~249</td><td>25</td><td>miRNA: 3' acacagUCAAAGU---GGGACGACg 5'
      | ||| | :|||||||
Target:5' cagaagAATTTAAAGTTCCTGCTGc 3'</td><td>-15.30</td><td>148.00</td><td rowspan="2"></td></tr>
<tr><td>2337~2356</td><td>20</td><td>miRNA: 3' acacagUCAAAGUGGGACGACg 5'
      | ||| |||||||
Target:5' cacaaaAATTT--GCCTGCTGt 3'</td><td>-12.80</td><td>144.00</td></tr>
<tr><td>335</td><td>hsa-miR-764</td><td>762~790</td><td>29</td><td>miRNA: 3' ucCUCCUGUU---C--AC--UCGUGGACg 5'
  || |:||| | || ||||:|||
Target:5' tgGATGGCAATTTGCTTGATAGCATCTGa 3'</td><td>-15.70</td><td>140.00</td><td></td></tr>
<tr><td>336</td><td>hsa-miR-802</td><td>2460~2481</td><td>22</td><td>miRNA: 3' ugUUCCUACUUAGAAACAAUGAc 5'
  ||| | || :|||||||:|
Target:5' acAAGTA-GACATTTTGTTATTa 3'</td><td>-9.00</td><td>148.00</td><td></td></tr>
<tr><td rowspan="3">337</td><td rowspan="3">hsa-miR-873</td><td>226~246</td><td>21</td><td>miRNA: 3' uccUCUGAGUGUUCAAGGACg 5'
   ||| |: |||||||||
Target:5' agaAGAATTTAAAGTTCCTGc 3'</td><td>-17.80</td><td>162.00</td><td rowspan="3"></td></tr>
<tr><td>169~189</td><td>21</td><td>miRNA: 3' uccUC-UGAGUGUUCAAGGACg 5'
   || ||||| : |||||||
Target:5' caaAGTACTCA-GTGTTCCTGa 3'</td><td>-16.40</td><td>157.00</td></tr>
<tr><td>2425~2445</td><td>21</td><td>miRNA: 3' ucCUCUG-AGUGUUCAAGGAcg 5'
  || || |:::||||||||
Target:5' ttGA-ACATTGTAAGTTCCTaa 3'</td><td>-11.40</td><td>142.00</td></tr>
<tr><td>338</td><td>hsa-miR-876-5p</td><td>1618~1639</td><td>22</td><td>miRNA: 3' accacuAAGUGUUUCUUUAGgu 5'
      ||| :||||||||
Target:5' ttgcccTTCCGGAAGAAATCag 3'</td><td>-9.00</td><td>140.00</td><td></td></tr>
<tr><td rowspan="2">339</td><td rowspan="2">hsa-miR-877</td><td>1066~1088</td><td>23</td><td>miRNA: 3' ggGACGCGGUA---GAGGAGAUg 5'
  ||| |::|| :||||:||
Target:5' tgCTGAGTTATACATTCCTTTAt 3'</td><td>-14.30</td><td>142.00</td><td rowspan="2"></td></tr>
<tr><td>1831~1850</td><td>20</td><td>miRNA: 3' ggGACGCGGUAGAGGAGAUg 5'
  :| | :||||:|||||
Target:5' tgTTTCAGTATCTTCTCTAt 3'</td><td>-13.90</td><td>142.00</td></tr>
<tr><td rowspan="2">340</td><td rowspan="2">hsa-miR-877*</td><td>1381~1403</td><td>23</td><td>miRNA: 3' gaCCCUCCUCC--CUCUUCUCCu 5'
  | || |:|| |||||:||
Target:5' caGTGAAGGGGAACAGAAGGGGg 3'</td><td>-17.90</td><td>141.00</td><td rowspan="2"></td></tr>
<tr><td>525~545</td><td>21</td><td>miRNA: 3' gacccuccucccuCUUCUCCu 5'
             |||||||
[illegible]</td><td>-10.40</td><td>140.00</td></tr>
</table>

<table>
<tr><td></td><td></td><td></td><td></td><td>Target:5' atactcttcctatGAAGAGGg 3'</td><td></td><td></td><td></td></tr>
<tr><td>341</td><td>hsa-miR-892b</td><td>1292~1318</td><td>27</td><td>miRNA: 3' agaUGGGU---CU--UUCCUCGGUCac 5'
||||| || ||| ||||||
Target:5' gtcACCCATGTGAATAAGAAGCCAGga 3'</td><td>-17.90</td><td>145.00</td><td>?</td></tr>
<tr><td rowspan="10">342</td><td rowspan="10">hsa-miR-9</td><td>2575~2605</td><td>31</td><td>miRNA: 3' agUAUGU----CGAUCUA----UUGGUUUcu 5'
||||| |:|| || |||||||
Target:5' aaATACATGTTGTTATATTGTAAACCAAAaa 3'</td><td>-8.10</td><td>145.00</td><td rowspan="10">?</td></tr>
<tr><td>923~948</td><td>26</td><td>miRNA: 3' aguAUGUCGA--UCUA-UUGGUUUcu 5'
| |||:| | || |||||||
Target:5' aatTTCAGTTTAATATCAACCAAAaa 3'</td><td>-8.20</td><td>142.00</td></tr>
<tr><td>1511~1534</td><td>24</td><td>miRNA: 3' agUAUGUCGAUCU-AUUGGUUUCu 5'
| :|||:|||| || |||||
Target:5' gaAGGCAGTTAGAGAAAACAAAGt 3'</td><td>-15.50</td><td>141.00</td></tr>
<tr><td>1966~1995</td><td>30</td><td>miRNA: 3' aguaUGUCGAUCUA-----U--UGGUUUCu 5'
||||| :||| | |:|||||
Target:5' atgaACAGCAGGATTATGAATTATCAAAGg 3'</td><td>-14.10</td><td>141.00</td></tr>
<tr><td>2575~2605</td><td>31</td><td>miRNA: 3' agUAUGU----CGAUCUA----UUGGUUUcu 5'
||||| |:|| || |||||||
Target:5' aaATACATGTTGTTATATTGTAAACCAAAaa 3'</td><td>-8.10</td><td>145.00</td></tr>
<tr><td>923~948</td><td>26</td><td>miRNA: 3' aguAUGUCGA--UCUA-UUGGUUUcu 5'
| |||:| | || |||||||
Target:5' aatTTCAGTTTAATATCAACCAAAaa 3'</td><td>-8.20</td><td>142.00</td></tr>
<tr><td>1511~1534</td><td>24</td><td>miRNA: 3' agUAUGUCGAUCU-AUUGGUUUCu 5'
| :|||:|||| || |||||
Target:5' gaAGGCAGTTAGAGAAAACAAAGt 3'</td><td>-15.50</td><td>141.00</td></tr>
<tr><td>1966~1995</td><td>30</td><td>miRNA: 3' aguaUGUCGAUCUA-----U--UGGUUUCu 5'
||||| :||| | |:|||||
Target:5' atgaACAGCAGGATTATGAATTATCAAAGg 3'</td><td>-14.10</td><td>141.00</td></tr>
<tr><td>2575~2605</td><td>31</td><td>miRNA: 3' agUAUGU----CGAUCUA----UUGGUUUcu 5'
||||| |:|| || |||||||
Target:5' aaATACATGTTGTTATATTGTAAACCAAAaa 3'</td><td>-8.10</td><td>145.00</td></tr>
<tr><td>923~948</td><td>26</td><td>miRNA: 3' aguAUGUCGA--UCUA-UUGGUUUcu 5'
| |||:| | || |||||||
Target:5' aatTTCAGTTTAATATCAACCAAAaa 3'</td><td>-8.20</td><td>142.00</td></tr>
</table>

<table>
<tr><td></td><td></td><td>1511~1534</td><td>24</td><td>miRNA: 3' agUAUGUCGAUCU-AUUGGUUUCu 5'
            | :|||:|||| || |||||
Target:5' gaAGGCAGTTAGAGAAAACAAAGt 3'</td><td>-15.50</td><td>141.00</td><td></td></tr>
<tr><td></td><td></td><td>1966~1995</td><td>30</td><td>miRNA: 3' aguaUGUCGAUCUA-----U--UGGUUUCu 5'
              ||||| :||| | |:|||||
Target:5' atgaACAGCAGGATTATGAATTATCAAAGg 3'</td><td>-14.10</td><td>141.00</td><td></td></tr>
<tr><td rowspan="2">343</td><td rowspan="2">hsa-miR-922</td><td>1049~1071</td><td>23</td><td>miRNA: 3' cugCAUCAGGAUAAGAGACGACg 5'
             |||| || ||:|||||
Target:5' acaGTAGAAGCATCCTTTGCTGa 3'</td><td>-13.40</td><td>144.00</td><td rowspan="2"></td></tr>
<tr><td>360~383</td><td>24</td><td>miRNA: 3' cugCAUCAG-GAUAAGAGACGAcg 5'
             | |||: :|| | ||||||
Target:5' ttgGAAGTTGTTAATATCTGCTac 3'</td><td>-9.50</td><td>140.00</td></tr>
<tr><td rowspan="2">344</td><td rowspan="2">hsa-miR-92a</td><td>241~262</td><td>22</td><td>miRNA: 3' ugucCGGCCCUGUUCACGUUAu 5'
              ||:| |||||||||||
Target:5' tcctGCTGCAACAAGTGCAATt 3'</td><td>-19.90</td><td>170.00</td><td rowspan="2"></td></tr>
<tr><td>241~262</td><td>22</td><td>miRNA: 3' ugucCGGCCCUGUUCACGUUAu 5'
              ||:| |||||||||||
Target:5' tcctGCTGCAACAAGTGCAATt 3'</td><td>-19.90</td><td>170.00</td></tr>
<tr><td>345</td><td>hsa-miR-92b</td><td>241~262</td><td>22</td><td>miRNA: 3' ccucCGGCCCUGCUCACGUUAu 5'
              ||:| || ||||||||
Target:5' tcctGCTGCAACAAGTGCAATt 3'</td><td>-14.40</td><td>162.00</td><td></td></tr>
<tr><td>346</td><td>hsa-miR-93</td><td>1011~1034</td><td>24</td><td>miRNA: 3' gaUGGACGUGCUU--GUCGUGAAAc 5'
            |||| :| ||| |||:|||||
Target:5' ggACCTATA-GAATCCAGTACTTTt 3'</td><td>-14.10</td><td>154.00</td><td></td></tr>
<tr><td>347</td><td>hsa-miR-934</td><td>2449~2470</td><td>22</td><td>miRNA: 3' ggucacagaggucaUCAUCUGu 5'
                        |||||||
Target:5' cattttattaaacaAGTAGACa 3'</td><td>-11.20</td><td>140.00</td><td></td></tr>
<tr><td>348</td><td>hsa-miR-936</td><td>1522~1543</td><td>22</td><td>miRNA: 3' gacgcuaaggagggAGAUGACa 5'
                        |||||||
Target:5' gagaaaacaaagtaTCTACTGg 3'</td><td>-7.90</td><td>140.00</td><td></td></tr>
<tr><td>349</td><td>hsa-miR-940</td><td>2092~2112</td><td>21</td><td>miRNA: 3' ccccucgcccccGGGACGGAa 5'
                      :|||||||
Target:5' tcgccaggcttcTCCTGCCTc 3'</td><td>-14.80</td><td>141.00</td><td></td></tr>
</table>

<table>
<tr><td rowspan="2">350</td><td rowspan="2">hsa-miR-944</td><td>469~489</td><td>21</td><td><pre>miRNA: 3' gaGUAGGCUACAUGUUAUUAAa 5'
            || | |: || ||||:|||
Target:5' aaCACCAGGAGT-CAATGATTa 3'</pre></td><td>-9.20</td><td>143.00</td><td rowspan="2">?</td></tr>
<tr><td>2497~2520</td><td>24</td><td><pre>miRNA: 3' gaguAGGCUACAUG--UUAUUAAa 5'
              |:| |||:| |||:|||
Target:5' taatTTCTTTGTGCAGAATGATTt 3'</pre></td><td>-7.60</td><td>140.00</td></tr>
<tr><td>351</td><td>hsa-miR-98</td><td>578~605</td><td>28</td><td><pre>miRNA: 3' uugUUA-UGUU-GA----AUGAUGGAGu 5'
             :|| |:|: || |:|||||||
Target:5' gaaGATCATAGTCTTTGATGCTACCTCa 3'</pre></td><td>-15.50</td><td>149.00</td><td>?</td></tr>
</table>
